# Supplementary material for: Lactate supports cell-autonomous ECM production to sustain metastatic behavior in prostate cancer
Source: EMBO Rep. 2024 Jun 21;25(8):19. doi: 10.1038/s44319-024-00180-z (PMC11315984; doi:10.1038/s44319-024-00180-z)
Supplement: Supplementary file 1 — Appendix [file 44319_2024_180_MOESM1_ESM.pdf]

Appendix Figures for

Lactate supports cell-autonomous ECM production to sustain metastatic behavior in prostate cancer

Luigi Ippolito[1], Assia Duatti[1], Marta Iozzo[1], Giuseppina Comito[1], Elisa Pardella[1], Nicla Lorito[1], Marina Bacci[1], Erica Pranzini[1], Alice Santi[1], Giada Sandrini[2], Carlo V Catapano[2], Sergio Serni[3], Pietro Spatafora[3], Andrea Morandi[1], Elisa Giannoni[1], Paola Chiarugi[1]

Index

Appendix Figure S1……………………………………………………………………..page 2
Appendix Figure S2……………………………………………………………………..page 3
Appendix Figure S3……………………………………………………………………..page 4
Appendix Figure S4……………………………………………………………………..page 5

Appendix Figure S1

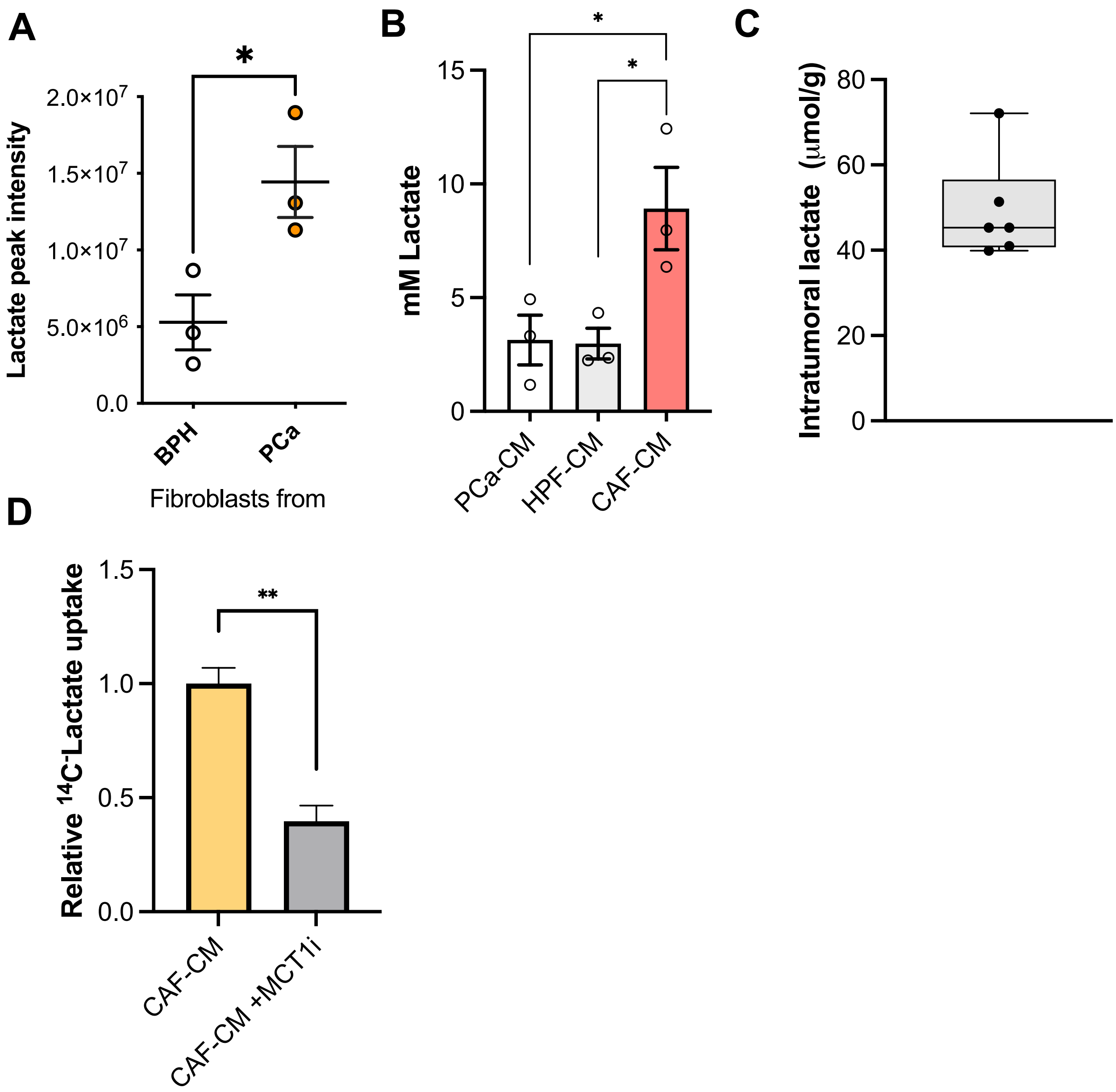

Appendix Figure S1. The main lactate source in PCa is CAFs.

A) GC-MS analysis of extracellular lactate content in fibroblasts isolated from BPH (benign prostatic hyperplasia) and PCa. *n* = 3 specimens. **B**) Levels of lactate measured in the supernatants (CM) from PCa cells, HPFs or CAFs, cultured for 48h in serum-free medium. **C**) Lactate content in DU145 cells-derived primary tumor xenografts. *n* = 6 specimens. **D**) Incorporation of radiolabeled 14C-Lactate in DU145 cells treated with CAF-CM ± MCT1i.

Data information: bar graphs in (**A-D**) represent means ± SEM of *n* = 3 biological replicates, and significance was determined using two-tailed *t* test analysis (**A**,**D**) (*p < 0.05, **p < 0.01), or one-way ANOVA, followed by Tukey's multiple comparisons test (**B**) (*p < 0.05).

Appendix Figure S2

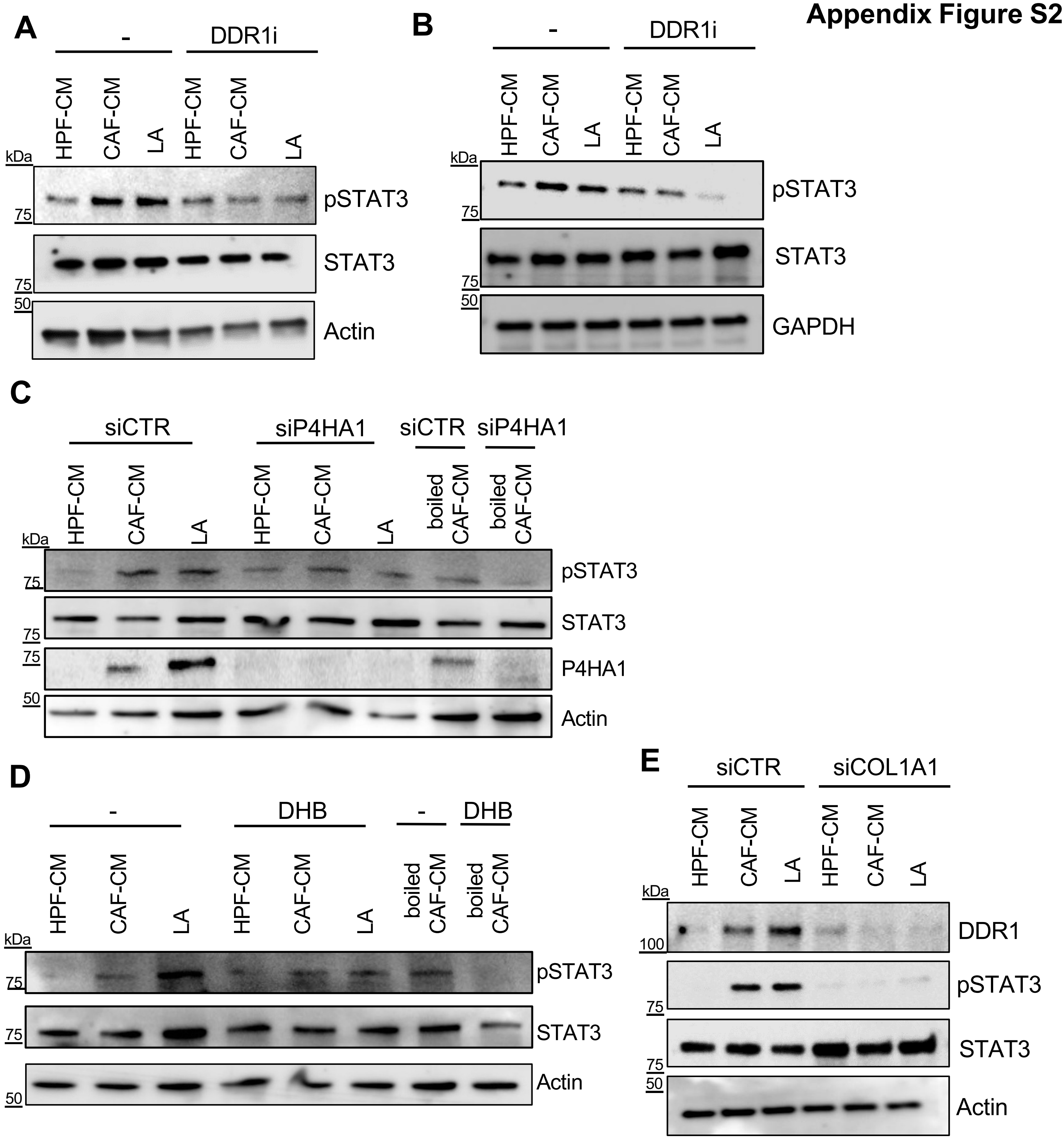

Appendix Figure S2. STAT3 activation underlies the collagen signature in LA-exposed PCa cells.

A-B) Immunoblot for p-STAT3 and STAT3 in DU145 (**A**) and 22Rv1 cells (**B**) treated as indicated, ± DDR1i (500nM). **C-E**) Representative western blot analysis of p-STAT3 and STAT3 in DU145 cells treated as indicated, silenced for P4HA1 (**C**) or treated with DHB (20μM) (**D**) or silenced for COL1A1 (**E**).

Appendix Figure S3

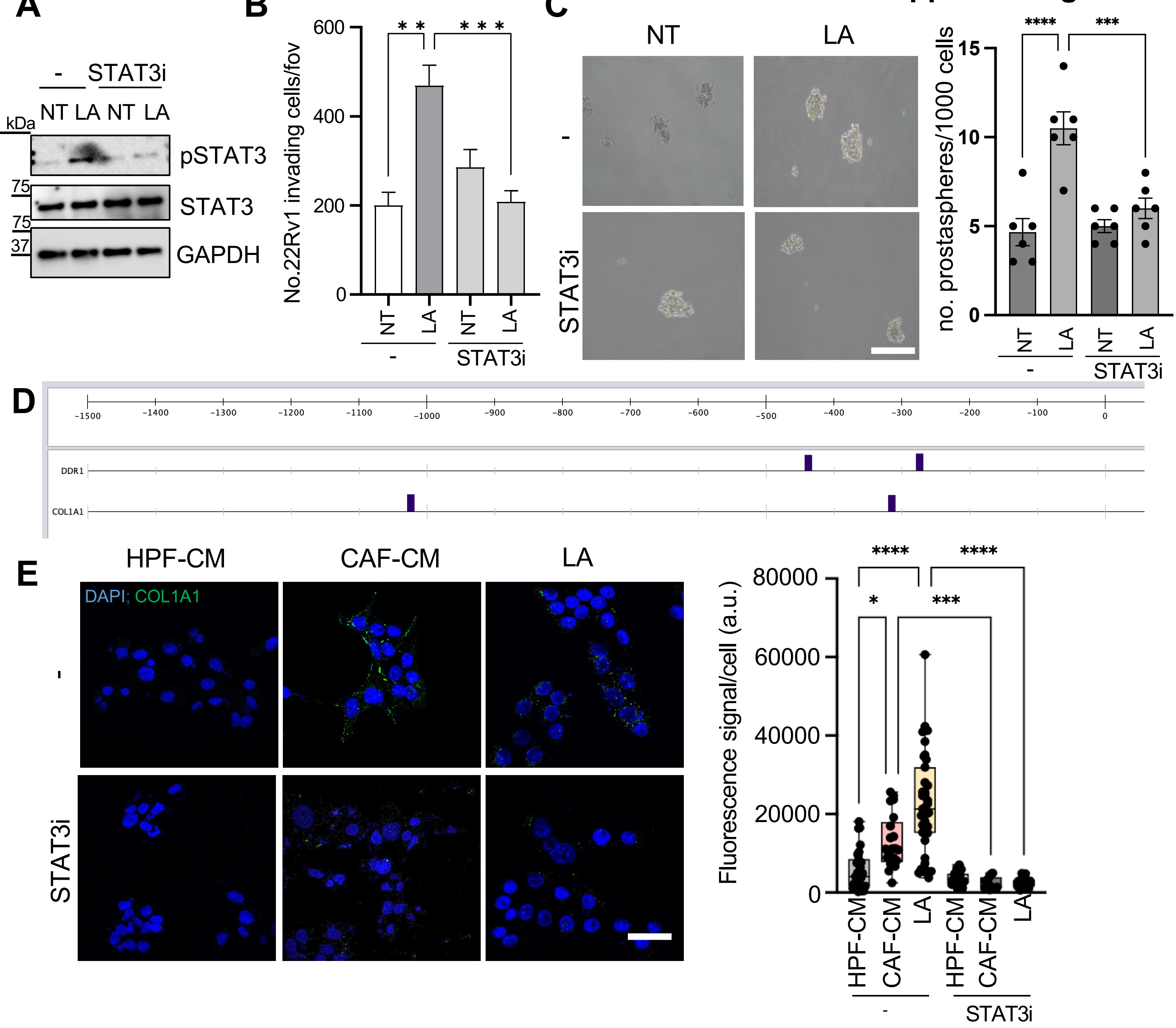

Appendix Figure S3. Targeting STAT3 activity dampens aggressive traits induced by LA in PCa cells.

A) Immunoblot for p-STAT3 and STAT3 in 22Rv1 cells treated as indicated, ± Stattic (2µM, STAT3i). GAPDH was used as loading control. **B)** Invasion assay on 22Rv1 cells treated as indicated, ± STAT3i. **C)** Representative of prostaspheres from 22Rv1 cells treated as indicated, ± STAT3i. The number of the prostaspheres derived from 22Rv1 were quantified and plotted as shown. **D)** Map of predicted sites for STAT3 in the COL1A1 and DDR1 promoters using the JASPAR tool. **E)** Representative pictures of collagen I (green) from immunofluorescence analysis of DU145, treated as indicated ± STAT3i. Quantification plot of fluorescence signal was reported. Nuclei (blue) were stained with DAPI. Scale bar: 10 µm. Box plot showing the quantification of fluorescence signal per cell: centerlines show the medians; box limits indicate the 25th and 75th percentiles; and whiskers extend to the minimum and maximum. *n* = 15-29 cells from 3 biological replicates.

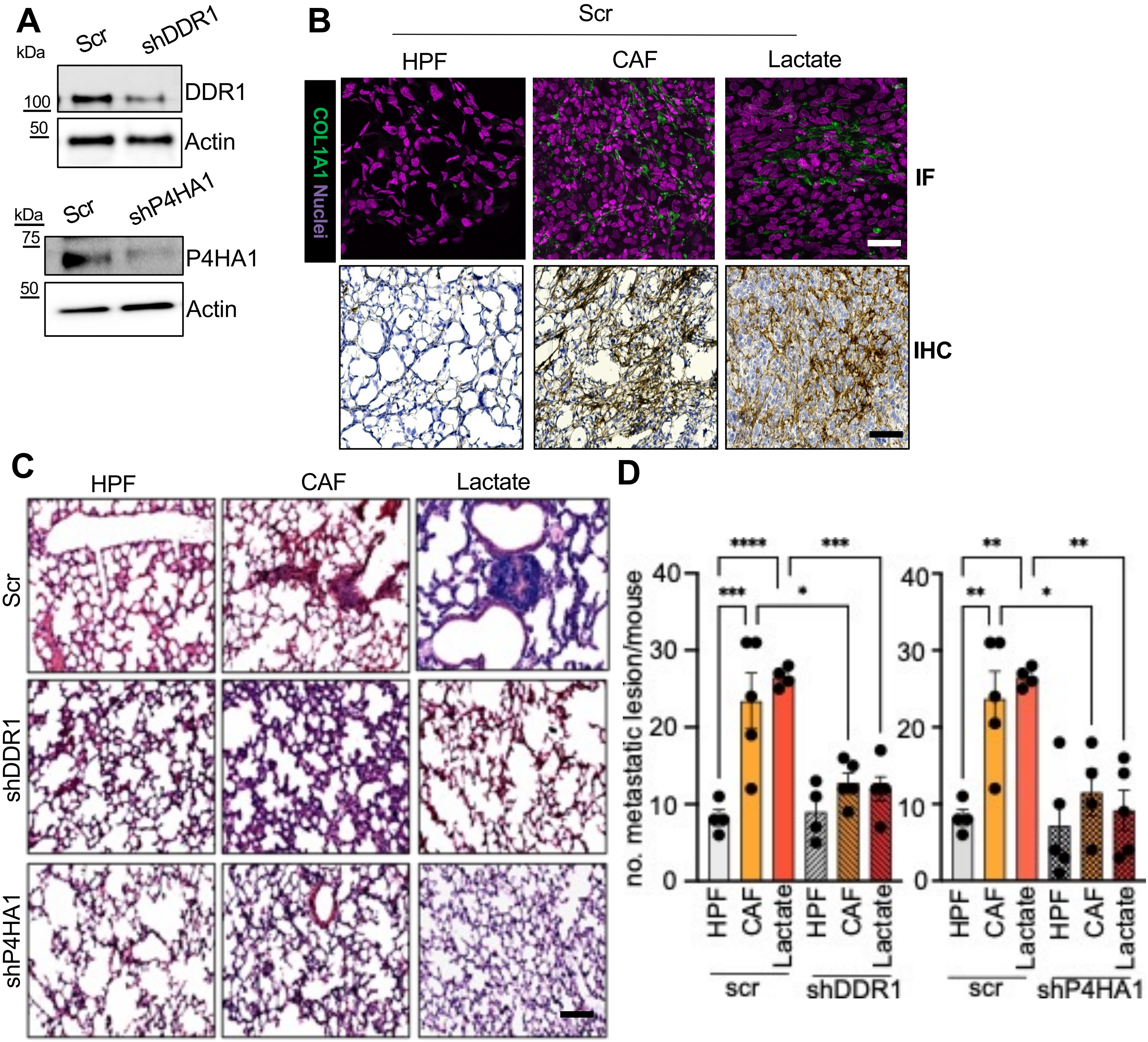

Appendix Figure S4. P4HA1 and DDR1 targeting *in vivo* impairs PCa cell metastatic burden sustained by lactate.

A) Immunoblot for P4HA1 and DDR1 in scrambled (scr) and sh-P4HA1 or sh-DDR1 DU145 cells. Actin was used as loading control. **B)** Representative immunofluorescence and immunohistochemical detection of Col1 in scrambled xenografts tumors. For IHC, scale bar: 100 μm, magnification ×20. For IF, nuclei were counterstained with DAPI (pseudocolor magenta). Scale bar: 10 μm. **C)** Representative tumor metastases in the lung at day 60. Scale bar, 100 μm, magnification ×20. **D)** Analysis of tumor burdens of lung metastasis at day 60 from shP4HA1 or shDDR1 tumor xenografts. Data information: bar graphs in (**D**) represent means ± SEM, n = 4-5 mice/group. Significance was determined using one-way ANOVA, followed by Tukey's multiple comparisons test (*p < 0.05; **p < 0.01; ***p < 0.001; ****p < 0.0001).
