# Supplementary figures and images for: Lactate supports cell-autonomous ECM production to sustain metastatic behavior in prostate cancer (part 1 of 3)
Source: EMBO Rep. 2024 Jun 21;25(8):19. doi: 10.1038/s44319-024-00180-z (PMC11315984; doi:10.1038/s44319-024-00180-z)

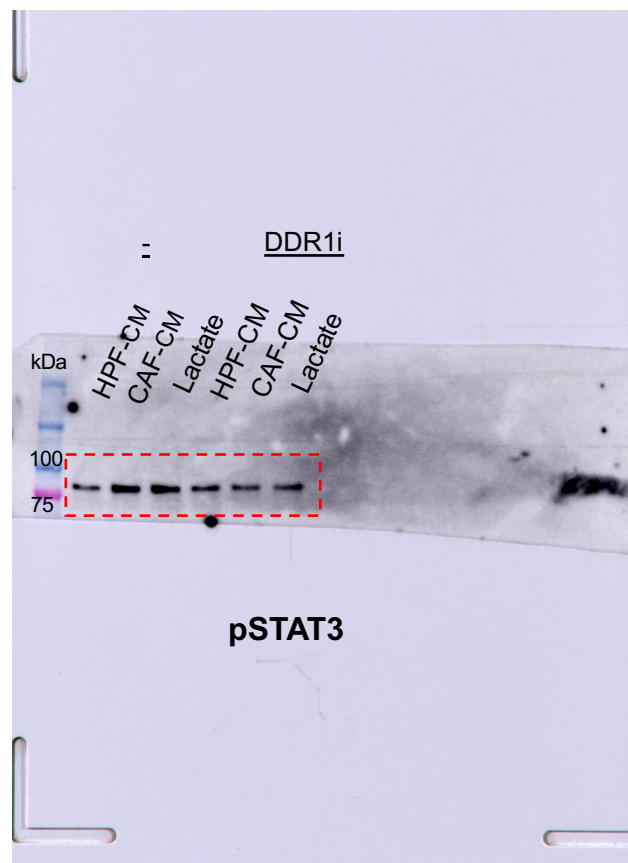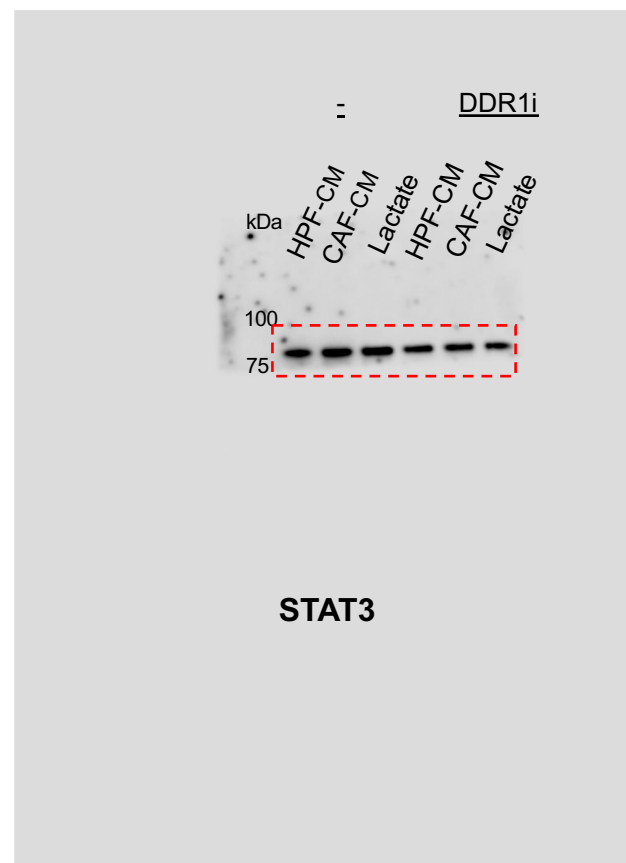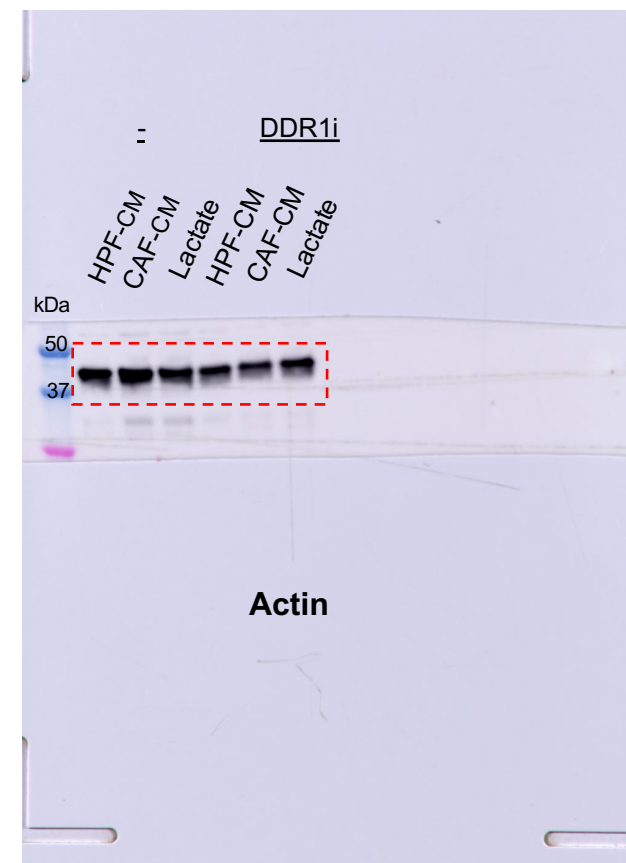

Supplement: Supplementary file 4 — Appendix Figure Source Data [file 44319_2024_180_MOESM4_ESM.zip › Appendix Figure S2/S2A/S2A blot.pdf]

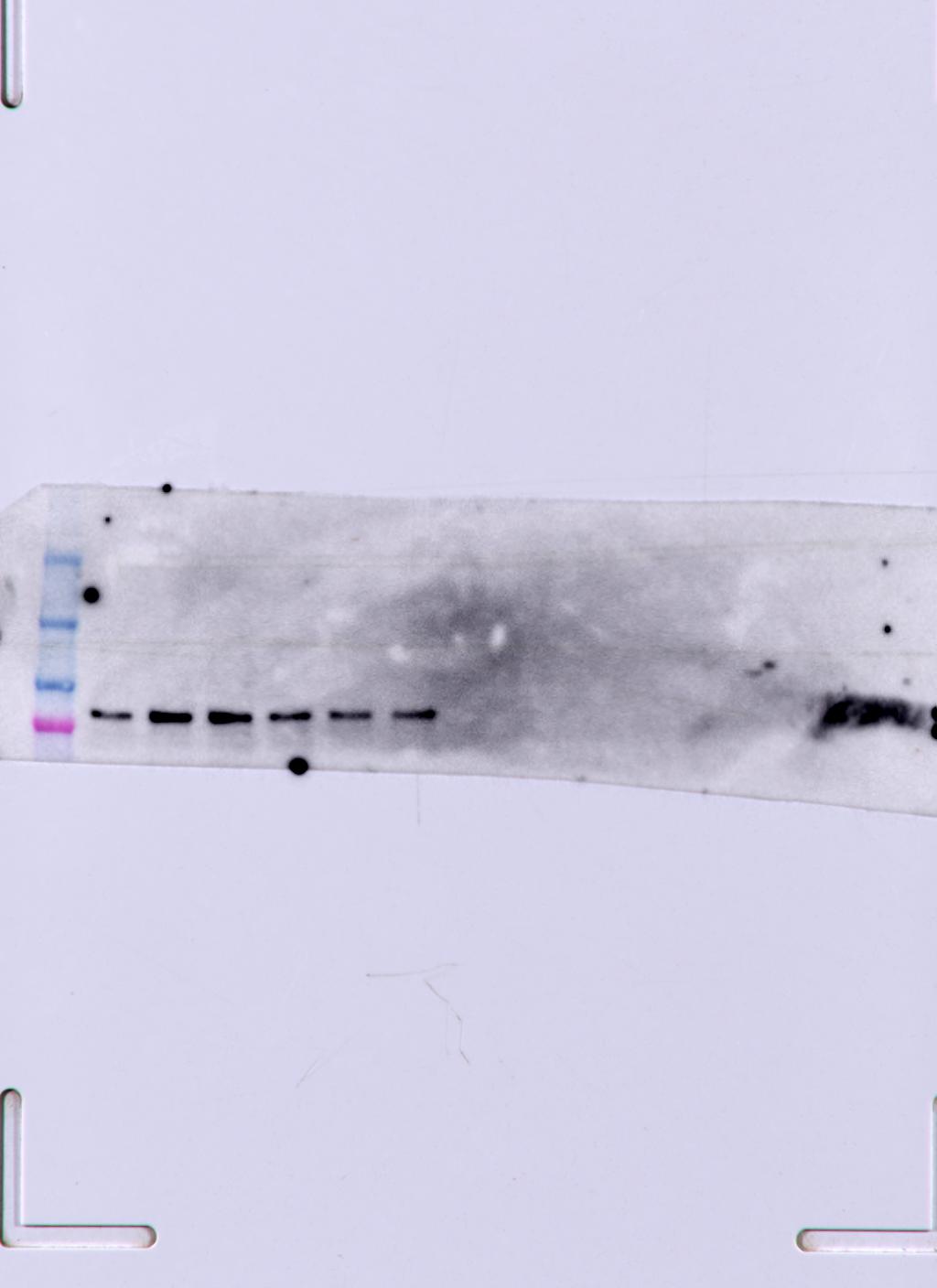

Supplement: Supplementary file 4 — Appendix Figure Source Data [file 44319_2024_180_MOESM4_ESM.zip › Appendix Figure S2/S2A/WB pSTAT3 DDR1i.jpg]

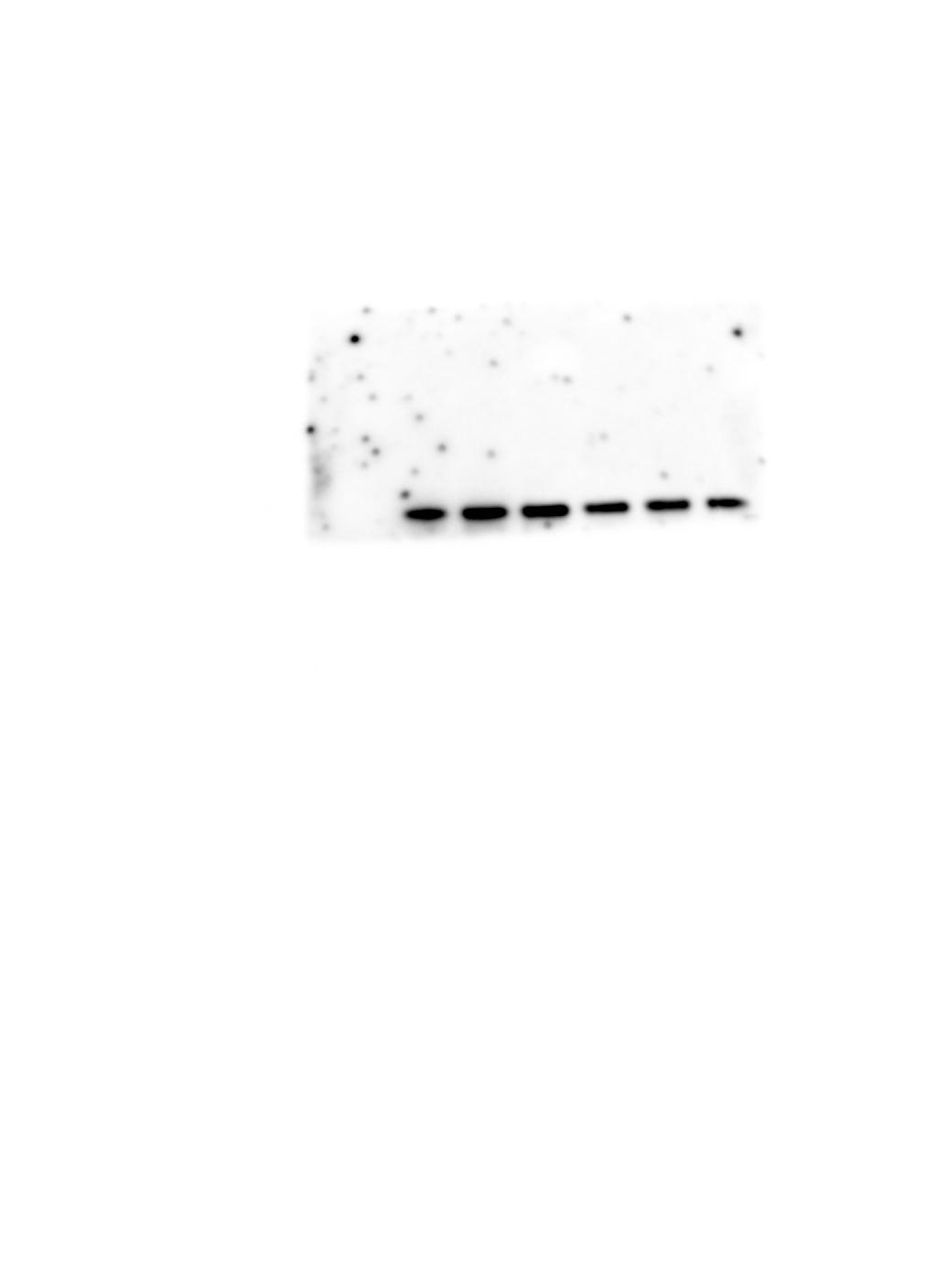

Supplement: Supplementary file 4 — Appendix Figure Source Data [file 44319_2024_180_MOESM4_ESM.zip › Appendix Figure S2/S2A/WB STAT3 DDR1i.jpg]

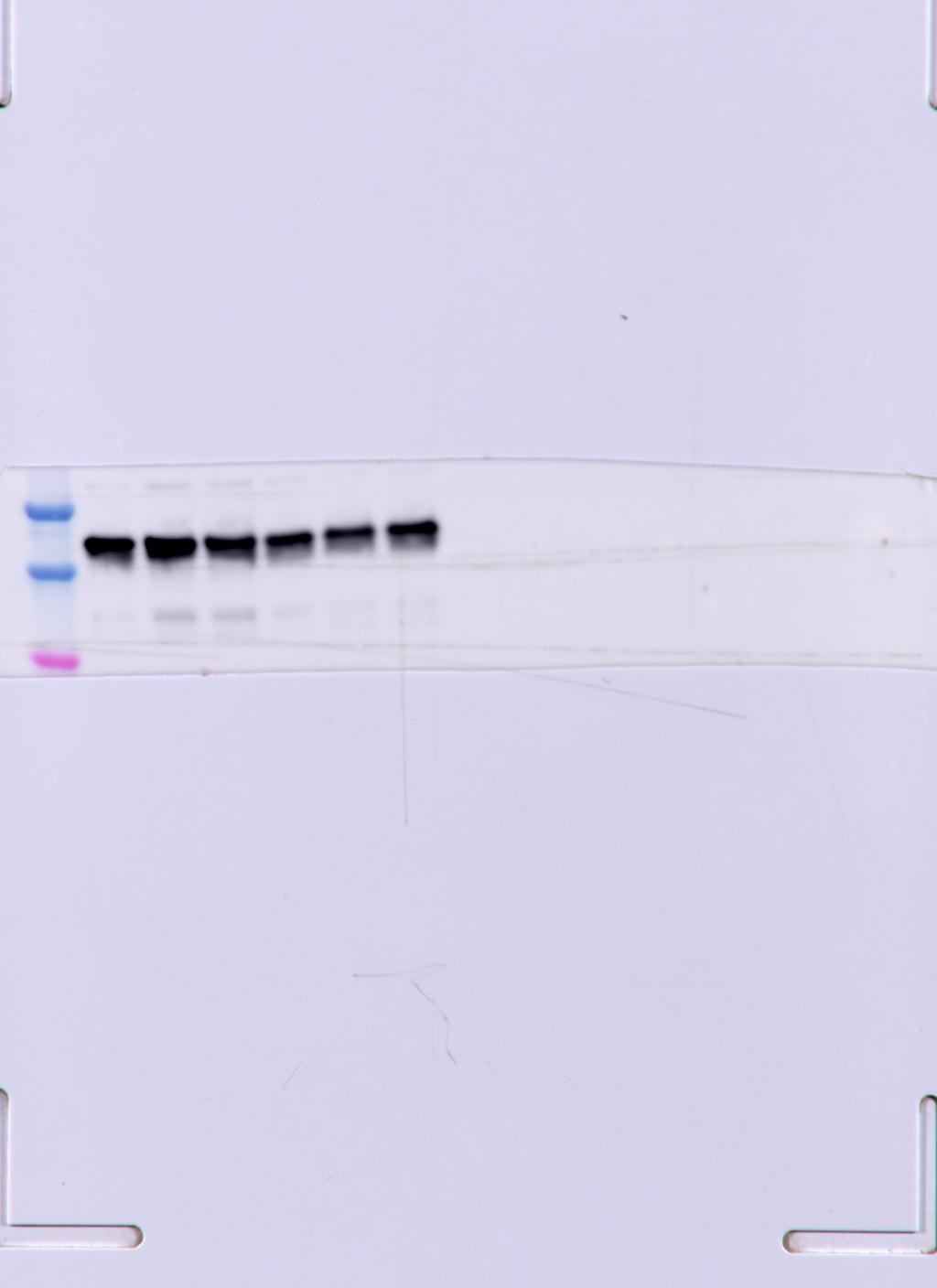

Supplement: Supplementary file 4 — Appendix Figure Source Data [file 44319_2024_180_MOESM4_ESM.zip › Appendix Figure S2/S2A/WB Actin DDR1i.jpg]

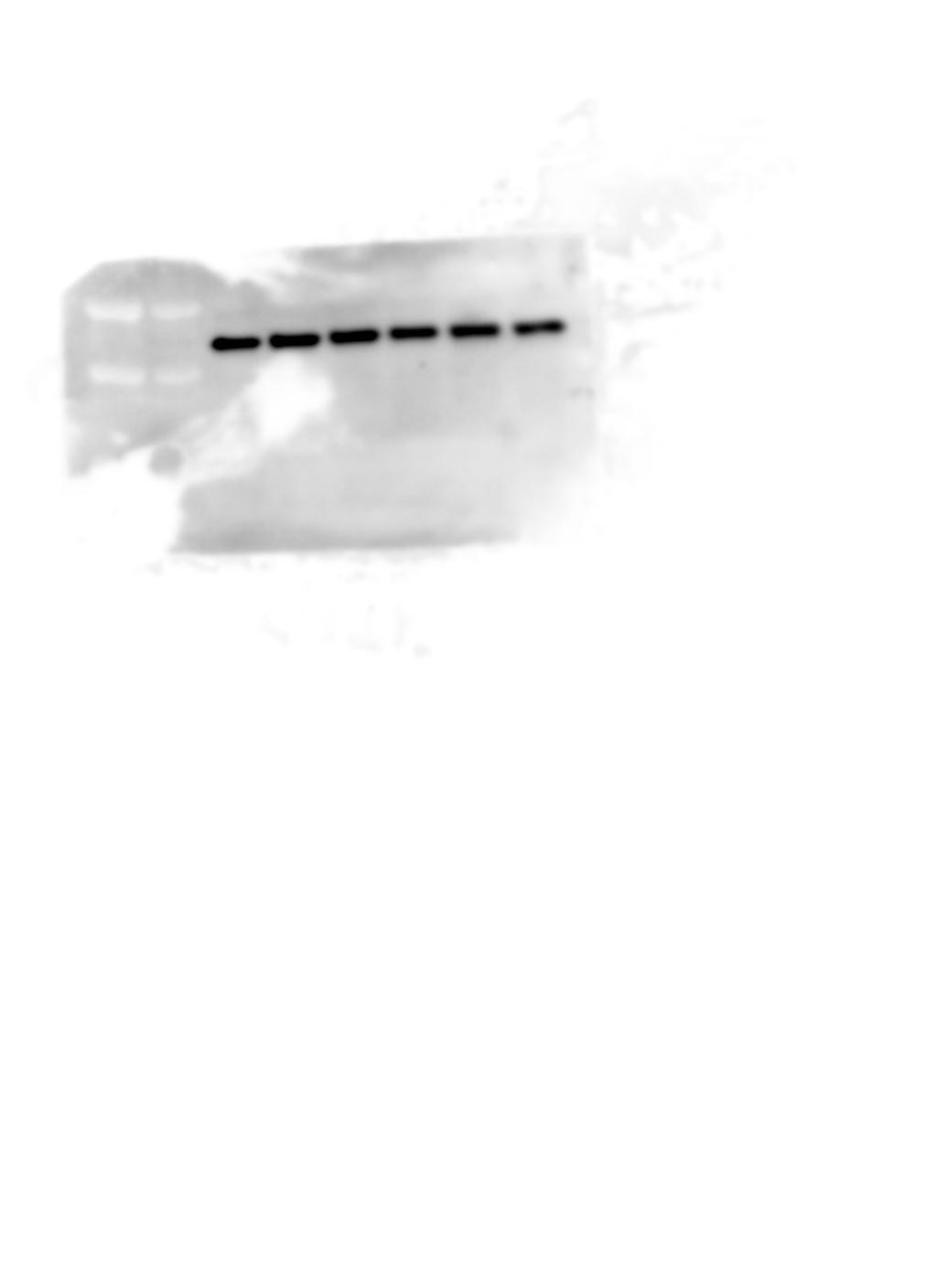

Supplement: Supplementary file 4 — Appendix Figure Source Data [file 44319_2024_180_MOESM4_ESM.zip › Appendix Figure S2/S2E/siCol1a1 Actin.jpg]

## Appendix Figure S2E

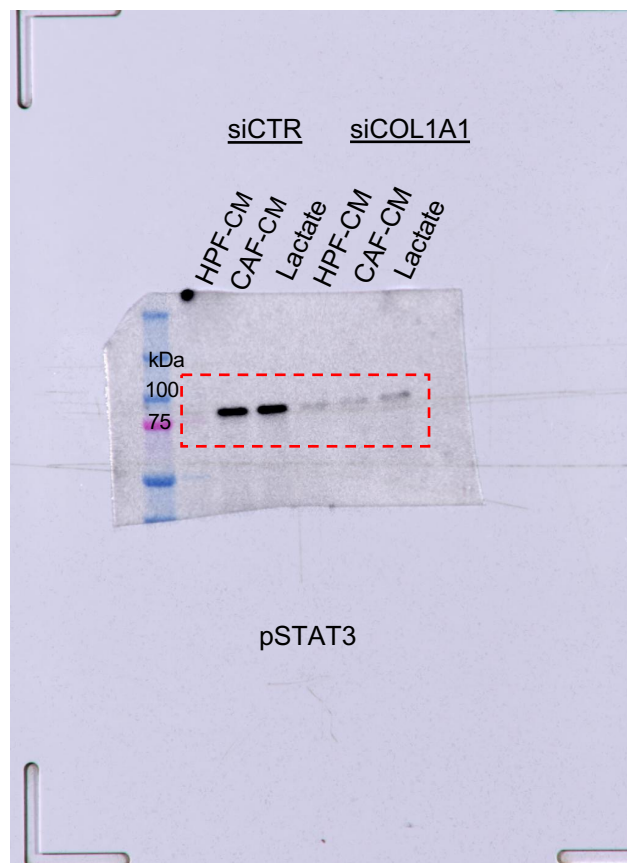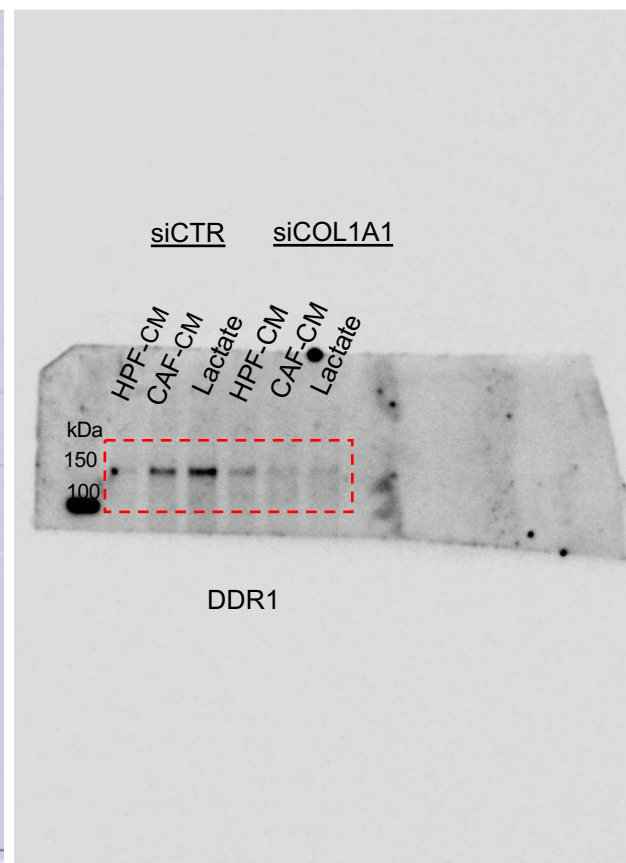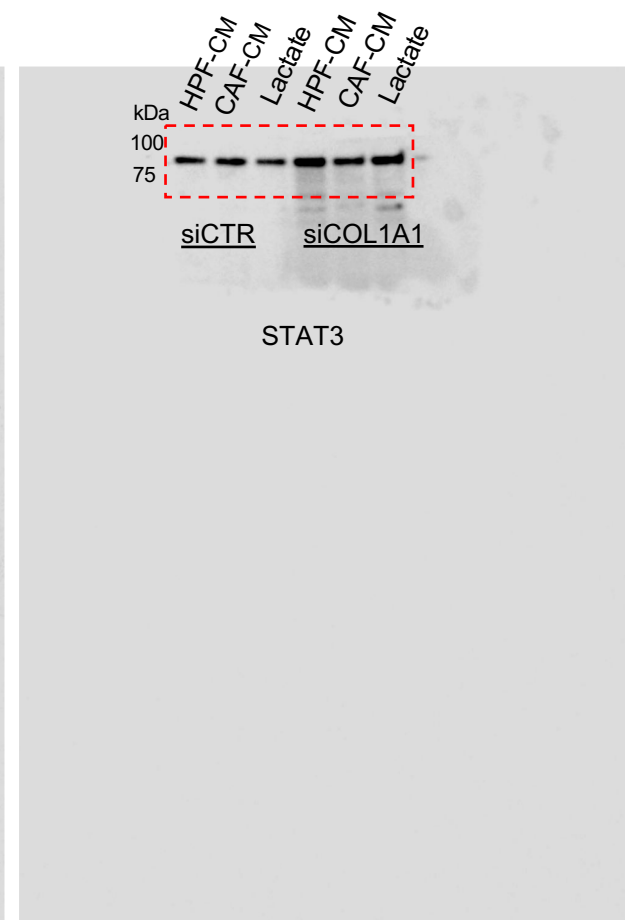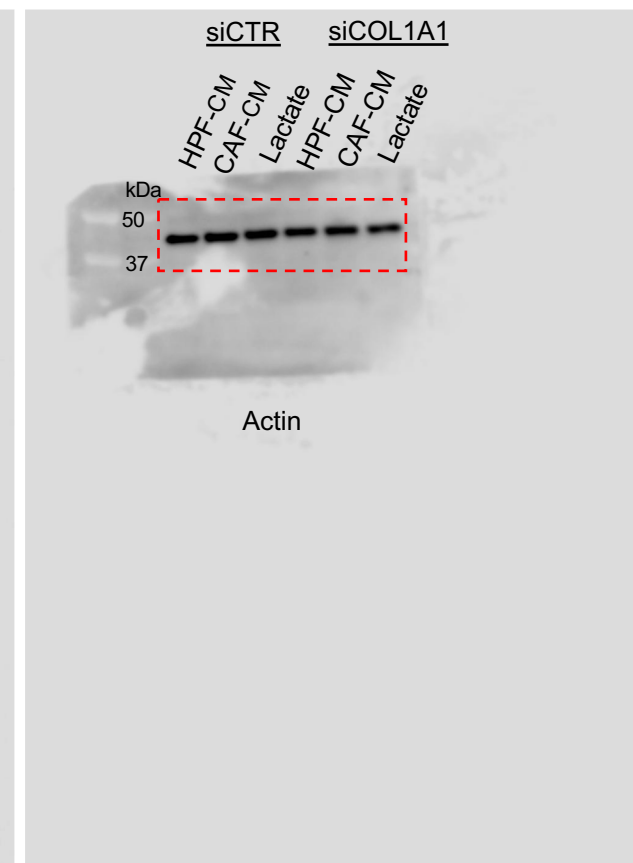

Supplement: Supplementary file 4 — Appendix Figure Source Data [file 44319_2024_180_MOESM4_ESM.zip › Appendix Figure S2/S2E/S2E blot.pdf]

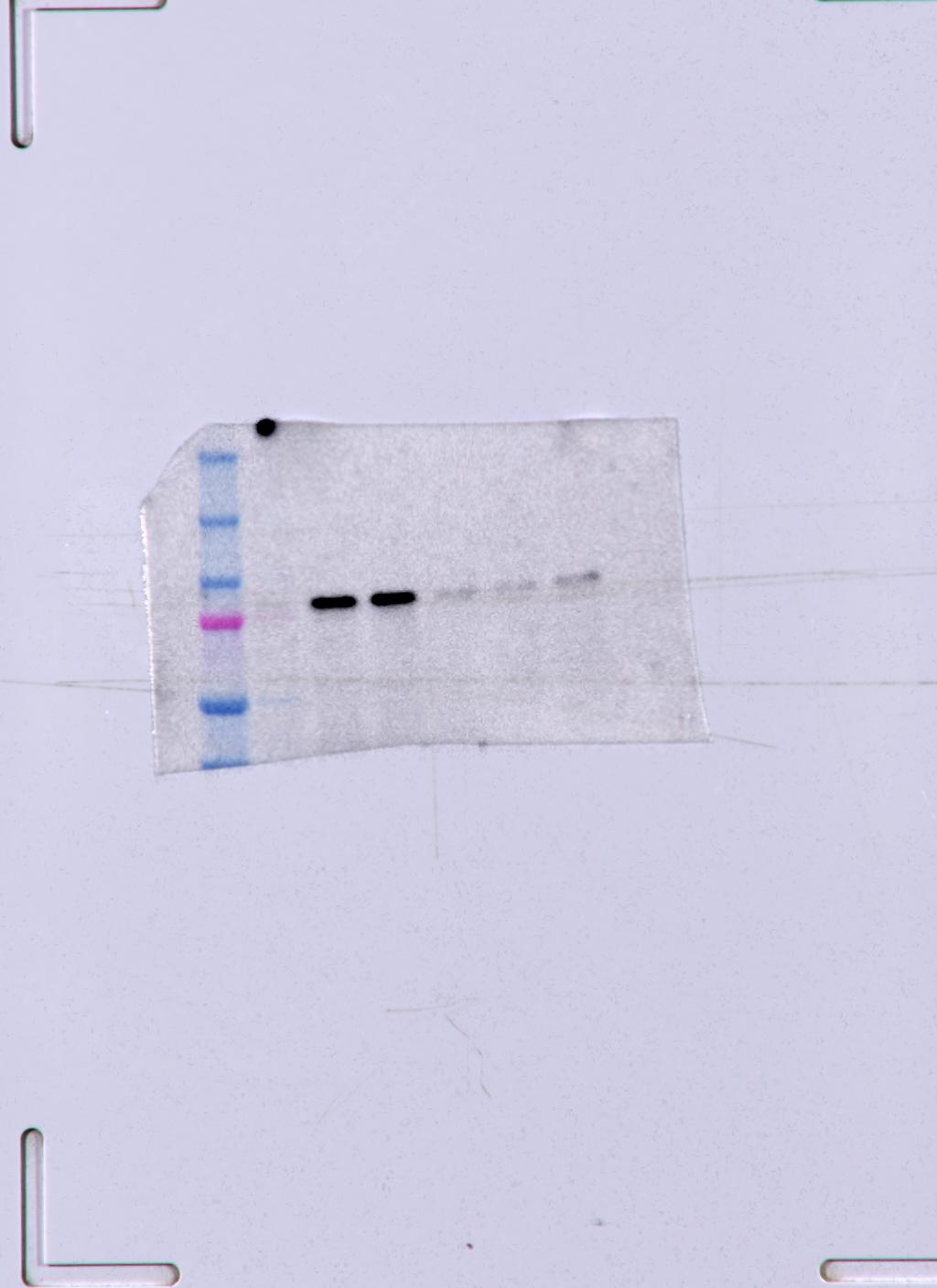

Supplement: Supplementary file 4 — Appendix Figure Source Data [file 44319_2024_180_MOESM4_ESM.zip › Appendix Figure S2/S2E/siCol1a1 pSTAT3.jpg]

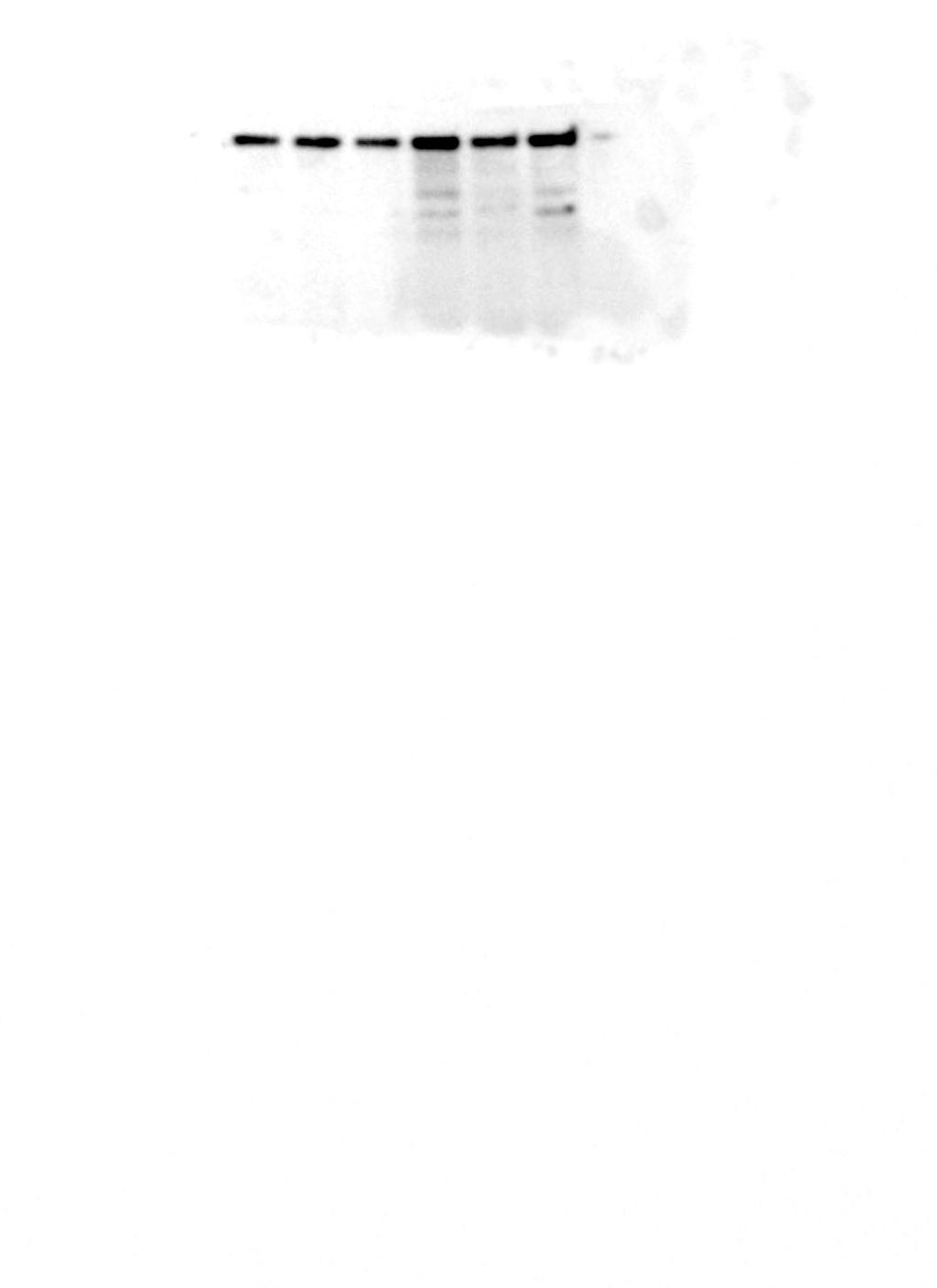

Supplement: Supplementary file 4 — Appendix Figure Source Data [file 44319_2024_180_MOESM4_ESM.zip › Appendix Figure S2/S2E/siCol1a1 STAT3.jpg]

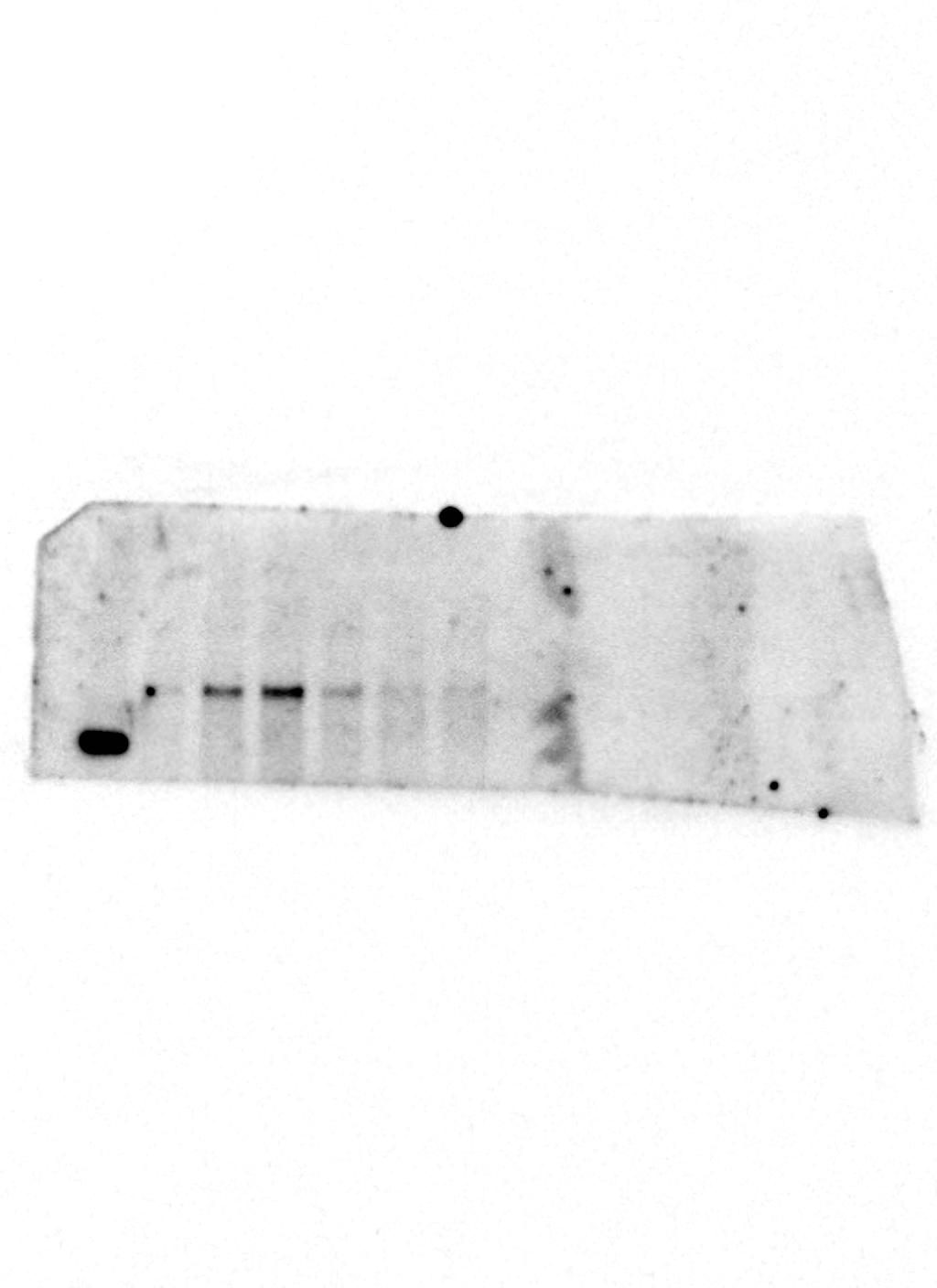

Supplement: Supplementary file 4 — Appendix Figure Source Data [file 44319_2024_180_MOESM4_ESM.zip › Appendix Figure S2/S2E/siCol1a1 DDR1.jpg]

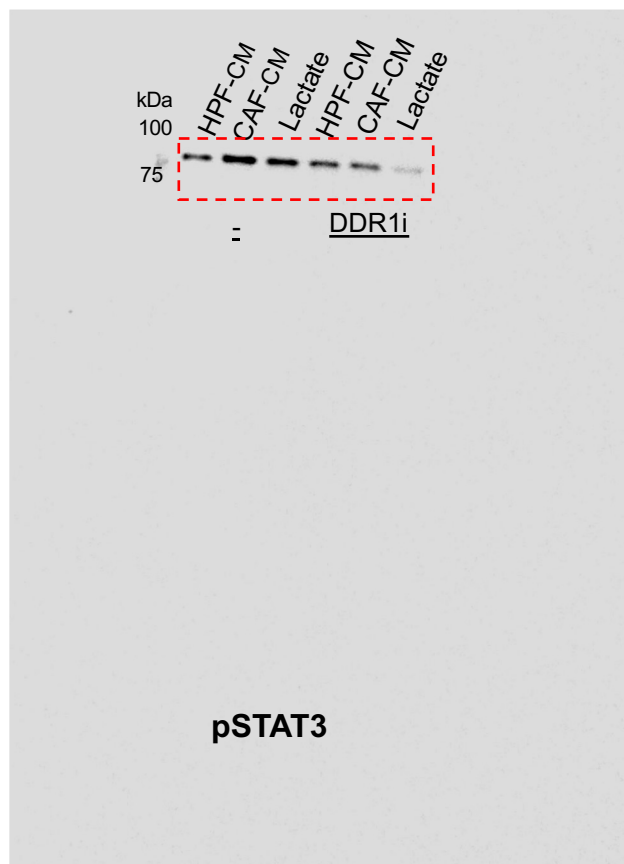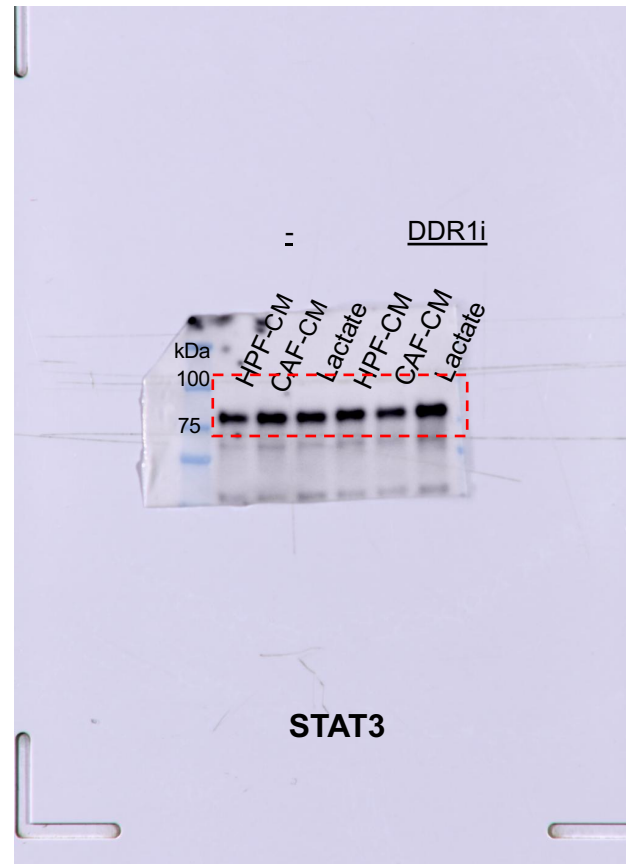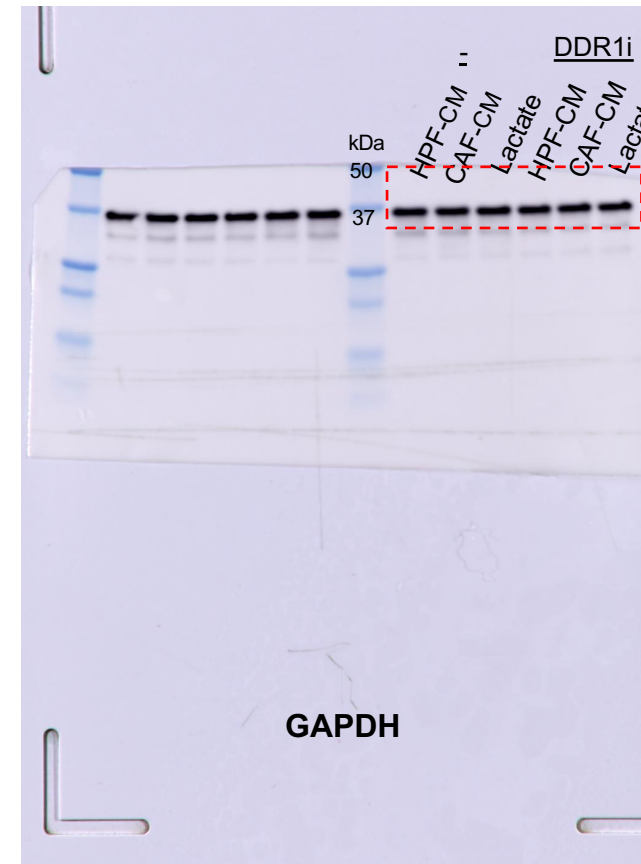

Supplement: Supplementary file 4 — Appendix Figure Source Data [file 44319_2024_180_MOESM4_ESM.zip › Appendix Figure S2/S2B/S2B blot.pdf]

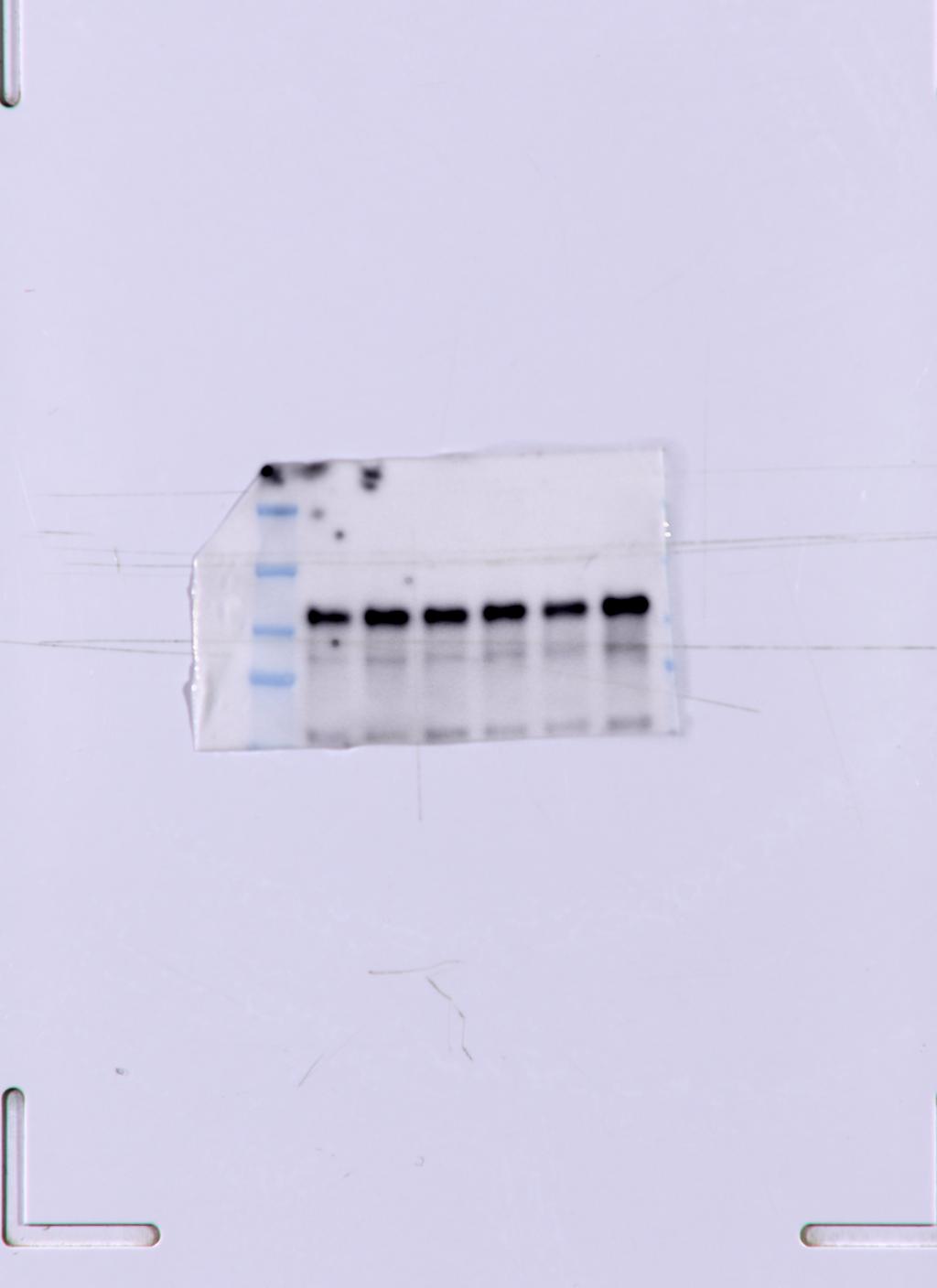

Supplement: Supplementary file 4 — Appendix Figure Source Data [file 44319_2024_180_MOESM4_ESM.zip › Appendix Figure S2/S2B/WB STAT3 22rv1.jpg]

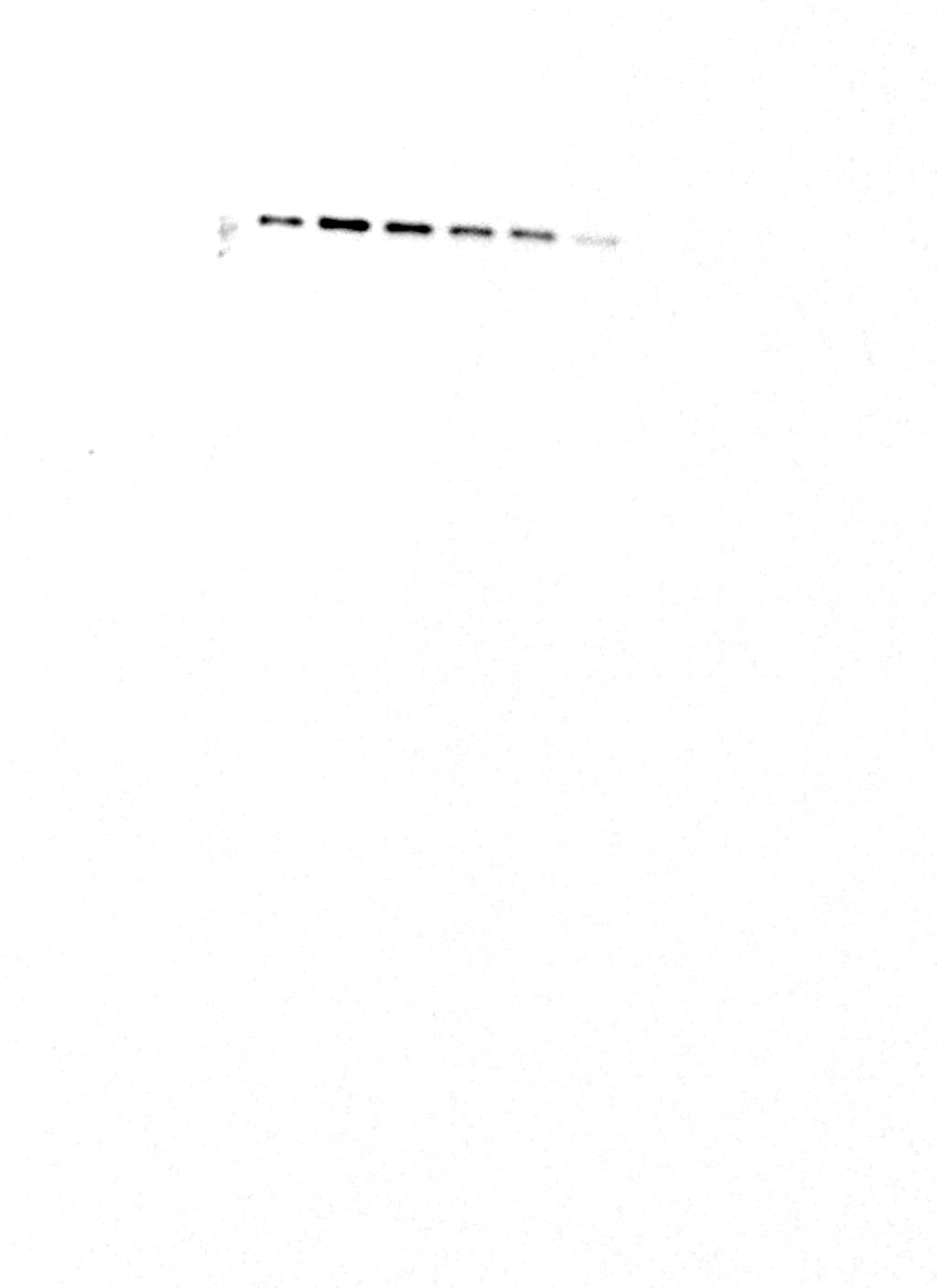

Supplement: Supplementary file 4 — Appendix Figure Source Data [file 44319_2024_180_MOESM4_ESM.zip › Appendix Figure S2/S2B/WB pSTAT3 22rv1 .jpg]

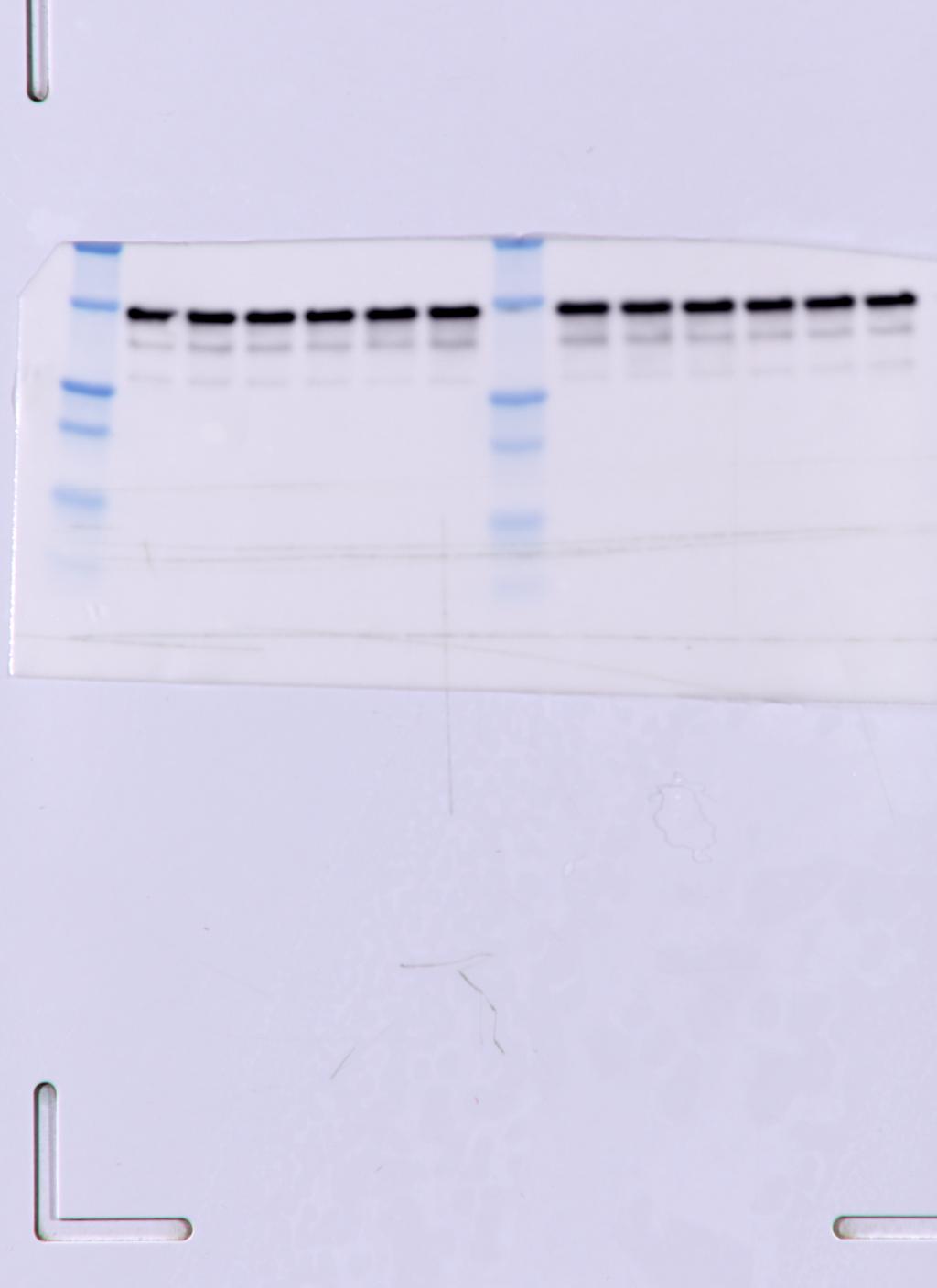

Supplement: Supplementary file 4 — Appendix Figure Source Data [file 44319_2024_180_MOESM4_ESM.zip › Appendix Figure S2/S2B/WB GAPDH_22rv1.jpg]

# Appendix Figure S2C

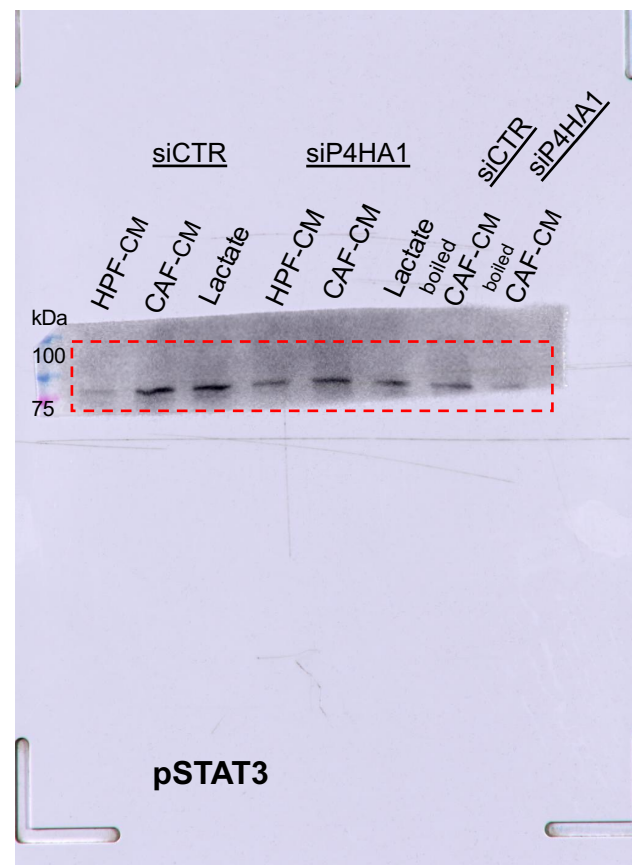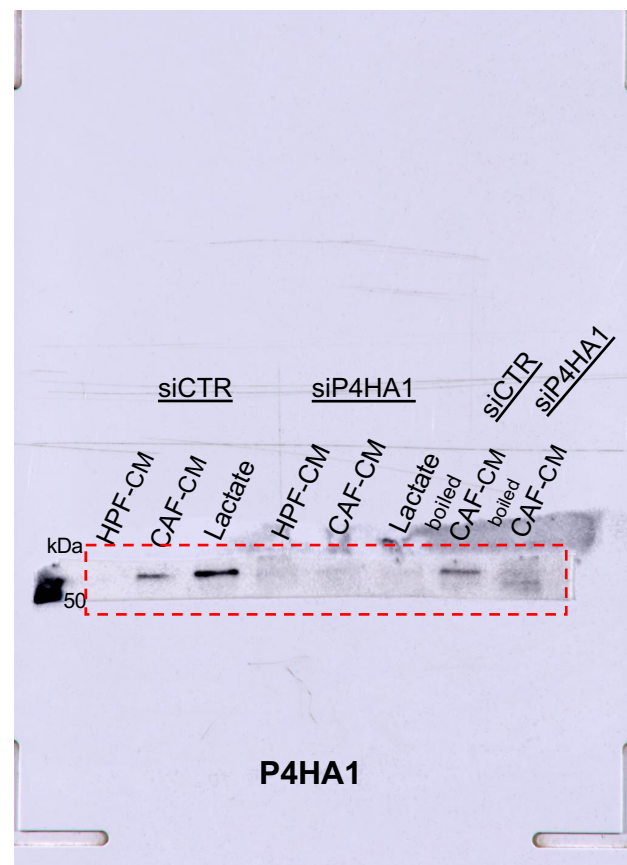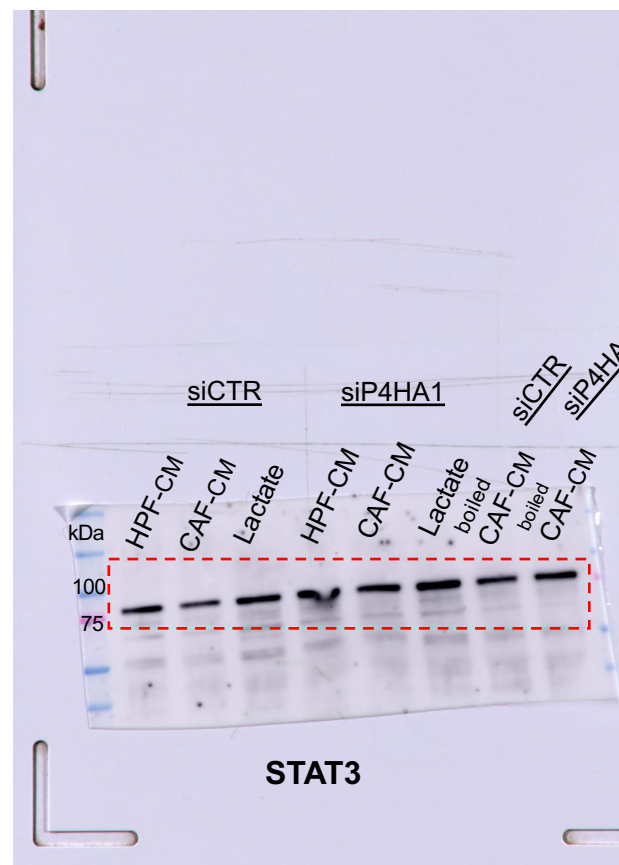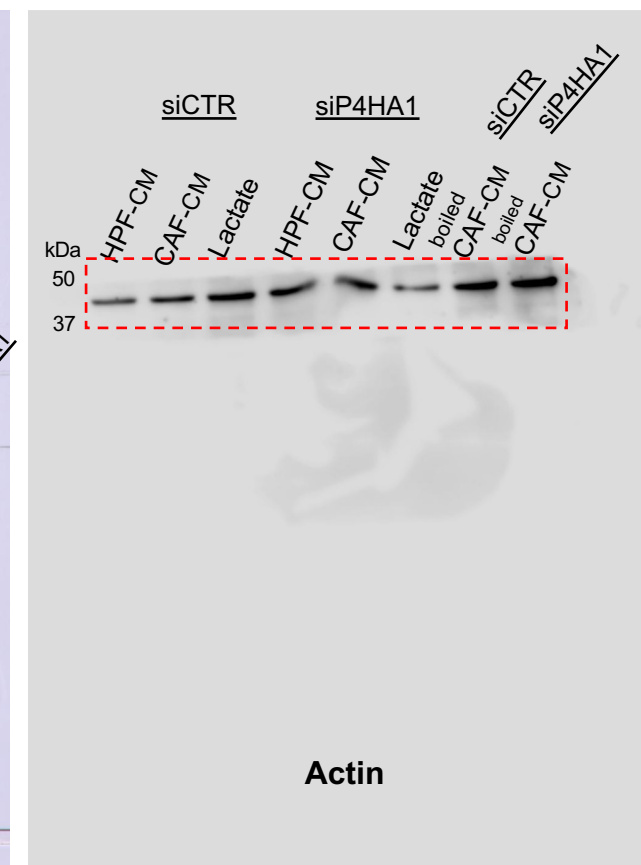

Supplement: Supplementary file 4 — Appendix Figure Source Data [file 44319_2024_180_MOESM4_ESM.zip › Appendix Figure S2/S2C/S2C blot.pdf]

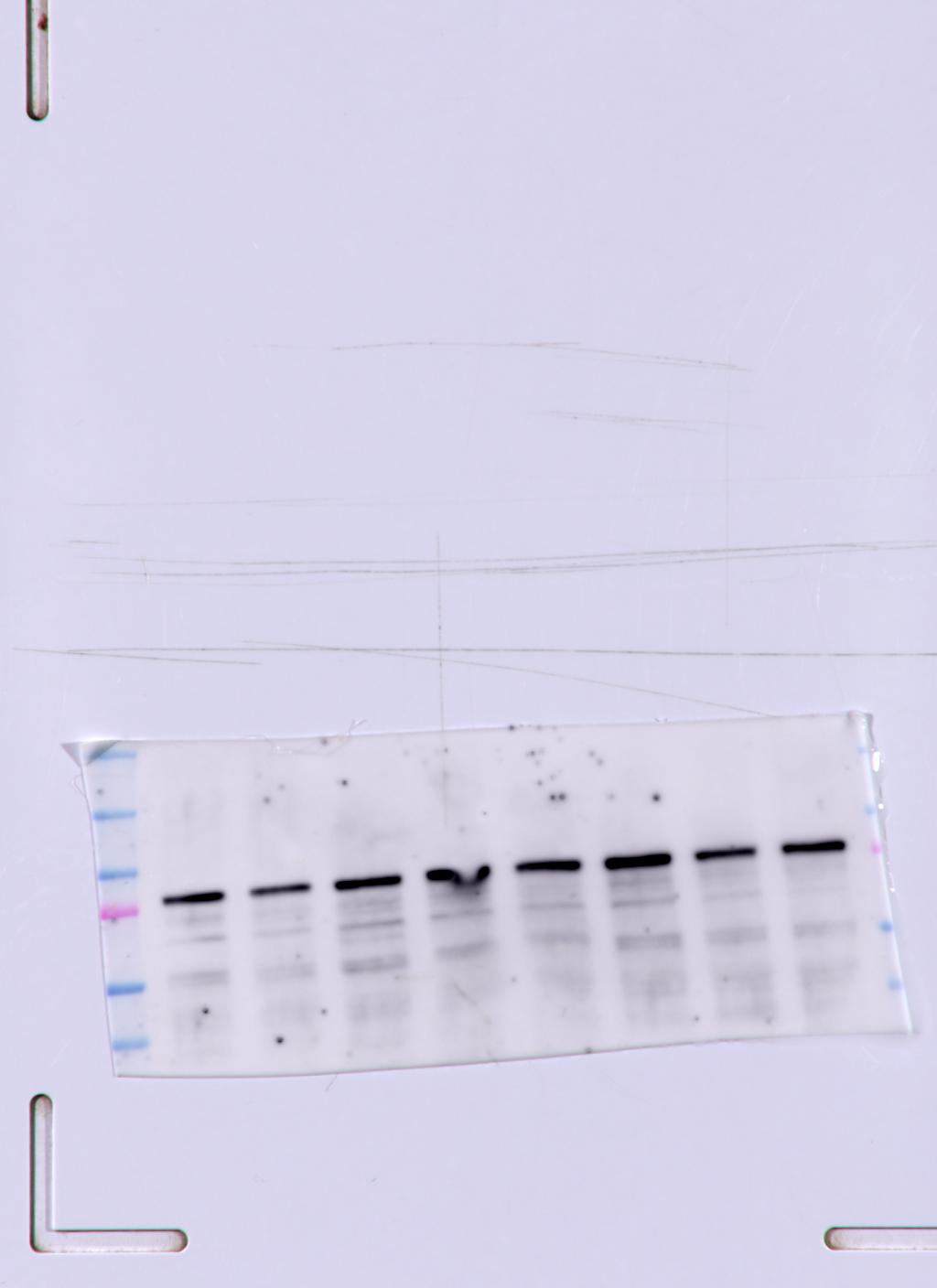

Supplement: Supplementary file 4 — Appendix Figure Source Data [file 44319_2024_180_MOESM4_ESM.zip › Appendix Figure S2/S2C/STAT3 siP4HA1.jpg]

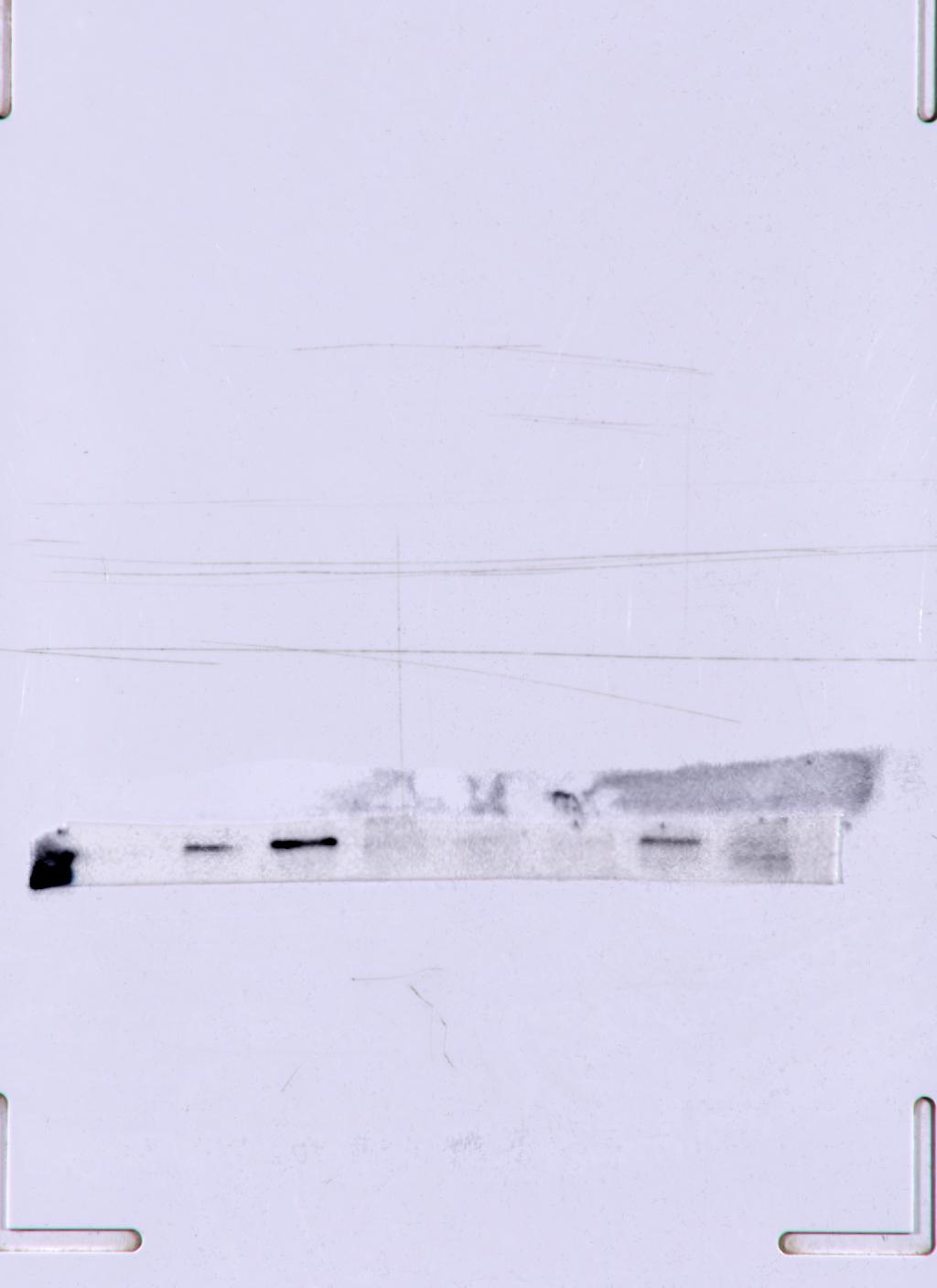

Supplement: Supplementary file 4 — Appendix Figure Source Data [file 44319_2024_180_MOESM4_ESM.zip › Appendix Figure S2/S2C/P4HA1 siP4HA1.jpg]

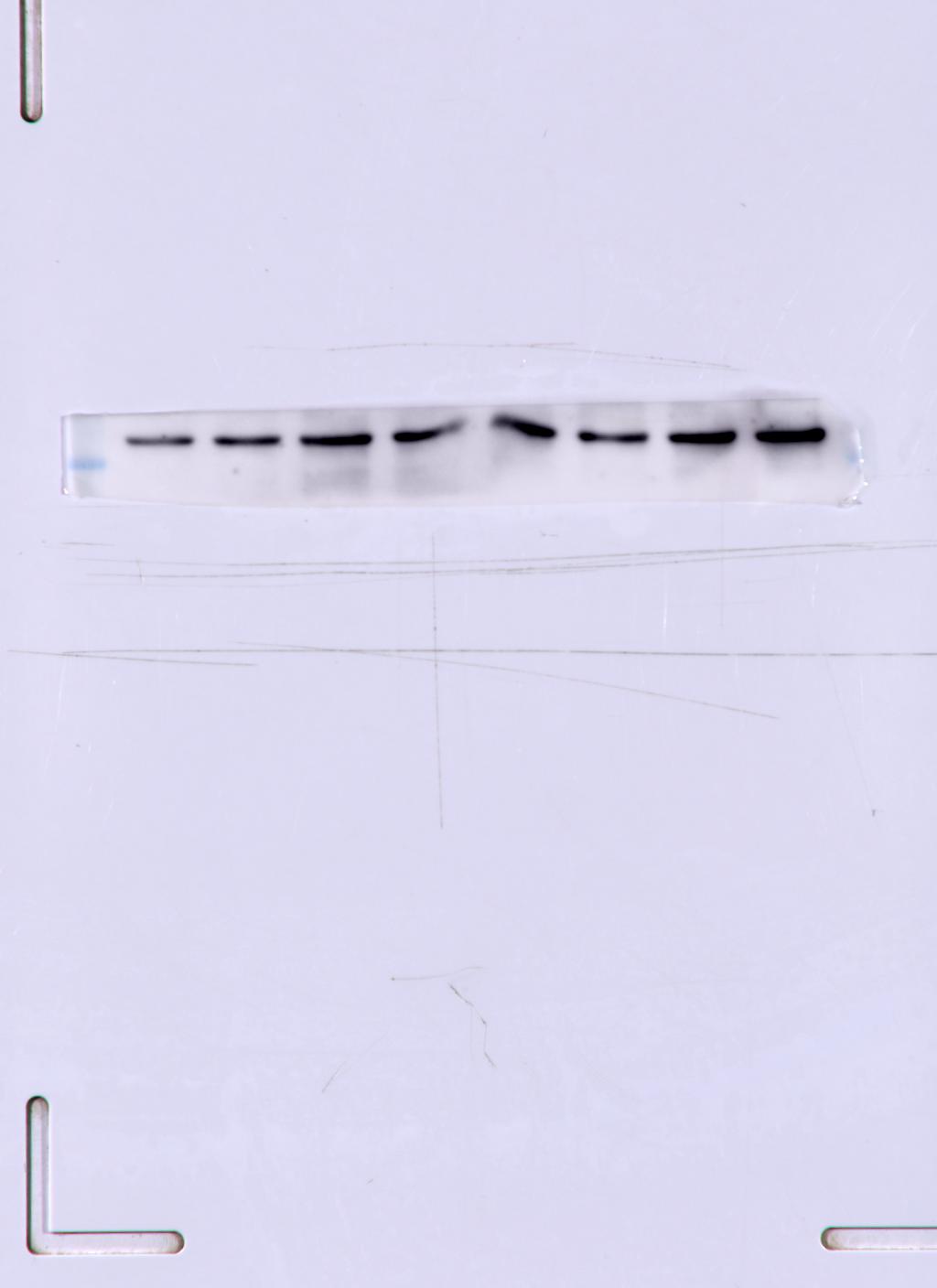

Supplement: Supplementary file 4 — Appendix Figure Source Data [file 44319_2024_180_MOESM4_ESM.zip › Appendix Figure S2/S2C/Actin siP4HA1.jpg]

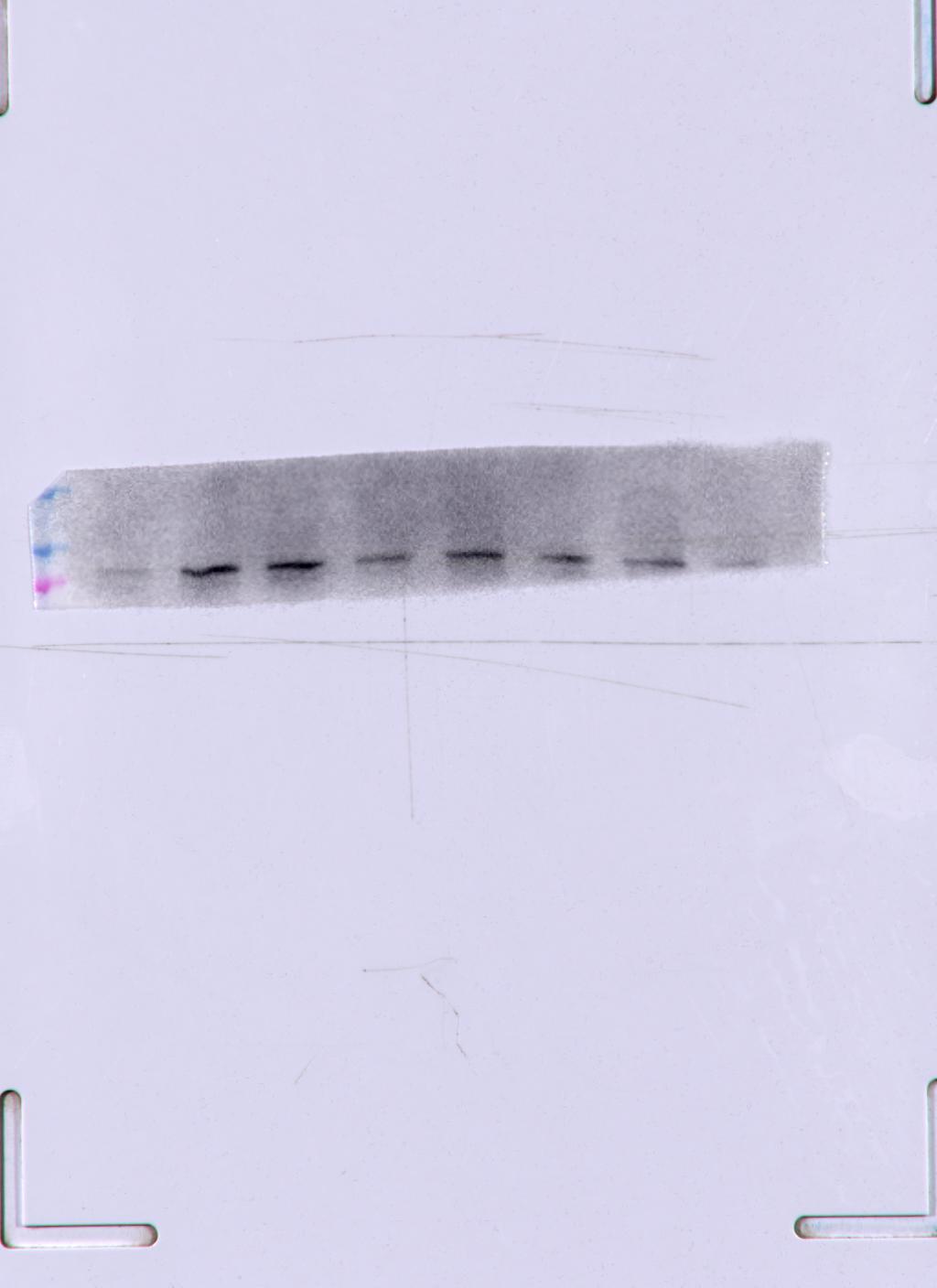

Supplement: Supplementary file 4 — Appendix Figure Source Data [file 44319_2024_180_MOESM4_ESM.zip › Appendix Figure S2/S2C/pSTAT3 siP4HA1.jpg]

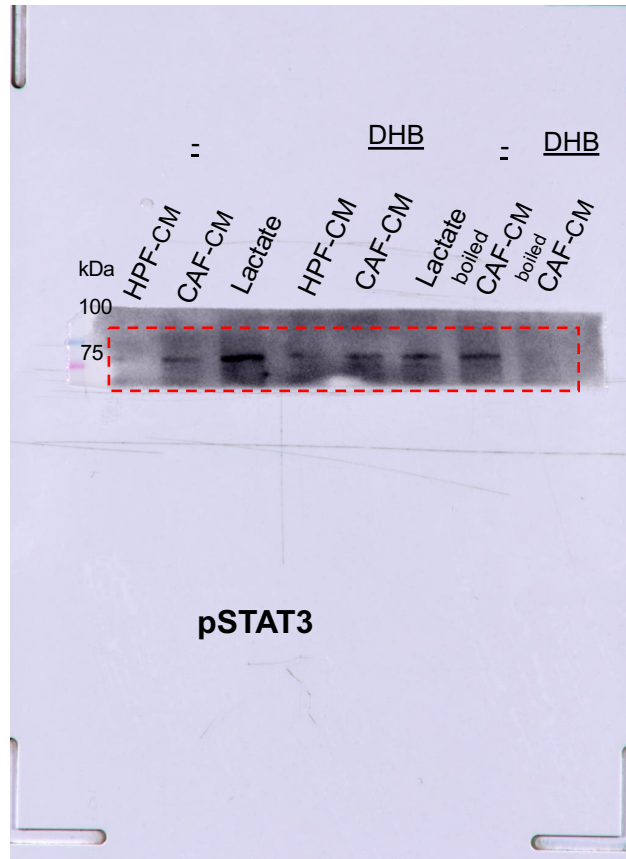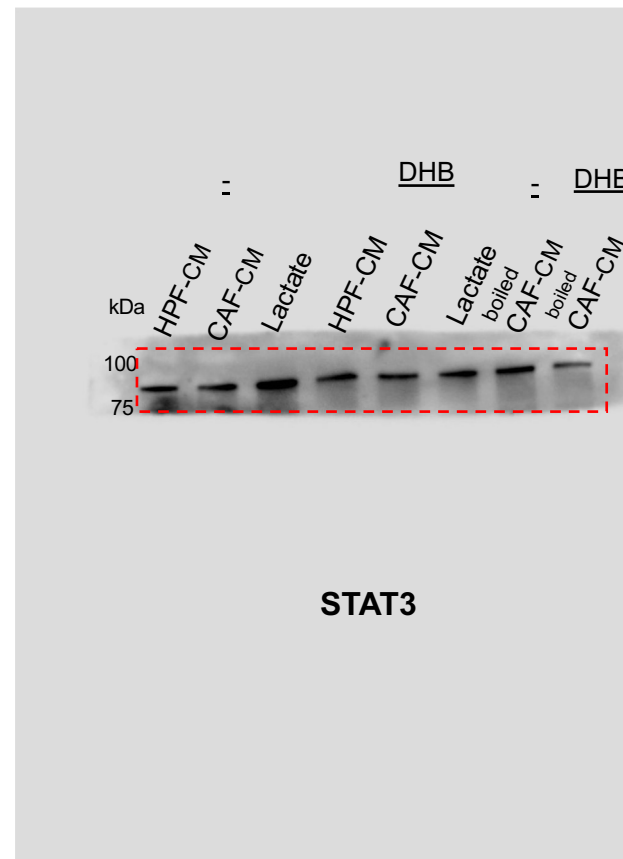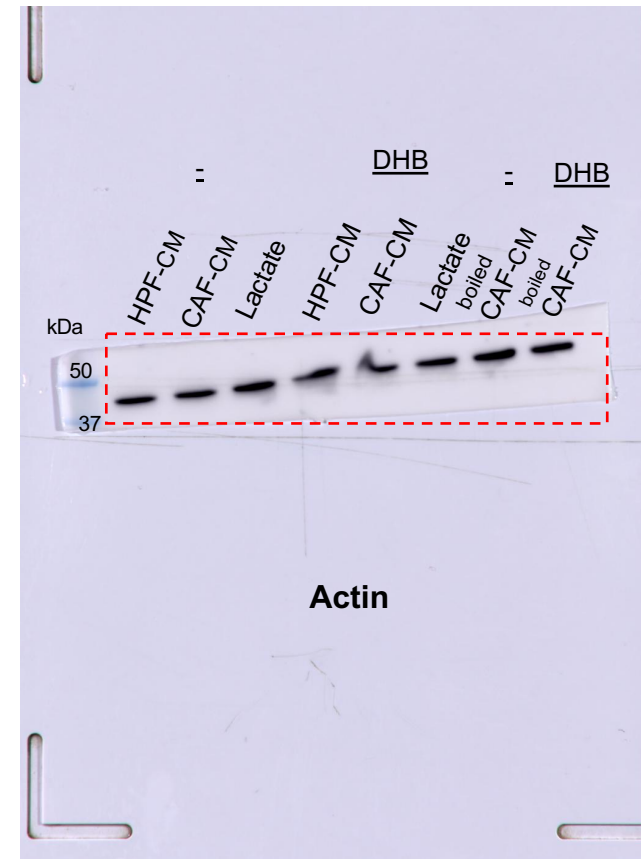

Supplement: Supplementary file 4 — Appendix Figure Source Data [file 44319_2024_180_MOESM4_ESM.zip › Appendix Figure S2/S2D/S2D blot.pdf]

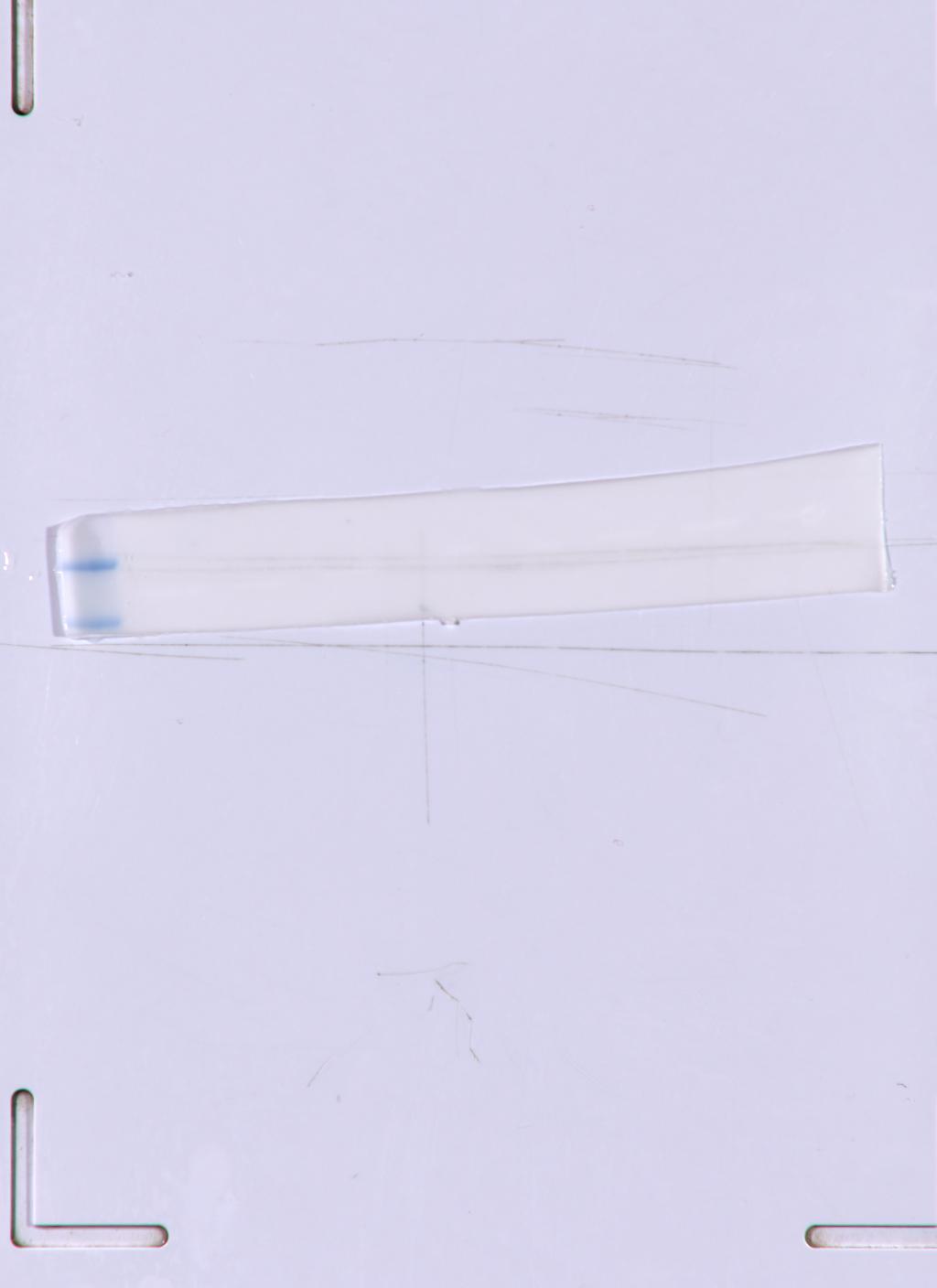

Supplement: Supplementary file 4 — Appendix Figure Source Data [file 44319_2024_180_MOESM4_ESM.zip › Appendix Figure S2/S2D/Actin DHB.jpg]

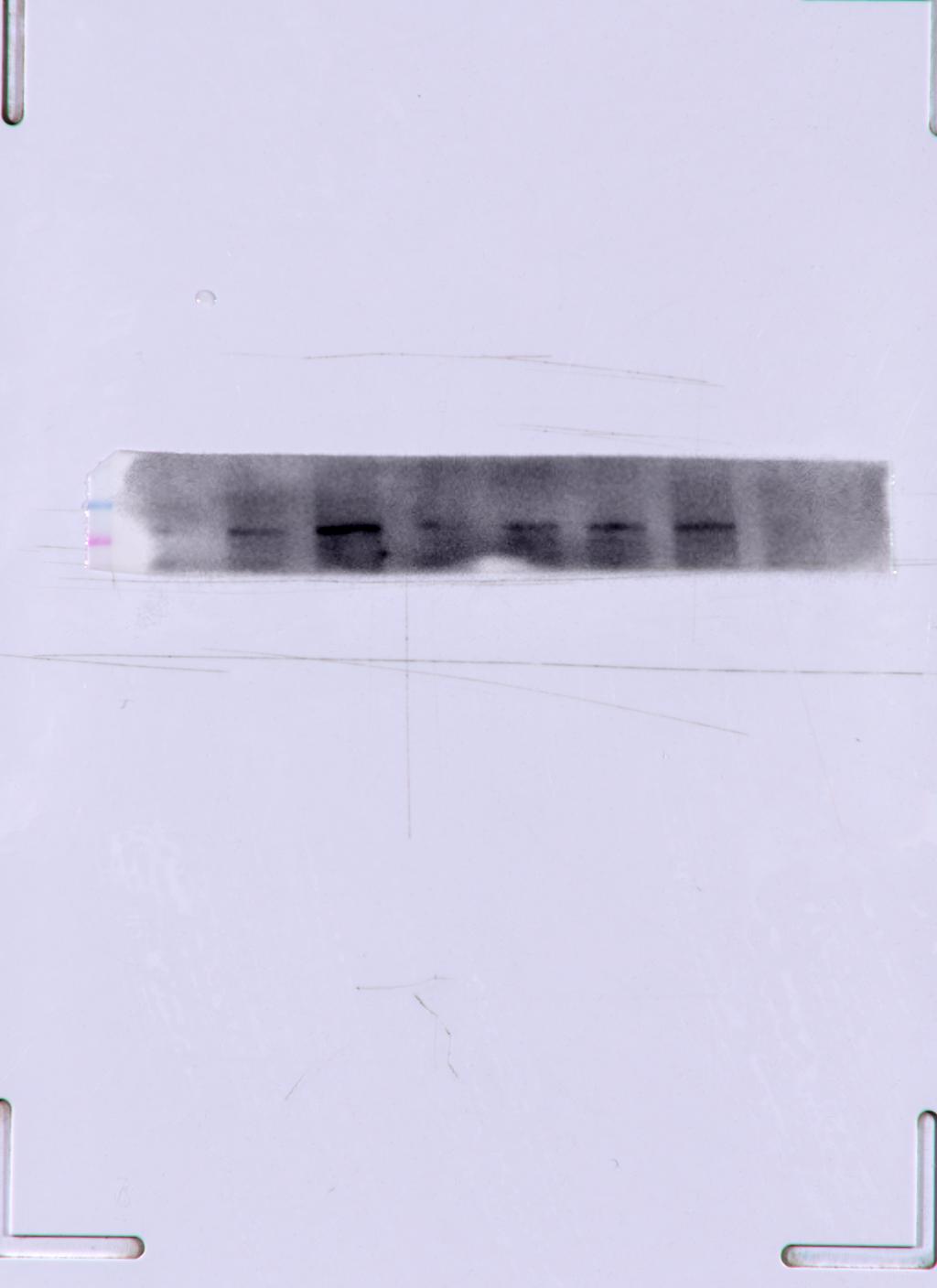

Supplement: Supplementary file 4 — Appendix Figure Source Data [file 44319_2024_180_MOESM4_ESM.zip › Appendix Figure S2/S2D/pSTAT3 DHB.jpg]

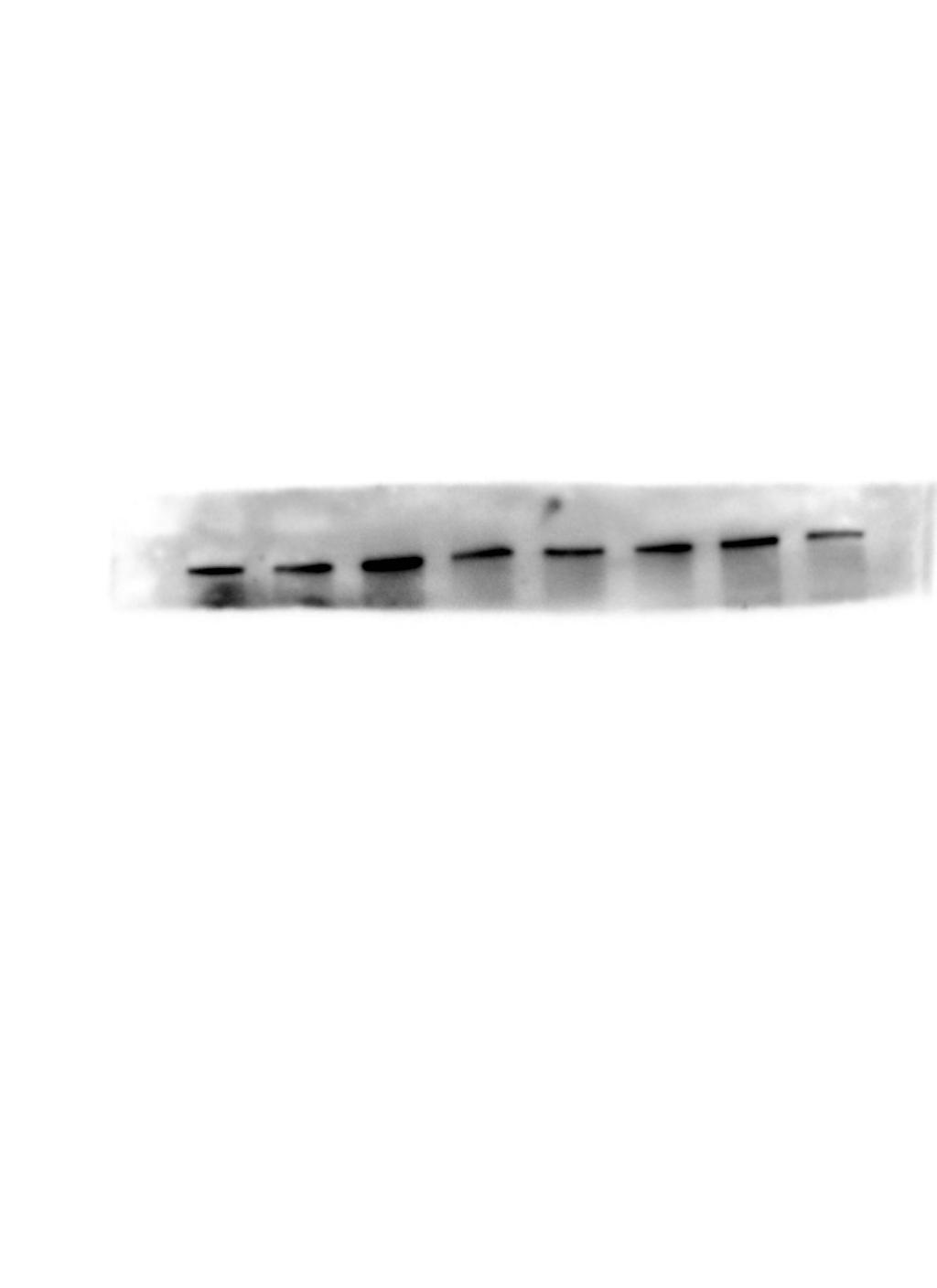

Supplement: Supplementary file 4 — Appendix Figure Source Data [file 44319_2024_180_MOESM4_ESM.zip › Appendix Figure S2/S2D/STAT3 DHB.jpg]

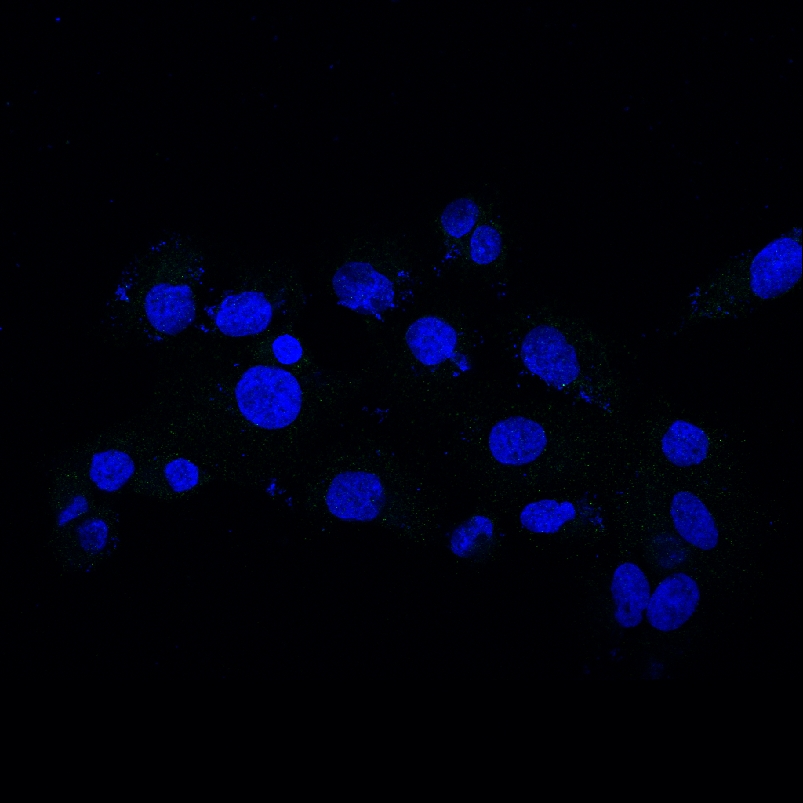

Supplement: Supplementary file 4 — Appendix Figure Source Data [file 44319_2024_180_MOESM4_ESM.zip › Appendix Figure S3/Appendix FIgure S3E/Col1 HPF-CM STAT3i.jpg]

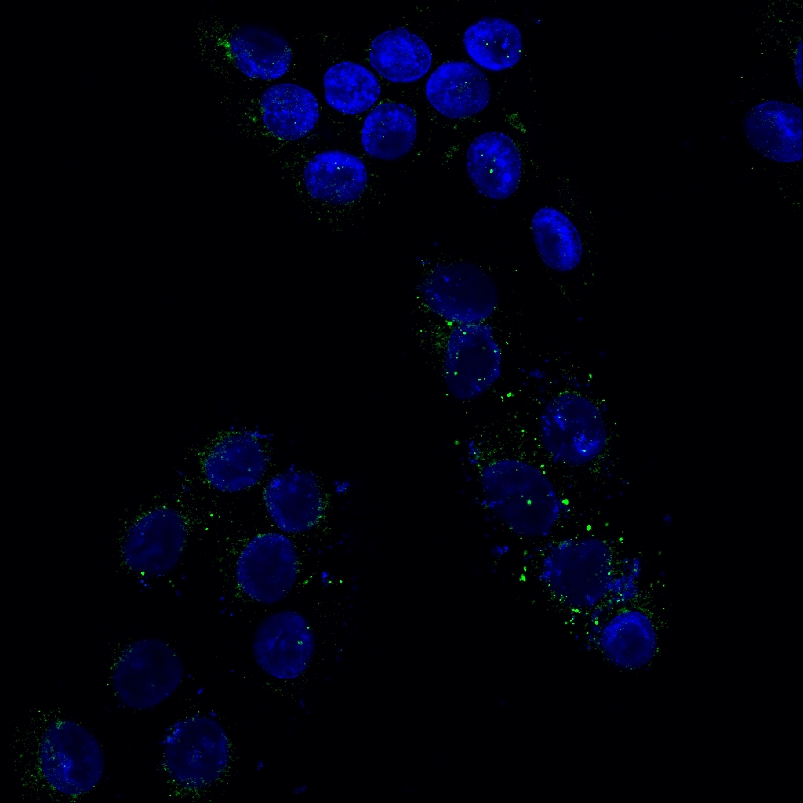

Supplement: Supplementary file 4 — Appendix Figure Source Data [file 44319_2024_180_MOESM4_ESM.zip › Appendix Figure S3/Appendix FIgure S3E/Col1 LA.jpg]

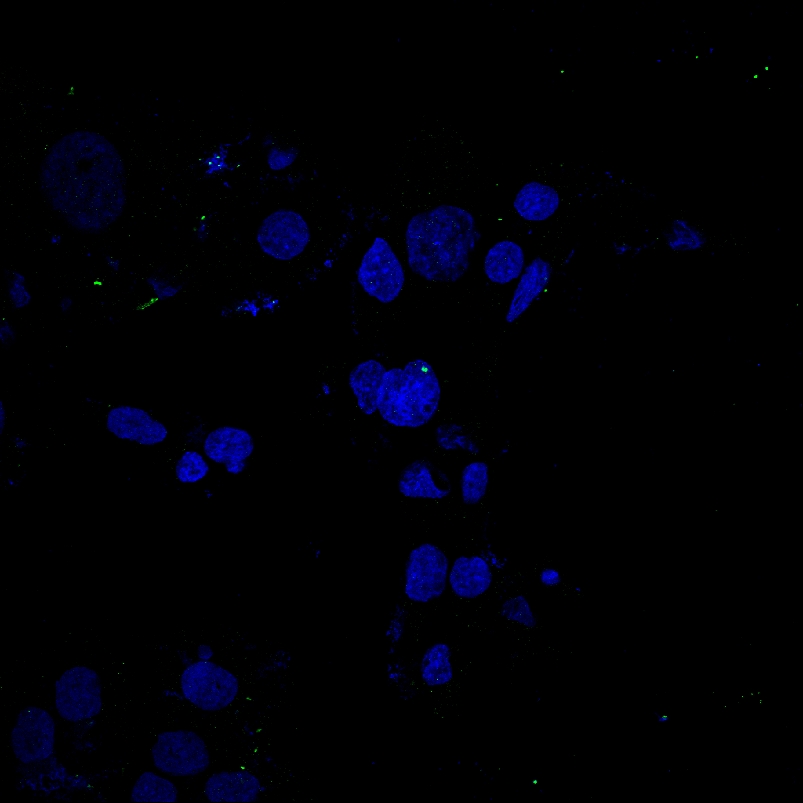

Supplement: Supplementary file 4 — Appendix Figure Source Data [file 44319_2024_180_MOESM4_ESM.zip › Appendix Figure S3/Appendix FIgure S3E/Col1 CAF-CM STAT3i.jpg]

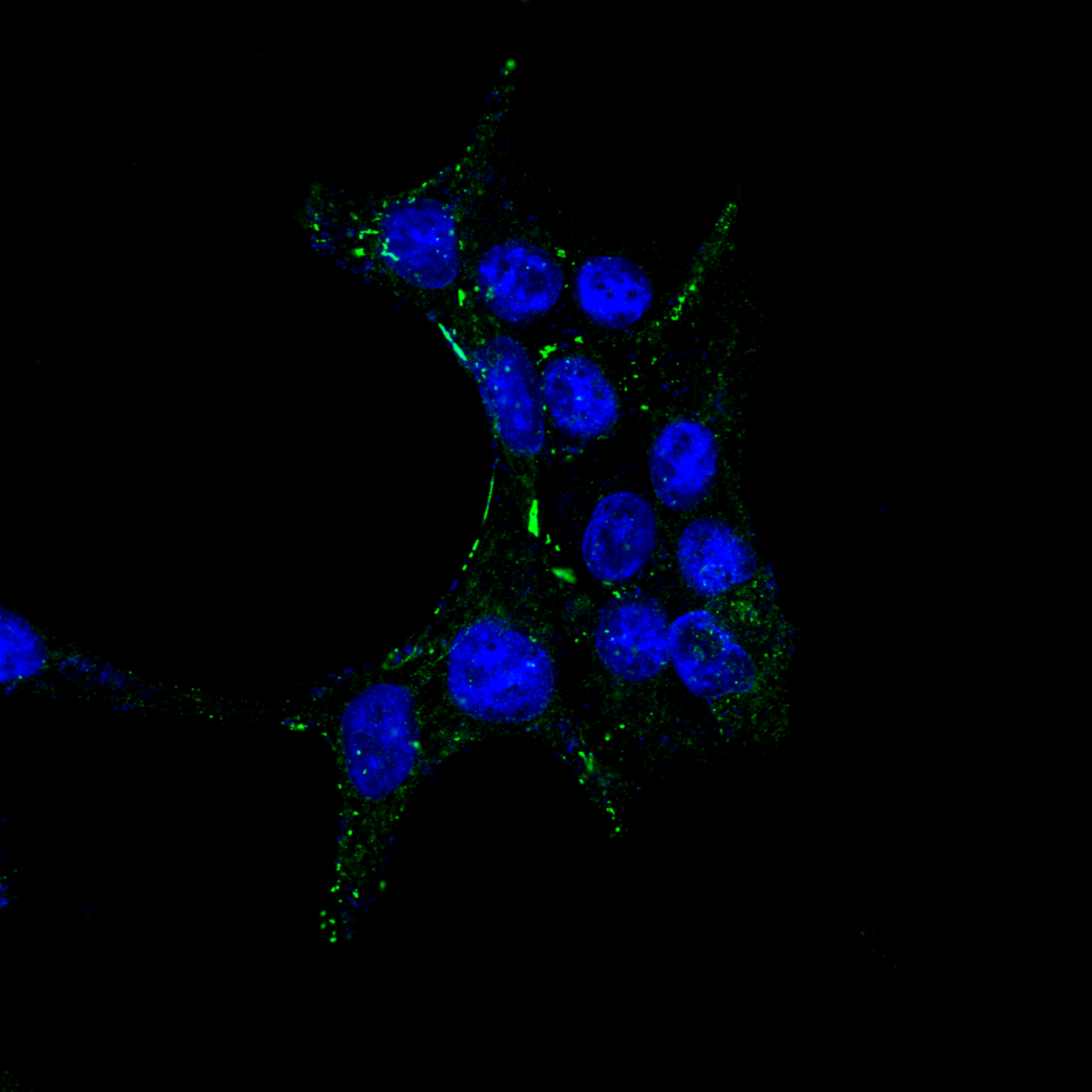

Supplement: Supplementary file 4 — Appendix Figure Source Data [file 44319_2024_180_MOESM4_ESM.zip › Appendix Figure S3/Appendix FIgure S3E/Col1 CAF-CM.tif]

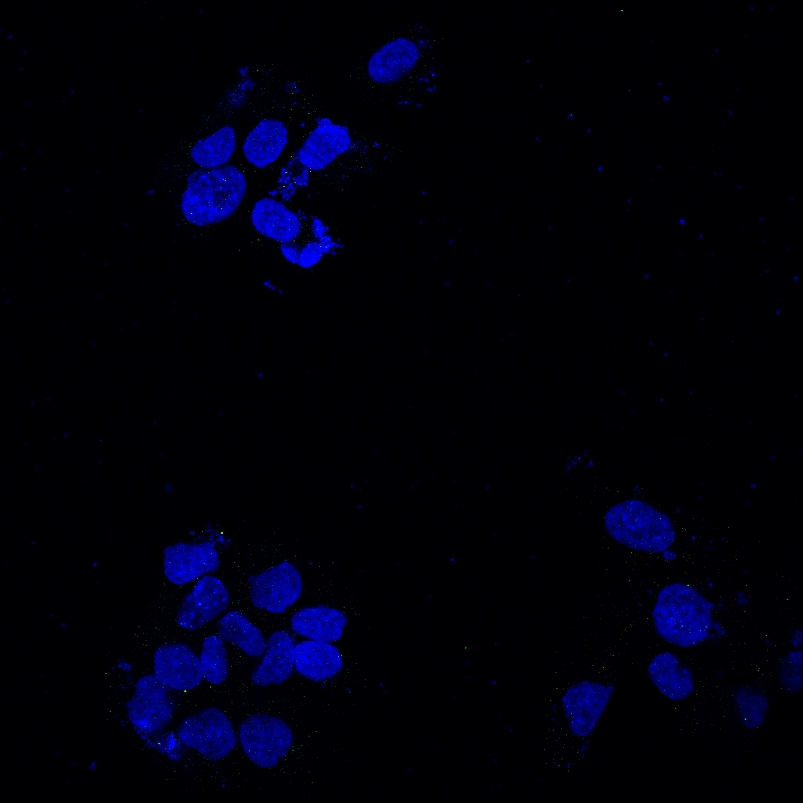

Supplement: Supplementary file 4 — Appendix Figure Source Data [file 44319_2024_180_MOESM4_ESM.zip › Appendix Figure S3/Appendix FIgure S3E/Col1 HPF-CM.jpg]

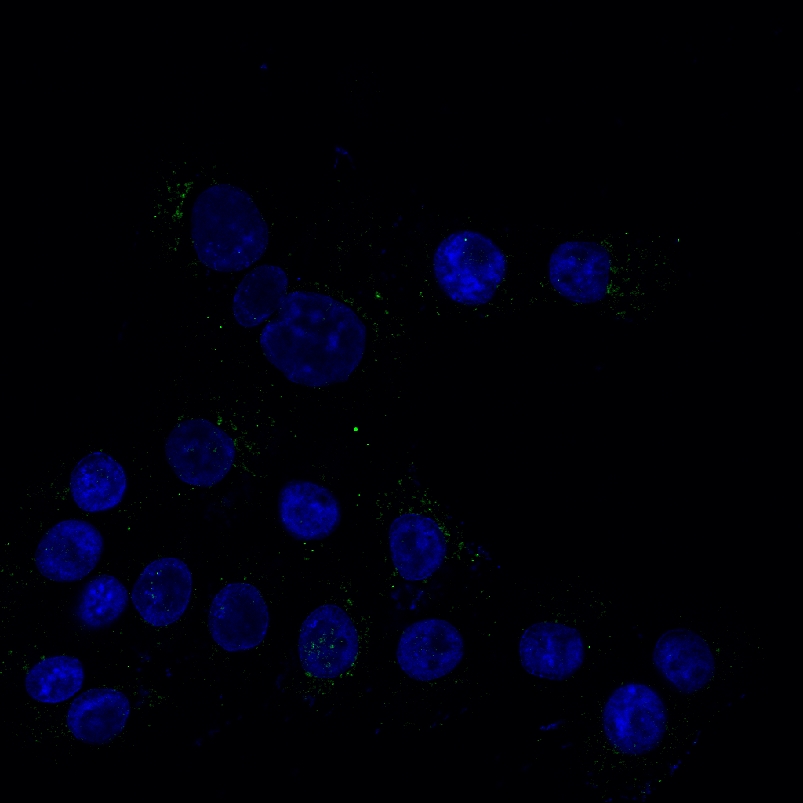

Supplement: Supplementary file 4 — Appendix Figure Source Data [file 44319_2024_180_MOESM4_ESM.zip › Appendix Figure S3/Appendix FIgure S3E/Col1 LA STAT3i.jpg]

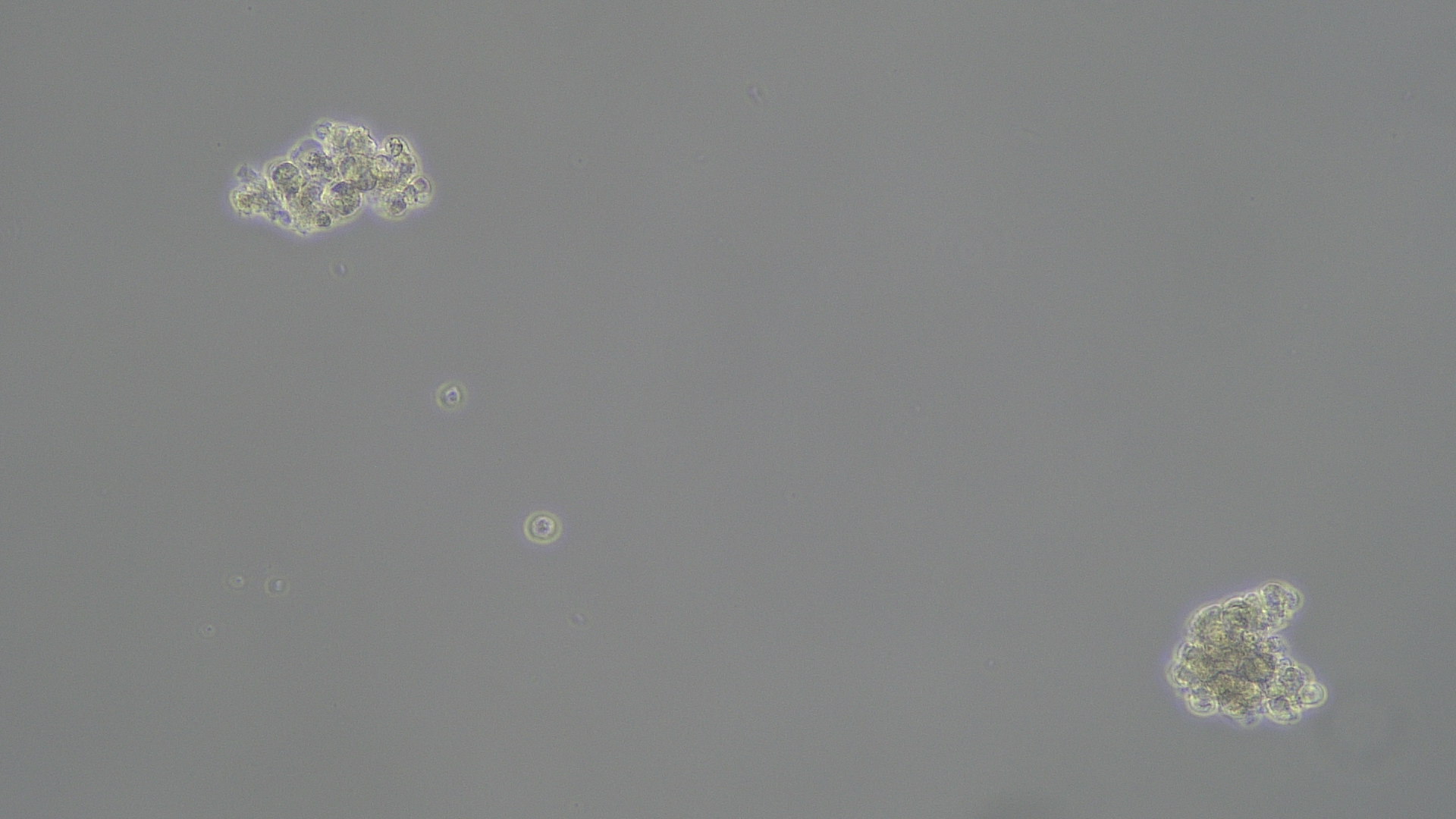

Supplement: Supplementary file 4 — Appendix Figure Source Data [file 44319_2024_180_MOESM4_ESM.zip › Appendix Figure S3/Appendix Figure S3C/LA STAT3i (22Rv1).JPG]

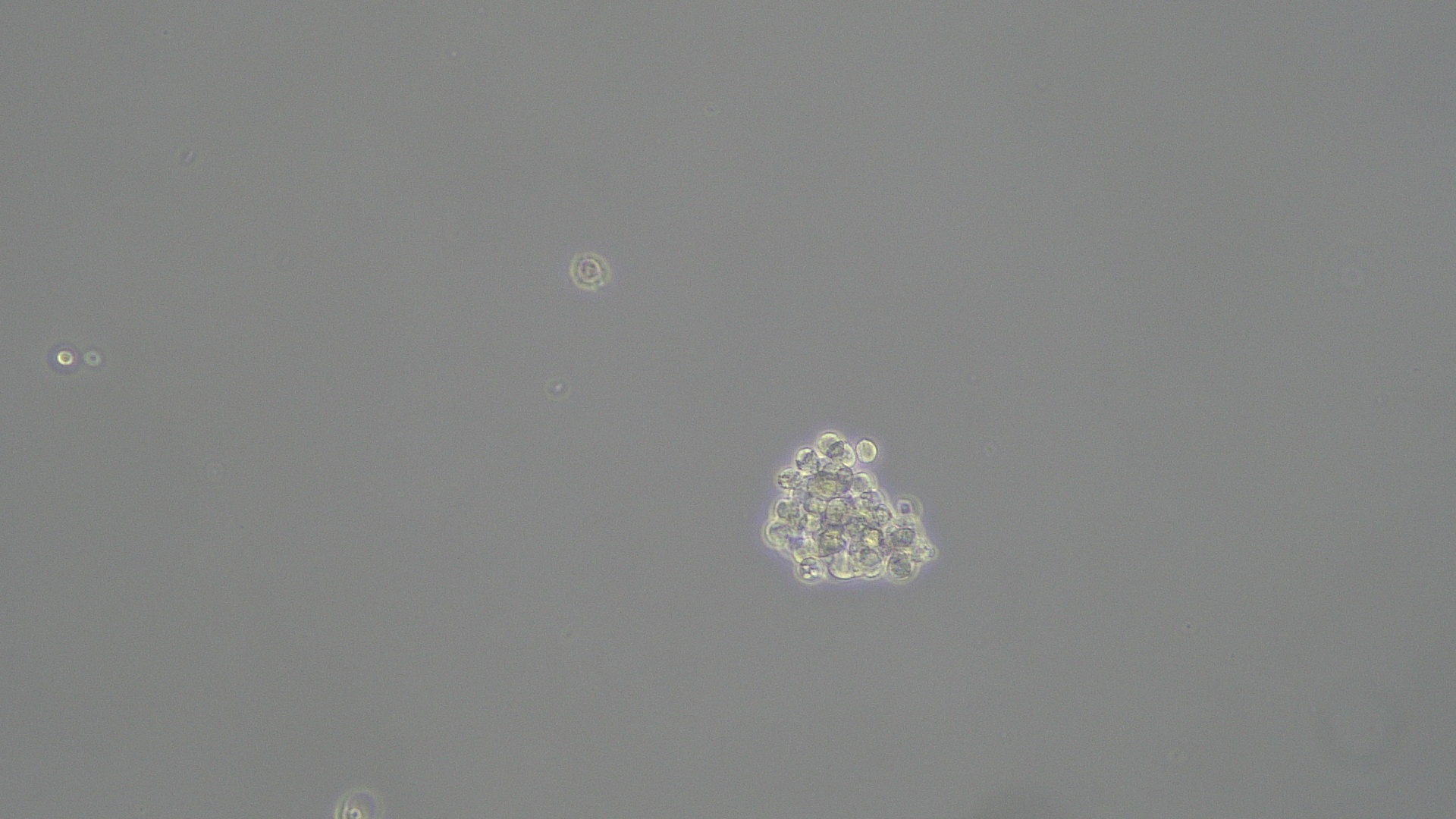

Supplement: Supplementary file 4 — Appendix Figure Source Data [file 44319_2024_180_MOESM4_ESM.zip › Appendix Figure S3/Appendix Figure S3C/NT STAT3i (22Rv1).JPG]

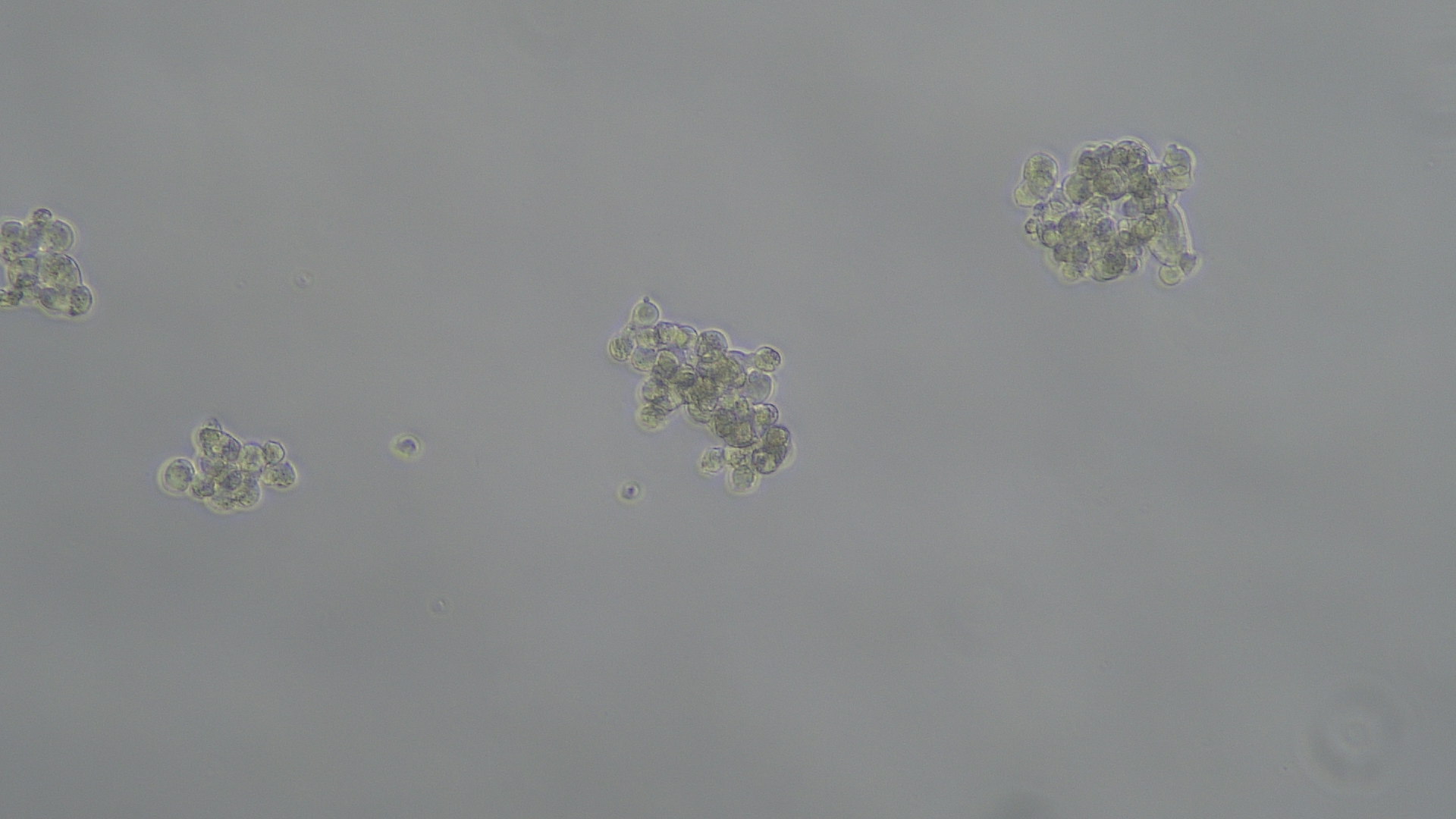

Supplement: Supplementary file 4 — Appendix Figure Source Data [file 44319_2024_180_MOESM4_ESM.zip › Appendix Figure S3/Appendix Figure S3C/NT (22Rv1).JPG]

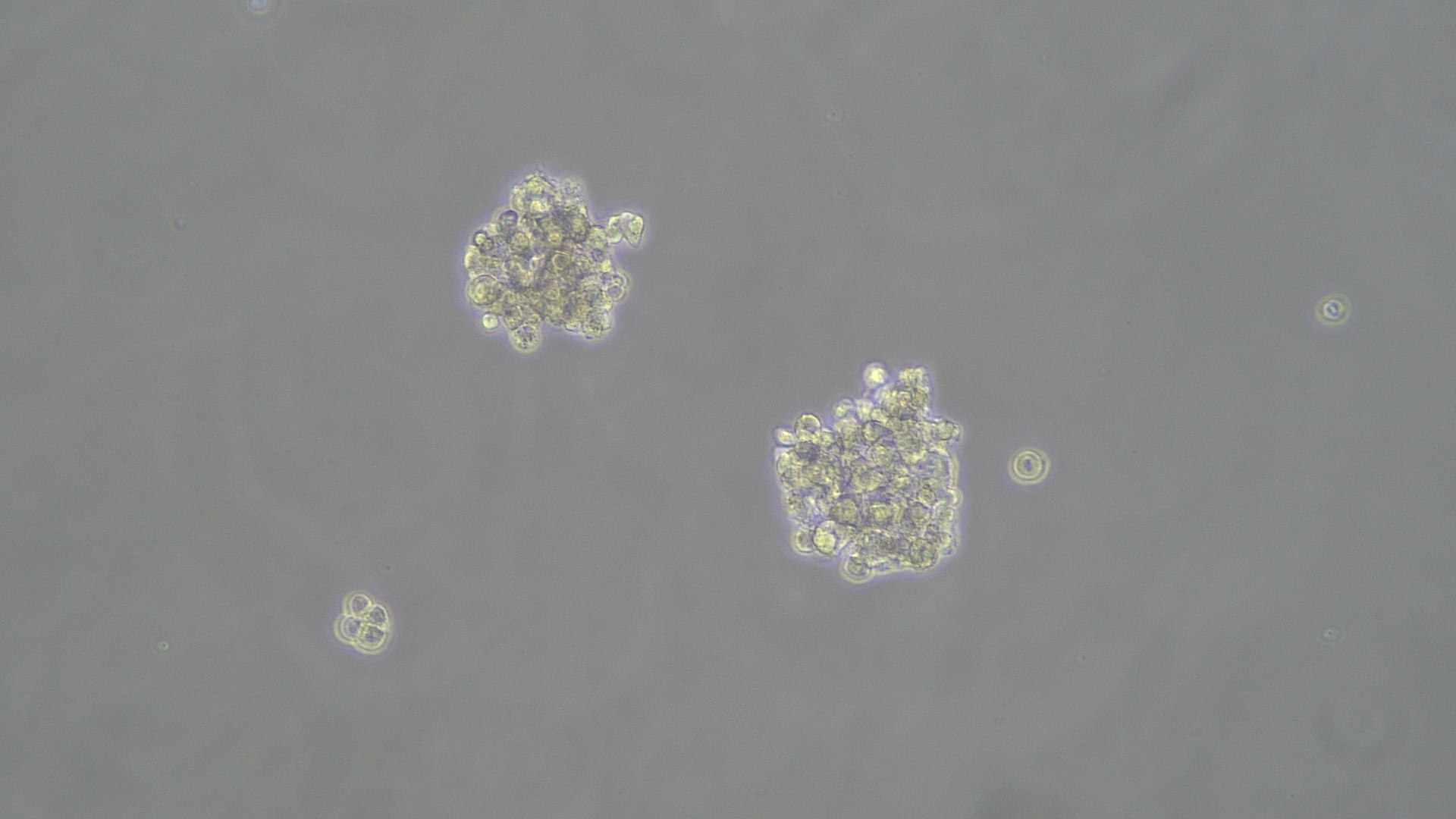

Supplement: Supplementary file 4 — Appendix Figure Source Data [file 44319_2024_180_MOESM4_ESM.zip › Appendix Figure S3/Appendix Figure S3C/LA (22Rv1.JPG]

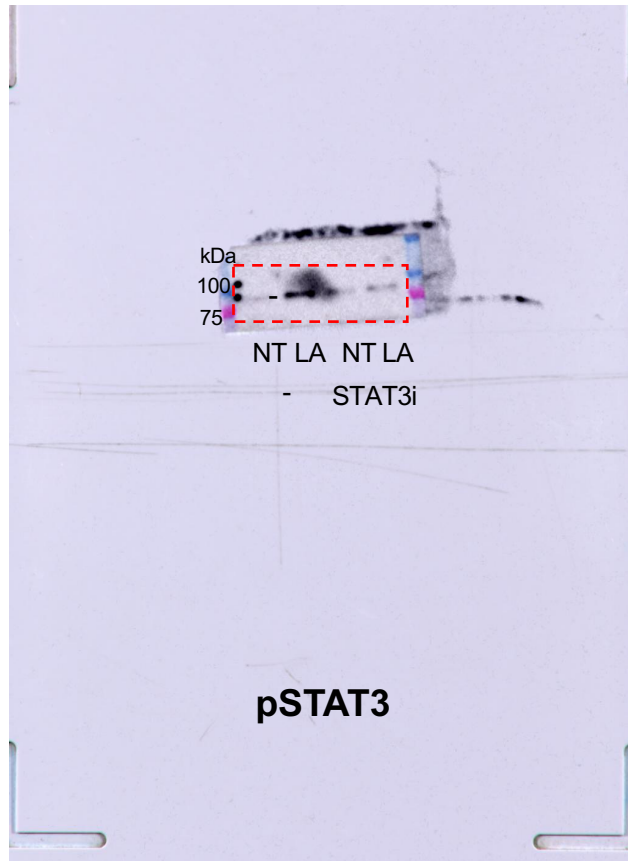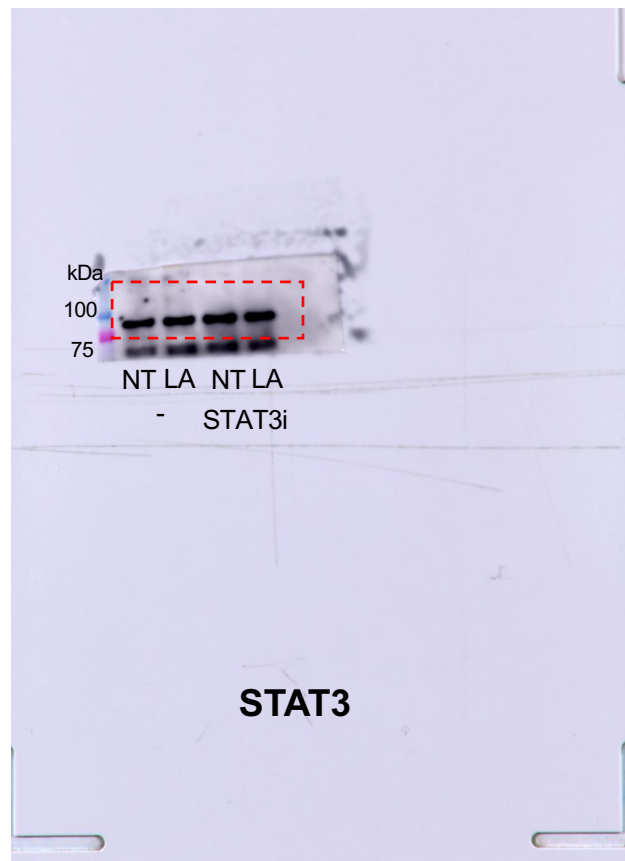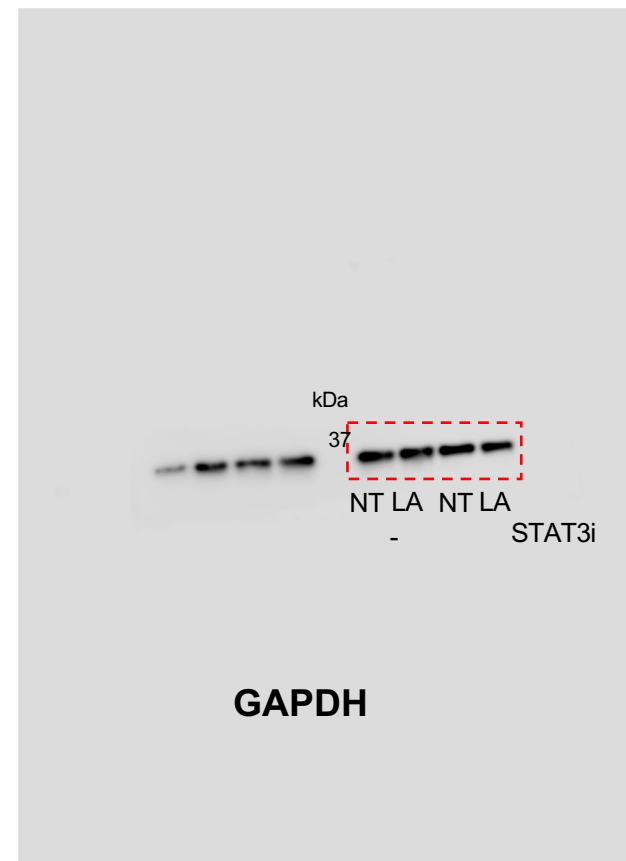

Supplement: Supplementary file 4 — Appendix Figure Source Data [file 44319_2024_180_MOESM4_ESM.zip › Appendix Figure S3/Appendix Figure S3A/Appendix Figure S3A blot.pdf]

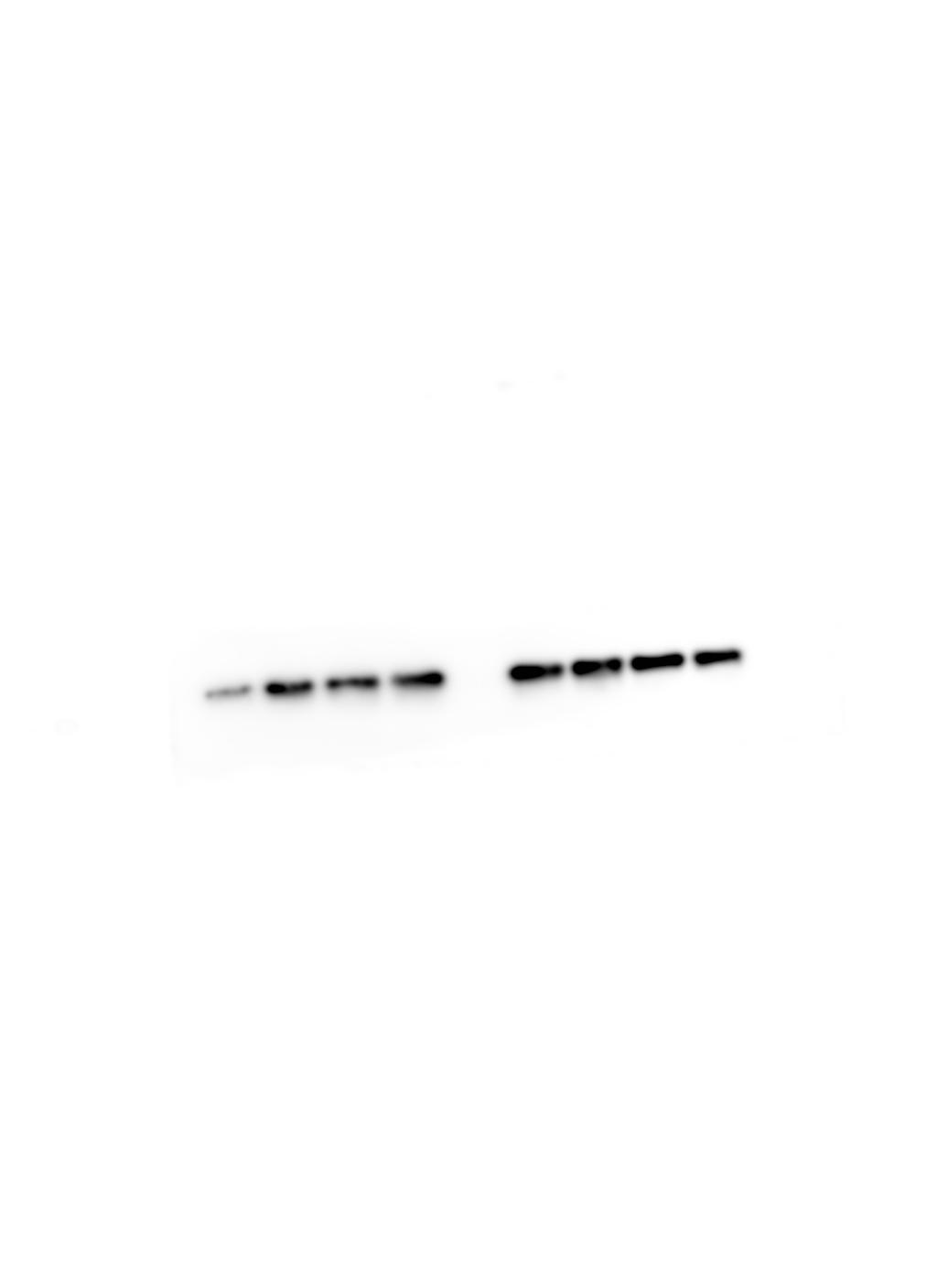

Supplement: Supplementary file 4 — Appendix Figure Source Data [file 44319_2024_180_MOESM4_ESM.zip › Appendix Figure S3/Appendix Figure S3A/WB Actin STAT3i_22rv1.jpg]

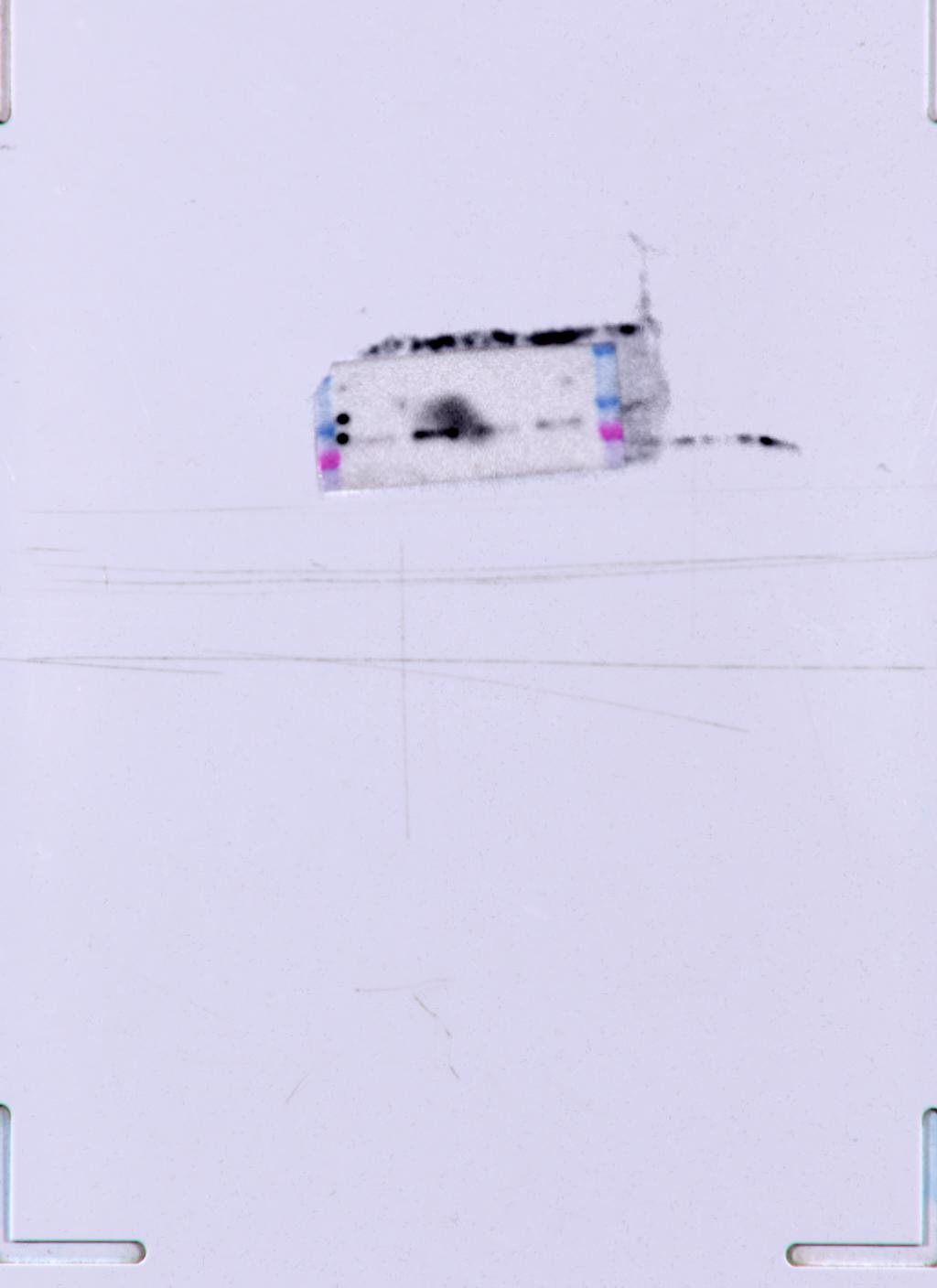

Supplement: Supplementary file 4 — Appendix Figure Source Data [file 44319_2024_180_MOESM4_ESM.zip › Appendix Figure S3/Appendix Figure S3A/WB pSTAT3 STAT3i_22Rv1.jpg]

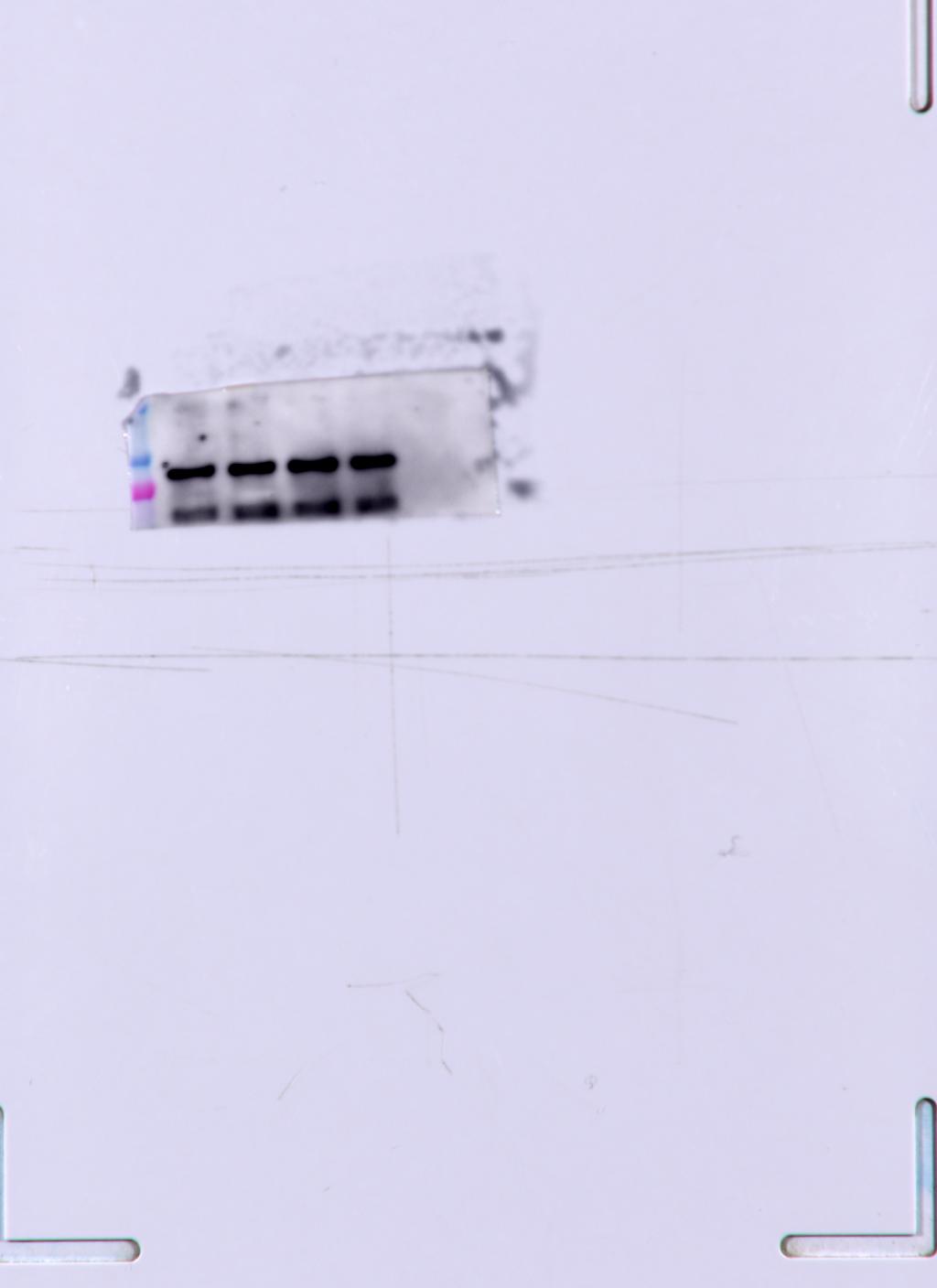

Supplement: Supplementary file 4 — Appendix Figure Source Data [file 44319_2024_180_MOESM4_ESM.zip › Appendix Figure S3/Appendix Figure S3A/WB STAT3 STAT3i_22Rv1.jpg]

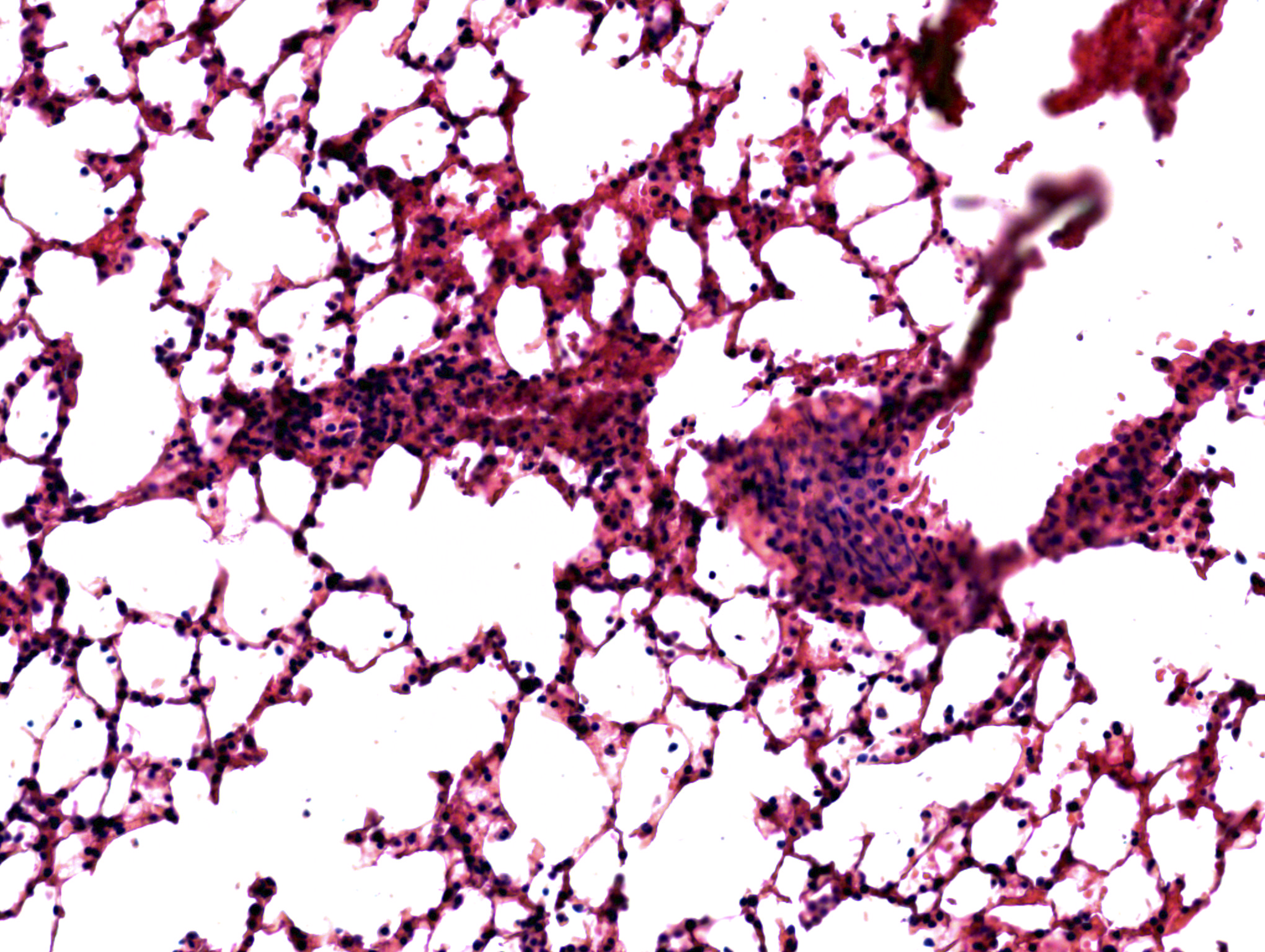

Supplement: Supplementary file 4 — Appendix Figure Source Data [file 44319_2024_180_MOESM4_ESM.zip › Appendix Figure S4/S4C/CAF Scr.tif]

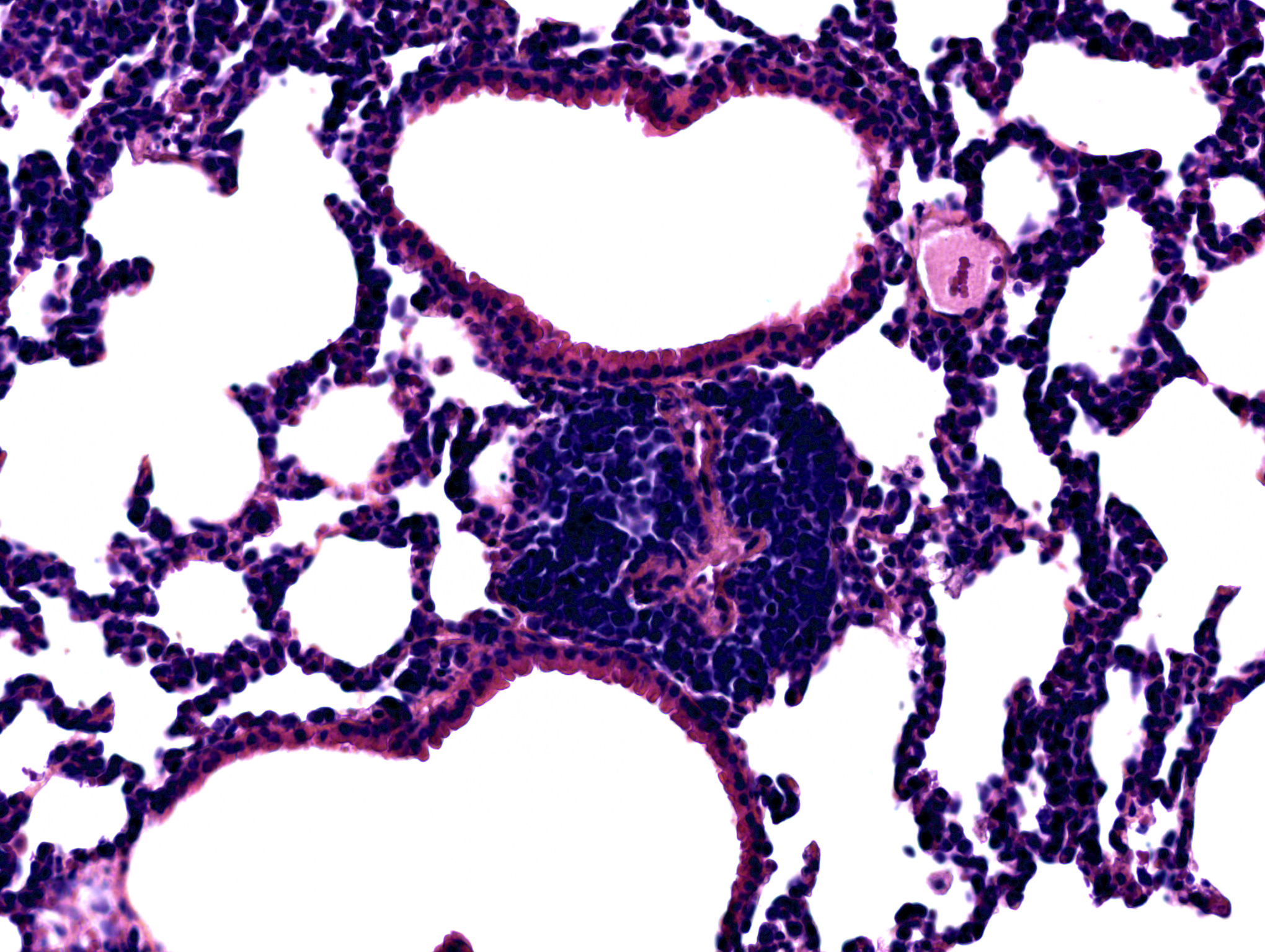

Supplement: Supplementary file 4 — Appendix Figure Source Data [file 44319_2024_180_MOESM4_ESM.zip › Appendix Figure S4/S4C/Lactate Scr.tif]

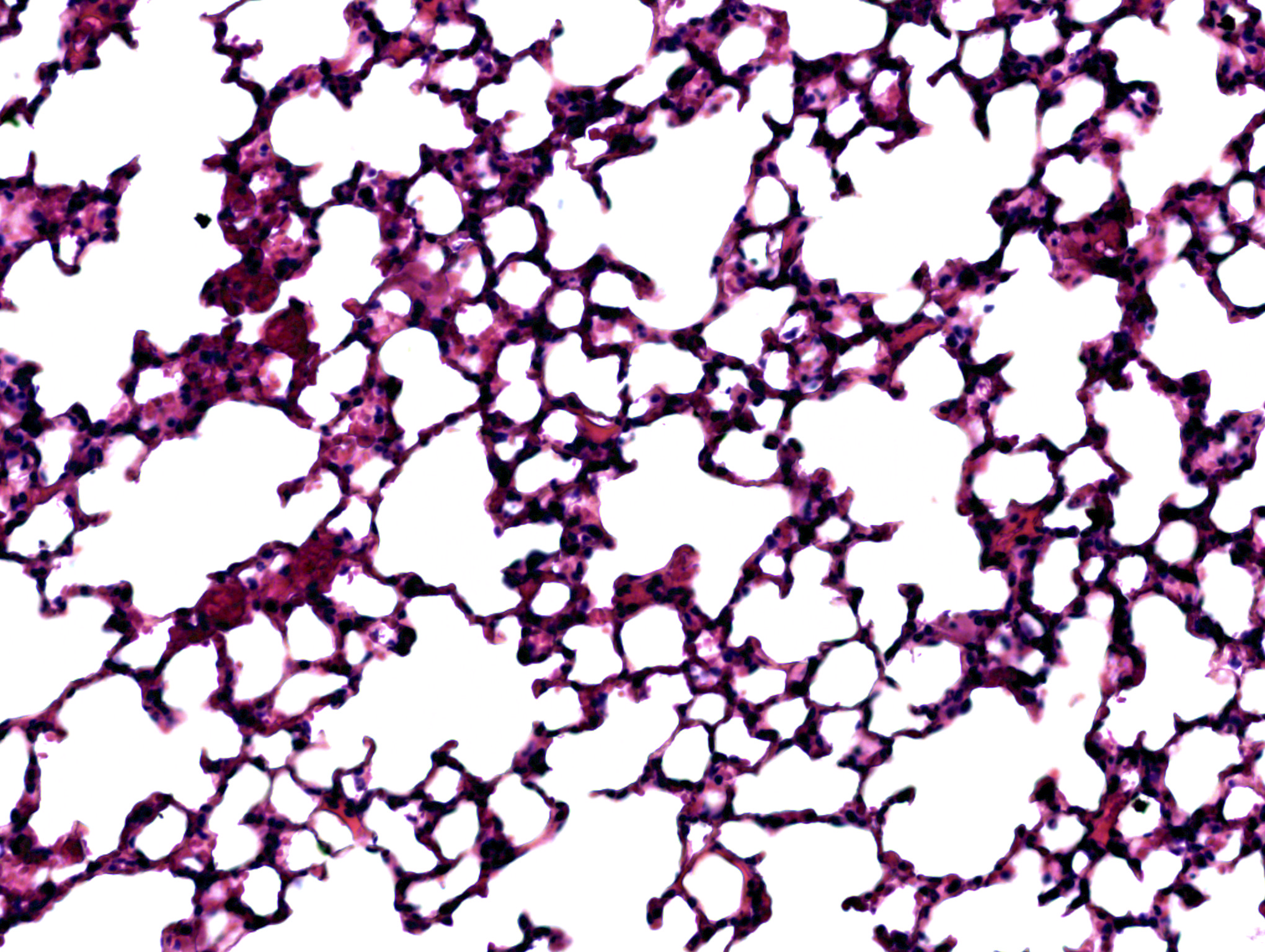

Supplement: Supplementary file 4 — Appendix Figure Source Data [file 44319_2024_180_MOESM4_ESM.zip › Appendix Figure S4/S4C/HPF shDDR1.tif]

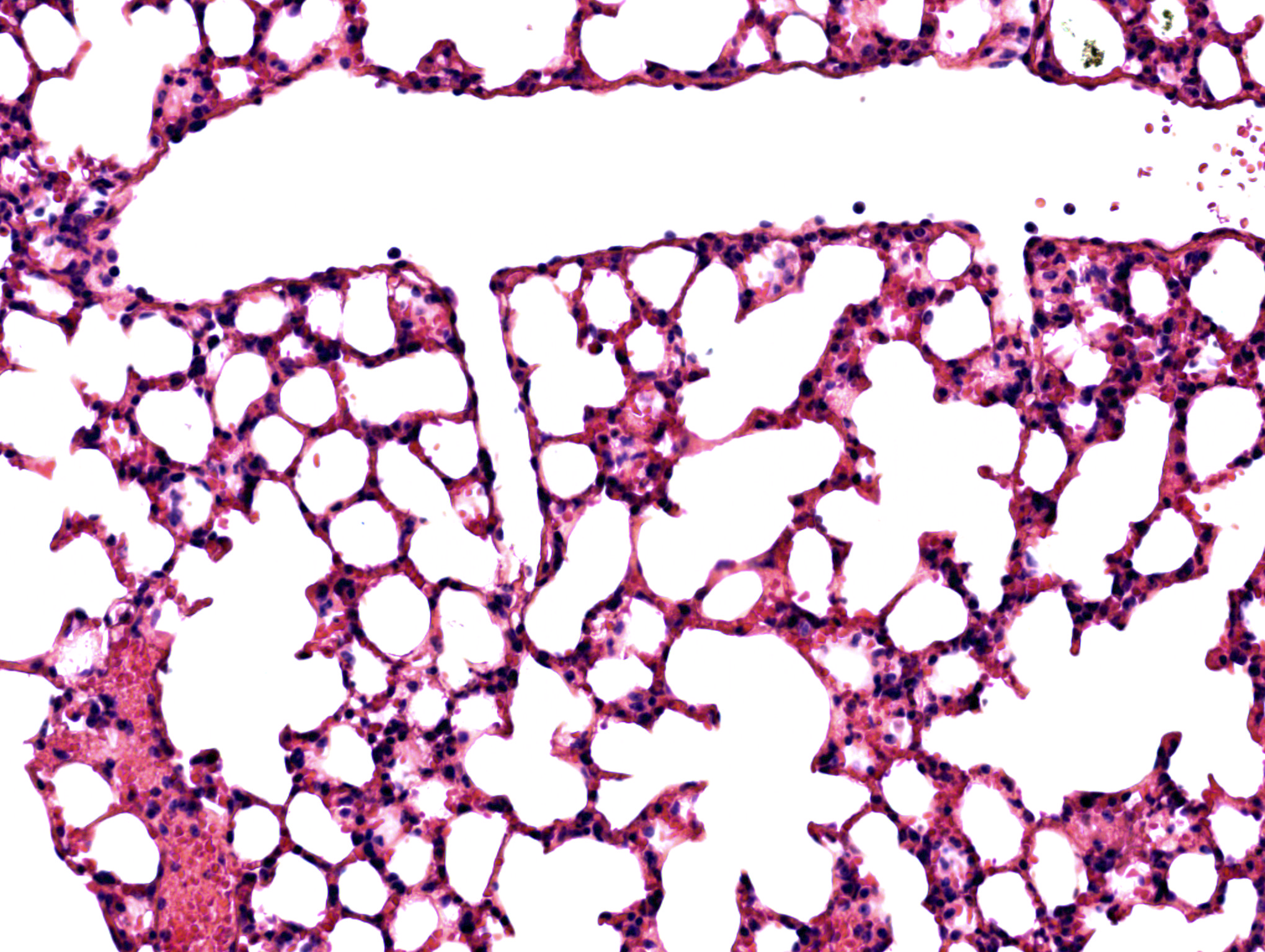

Supplement: Supplementary file 4 — Appendix Figure Source Data [file 44319_2024_180_MOESM4_ESM.zip › Appendix Figure S4/S4C/HPF Scr .tif]

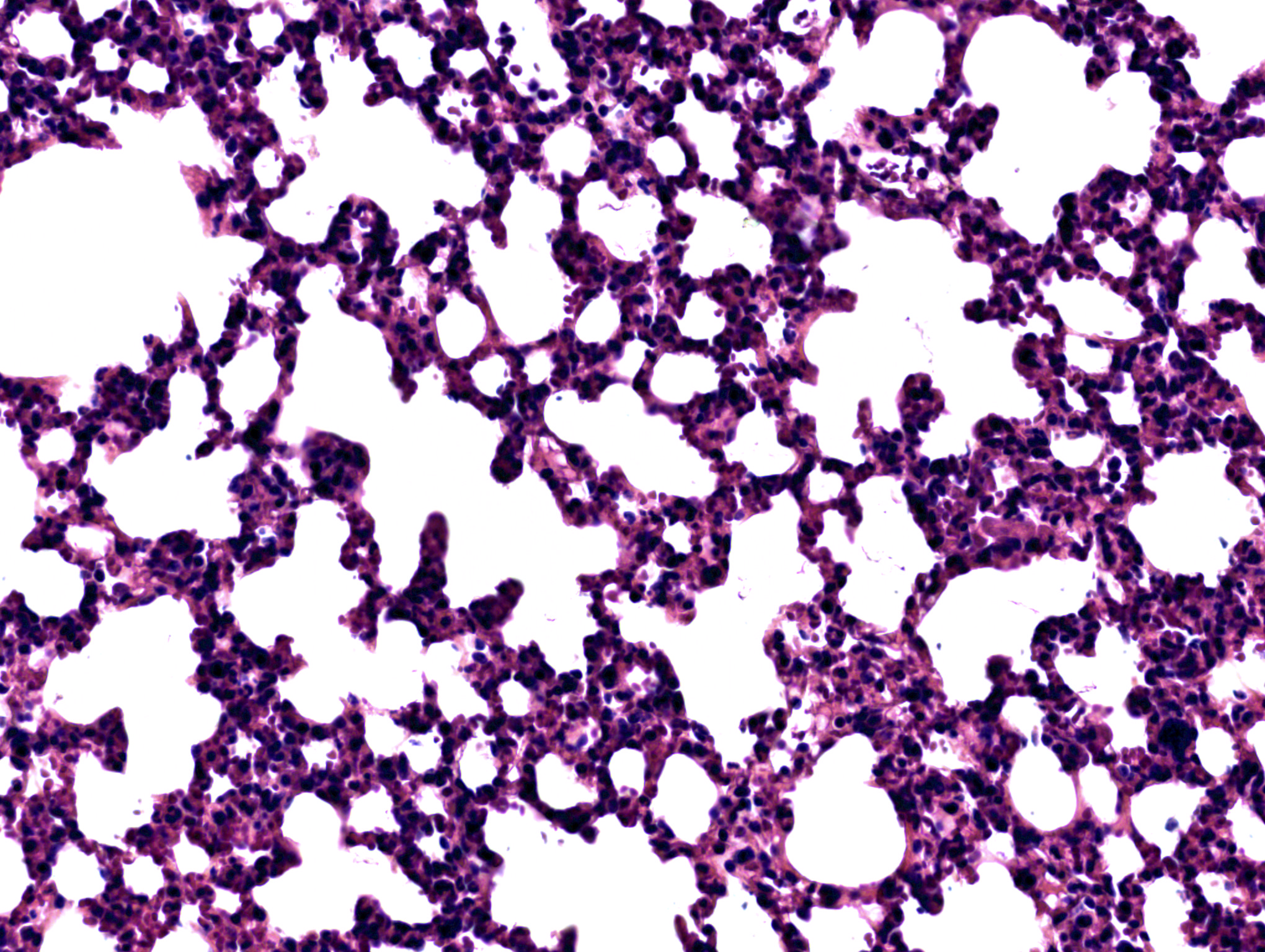

Supplement: Supplementary file 4 — Appendix Figure Source Data [file 44319_2024_180_MOESM4_ESM.zip › Appendix Figure S4/S4C/CAF shDDR1.tif]

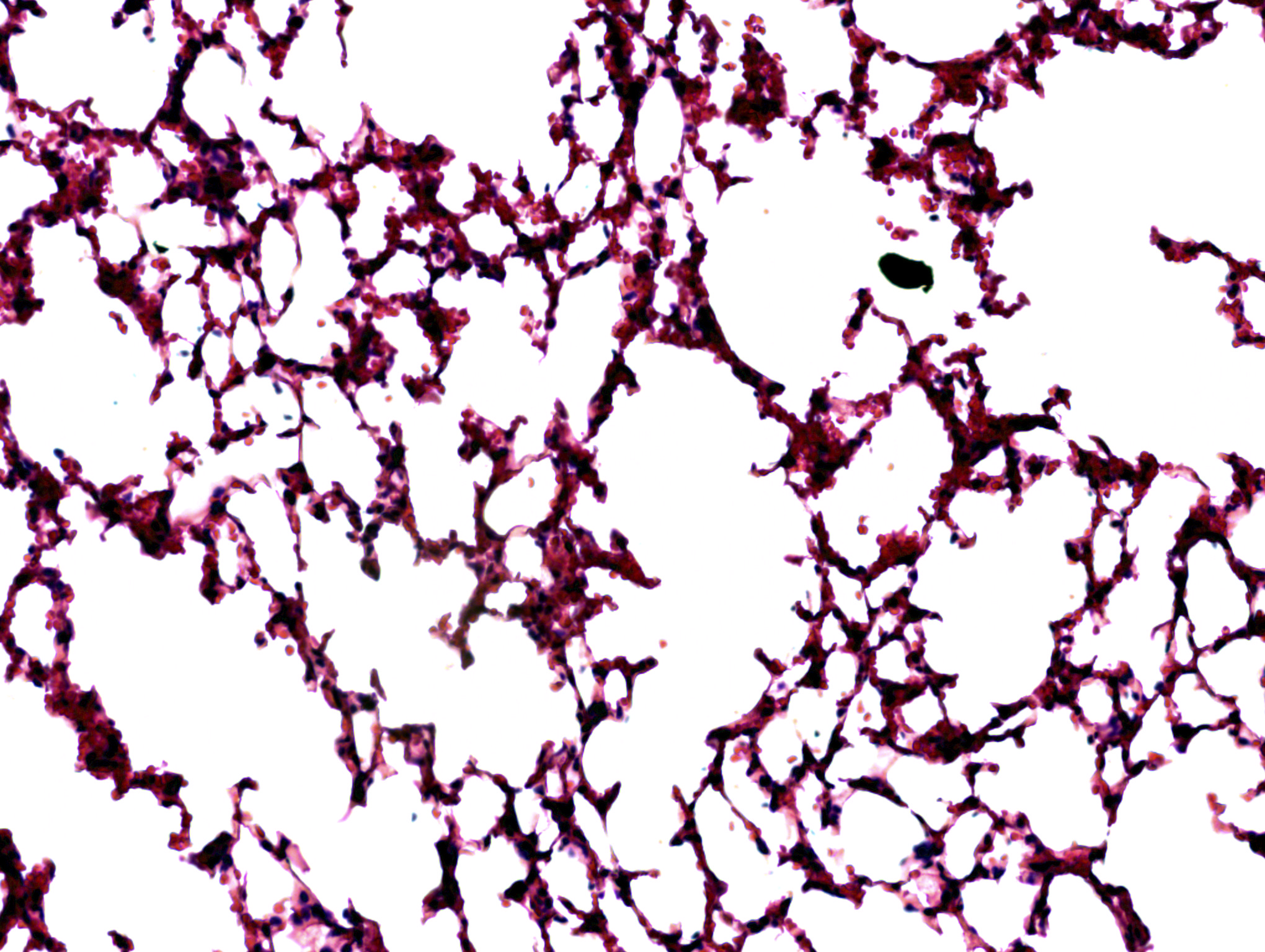

Supplement: Supplementary file 4 — Appendix Figure Source Data [file 44319_2024_180_MOESM4_ESM.zip › Appendix Figure S4/S4C/Lactate shDDR1.tif]

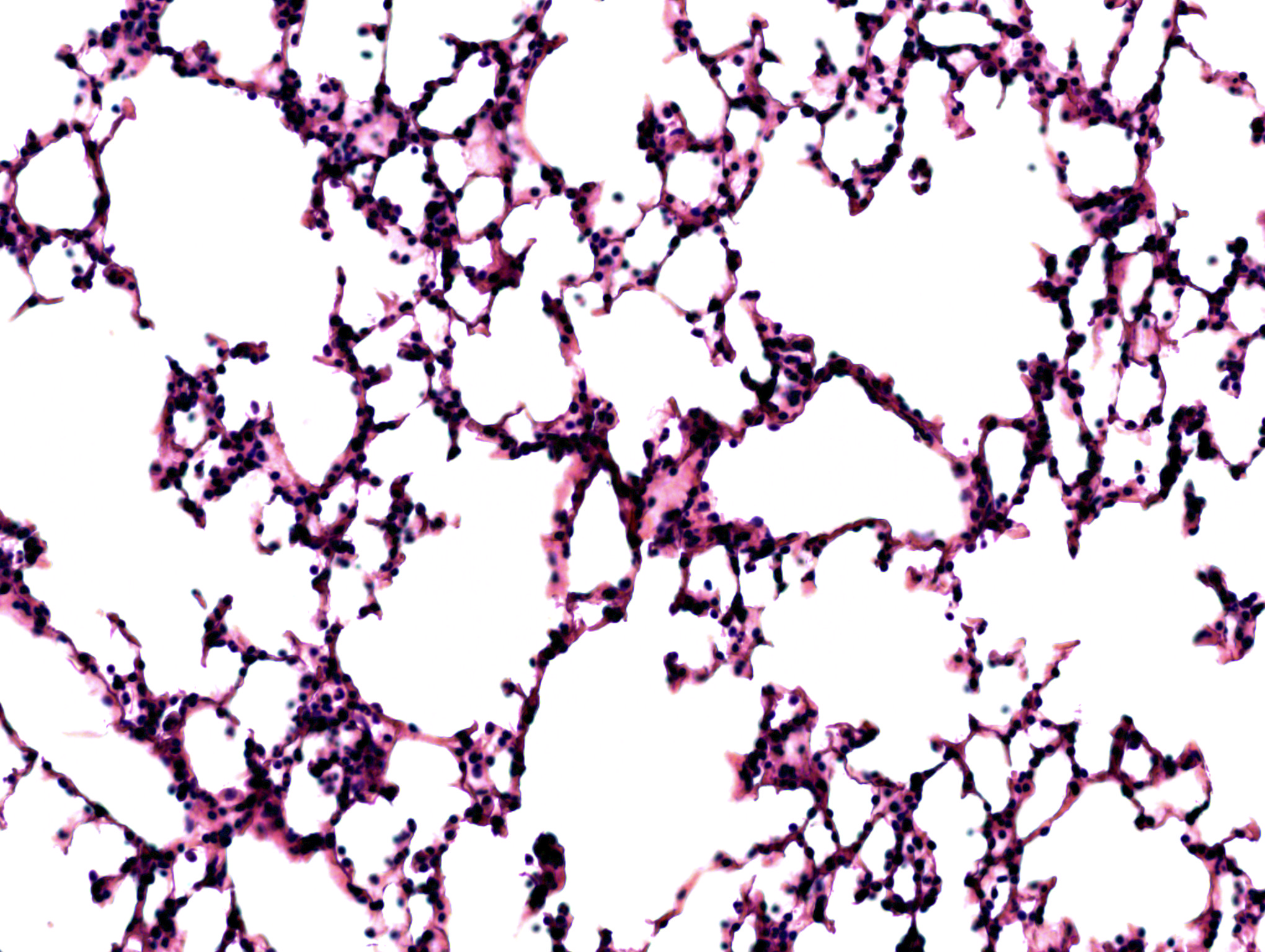

Supplement: Supplementary file 4 — Appendix Figure Source Data [file 44319_2024_180_MOESM4_ESM.zip › Appendix Figure S4/S4C/HPF shP4HA1.tif]

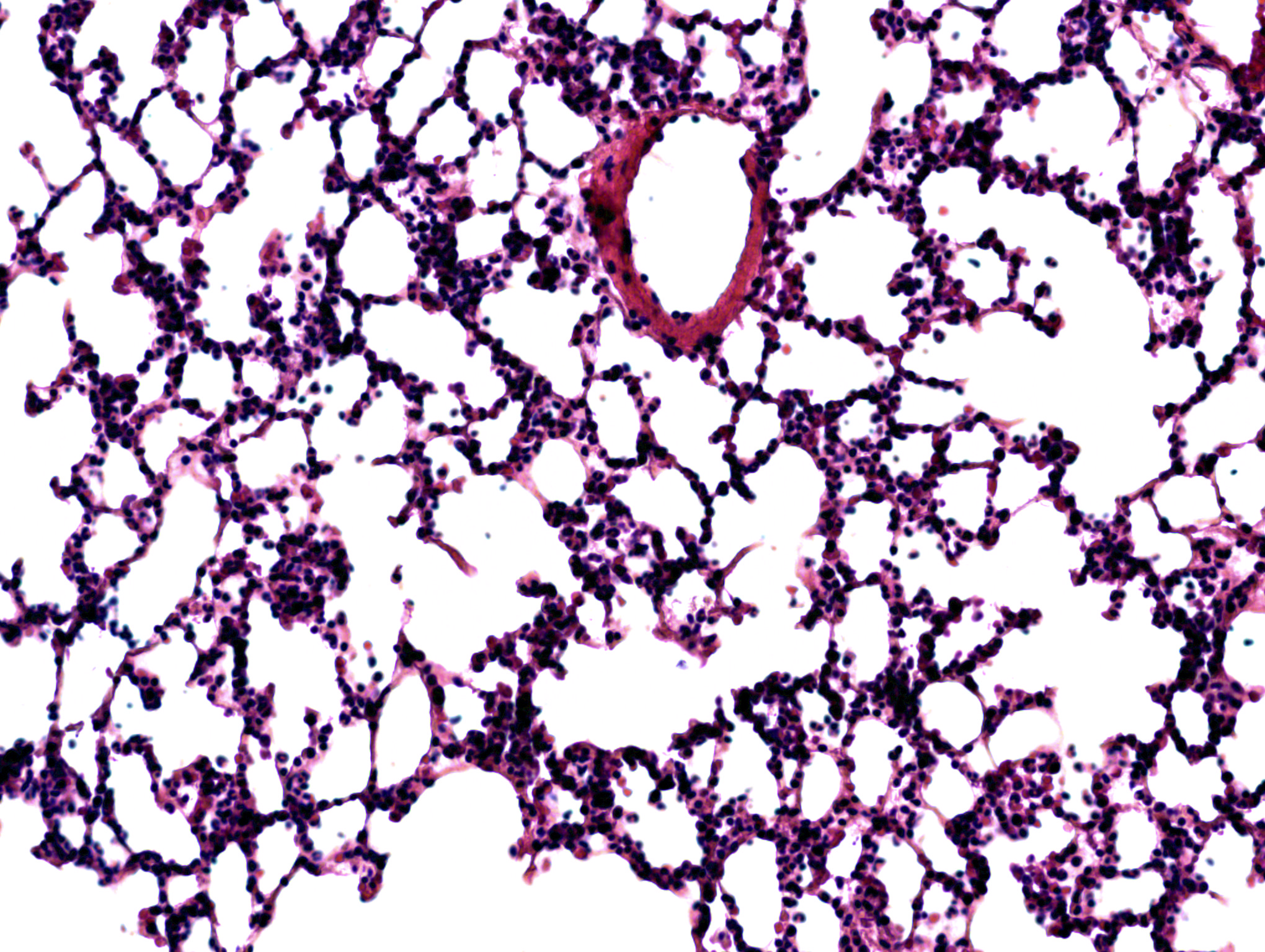

Supplement: Supplementary file 4 — Appendix Figure Source Data [file 44319_2024_180_MOESM4_ESM.zip › Appendix Figure S4/S4C/CAF shP4HA1 .tif]

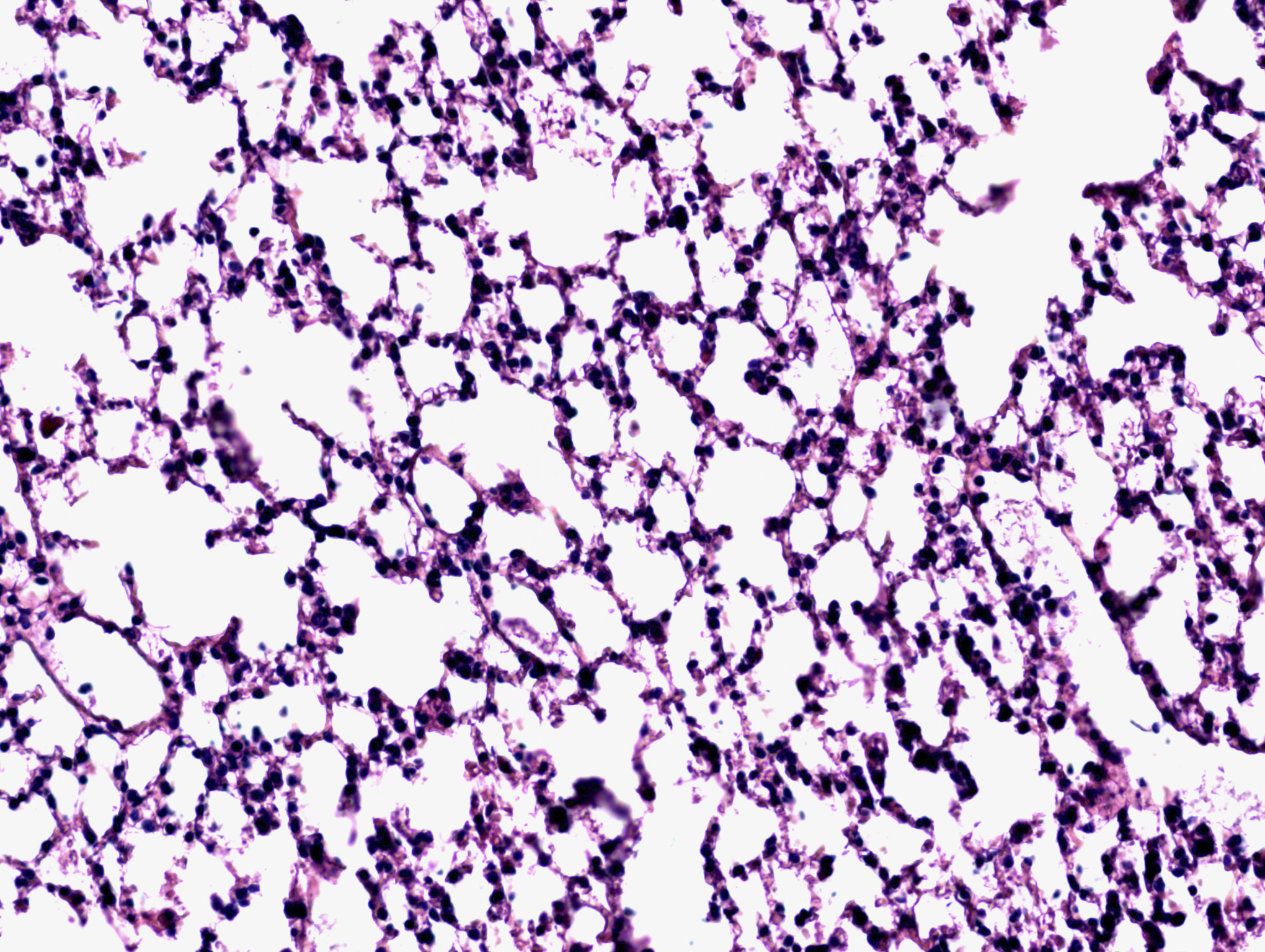

Supplement: Supplementary file 4 — Appendix Figure Source Data [file 44319_2024_180_MOESM4_ESM.zip › Appendix Figure S4/S4C/Lactate shP4HA1.tif]

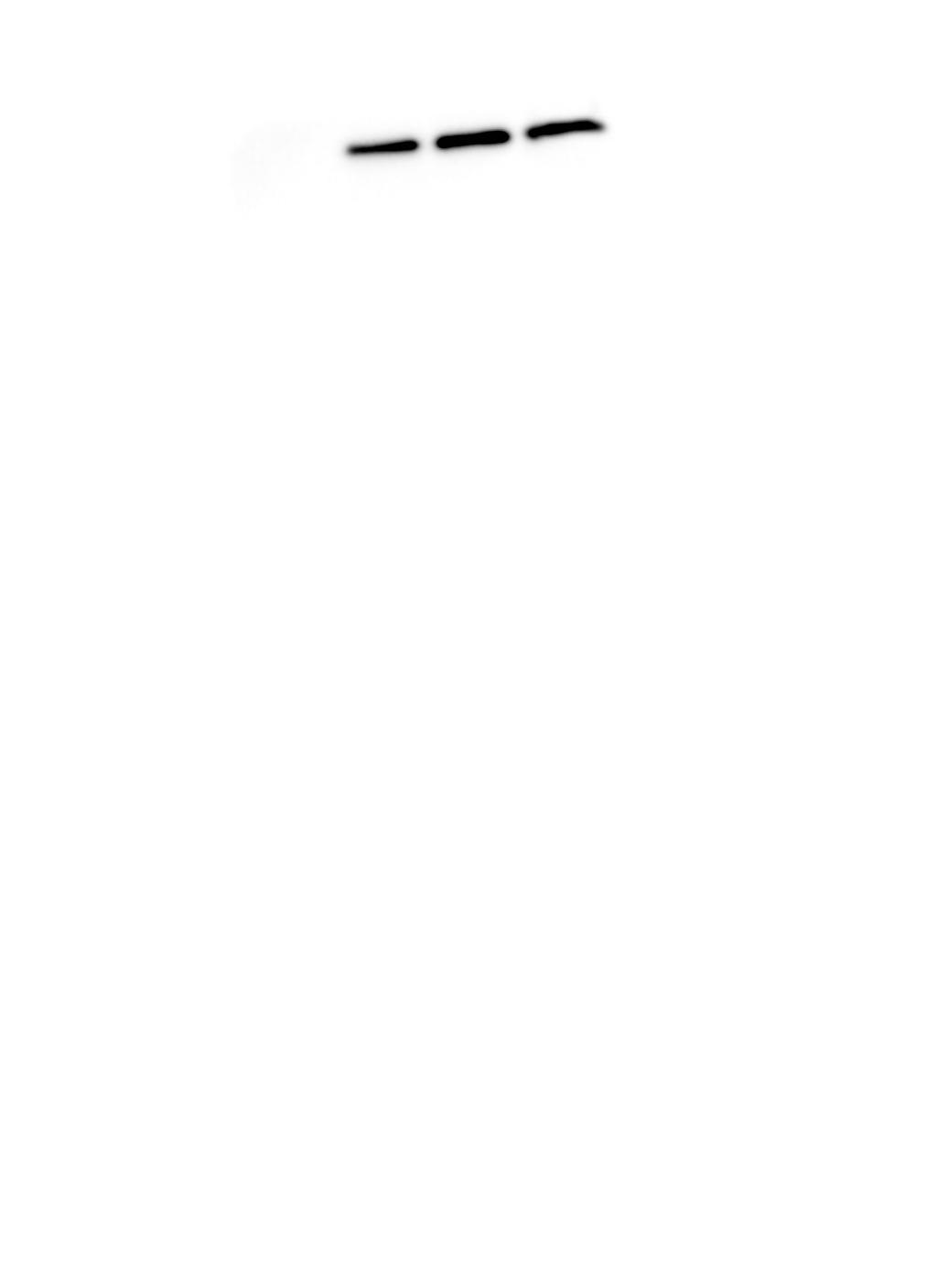

Supplement: Supplementary file 4 — Appendix Figure Source Data [file 44319_2024_180_MOESM4_ESM.zip › Appendix Figure S4/S4A/WB Actin shP4HA1.jpg]

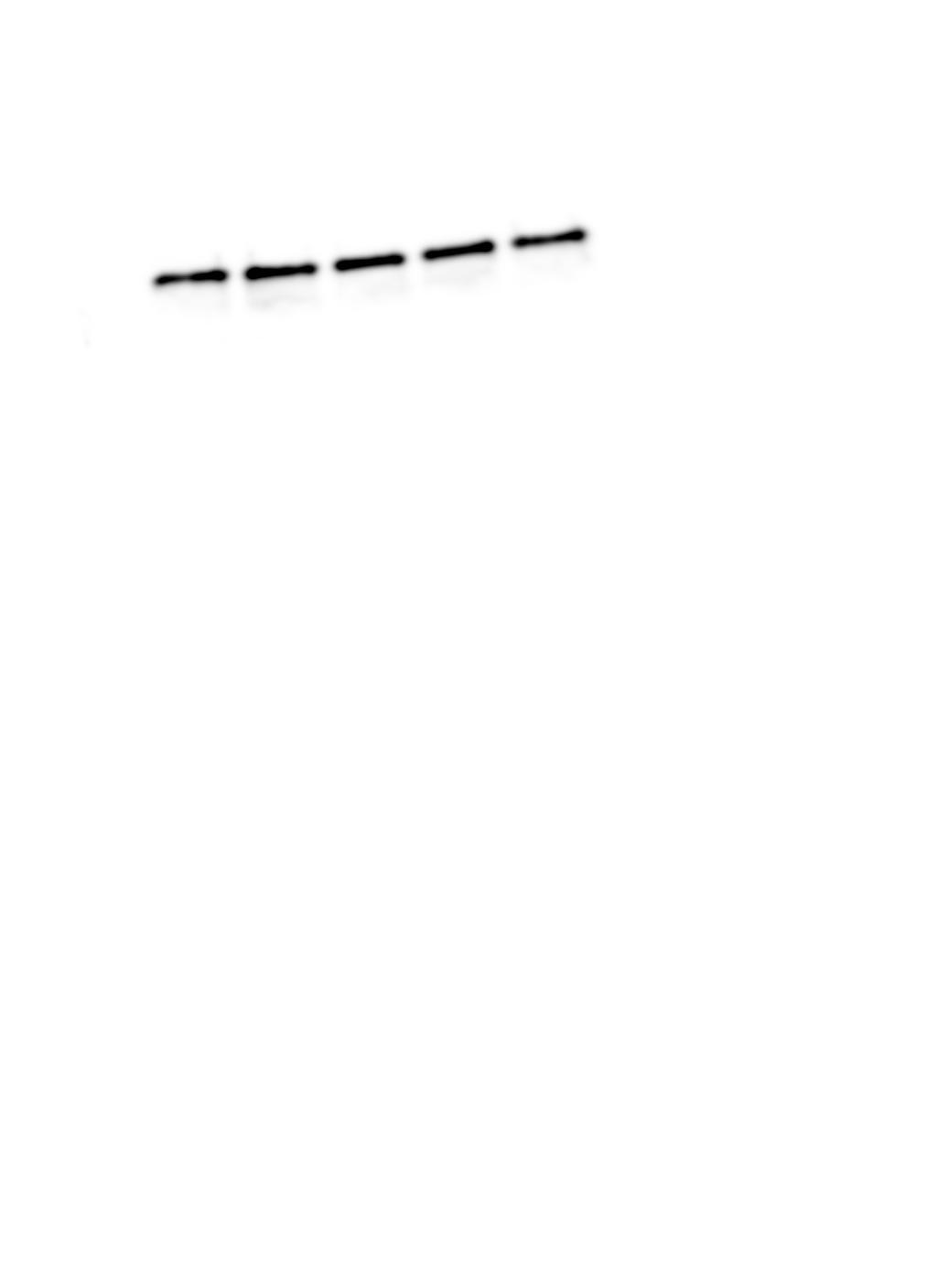

Supplement: Supplementary file 4 — Appendix Figure Source Data [file 44319_2024_180_MOESM4_ESM.zip › Appendix Figure S4/S4A/WB Actin shDDR1.jpg]

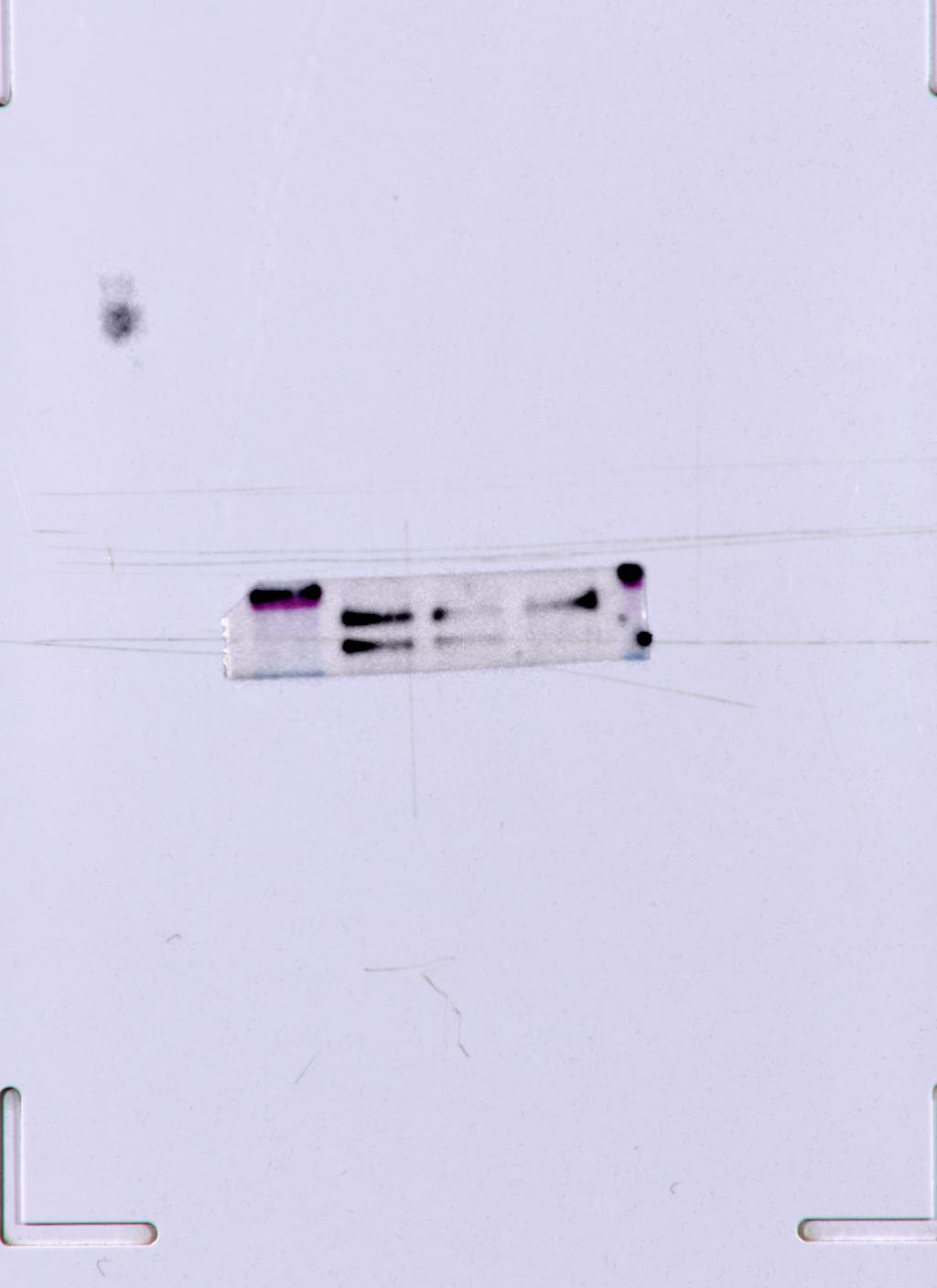

Supplement: Supplementary file 4 — Appendix Figure Source Data [file 44319_2024_180_MOESM4_ESM.zip › Appendix Figure S4/S4A/WB P4HA1 shP4HA1.jpg]

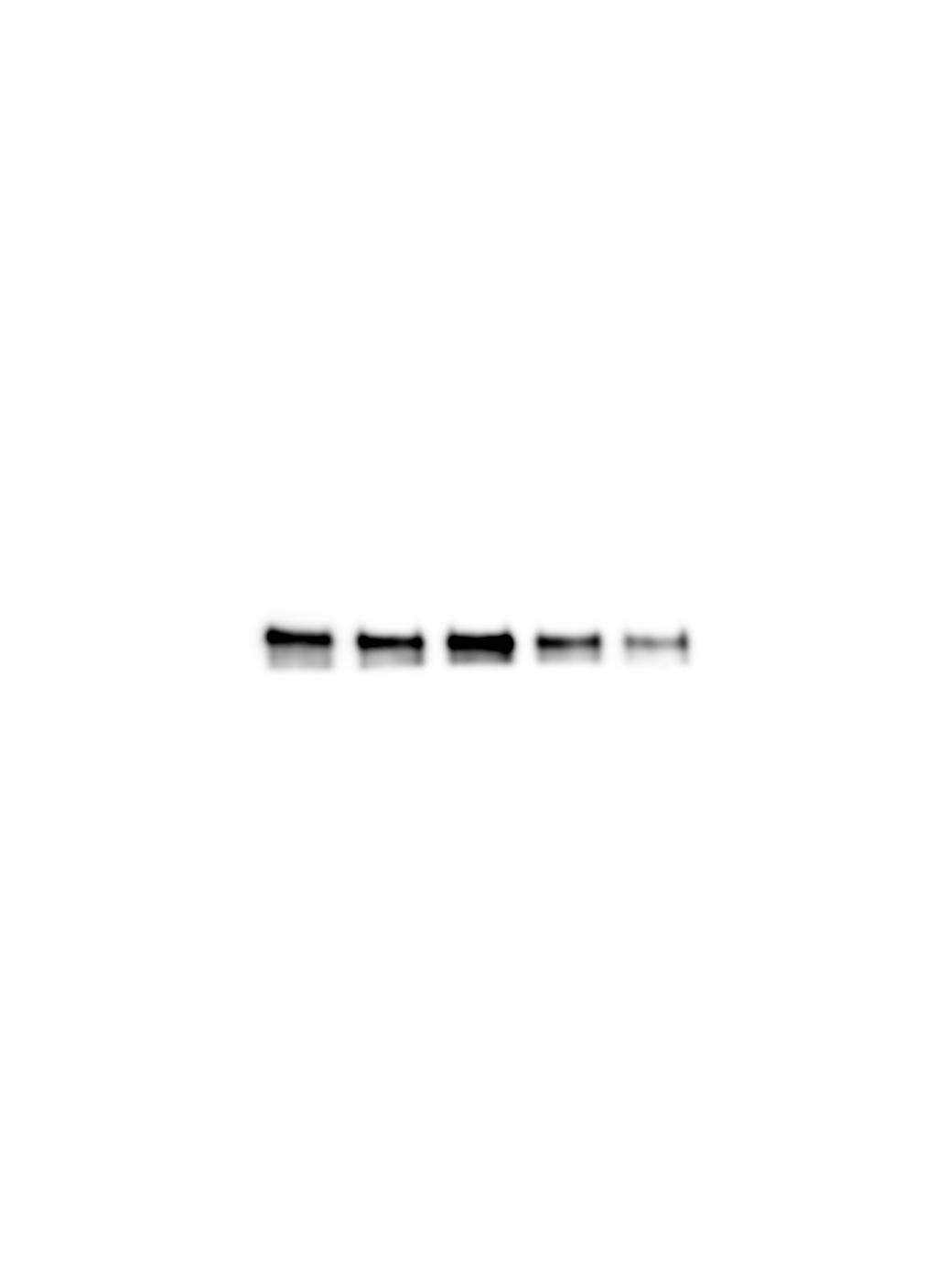

Supplement: Supplementary file 4 — Appendix Figure Source Data [file 44319_2024_180_MOESM4_ESM.zip › Appendix Figure S4/S4A/WB DDR1 shDDR1.jpg]

Appendix Figure S4a

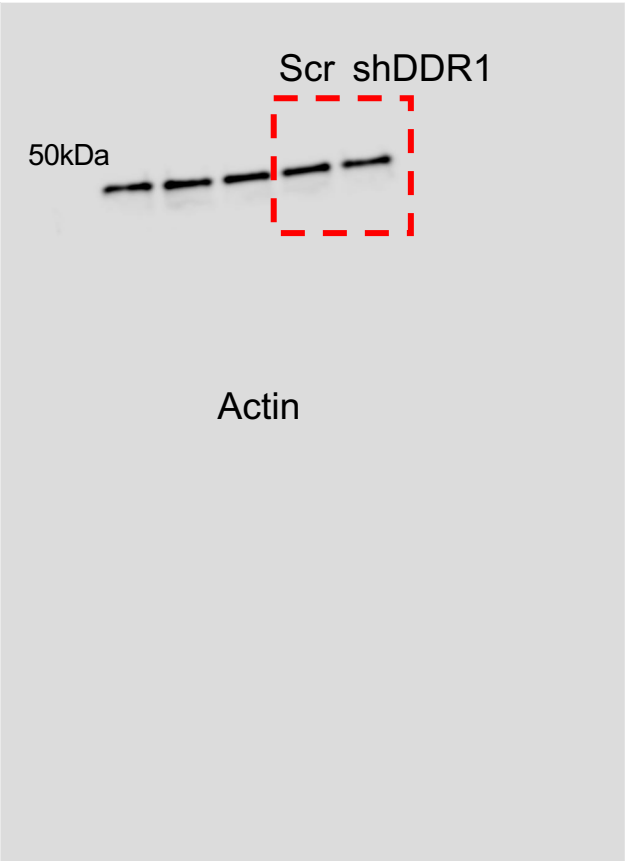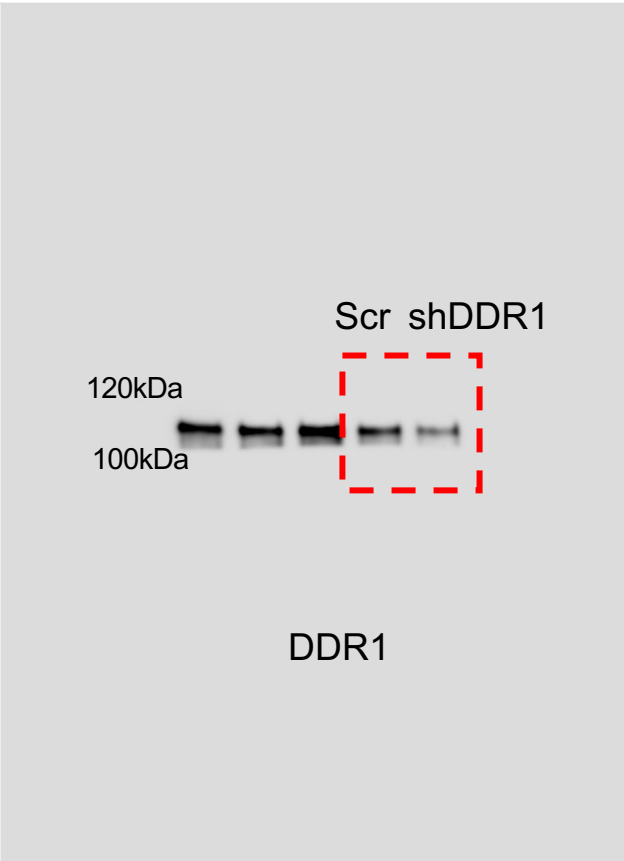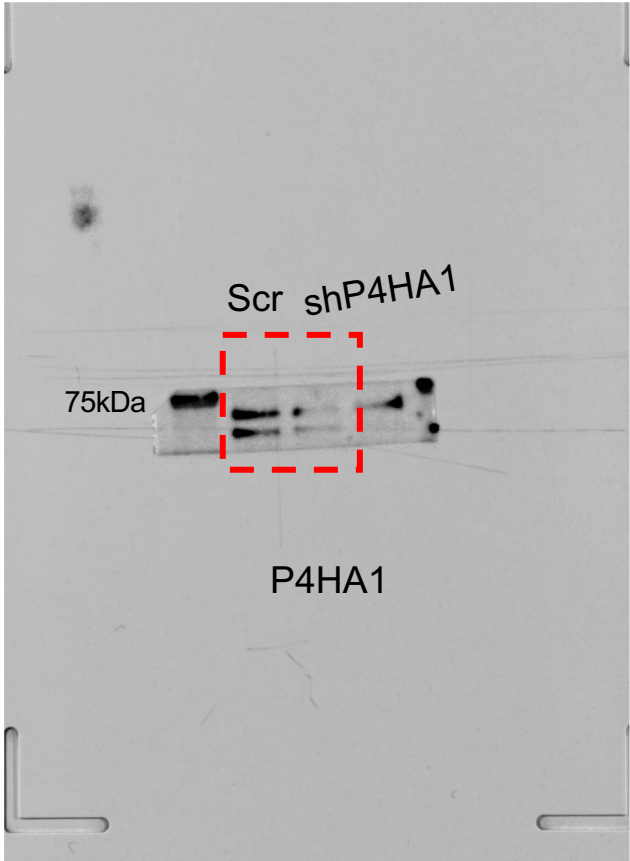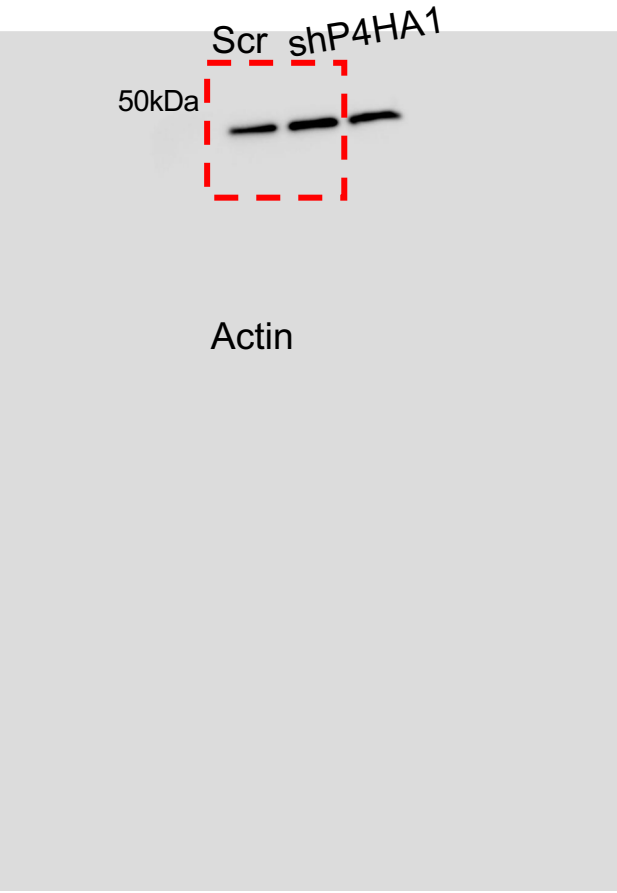

Supplement: Supplementary file 4 — Appendix Figure Source Data [file 44319_2024_180_MOESM4_ESM.zip › Appendix Figure S4/S4A/Appendix Figure S4a WB.pdf]

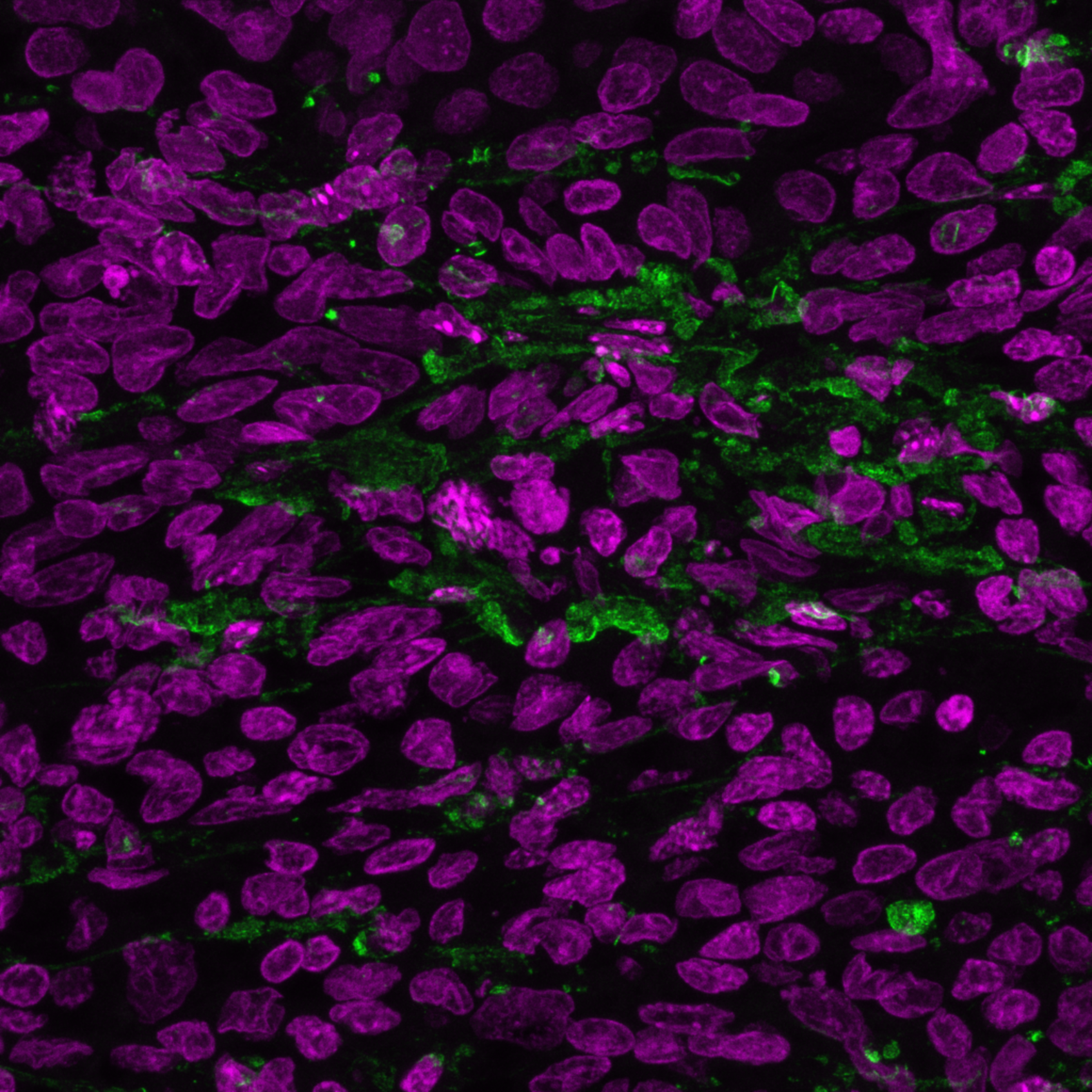

Supplement: Supplementary file 4 — Appendix Figure Source Data [file 44319_2024_180_MOESM4_ESM.zip › Appendix Figure S4/S4B/IF/Col1a1 scr_Lactate.tif]

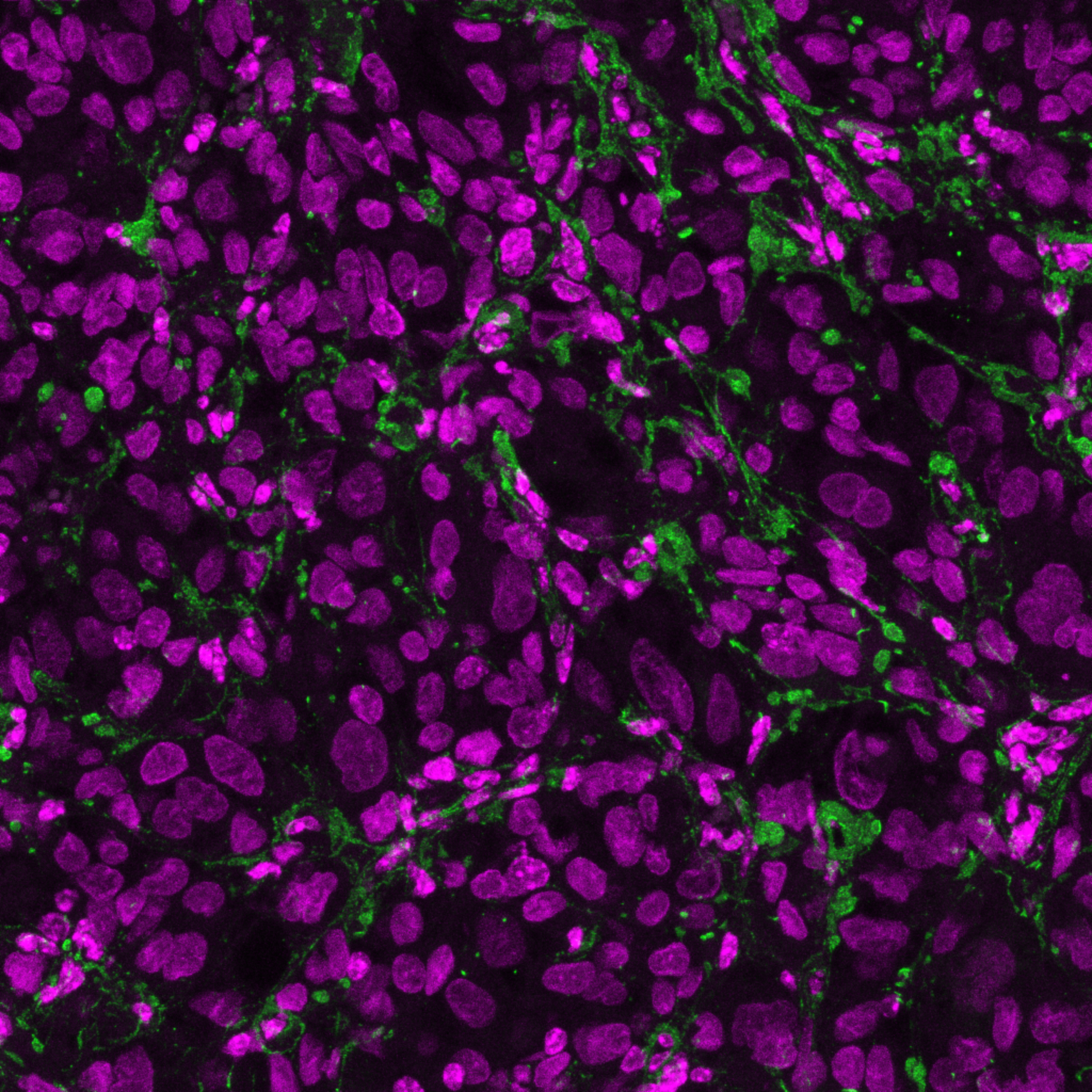

Supplement: Supplementary file 4 — Appendix Figure Source Data [file 44319_2024_180_MOESM4_ESM.zip › Appendix Figure S4/S4B/IF/Col1a1 scr_CAF.tif]

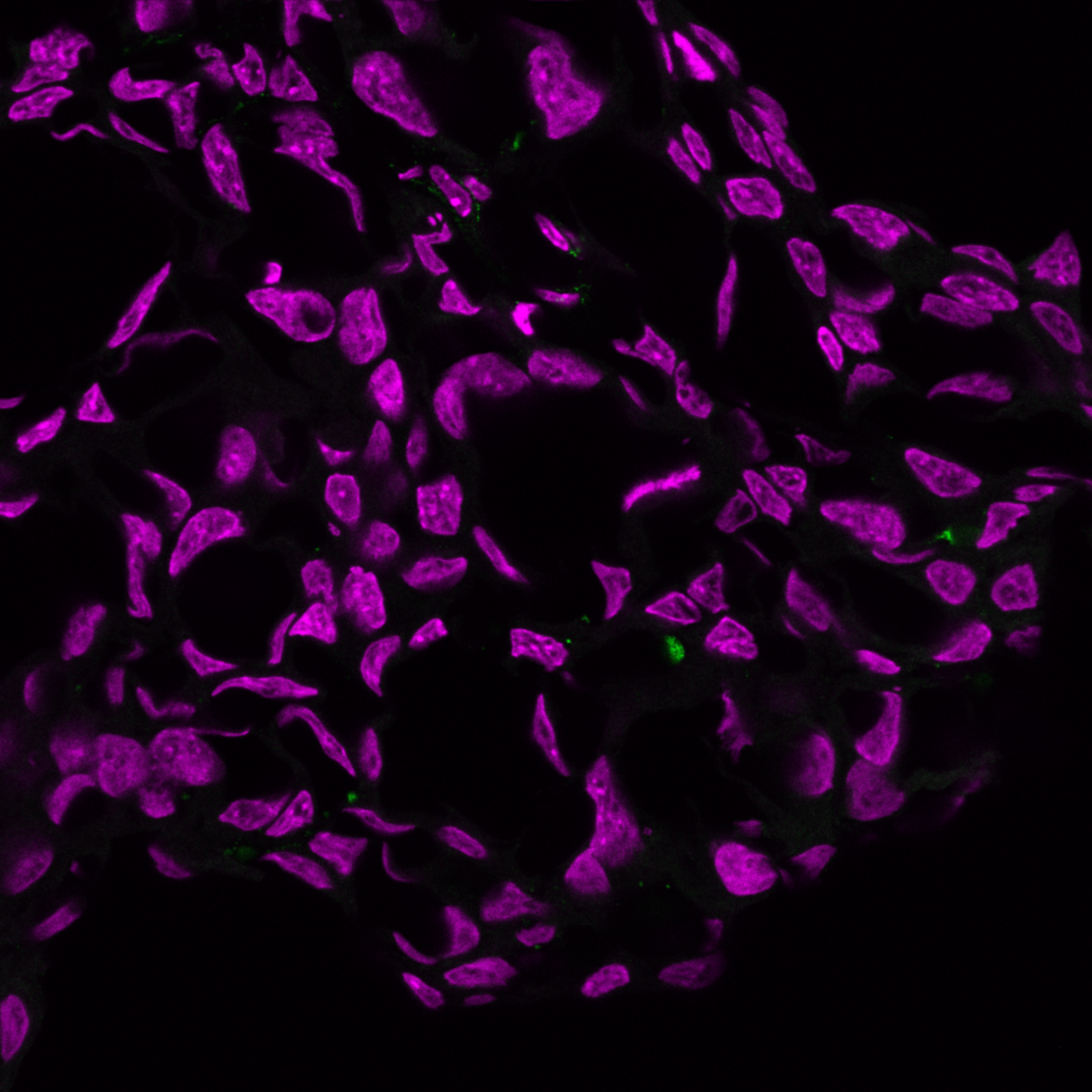

Supplement: Supplementary file 4 — Appendix Figure Source Data [file 44319_2024_180_MOESM4_ESM.zip › Appendix Figure S4/S4B/IF/Col1a1 scr_HPF.tif]

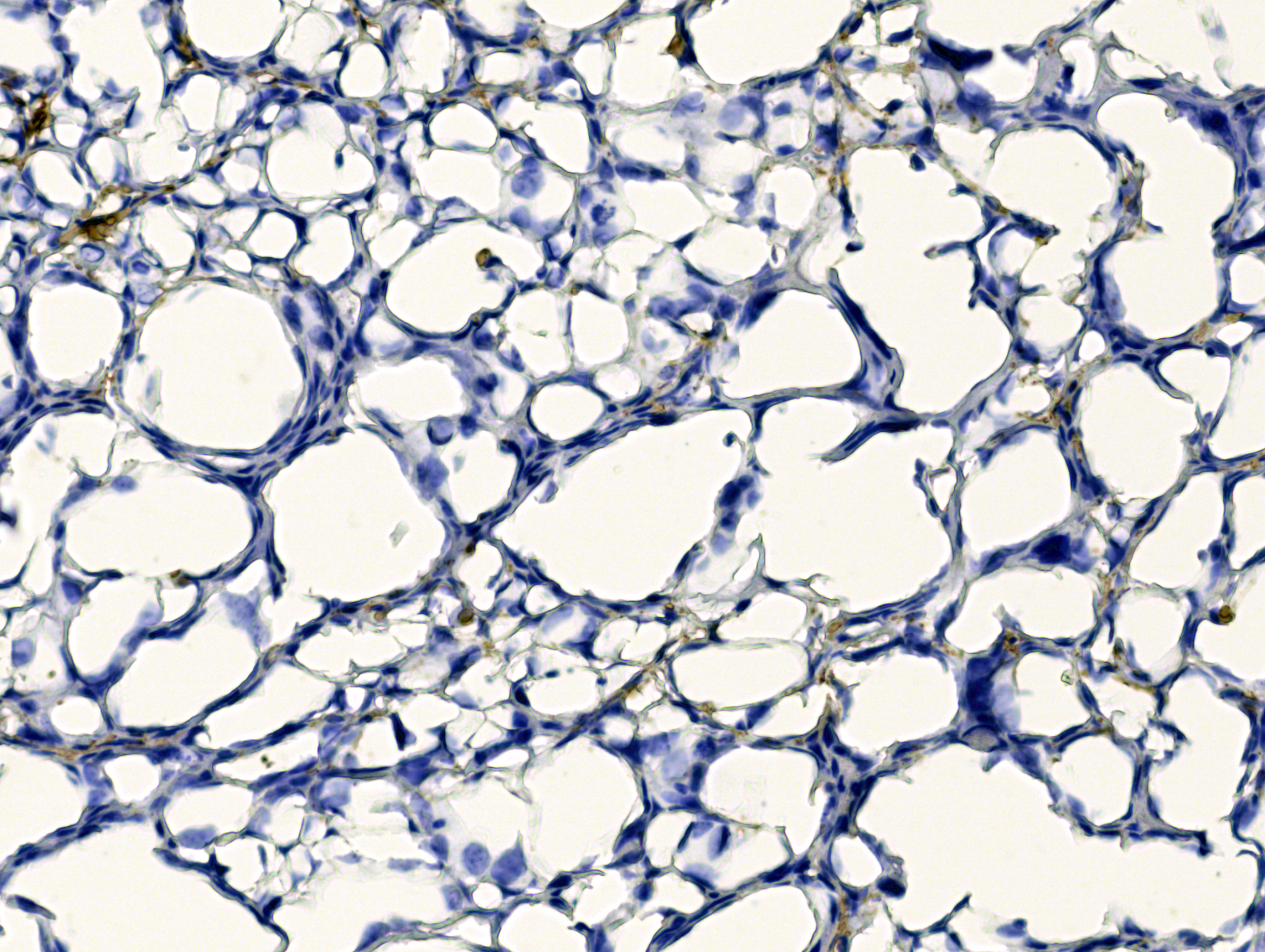

Supplement: Supplementary file 4 — Appendix Figure Source Data [file 44319_2024_180_MOESM4_ESM.zip › Appendix Figure S4/S4B/IHC/Col1a1 HPF_scr.tif]

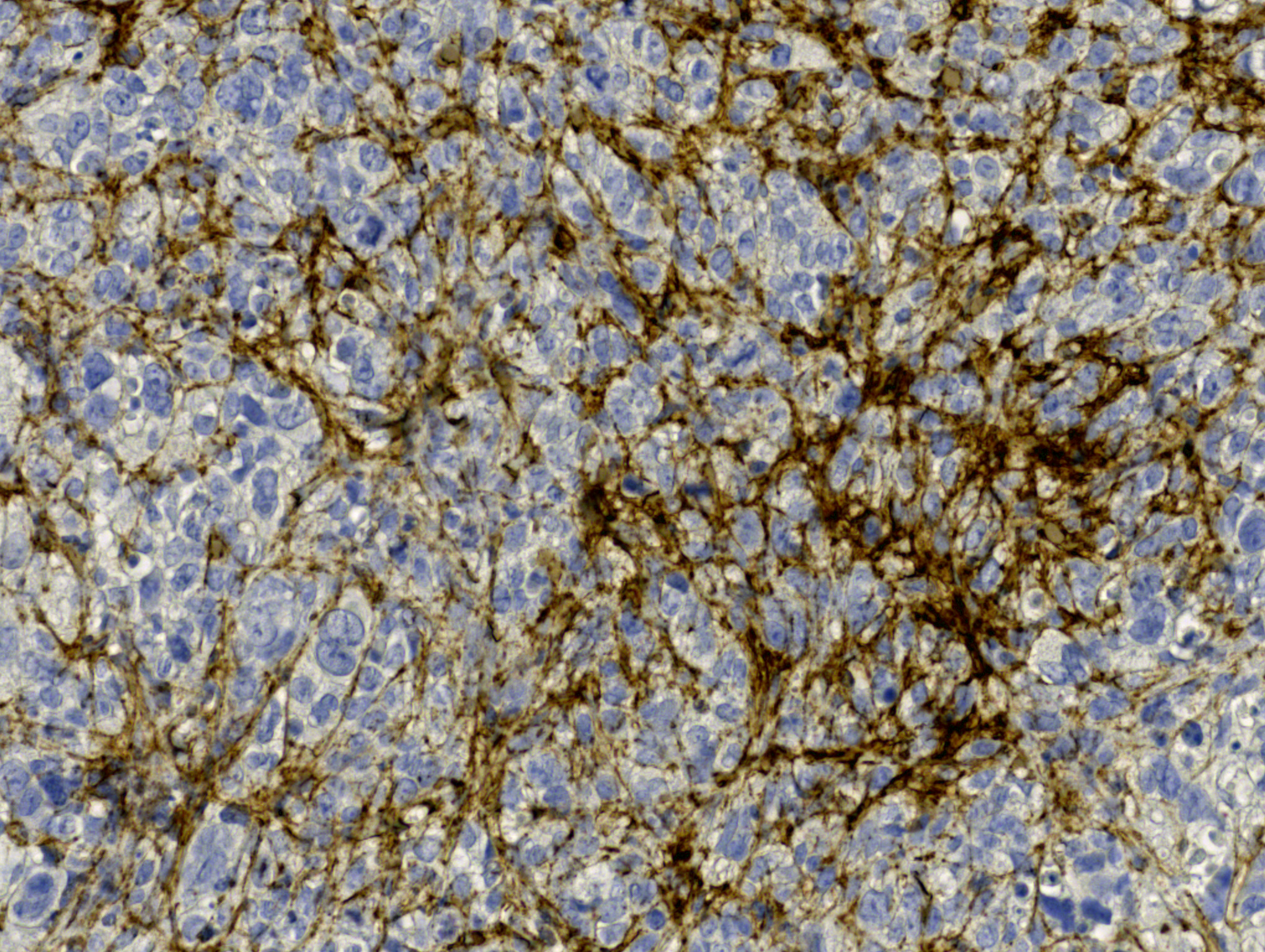

Supplement: Supplementary file 4 — Appendix Figure Source Data [file 44319_2024_180_MOESM4_ESM.zip › Appendix Figure S4/S4B/IHC/Col1a1 Lactate_Scr.tif]

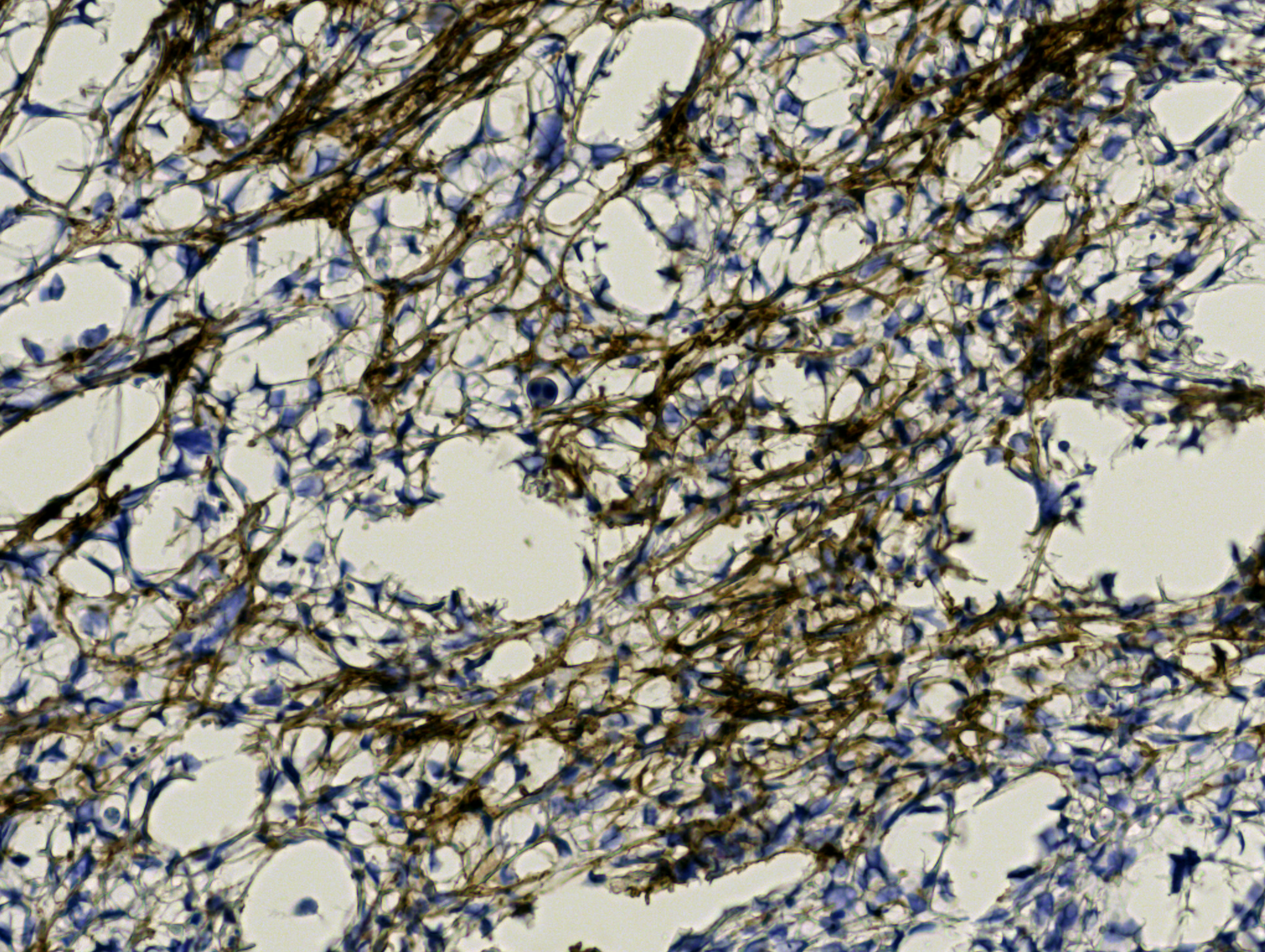

Supplement: Supplementary file 4 — Appendix Figure Source Data [file 44319_2024_180_MOESM4_ESM.zip › Appendix Figure S4/S4B/IHC/Col1a1 CAF_Scr.tif]

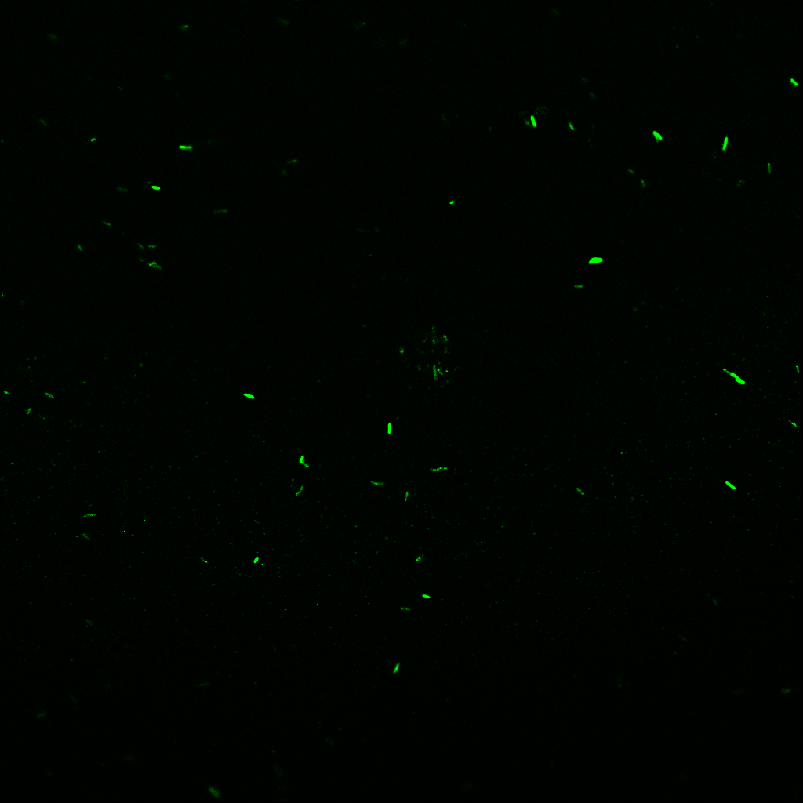

Supplement: Supplementary file 5 — Expanded View Figure Source Data [file 44319_2024_180_MOESM5_ESM.zip › EV Figure Source/EV1/EV1C/extraCol1_HPF-CM.tif]

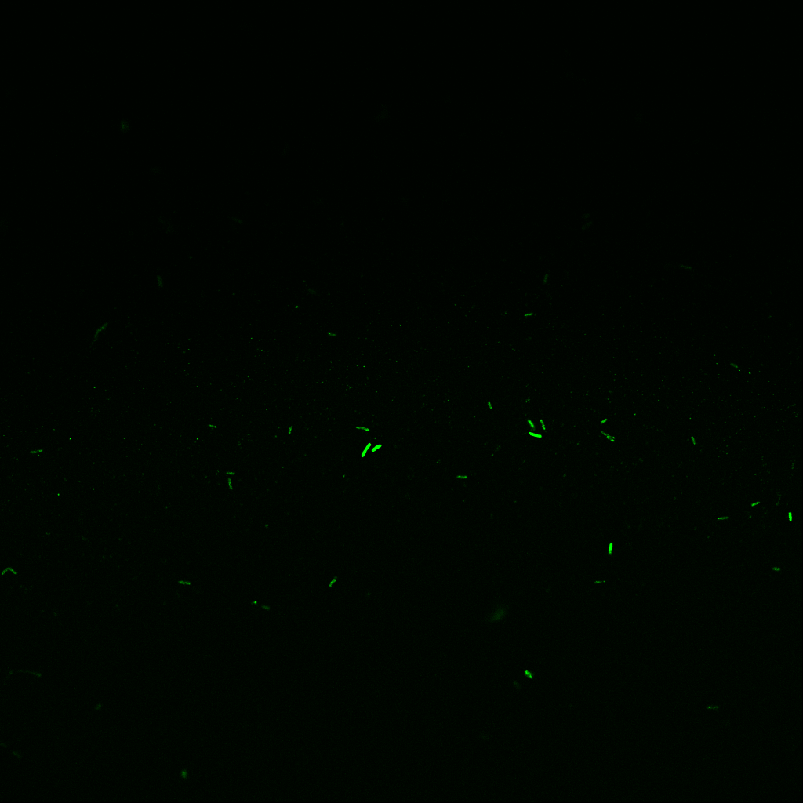

Supplement: Supplementary file 5 — Expanded View Figure Source Data [file 44319_2024_180_MOESM5_ESM.zip › EV Figure Source/EV1/EV1C/extraCol1_HPF-CM MCT1i.tif]

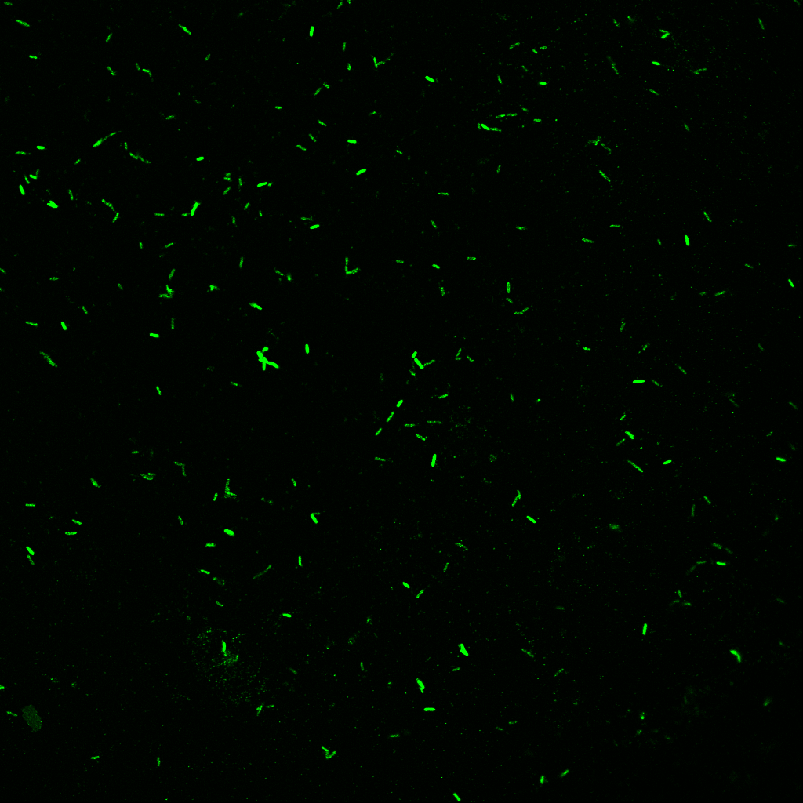

Supplement: Supplementary file 5 — Expanded View Figure Source Data [file 44319_2024_180_MOESM5_ESM.zip › EV Figure Source/EV1/EV1C/extraCol1_LA.tif]

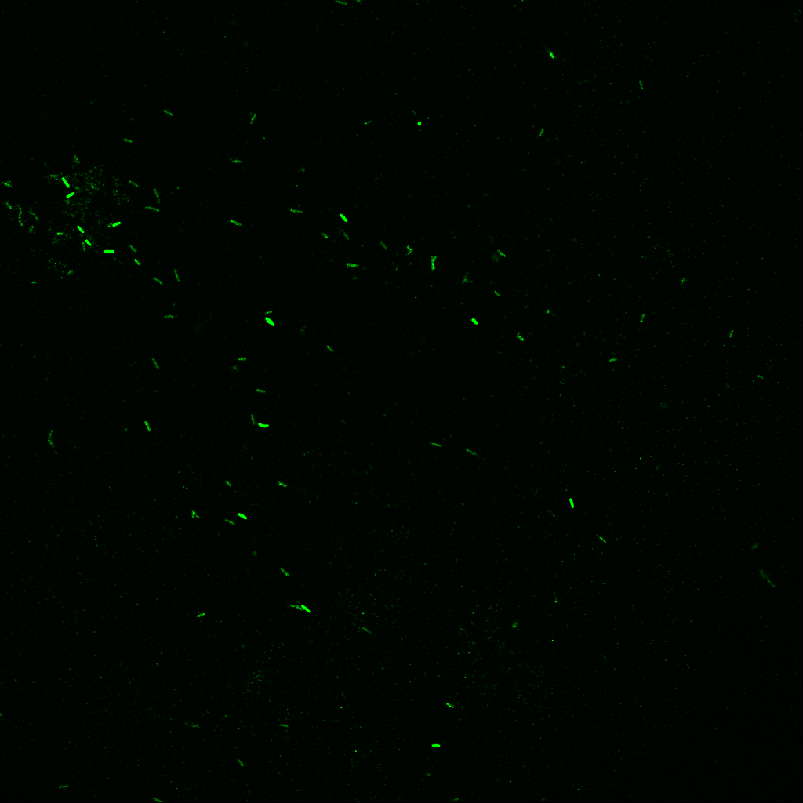

Supplement: Supplementary file 5 — Expanded View Figure Source Data [file 44319_2024_180_MOESM5_ESM.zip › EV Figure Source/EV1/EV1C/extraCol1_CAF-CM MCT1i.tif]

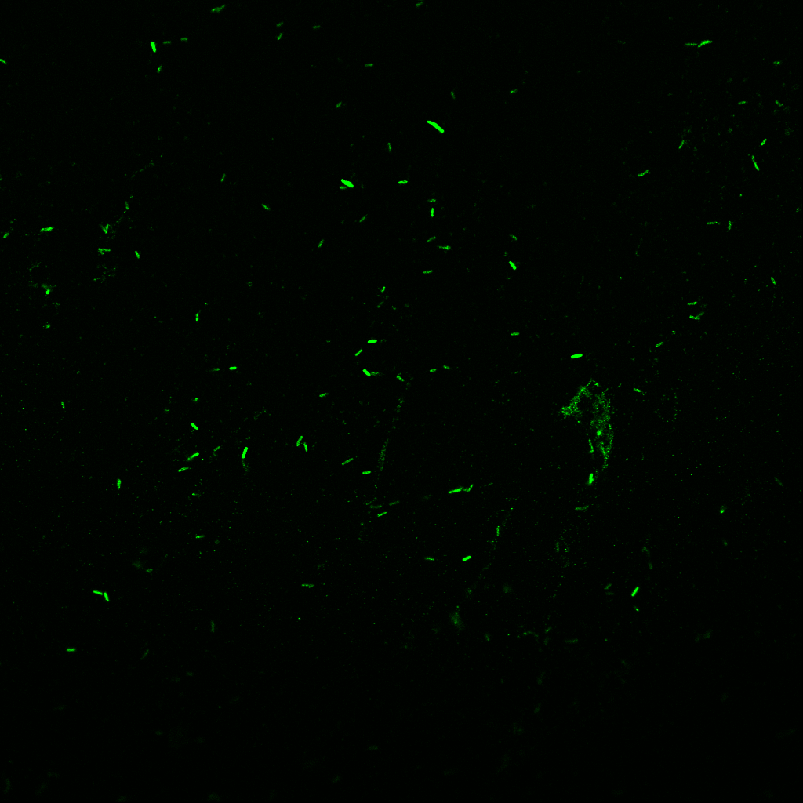

Supplement: Supplementary file 5 — Expanded View Figure Source Data [file 44319_2024_180_MOESM5_ESM.zip › EV Figure Source/EV1/EV1C/extraCol1 CAF_CM.tif]

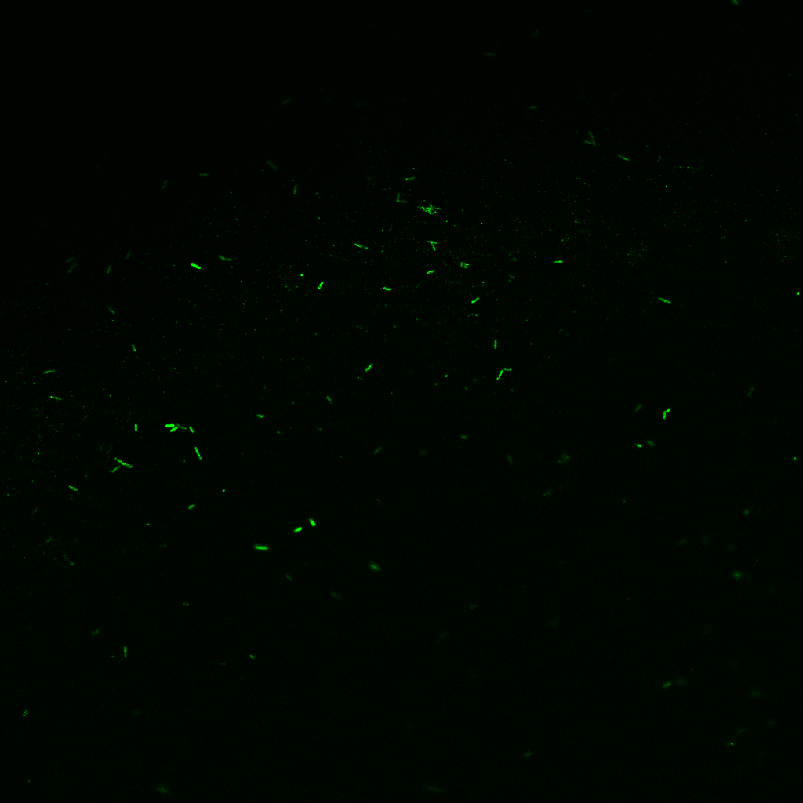

Supplement: Supplementary file 5 — Expanded View Figure Source Data [file 44319_2024_180_MOESM5_ESM.zip › EV Figure Source/EV1/EV1C/extraCol1_LA mCT1i.tif]

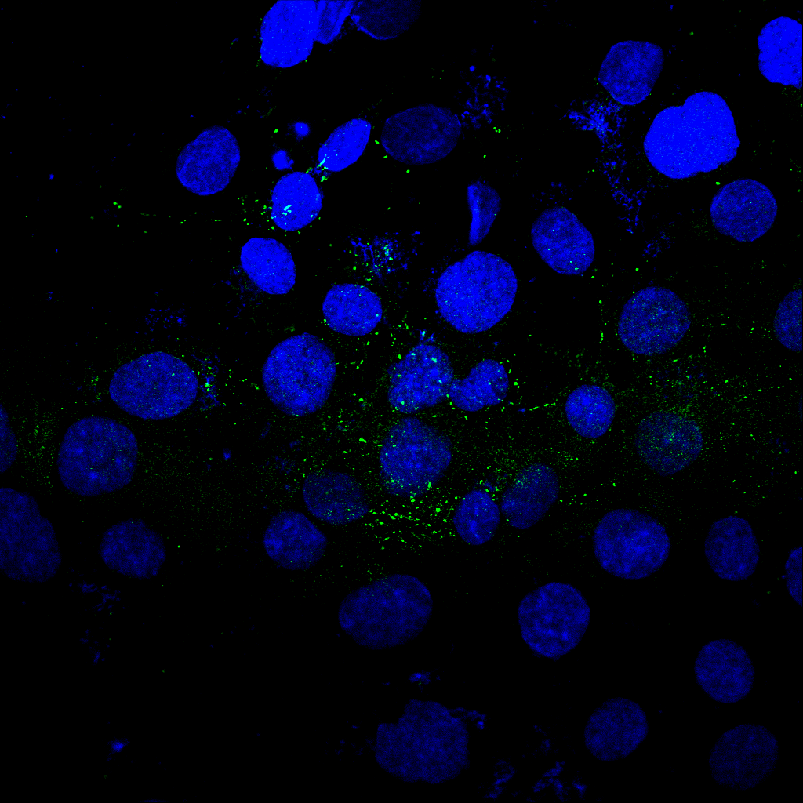

Supplement: Supplementary file 5 — Expanded View Figure Source Data [file 44319_2024_180_MOESM5_ESM.zip › EV Figure Source/EV1/EV1D/CAF-CM siCTR.tif]

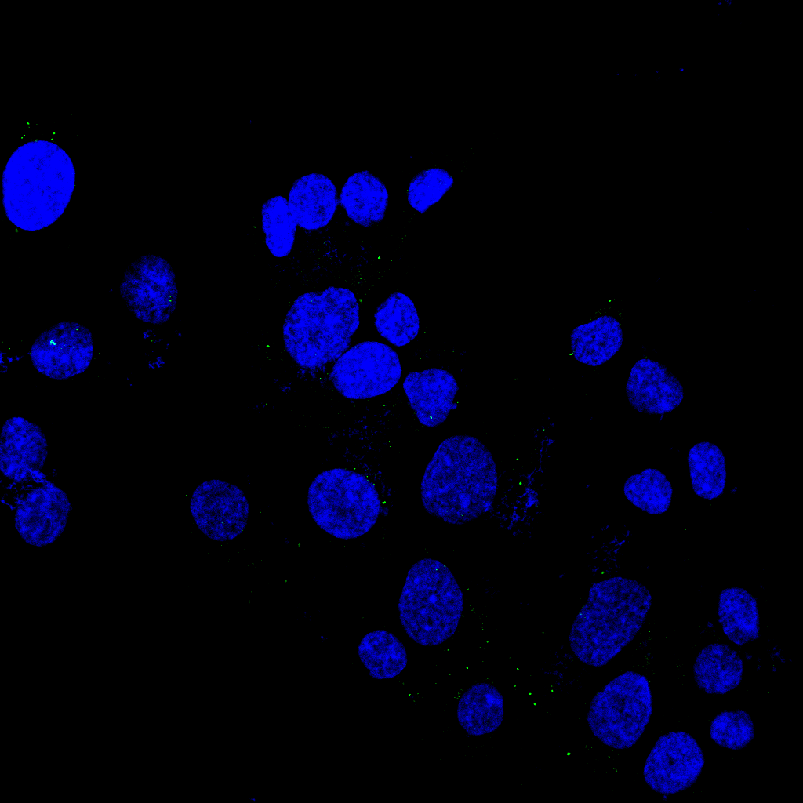

Supplement: Supplementary file 5 — Expanded View Figure Source Data [file 44319_2024_180_MOESM5_ESM.zip › EV Figure Source/EV1/EV1D/Lactate siMCT1.tif]

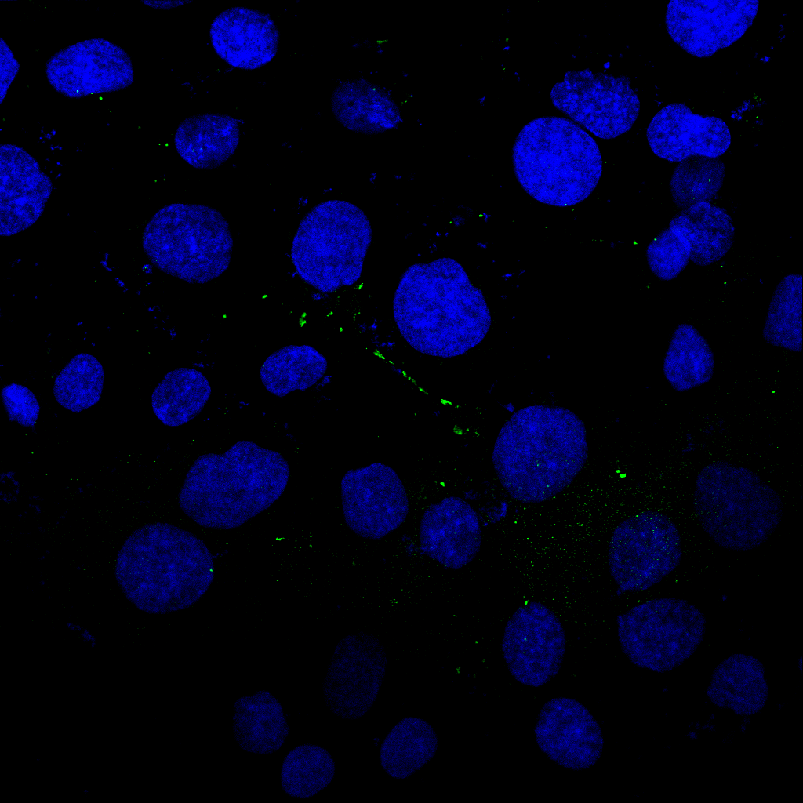

Supplement: Supplementary file 5 — Expanded View Figure Source Data [file 44319_2024_180_MOESM5_ESM.zip › EV Figure Source/EV1/EV1D/HPF-CM siCTR.tif]

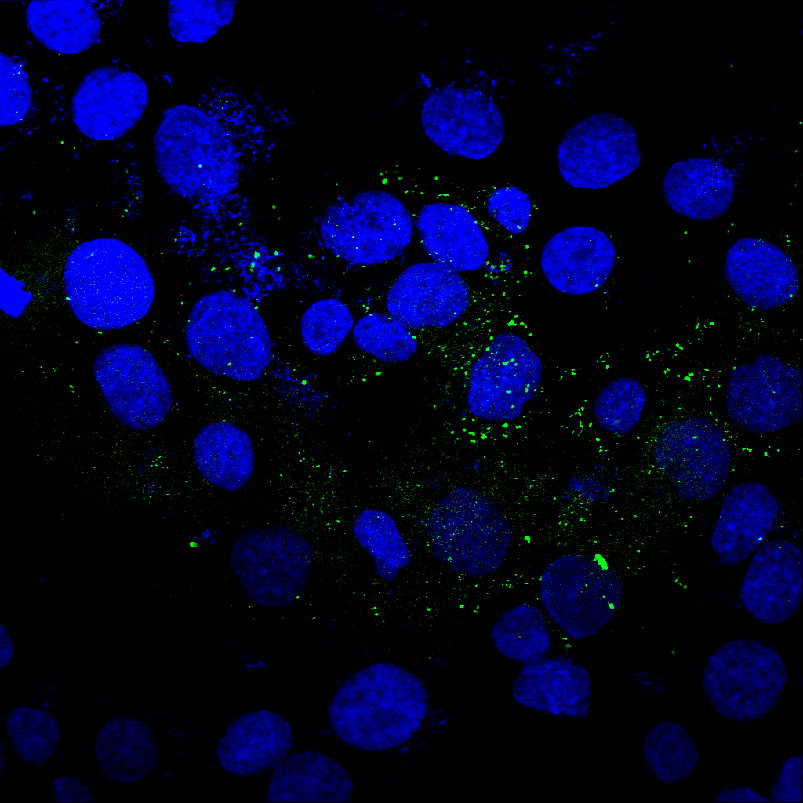

Supplement: Supplementary file 5 — Expanded View Figure Source Data [file 44319_2024_180_MOESM5_ESM.zip › EV Figure Source/EV1/EV1D/Lactate siCTR.tif]

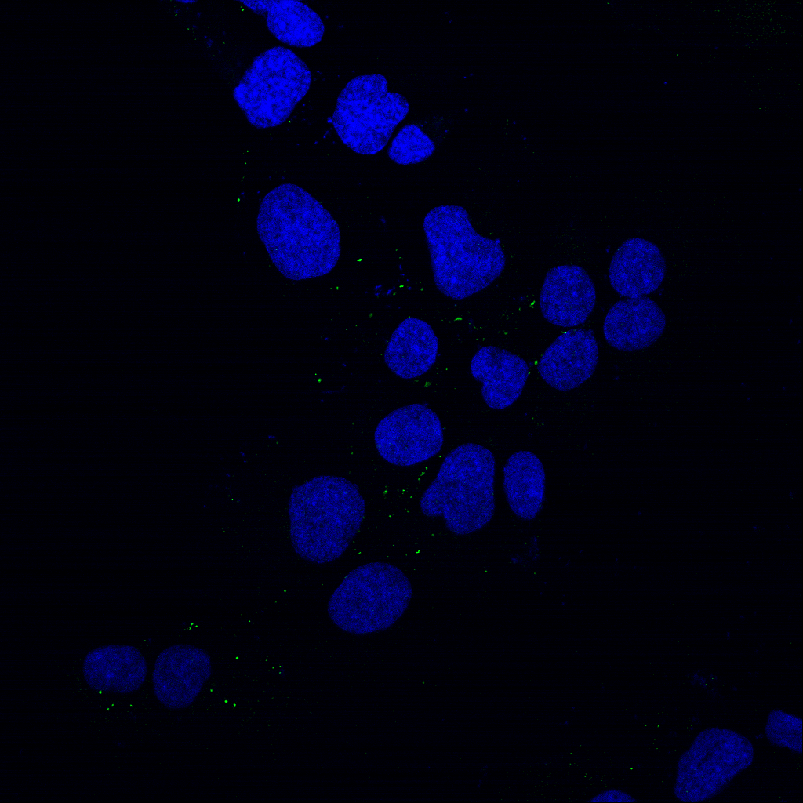

Supplement: Supplementary file 5 — Expanded View Figure Source Data [file 44319_2024_180_MOESM5_ESM.zip › EV Figure Source/EV1/EV1D/HPF-CM siMCT1.tif]

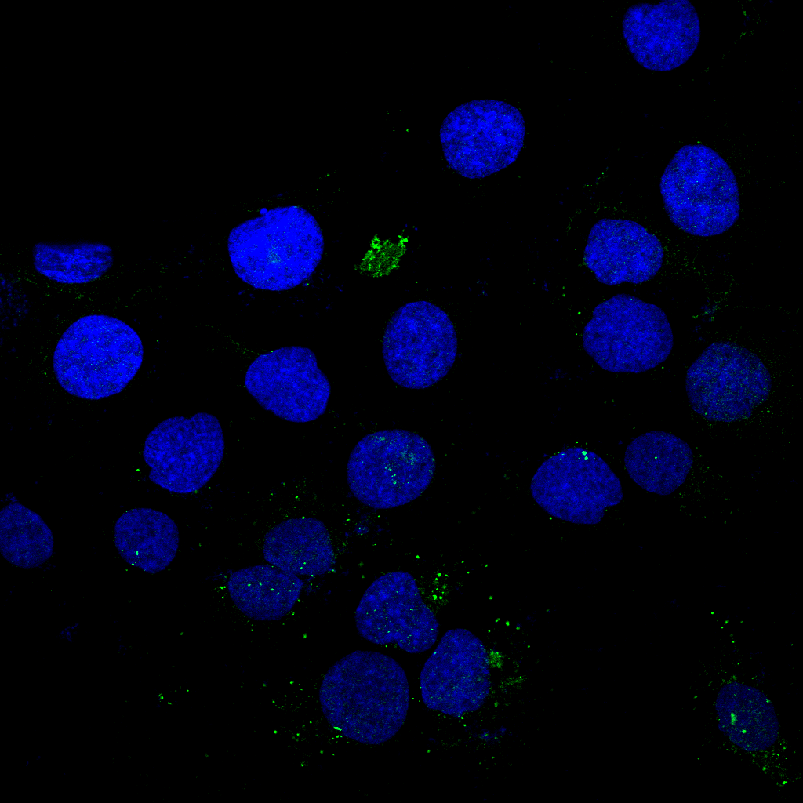

Supplement: Supplementary file 5 — Expanded View Figure Source Data [file 44319_2024_180_MOESM5_ESM.zip › EV Figure Source/EV1/EV1D/CAF-CM siMCT1.tif]

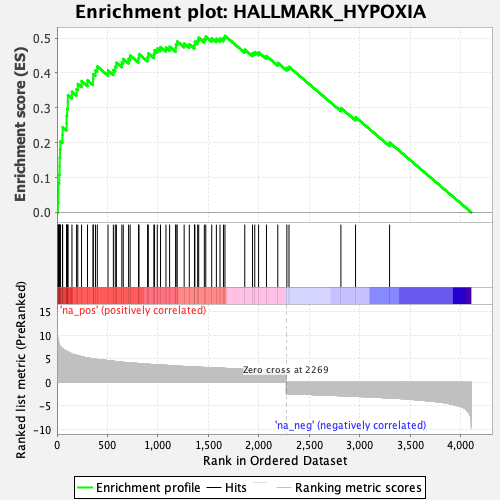

Supplement: Supplementary file 5 — Expanded View Figure Source Data [file 44319_2024_180_MOESM5_ESM.zip › EV Figure Source/EV1/EV1E/enplot_HALLMARK_HYPOXIA_125.png]

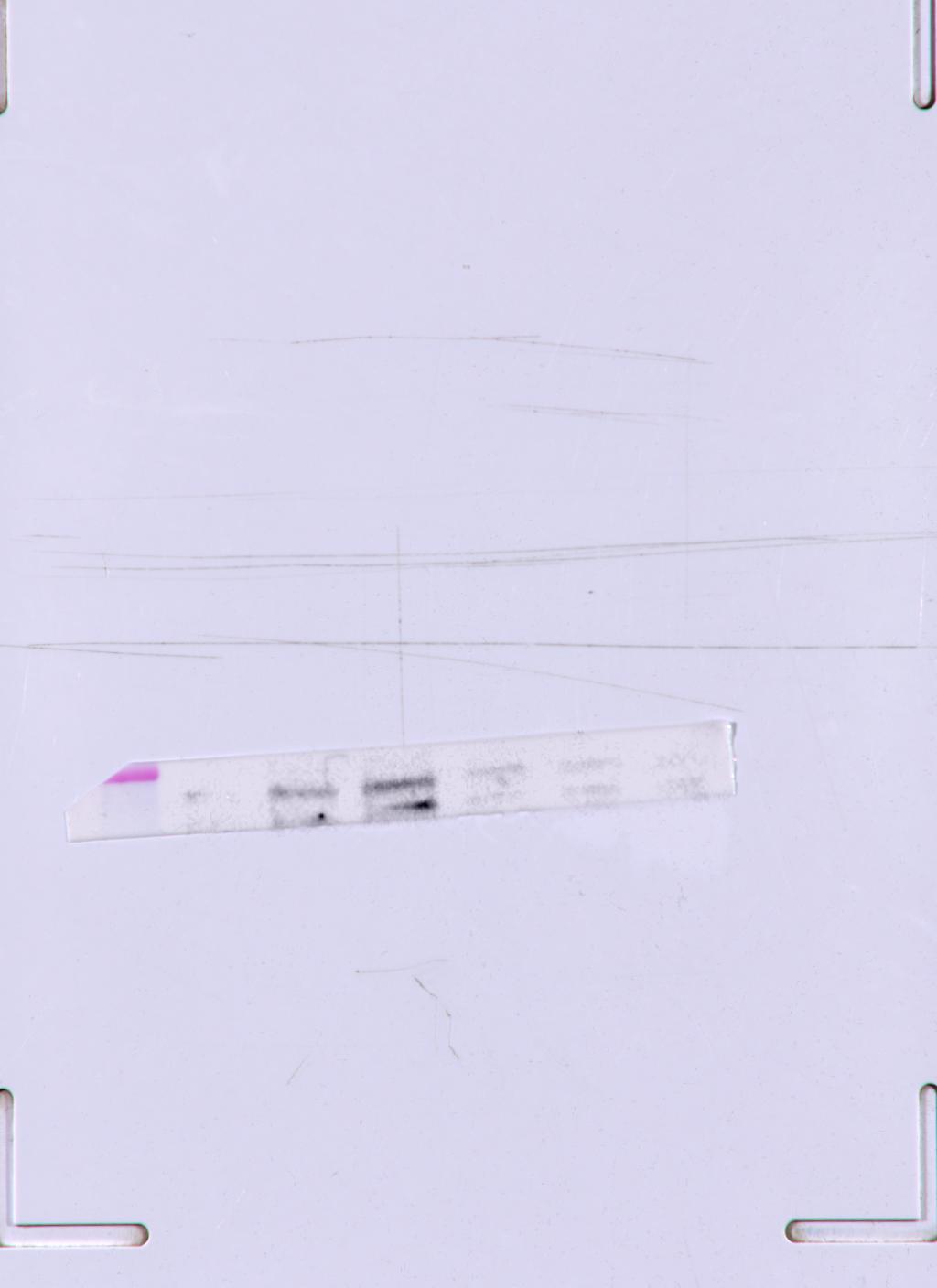

Supplement: Supplementary file 5 — Expanded View Figure Source Data [file 44319_2024_180_MOESM5_ESM.zip › EV Figure Source/EV1/EV1G/WB P4HA1_simct1.jpg]

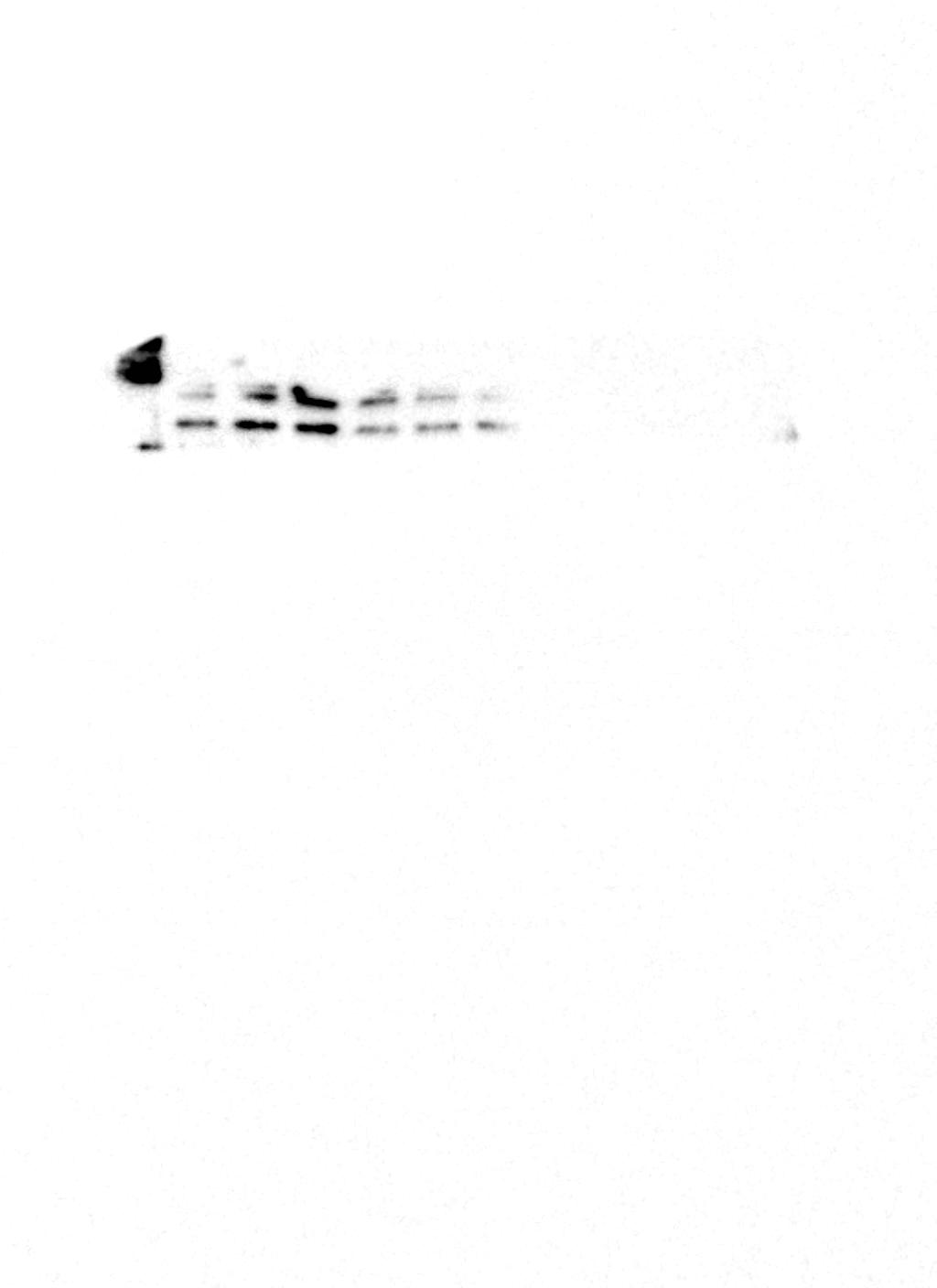

Supplement: Supplementary file 5 — Expanded View Figure Source Data [file 44319_2024_180_MOESM5_ESM.zip › EV Figure Source/EV1/EV1G/WB (PC3) P4HA1.jpg]

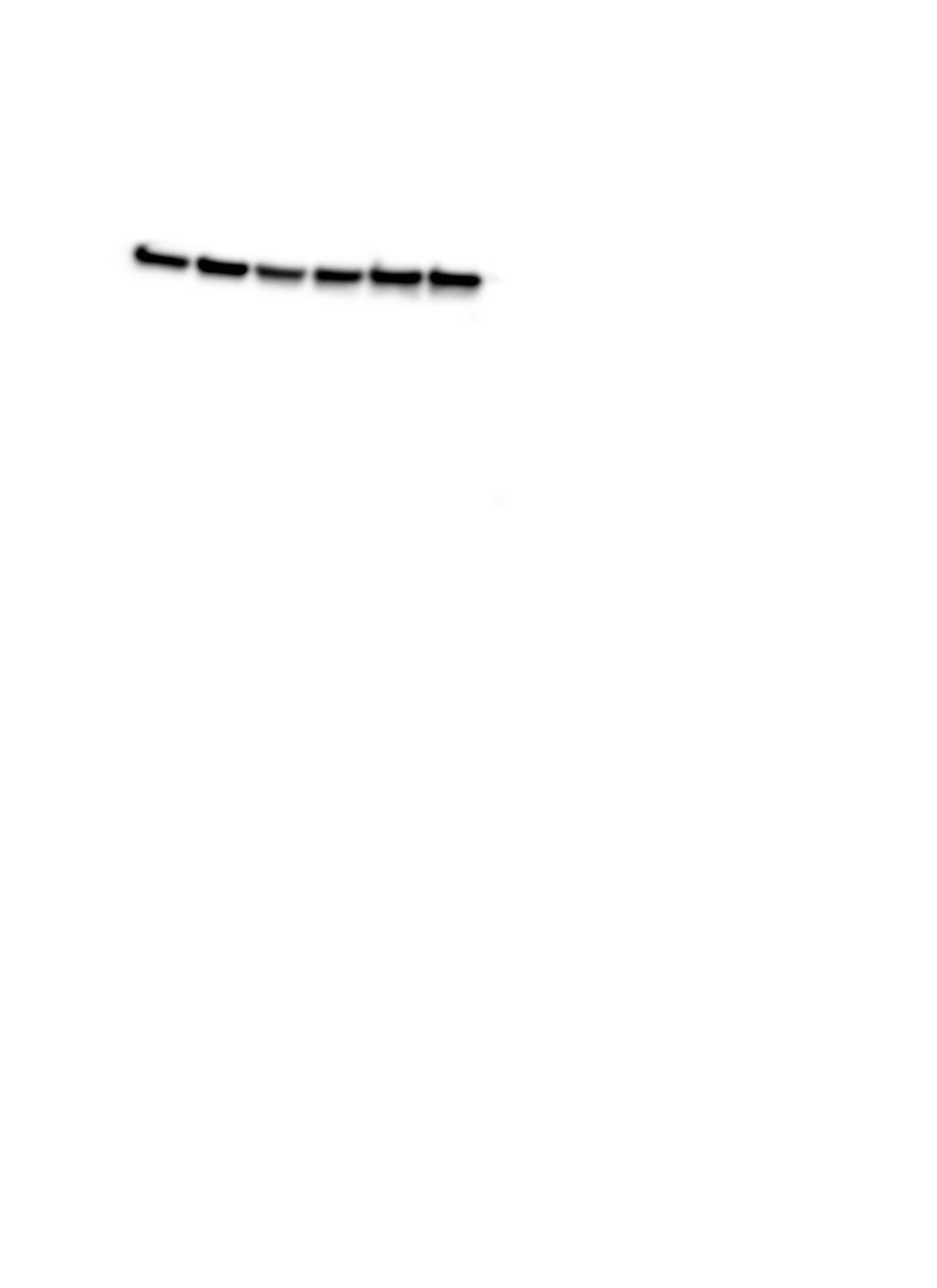

Supplement: Supplementary file 5 — Expanded View Figure Source Data [file 44319_2024_180_MOESM5_ESM.zip › EV Figure Source/EV1/EV1G/WB (PC3) actin.jpg]

# Expanded View Figure E1G

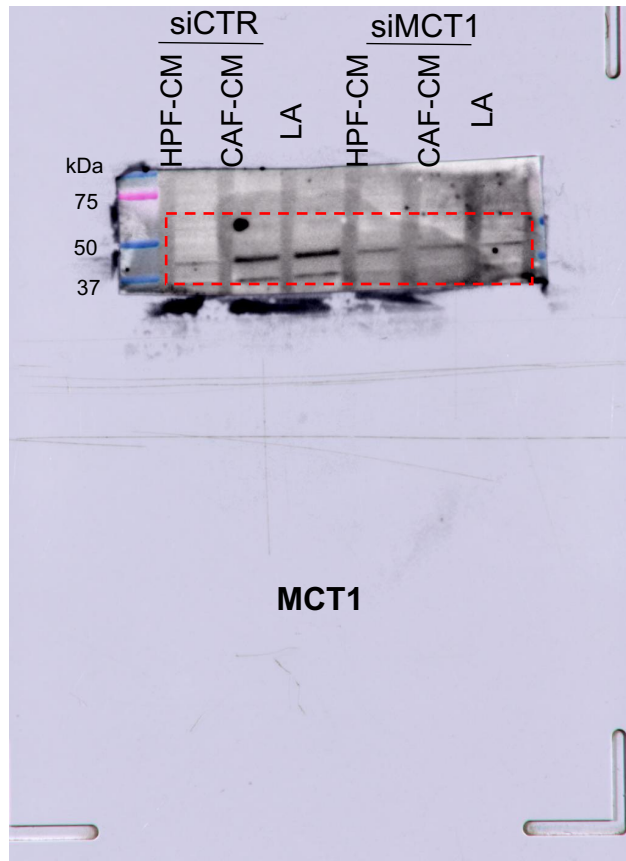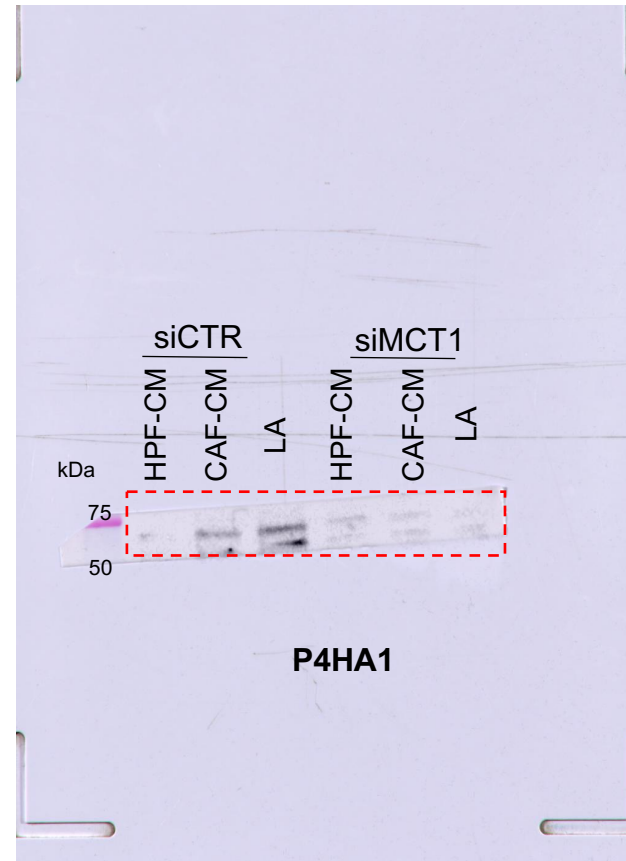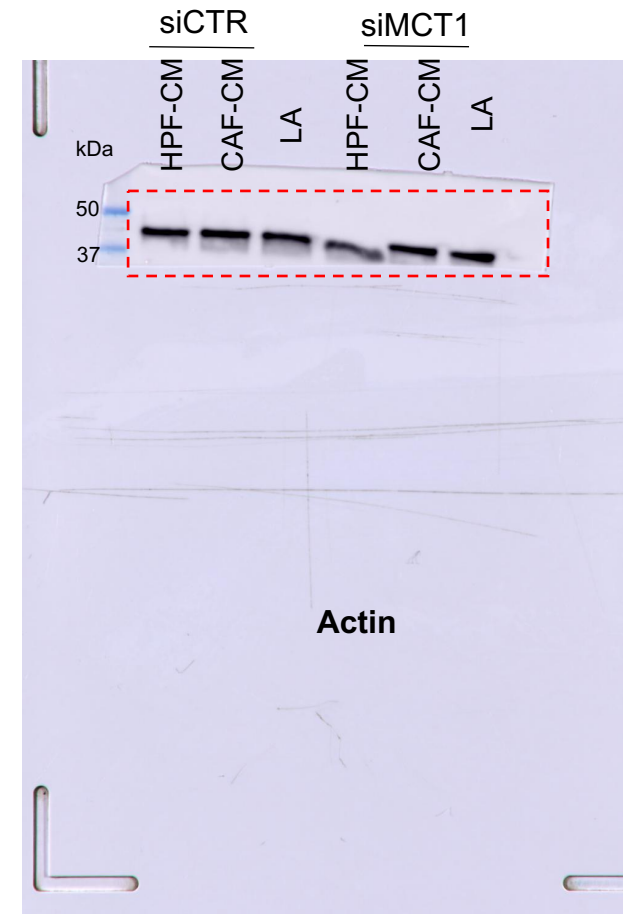

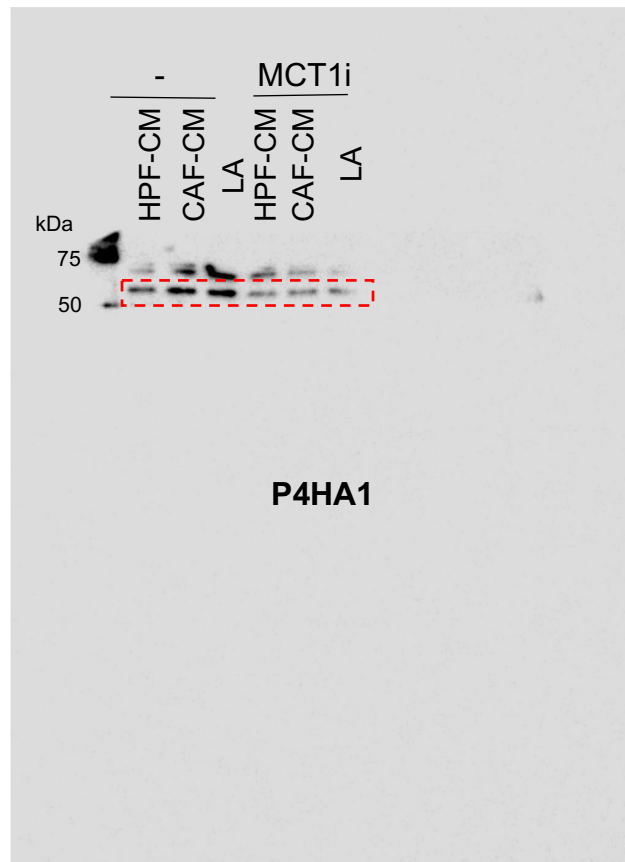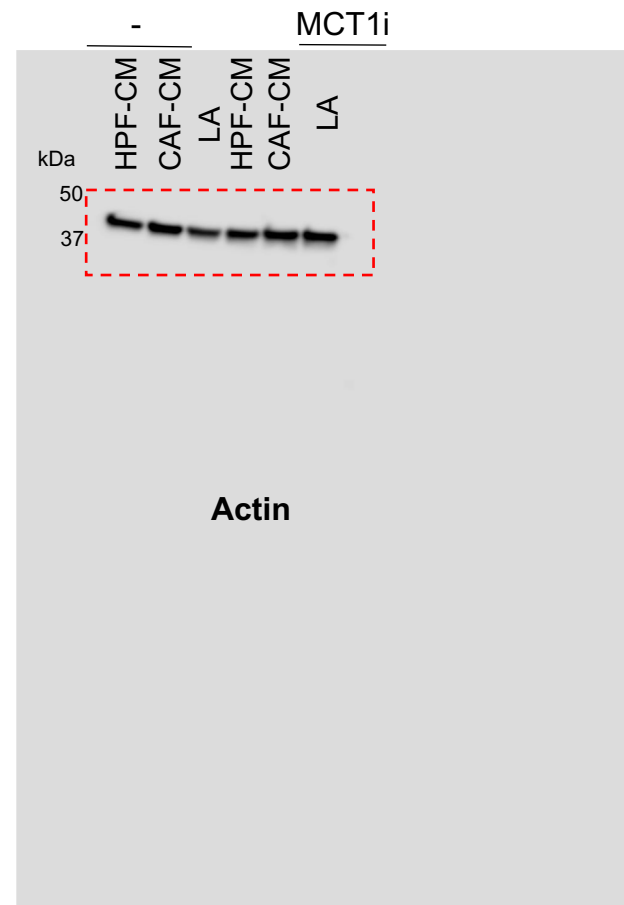

Supplement: Supplementary file 5 — Expanded View Figure Source Data [file 44319_2024_180_MOESM5_ESM.zip › EV Figure Source/EV1/EV1G/EV1G blot.pdf]

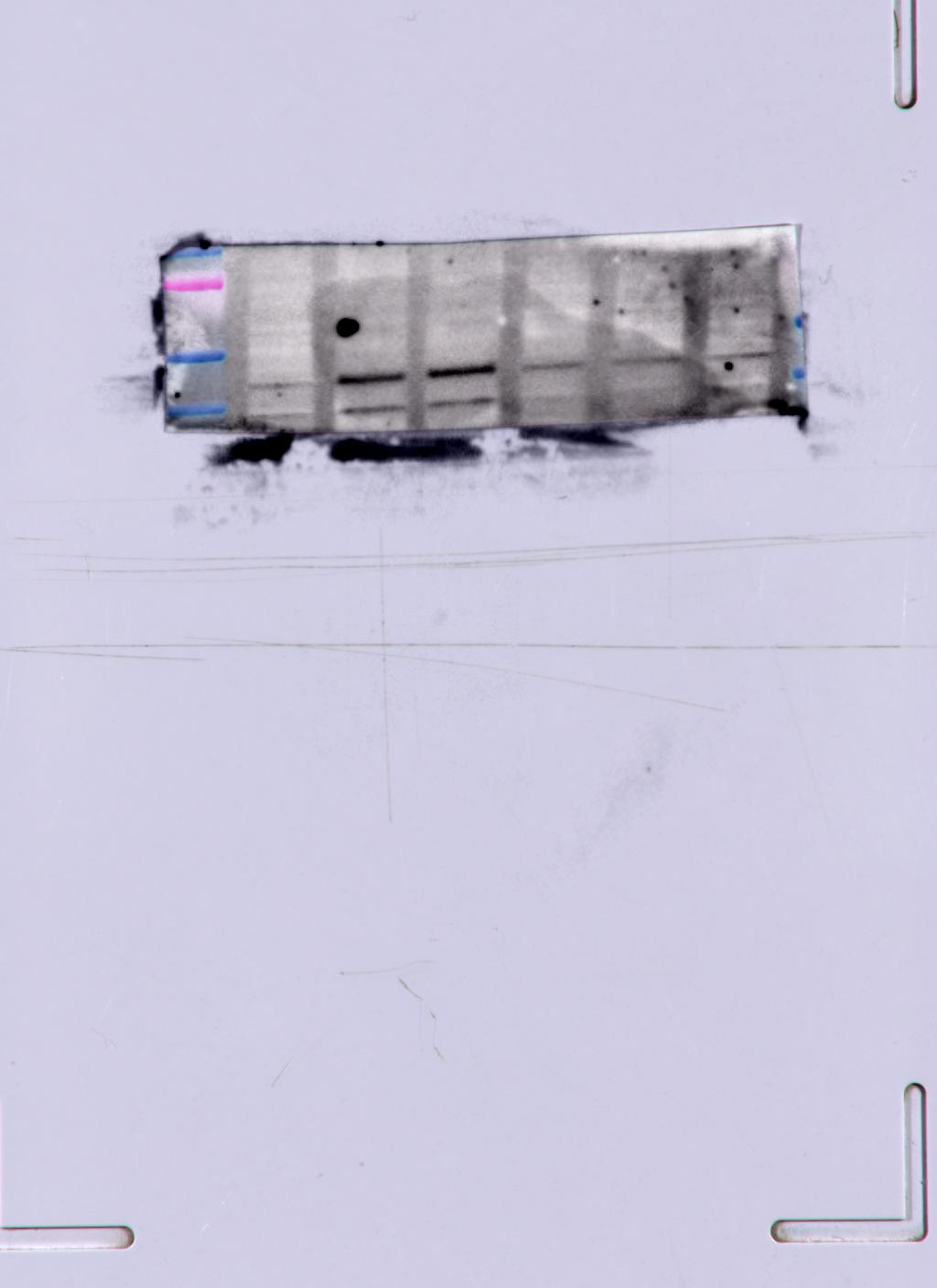

Supplement: Supplementary file 5 — Expanded View Figure Source Data [file 44319_2024_180_MOESM5_ESM.zip › EV Figure Source/EV1/EV1G/WB MCT1_simct1.jpg]

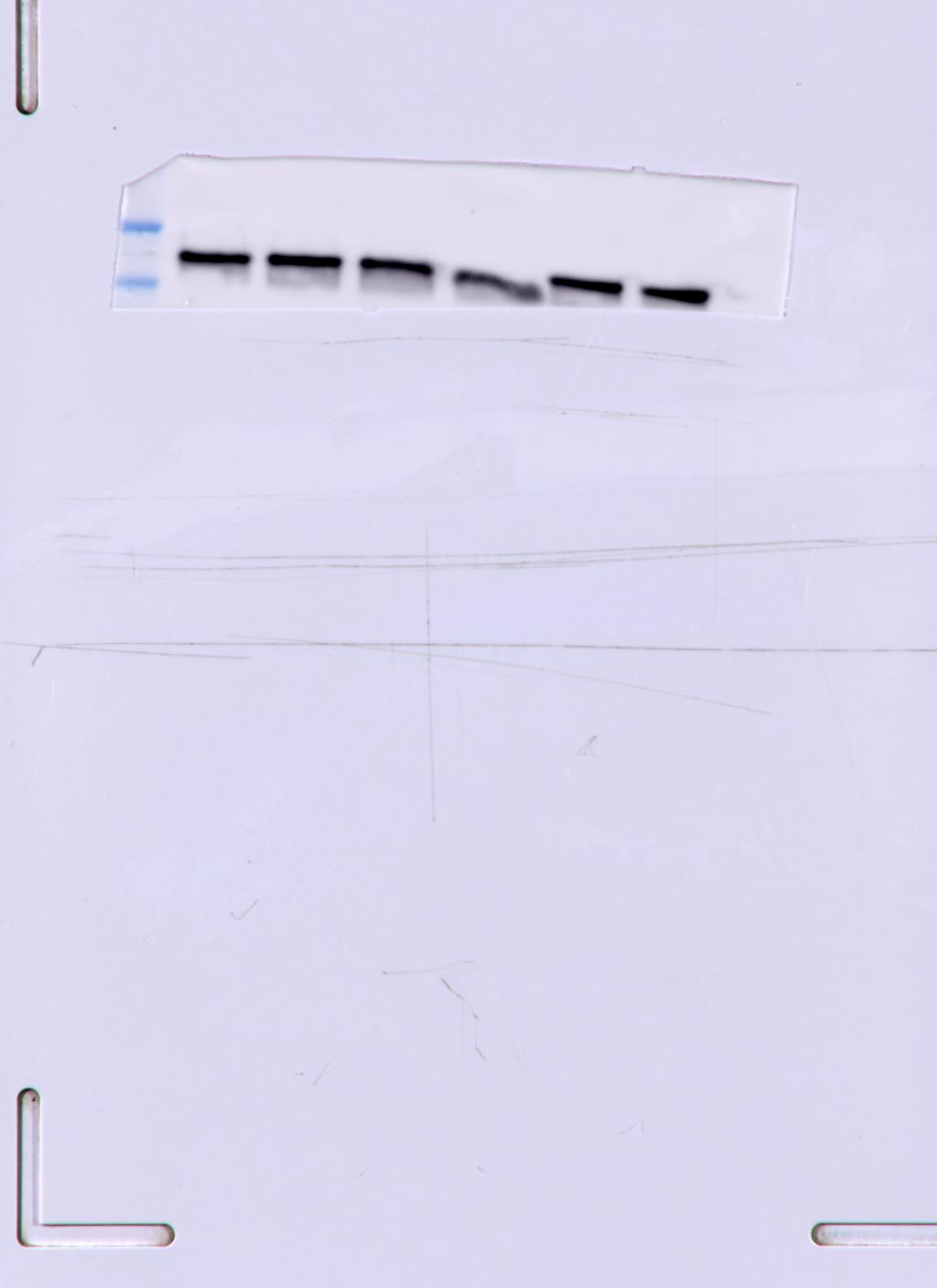

Supplement: Supplementary file 5 — Expanded View Figure Source Data [file 44319_2024_180_MOESM5_ESM.zip › EV Figure Source/EV1/EV1G/WB Actin_simct1.jpg]

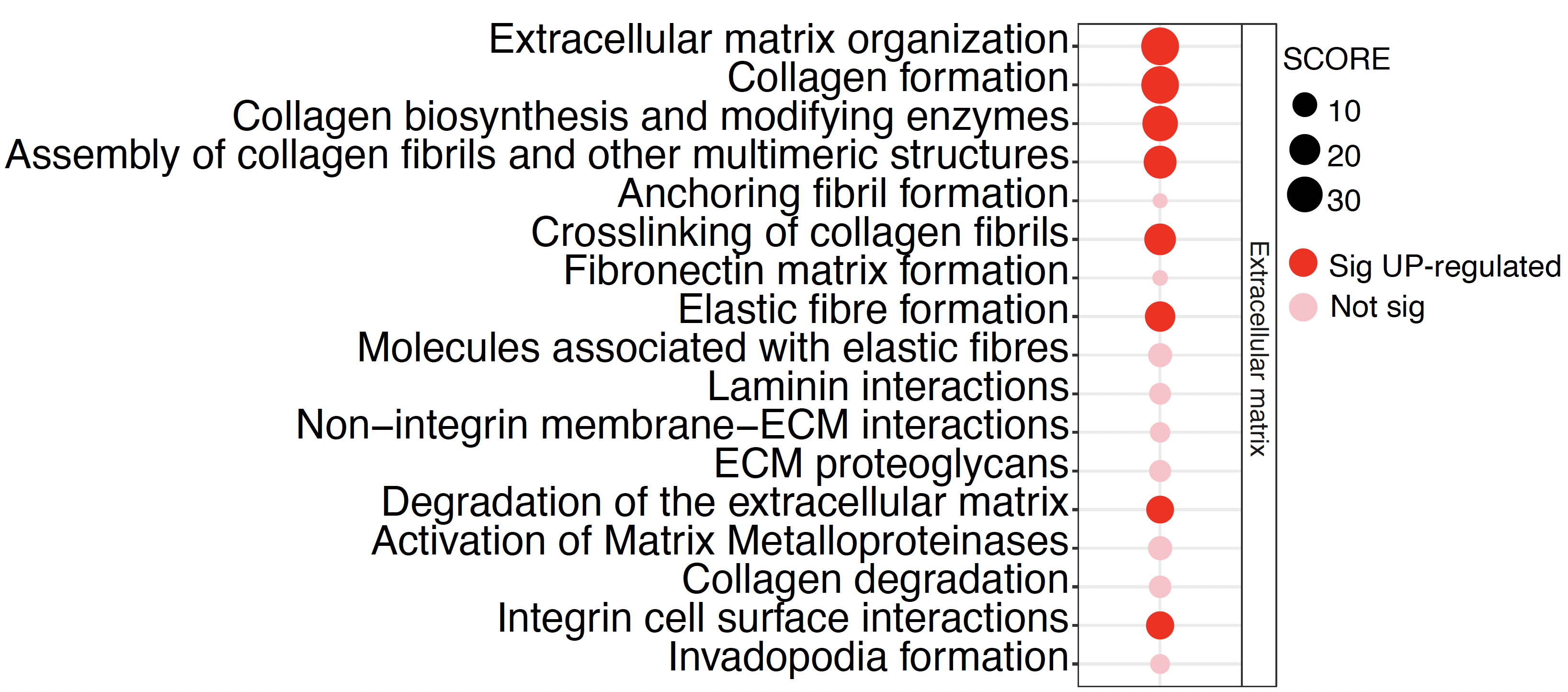

Supplement: Supplementary file 5 — Expanded View Figure Source Data [file 44319_2024_180_MOESM5_ESM.zip › EV Figure Source/EV1/EV1A/Enrich_ECM.png]

**Expanded View Figure 1F**

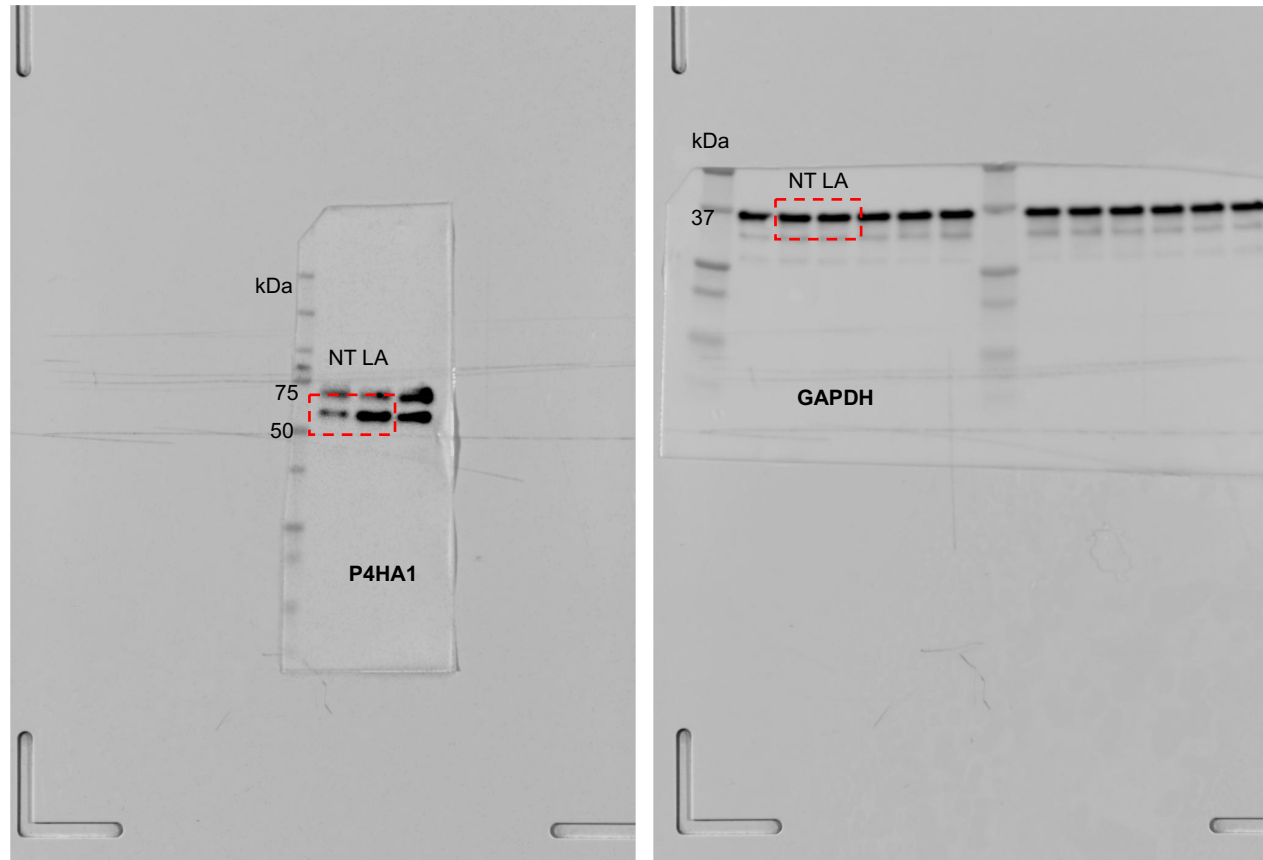

Supplement: Supplementary file 5 — Expanded View Figure Source Data [file 44319_2024_180_MOESM5_ESM.zip › EV Figure Source/EV1/EV1F/WB EV1F.pdf]

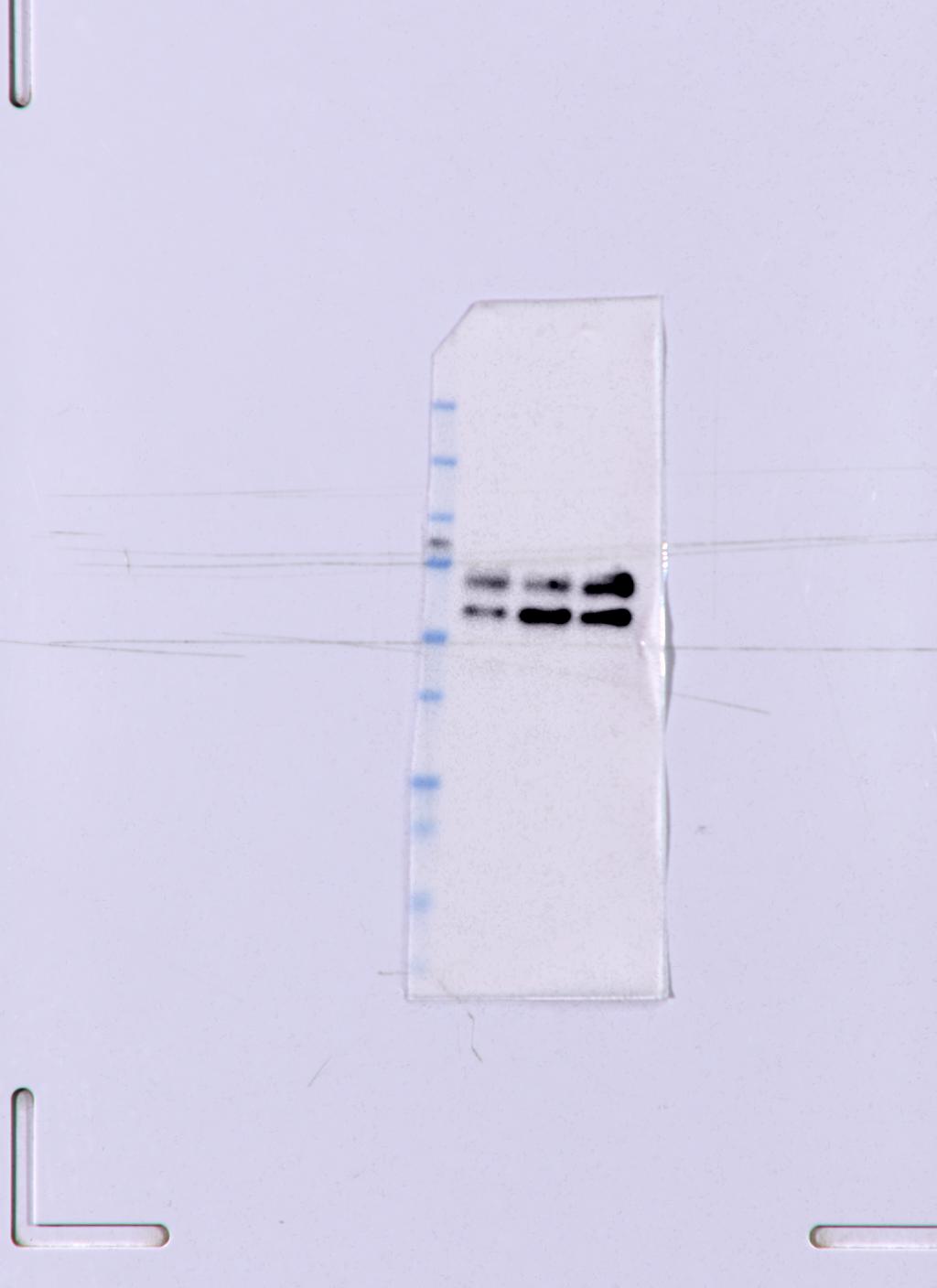

Supplement: Supplementary file 5 — Expanded View Figure Source Data [file 44319_2024_180_MOESM5_ESM.zip › EV Figure Source/EV1/EV1F/WB P4HA1.jpg]

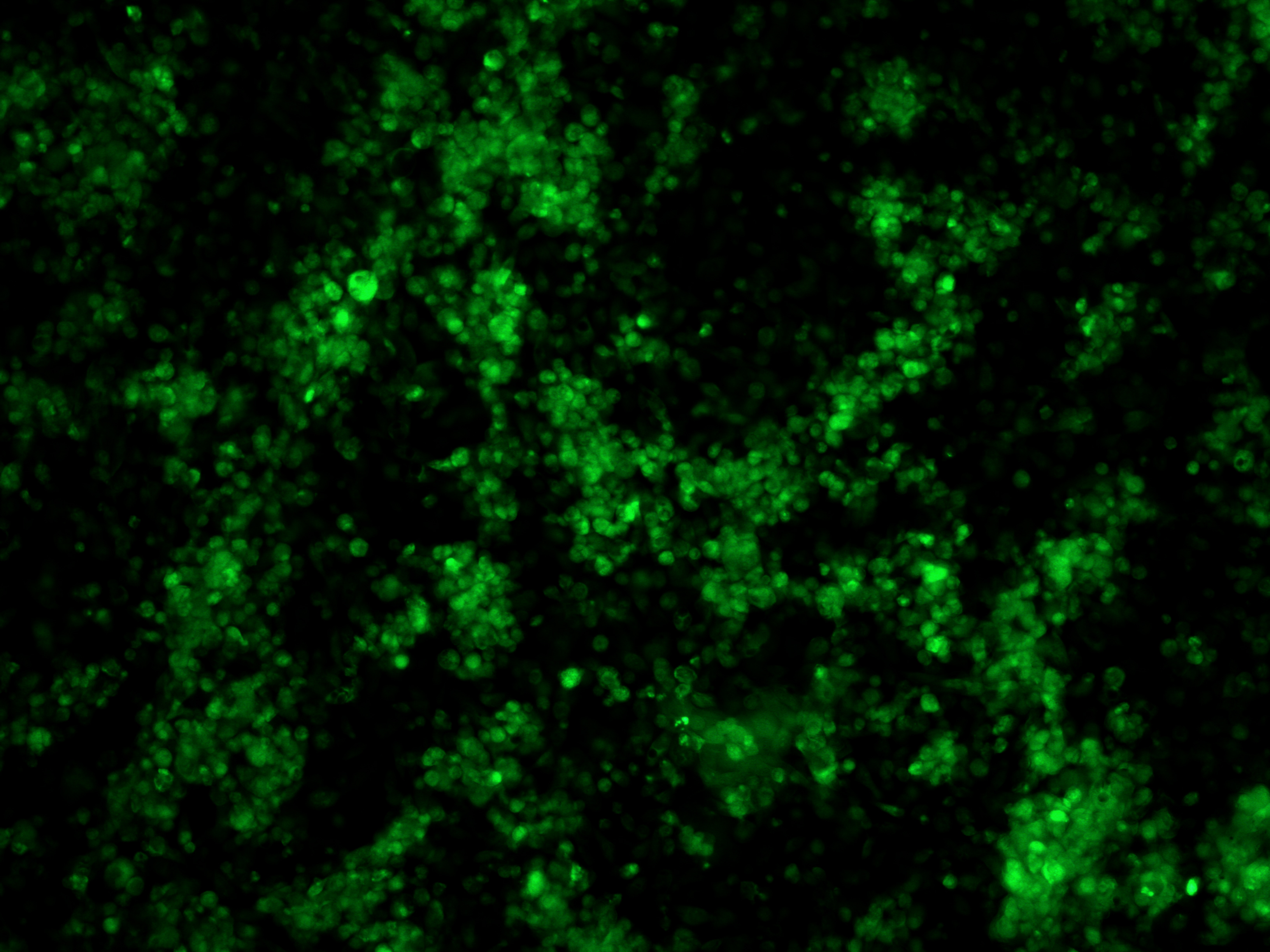

Supplement: Supplementary file 5 — Expanded View Figure Source Data [file 44319_2024_180_MOESM5_ESM.zip › EV Figure Source/EV2/EV2D/extraCol1 siCTR LA.jpg]

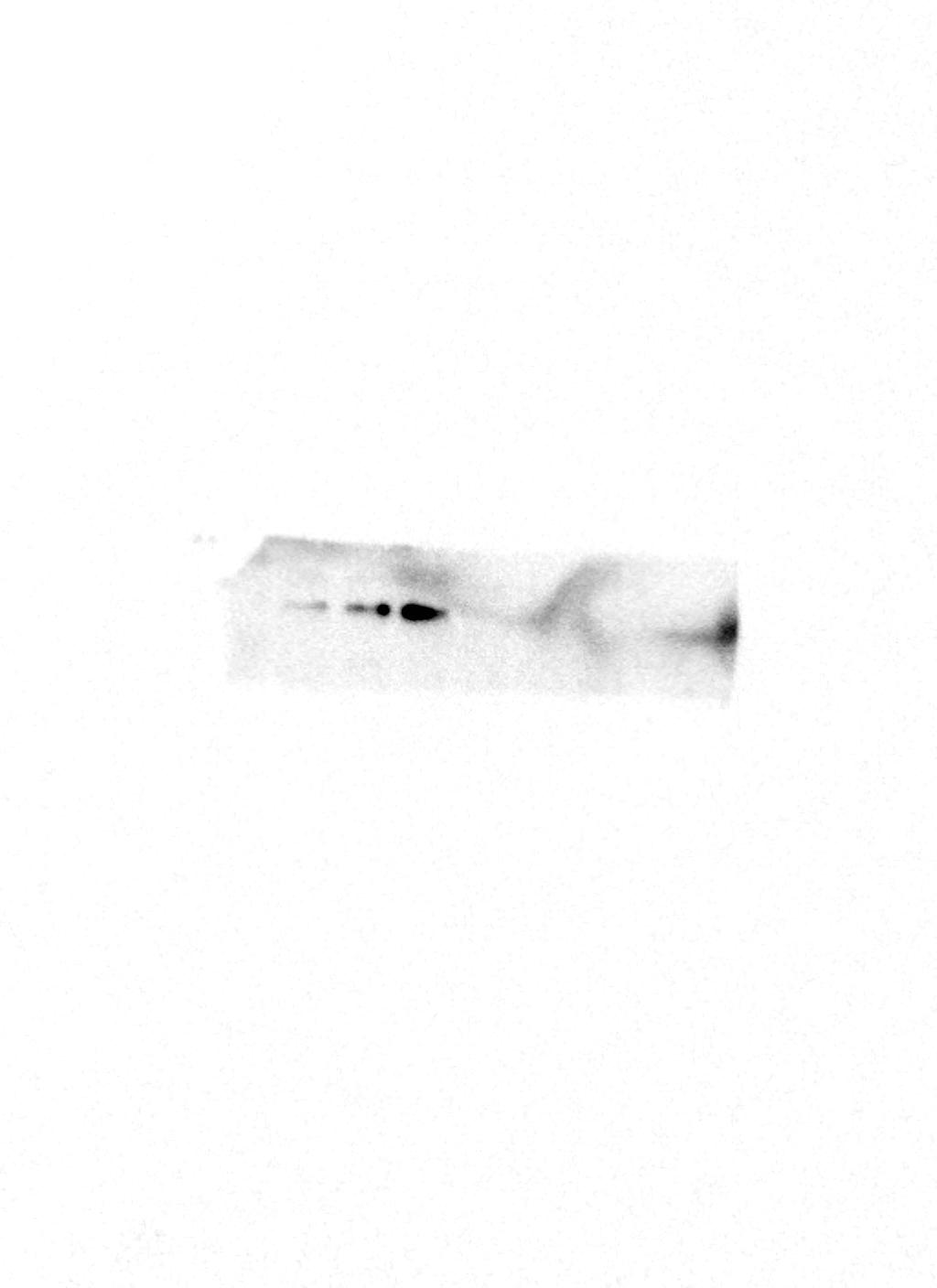

Supplement: Supplementary file 5 — Expanded View Figure Source Data [file 44319_2024_180_MOESM5_ESM.zip › EV Figure Source/EV2/EV2D/WB P4HA1 (PC3).jpg]

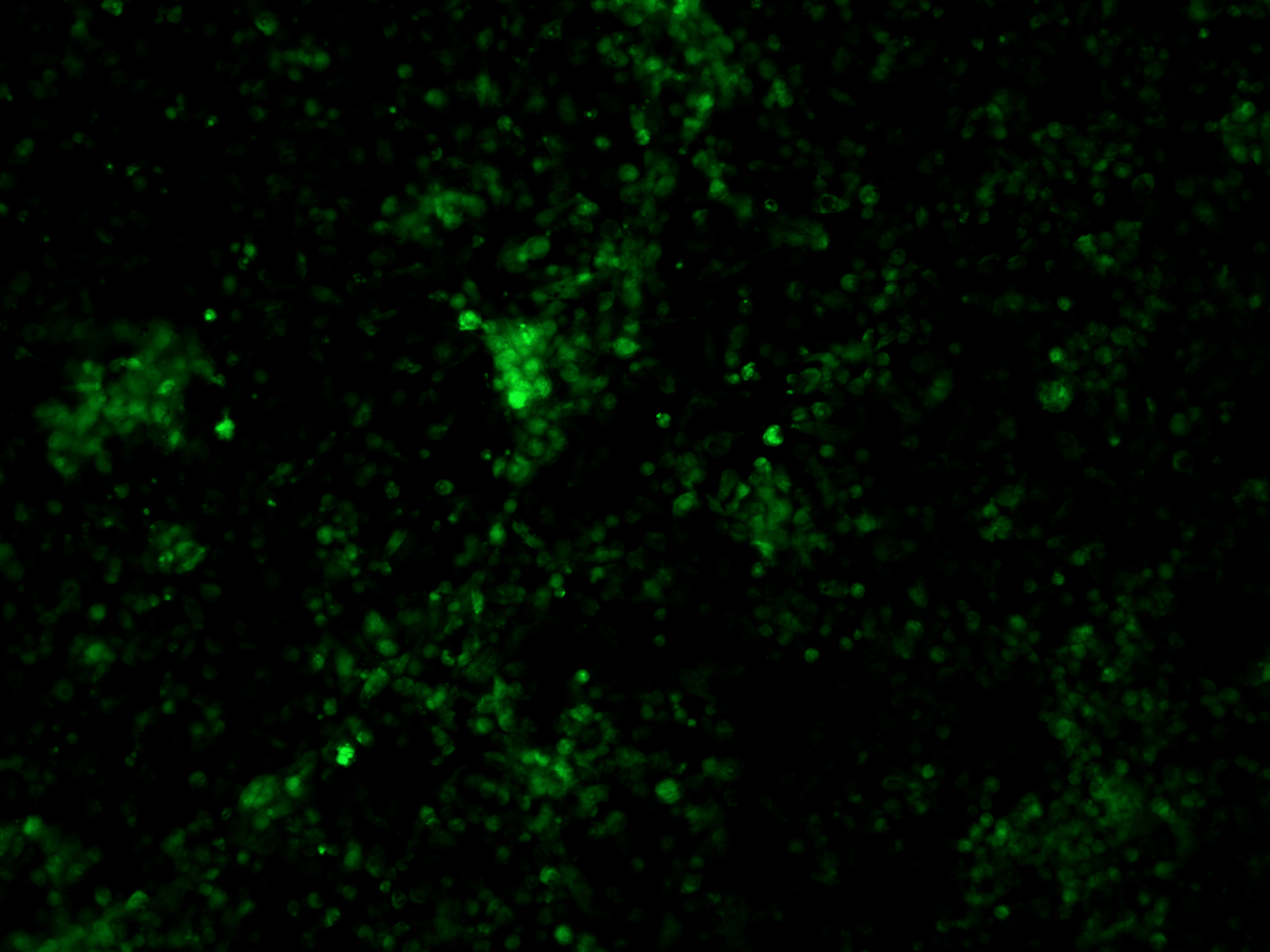

Supplement: Supplementary file 5 — Expanded View Figure Source Data [file 44319_2024_180_MOESM5_ESM.zip › EV Figure Source/EV2/EV2D/extraCol1 siP4HA1 CAF-CM.jpg]

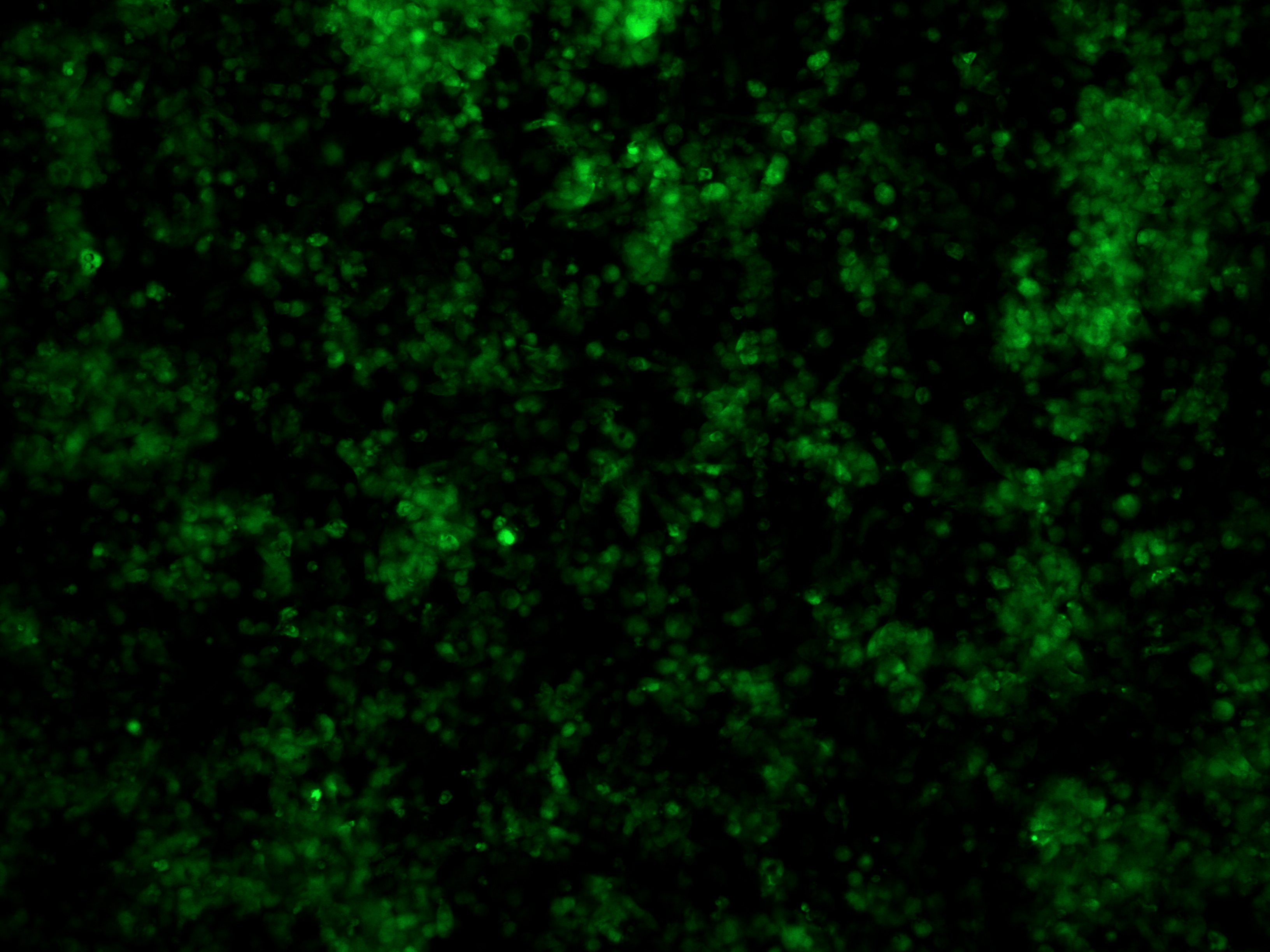

Supplement: Supplementary file 5 — Expanded View Figure Source Data [file 44319_2024_180_MOESM5_ESM.zip › EV Figure Source/EV2/EV2D/extraCol1 siP4HA1 LA.jpg]

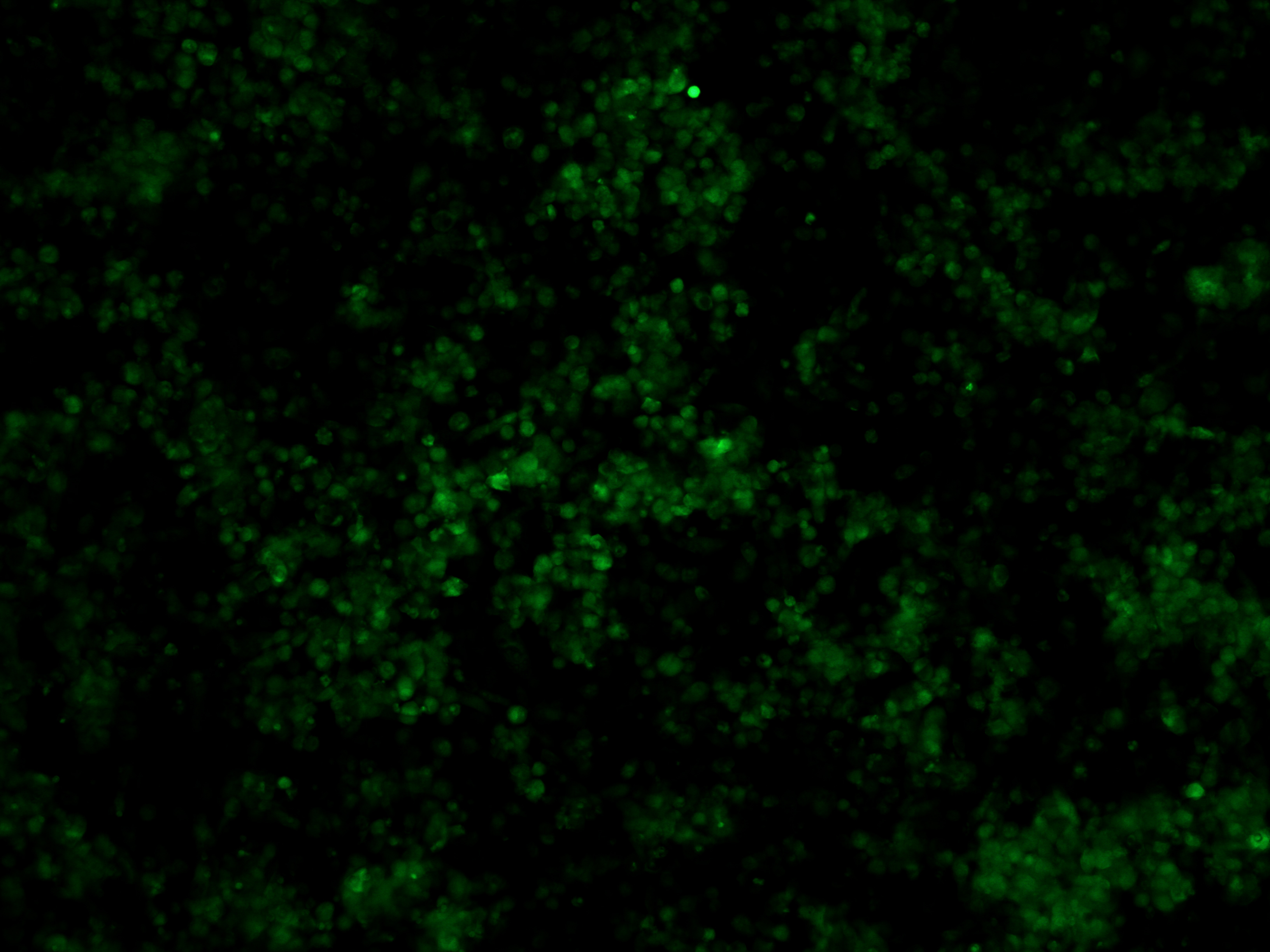

Supplement: Supplementary file 5 — Expanded View Figure Source Data [file 44319_2024_180_MOESM5_ESM.zip › EV Figure Source/EV2/EV2D/extraCol1 siP4HA1 HPF-CM.jpg]

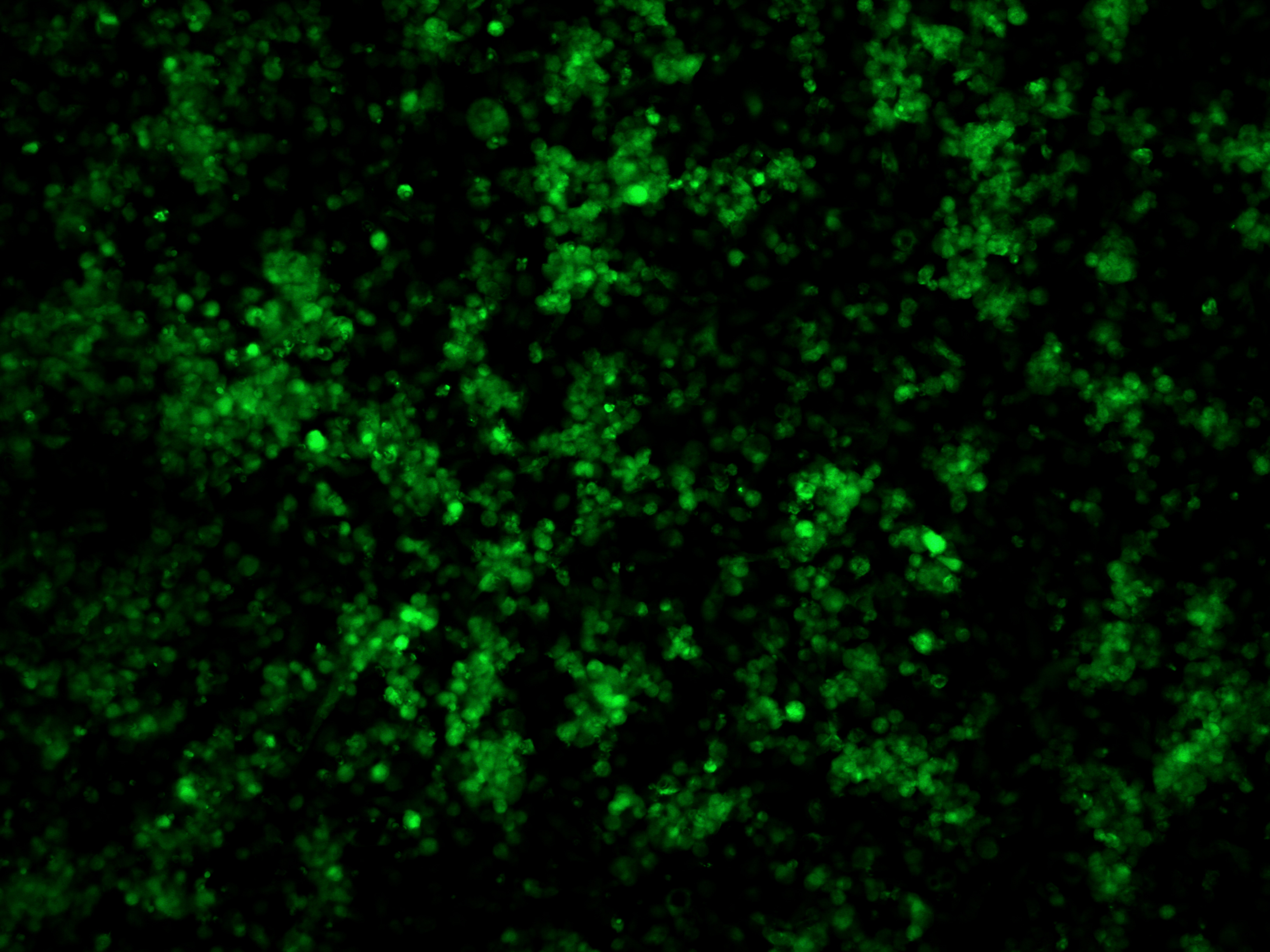

Supplement: Supplementary file 5 — Expanded View Figure Source Data [file 44319_2024_180_MOESM5_ESM.zip › EV Figure Source/EV2/EV2D/extraCol1 siCTR CAF_CM.jpg]

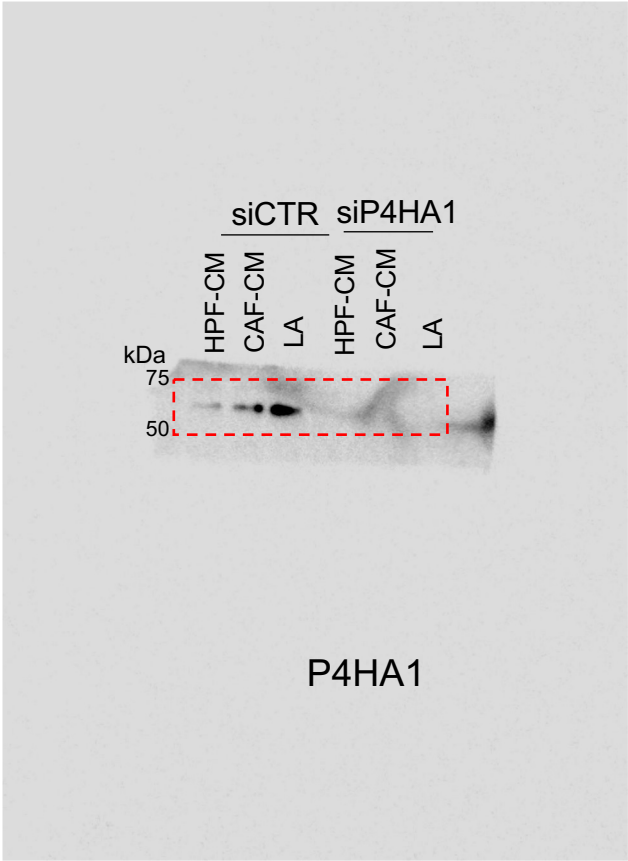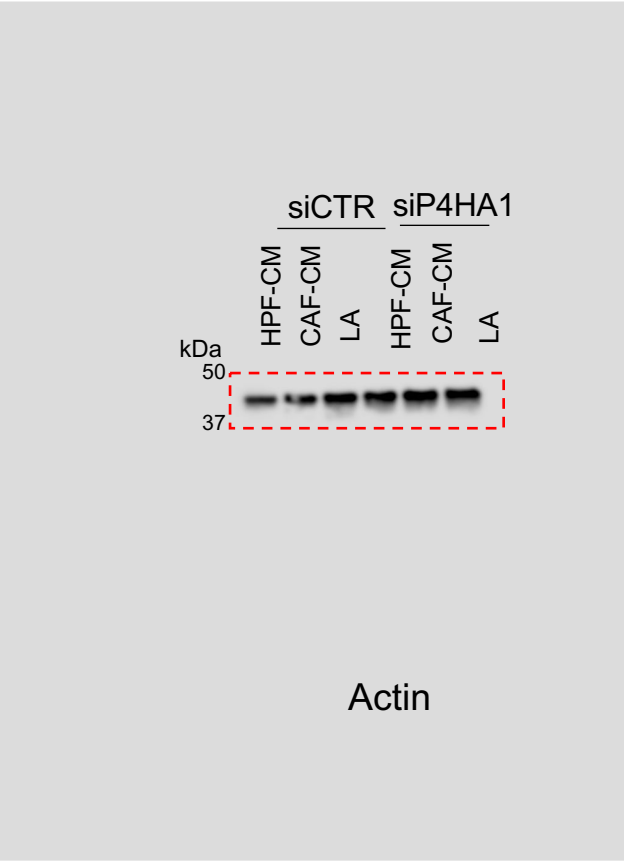

Supplement: Supplementary file 5 — Expanded View Figure Source Data [file 44319_2024_180_MOESM5_ESM.zip › EV Figure Source/EV2/EV2D/EV2D blot.pdf]

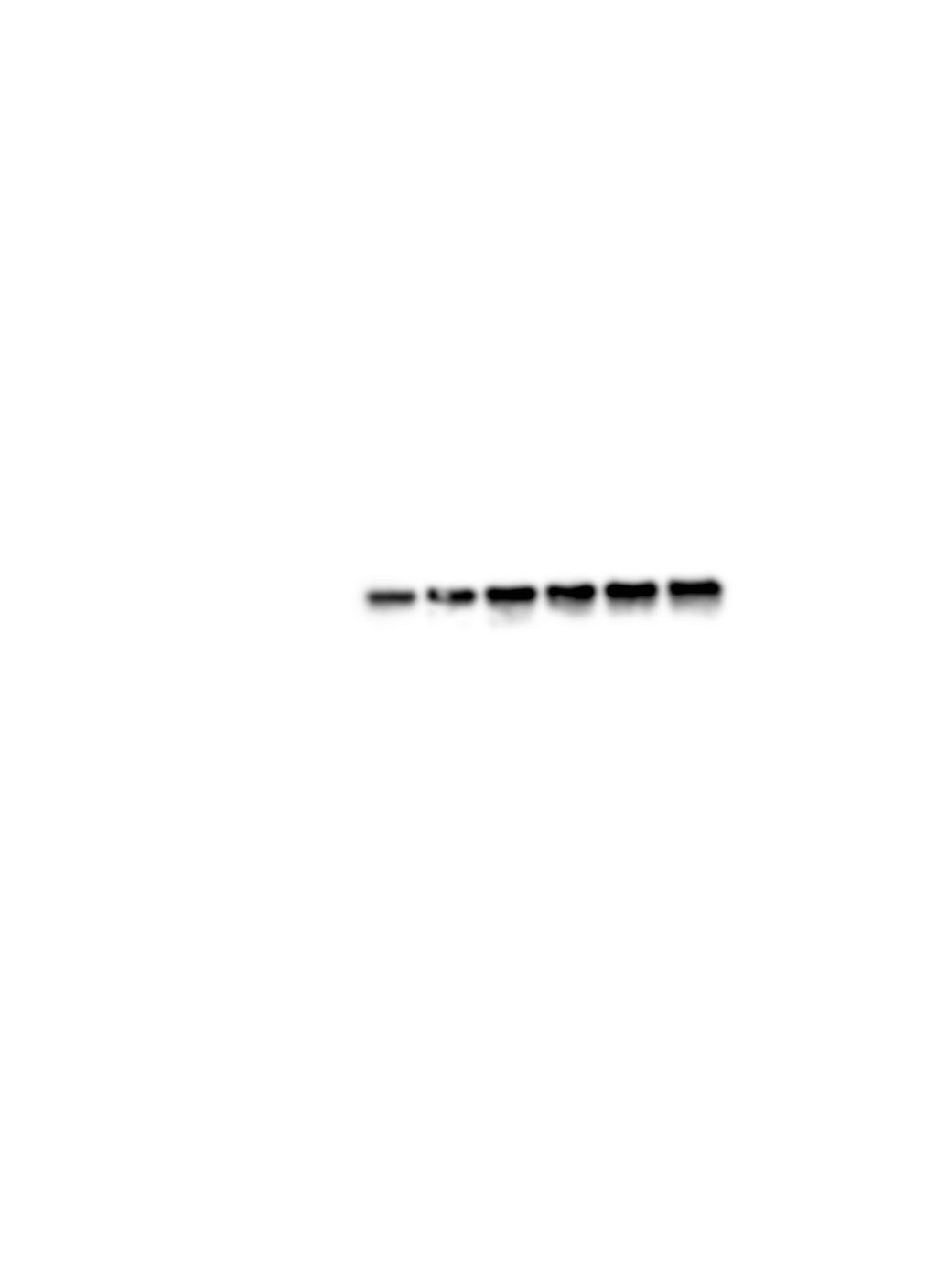

Supplement: Supplementary file 5 — Expanded View Figure Source Data [file 44319_2024_180_MOESM5_ESM.zip › EV Figure Source/EV2/EV2D/WB Actin (PC3).jpg]

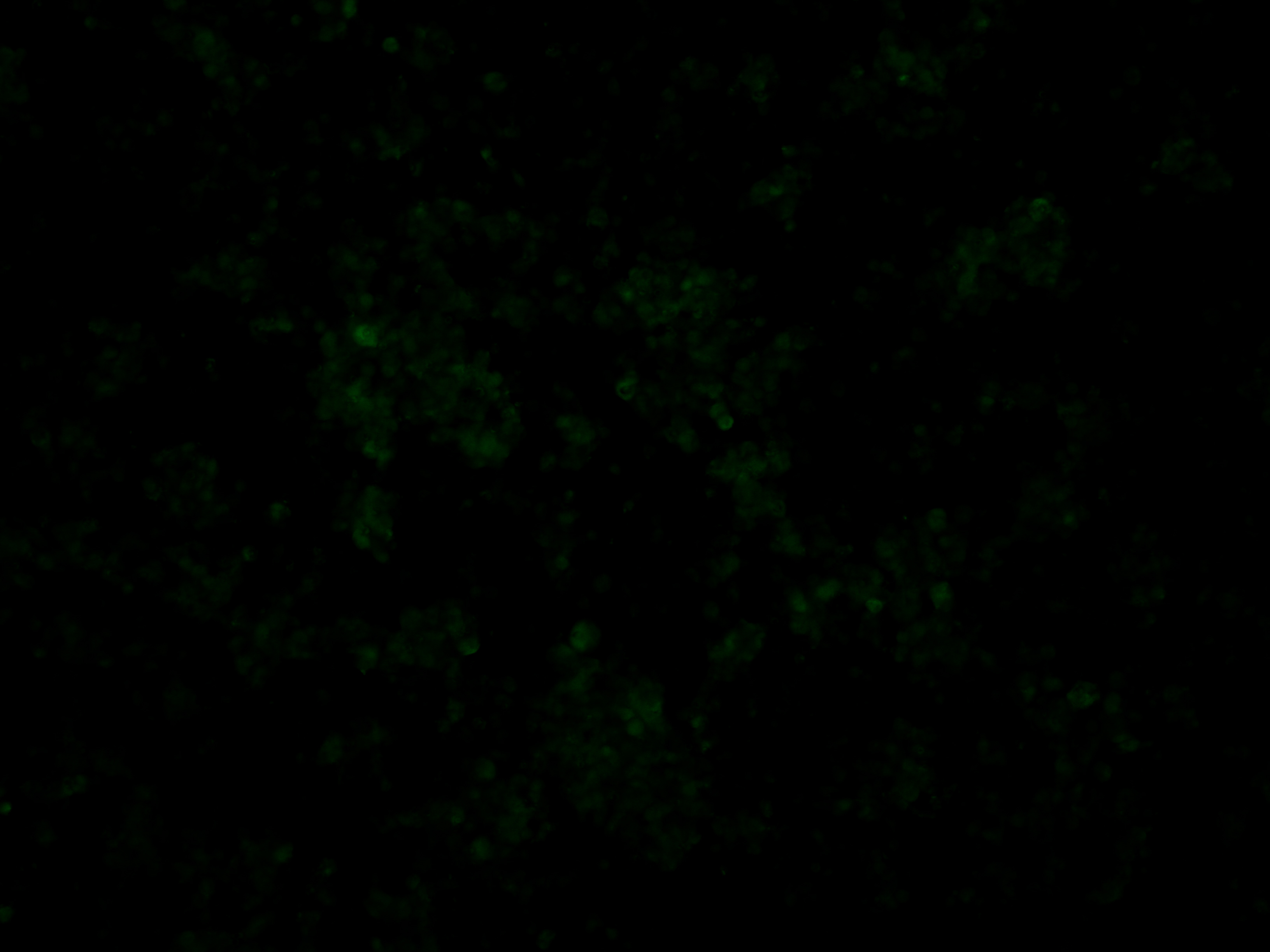

Supplement: Supplementary file 5 — Expanded View Figure Source Data [file 44319_2024_180_MOESM5_ESM.zip › EV Figure Source/EV2/EV2D/extraCol1 siCTR HPF-CM.jpg]

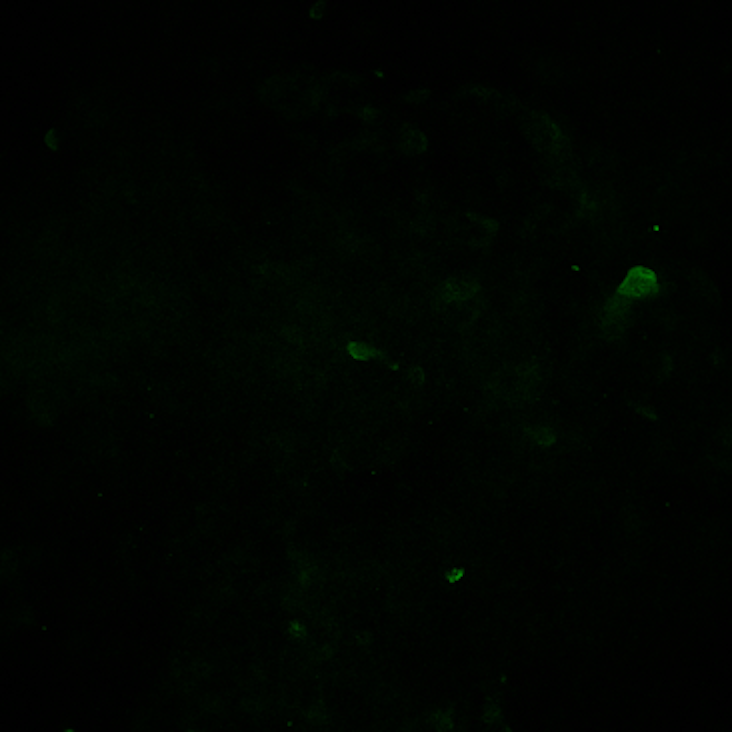

Supplement: Supplementary file 5 — Expanded View Figure Source Data [file 44319_2024_180_MOESM5_ESM.zip › EV Figure Source/EV2/EV2C/extraCol1 siCTR HPF_CM.tif]

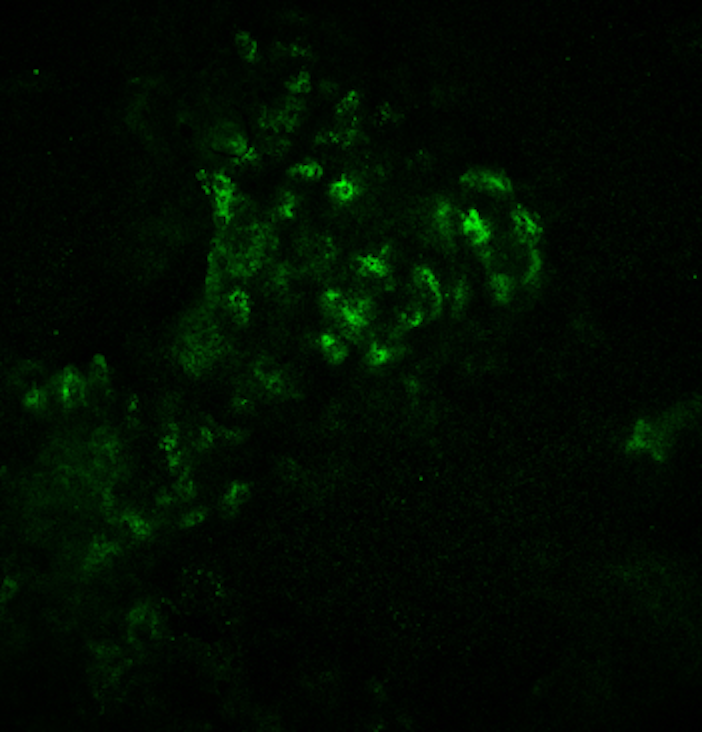

Supplement: Supplementary file 5 — Expanded View Figure Source Data [file 44319_2024_180_MOESM5_ESM.zip › EV Figure Source/EV2/EV2C/extraCol1 siCTR LA.tif]

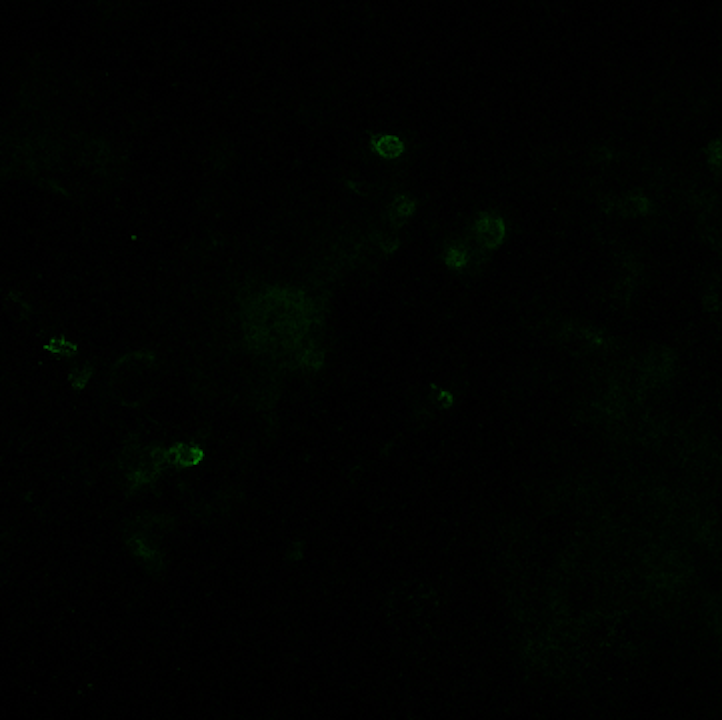

Supplement: Supplementary file 5 — Expanded View Figure Source Data [file 44319_2024_180_MOESM5_ESM.zip › EV Figure Source/EV2/EV2C/extraCol1 siP4HA1 HPF_CM.tif]

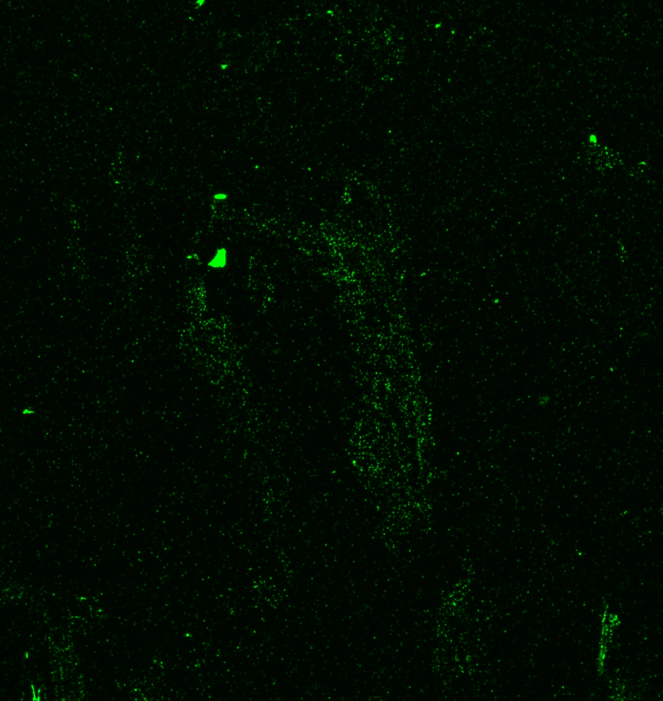

Supplement: Supplementary file 5 — Expanded View Figure Source Data [file 44319_2024_180_MOESM5_ESM.zip › EV Figure Source/EV2/EV2C/extraCol1 siP4HA1 CAF_CM.tif]

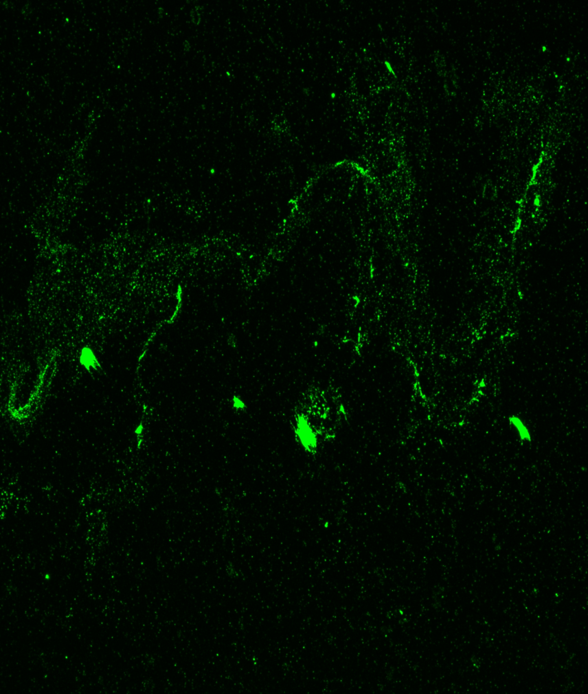

Supplement: Supplementary file 5 — Expanded View Figure Source Data [file 44319_2024_180_MOESM5_ESM.zip › EV Figure Source/EV2/EV2C/extraCol1 siCTR CAF_CM.tif]

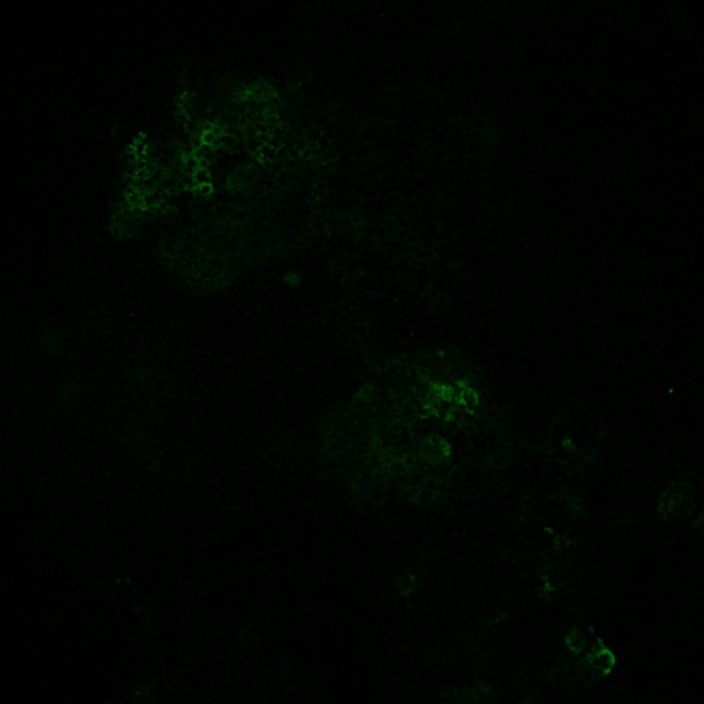

Supplement: Supplementary file 5 — Expanded View Figure Source Data [file 44319_2024_180_MOESM5_ESM.zip › EV Figure Source/EV2/EV2C/extraCol1 siP4HA1 LA.tif]

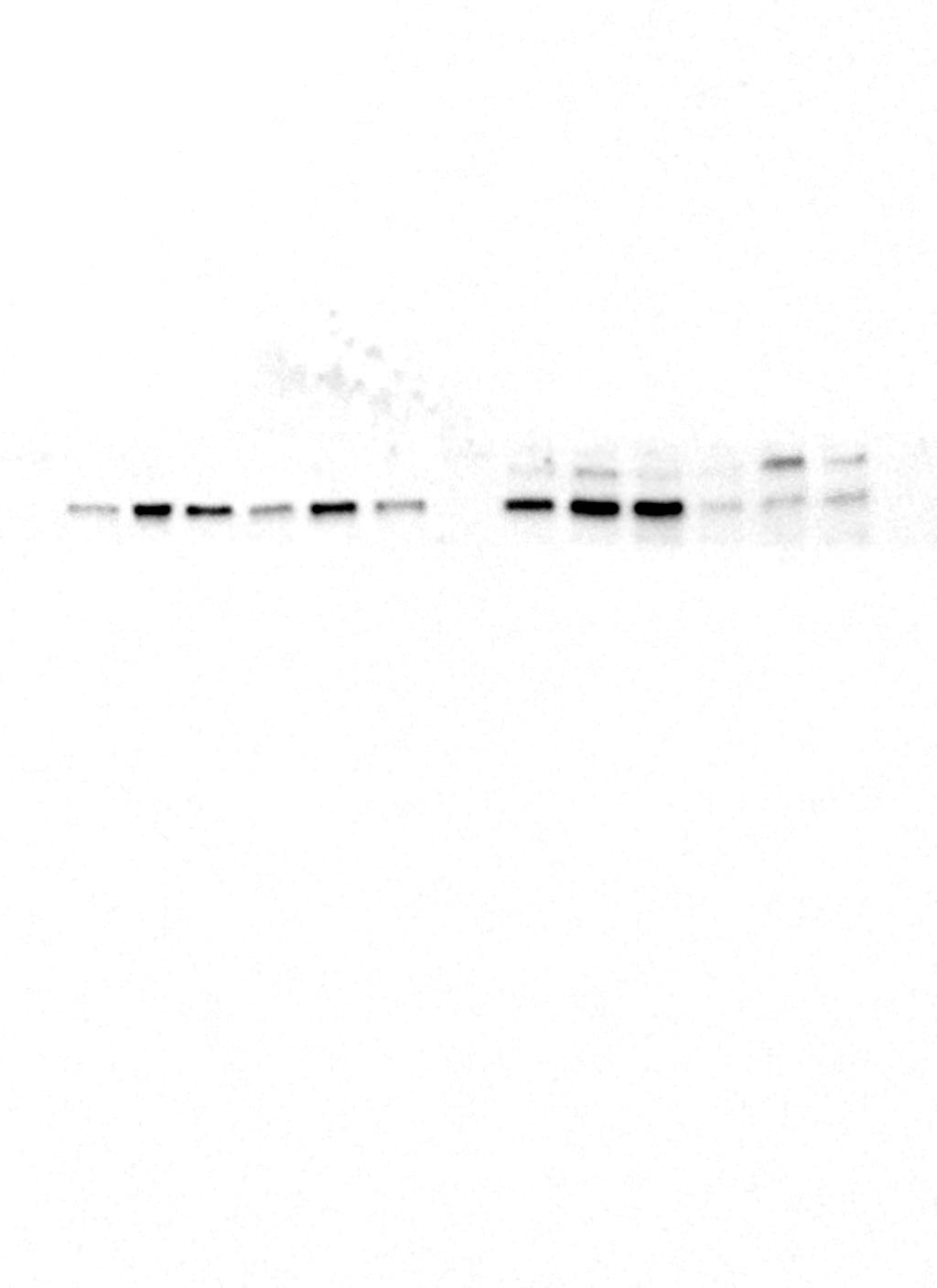

Supplement: Supplementary file 5 — Expanded View Figure Source Data [file 44319_2024_180_MOESM5_ESM.zip › EV Figure Source/EV2/EV2A/WB P4HA1 (DU145).jpg]

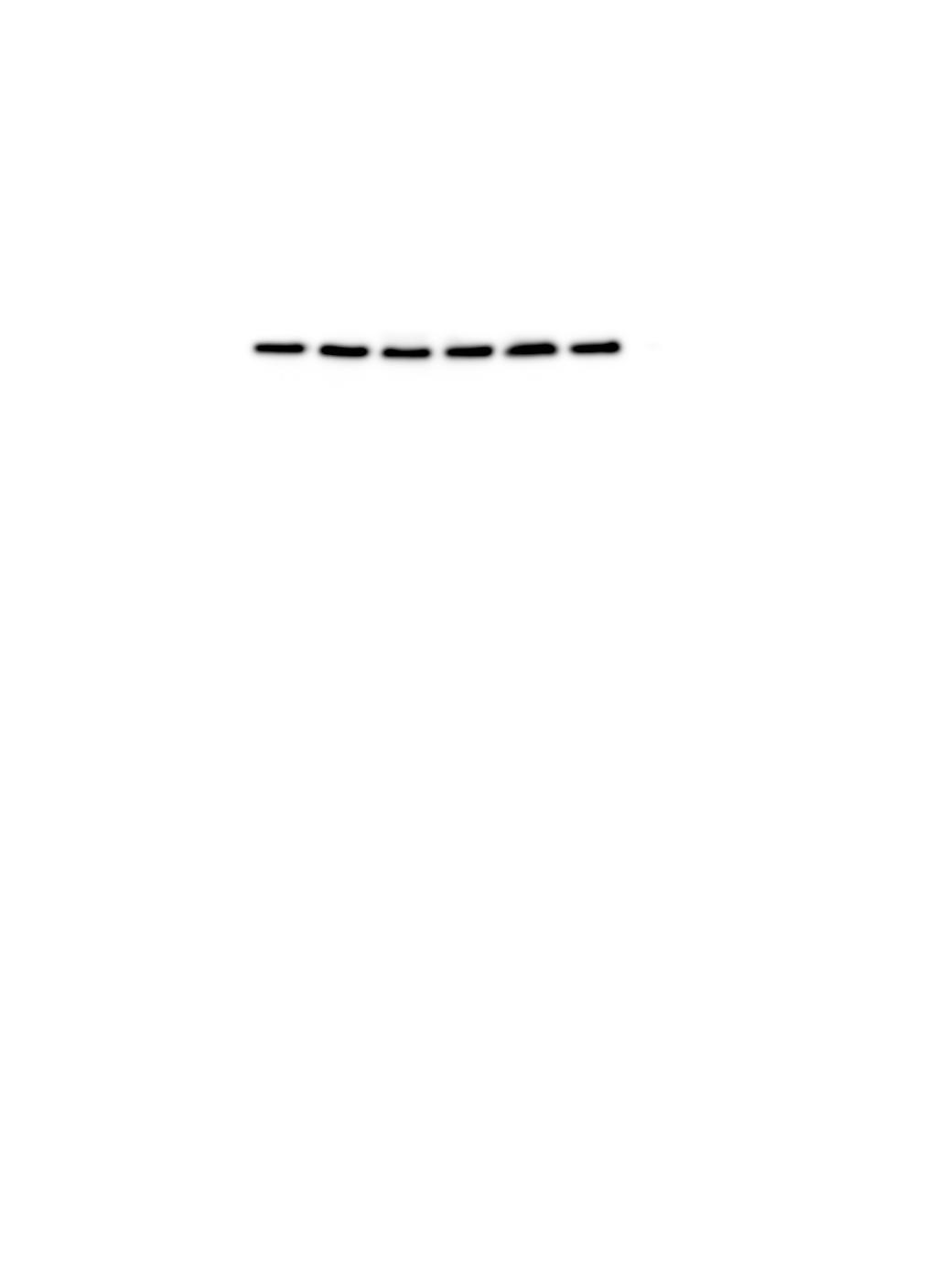

Supplement: Supplementary file 5 — Expanded View Figure Source Data [file 44319_2024_180_MOESM5_ESM.zip › EV Figure Source/EV2/EV2A/WB Actin (DU145).jpg]

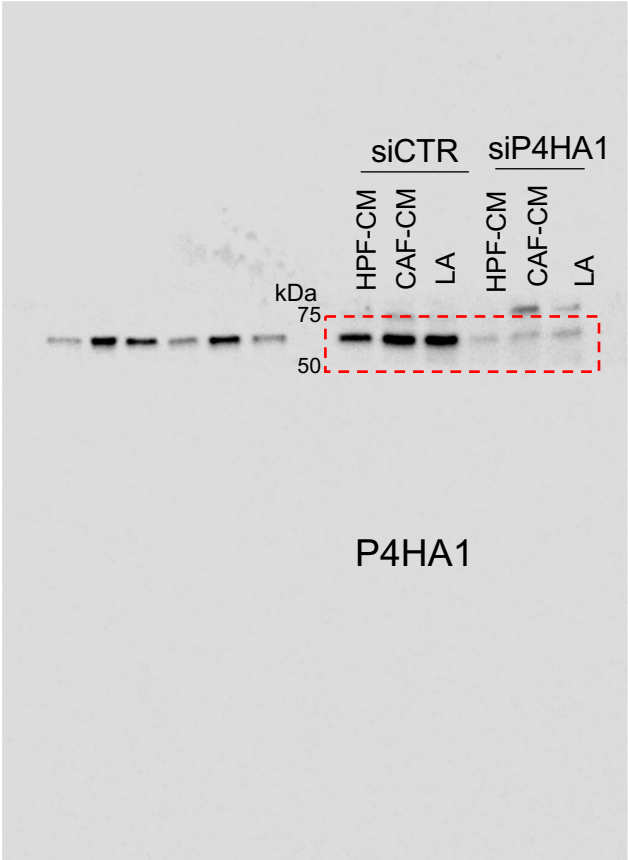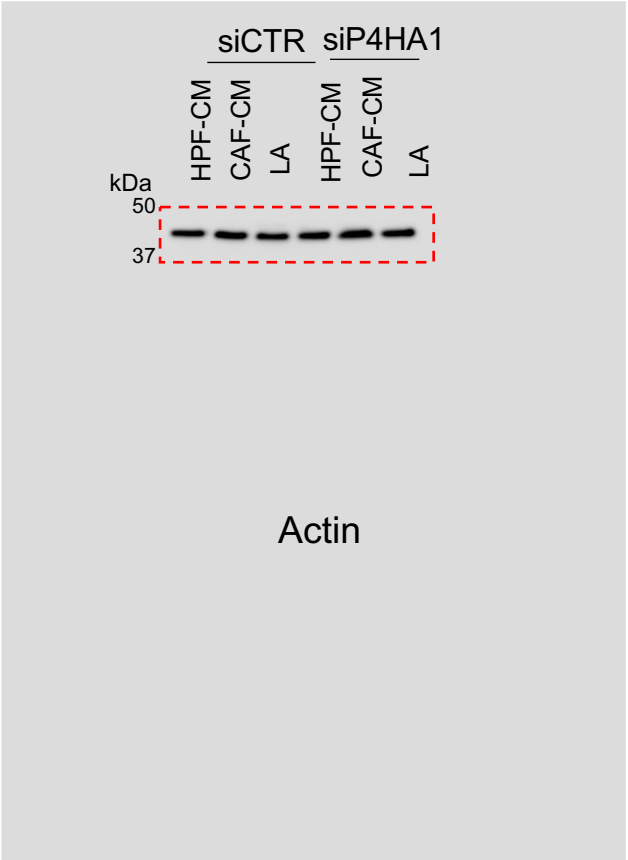

Supplement: Supplementary file 5 — Expanded View Figure Source Data [file 44319_2024_180_MOESM5_ESM.zip › EV Figure Source/EV2/EV2A/EV2A blot.pdf]

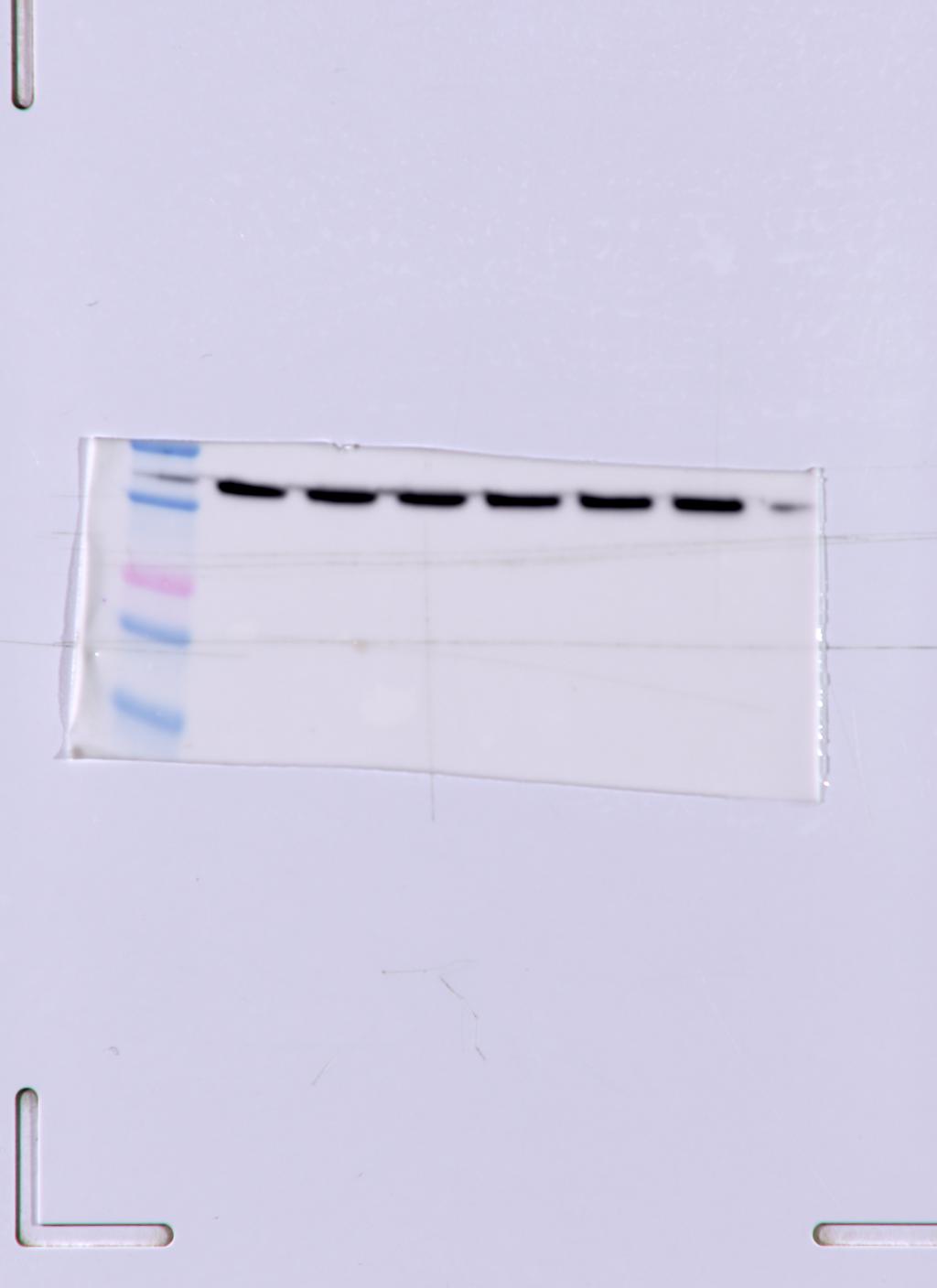

Supplement: Supplementary file 5 — Expanded View Figure Source Data [file 44319_2024_180_MOESM5_ESM.zip › EV Figure Source/EV5/EV5C/WB Actin siCol1.jpg]

## Extended Figure EV5C

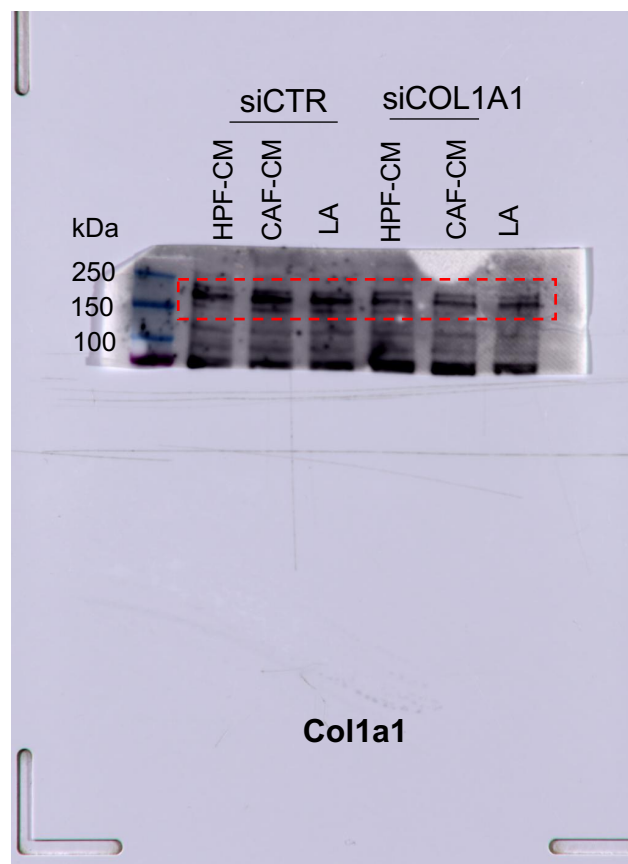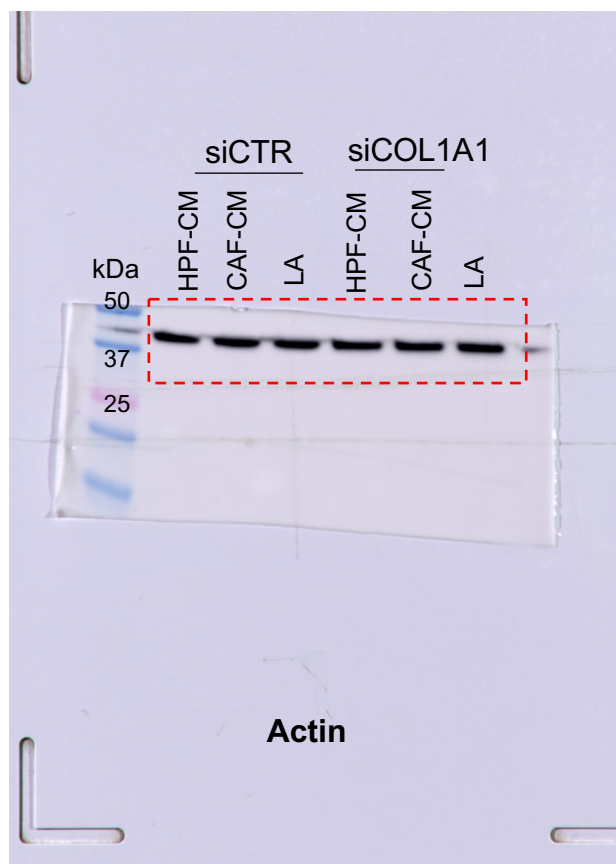

Supplement: Supplementary file 5 — Expanded View Figure Source Data [file 44319_2024_180_MOESM5_ESM.zip › EV Figure Source/EV5/EV5C/EV5C blot.pdf]

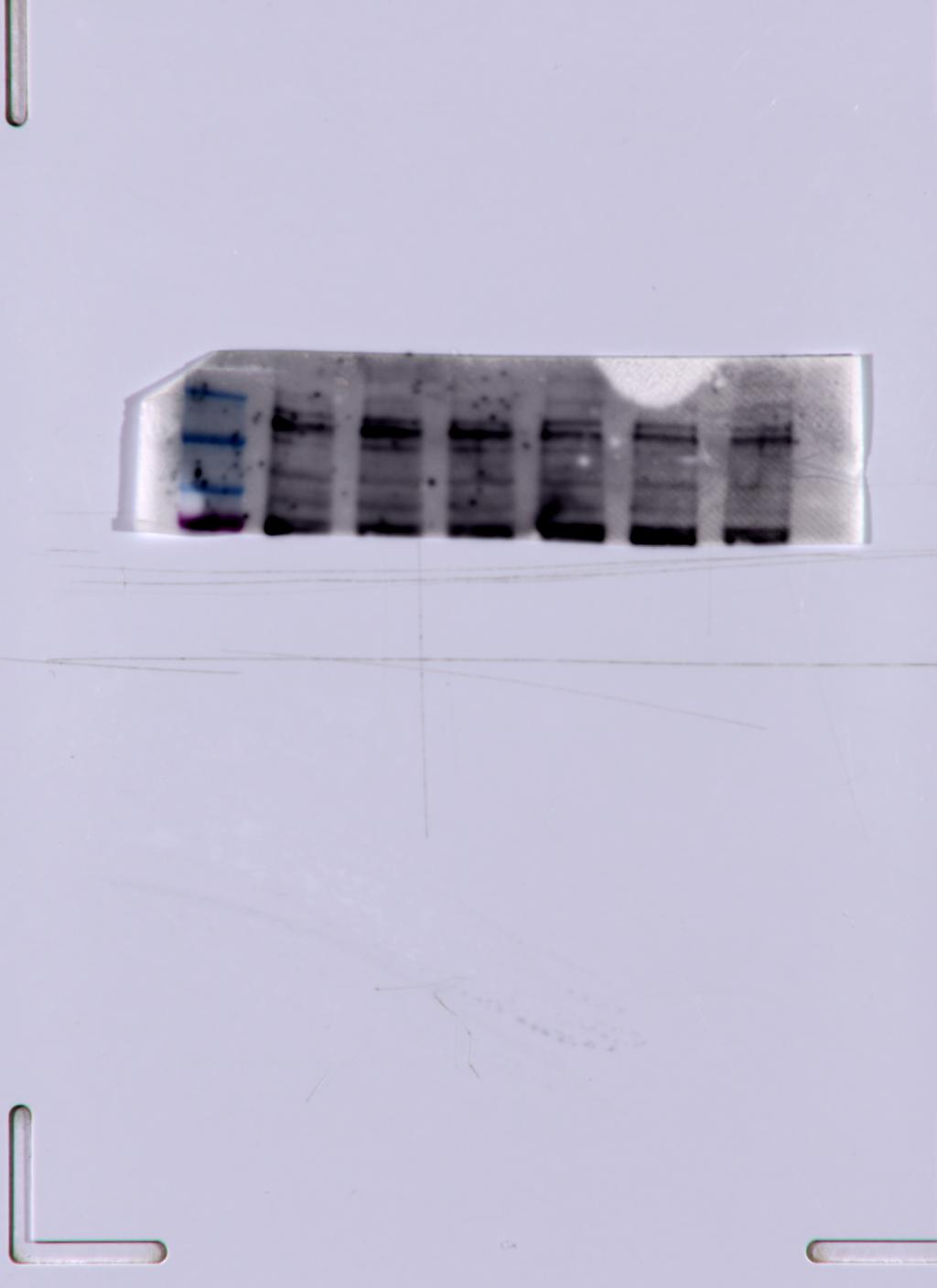

Supplement: Supplementary file 5 — Expanded View Figure Source Data [file 44319_2024_180_MOESM5_ESM.zip › EV Figure Source/EV5/EV5C/WB Col1 siCol1.jpg]

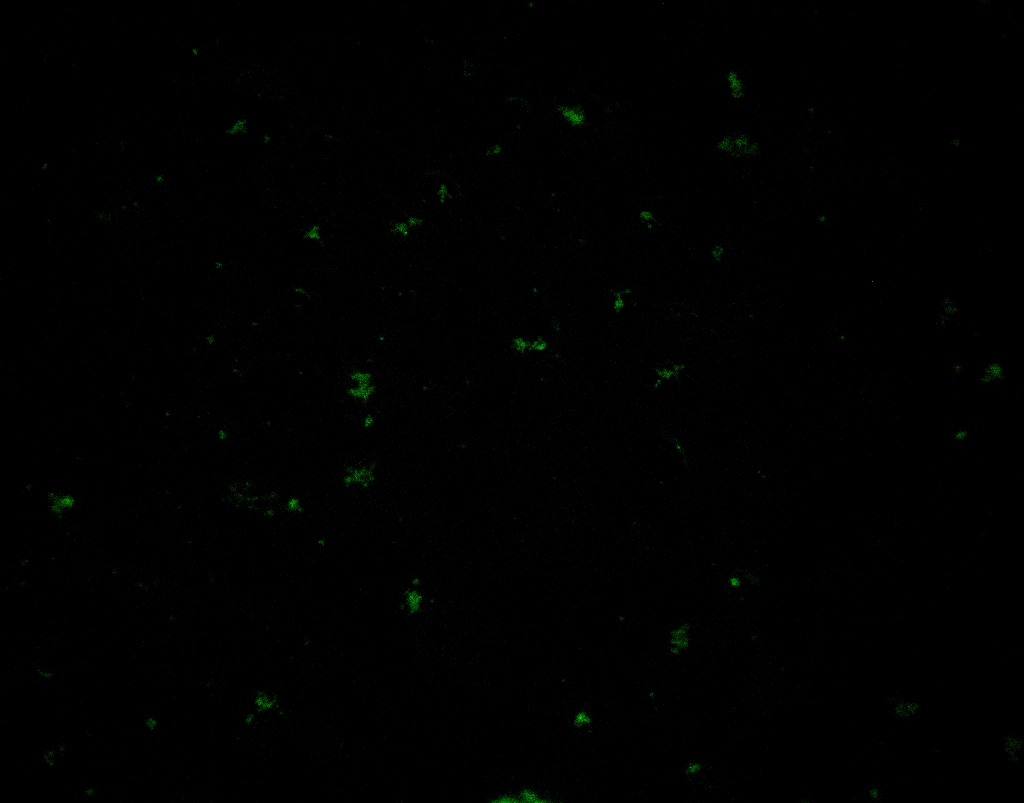

Supplement: Supplementary file 5 — Expanded View Figure Source Data [file 44319_2024_180_MOESM5_ESM.zip › EV Figure Source/EV5/EV5A/extraCol1_NT.jpg]
