# Supplementary material for: Lactate supports cell-autonomous ECM production to sustain metastatic behavior in prostate cancer
Source: EMBO Rep. 2024 Jun 21;25(8):19. doi: 10.1038/s44319-024-00180-z (PMC11315984; doi:10.1038/s44319-024-00180-z)
Supplement: Supplementary file 5 — Expanded View Figure Source Data [file 44319_2024_180_MOESM5_ESM.zip › EV Figure Source/EV1/EV1E/HALLMARK_HYPOXIA.html]

Details for gene set HALLMARK\_HYPOXIA[GSEA]

|  || Dataset | LAvsCTRL\_all\_sig\_stat.rnk\_remapped |
| Phenotype | NoPhenotypeAvailable |
| Upregulated in class | na\_pos |
| GeneSet | HALLMARK\_HYPOXIA |
| Enrichment Score (ES) | 0.5064479 |
| Normalized Enrichment Score (NES) | 3.3678775 |
| Nominal p-value | 0.0 |
| FDR q-value | 0.0 |
| FWER p-Value | 0.0 |
Table: GSEA Results Summary

  

Fig 1: Enrichment plot: HALLMARK\_HYPOXIA      
 Profile of the Running ES Score & Positions of GeneSet Members on the Rank Ordered List

  

| SYMBOL | TITLE | RANK IN GENE LIST | RANK METRIC SCORE | RUNNING ES | CORE ENRICHMENT || 1 | PPFIA4 | PTPRF interacting protein alpha 4 [Source:HGNC Symbol;Acc:HGNC:9248] | 9 | 9.459 | 0.0278 | Yes |
| 2 | ENO2 | enolase 2 [Source:HGNC Symbol;Acc:HGNC:3353] | 14 | 9.093 | 0.0556 | Yes |
| 3 | LOX | lysyl oxidase [Source:HGNC Symbol;Acc:HGNC:6664] | 15 | 8.983 | 0.0841 | Yes |
| 4 | NDRG1 | N-myc downstream regulated 1 [Source:HGNC Symbol;Acc:HGNC:7679] | 23 | 8.021 | 0.1079 | Yes |
| 5 | PGK1 | phosphoglycerate kinase 1 [Source:HGNC Symbol;Acc:HGNC:8896] | 26 | 7.756 | 0.1320 | Yes |
| 6 | ANKZF1 | ankyrin repeat and zinc finger peptidyl tRNA hydrolase 1 [Source:HGNC Symbol;Acc:HGNC:25527] | 27 | 7.751 | 0.1566 | Yes |
| 7 | PDK1 | pyruvate dehydrogenase kinase 1 [Source:HGNC Symbol;Acc:HGNC:8809] | 30 | 7.683 | 0.1804 | Yes |
| 8 | P4HA1 | prolyl 4-hydroxylase subunit alpha 1 [Source:HGNC Symbol;Acc:HGNC:8546] | 34 | 7.606 | 0.2038 | Yes |
| 9 | GAA | glucosidase alpha, acid [Source:HGNC Symbol;Acc:HGNC:4065] | 54 | 7.175 | 0.2219 | Yes |
| 10 | PFKL | phosphofructokinase, liver type [Source:HGNC Symbol;Acc:HGNC:8876] | 56 | 7.122 | 0.2442 | Yes |
| 11 | IGFBP3 | insulin like growth factor binding protein 3 [Source:HGNC Symbol;Acc:HGNC:5472] | 95 | 6.609 | 0.2558 | Yes |
| 12 | CCN5 | cellular communication network factor 5 [Source:HGNC Symbol;Acc:HGNC:12770] | 96 | 6.600 | 0.2767 | Yes |
| 13 | FAM162A | family with sequence similarity 162 member A [Source:HGNC Symbol;Acc:HGNC:17865] | 101 | 6.572 | 0.2966 | Yes |
| 14 | BHLHE40 | basic helix-loop-helix family member e40 [Source:HGNC Symbol;Acc:HGNC:1046] | 107 | 6.510 | 0.3160 | Yes |
| 15 | ANGPTL4 | angiopoietin like 4 [Source:HGNC Symbol;Acc:HGNC:16039] | 108 | 6.504 | 0.3366 | Yes |
| 16 | GYS1 | glycogen synthase 1 [Source:HGNC Symbol;Acc:HGNC:4706] | 148 | 6.043 | 0.3461 | Yes |
| 17 | P4HA2 | prolyl 4-hydroxylase subunit alpha 2 [Source:HGNC Symbol;Acc:HGNC:8547] | 194 | 5.746 | 0.3532 | Yes |
| 18 | GALK1 | galactokinase 1 [Source:HGNC Symbol;Acc:HGNC:4118] | 206 | 5.637 | 0.3684 | Yes |
| 19 | HK2 | hexokinase 2 [Source:HGNC Symbol;Acc:HGNC:4923] | 243 | 5.457 | 0.3768 | Yes |
| 20 | KDELR3 | KDEL endoplasmic reticulum protein retention receptor 3 [Source:HGNC Symbol;Acc:HGNC:6306] | 302 | 5.199 | 0.3789 | Yes |
| 21 | BNIP3L | BCL2 interacting protein 3 like [Source:HGNC Symbol;Acc:HGNC:1085] | 357 | 4.999 | 0.3814 | Yes |
| 22 | PGM1 | phosphoglucomutase 1 [Source:HGNC Symbol;Acc:HGNC:8905] | 359 | 4.998 | 0.3970 | Yes |
| 23 | RORA | RAR related orphan receptor A [Source:HGNC Symbol;Acc:HGNC:10258] | 381 | 4.924 | 0.4074 | Yes |
| 24 | JUN | Jun proto-oncogene, AP-1 transcription factor subunit [Source:HGNC Symbol;Acc:HGNC:6204] | 399 | 4.860 | 0.4186 | Yes |
| 25 | ILVBL | ilvB acetolactate synthase like [Source:HGNC Symbol;Acc:HGNC:6041] | 505 | 4.603 | 0.4072 | Yes |
| 26 | EFNA3 | ephrin A3 [Source:HGNC Symbol;Acc:HGNC:3223] | 558 | 4.487 | 0.4085 | Yes |
| 27 | CSRP2 | cysteine and glycine rich protein 2 [Source:HGNC Symbol;Acc:HGNC:2470] | 577 | 4.440 | 0.4182 | Yes |
| 28 | TPI1 | triosephosphate isomerase 1 [Source:HGNC Symbol;Acc:HGNC:12009] | 588 | 4.400 | 0.4296 | Yes |
| 29 | ENO1 | enolase 1 [Source:HGNC Symbol;Acc:HGNC:3350] | 642 | 4.275 | 0.4301 | Yes |
| 30 | GPC1 | glypican 1 [Source:HGNC Symbol;Acc:HGNC:4449] | 656 | 4.248 | 0.4403 | Yes |
| 31 | CAVIN3 | caveolae associated protein 3 [Source:HGNC Symbol;Acc:HGNC:9400] | 709 | 4.155 | 0.4406 | Yes |
| 32 | PDK3 | pyruvate dehydrogenase kinase 3 [Source:HGNC Symbol;Acc:HGNC:8811] | 724 | 4.134 | 0.4503 | Yes |
| 33 | TMEM45A | transmembrane protein 45A [Source:HGNC Symbol;Acc:HGNC:25480] | 807 | 3.984 | 0.4426 | Yes |
| 34 | GBE1 | 1,4-alpha-glucan branching enzyme 1 [Source:HGNC Symbol;Acc:HGNC:4180] | 812 | 3.978 | 0.4542 | Yes |
| 35 | DDIT4 | DNA damage inducible transcript 4 [Source:HGNC Symbol;Acc:HGNC:24944] | 897 | 3.841 | 0.4456 | Yes |
| 36 | ALDOA | aldolase, fructose-bisphosphate A [Source:HGNC Symbol;Acc:HGNC:414] | 904 | 3.830 | 0.4562 | Yes |
| 37 | SLC25A1 | solute carrier family 25 member 1 [Source:HGNC Symbol;Acc:HGNC:10979] | 959 | 3.760 | 0.4548 | Yes |
| 38 | DPYSL4 | dihydropyrimidinase like 4 [Source:HGNC Symbol;Acc:HGNC:3016] | 966 | 3.749 | 0.4652 | Yes |
| 39 | PLIN2 | perilipin 2 [Source:HGNC Symbol;Acc:HGNC:248] | 993 | 3.715 | 0.4705 | Yes |
| 40 | PGF | placental growth factor [Source:HGNC Symbol;Acc:HGNC:8893] | 1025 | 3.662 | 0.4745 | Yes |
| 41 | PFKFB3 | 6-phosphofructo-2-kinase/fructose-2,6-biphosphatase 3 [Source:HGNC Symbol;Acc:HGNC:8874] | 1079 | 3.602 | 0.4727 | Yes |
| 42 | PRDX5 | peroxiredoxin 5 [Source:HGNC Symbol;Acc:HGNC:9355] | 1115 | 3.557 | 0.4754 | Yes |
| 43 | GPI | glucose-6-phosphate isomerase [Source:HGNC Symbol;Acc:HGNC:4458] | 1175 | 3.460 | 0.4717 | Yes |
| 44 | IL6 | interleukin 6 [Source:HGNC Symbol;Acc:HGNC:6018] | 1178 | 3.460 | 0.4822 | Yes |
| 45 | DTNA | dystrobrevin alpha [Source:HGNC Symbol;Acc:HGNC:3057] | 1190 | 3.449 | 0.4904 | Yes |
| 46 | SDC3 | syndecan 3 [Source:HGNC Symbol;Acc:HGNC:10660] | 1259 | 3.364 | 0.4842 | Yes |
| 47 | TGFBI | transforming growth factor beta induced [Source:HGNC Symbol;Acc:HGNC:11771] | 1310 | 3.309 | 0.4823 | Yes |
| 48 | SRPX | sushi repeat containing protein X-linked [Source:HGNC Symbol;Acc:HGNC:11309] | 1361 | 3.252 | 0.4802 | Yes |
| 49 | PFKP | phosphofructokinase, platelet [Source:HGNC Symbol;Acc:HGNC:8878] | 1365 | 3.245 | 0.4898 | Yes |
| 50 | PPP1R3C | protein phosphatase 1 regulatory subunit 3C [Source:HGNC Symbol;Acc:HGNC:9293] | 1393 | 3.215 | 0.4933 | Yes |
| 51 | CITED2 | Cbp/p300 interacting transactivator with Glu/Asp rich carboxy-terminal domain 2 [Source:HGNC Symbol;Acc:HGNC:1987] | 1403 | 3.205 | 0.5012 | Yes |
| 52 | PKP1 | plakophilin 1 [Source:HGNC Symbol;Acc:HGNC:9023] | 1459 | 3.156 | 0.4976 | Yes |
| 53 | SCARB1 | scavenger receptor class B member 1 [Source:HGNC Symbol;Acc:HGNC:1664] | 1473 | 3.147 | 0.5044 | Yes |
| 54 | SLC2A1 | solute carrier family 2 member 1 [Source:HGNC Symbol;Acc:HGNC:11005] | 1532 | 3.096 | 0.4998 | Yes |
| 55 | SLC37A4 | solute carrier family 37 member 4 [Source:HGNC Symbol;Acc:HGNC:4061] | 1578 | 3.050 | 0.4984 | Yes |
| 56 | GRHPR | glyoxylate and hydroxypyruvate reductase [Source:HGNC Symbol;Acc:HGNC:4570] | 1614 | 3.010 | 0.4992 | Yes |
| 57 | HK1 | hexokinase 1 [Source:HGNC Symbol;Acc:HGNC:4922] | 1648 | 2.984 | 0.5005 | Yes |
| 58 | LDHA | lactate dehydrogenase A [Source:HGNC Symbol;Acc:HGNC:6535] | 1663 | 2.964 | 0.5064 | Yes |
| 59 | B3GALT6 | beta-1,3-galactosyltransferase 6 [Source:HGNC Symbol;Acc:HGNC:17978] | 1859 | 2.788 | 0.4670 | No |
| 60 | TPD52 | tumor protein D52 [Source:HGNC Symbol;Acc:HGNC:12005] | 1936 | 2.723 | 0.4568 | No |
| 61 | IRS2 | insulin receptor substrate 2 [Source:HGNC Symbol;Acc:HGNC:6126] | 1958 | 2.704 | 0.4601 | No |
| 62 | SULT2B1 | sulfotransferase family 2B member 1 [Source:HGNC Symbol;Acc:HGNC:11459] | 1996 | 2.667 | 0.4594 | No |
| 63 | MXI1 | MAX interactor 1, dimerization protein [Source:HGNC Symbol;Acc:HGNC:7534] | 2074 | 2.602 | 0.4486 | No |
| 64 | ISG20 | interferon stimulated exonuclease gene 20 [Source:HGNC Symbol;Acc:HGNC:6130] | 2186 | 2.504 | 0.4290 | No |
| 65 | NEDD4L | NEDD4 like E3 ubiquitin protein ligase [Source:HGNC Symbol;Acc:HGNC:7728] | 2276 | -2.444 | 0.4147 | No |
| 66 | NDST1 | N-deacetylase and N-sulfotransferase 1 [Source:HGNC Symbol;Acc:HGNC:7680] | 2297 | -2.459 | 0.4176 | No |
| 67 | VHL | von Hippel-Lindau tumor suppressor [Source:HGNC Symbol;Acc:HGNC:12687] | 2811 | -2.868 | 0.2995 | No |
| 68 | PGM2 | phosphoglucomutase 2 [Source:HGNC Symbol;Acc:HGNC:8906] | 2956 | -2.998 | 0.2733 | No |
| 69 | IDS | iduronate 2-sulfatase [Source:HGNC Symbol;Acc:HGNC:5389] | 3293 | -3.322 | 0.2005 | No |
Table: GSEA details [plain text format]

  

Fig 2: HALLMARK\_HYPOXIA: Random ES distribution      
 Gene set null distribution of ES for **HALLMARK\_HYPOXIA**

  
