# Supplementary figures and images for: Lactate supports cell-autonomous ECM production to sustain metastatic behavior in prostate cancer (part 2 of 3)
Source: EMBO Rep. 2024 Jun 21;25(8):19. doi: 10.1038/s44319-024-00180-z (PMC11315984; doi:10.1038/s44319-024-00180-z)

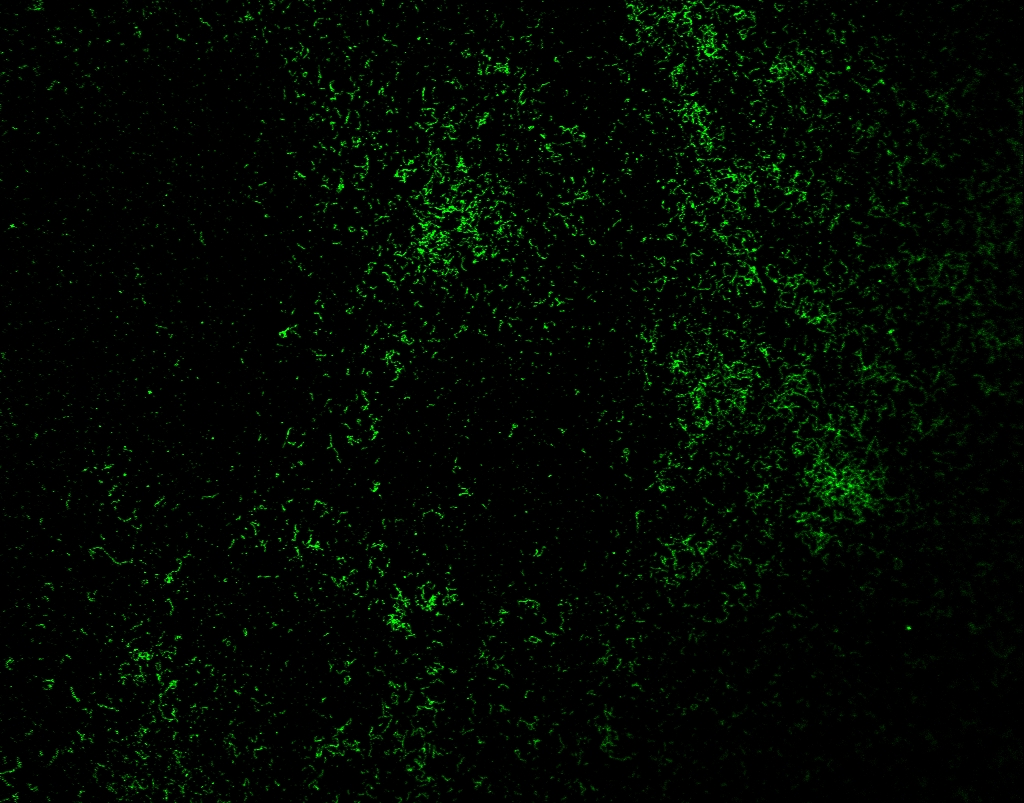

Supplement: Supplementary file 5 — Expanded View Figure Source Data [file 44319_2024_180_MOESM5_ESM.zip › EV Figure Source/EV5/EV5A/extraCol1_CAF.jpg]

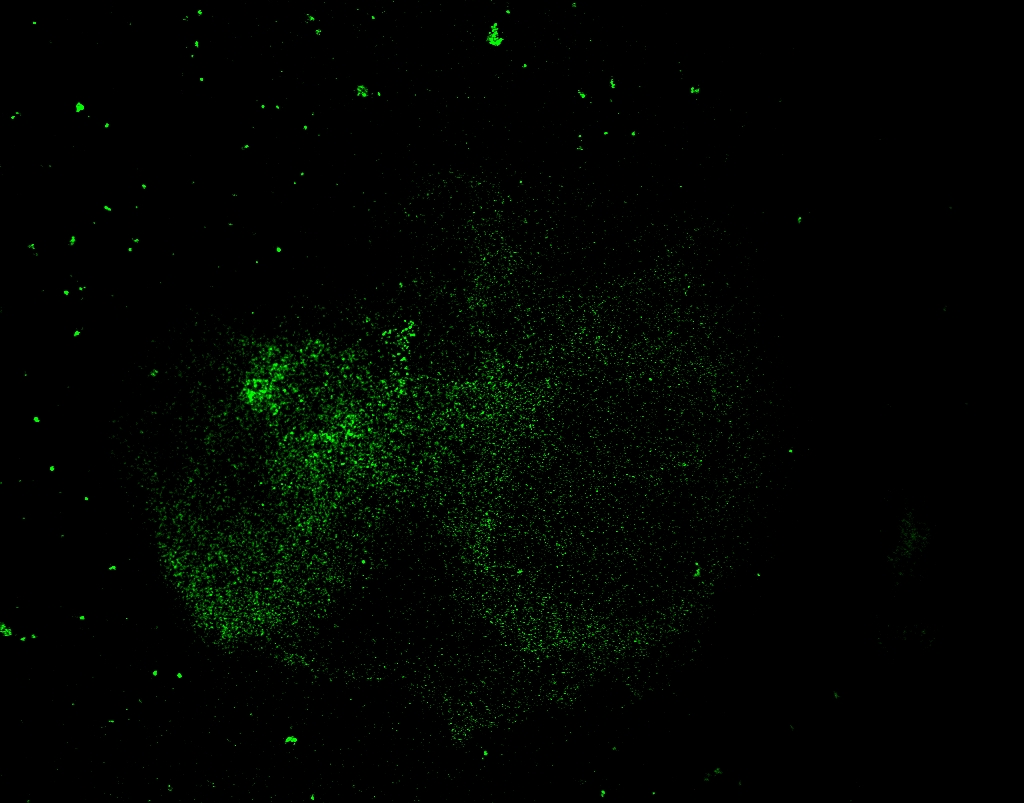

Supplement: Supplementary file 5 — Expanded View Figure Source Data [file 44319_2024_180_MOESM5_ESM.zip › EV Figure Source/EV5/EV5A/extraCol1_Lactate.jpg]

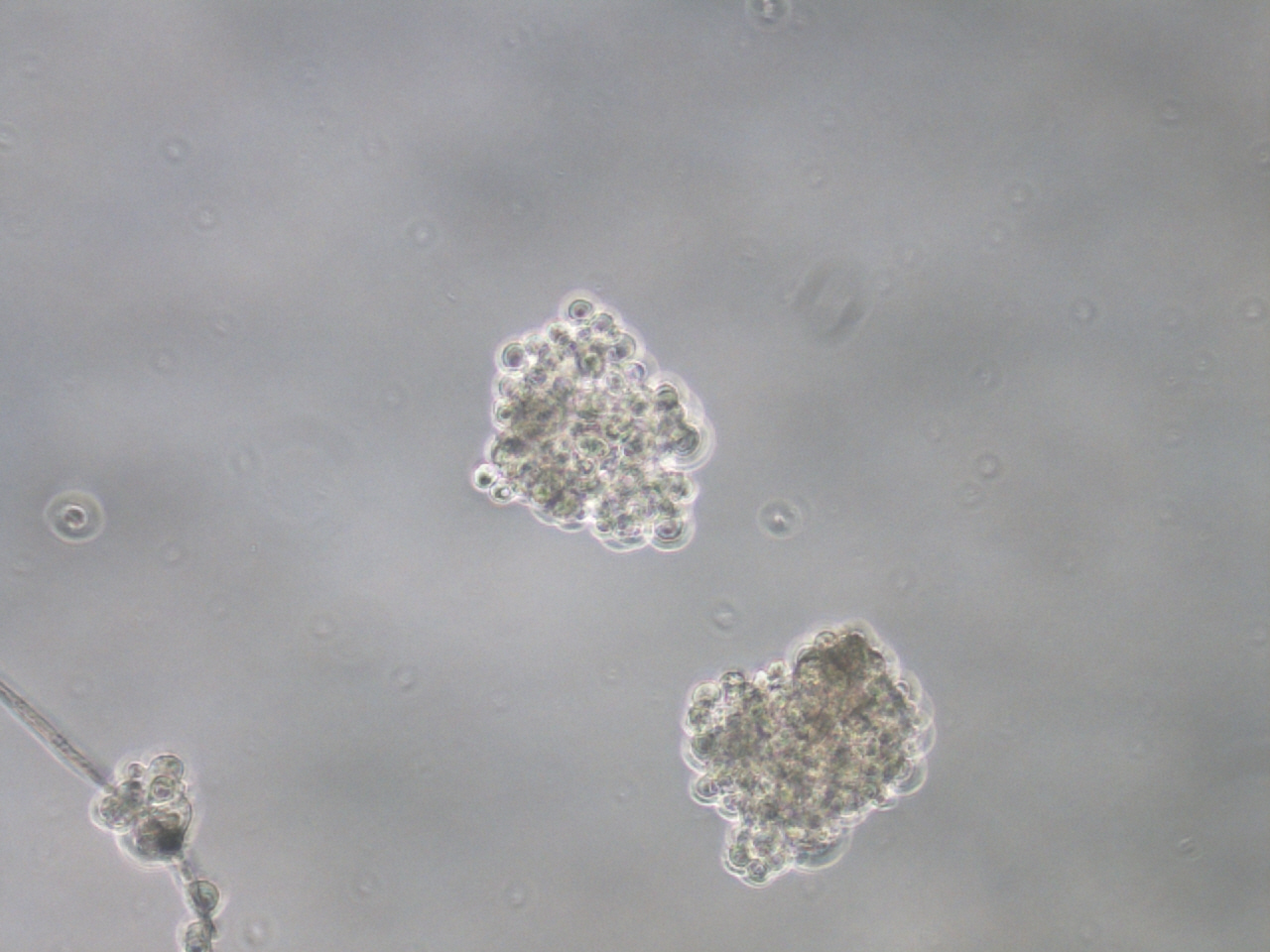

Supplement: Supplementary file 5 — Expanded View Figure Source Data [file 44319_2024_180_MOESM5_ESM.zip › EV Figure Source/EV4/EV4G/LA siDDR1.jpg]

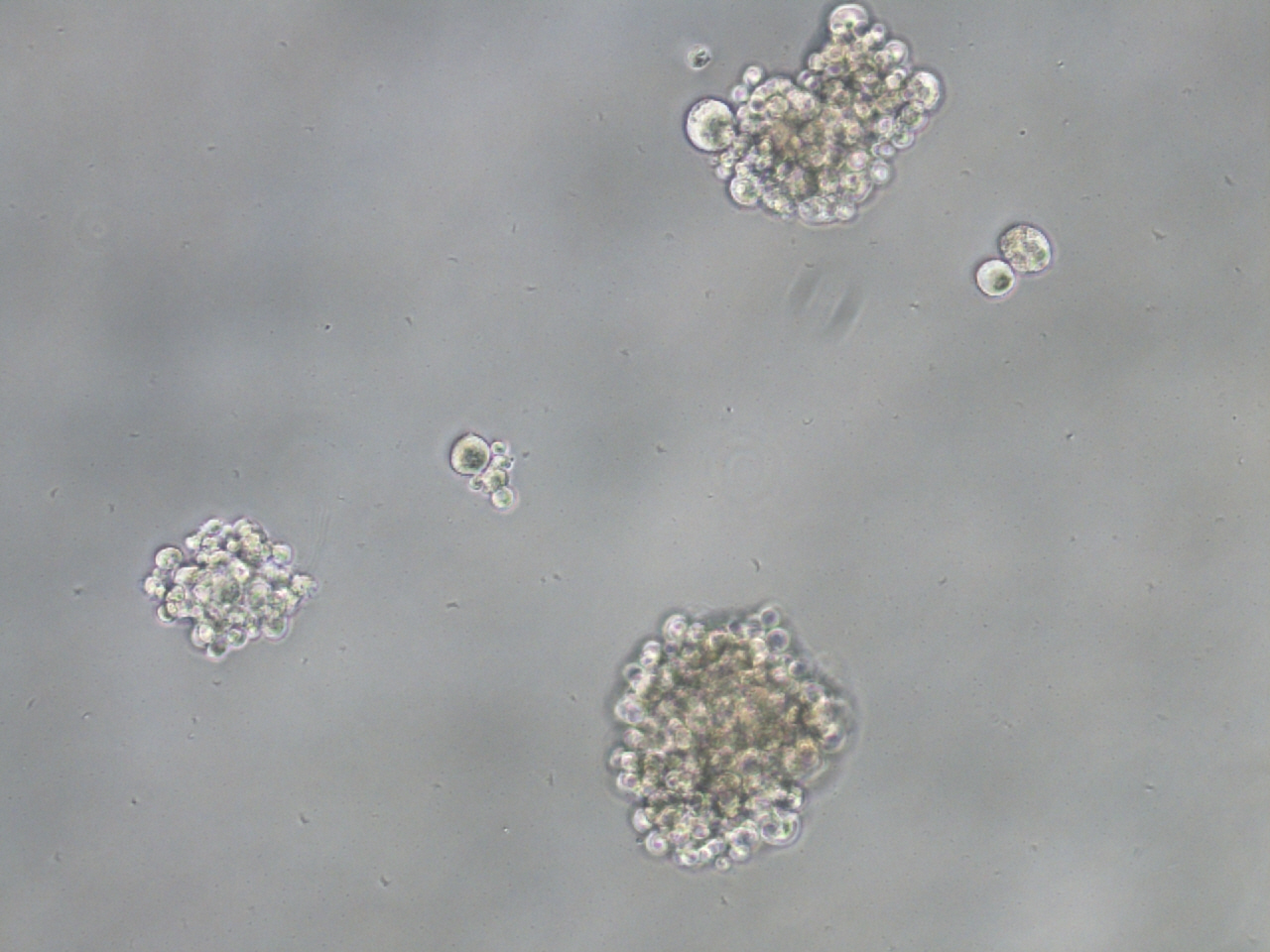

Supplement: Supplementary file 5 — Expanded View Figure Source Data [file 44319_2024_180_MOESM5_ESM.zip › EV Figure Source/EV4/EV4G/HPF-CM siCTR.jpg]

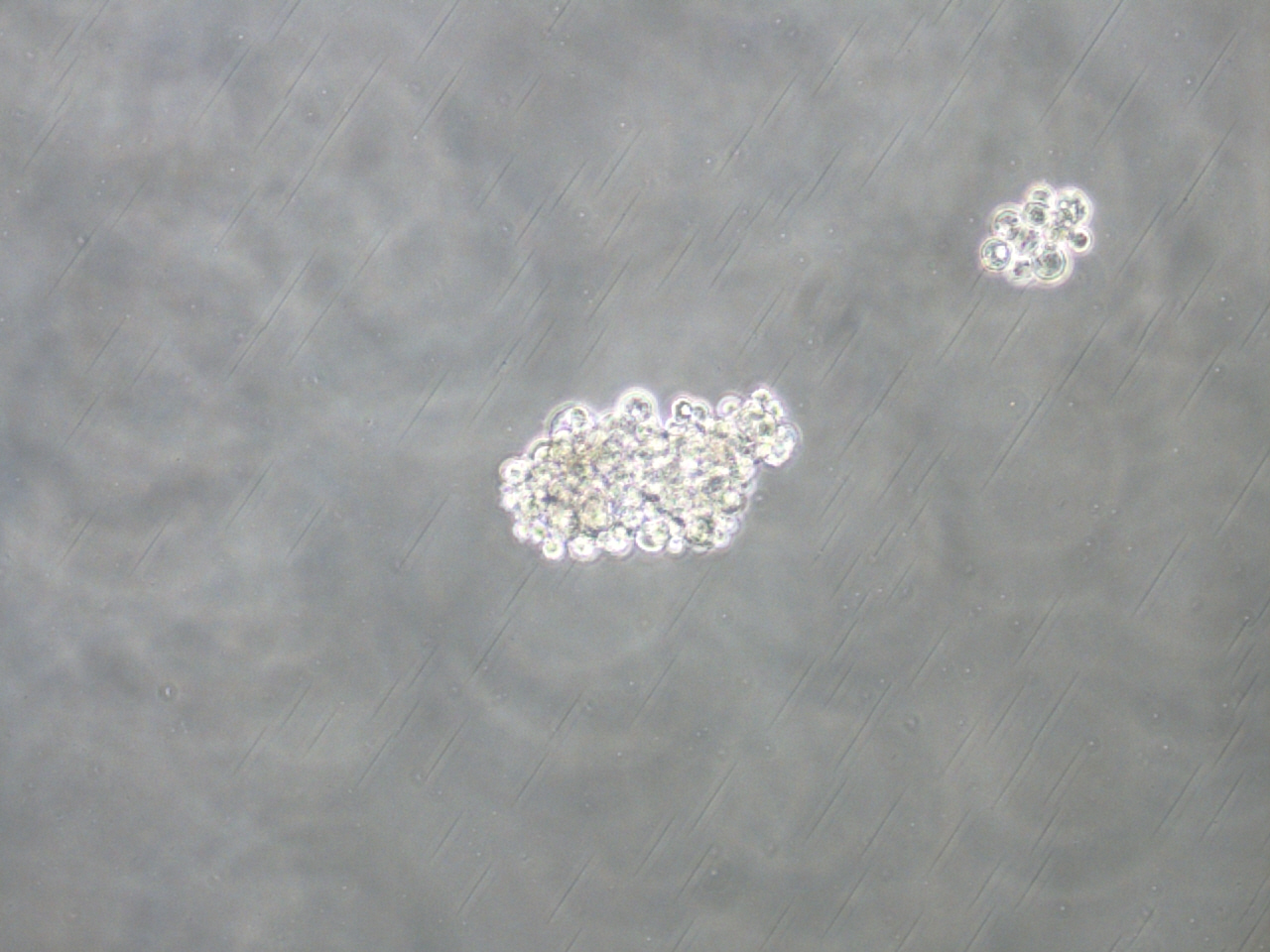

Supplement: Supplementary file 5 — Expanded View Figure Source Data [file 44319_2024_180_MOESM5_ESM.zip › EV Figure Source/EV4/EV4G/CAF-CM siDDR1.jpg]

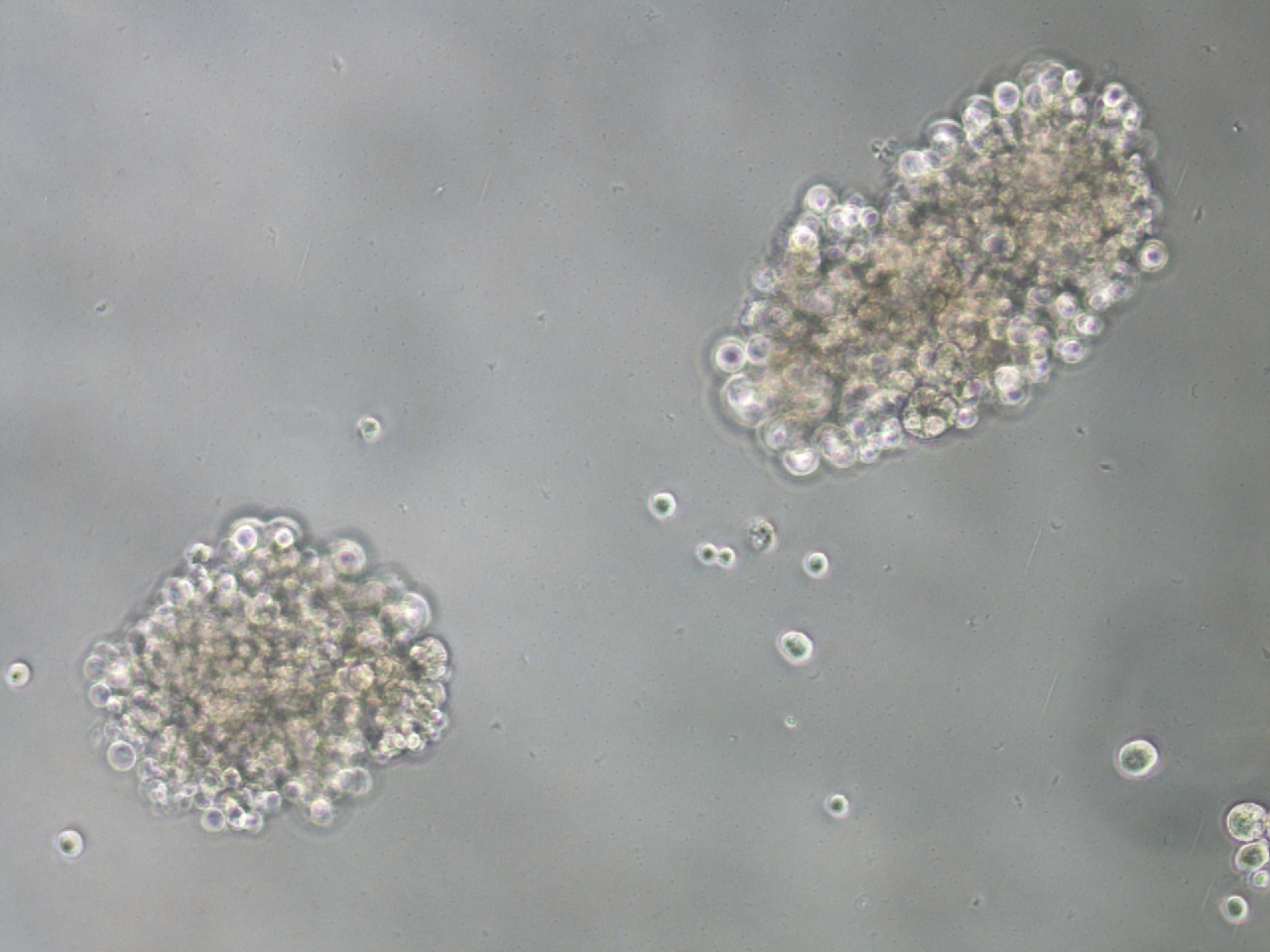

Supplement: Supplementary file 5 — Expanded View Figure Source Data [file 44319_2024_180_MOESM5_ESM.zip › EV Figure Source/EV4/EV4G/LA siCTR.jpg]

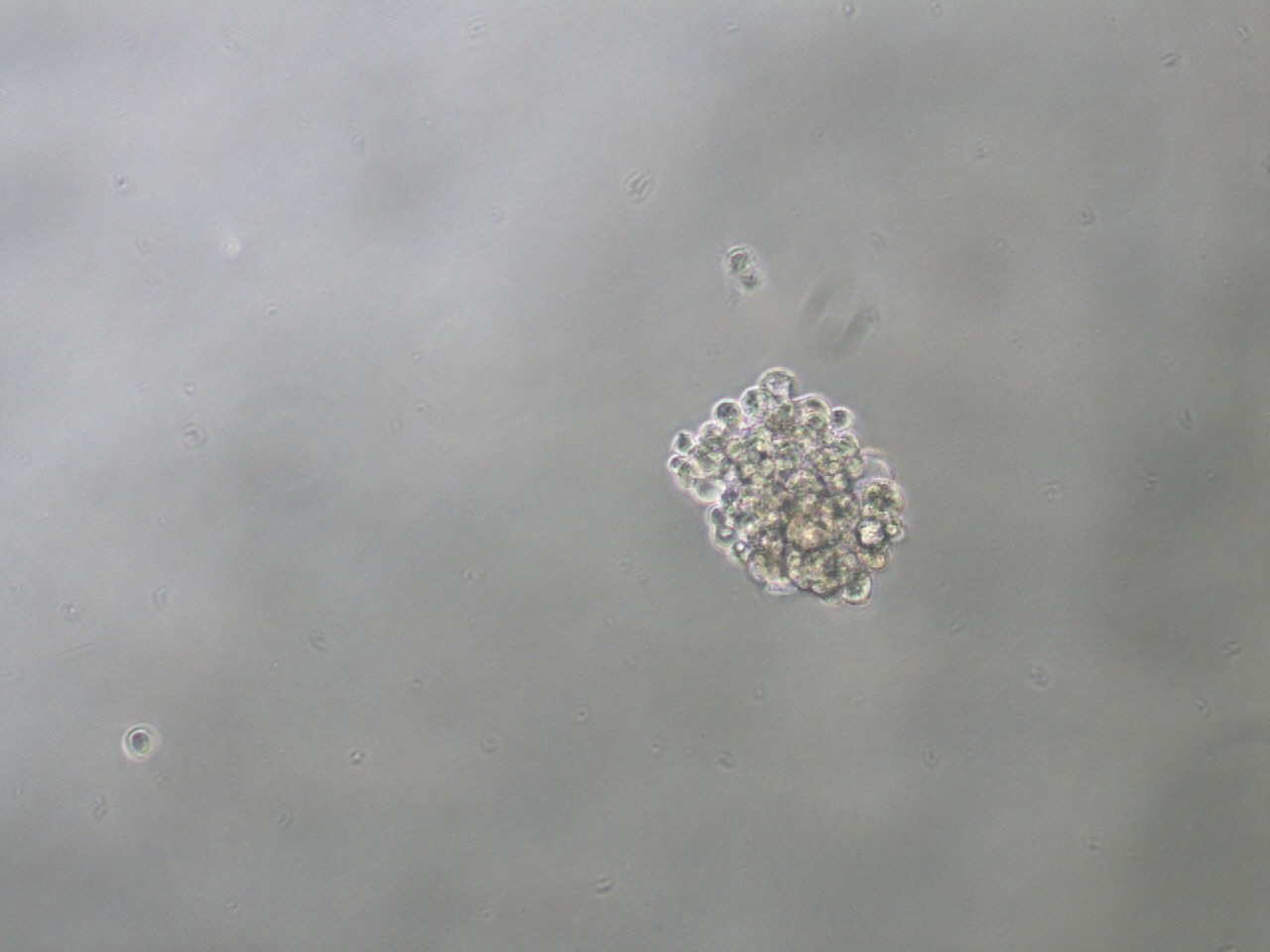

Supplement: Supplementary file 5 — Expanded View Figure Source Data [file 44319_2024_180_MOESM5_ESM.zip › EV Figure Source/EV4/EV4G/HPF-CM siDDR1.jpg]

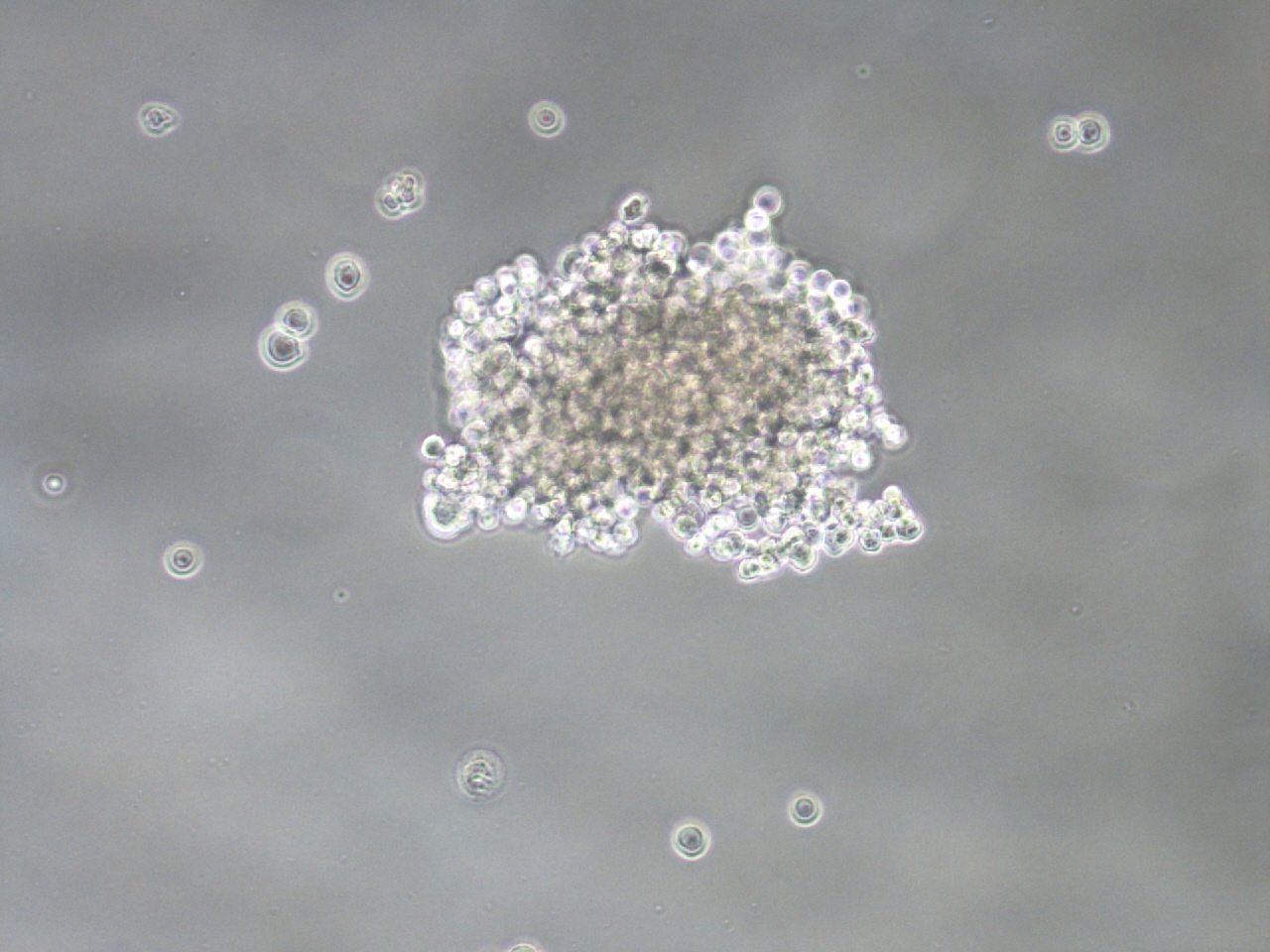

Supplement: Supplementary file 5 — Expanded View Figure Source Data [file 44319_2024_180_MOESM5_ESM.zip › EV Figure Source/EV4/EV4G/CAF-CM siCTR.jpg]

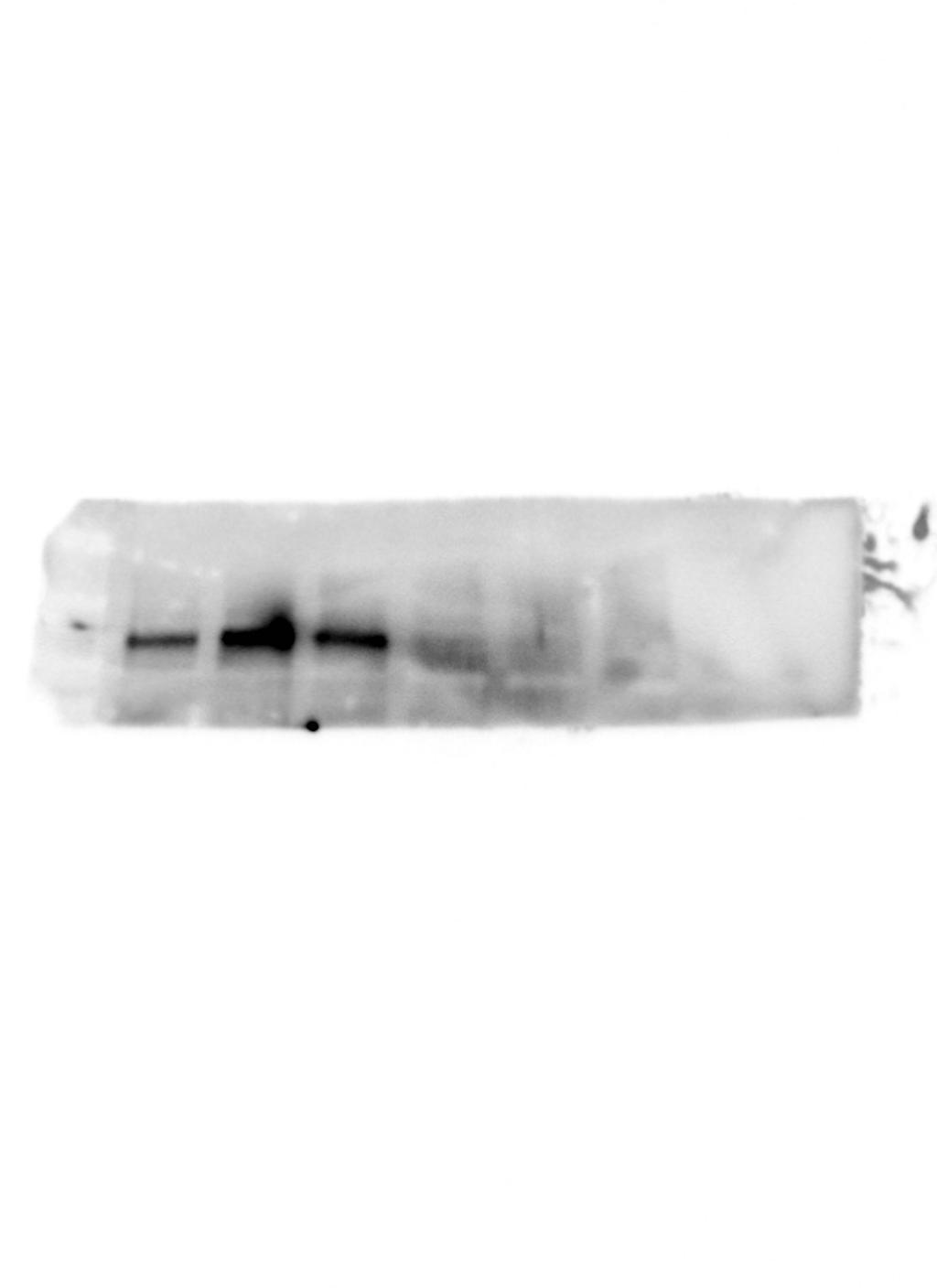

Supplement: Supplementary file 5 — Expanded View Figure Source Data [file 44319_2024_180_MOESM5_ESM.zip › EV Figure Source/EV4/EV4D/WB DDR1 siDDR1.jpg]

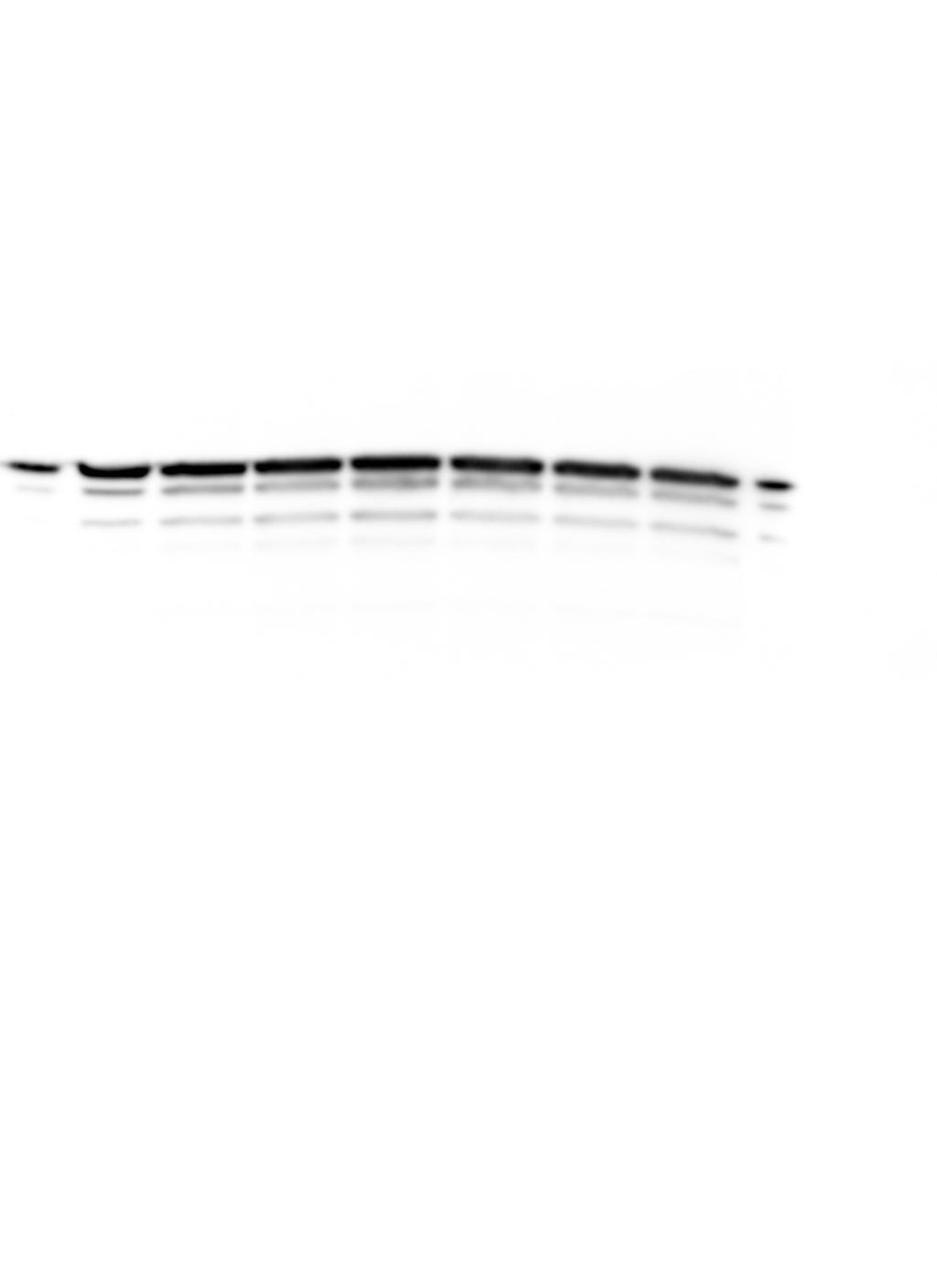

Supplement: Supplementary file 5 — Expanded View Figure Source Data [file 44319_2024_180_MOESM5_ESM.zip › EV Figure Source/EV4/EV4D/WB Actin siDDR1.jpg]

## Expanded View Figure E3D

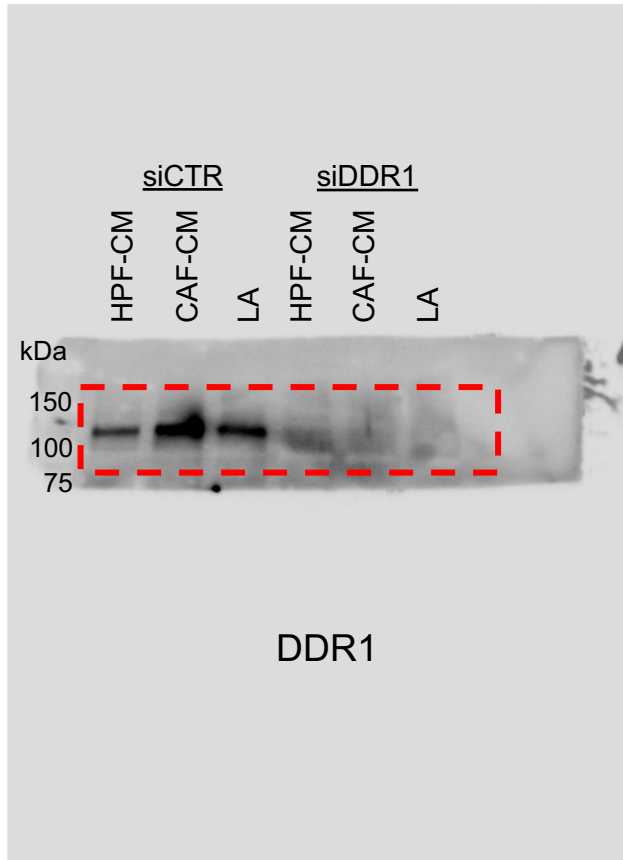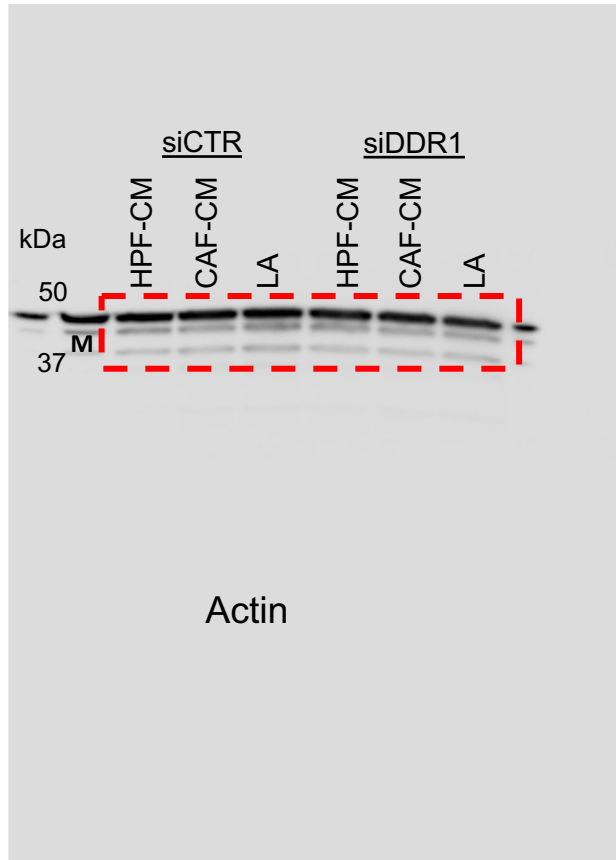

Supplement: Supplementary file 5 — Expanded View Figure Source Data [file 44319_2024_180_MOESM5_ESM.zip › EV Figure Source/EV4/EV4D/EV4D blot.pdf]

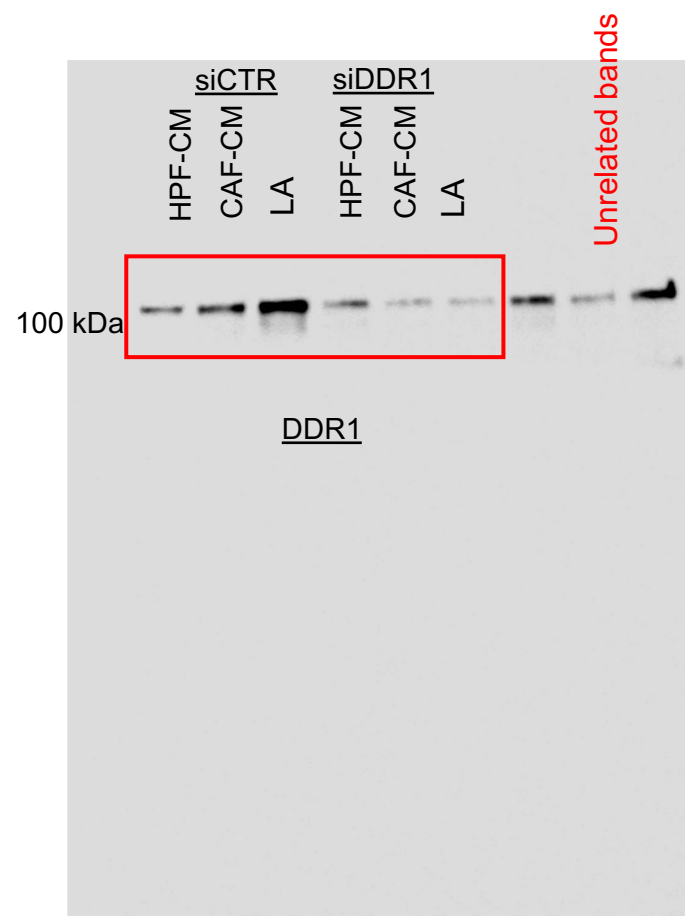

Supplement: Supplementary file 5 — Expanded View Figure Source Data [file 44319_2024_180_MOESM5_ESM.zip › EV Figure Source/EV4/EV4C/WB DDR1 siDDR1.pdf]

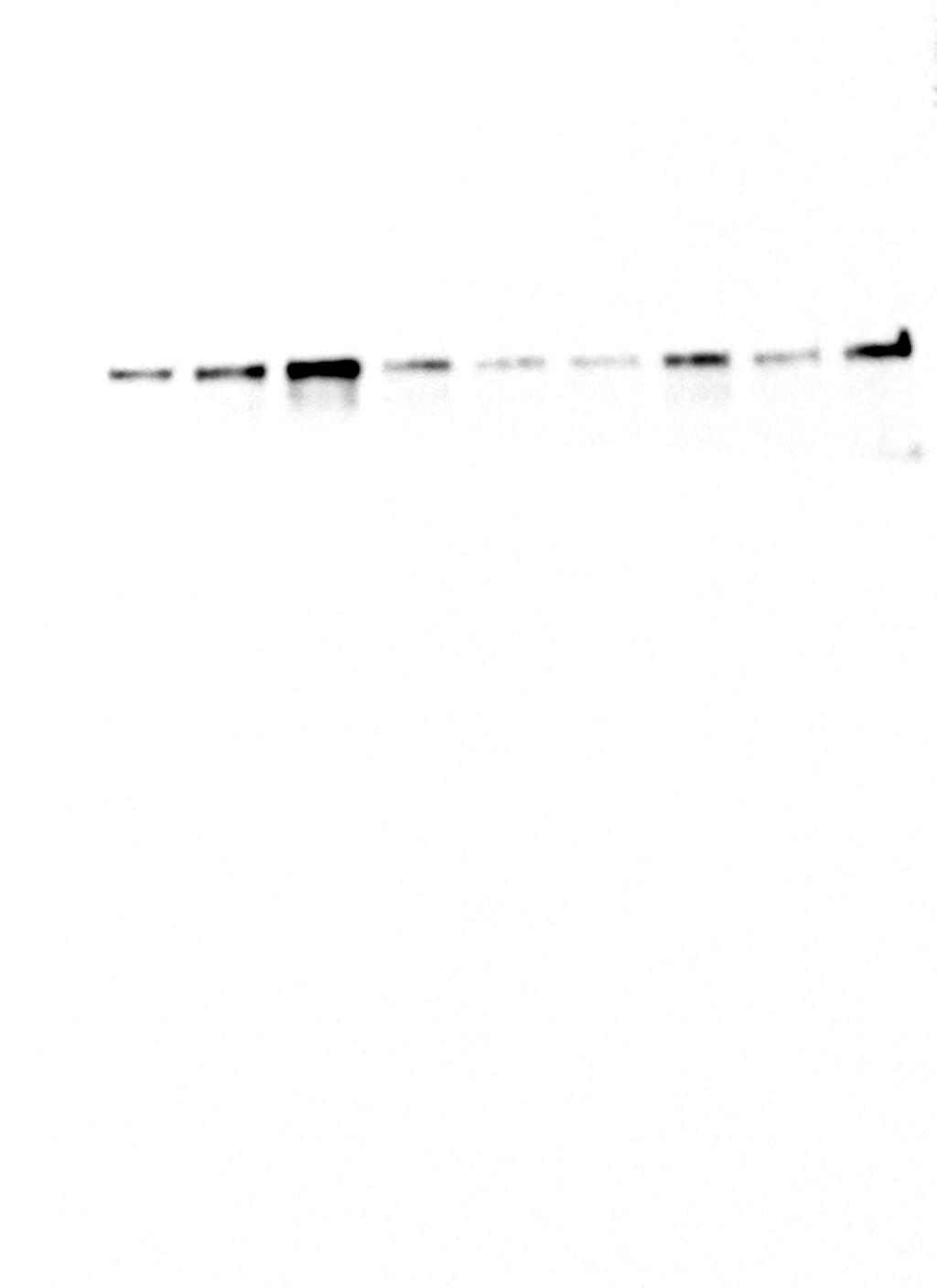

Supplement: Supplementary file 5 — Expanded View Figure Source Data [file 44319_2024_180_MOESM5_ESM.zip › EV Figure Source/EV4/EV4C/WB DDR1 siDDR1.jpg]

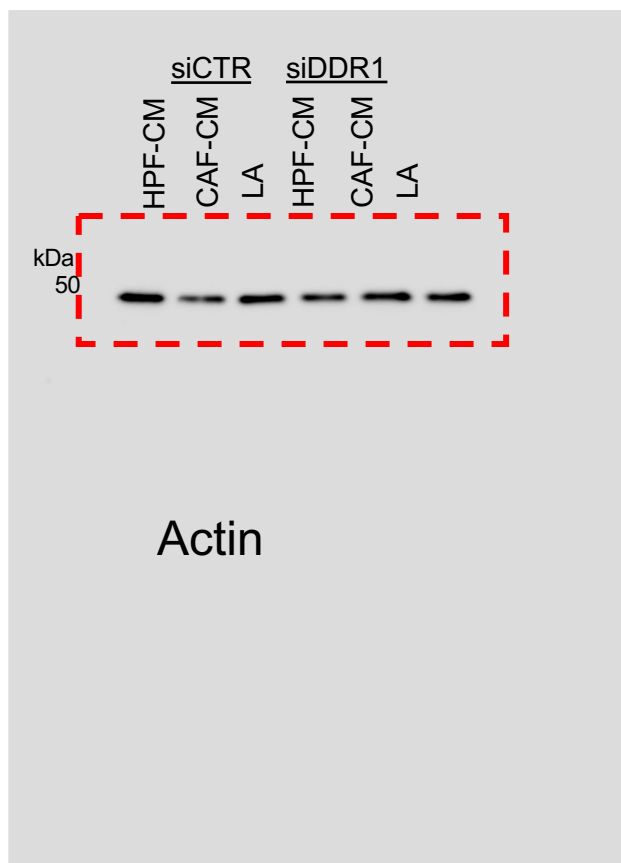

Supplement: Supplementary file 5 — Expanded View Figure Source Data [file 44319_2024_180_MOESM5_ESM.zip › EV Figure Source/EV4/EV4C/WB Actin siDDR1.pdf]

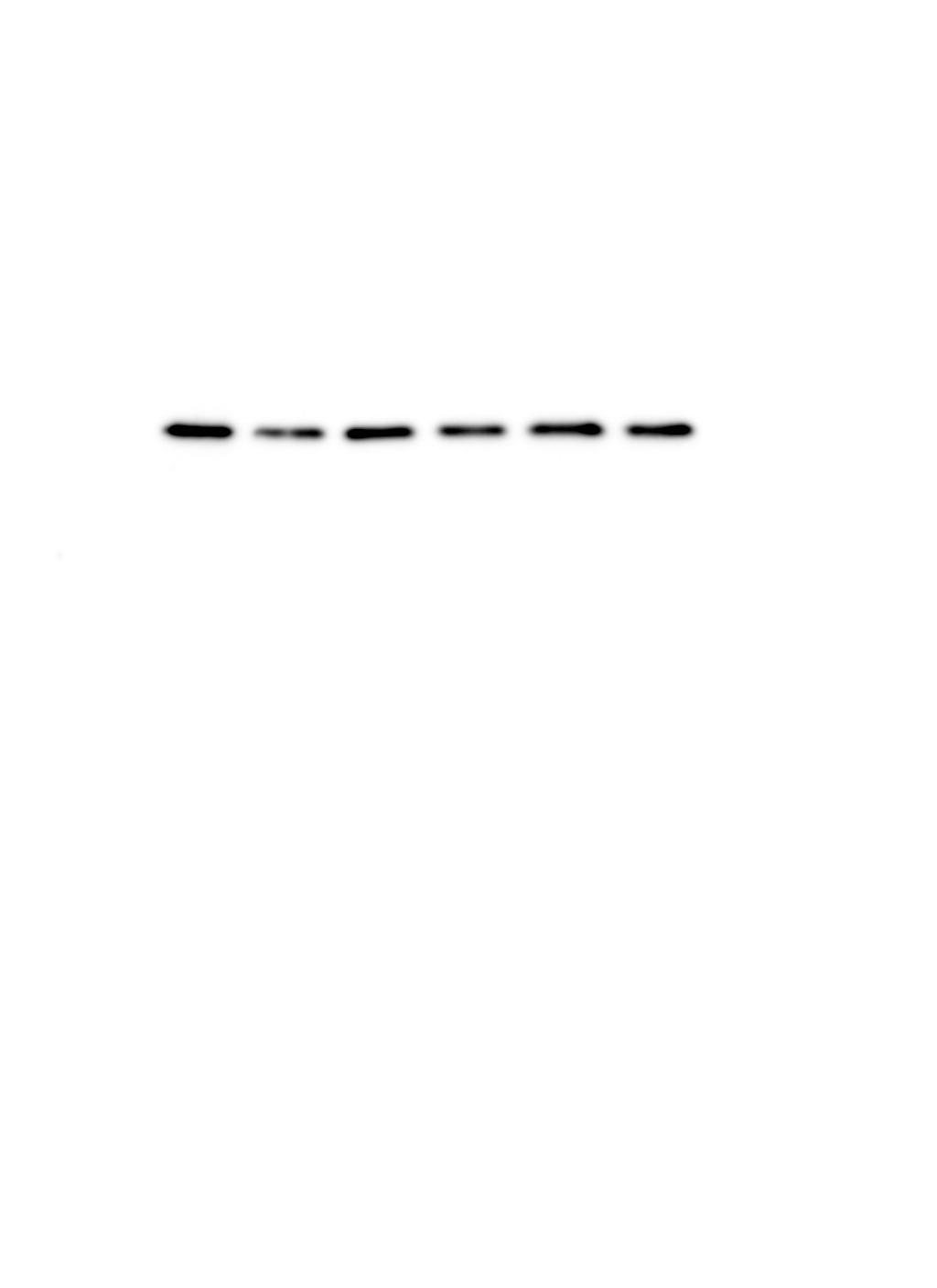

Supplement: Supplementary file 5 — Expanded View Figure Source Data [file 44319_2024_180_MOESM5_ESM.zip › EV Figure Source/EV4/EV4C/WB Actin siDDR1.jpg]

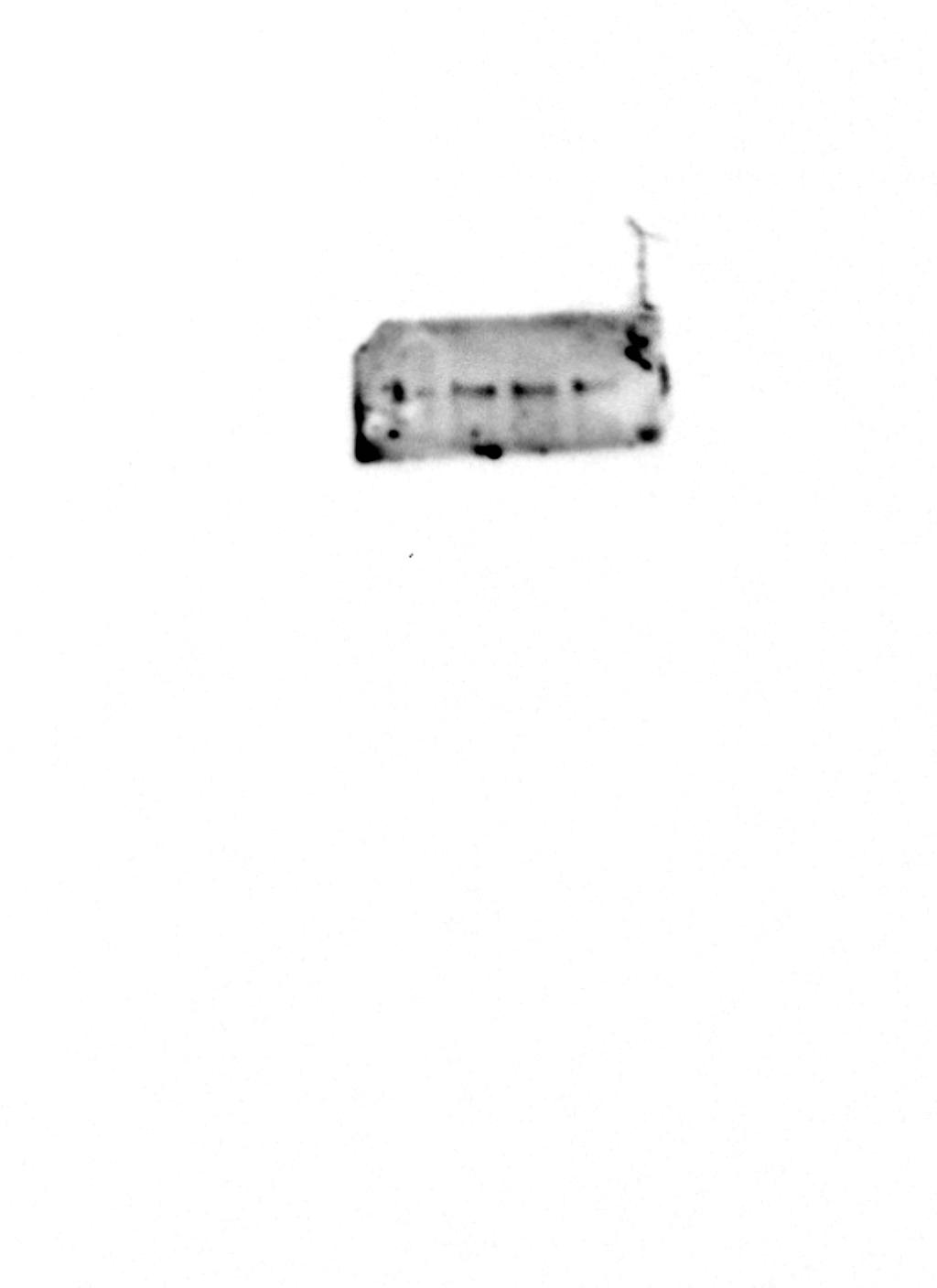

Supplement: Supplementary file 5 — Expanded View Figure Source Data [file 44319_2024_180_MOESM5_ESM.zip › EV Figure Source/EV4/EV4B/WB DDR1_22rv1.jpg]

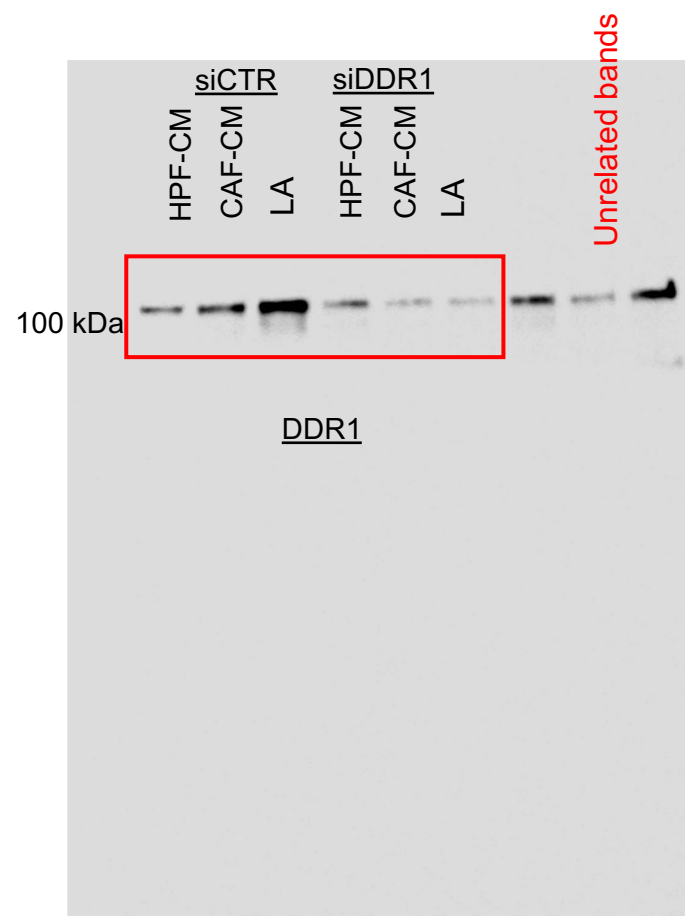

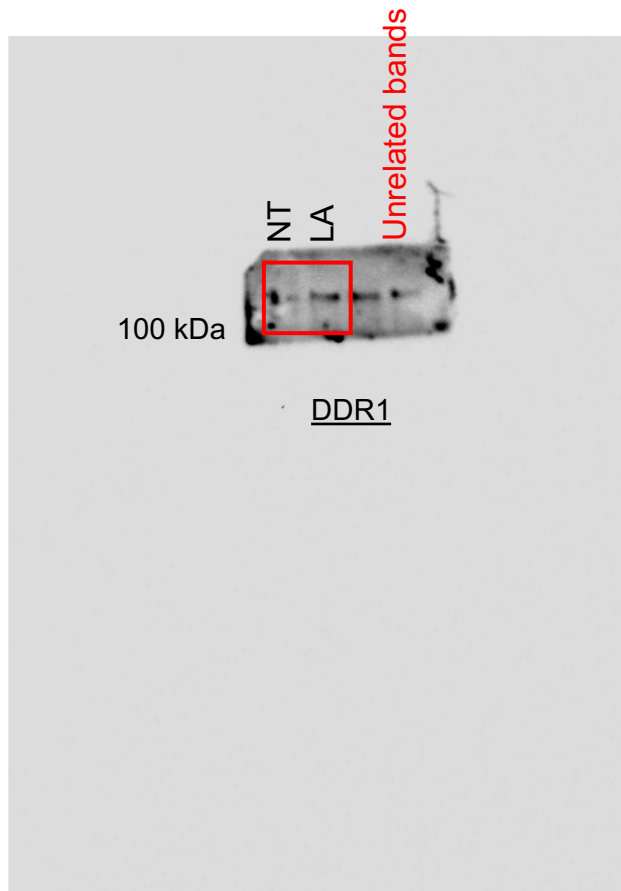

Supplement: Supplementary file 5 — Expanded View Figure Source Data [file 44319_2024_180_MOESM5_ESM.zip › EV Figure Source/EV4/EV4B/WB DDR1_22rv1.pdf]

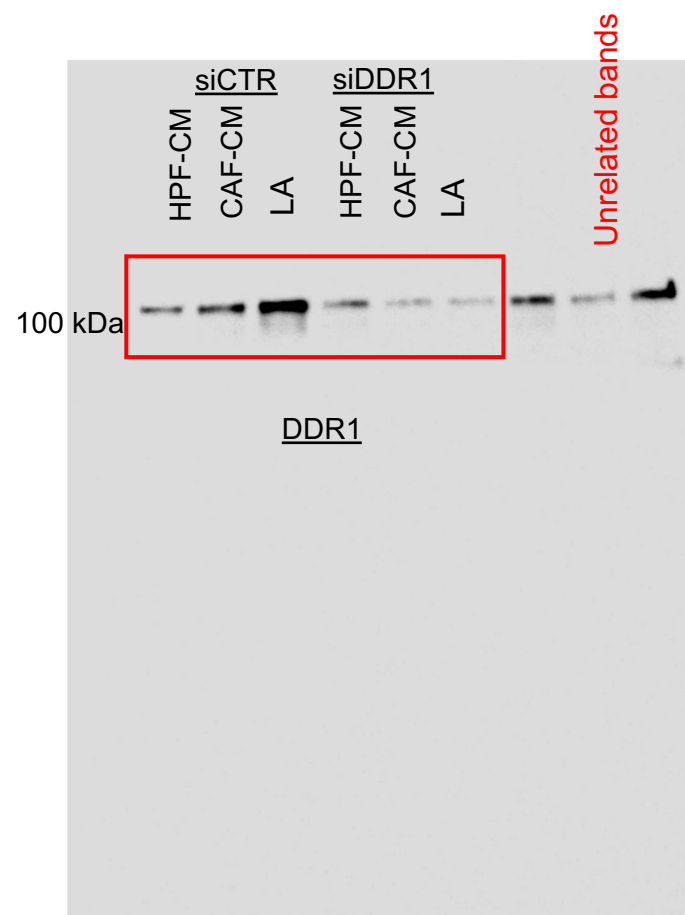

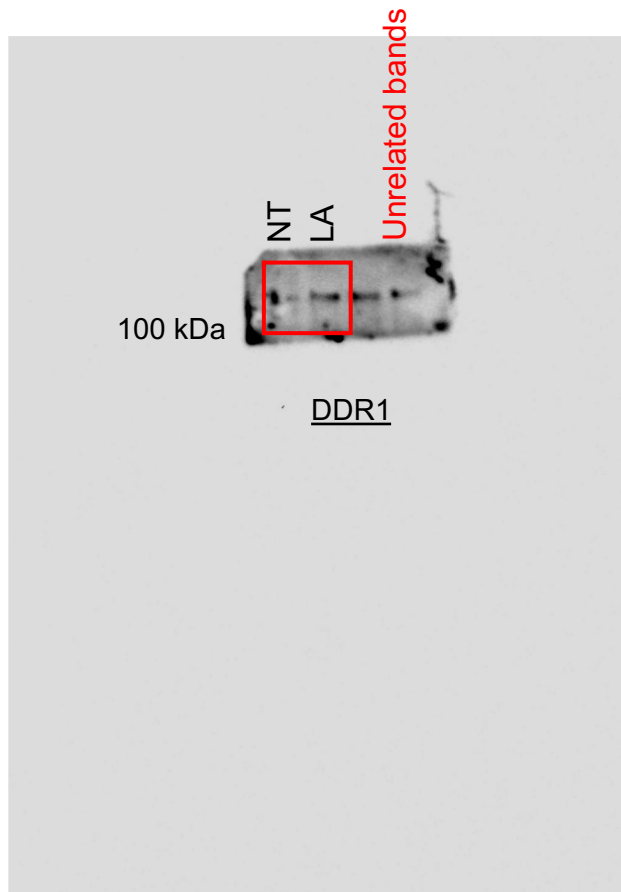

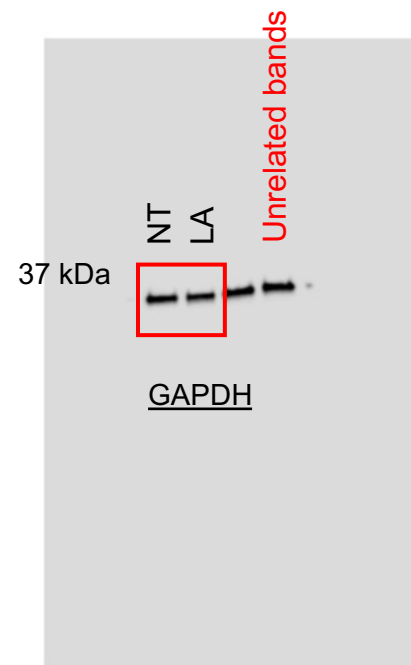

Supplement: Supplementary file 5 — Expanded View Figure Source Data [file 44319_2024_180_MOESM5_ESM.zip › EV Figure Source/EV4/EV4B/WB GAPDH 22Rv1.pdf]

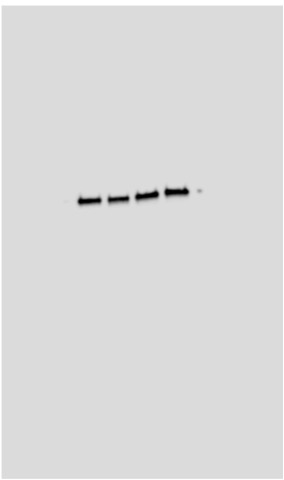

Supplement: Supplementary file 5 — Expanded View Figure Source Data [file 44319_2024_180_MOESM5_ESM.zip › EV Figure Source/EV4/EV4B/WB GAPDH 22Rv1.jpg]

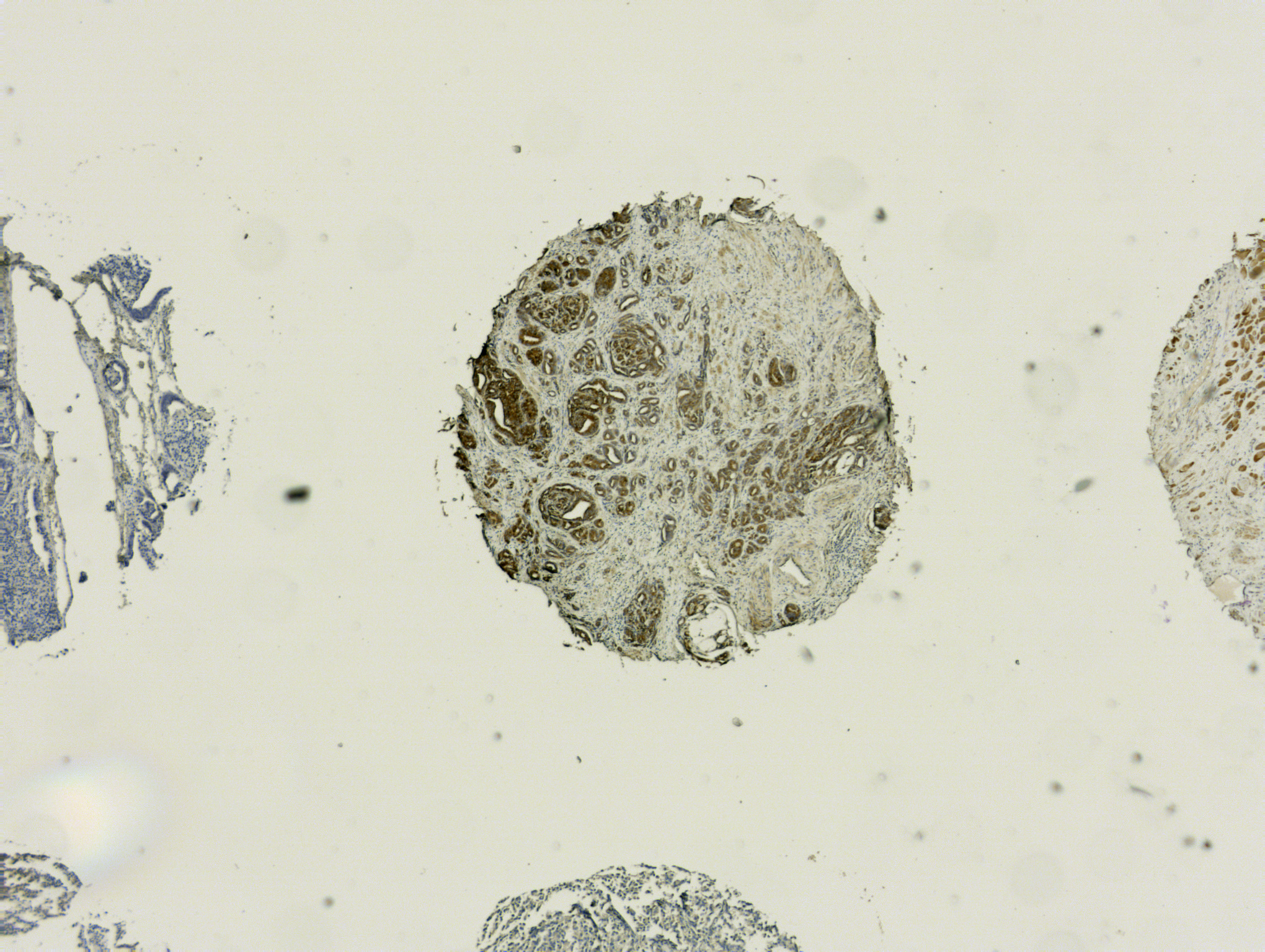

Supplement: Supplementary file 6 — Source data Fig. 1 [file 44319_2024_180_MOESM6_ESM.zip › Figure 1/1H/High Grade P4HA1.tif]

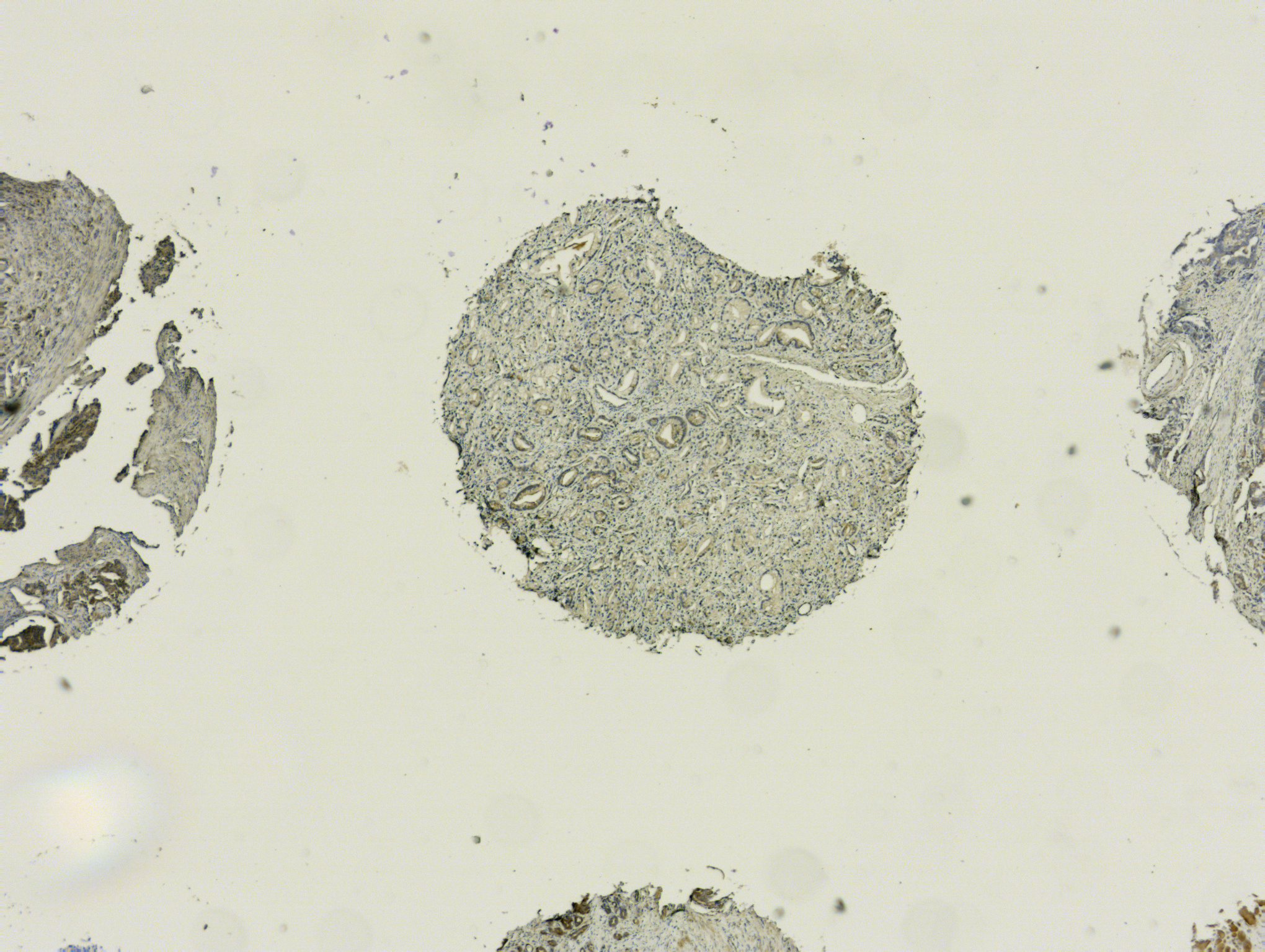

Supplement: Supplementary file 6 — Source data Fig. 1 [file 44319_2024_180_MOESM6_ESM.zip › Figure 1/1H/Low Grade P4HA1.tif]

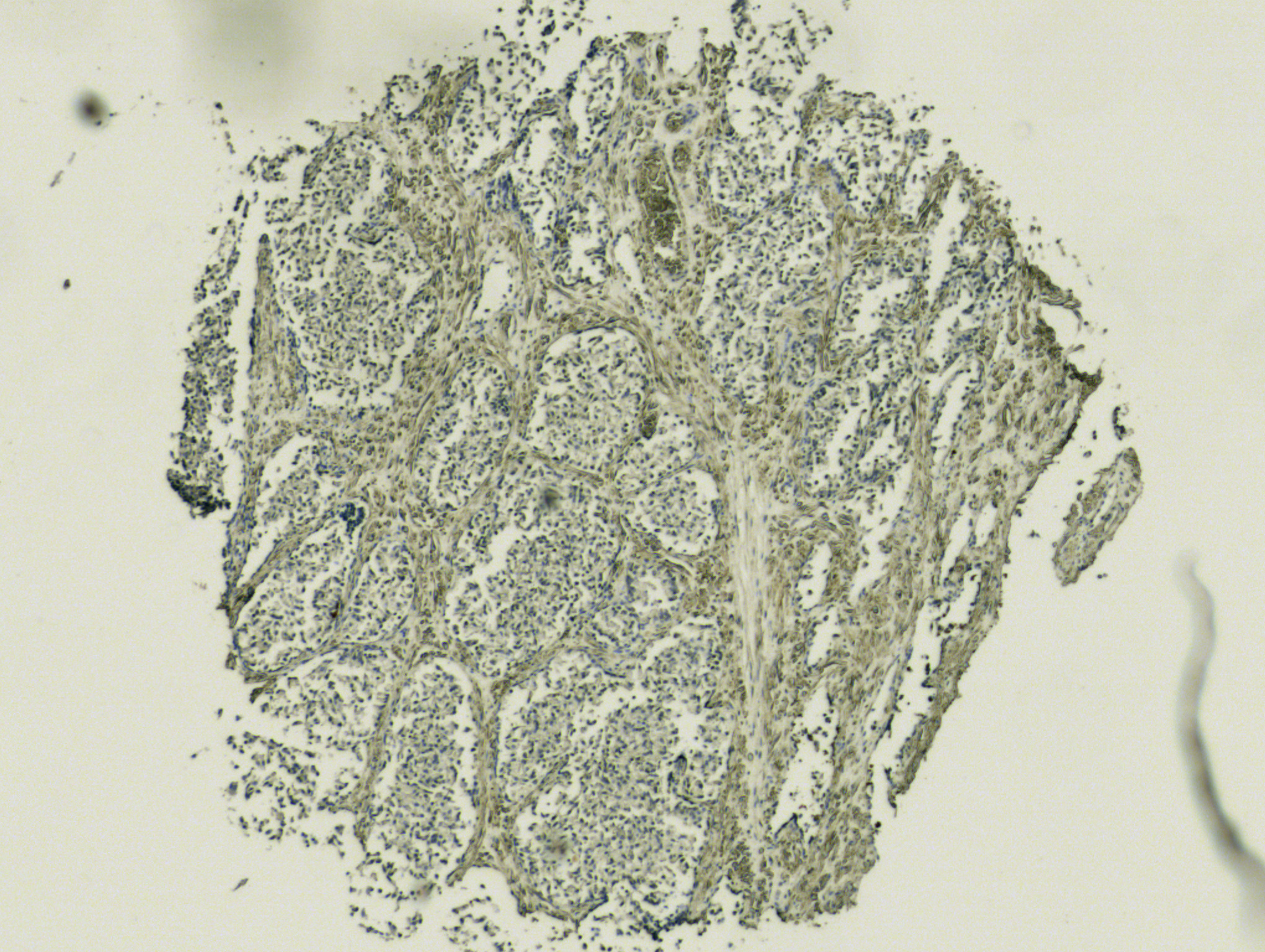

Supplement: Supplementary file 6 — Source data Fig. 1 [file 44319_2024_180_MOESM6_ESM.zip › Figure 1/1H/Benign P4HA1.tif]

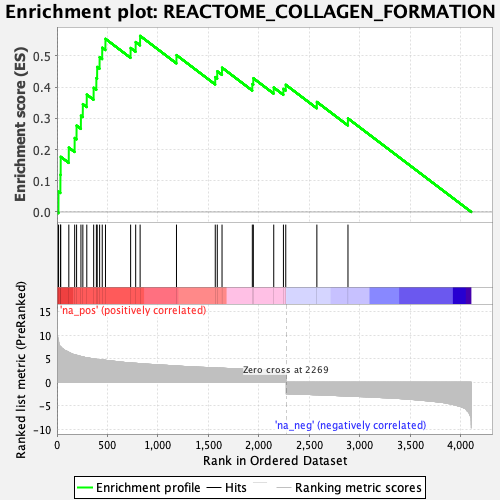

Supplement: Supplementary file 6 — Source data Fig. 1 [file 44319_2024_180_MOESM6_ESM.zip › Figure 1/1A/enplot_REACTOME_COLLAGEN_FORMATION_195.png]

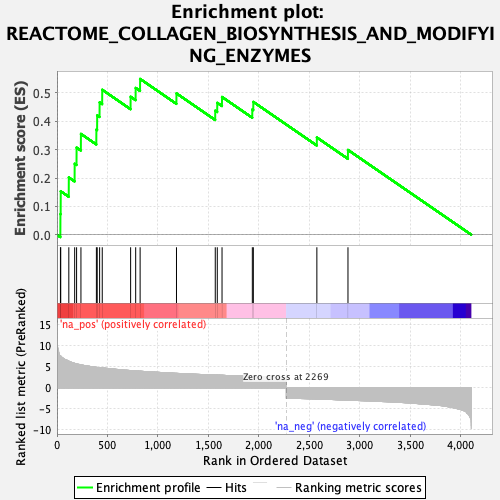

Supplement: Supplementary file 6 — Source data Fig. 1 [file 44319_2024_180_MOESM6_ESM.zip › Figure 1/1A/enplot_REACTOME_COLLAGEN_BIOSYNTHESIS_AND_MODIFYING_ENZYMES_203.png]

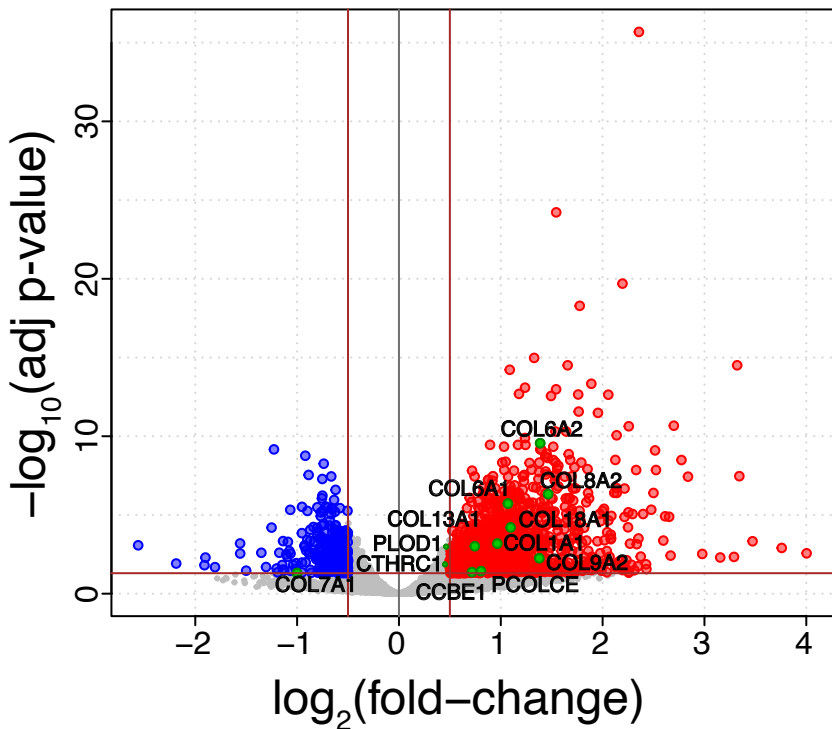

Supplement: Supplementary file 6 — Source data Fig. 1 [file 44319_2024_180_MOESM6_ESM.zip › Figure 1/1B/Volcano Plot - Collagen.pdf]

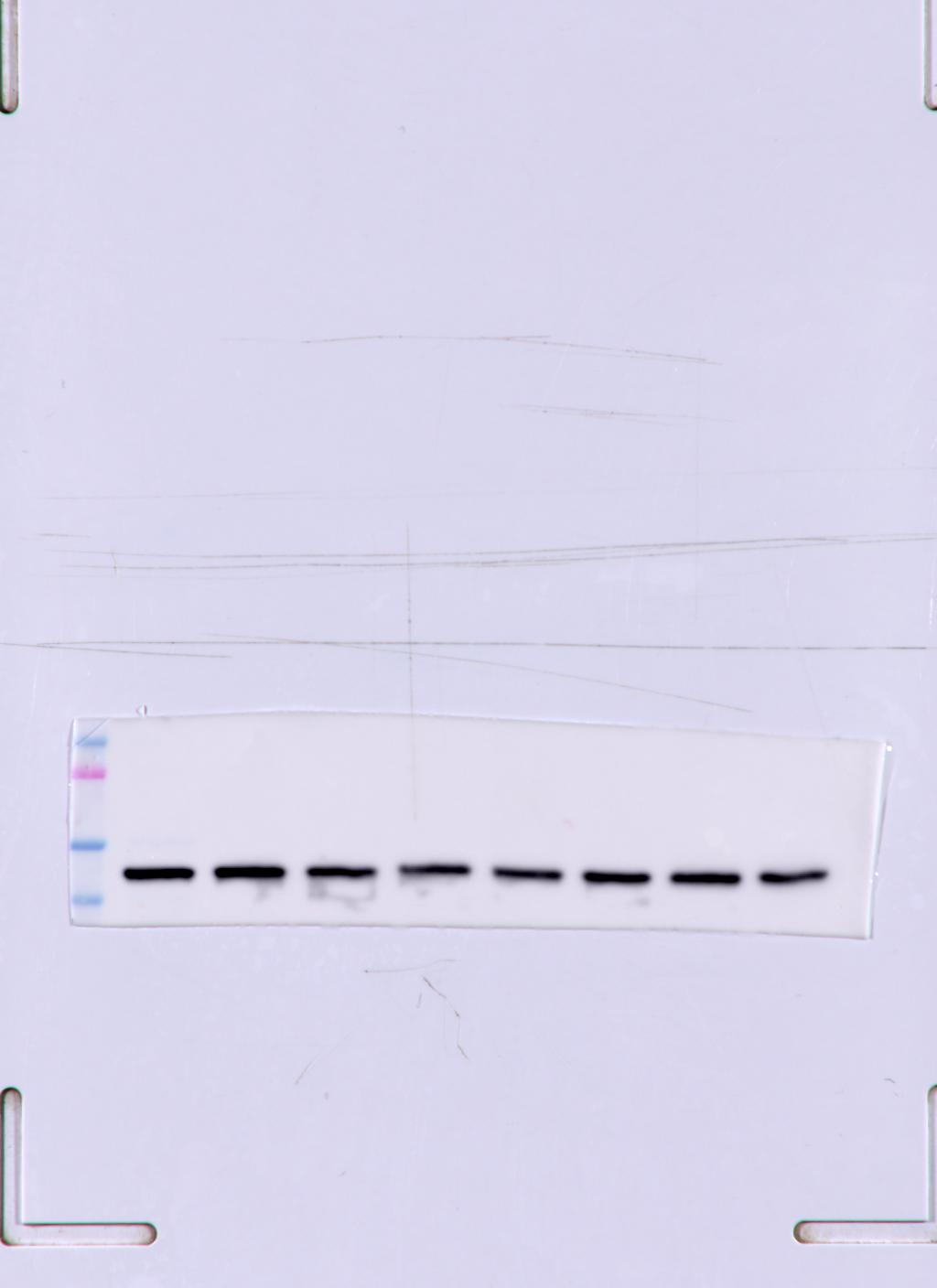

Supplement: Supplementary file 6 — Source data Fig. 1 [file 44319_2024_180_MOESM6_ESM.zip › Figure 1/1E/WB Actin MCT1i.tif]

Figure 1E

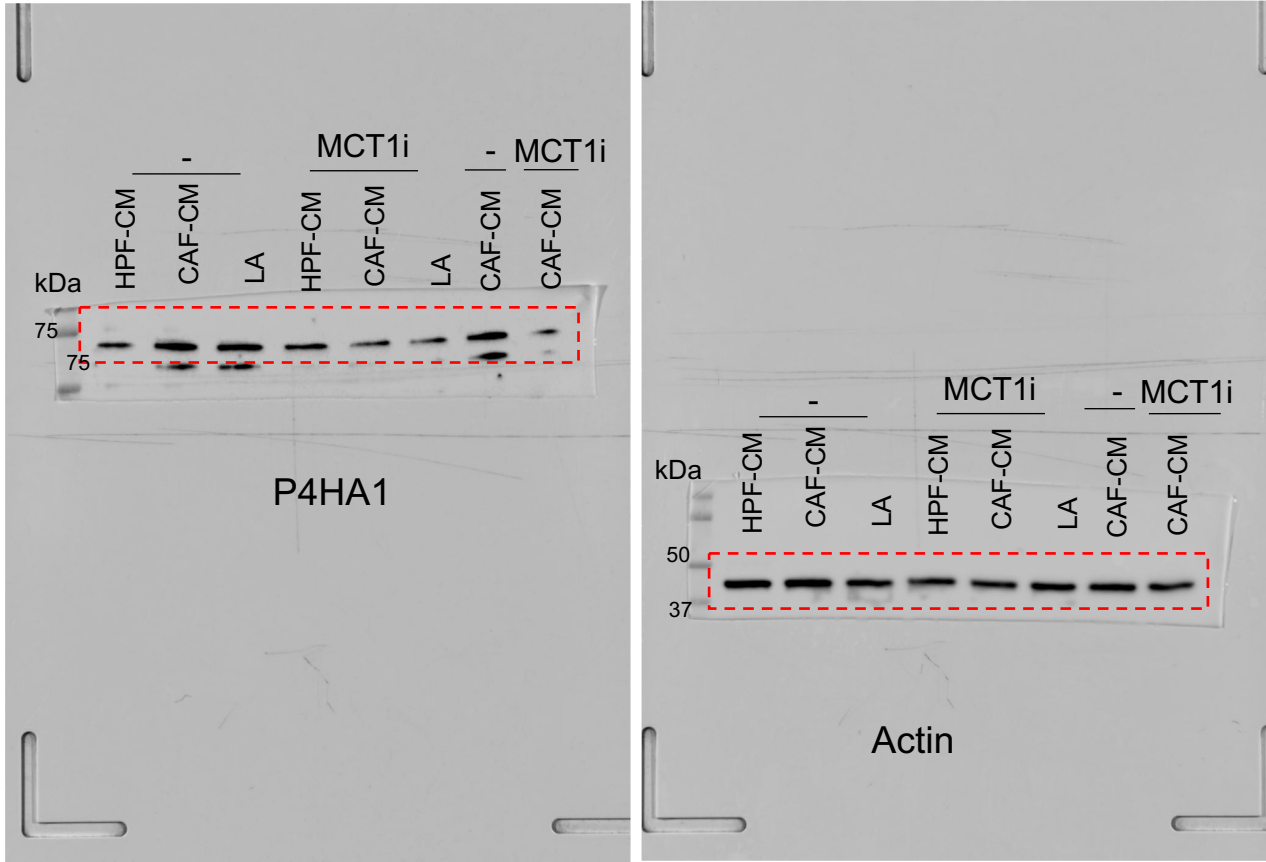

Supplement: Supplementary file 6 — Source data Fig. 1 [file 44319_2024_180_MOESM6_ESM.zip › Figure 1/1E/WB 1E.pdf]

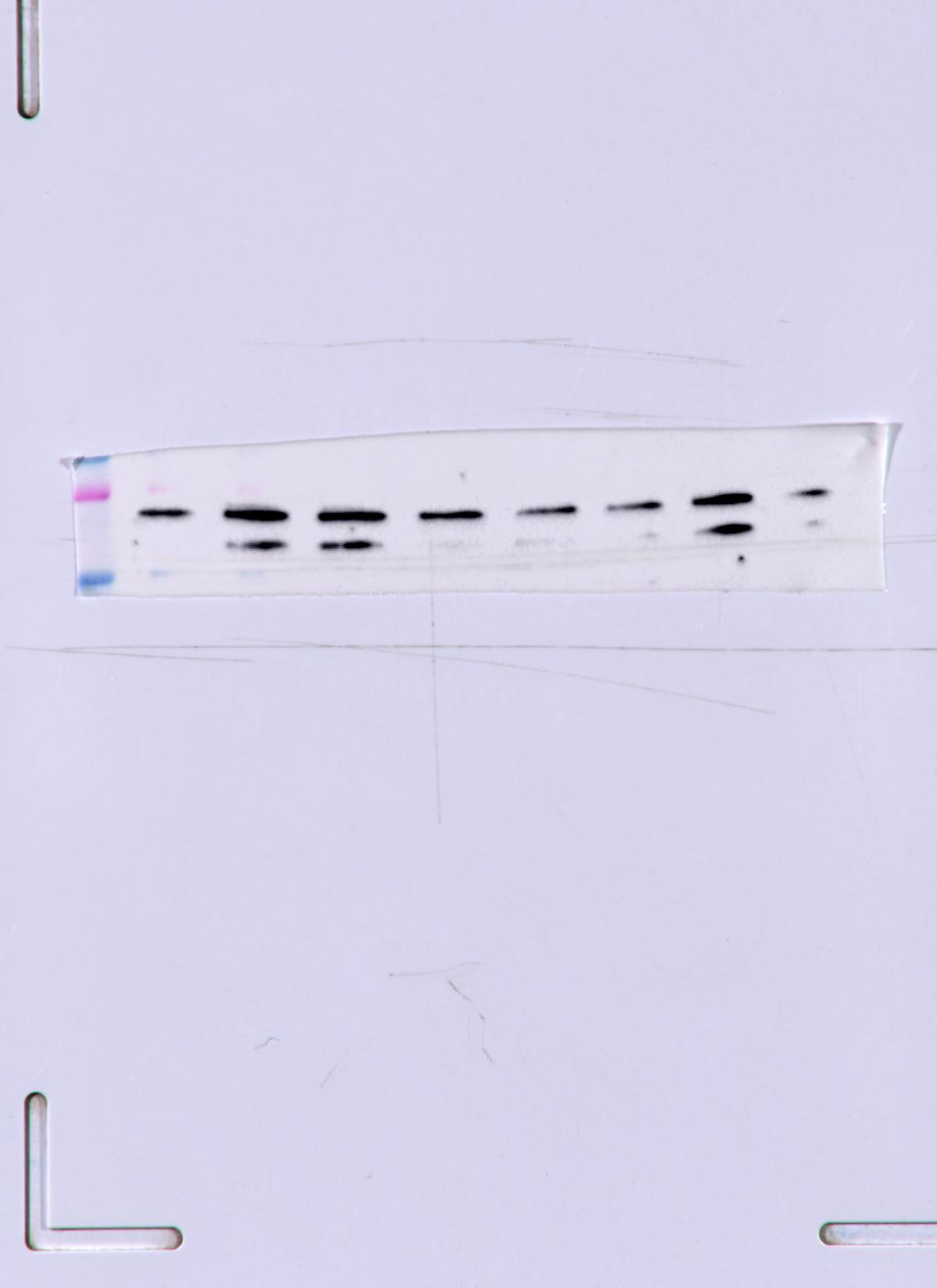

Supplement: Supplementary file 6 — Source data Fig. 1 [file 44319_2024_180_MOESM6_ESM.zip › Figure 1/1E/WB P4HA1_MCT1i.tif]

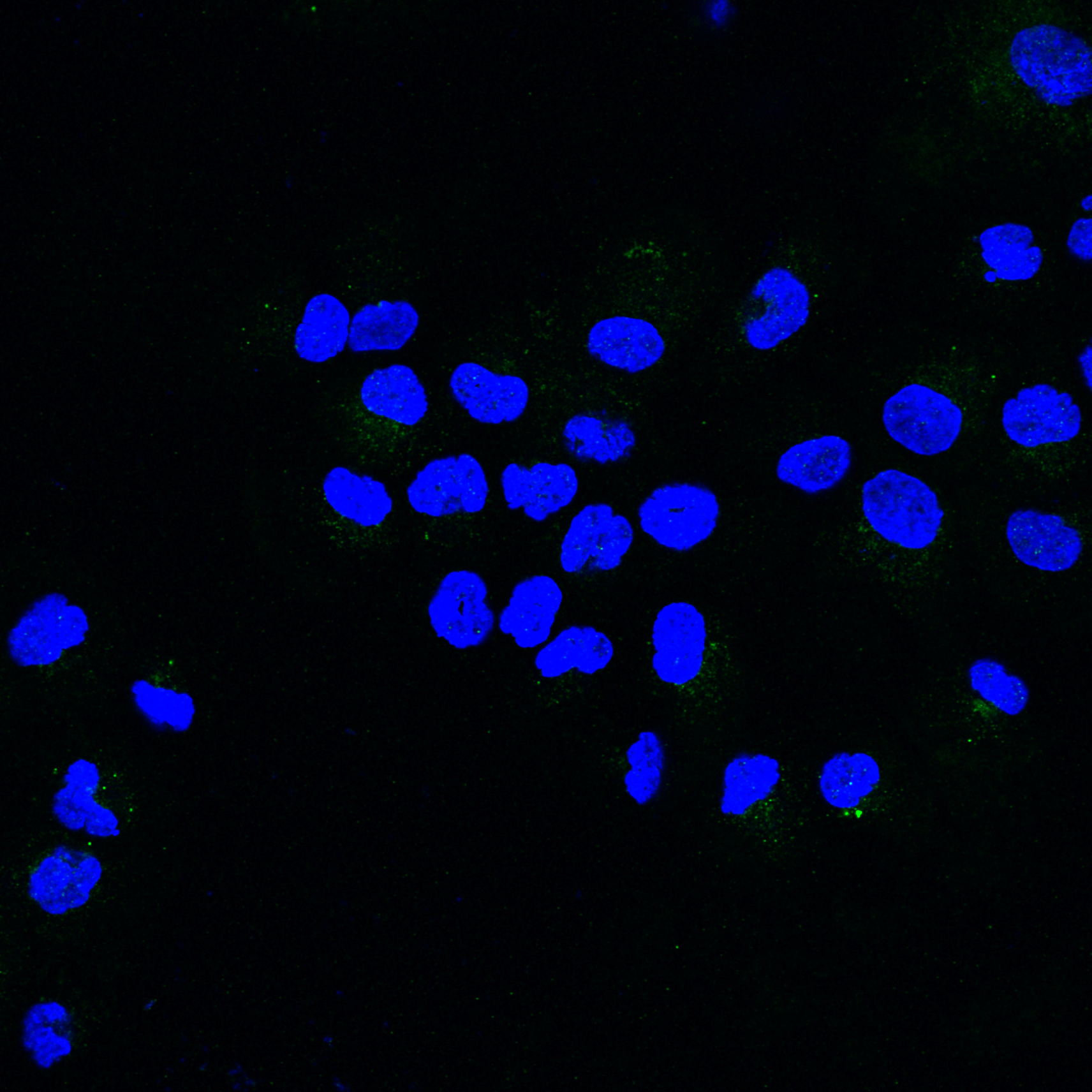

Supplement: Supplementary file 6 — Source data Fig. 1 [file 44319_2024_180_MOESM6_ESM.zip › Figure 1/1C/HPF-CM_MCT1i-1.tif]

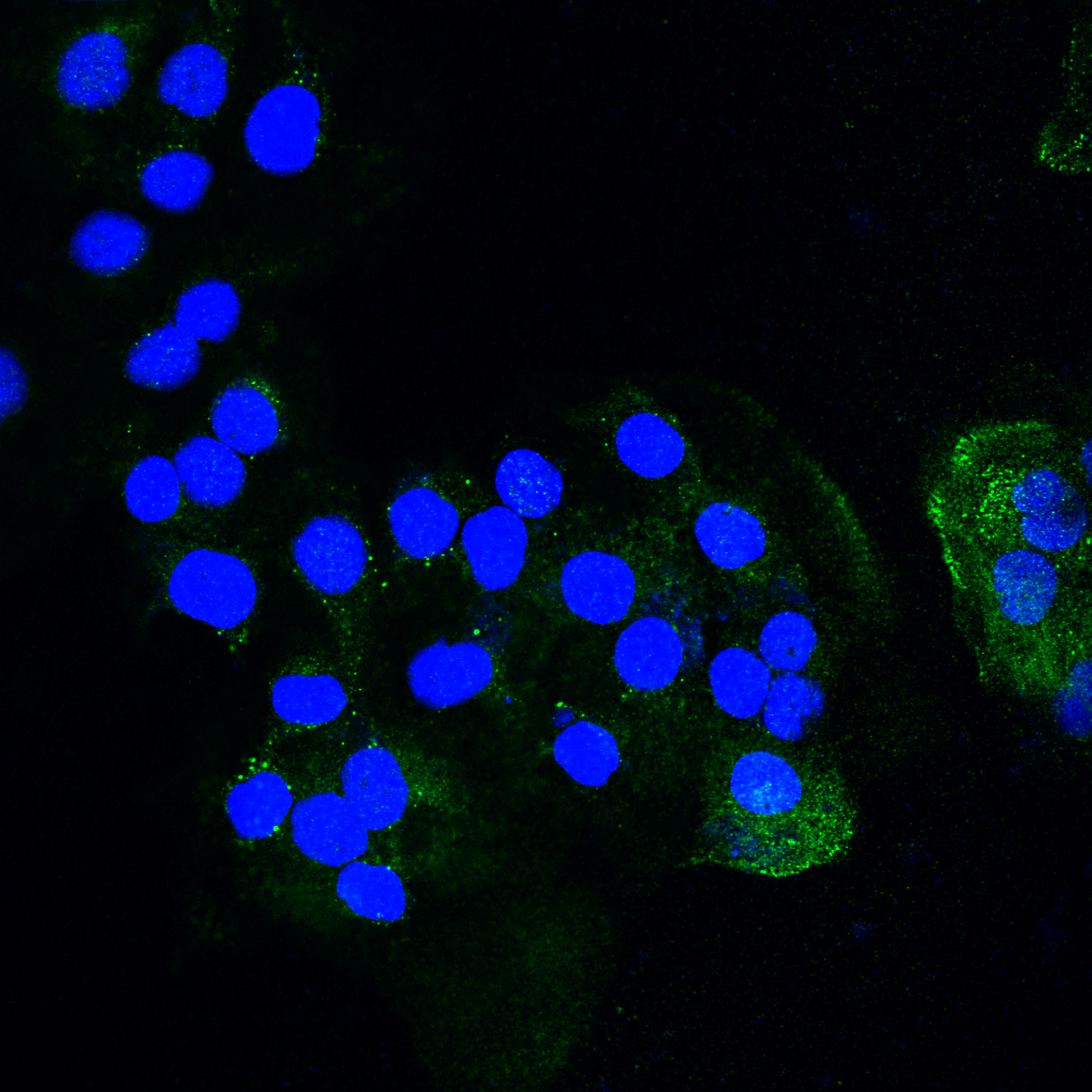

Supplement: Supplementary file 6 — Source data Fig. 1 [file 44319_2024_180_MOESM6_ESM.zip › Figure 1/1C/Lactate.tif]

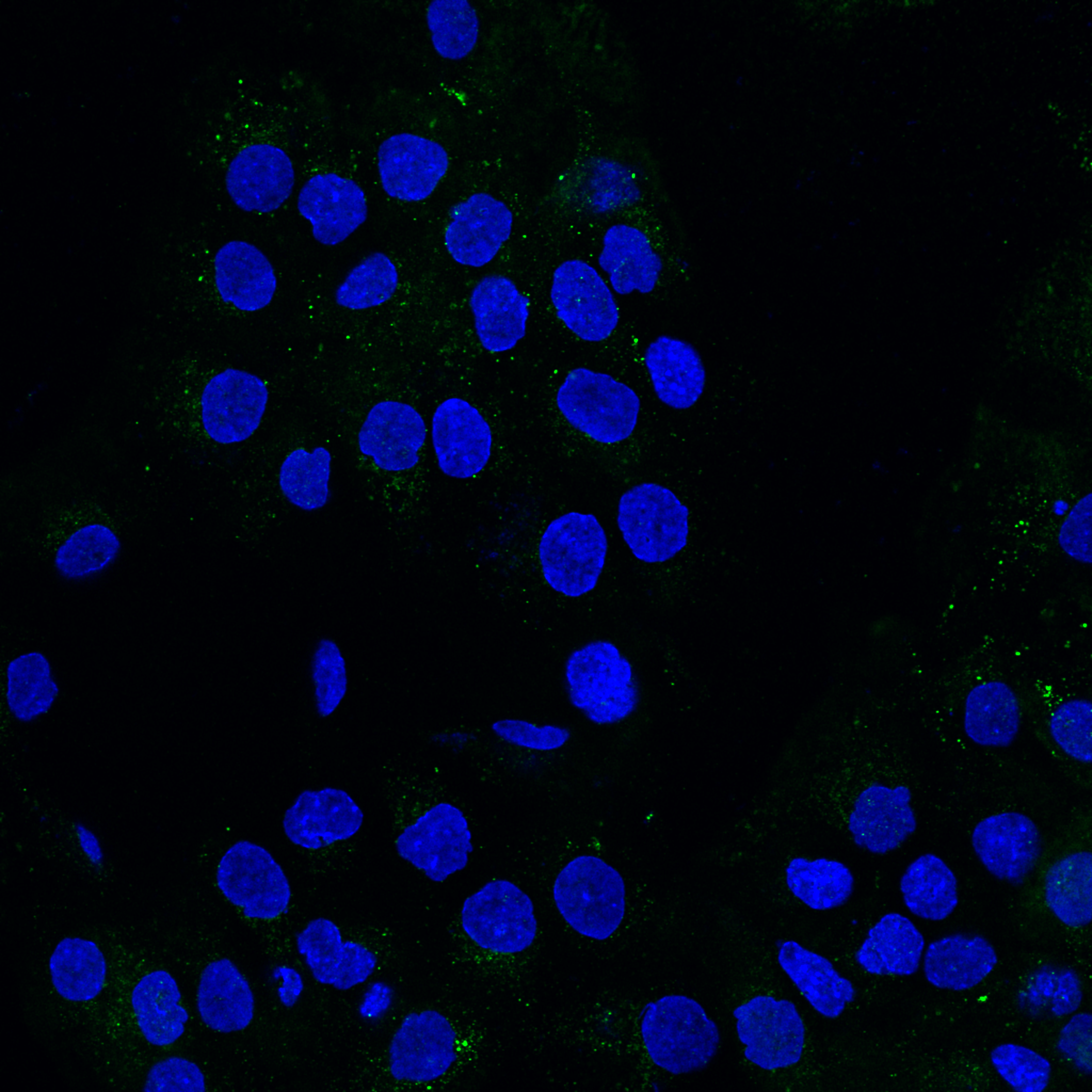

Supplement: Supplementary file 6 — Source data Fig. 1 [file 44319_2024_180_MOESM6_ESM.zip › Figure 1/1C/Lactate_MCT1i-1.tif]

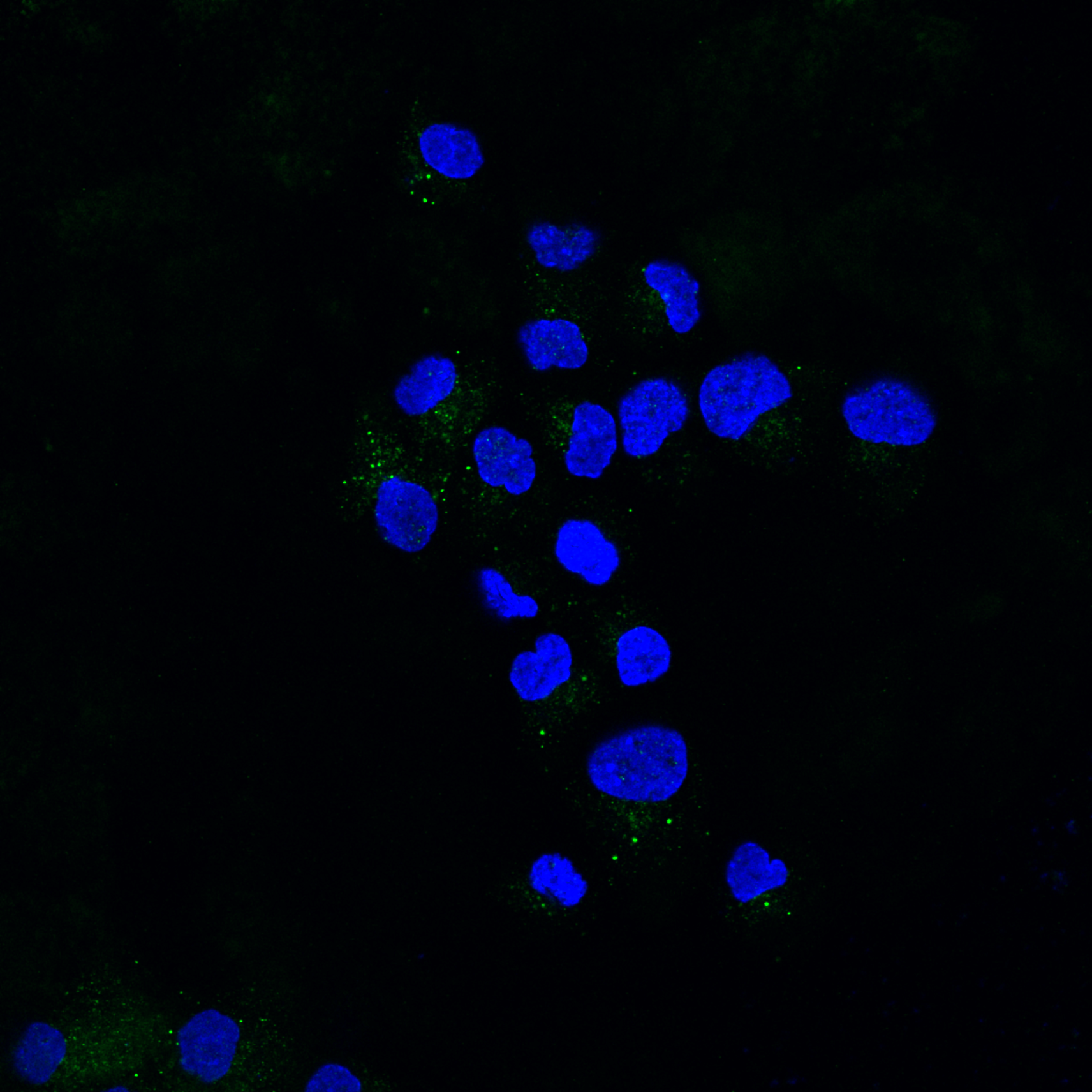

Supplement: Supplementary file 6 — Source data Fig. 1 [file 44319_2024_180_MOESM6_ESM.zip › Figure 1/1C/HPF-CM-1.tif]

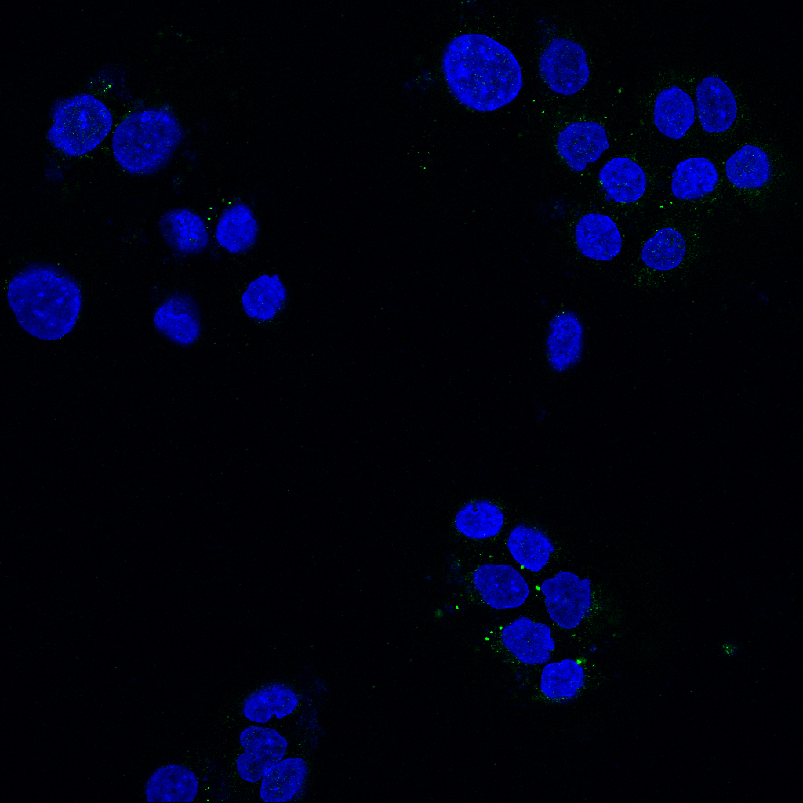

Supplement: Supplementary file 6 — Source data Fig. 1 [file 44319_2024_180_MOESM6_ESM.zip › Figure 1/1C/CAF-CM_MCT1i-1.tif]

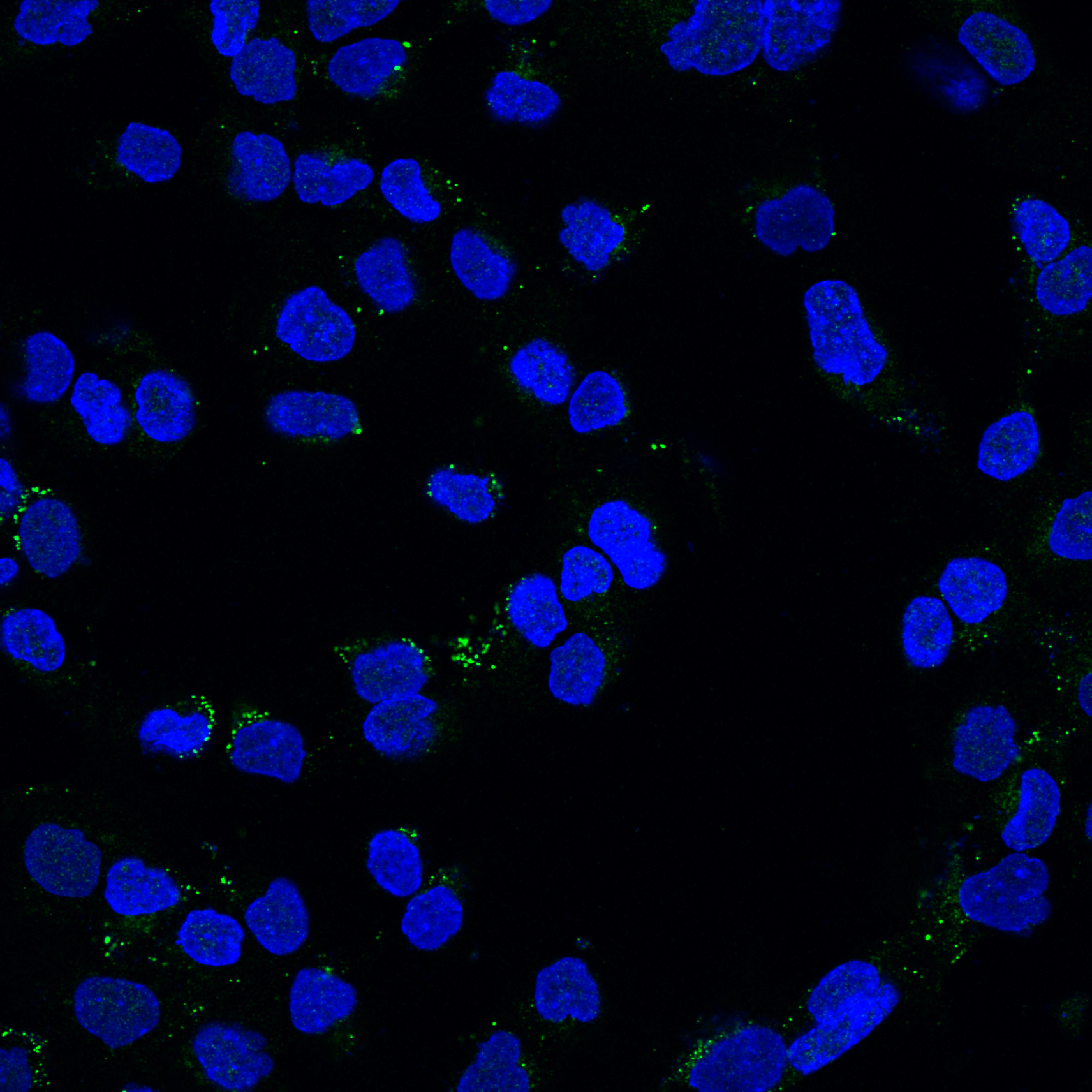

Supplement: Supplementary file 6 — Source data Fig. 1 [file 44319_2024_180_MOESM6_ESM.zip › Figure 1/1C/CAF-CM.tif]

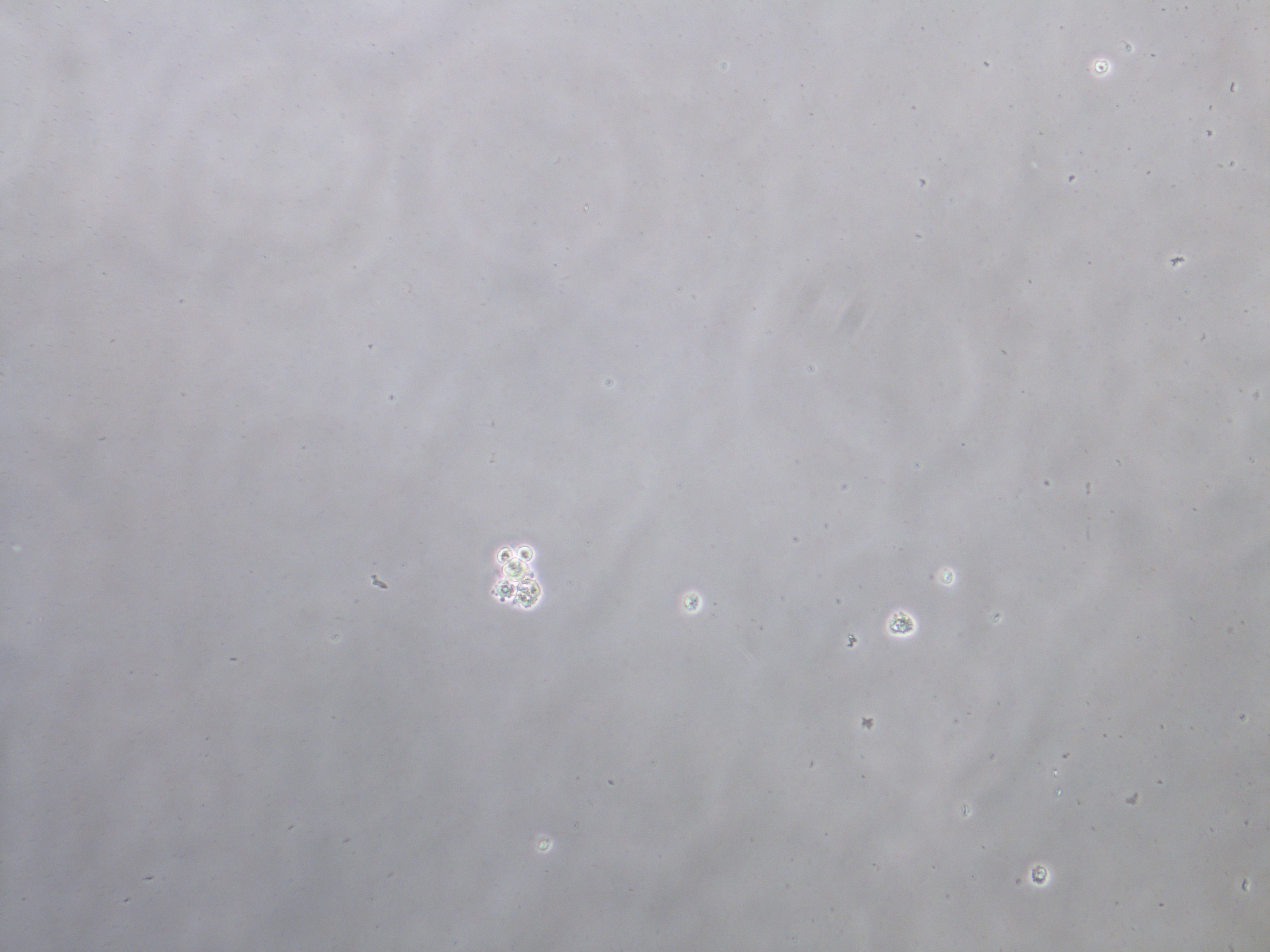

Supplement: Supplementary file 7 — Source data Fig. 2 [file 44319_2024_180_MOESM7_ESM.zip › Figure 2/2C/siP4HA1_Lactate.tif]

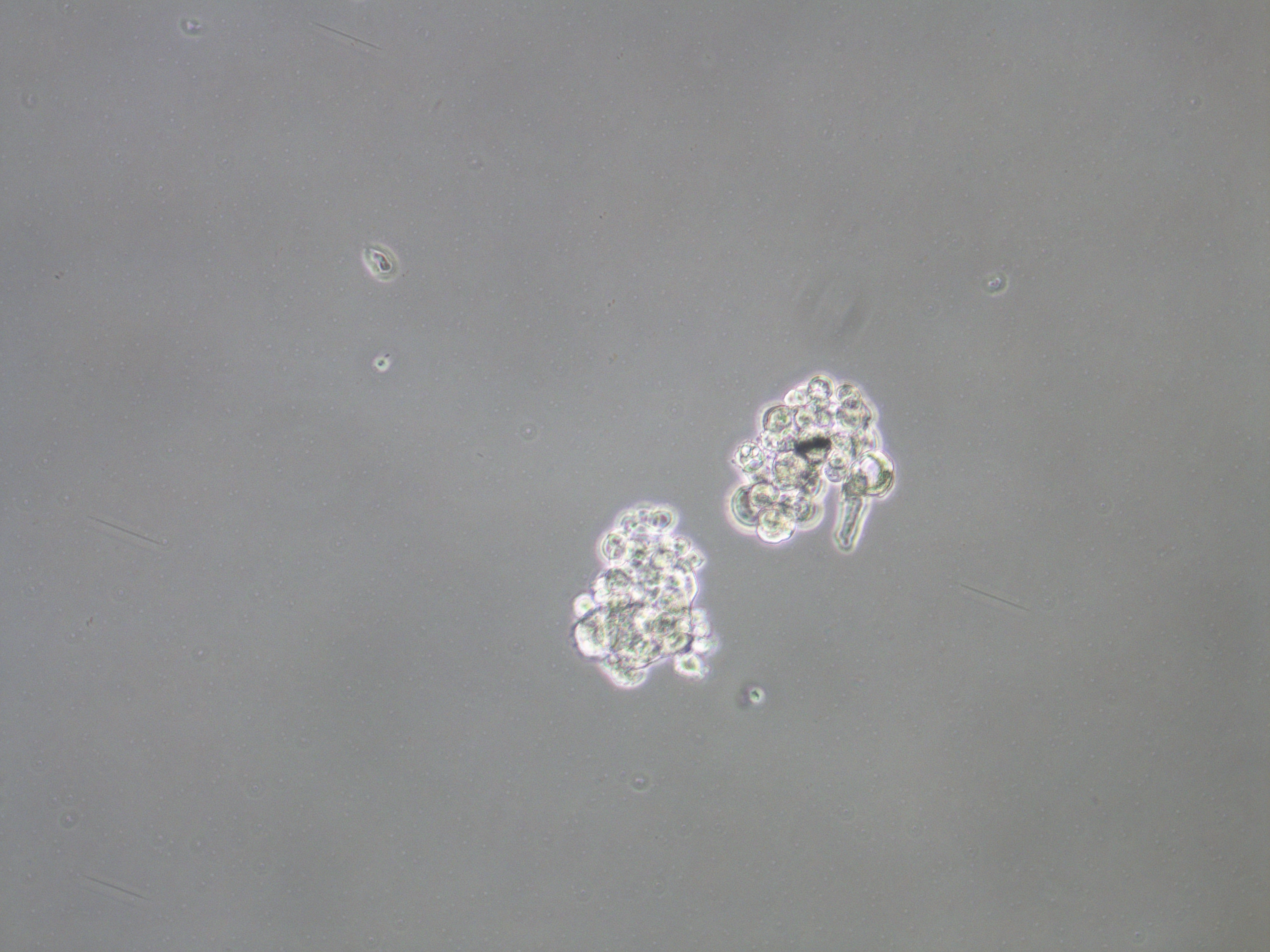

Supplement: Supplementary file 7 — Source data Fig. 2 [file 44319_2024_180_MOESM7_ESM.zip › Figure 2/2C/siCTR_HPF-CM.tif]

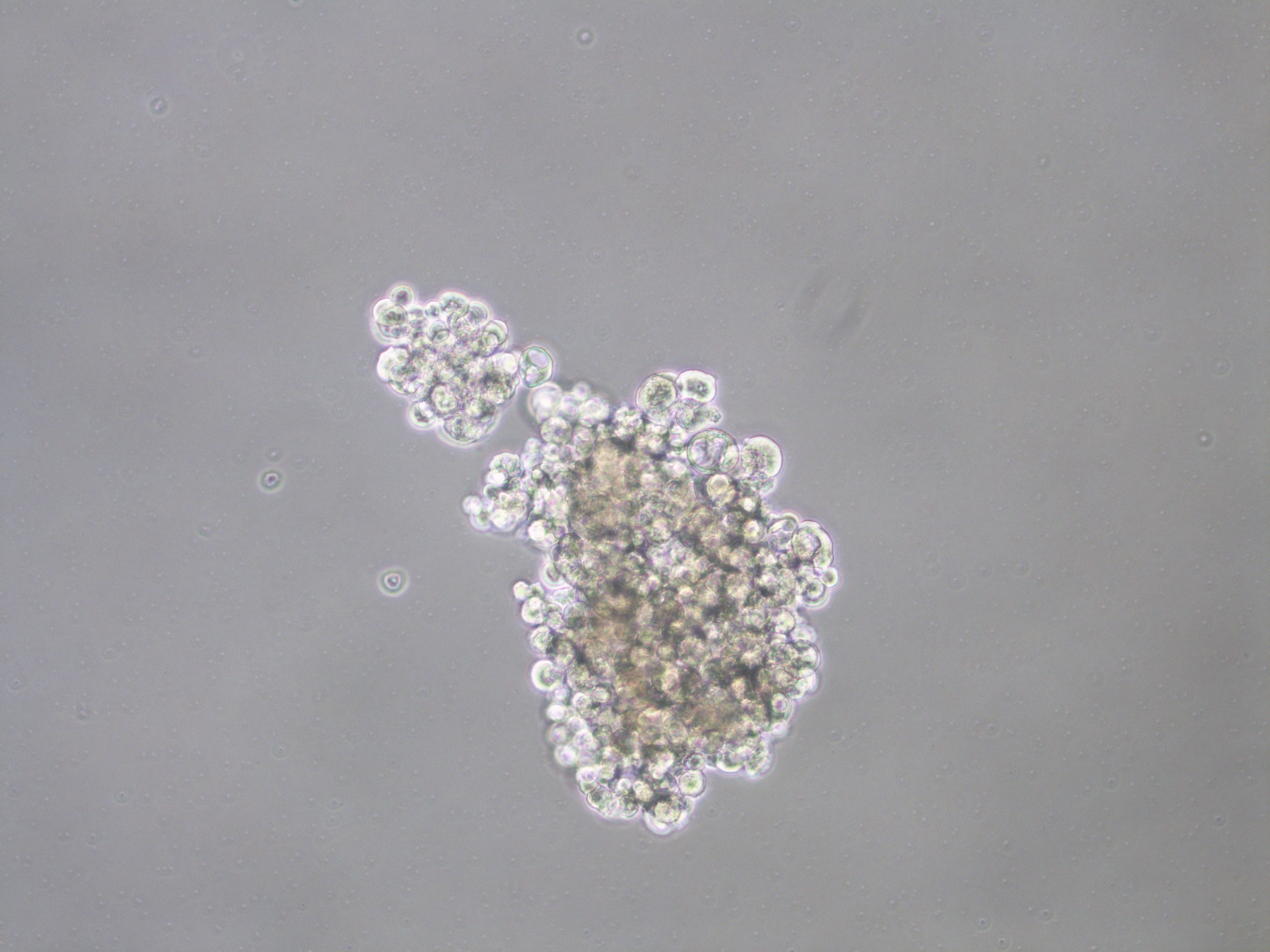

Supplement: Supplementary file 7 — Source data Fig. 2 [file 44319_2024_180_MOESM7_ESM.zip › Figure 2/2C/siCTR_Lactate.tif]

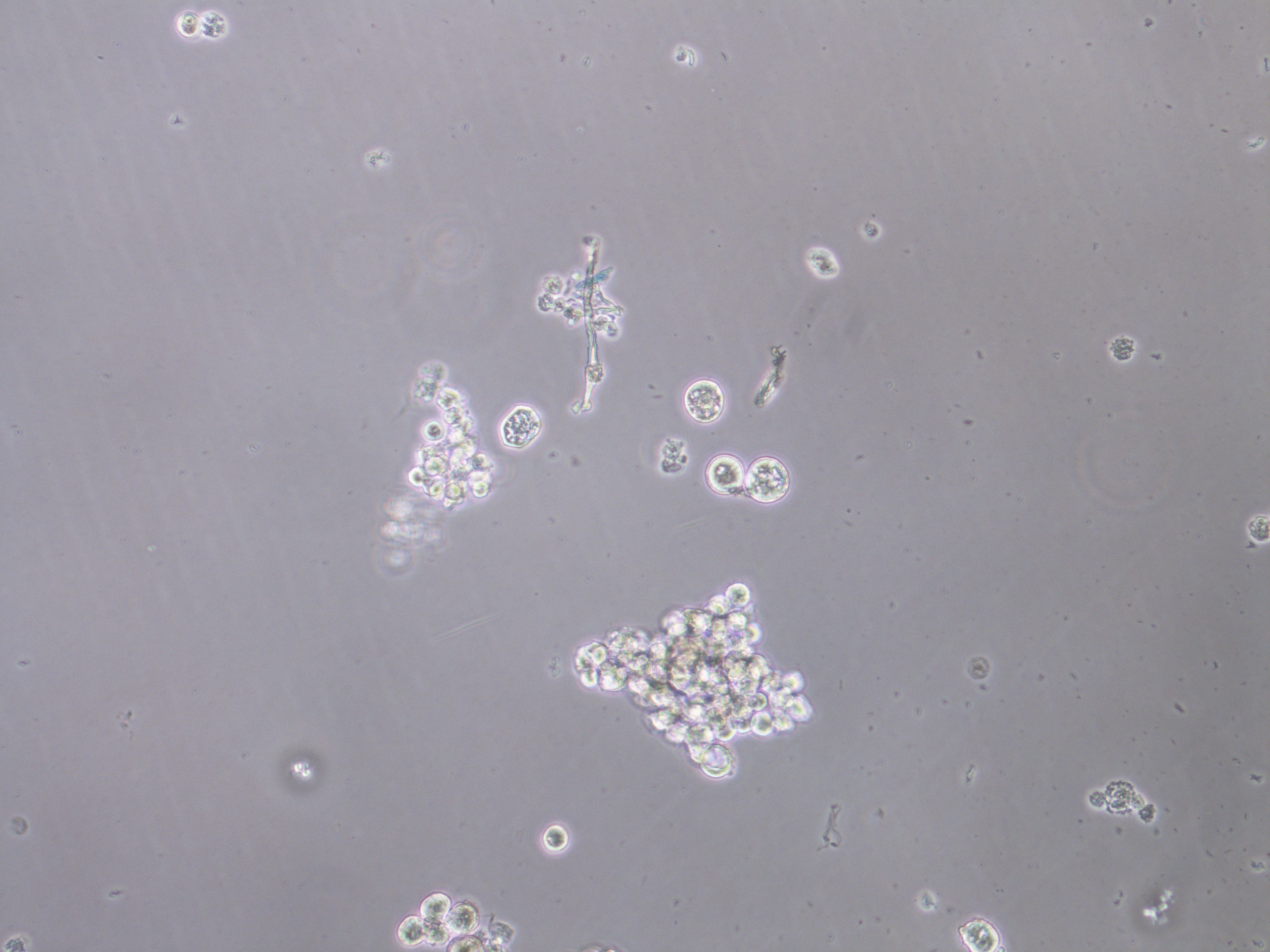

Supplement: Supplementary file 7 — Source data Fig. 2 [file 44319_2024_180_MOESM7_ESM.zip › Figure 2/2C/siP4HA1_HPF-CM.tif]

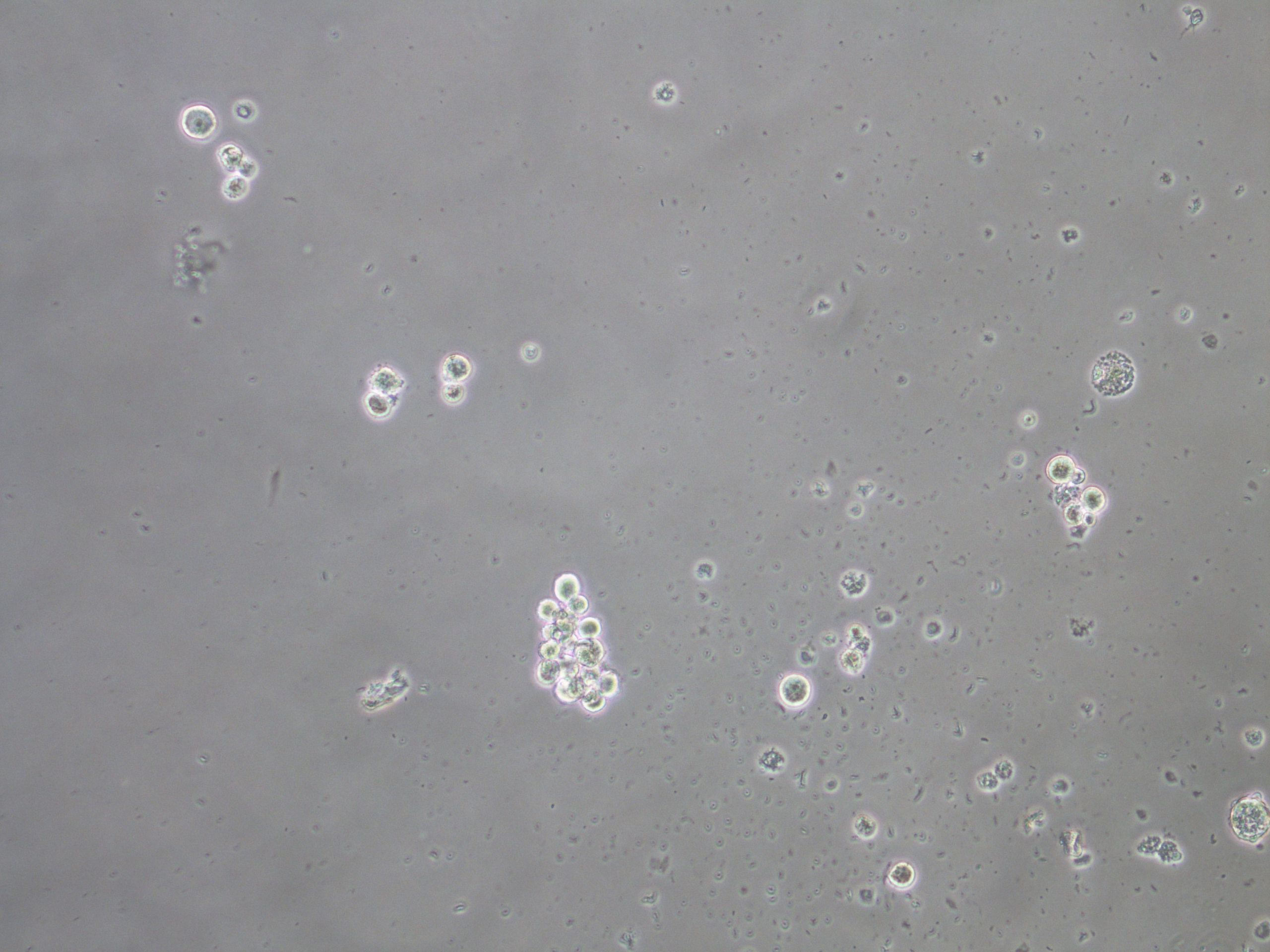

Supplement: Supplementary file 7 — Source data Fig. 2 [file 44319_2024_180_MOESM7_ESM.zip › Figure 2/2C/siP4HA1_CAF-CM.tif]

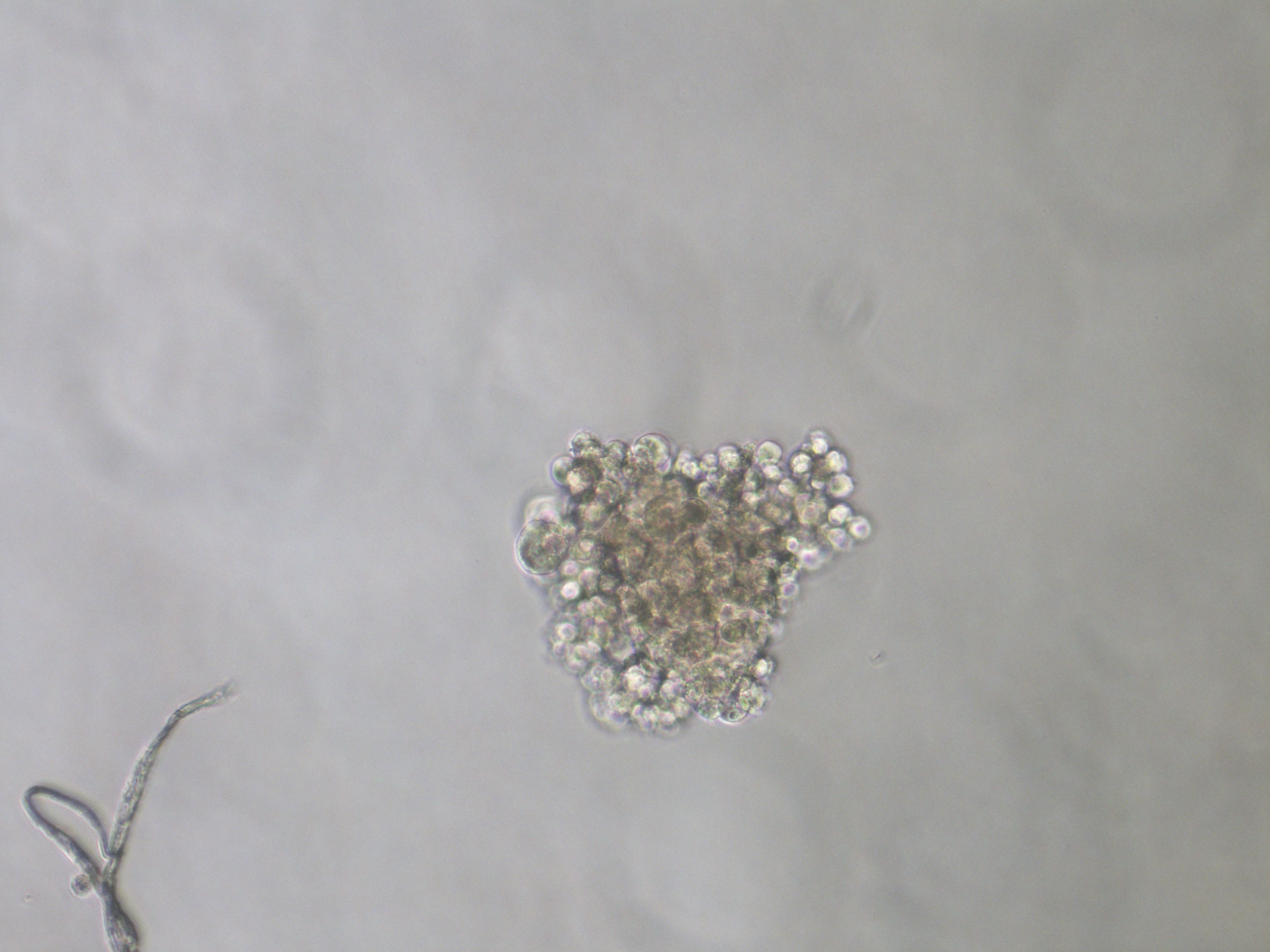

Supplement: Supplementary file 7 — Source data Fig. 2 [file 44319_2024_180_MOESM7_ESM.zip › Figure 2/2C/siCTR_CAF-CM.tif]

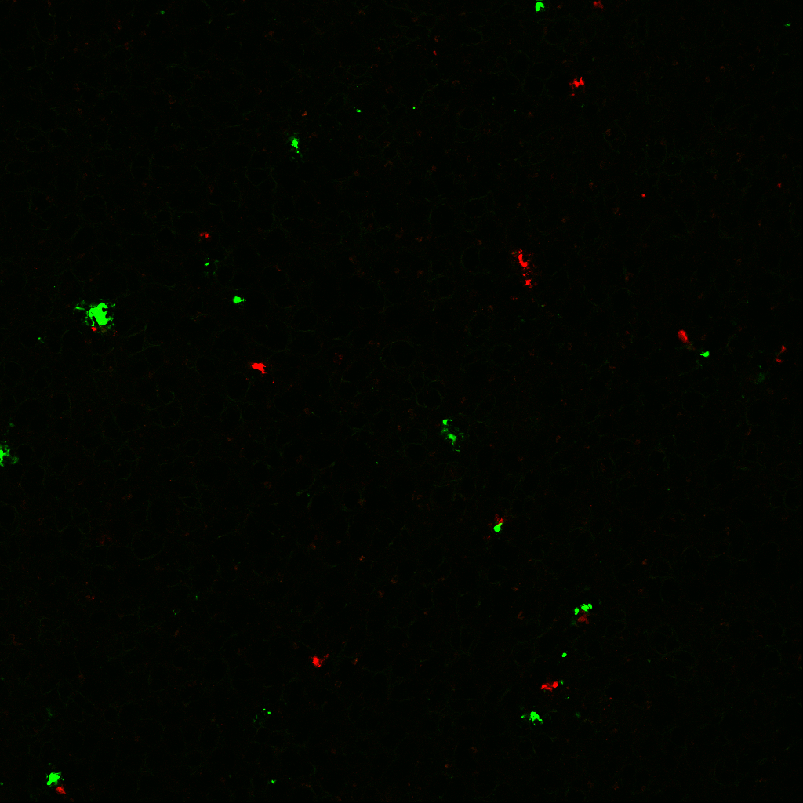

Supplement: Supplementary file 7 — Source data Fig. 2 [file 44319_2024_180_MOESM7_ESM.zip › Figure 2/2D/HPF-CM.tif]

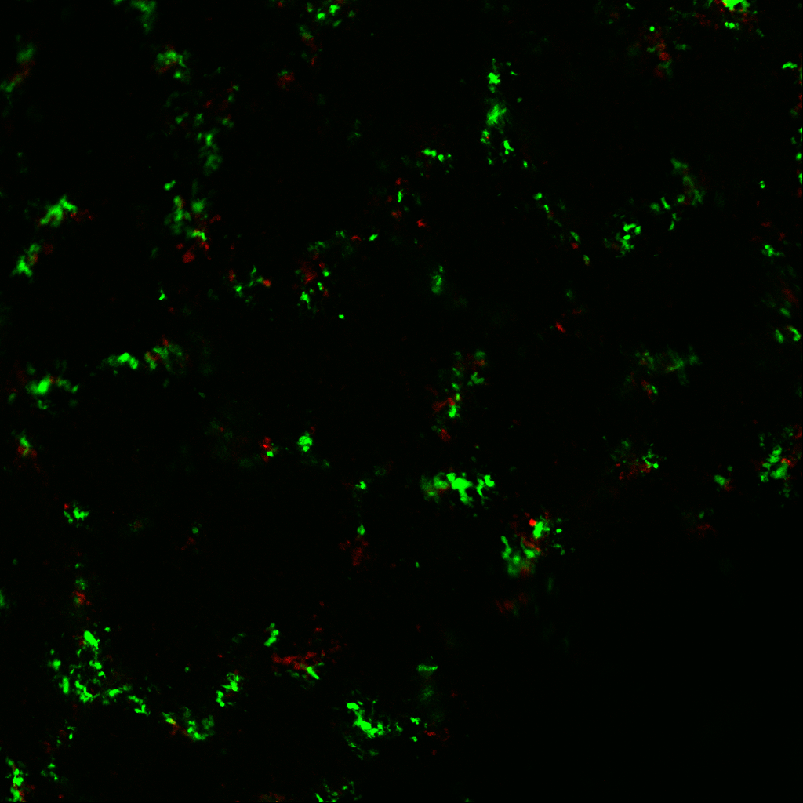

Supplement: Supplementary file 7 — Source data Fig. 2 [file 44319_2024_180_MOESM7_ESM.zip › Figure 2/2D/Lactate.tif]

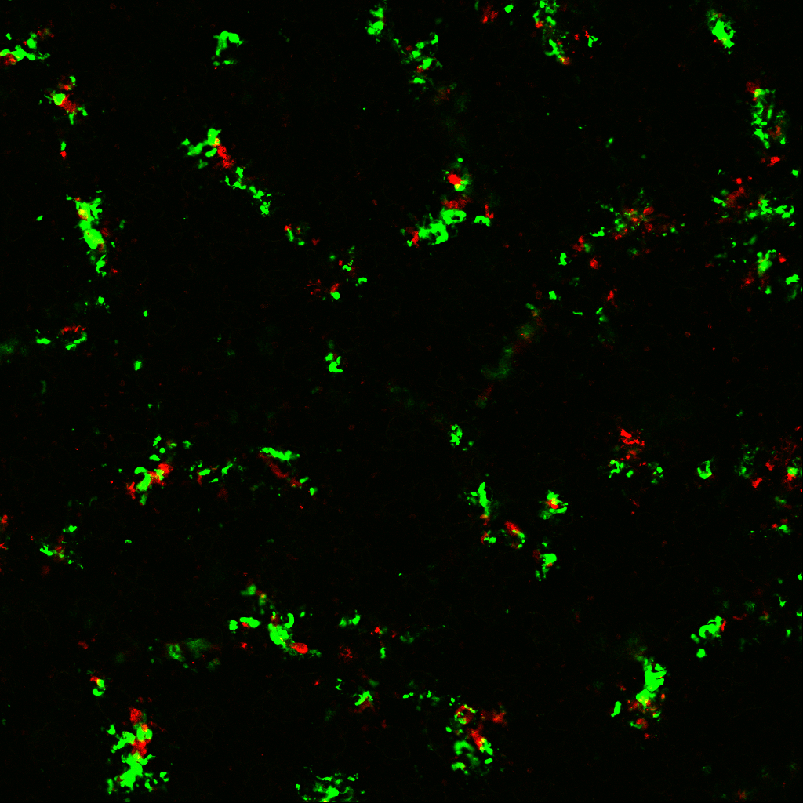

Supplement: Supplementary file 7 — Source data Fig. 2 [file 44319_2024_180_MOESM7_ESM.zip › Figure 2/2D/CAF-CM.tif]

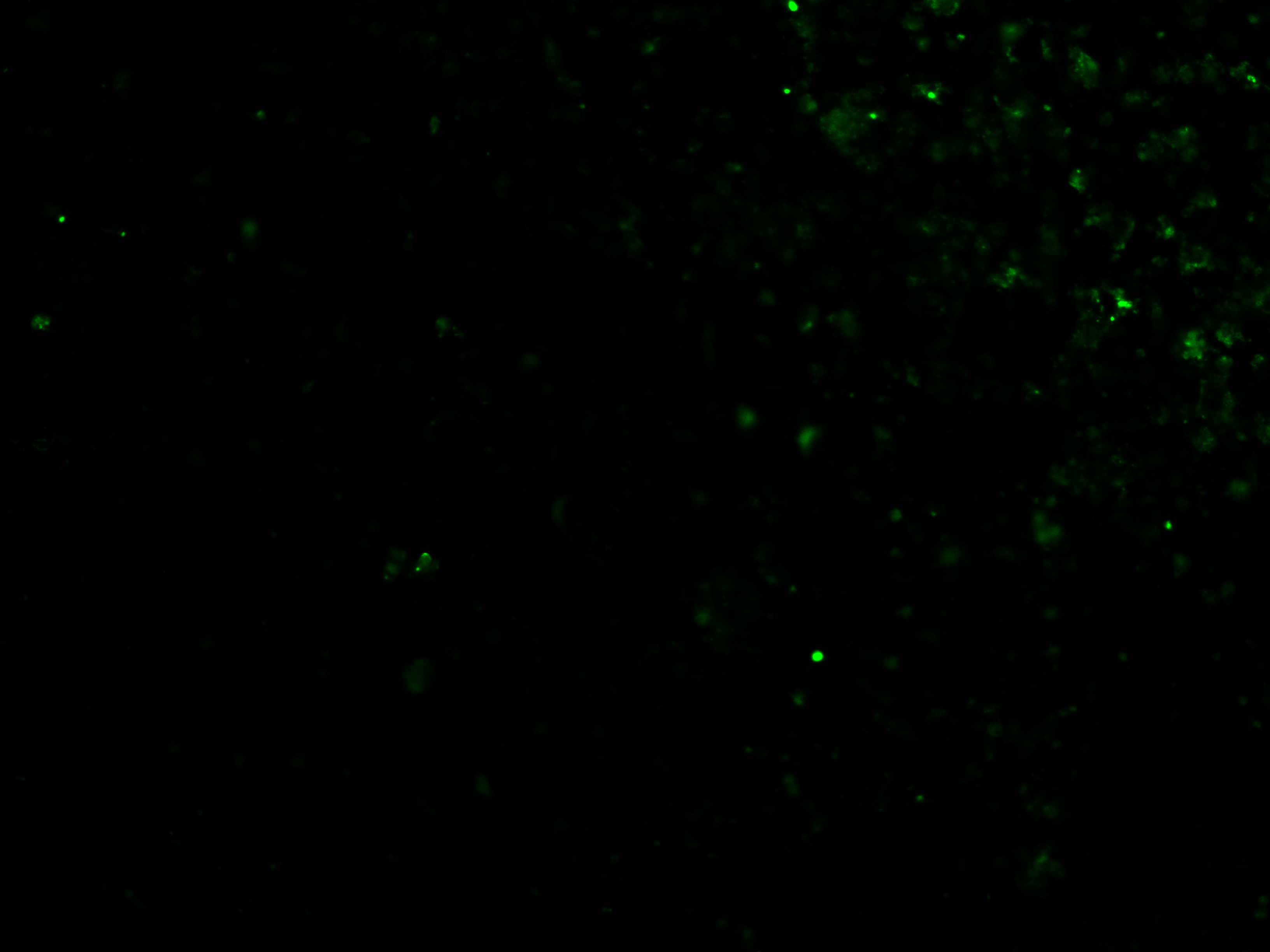

Supplement: Supplementary file 7 — Source data Fig. 2 [file 44319_2024_180_MOESM7_ESM.zip › Figure 2/2B/HPF-CM siP4HA1.tif]

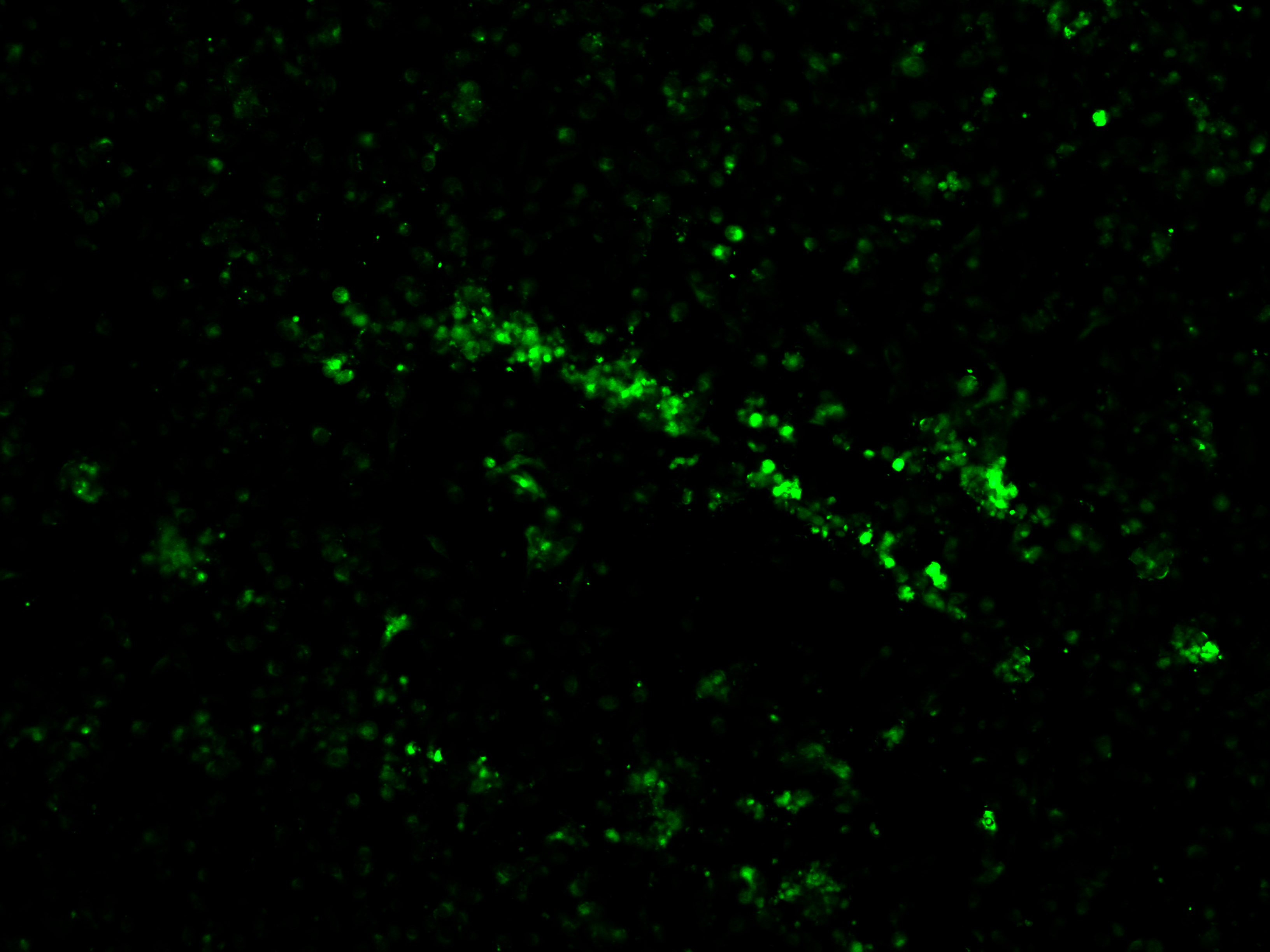

Supplement: Supplementary file 7 — Source data Fig. 2 [file 44319_2024_180_MOESM7_ESM.zip › Figure 2/2B/CAF-CM siCTR.tif]

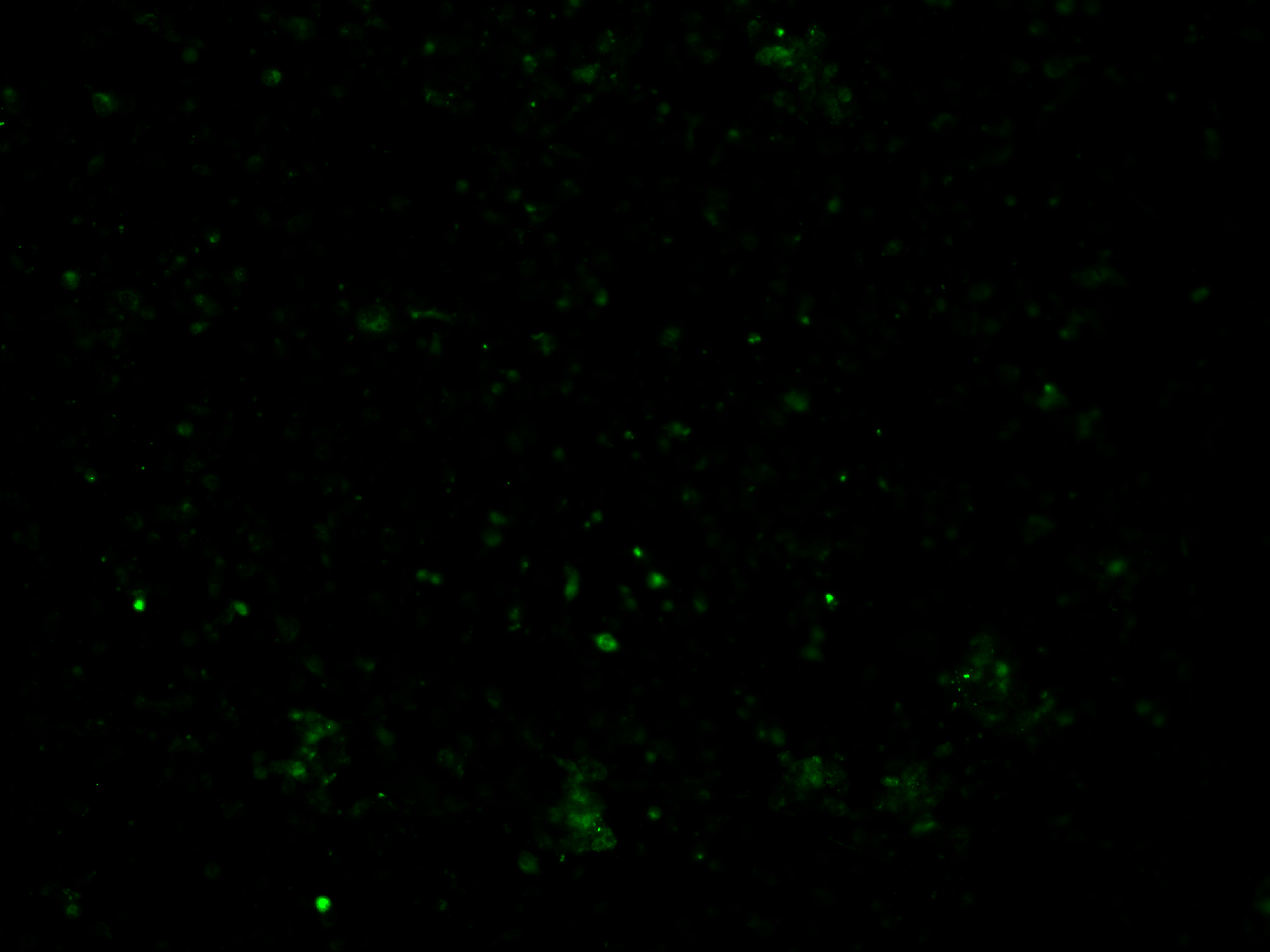

Supplement: Supplementary file 7 — Source data Fig. 2 [file 44319_2024_180_MOESM7_ESM.zip › Figure 2/2B/HPF-CM siCTR.tif]

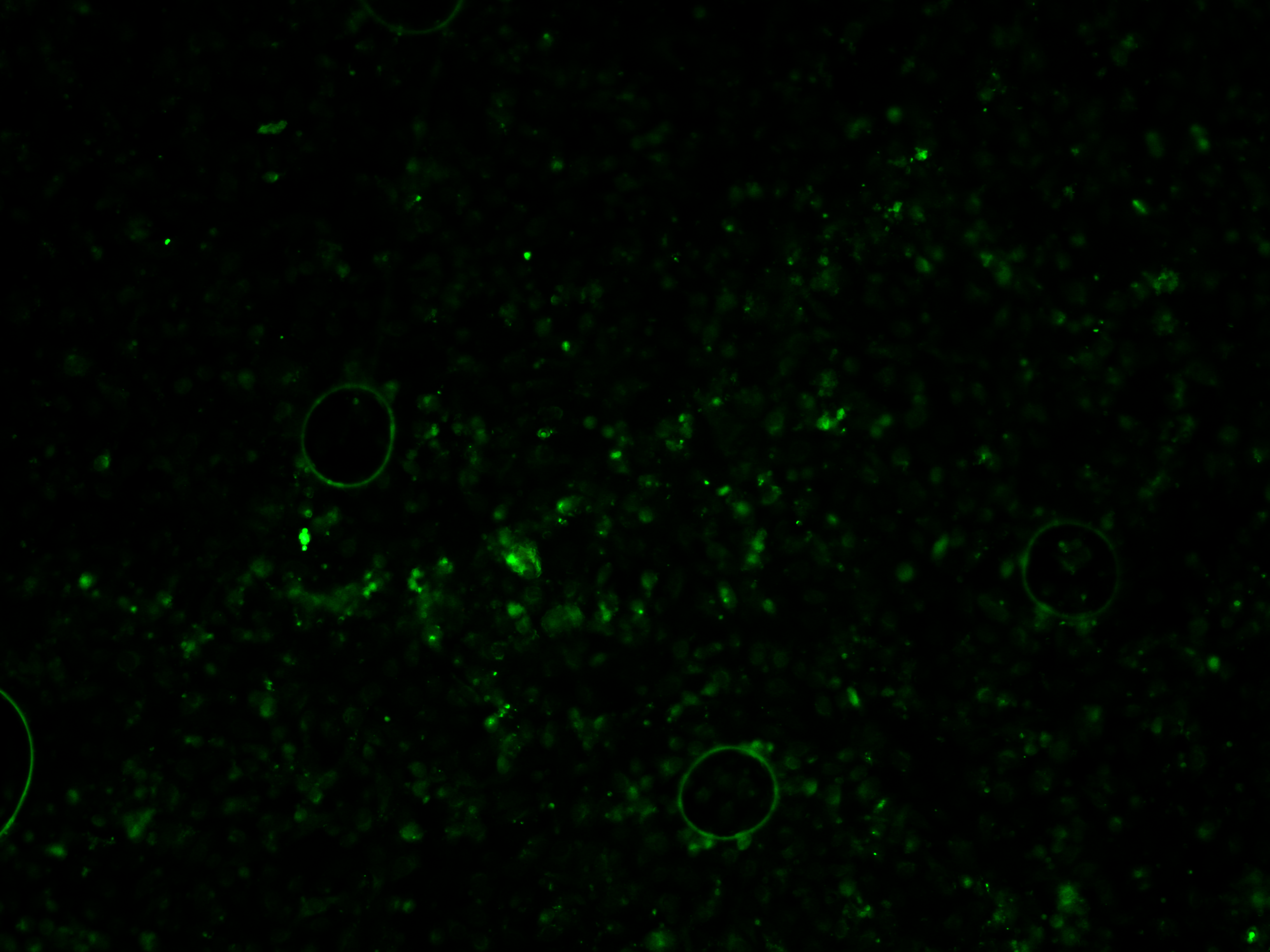

Supplement: Supplementary file 7 — Source data Fig. 2 [file 44319_2024_180_MOESM7_ESM.zip › Figure 2/2B/Lactate siCTR.tif]

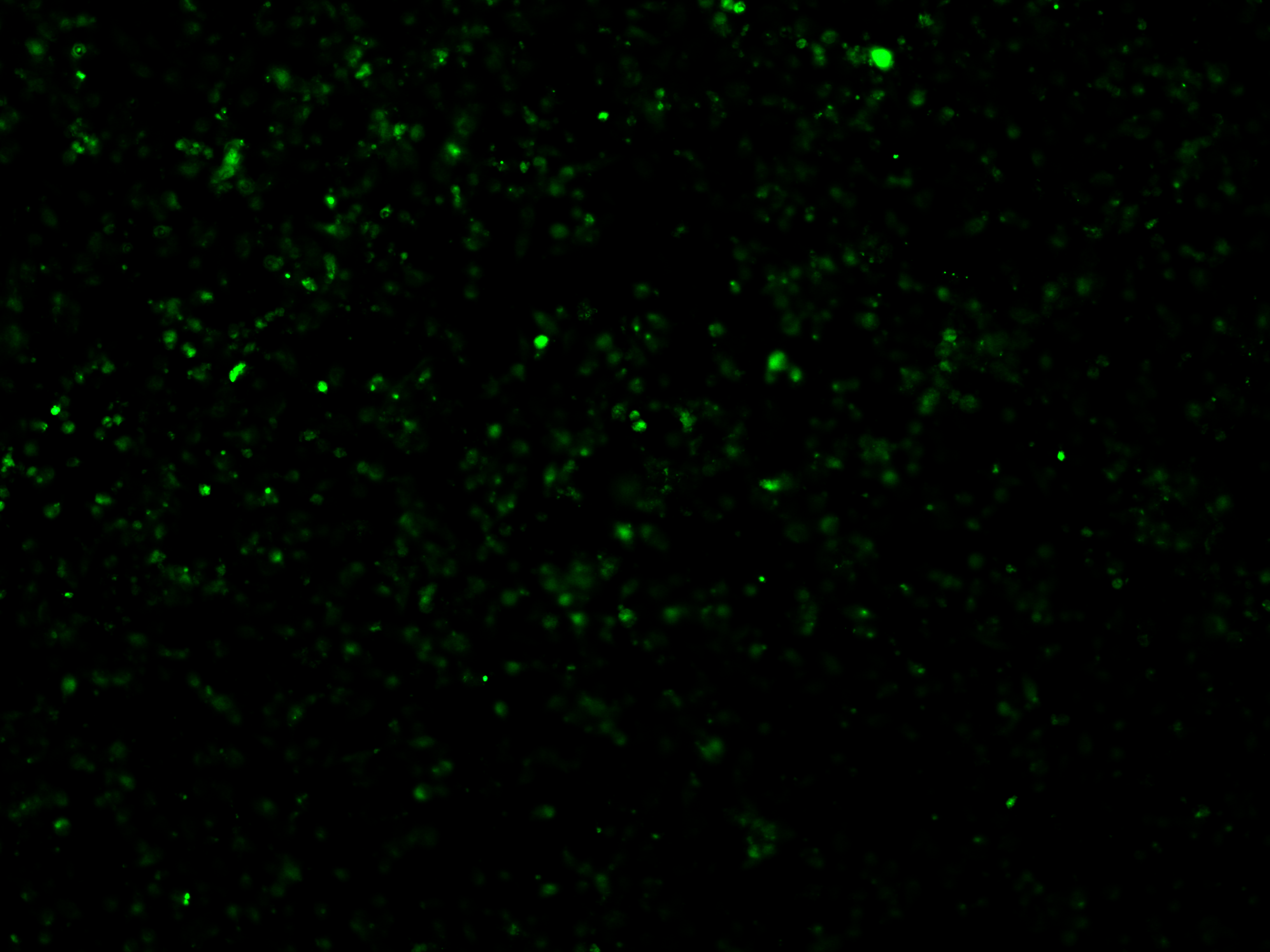

Supplement: Supplementary file 7 — Source data Fig. 2 [file 44319_2024_180_MOESM7_ESM.zip › Figure 2/2B/CAF-CM siP4HA1.tif]

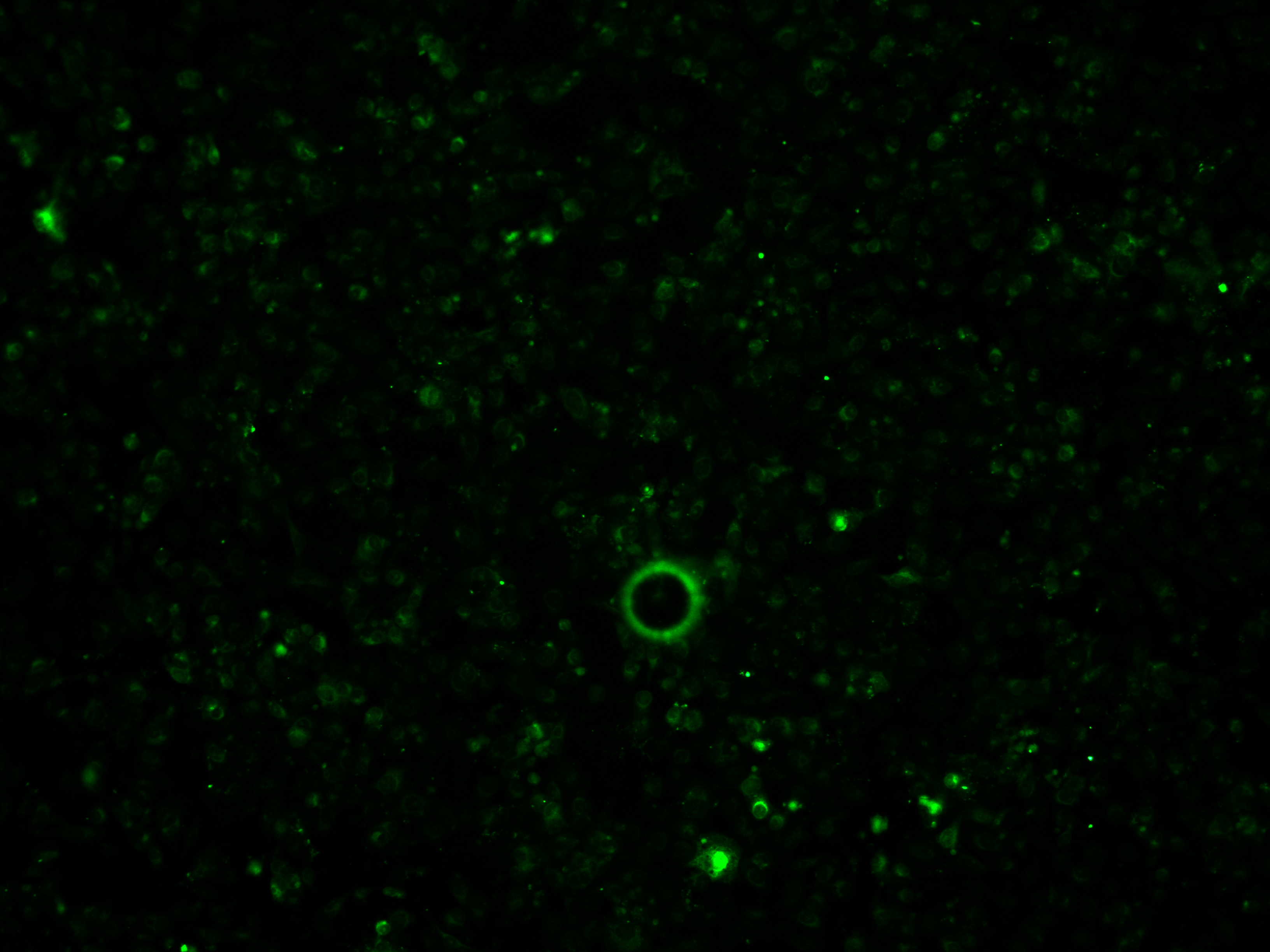

Supplement: Supplementary file 7 — Source data Fig. 2 [file 44319_2024_180_MOESM7_ESM.zip › Figure 2/2B/Lactate siP4HA1.tif]

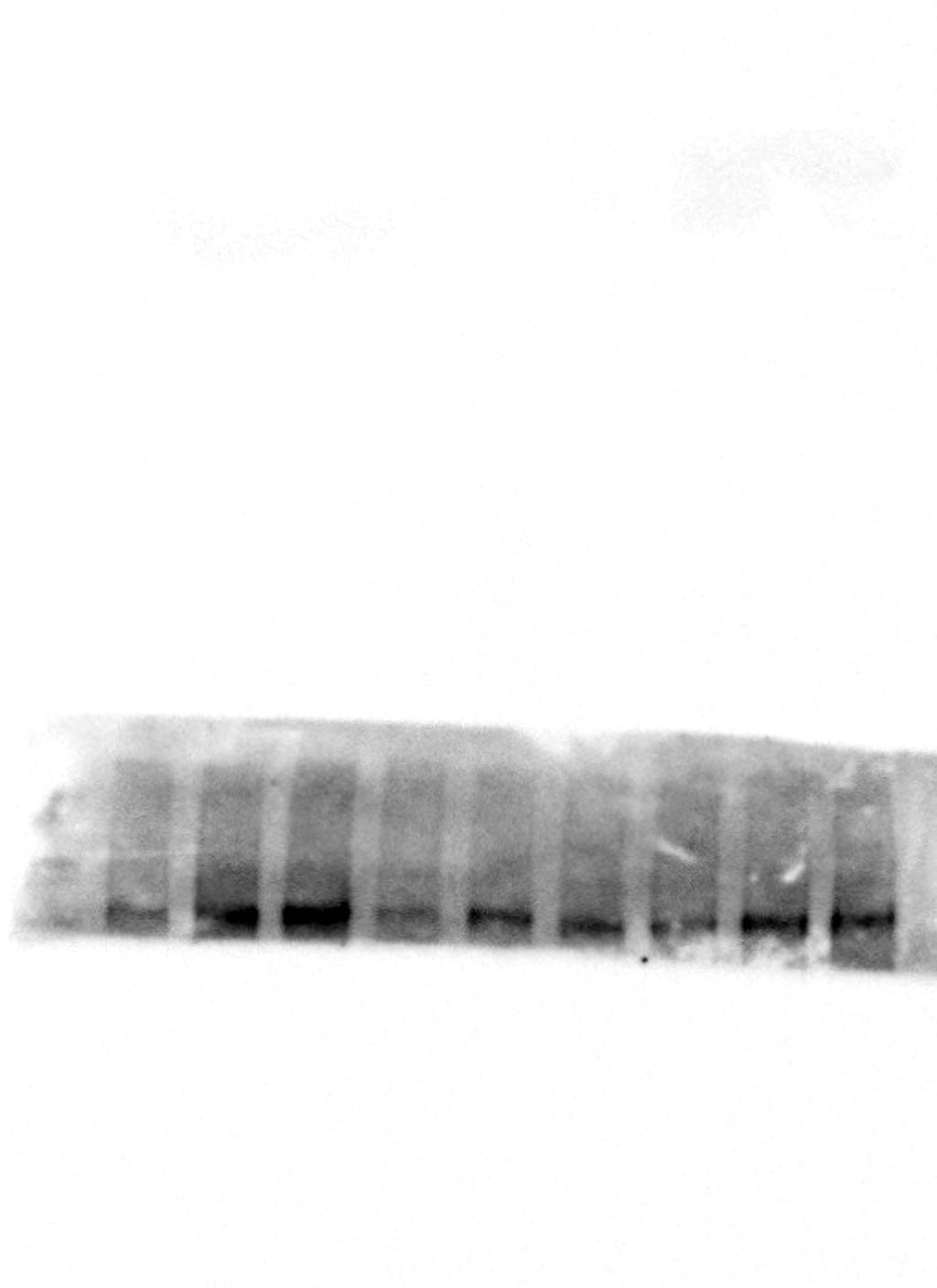

Supplement: Supplementary file 8 — Source data Fig. 3 [file 44319_2024_180_MOESM8_ESM.zip › Figure 3/3B/DDR1 MCT1i.tif]

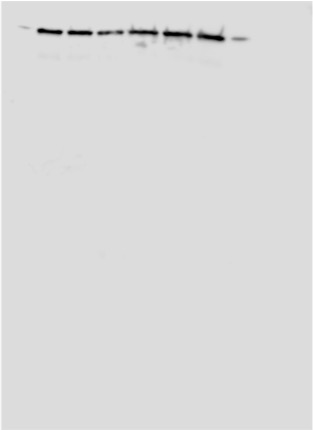

Supplement: Supplementary file 8 — Source data Fig. 3 [file 44319_2024_180_MOESM8_ESM.zip › Figure 3/3B/Actin MCT1i.tif]

Figure 3b

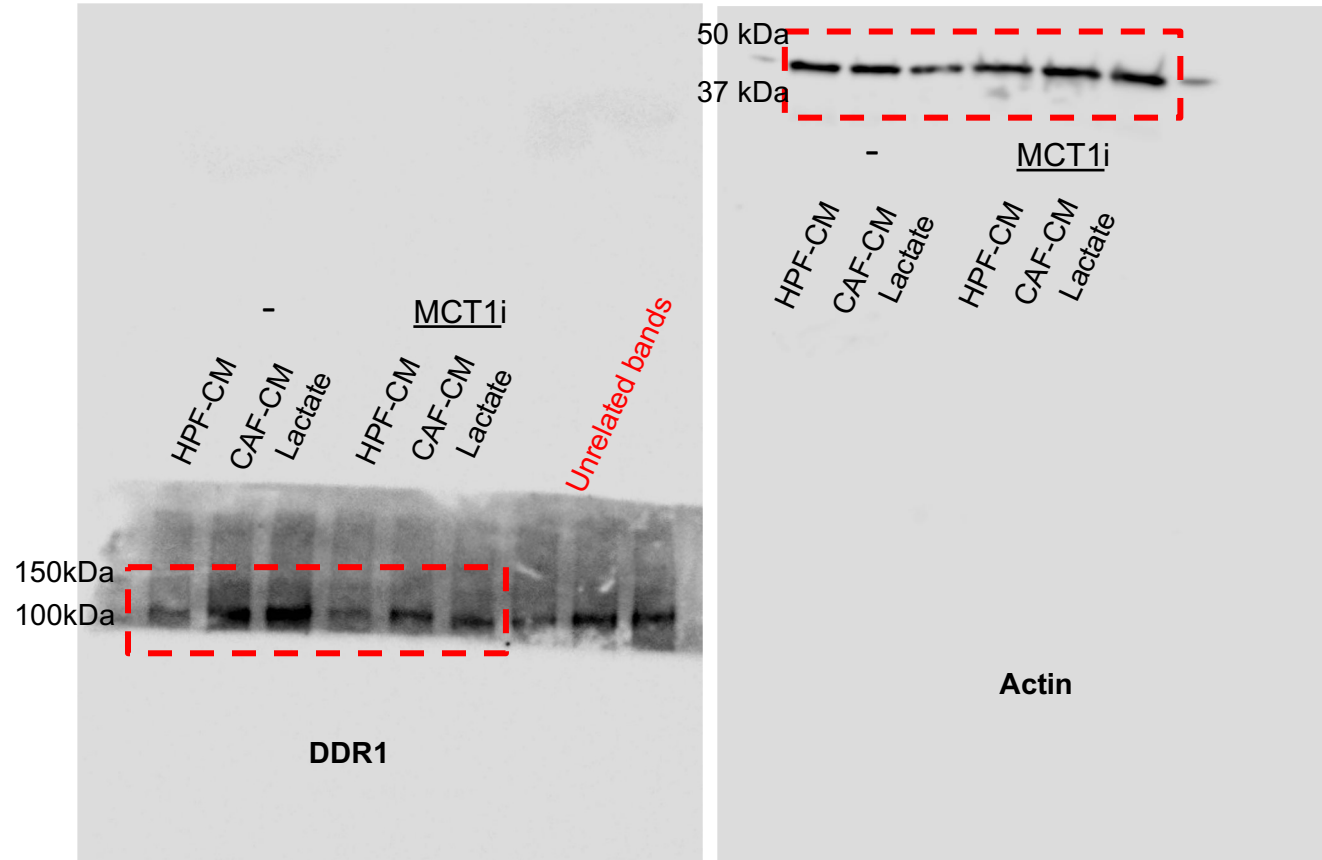

Supplement: Supplementary file 8 — Source data Fig. 3 [file 44319_2024_180_MOESM8_ESM.zip › Figure 3/3B/3b blot.pdf]

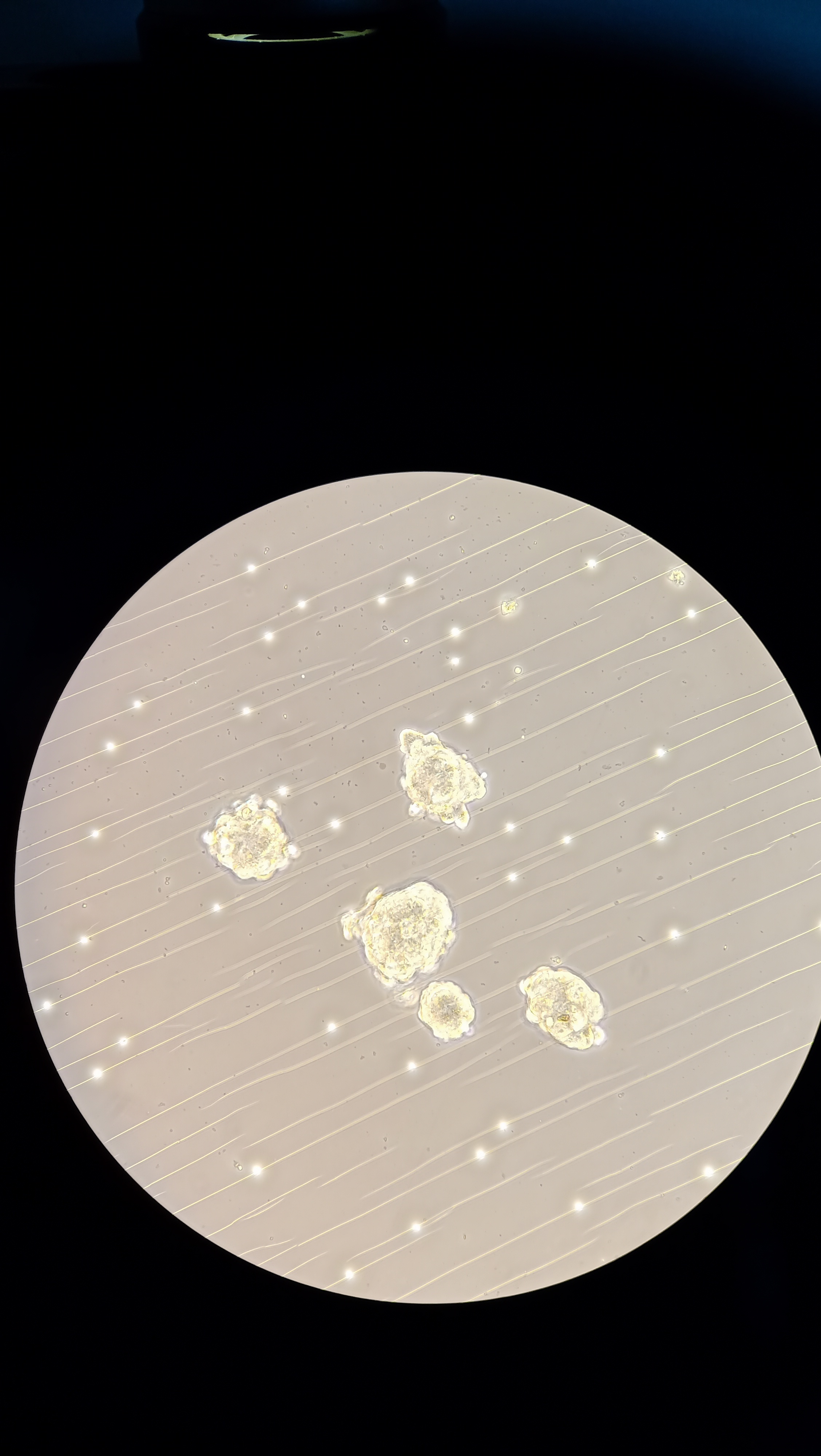

Supplement: Supplementary file 8 — Source data Fig. 3 [file 44319_2024_180_MOESM8_ESM.zip › Figure 3/3D/CAF-CM siDDR1.tif]

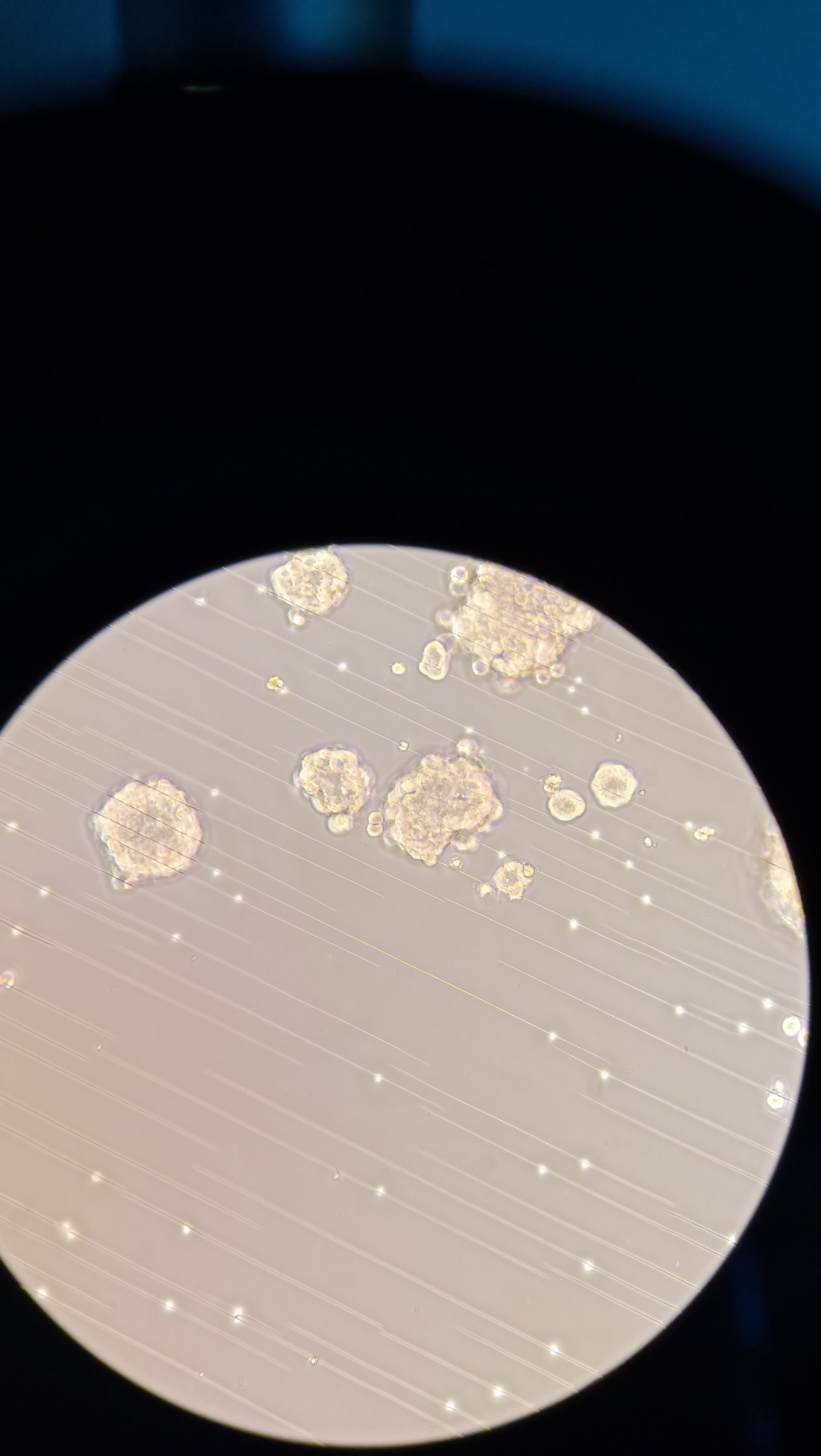

Supplement: Supplementary file 8 — Source data Fig. 3 [file 44319_2024_180_MOESM8_ESM.zip › Figure 3/3D/HPF-CM siDDR1.tif]

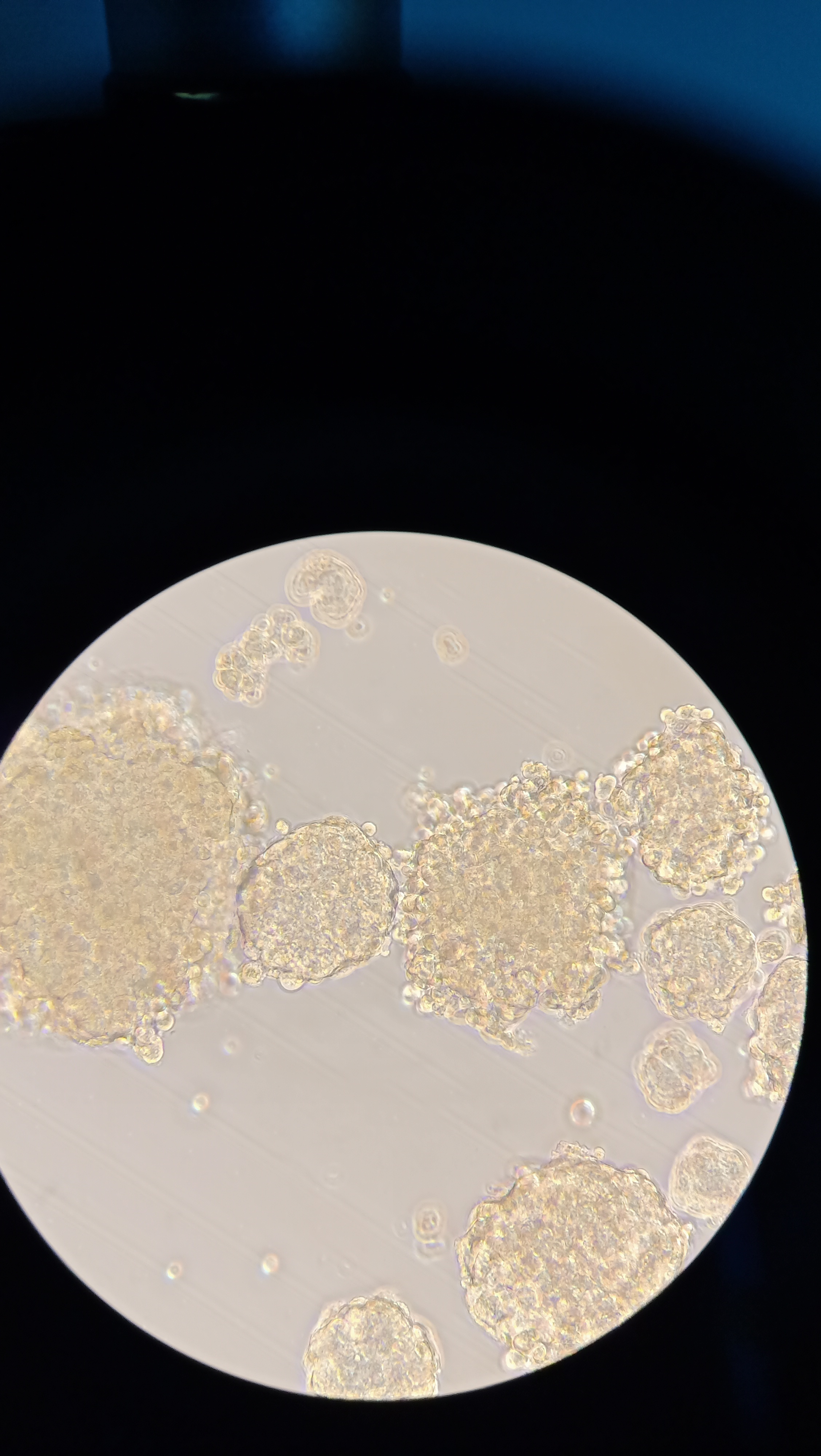

Supplement: Supplementary file 8 — Source data Fig. 3 [file 44319_2024_180_MOESM8_ESM.zip › Figure 3/3D/CAF-CM siCTR.tif]

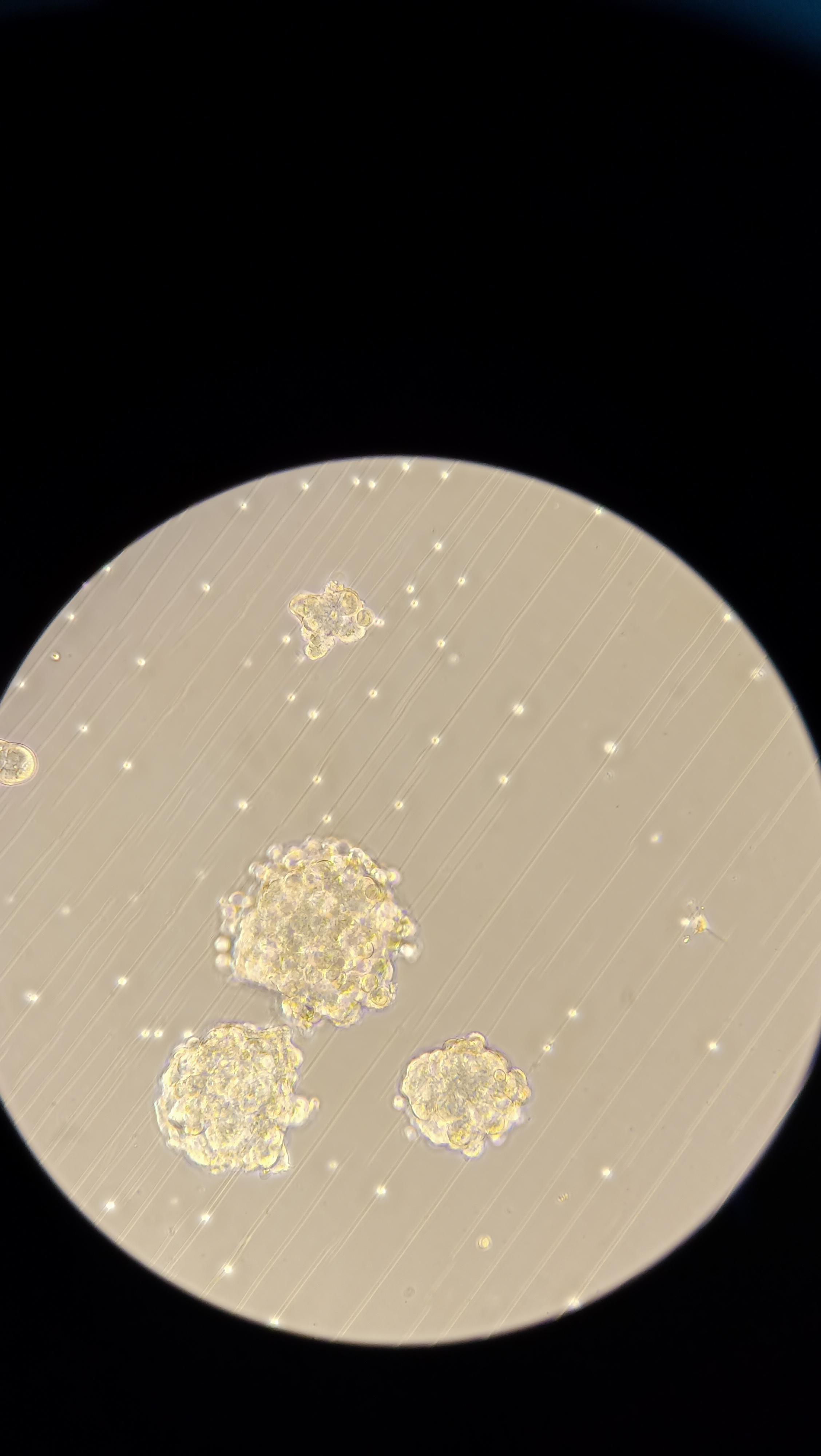

Supplement: Supplementary file 8 — Source data Fig. 3 [file 44319_2024_180_MOESM8_ESM.zip › Figure 3/3D/HPF-CM siCTR.tif]

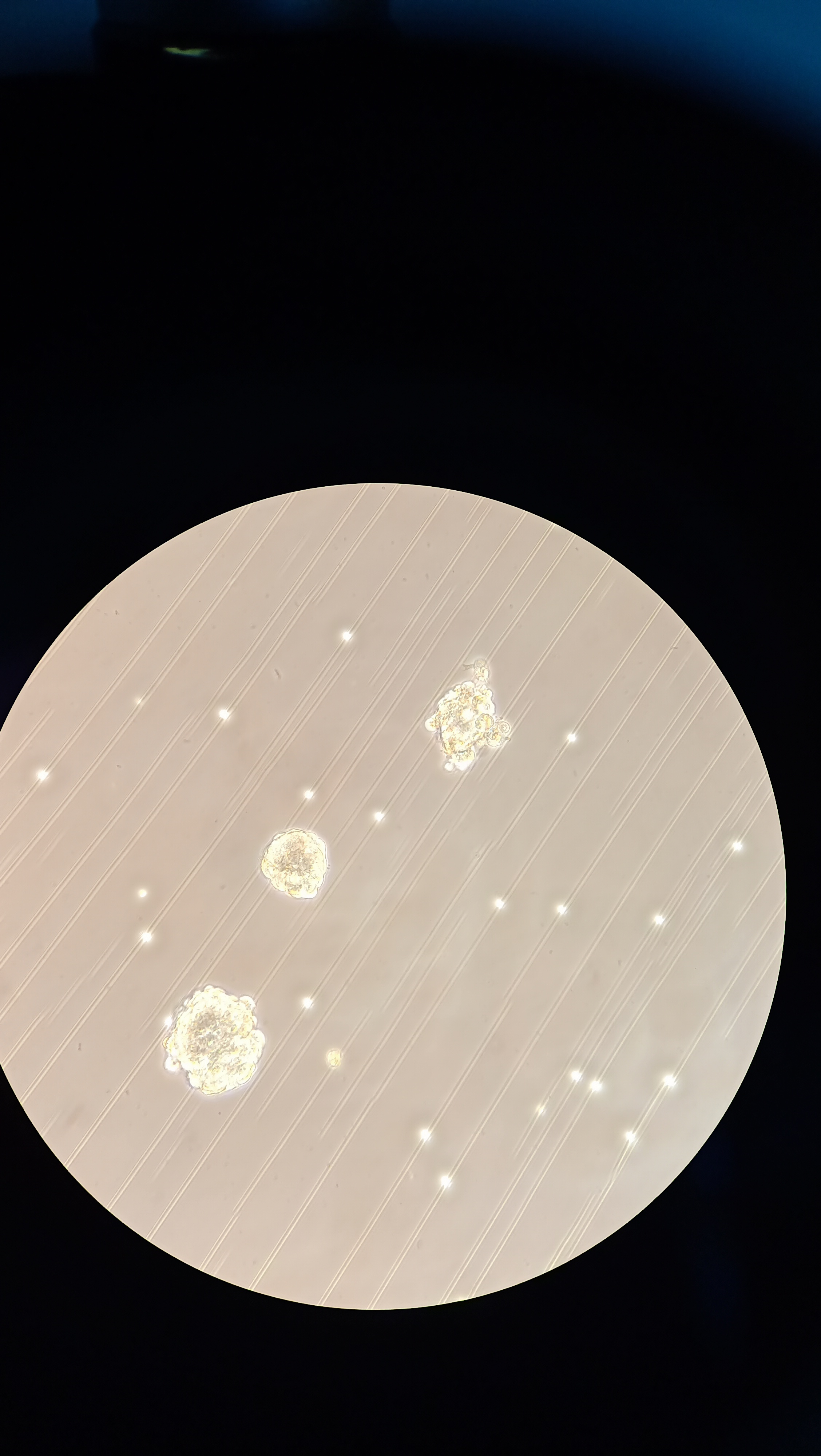

Supplement: Supplementary file 8 — Source data Fig. 3 [file 44319_2024_180_MOESM8_ESM.zip › Figure 3/3D/Lactate siDDR1.tif]

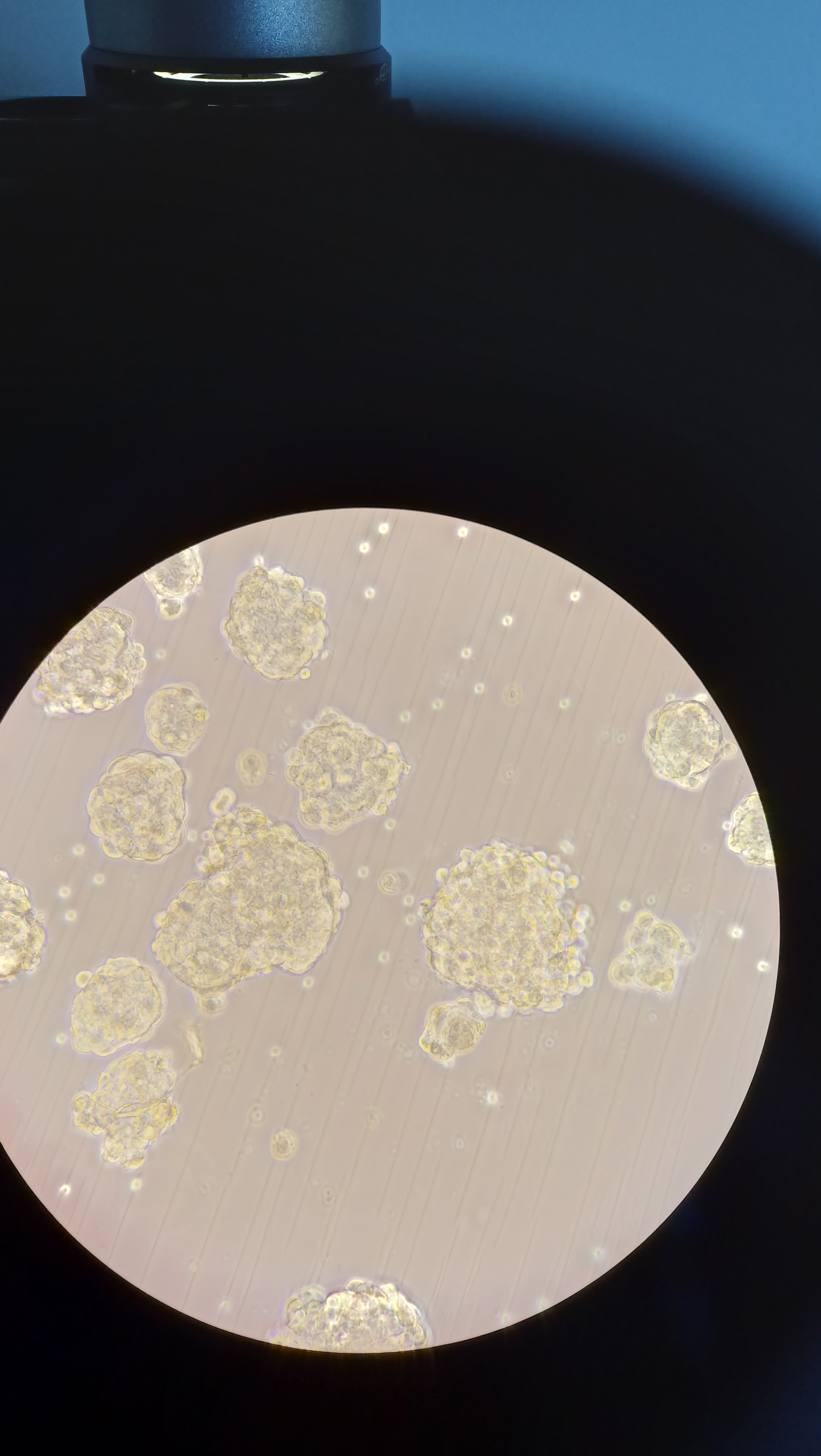

Supplement: Supplementary file 8 — Source data Fig. 3 [file 44319_2024_180_MOESM8_ESM.zip › Figure 3/3D/Lactate siCTR.tif]

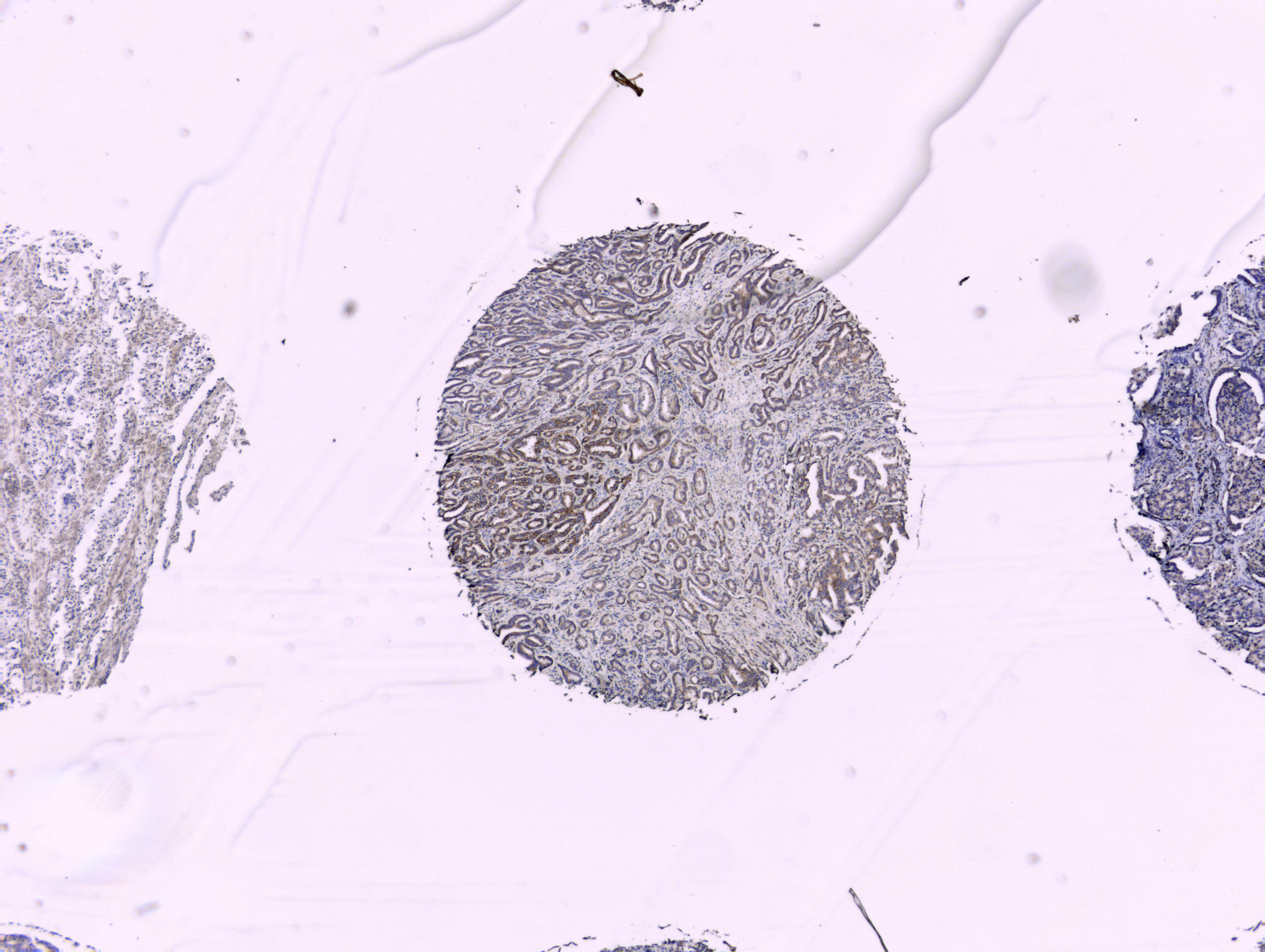

Supplement: Supplementary file 8 — Source data Fig. 3 [file 44319_2024_180_MOESM8_ESM.zip › Figure 3/3H/Low.tif]

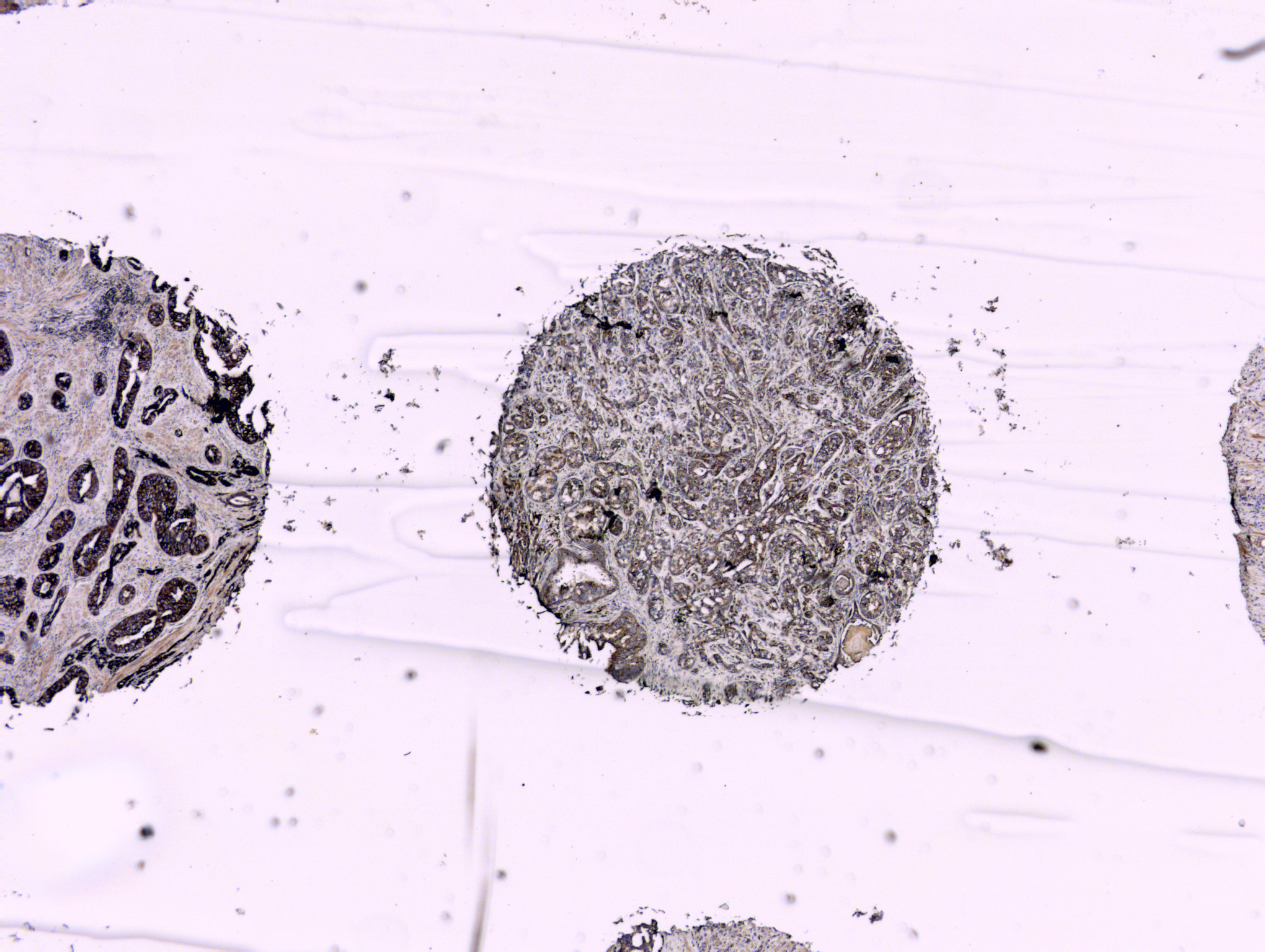

Supplement: Supplementary file 8 — Source data Fig. 3 [file 44319_2024_180_MOESM8_ESM.zip › Figure 3/3H/High.tif]

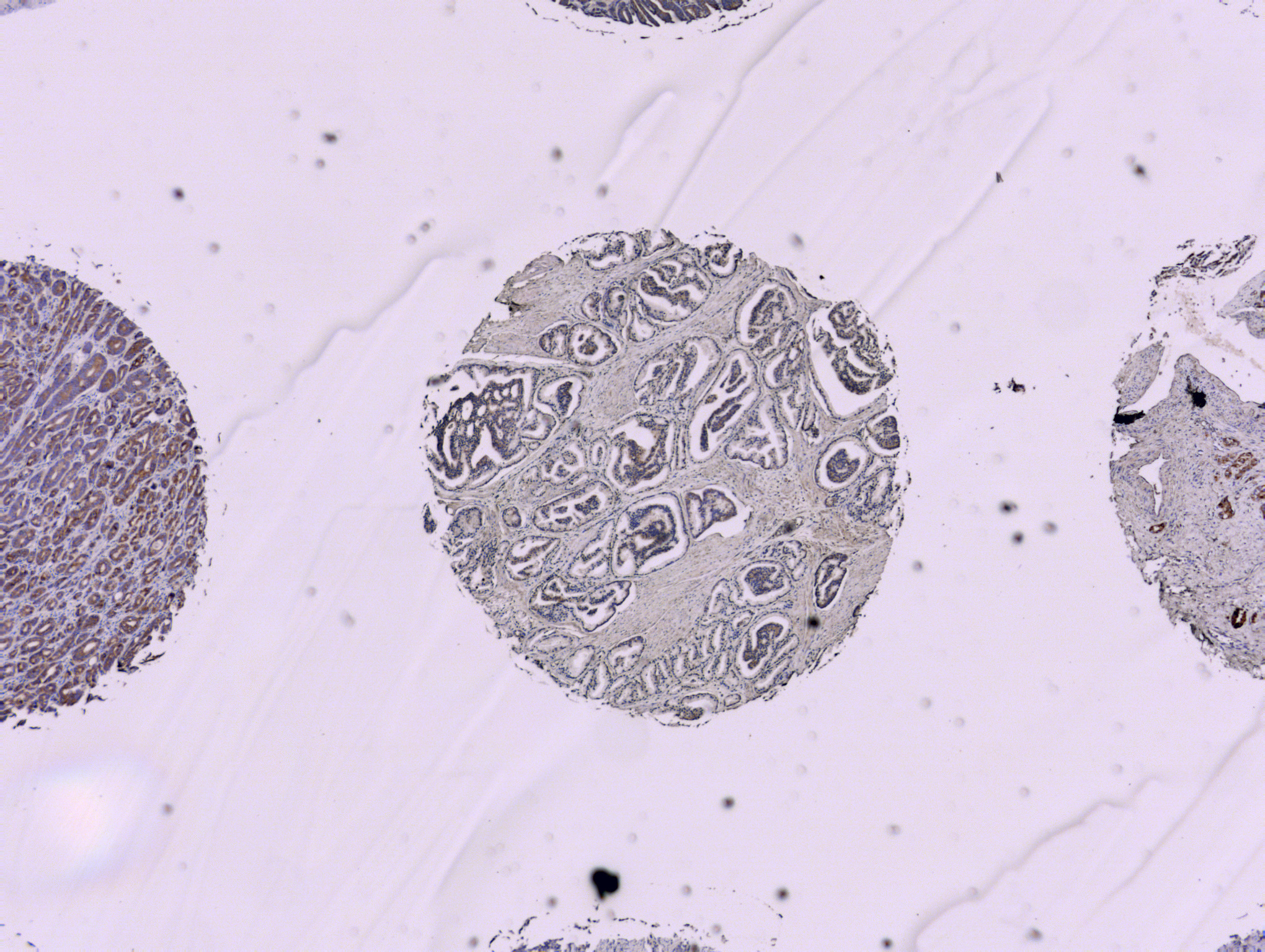

Supplement: Supplementary file 8 — Source data Fig. 3 [file 44319_2024_180_MOESM8_ESM.zip › Figure 3/3H/Benign.tif]

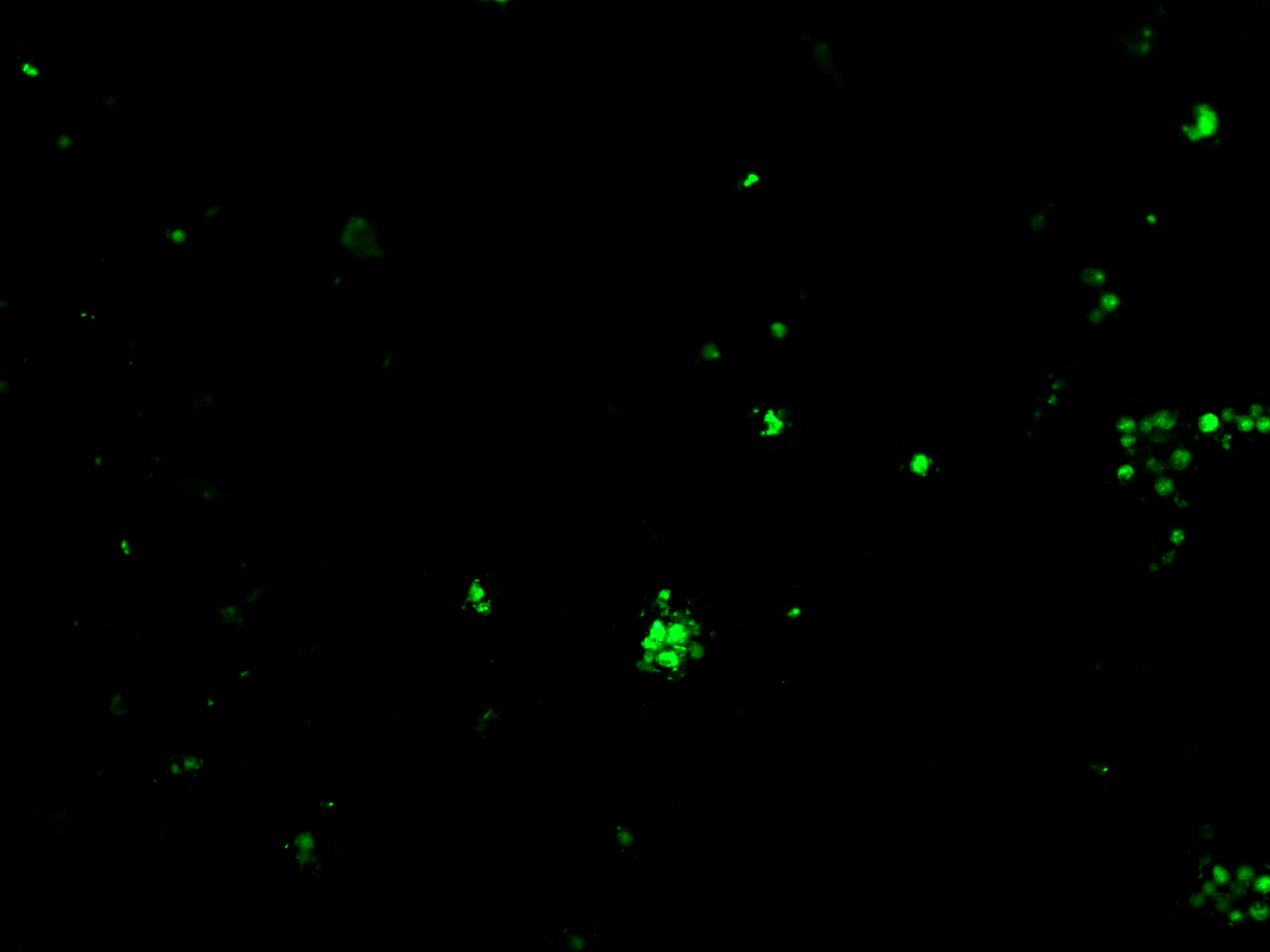

Supplement: Supplementary file 8 — Source data Fig. 3 [file 44319_2024_180_MOESM8_ESM.zip › Figure 3/3G/siCTR HPF-CM.tif]

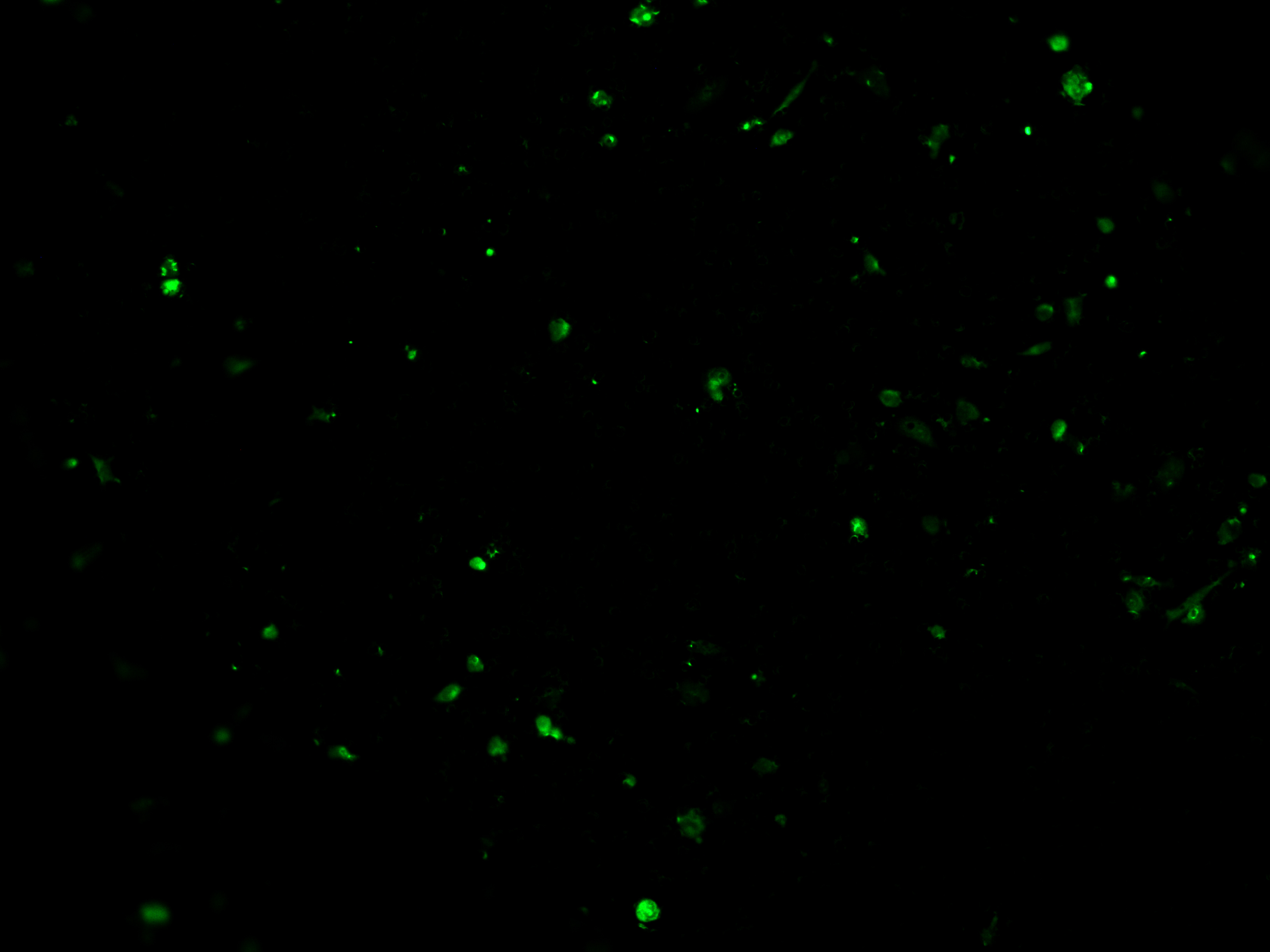

Supplement: Supplementary file 8 — Source data Fig. 3 [file 44319_2024_180_MOESM8_ESM.zip › Figure 3/3G/siDDR1 HPF-CM.tif]

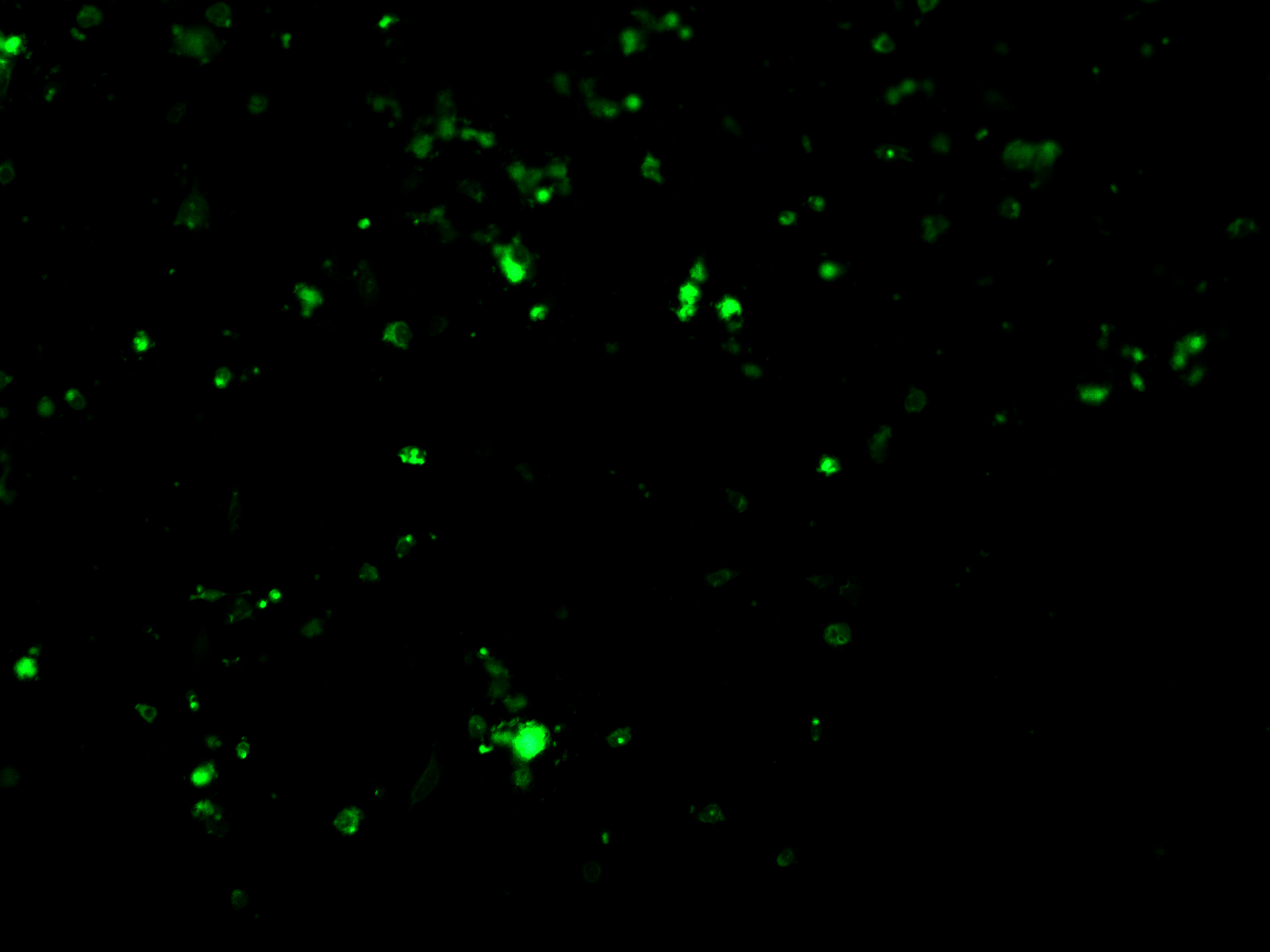

Supplement: Supplementary file 8 — Source data Fig. 3 [file 44319_2024_180_MOESM8_ESM.zip › Figure 3/3G/siDDR1 Lactate.tif]

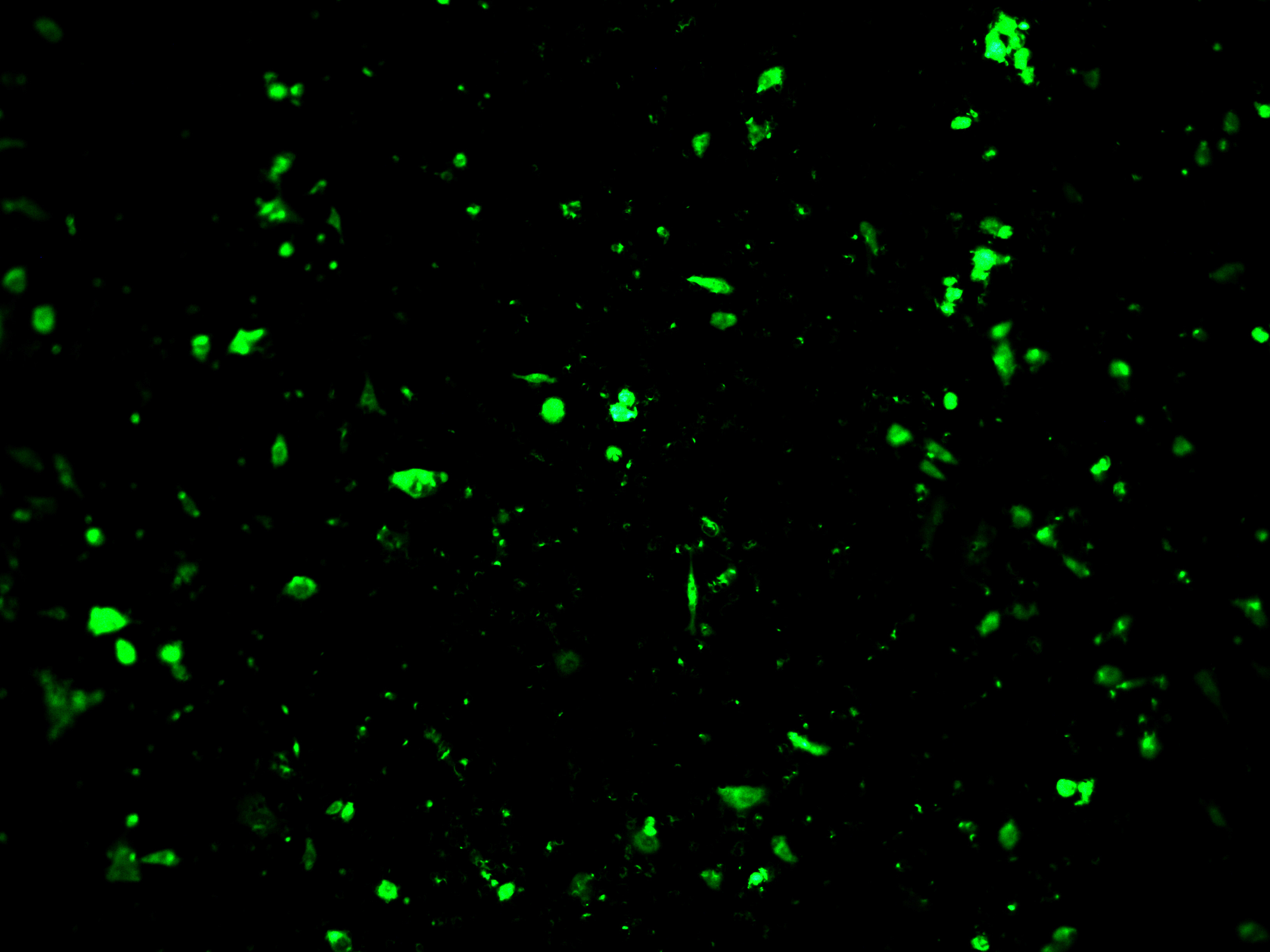

Supplement: Supplementary file 8 — Source data Fig. 3 [file 44319_2024_180_MOESM8_ESM.zip › Figure 3/3G/siCTR Lactate.tif]

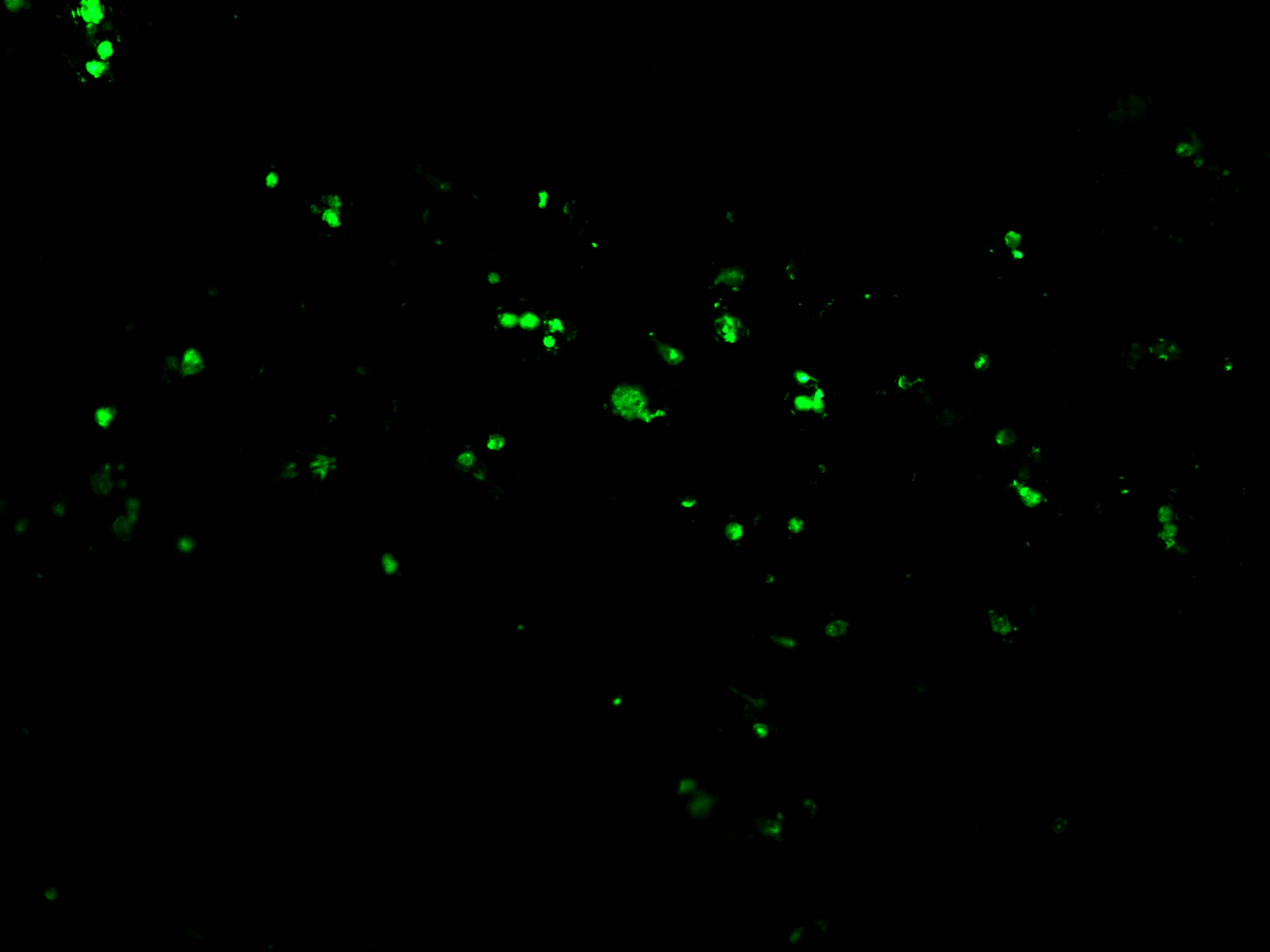

Supplement: Supplementary file 8 — Source data Fig. 3 [file 44319_2024_180_MOESM8_ESM.zip › Figure 3/3G/siDDR1 CAF-CM.tif]

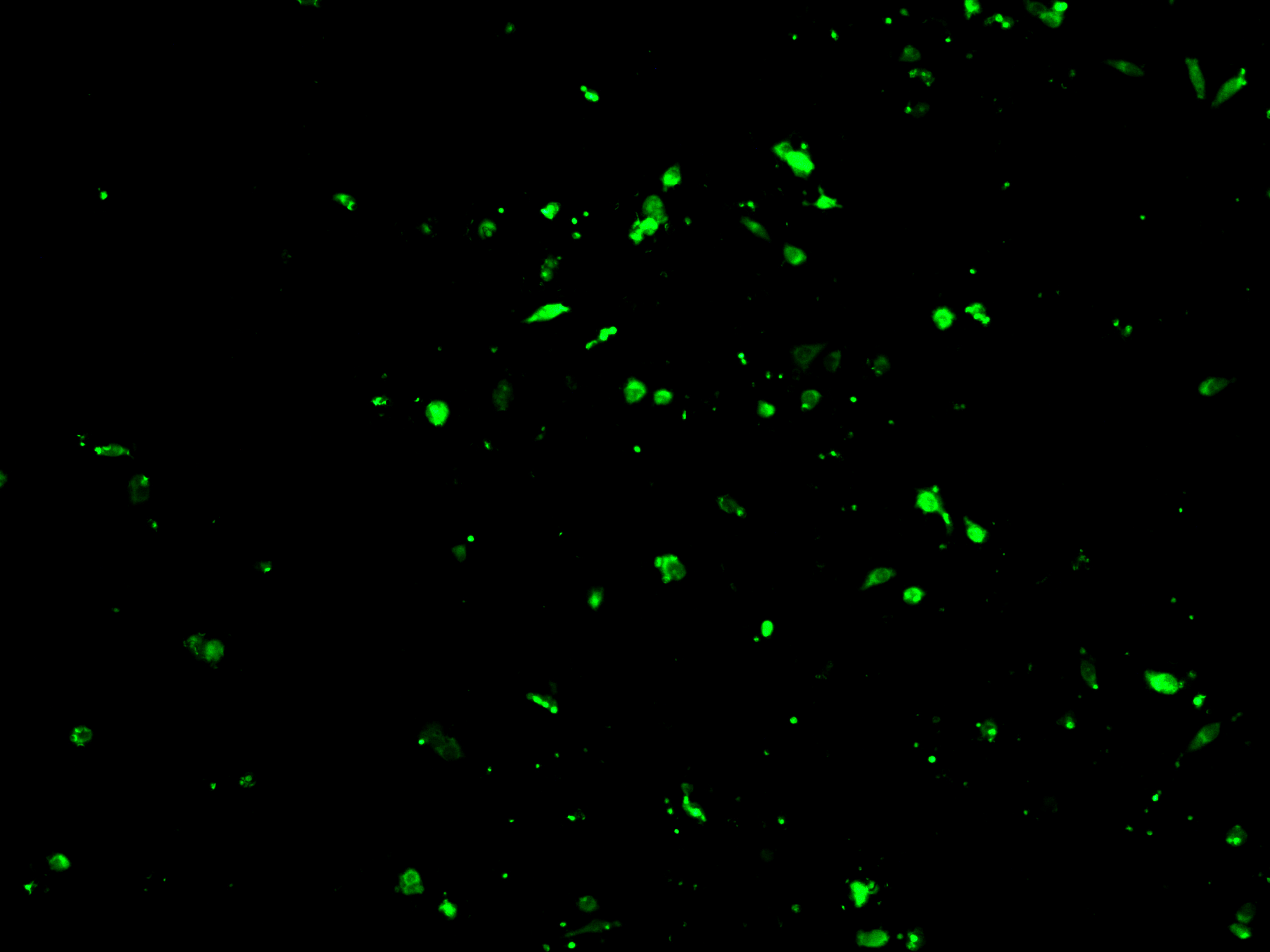

Supplement: Supplementary file 8 — Source data Fig. 3 [file 44319_2024_180_MOESM8_ESM.zip › Figure 3/3G/siCTR CAF-CM.tif]

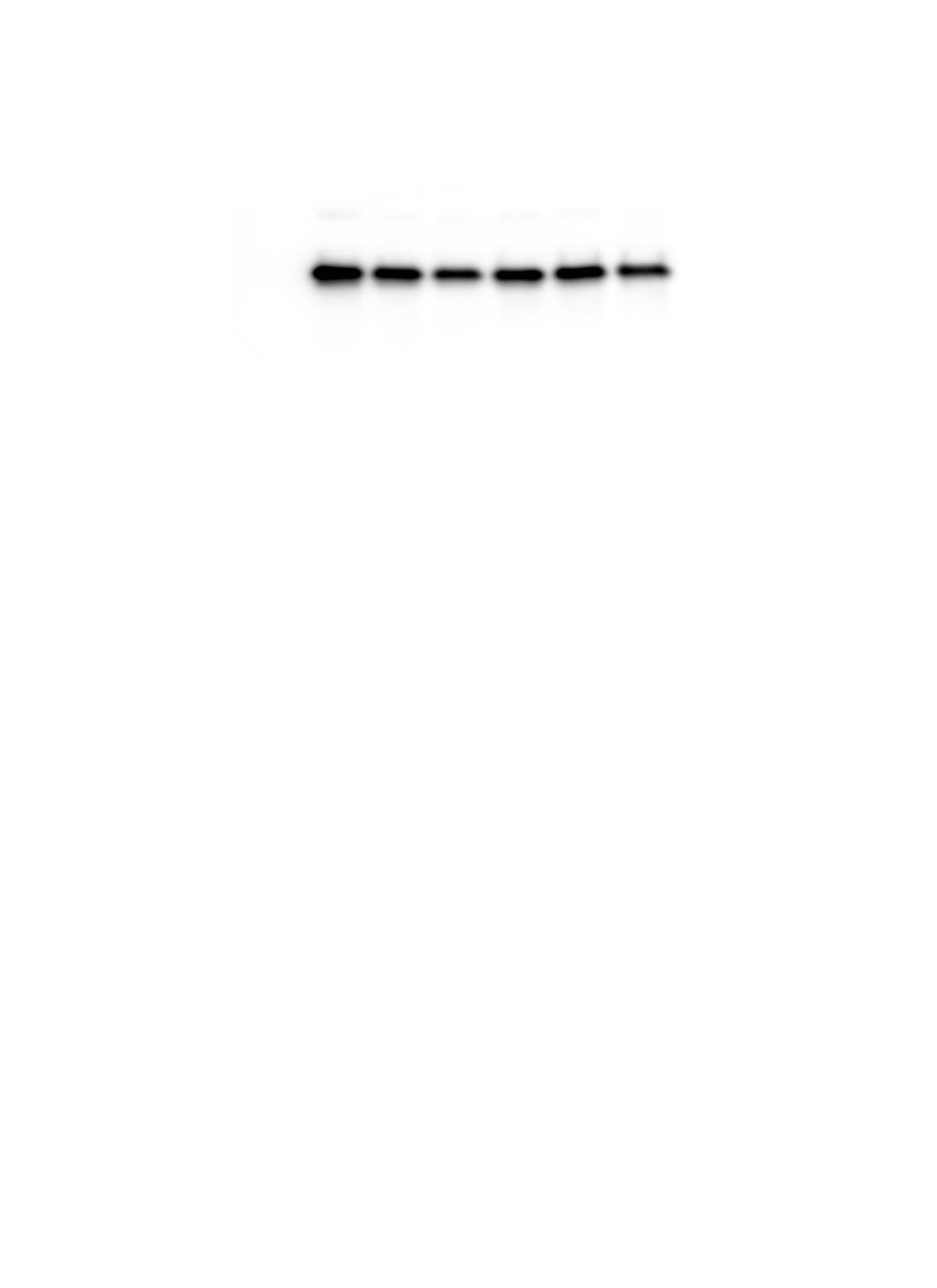

Supplement: Supplementary file 9 — Source data Fig. 4 [file 44319_2024_180_MOESM9_ESM.zip › Figure 4/4B/WB Actin ecm.tif]

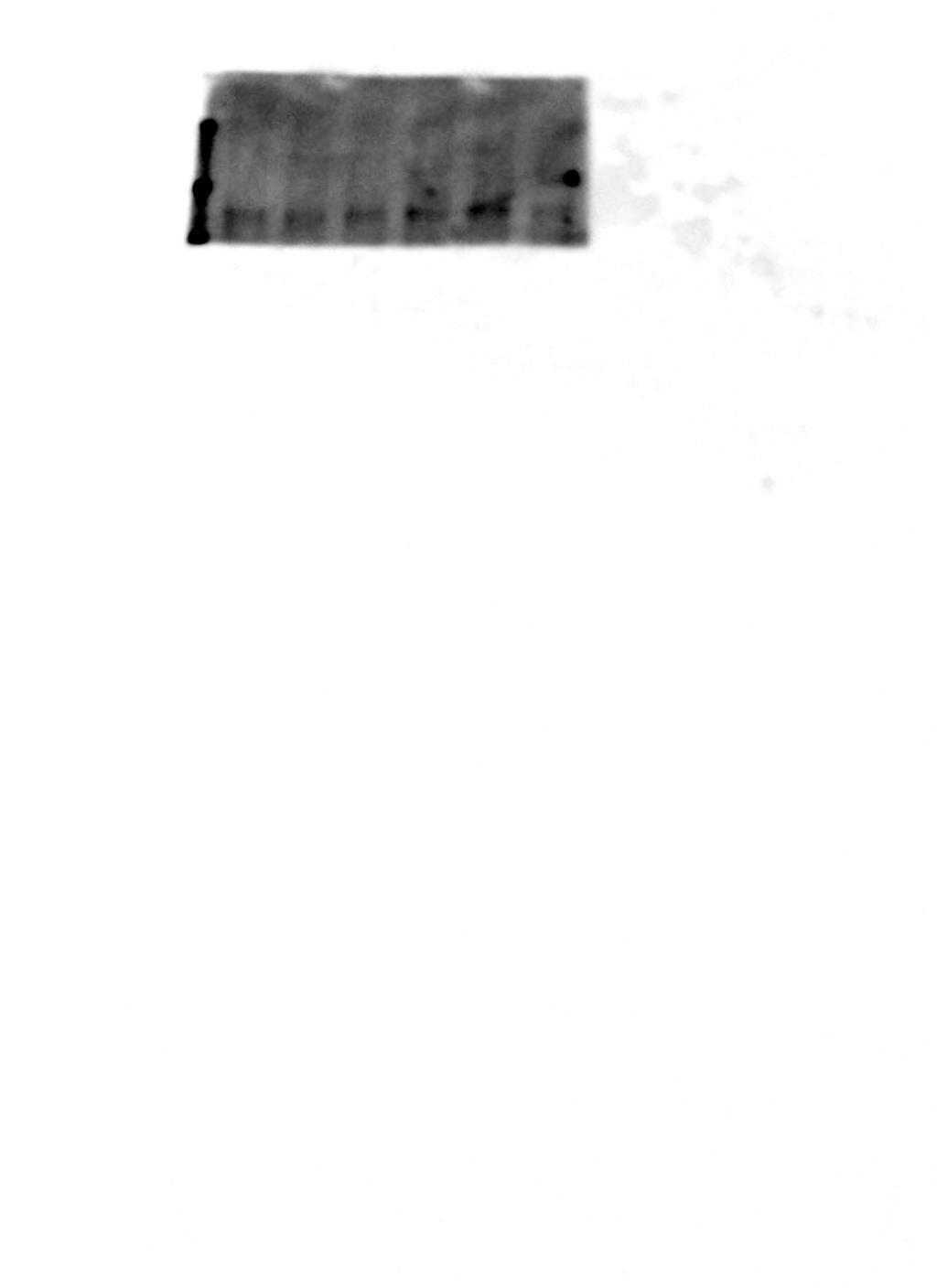

Supplement: Supplementary file 9 — Source data Fig. 4 [file 44319_2024_180_MOESM9_ESM.zip › Figure 4/4B/WB pDDR1 ecm.tif]

Figure 4b

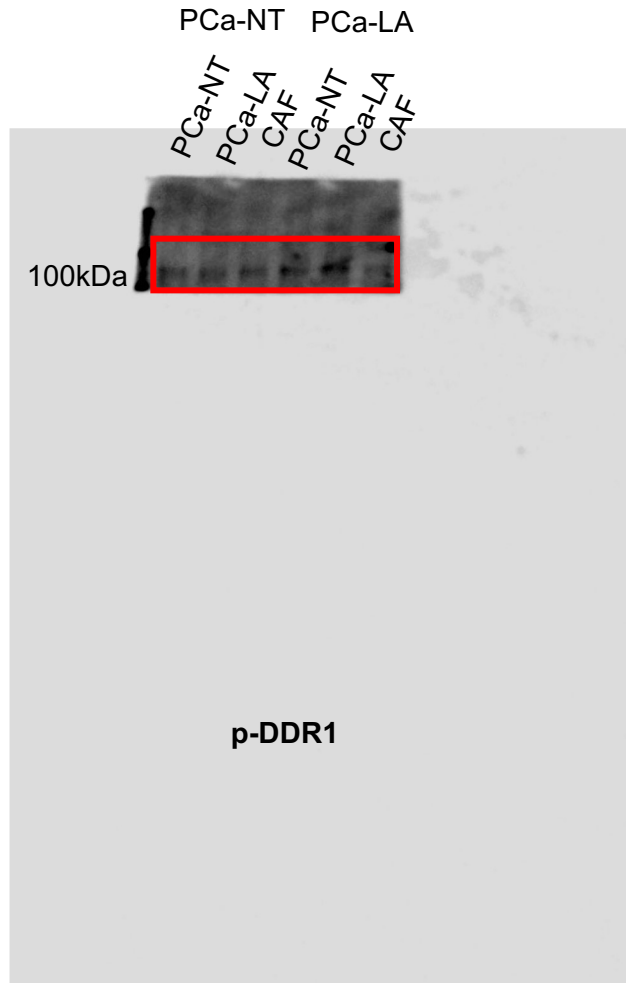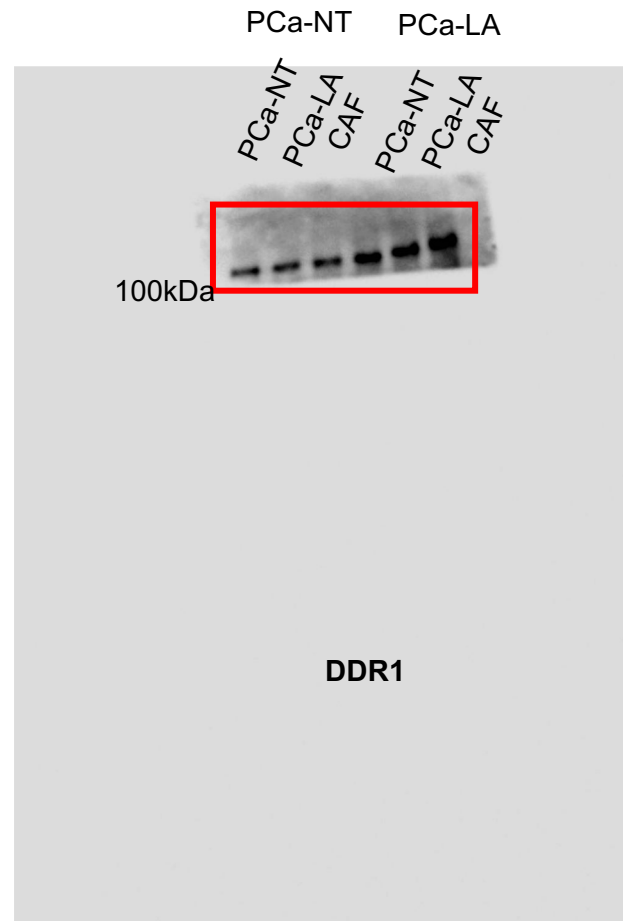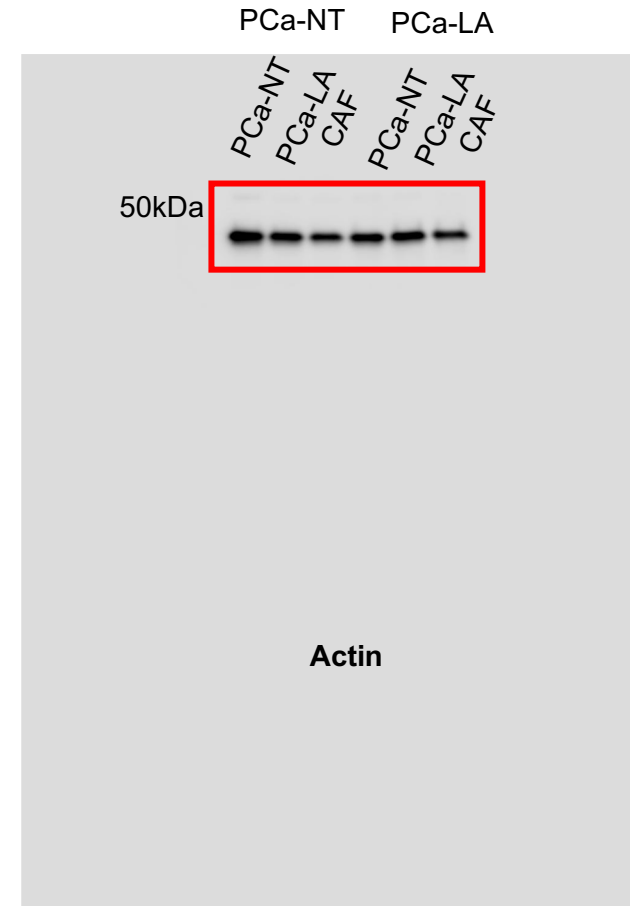

Supplement: Supplementary file 9 — Source data Fig. 4 [file 44319_2024_180_MOESM9_ESM.zip › Figure 4/4B/4b blot.pdf]

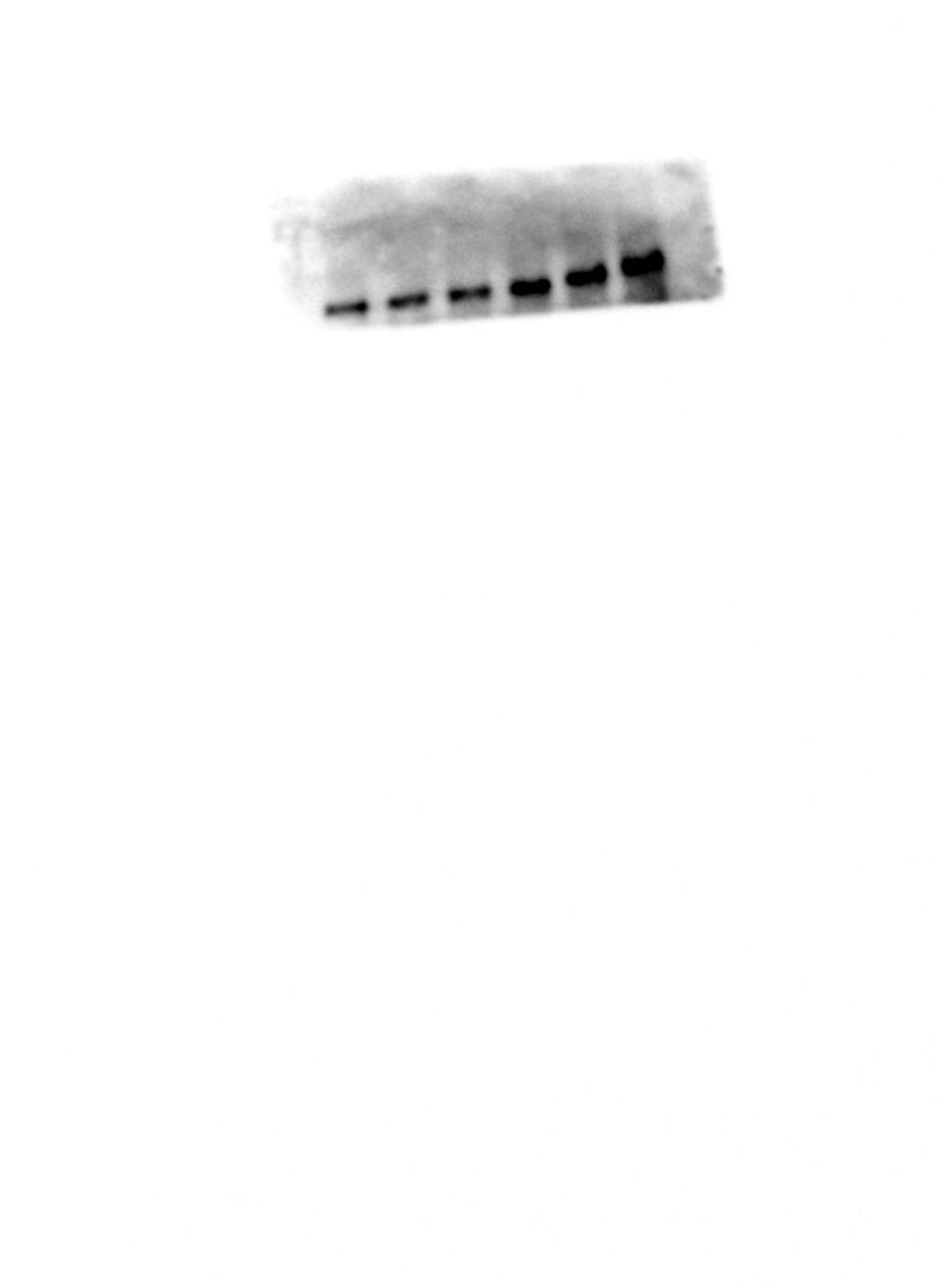

Supplement: Supplementary file 9 — Source data Fig. 4 [file 44319_2024_180_MOESM9_ESM.zip › Figure 4/4B/WB DDR1 ecm.tif]

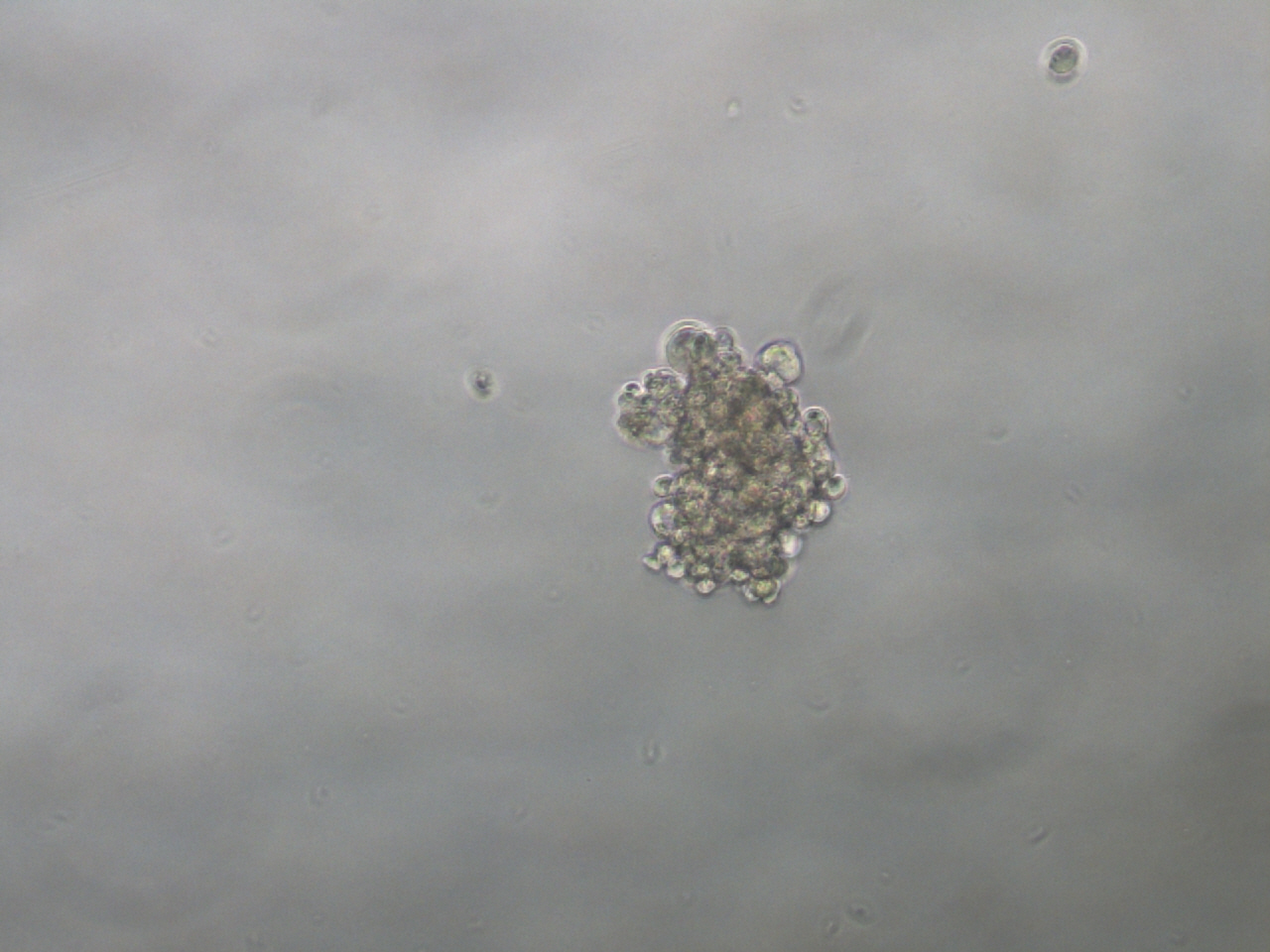

Supplement: Supplementary file 9 — Source data Fig. 4 [file 44319_2024_180_MOESM9_ESM.zip › Figure 4/4D/HPF-CM siCOL1.tif]

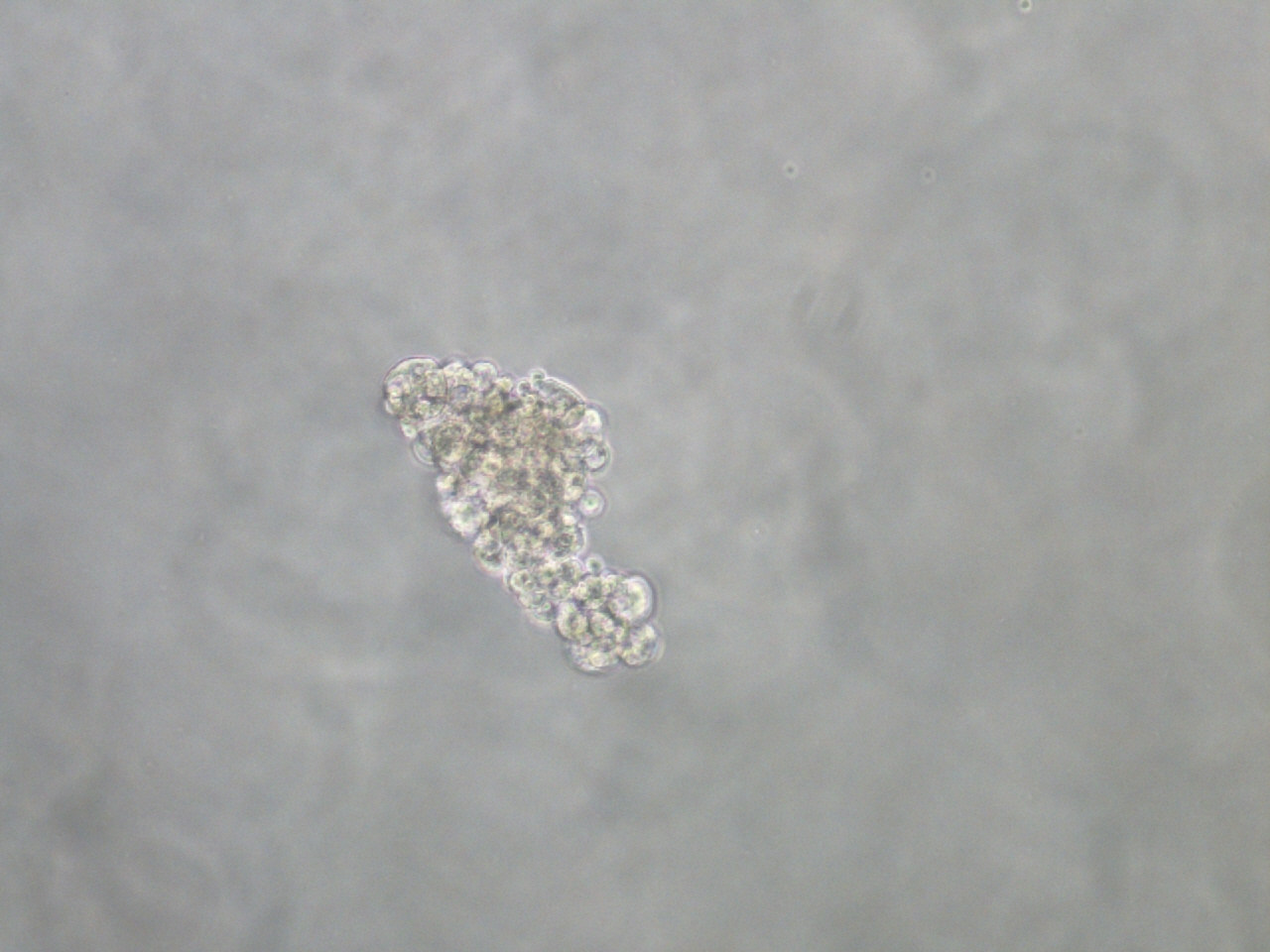

Supplement: Supplementary file 9 — Source data Fig. 4 [file 44319_2024_180_MOESM9_ESM.zip › Figure 4/4D/CAF-CM siCOL1.tif]

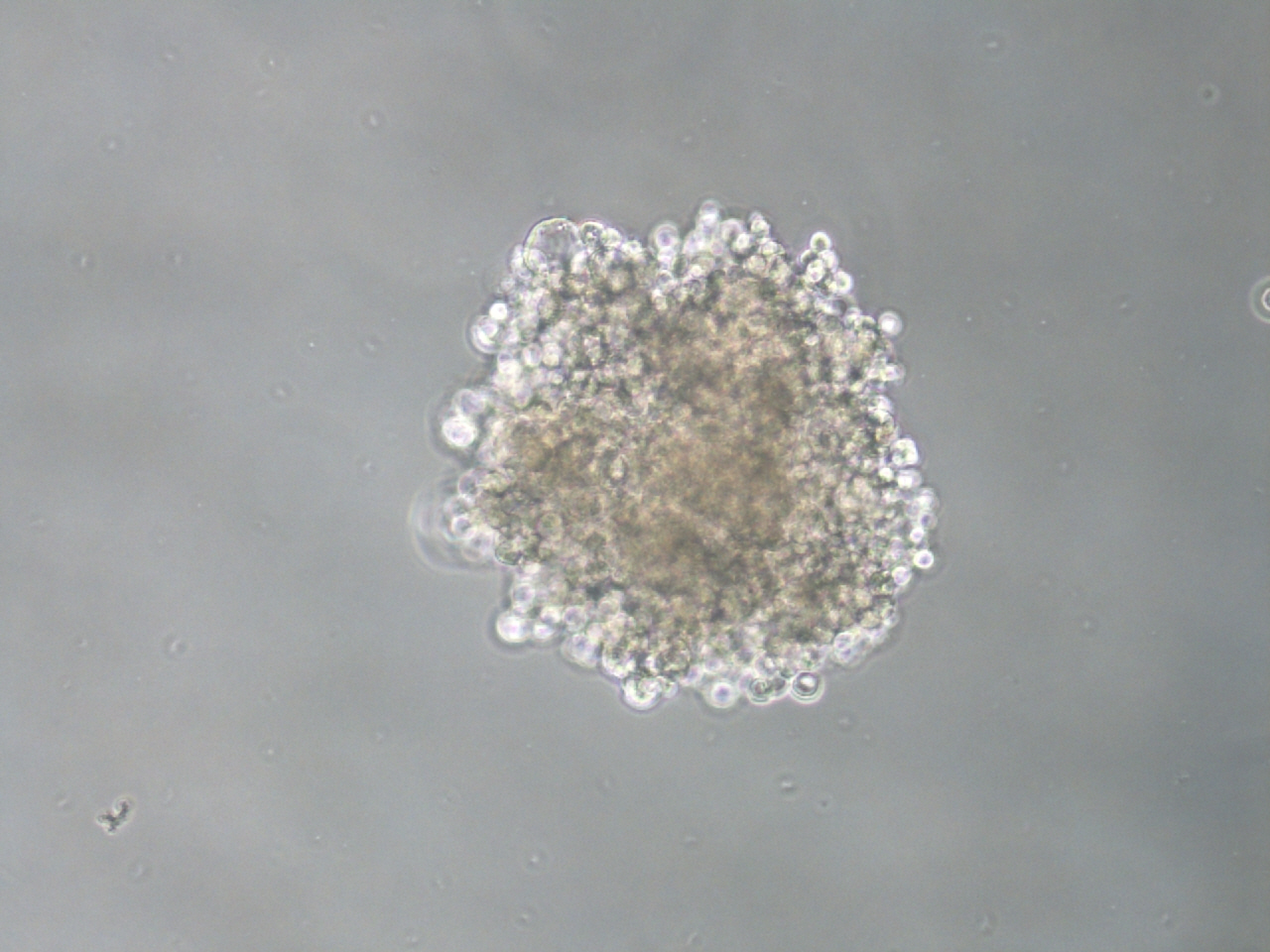

Supplement: Supplementary file 9 — Source data Fig. 4 [file 44319_2024_180_MOESM9_ESM.zip › Figure 4/4D/CAF-CM siCTR.tif]

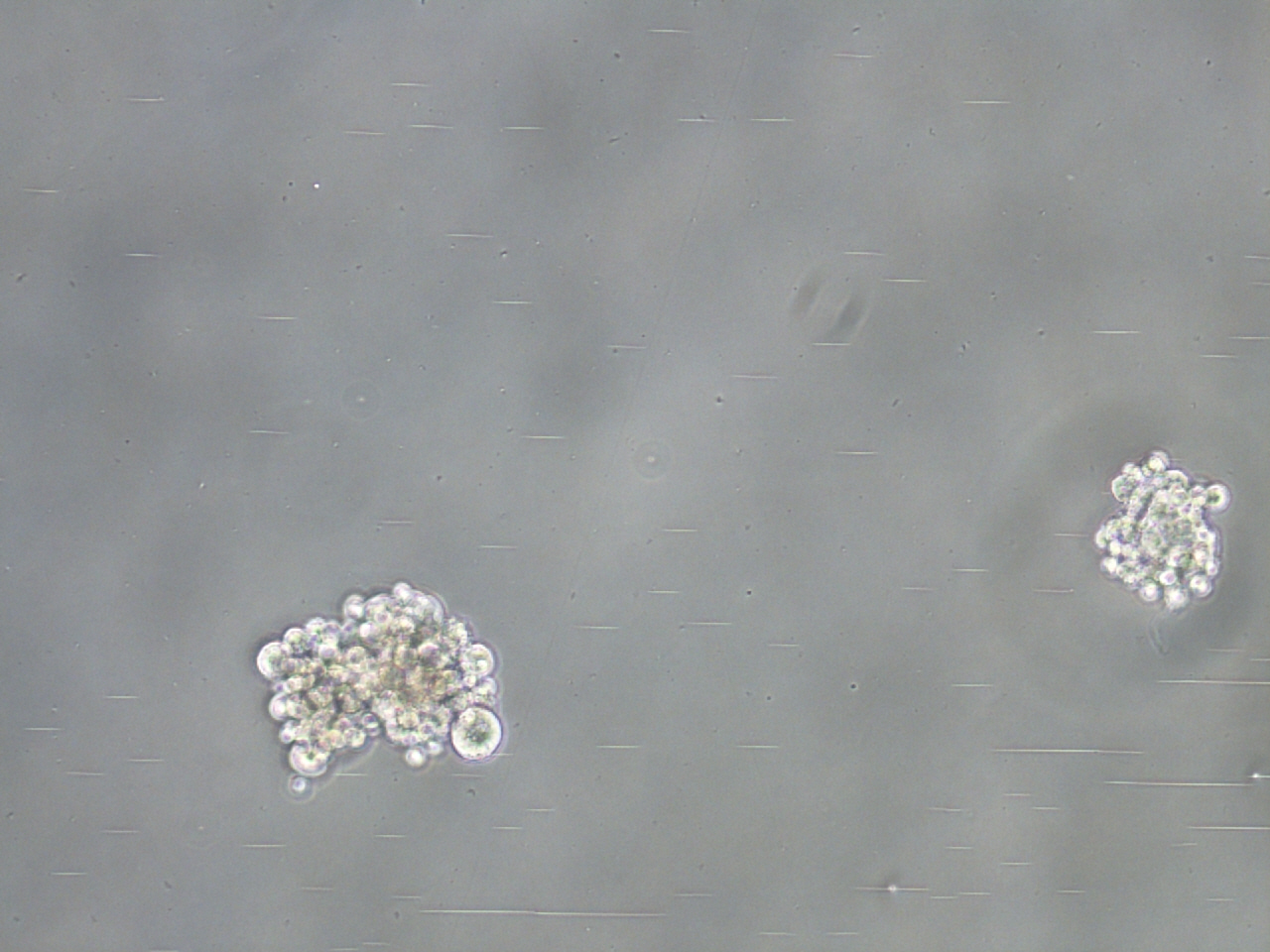

Supplement: Supplementary file 9 — Source data Fig. 4 [file 44319_2024_180_MOESM9_ESM.zip › Figure 4/4D/HPF-CM siCTR.tif]

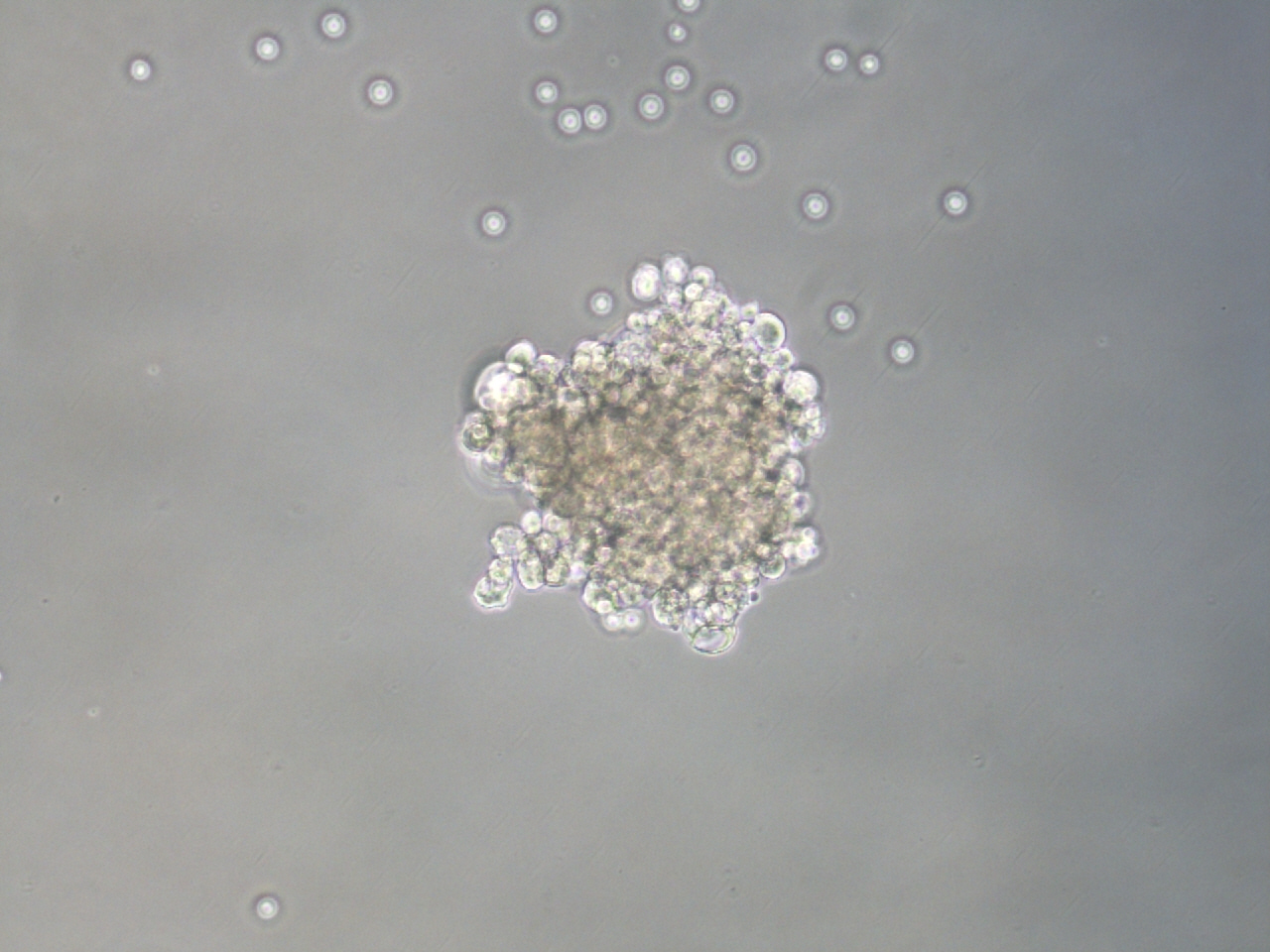

Supplement: Supplementary file 9 — Source data Fig. 4 [file 44319_2024_180_MOESM9_ESM.zip › Figure 4/4D/Lactate siCTR.tif]

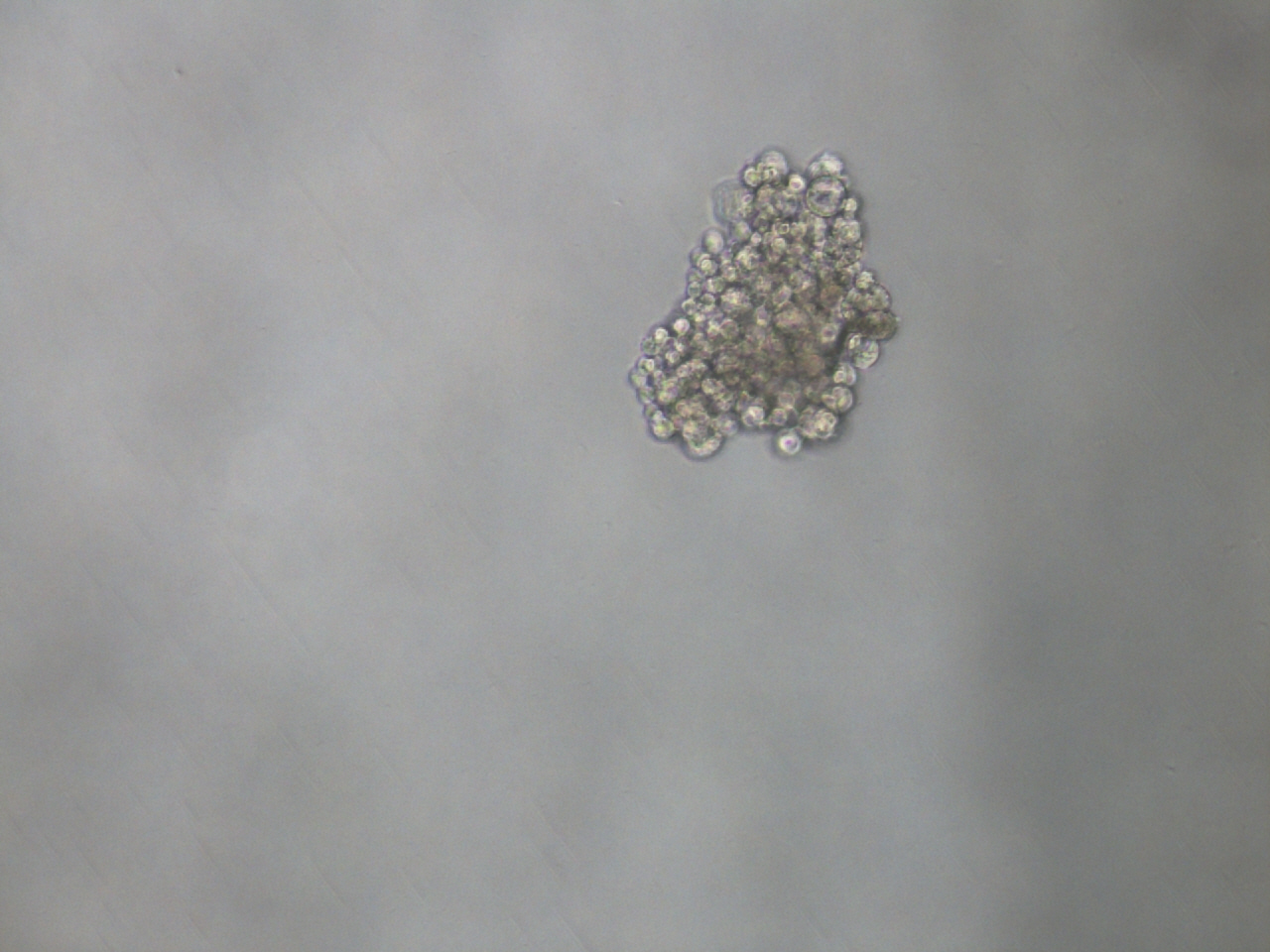

Supplement: Supplementary file 9 — Source data Fig. 4 [file 44319_2024_180_MOESM9_ESM.zip › Figure 4/4D/Lactate siCOL1.tif]

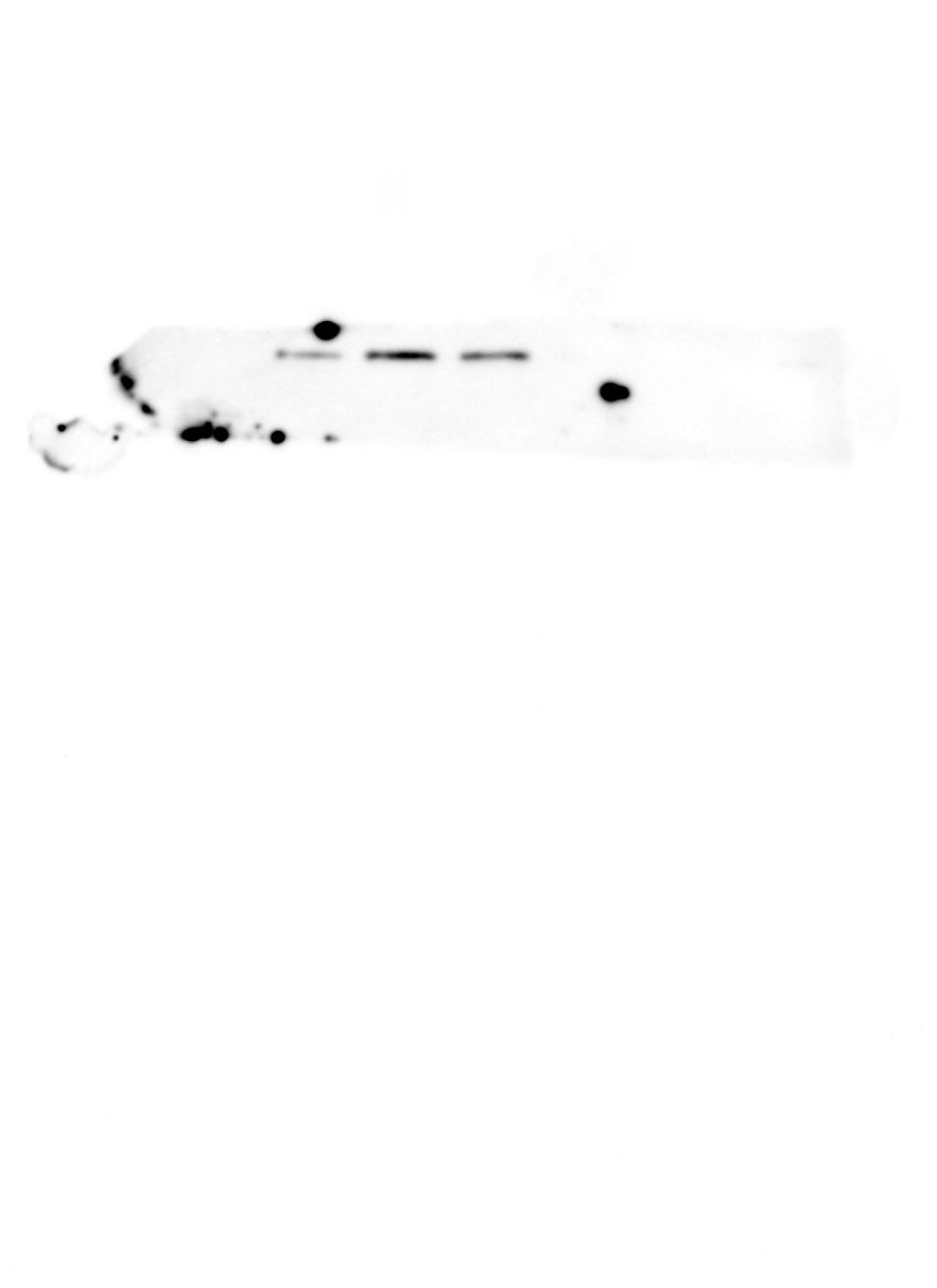

Supplement: Supplementary file 10 — Source data Fig. 5 [file 44319_2024_180_MOESM10_ESM.zip › Figure 5/5G/WB pSTAT3 siSTAT3.tif]

Figure 5g

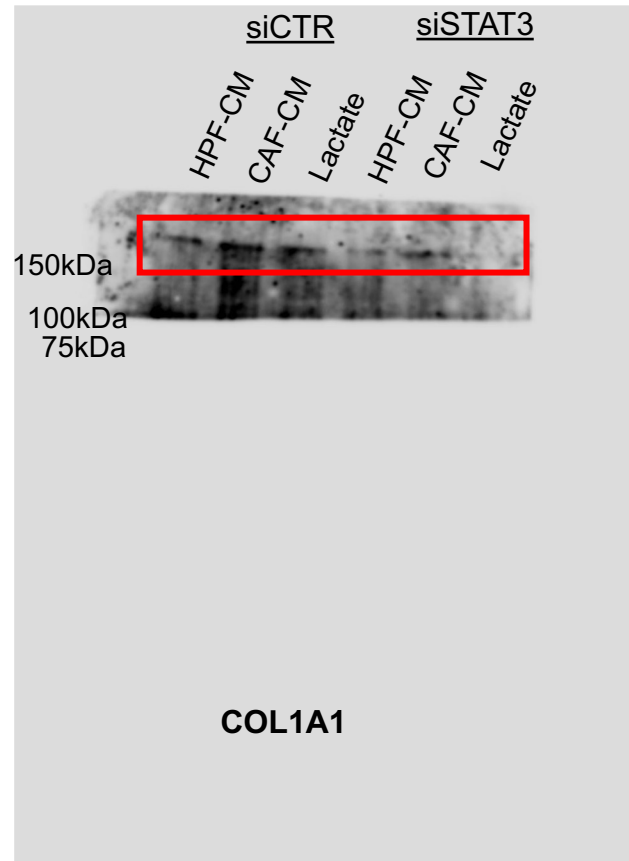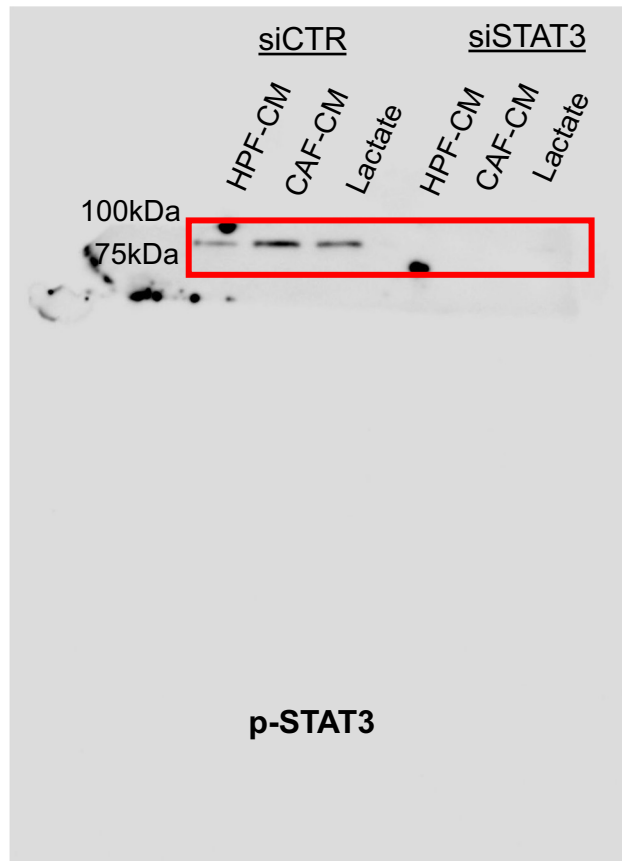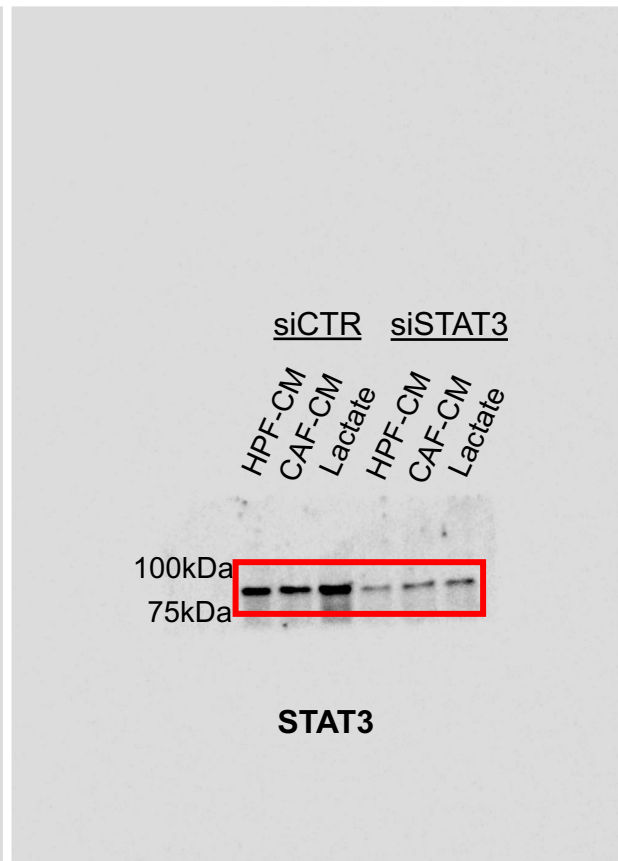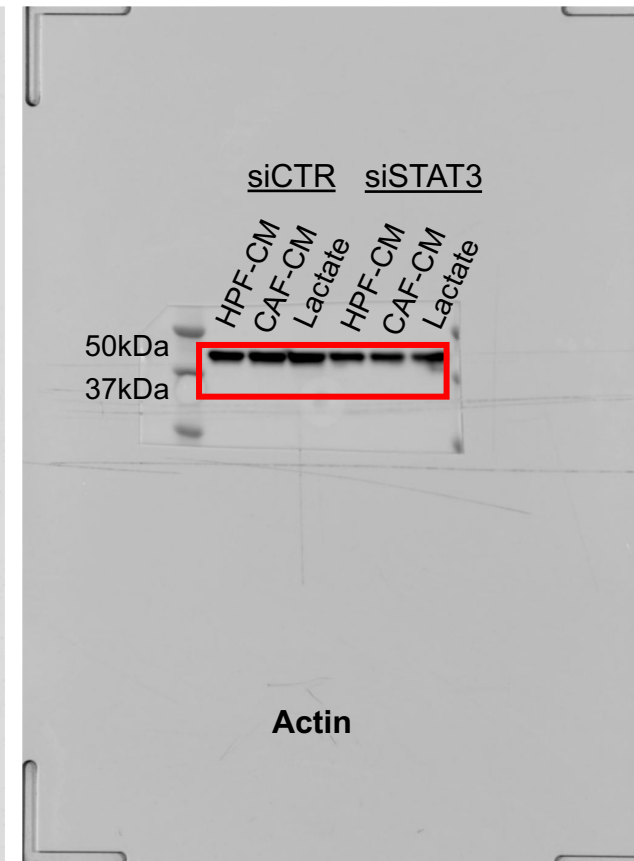

Supplement: Supplementary file 10 — Source data Fig. 5 [file 44319_2024_180_MOESM10_ESM.zip › Figure 5/5G/5g blot.pdf]

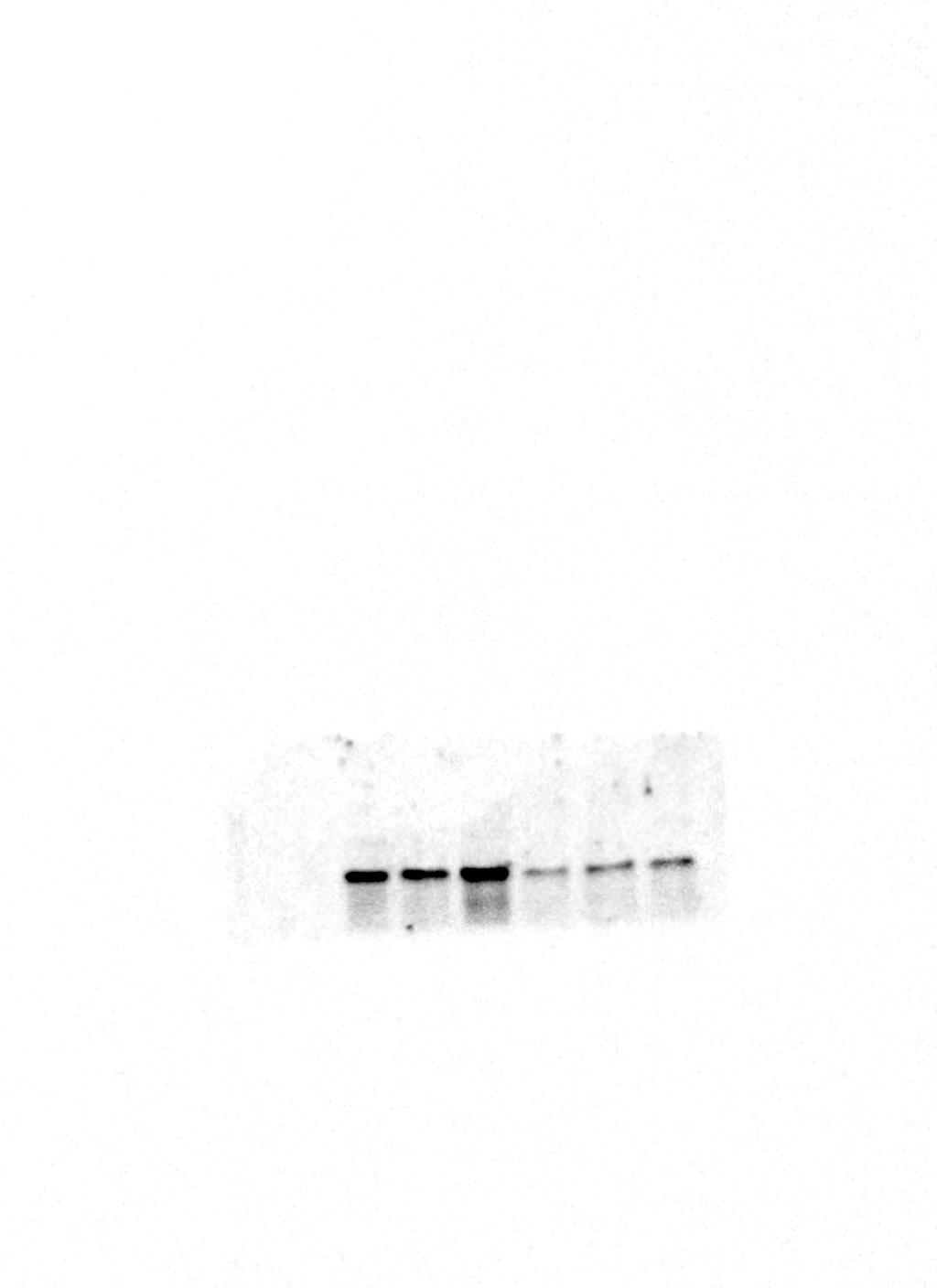

Supplement: Supplementary file 10 — Source data Fig. 5 [file 44319_2024_180_MOESM10_ESM.zip › Figure 5/5G/WB STAT3 siSTAT3.tif]

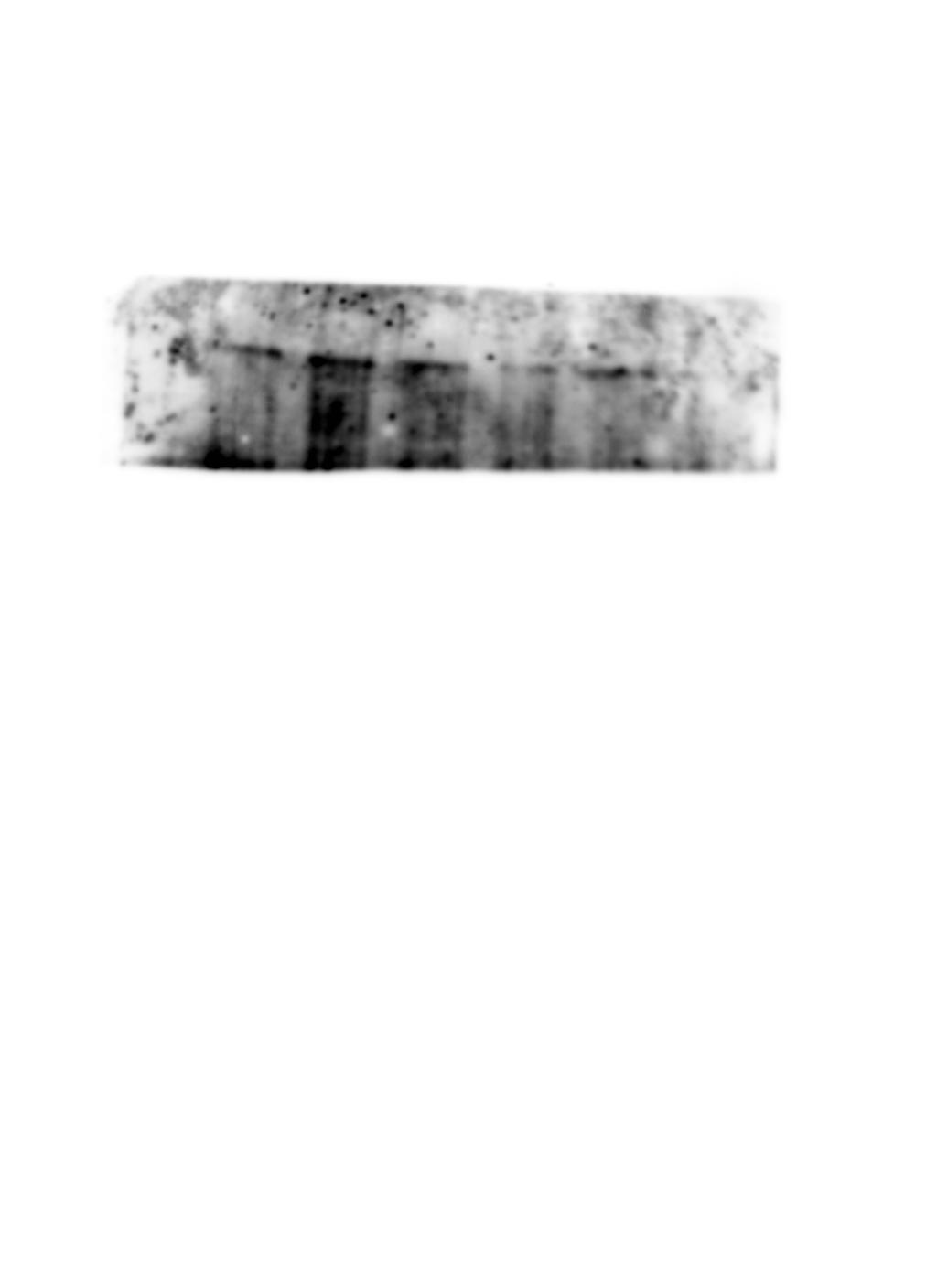

Supplement: Supplementary file 10 — Source data Fig. 5 [file 44319_2024_180_MOESM10_ESM.zip › Figure 5/5G/WB Col1 siSTAT3.tif]

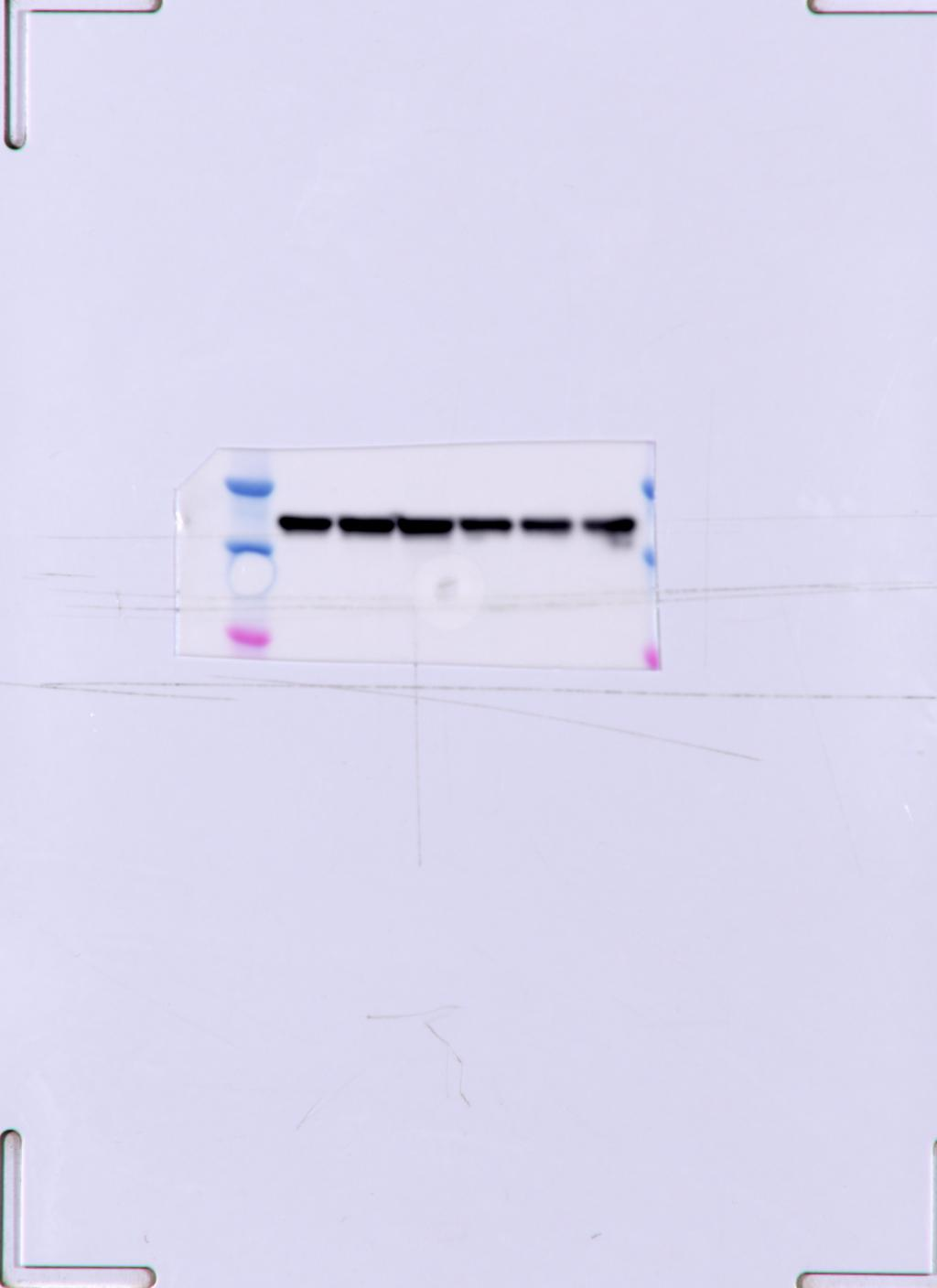

Supplement: Supplementary file 10 — Source data Fig. 5 [file 44319_2024_180_MOESM10_ESM.zip › Figure 5/5G/WB Actin siSTAT3.tif]

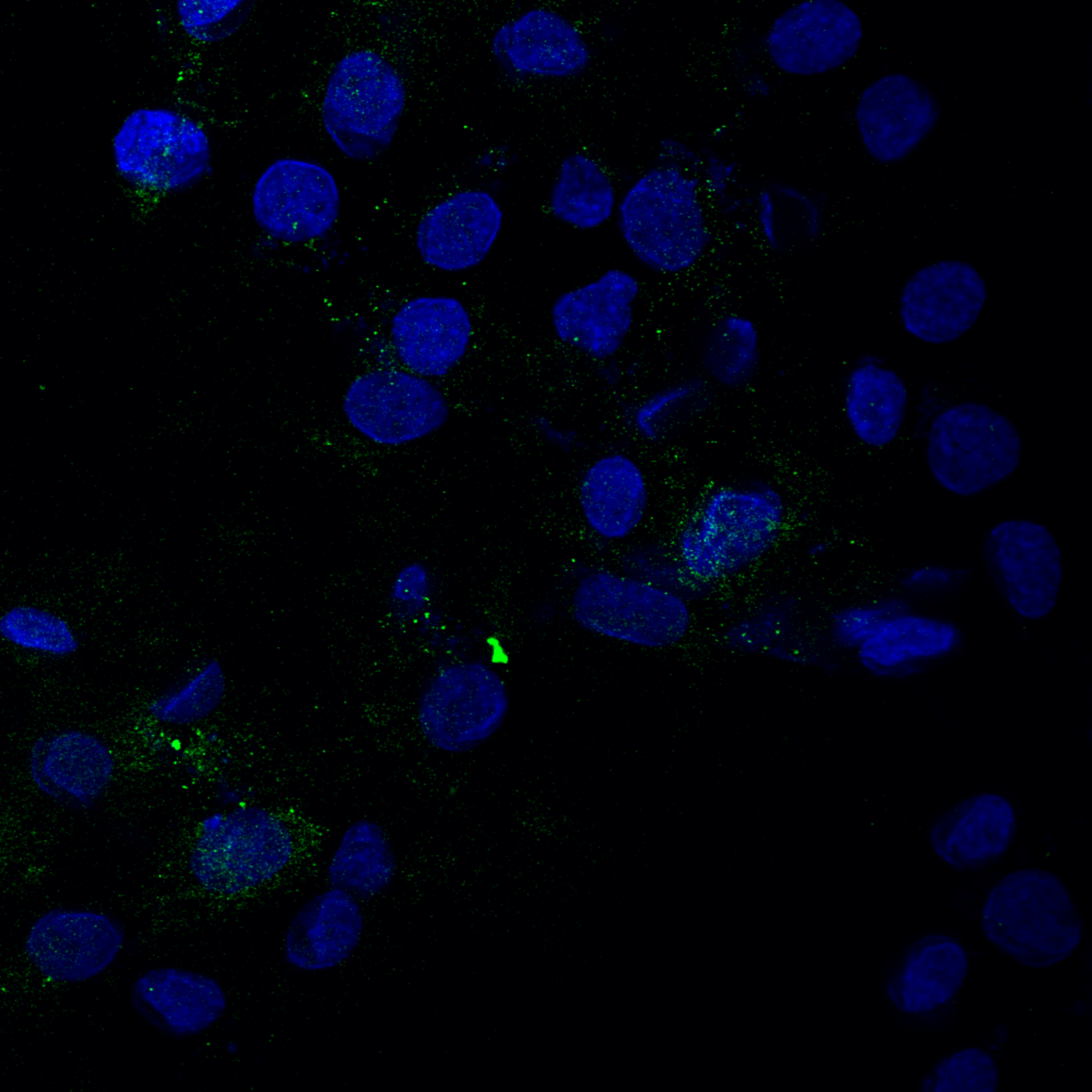

Supplement: Supplementary file 10 — Source data Fig. 5 [file 44319_2024_180_MOESM10_ESM.zip › Figure 5/5F/CAF-CM sistat3.tif]

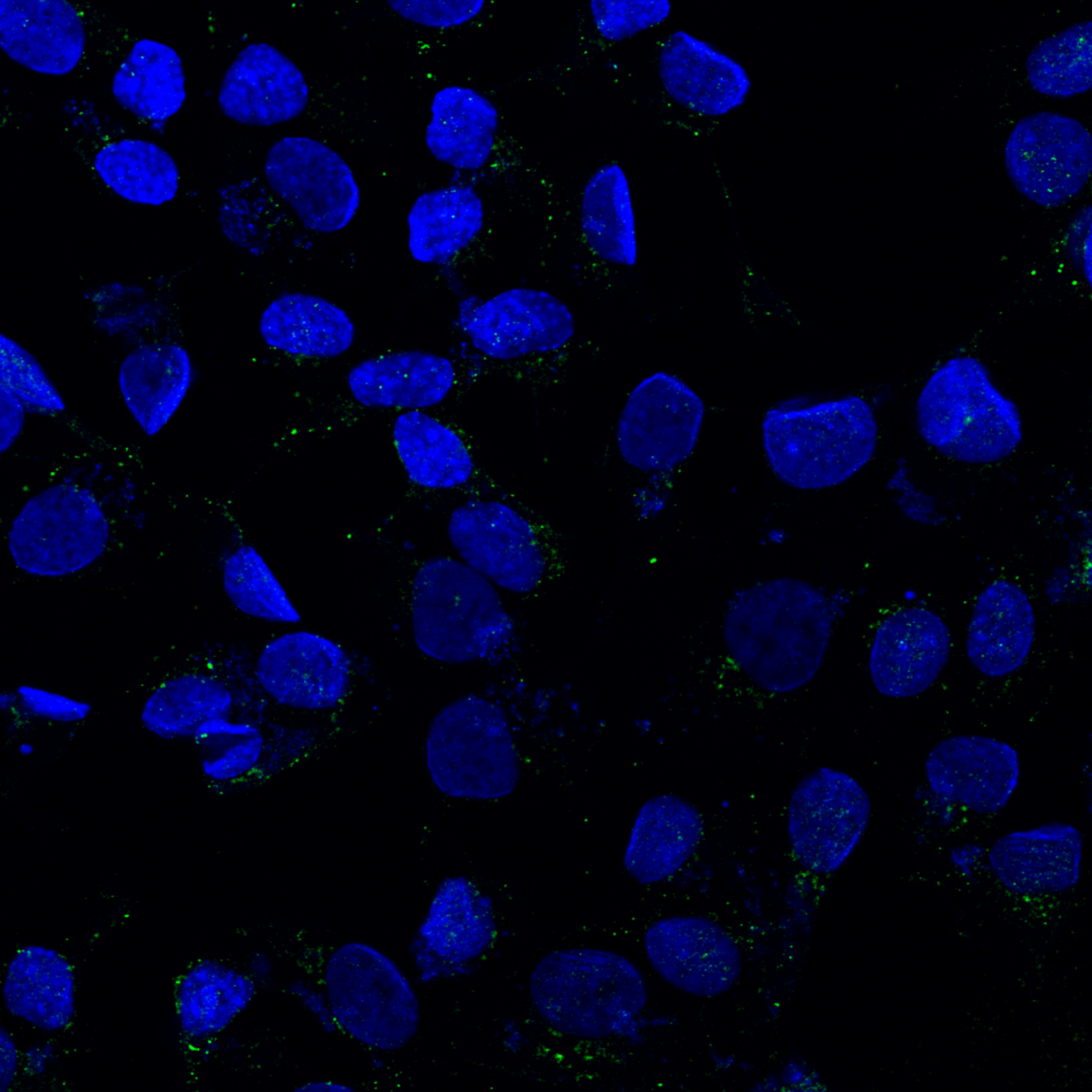

Supplement: Supplementary file 10 — Source data Fig. 5 [file 44319_2024_180_MOESM10_ESM.zip › Figure 5/5F/siCTR HPF-CM.tif]

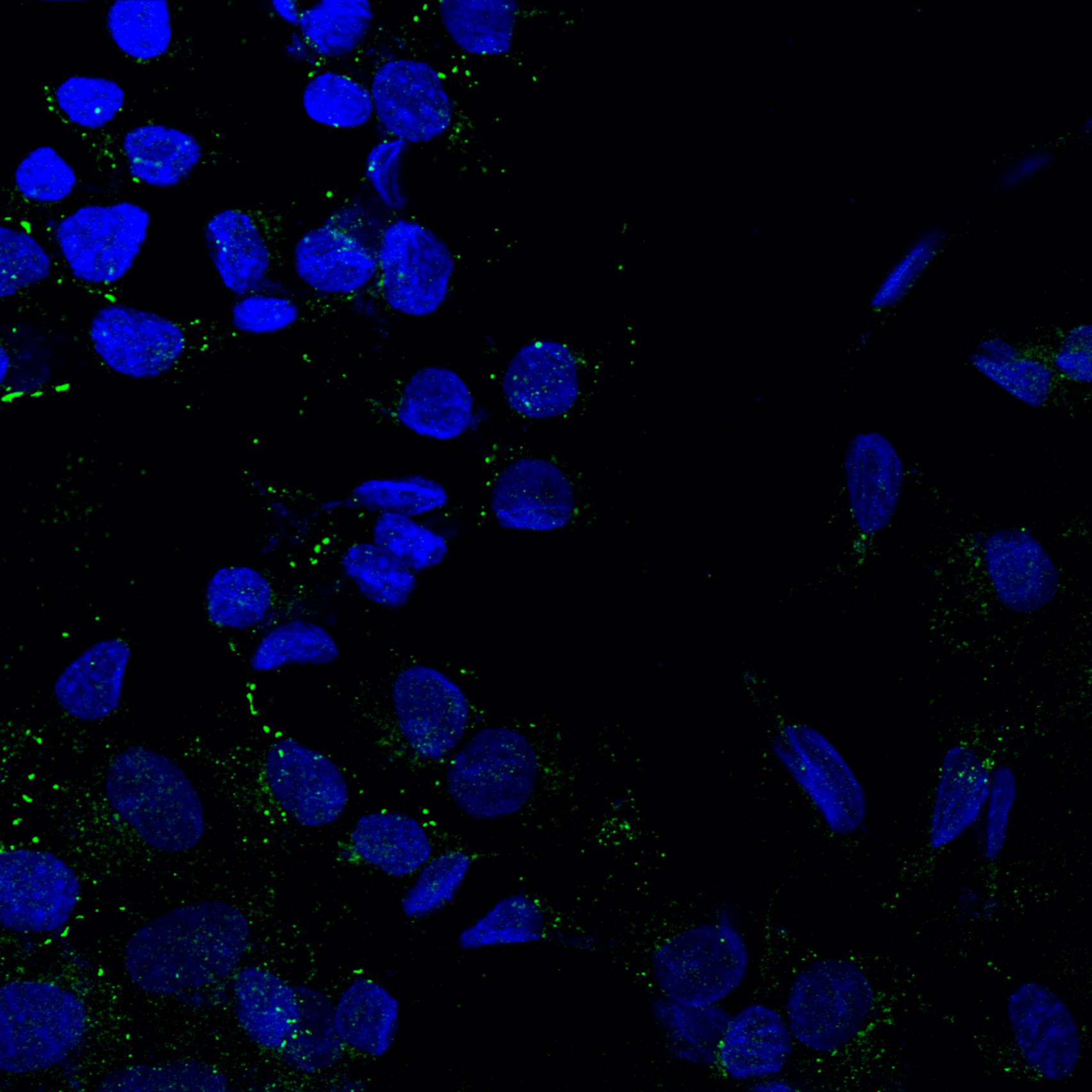

Supplement: Supplementary file 10 — Source data Fig. 5 [file 44319_2024_180_MOESM10_ESM.zip › Figure 5/5F/Lactate sistat3.tif]

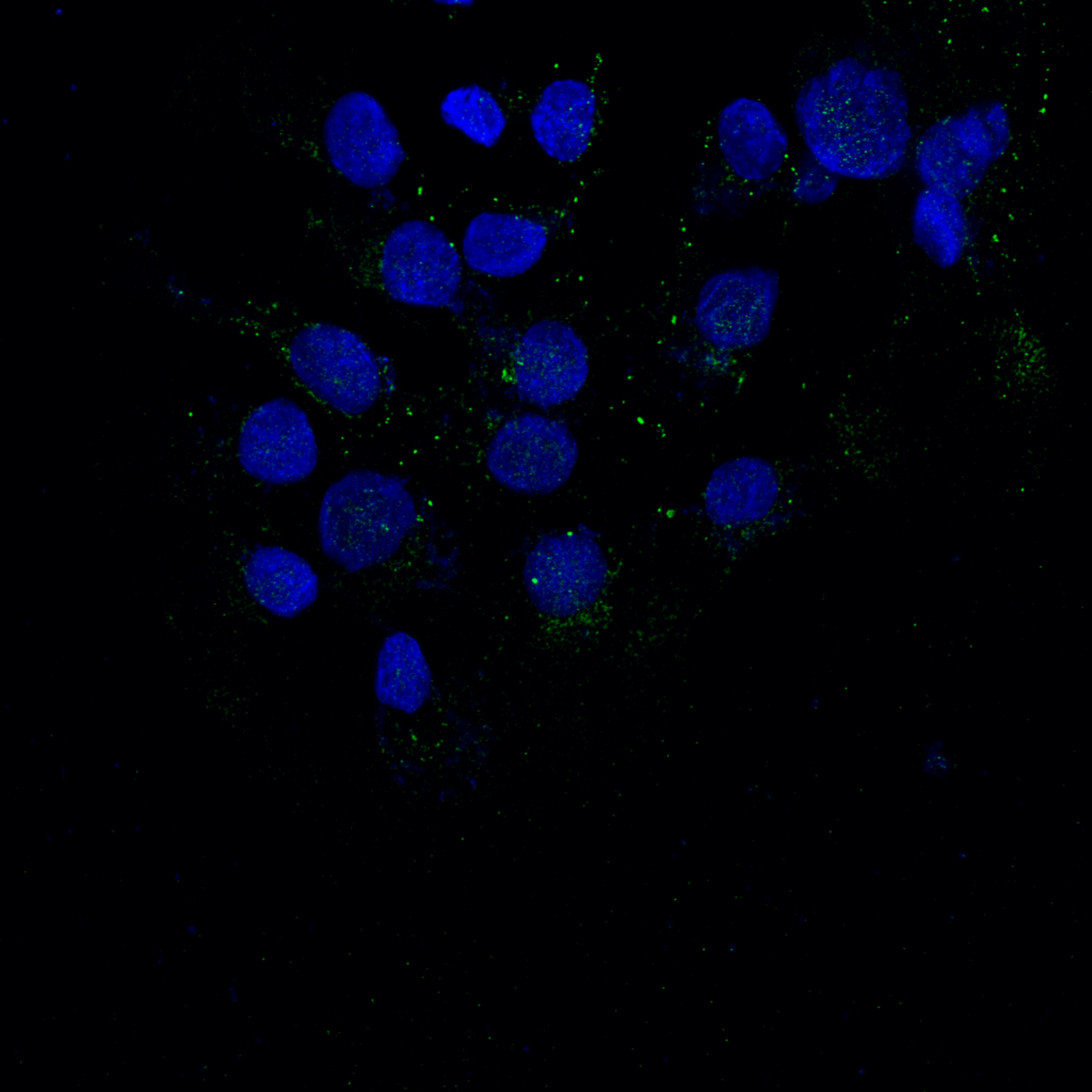

Supplement: Supplementary file 10 — Source data Fig. 5 [file 44319_2024_180_MOESM10_ESM.zip › Figure 5/5F/HPF-CM sistat3.tif]

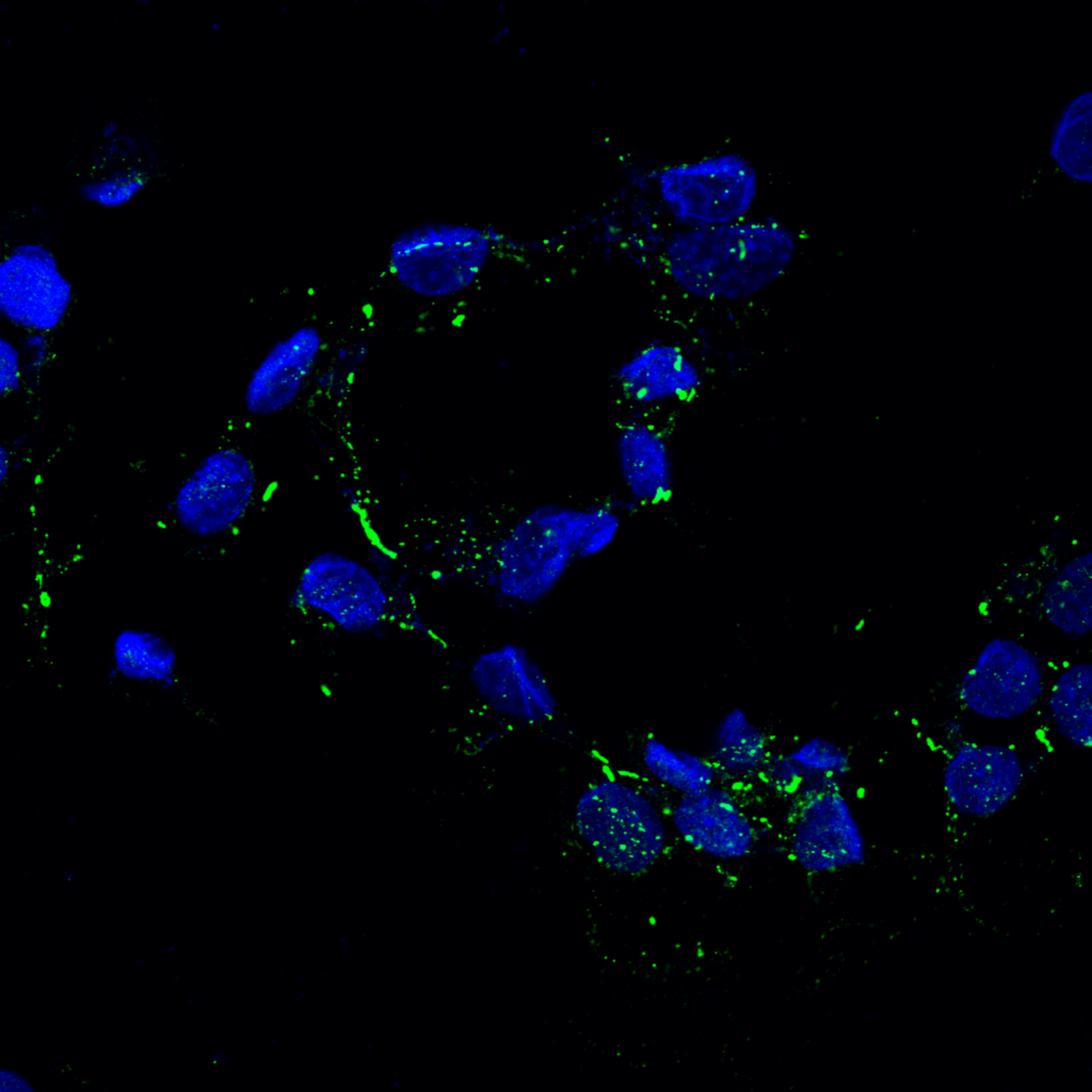

Supplement: Supplementary file 10 — Source data Fig. 5 [file 44319_2024_180_MOESM10_ESM.zip › Figure 5/5F/siCTR Lactate.tif]

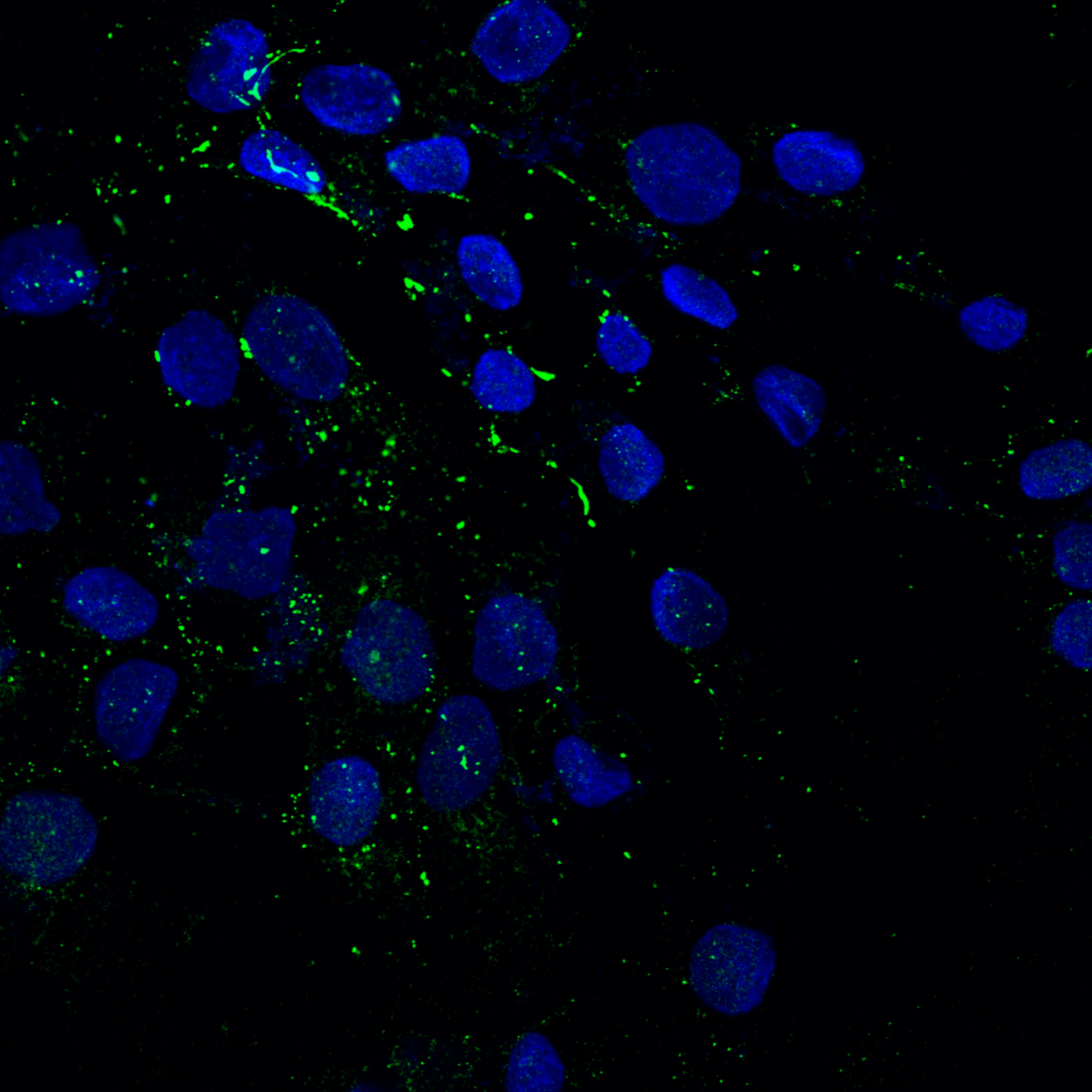

Supplement: Supplementary file 10 — Source data Fig. 5 [file 44319_2024_180_MOESM10_ESM.zip › Figure 5/5F/siCTR CAF-CM.tif]

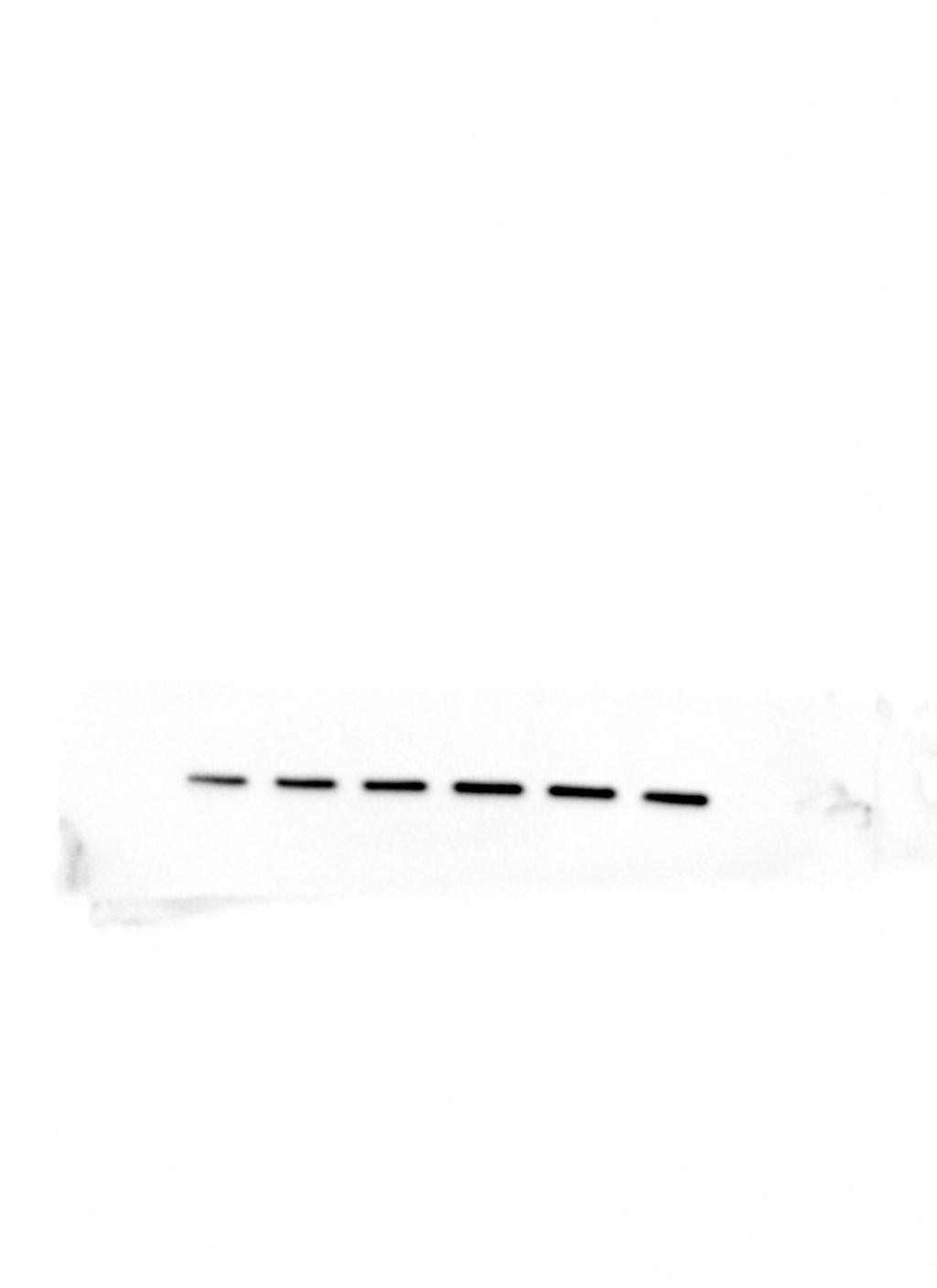

Supplement: Supplementary file 10 — Source data Fig. 5 [file 44319_2024_180_MOESM10_ESM.zip › Figure 5/5C/WB Actin ecm.tif]

Figure 5c

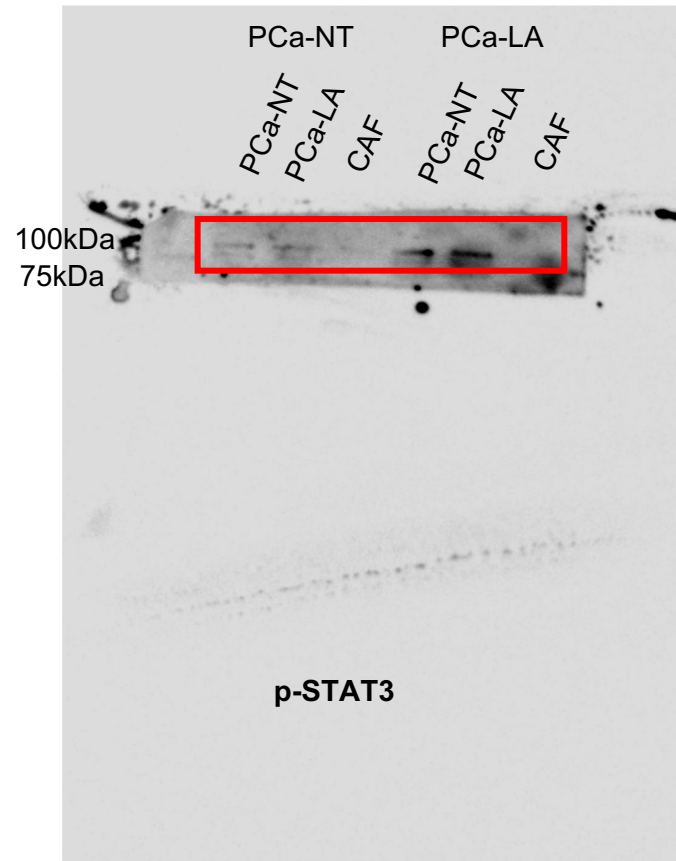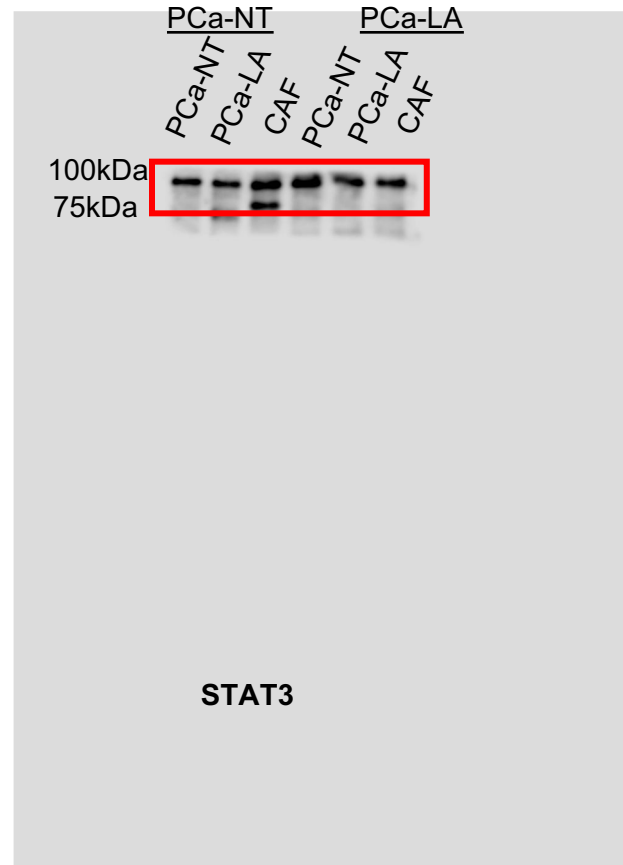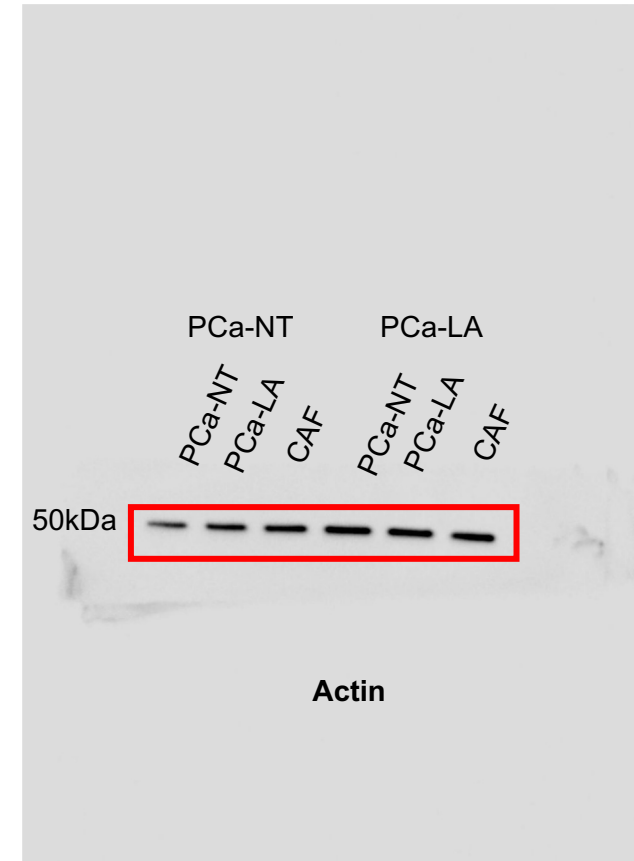

Supplement: Supplementary file 10 — Source data Fig. 5 [file 44319_2024_180_MOESM10_ESM.zip › Figure 5/5C/5c blot.pdf]

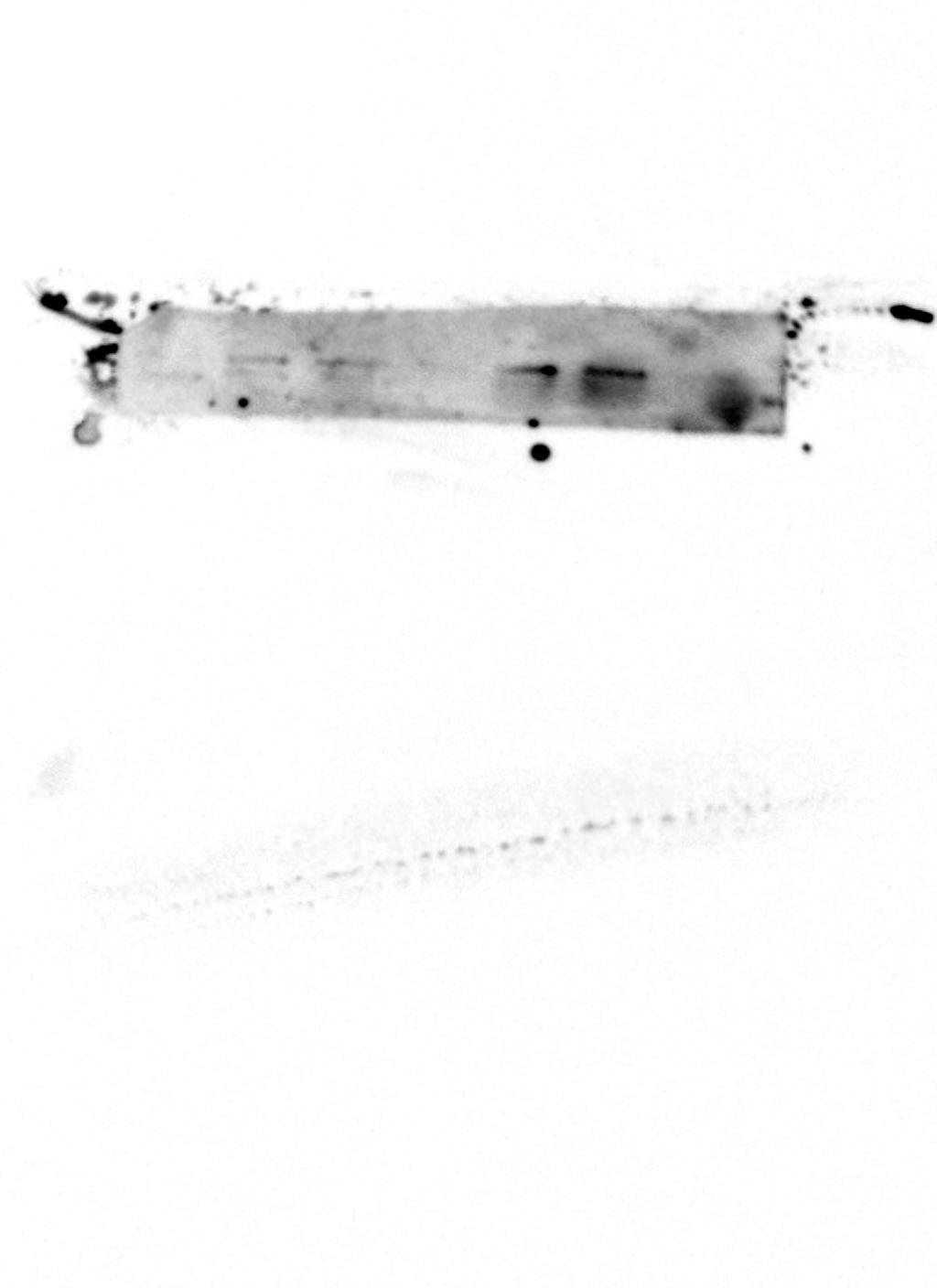

Supplement: Supplementary file 10 — Source data Fig. 5 [file 44319_2024_180_MOESM10_ESM.zip › Figure 5/5C/WB p-STAT3 ecm.tif]

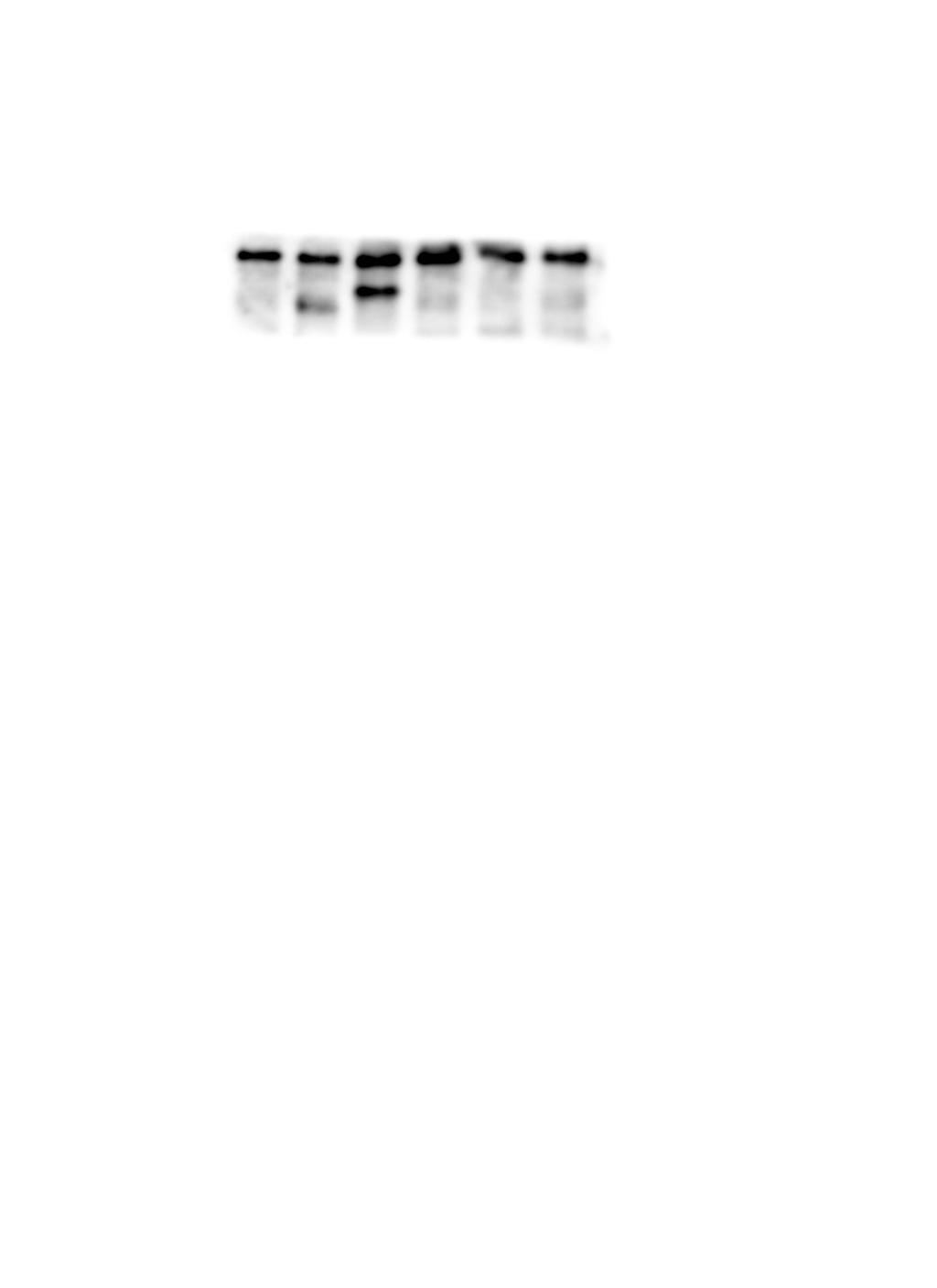

Supplement: Supplementary file 10 — Source data Fig. 5 [file 44319_2024_180_MOESM10_ESM.zip › Figure 5/5C/WB STAT3 ecm.tif]

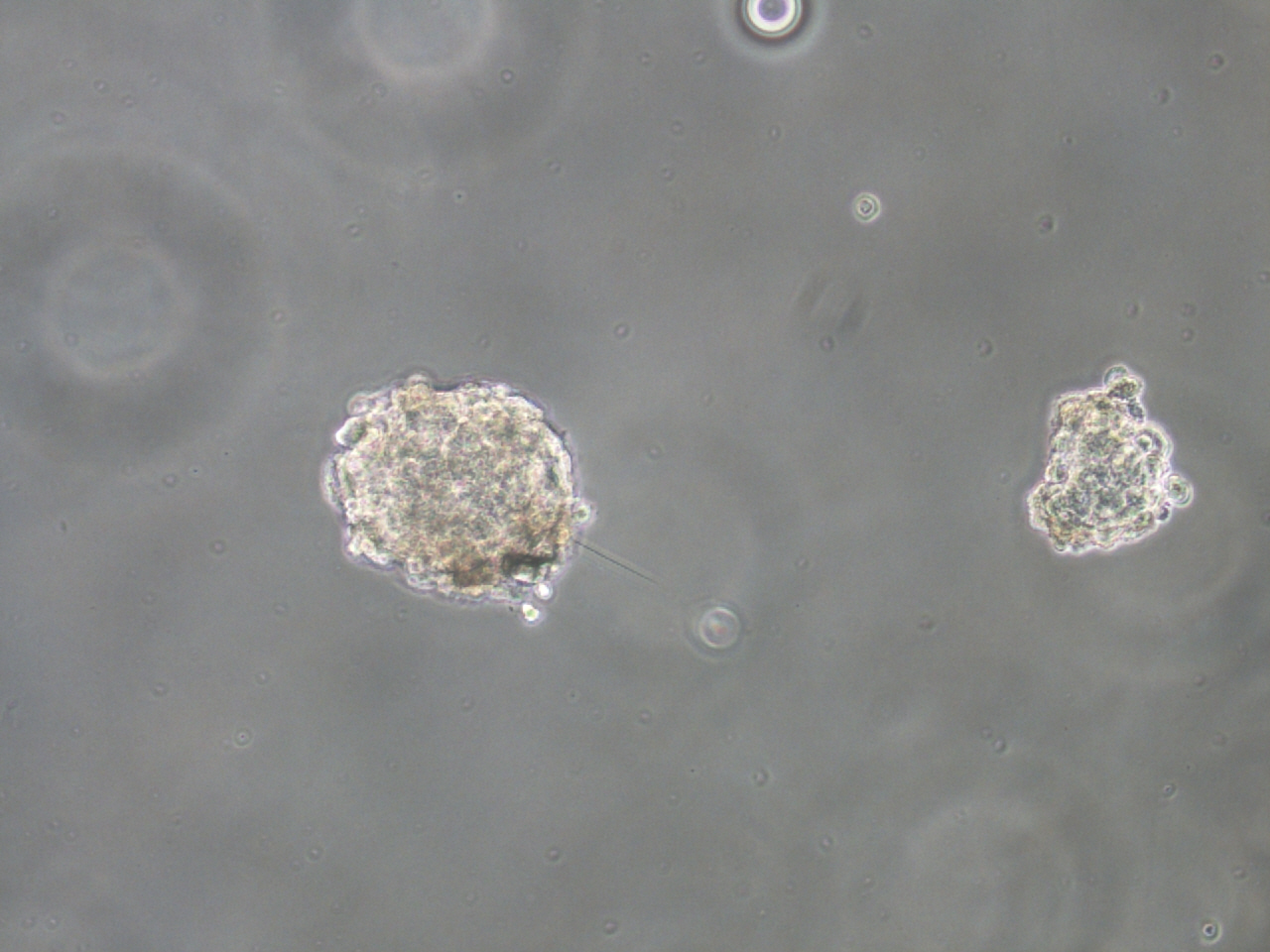

Supplement: Supplementary file 10 — Source data Fig. 5 [file 44319_2024_180_MOESM10_ESM.zip › Figure 5/5E/CAF-CM siSTAT3.tif]

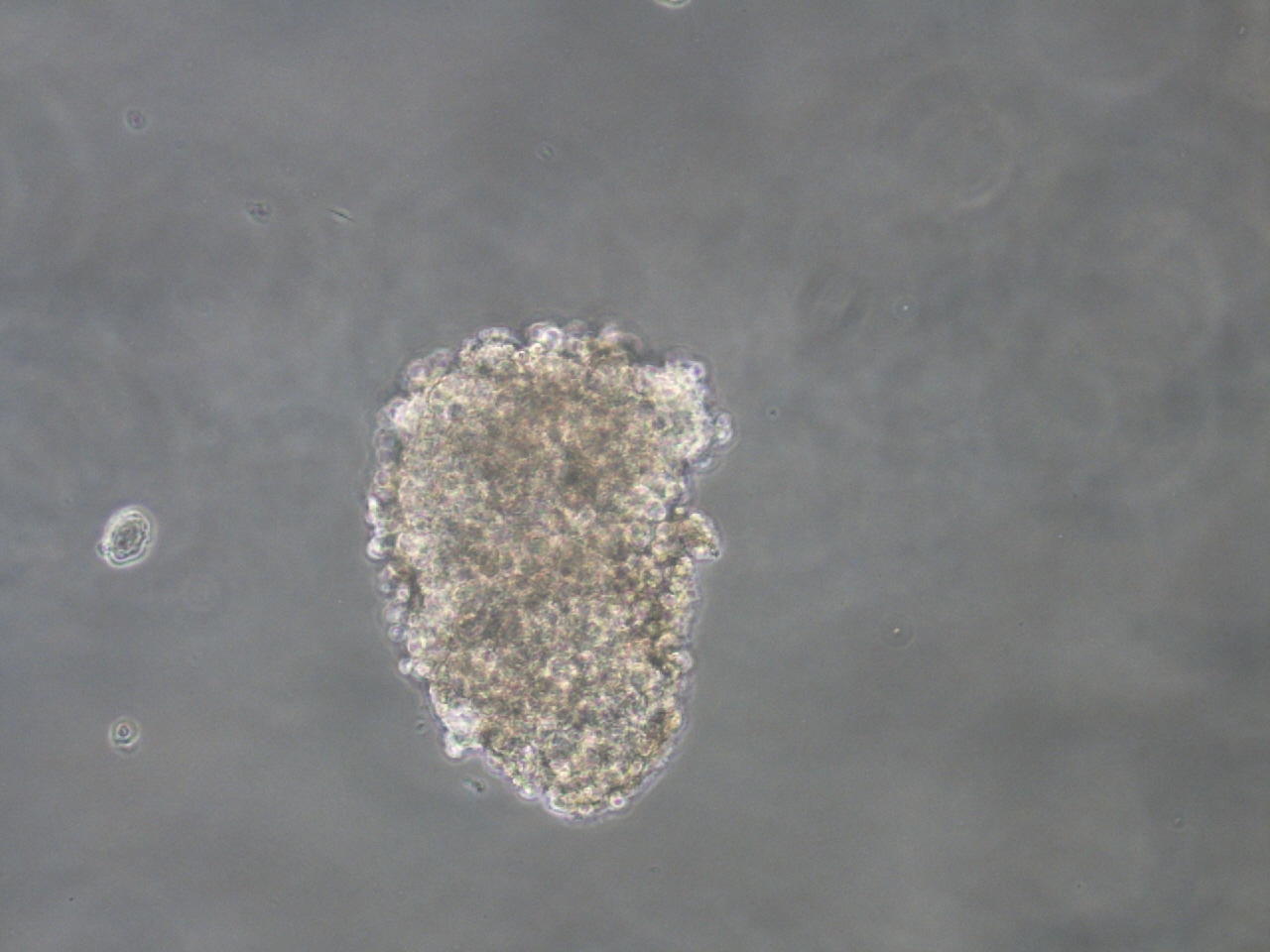

Supplement: Supplementary file 10 — Source data Fig. 5 [file 44319_2024_180_MOESM10_ESM.zip › Figure 5/5E/CAF-CM siCTR.tif]

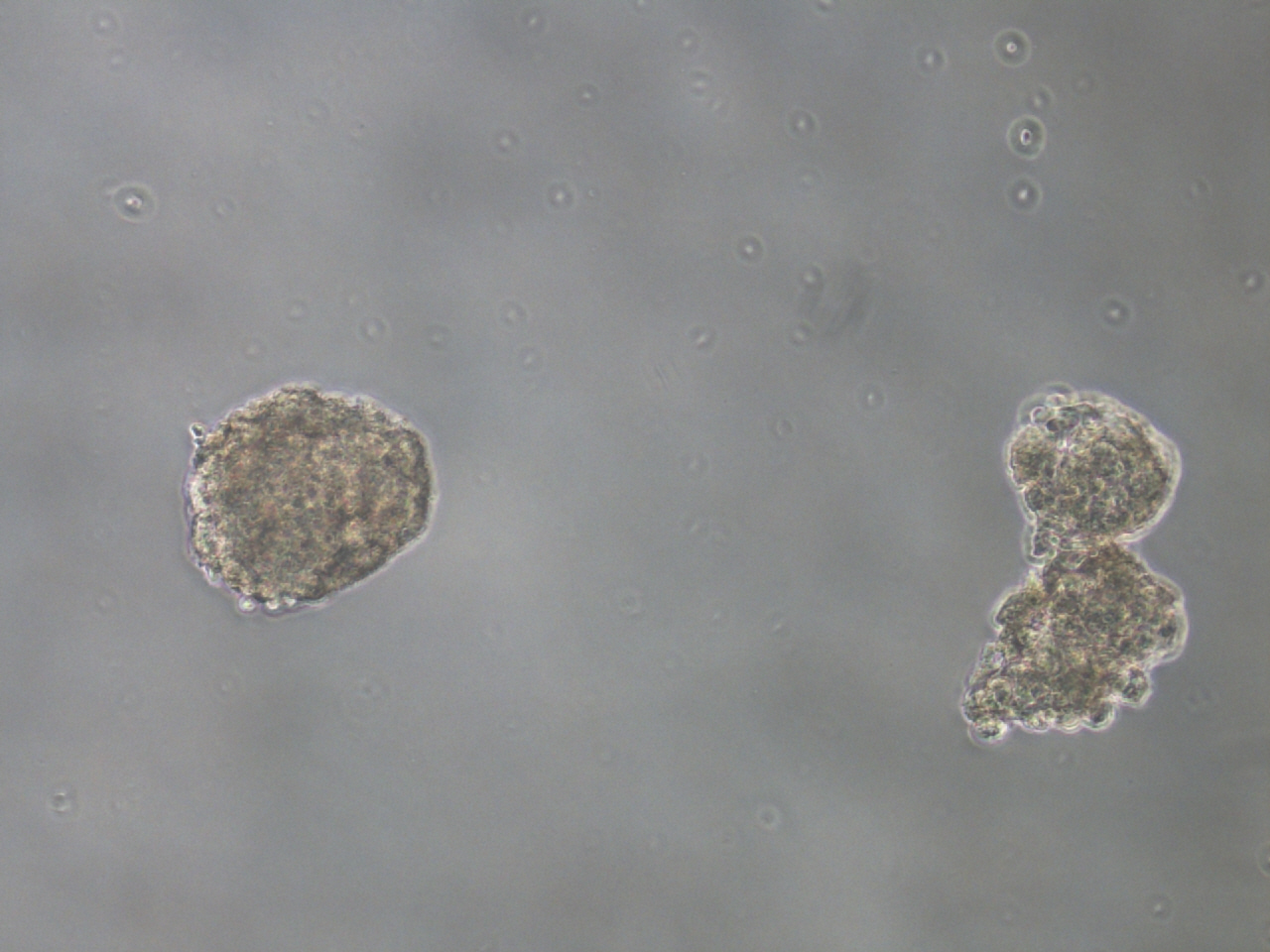

Supplement: Supplementary file 10 — Source data Fig. 5 [file 44319_2024_180_MOESM10_ESM.zip › Figure 5/5E/Lactate siSTAT3.tif]

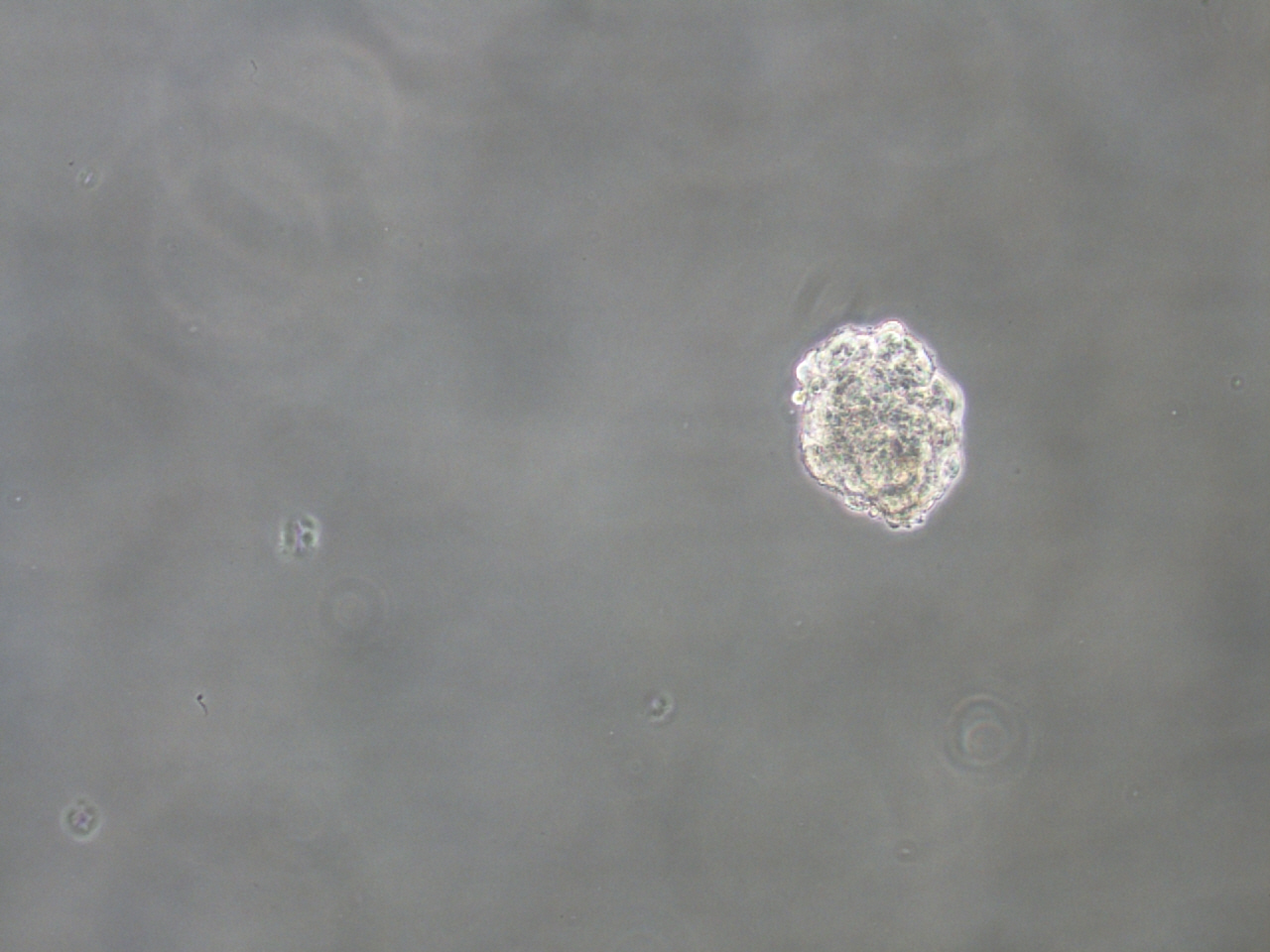

Supplement: Supplementary file 10 — Source data Fig. 5 [file 44319_2024_180_MOESM10_ESM.zip › Figure 5/5E/HPF-CM siCTR.tif]

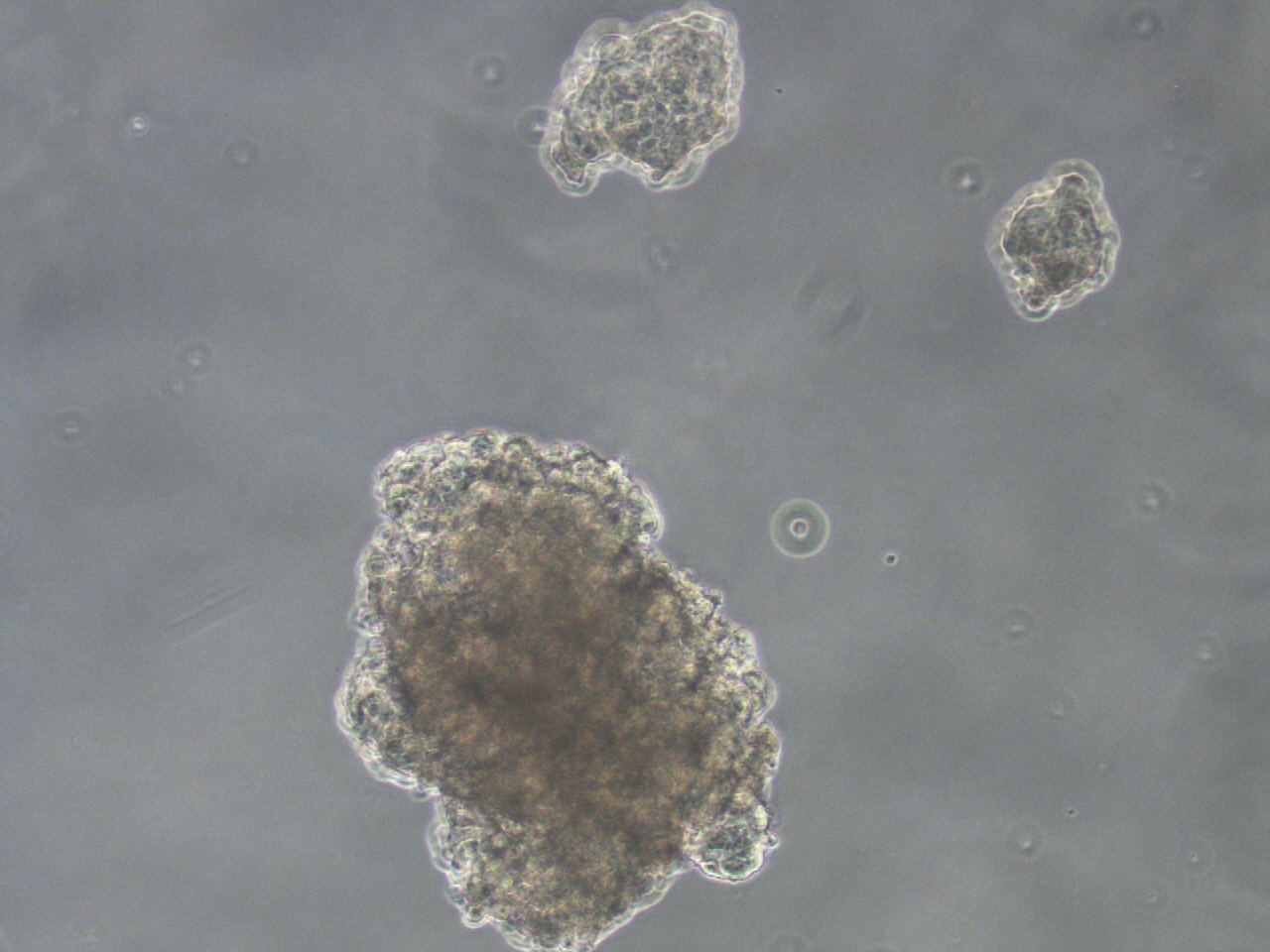

Supplement: Supplementary file 10 — Source data Fig. 5 [file 44319_2024_180_MOESM10_ESM.zip › Figure 5/5E/Lactate siCTR.tif]

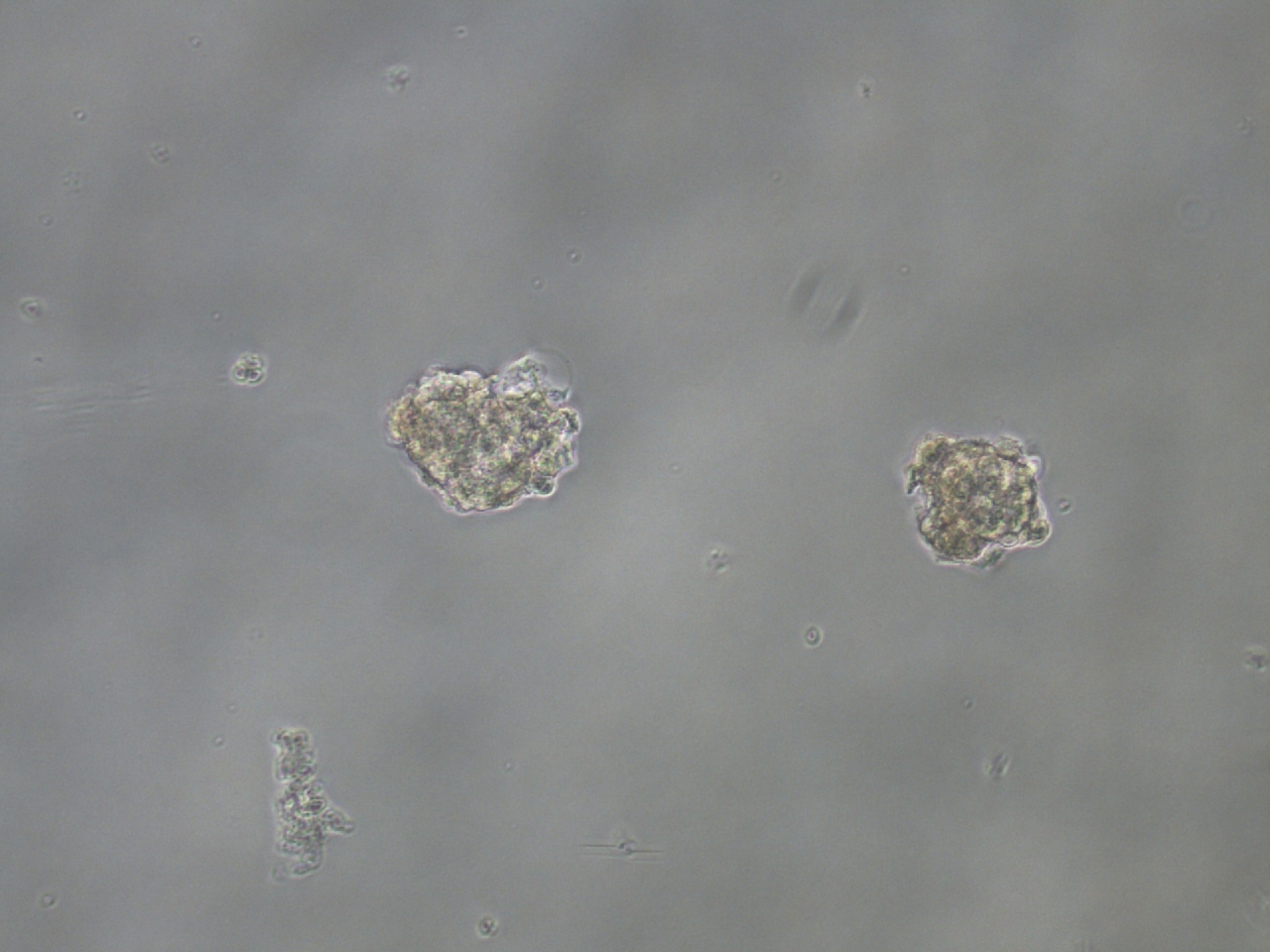

Supplement: Supplementary file 10 — Source data Fig. 5 [file 44319_2024_180_MOESM10_ESM.zip › Figure 5/5E/HPF-CM siSTAT3.tif]

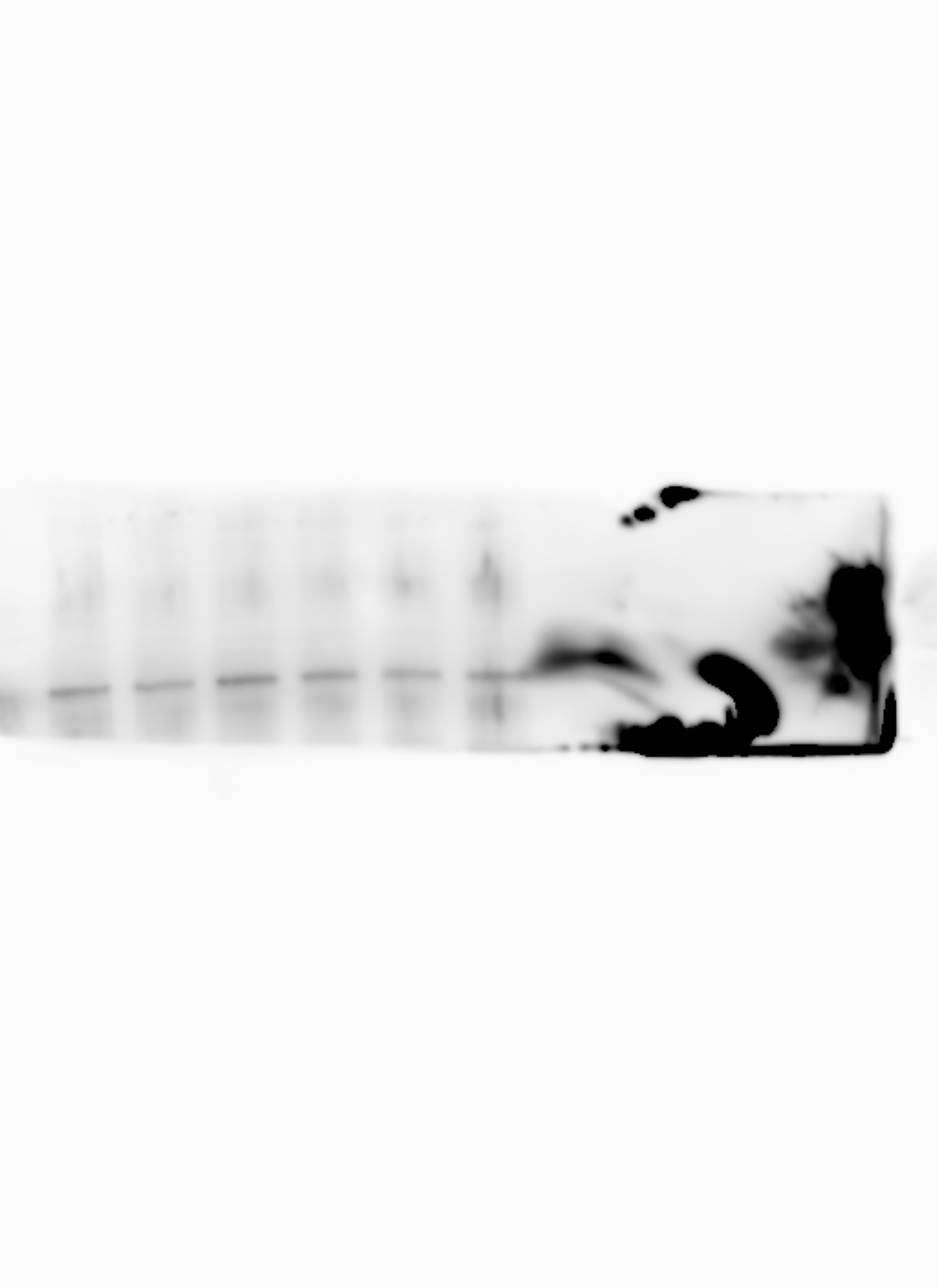

Supplement: Supplementary file 10 — Source data Fig. 5 [file 44319_2024_180_MOESM10_ESM.zip › Figure 5/5B/WB STAT3 siDDR1-1.tif]

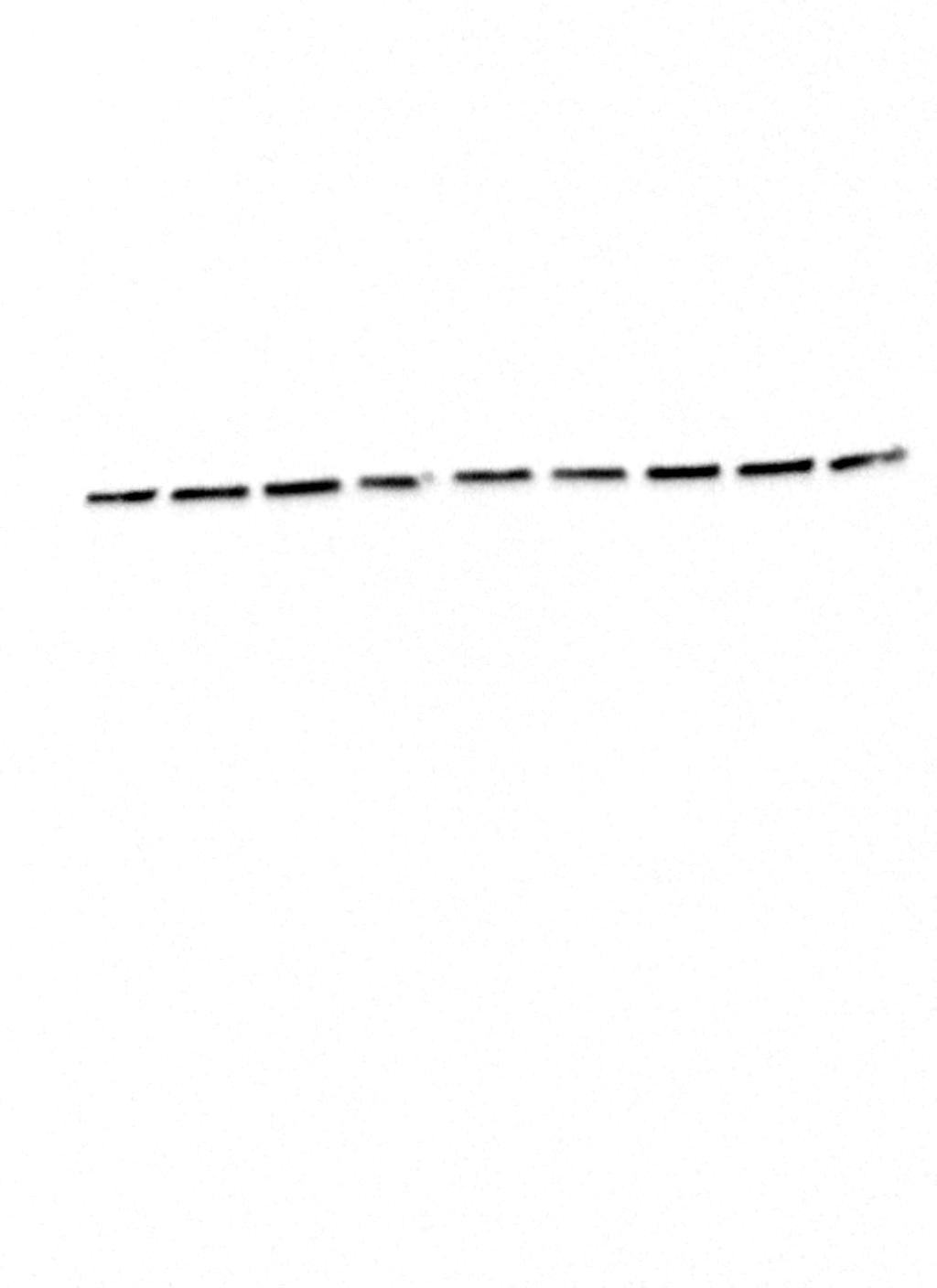

Supplement: Supplementary file 10 — Source data Fig. 5 [file 44319_2024_180_MOESM10_ESM.zip › Figure 5/5B/WB Actin siDDR1-1.tif]
