# Supplementary material for: Lactate supports cell-autonomous ECM production to sustain metastatic behavior in prostate cancer
Source: EMBO Rep. 2024 Jun 21;25(8):19. doi: 10.1038/s44319-024-00180-z (PMC11315984; doi:10.1038/s44319-024-00180-z)
Supplement: Supplementary file 6 — Source data Fig. 1 [file 44319_2024_180_MOESM6_ESM.zip › Figure 1/1A/REACTOME_COLLAGEN_BIOSYNTHESIS_AND_MODIFYING_ENZYMES.html]

Details for gene set REACTOME\_COLLAGEN\_BIOSYNTHESIS\_AND\_MODIFYING\_ENZYMES[GSEA]

|  || Dataset | LAvsCTRL\_all\_sig\_stat.rnk\_remapped |
| Phenotype | NoPhenotypeAvailable |
| Upregulated in class | na\_pos |
| GeneSet | REACTOME\_COLLAGEN\_BIOSYNTHESIS\_AND\_MODIFYING\_ENZYMES |
| Enrichment Score (ES) | 0.549597 |
| Normalized Enrichment Score (NES) | 2.471465 |
| Nominal p-value | 0.0 |
| FDR q-value | 9.0011535E-4 |
| FWER p-Value | 0.009 |
Table: GSEA Results Summary

  

Fig 1: Enrichment plot: REACTOME\_COLLAGEN\_BIOSYNTHESIS\_AND\_MODIFYING\_ENZYMES      
 Profile of the Running ES Score & Positions of GeneSet Members on the Rank Ordered List

  

| SYMBOL | TITLE | RANK IN GENE LIST | RANK METRIC SCORE | RUNNING ES | CORE ENRICHMENT || 1 | P4HA1 | prolyl 4-hydroxylase subunit alpha 1 [Source:HGNC Symbol;Acc:HGNC:8546] | 34 | 7.606 | 0.0729 | Yes |
| 2 | COL6A2 | collagen type VI alpha 2 chain [Source:HGNC Symbol;Acc:HGNC:2212] | 37 | 7.576 | 0.1533 | Yes |
| 3 | COL8A2 | collagen type VIII alpha 2 chain [Source:HGNC Symbol;Acc:HGNC:2216] | 117 | 6.369 | 0.2020 | Yes |
| 4 | P4HB | prolyl 4-hydroxylase subunit beta [Source:HGNC Symbol;Acc:HGNC:8548] | 175 | 5.833 | 0.2503 | Yes |
| 5 | P4HA2 | prolyl 4-hydroxylase subunit alpha 2 [Source:HGNC Symbol;Acc:HGNC:8547] | 194 | 5.746 | 0.3073 | Yes |
| 6 | COL6A1 | collagen type VI alpha 1 chain [Source:HGNC Symbol;Acc:HGNC:2211] | 237 | 5.493 | 0.3557 | Yes |
| 7 | P3H3 | prolyl 3-hydroxylase 3 [Source:HGNC Symbol;Acc:HGNC:19318] | 390 | 4.895 | 0.3707 | Yes |
| 8 | PLOD1 | procollagen-lysine,2-oxoglutarate 5-dioxygenase 1 [Source:HGNC Symbol;Acc:HGNC:9081] | 398 | 4.863 | 0.4210 | Yes |
| 9 | COL18A1 | collagen type XVIII alpha 1 chain [Source:HGNC Symbol;Acc:HGNC:2195] | 422 | 4.812 | 0.4667 | Yes |
| 10 | P4HA3 | prolyl 4-hydroxylase subunit alpha 3 [Source:HGNC Symbol;Acc:HGNC:30135] | 448 | 4.752 | 0.5113 | Yes |
| 11 | BMP1 | bone morphogenetic protein 1 [Source:HGNC Symbol;Acc:HGNC:1067] | 729 | 4.119 | 0.4867 | Yes |
| 12 | COL9A2 | collagen type IX alpha 2 chain [Source:HGNC Symbol;Acc:HGNC:2218] | 779 | 4.033 | 0.5178 | Yes |
| 13 | COL1A1 | collagen type I alpha 1 chain [Source:HGNC Symbol;Acc:HGNC:2197] | 823 | 3.963 | 0.5496 | Yes |
| 14 | COL13A1 | collagen type XIII alpha 1 chain [Source:HGNC Symbol;Acc:HGNC:2190] | 1183 | 3.455 | 0.4986 | No |
| 15 | PCOLCE | procollagen C-endopeptidase enhancer [Source:HGNC Symbol;Acc:HGNC:8738] | 1567 | 3.057 | 0.4374 | No |
| 16 | PLOD3 | procollagen-lysine,2-oxoglutarate 5-dioxygenase 3 [Source:HGNC Symbol;Acc:HGNC:9083] | 1587 | 3.039 | 0.4652 | No |
| 17 | SERPINH1 | serpin family H member 1 [Source:HGNC Symbol;Acc:HGNC:1546] | 1634 | 2.998 | 0.4859 | No |
| 18 | COL4A2 | collagen type IV alpha 2 chain [Source:HGNC Symbol;Acc:HGNC:2203] | 1934 | 2.723 | 0.4418 | No |
| 19 | COLGALT1 | collagen beta(1-O)galactosyltransferase 1 [Source:HGNC Symbol;Acc:HGNC:26182] | 1944 | 2.714 | 0.4686 | No |
| 20 | COL24A1 | collagen type XXIV alpha 1 chain [Source:HGNC Symbol;Acc:HGNC:20821] | 2573 | -2.653 | 0.3430 | No |
| 21 | COL7A1 | collagen type VII alpha 1 chain [Source:HGNC Symbol;Acc:HGNC:2214] | 2881 | -2.930 | 0.2991 | No |
Table: GSEA details [plain text format]

  

Fig 2: REACTOME\_COLLAGEN\_BIOSYNTHESIS\_AND\_MODIFYING\_ENZYMES: Random ES distribution      
 Gene set null distribution of ES for **REACTOME\_COLLAGEN\_BIOSYNTHESIS\_AND\_MODIFYING\_ENZYMES**

  
