# Supplementary material for: Lactate supports cell-autonomous ECM production to sustain metastatic behavior in prostate cancer
Source: EMBO Rep. 2024 Jun 21;25(8):19. doi: 10.1038/s44319-024-00180-z (PMC11315984; doi:10.1038/s44319-024-00180-z)
Supplement: Supplementary file 7 — Source data Fig. 2 [file 44319_2024_180_MOESM7_ESM.zip › Figure 2/2B/2B.rtf]

The images in the Figure 2B were rotated 90° on left. 	
