# Supplementary material for: Lactate supports cell-autonomous ECM production to sustain metastatic behavior in prostate cancer
Source: EMBO Rep. 2024 Jun 21;25(8):19. doi: 10.1038/s44319-024-00180-z (PMC11315984; doi:10.1038/s44319-024-00180-z)
Supplement: Supplementary file 8 — Source data Fig. 3 [file 44319_2024_180_MOESM8_ESM.zip › Figure 3/3G/3G.rtf]

The images in the Figure 3G were rotated 90° on left. 	
