# Supplementary figures and images for: Lactate supports cell-autonomous ECM production to sustain metastatic behavior in prostate cancer (part 3 of 3)
Source: EMBO Rep. 2024 Jun 21;25(8):19. doi: 10.1038/s44319-024-00180-z (PMC11315984; doi:10.1038/s44319-024-00180-z)

Figure 5b

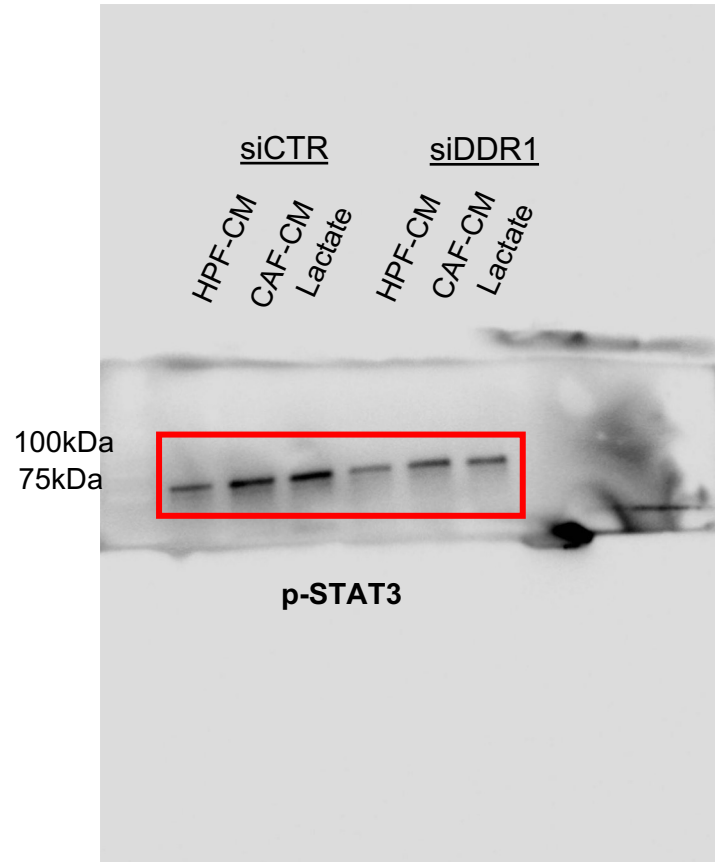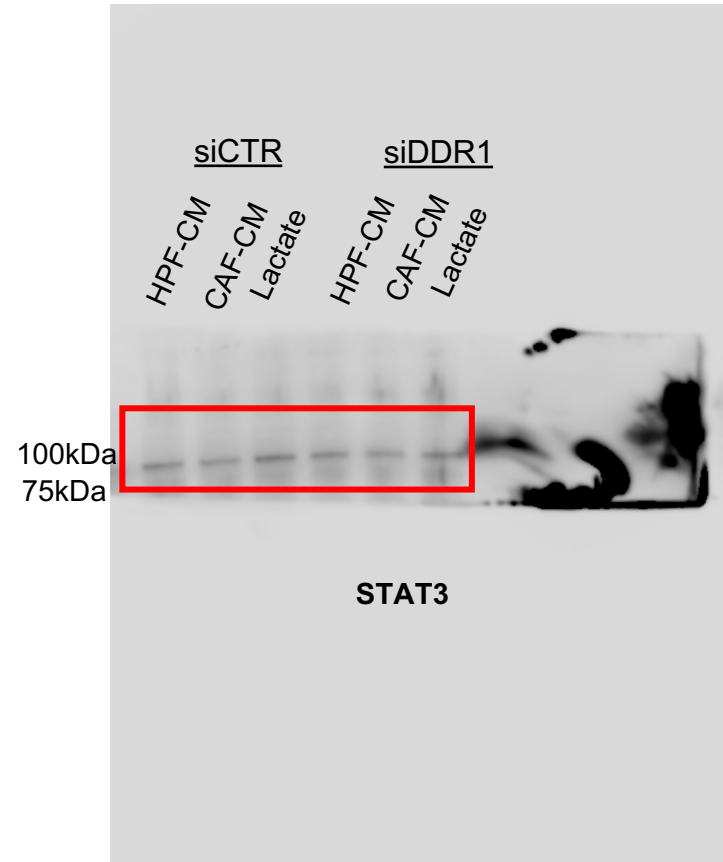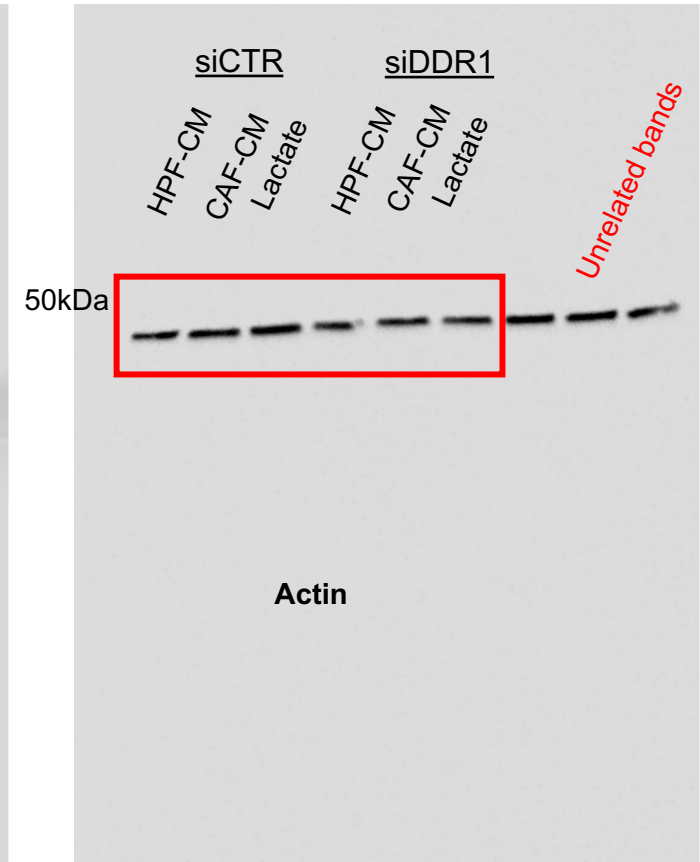

Supplement: Supplementary file 10 — Source data Fig. 5 [file 44319_2024_180_MOESM10_ESM.zip › Figure 5/5B/5b blot.pdf]

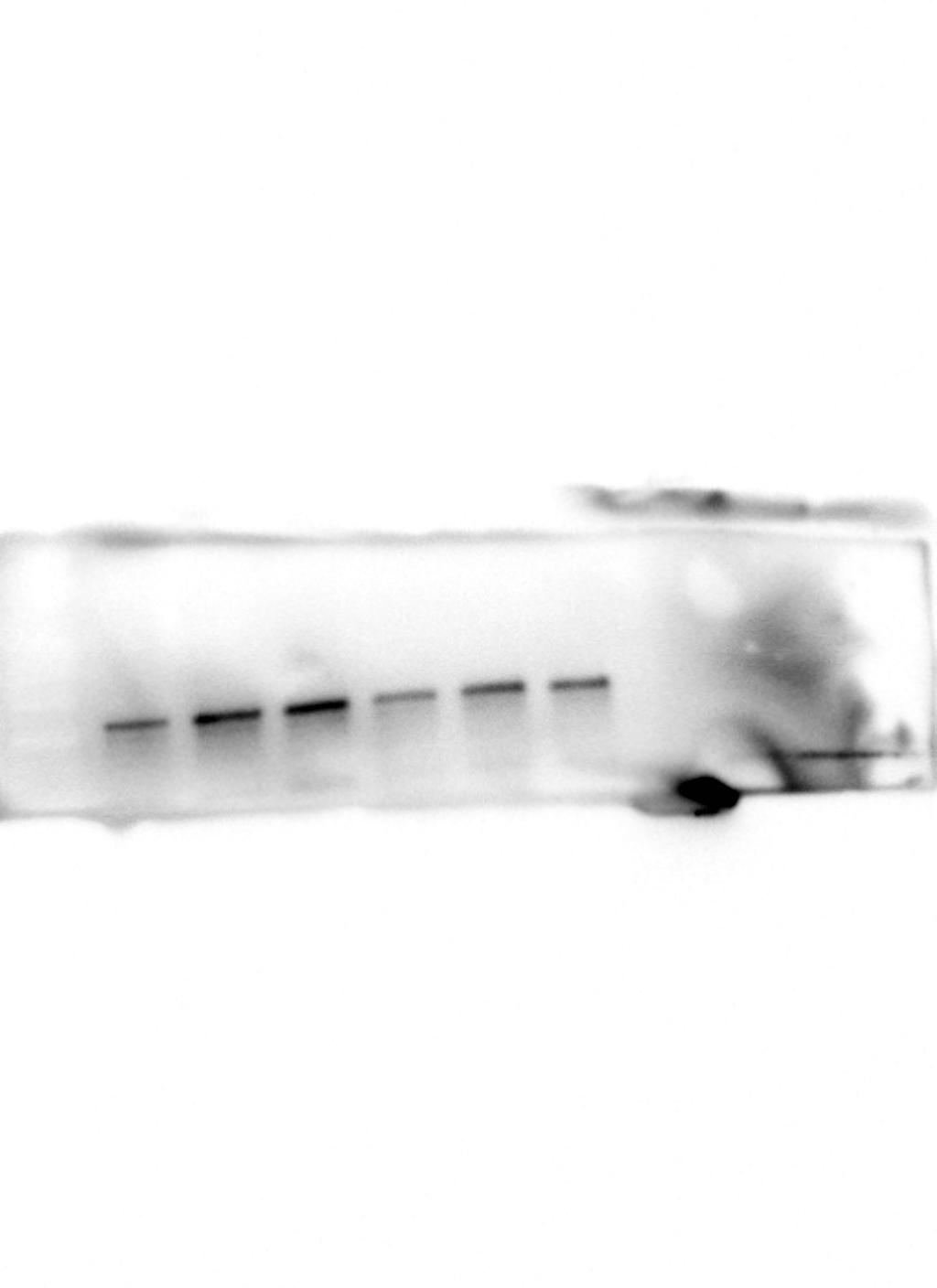

Supplement: Supplementary file 10 — Source data Fig. 5 [file 44319_2024_180_MOESM10_ESM.zip › Figure 5/5B/WB p-STAT3 siDDR1-1.tif]

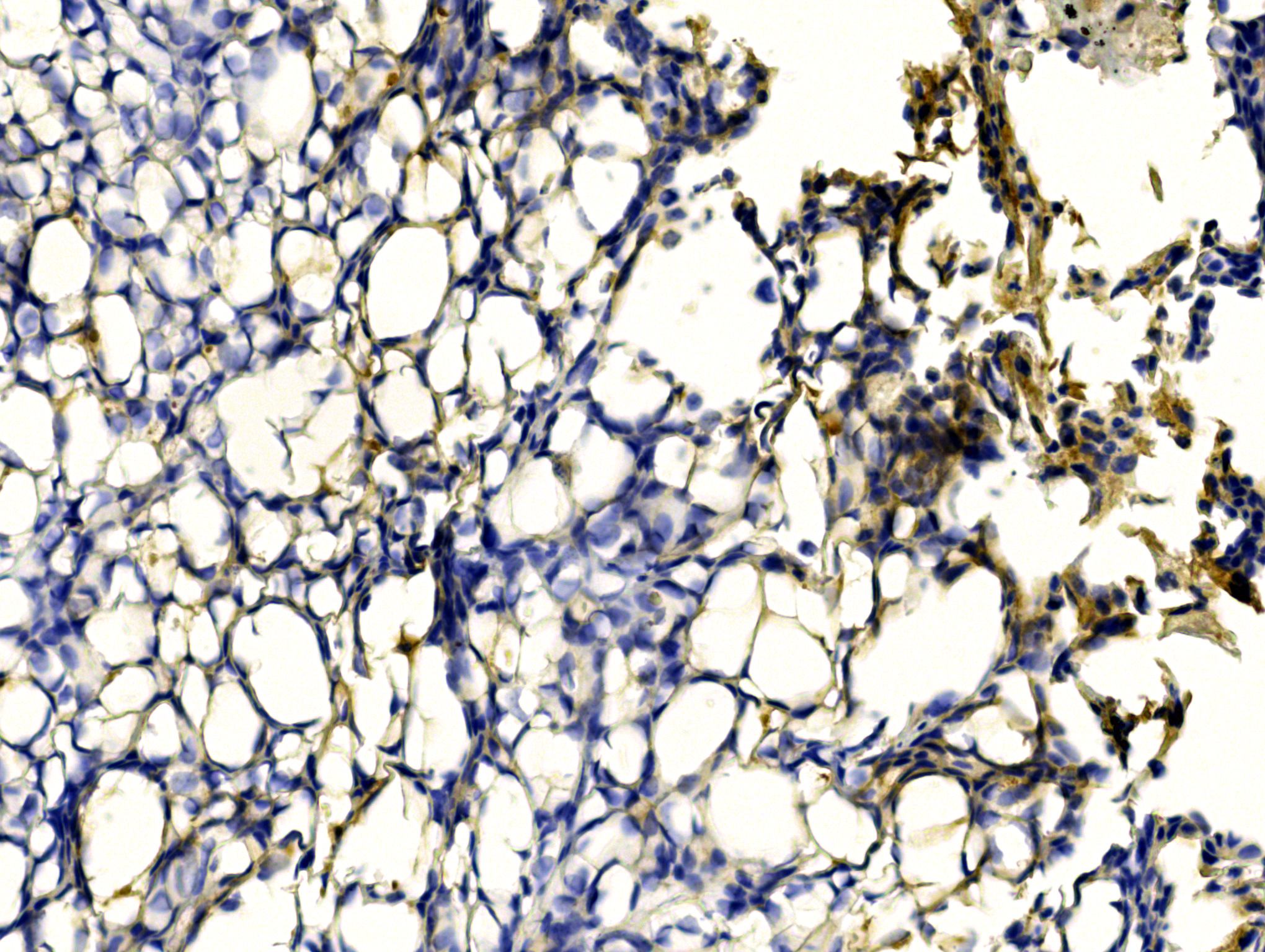

Supplement: Supplementary file 11 — Source data Fig. 6 [file 44319_2024_180_MOESM11_ESM.zip › Figure 6/6A/CAF Scr.tif]

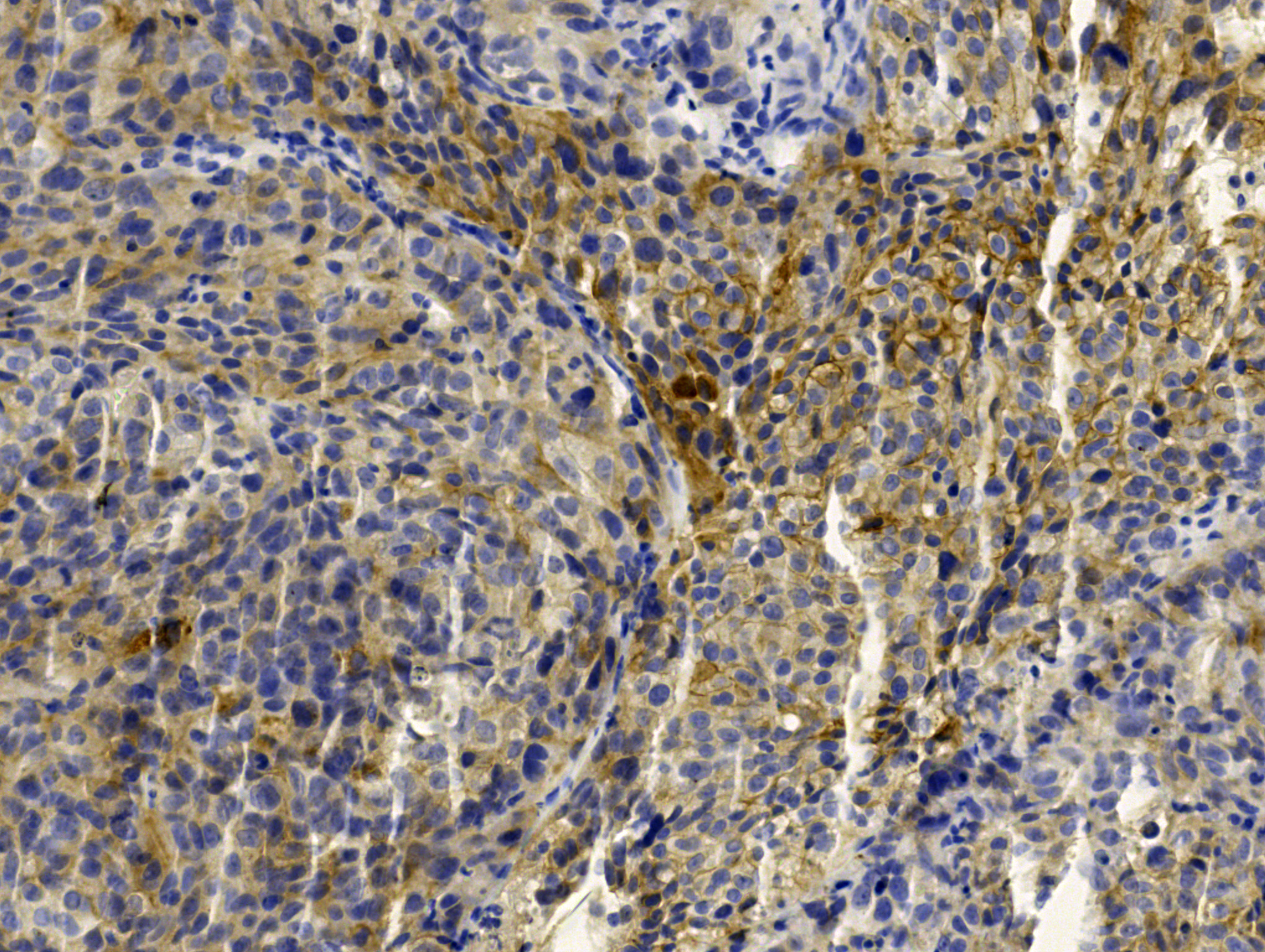

Supplement: Supplementary file 11 — Source data Fig. 6 [file 44319_2024_180_MOESM11_ESM.zip › Figure 6/6A/Lactate Scr.tif]

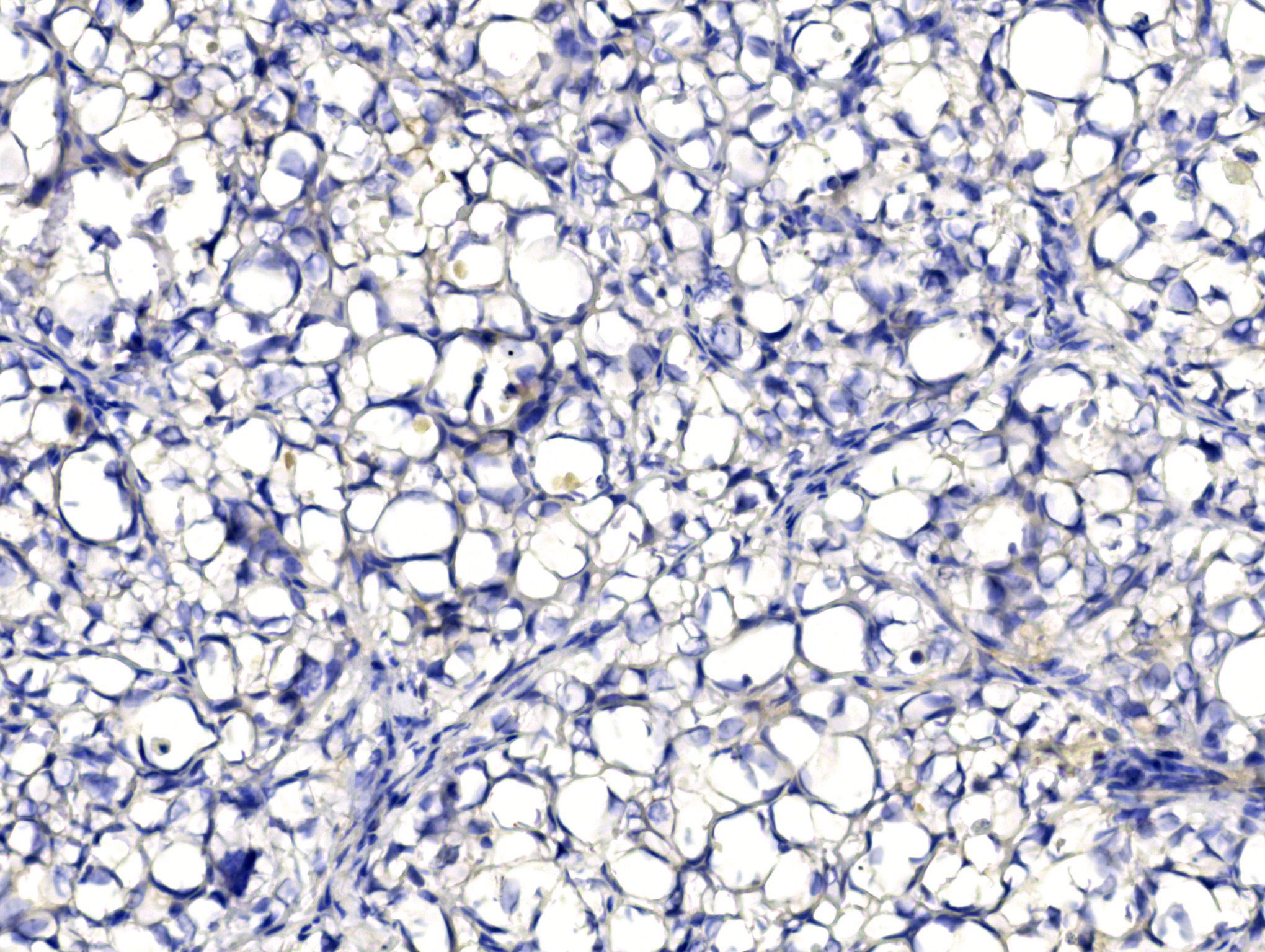

Supplement: Supplementary file 11 — Source data Fig. 6 [file 44319_2024_180_MOESM11_ESM.zip › Figure 6/6A/HPF shDDR1.tif]

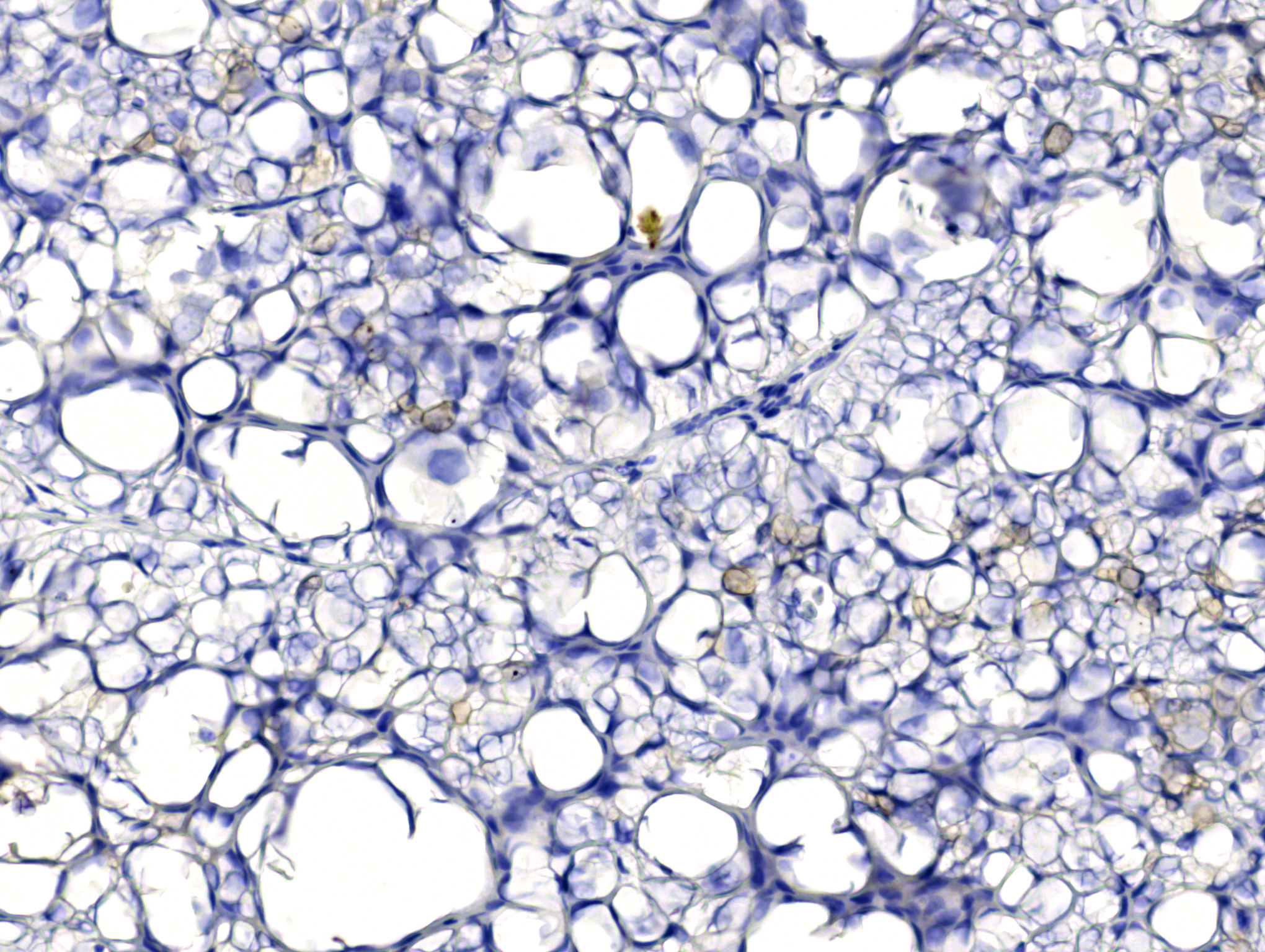

Supplement: Supplementary file 11 — Source data Fig. 6 [file 44319_2024_180_MOESM11_ESM.zip › Figure 6/6A/CAF shDDR1.tif]

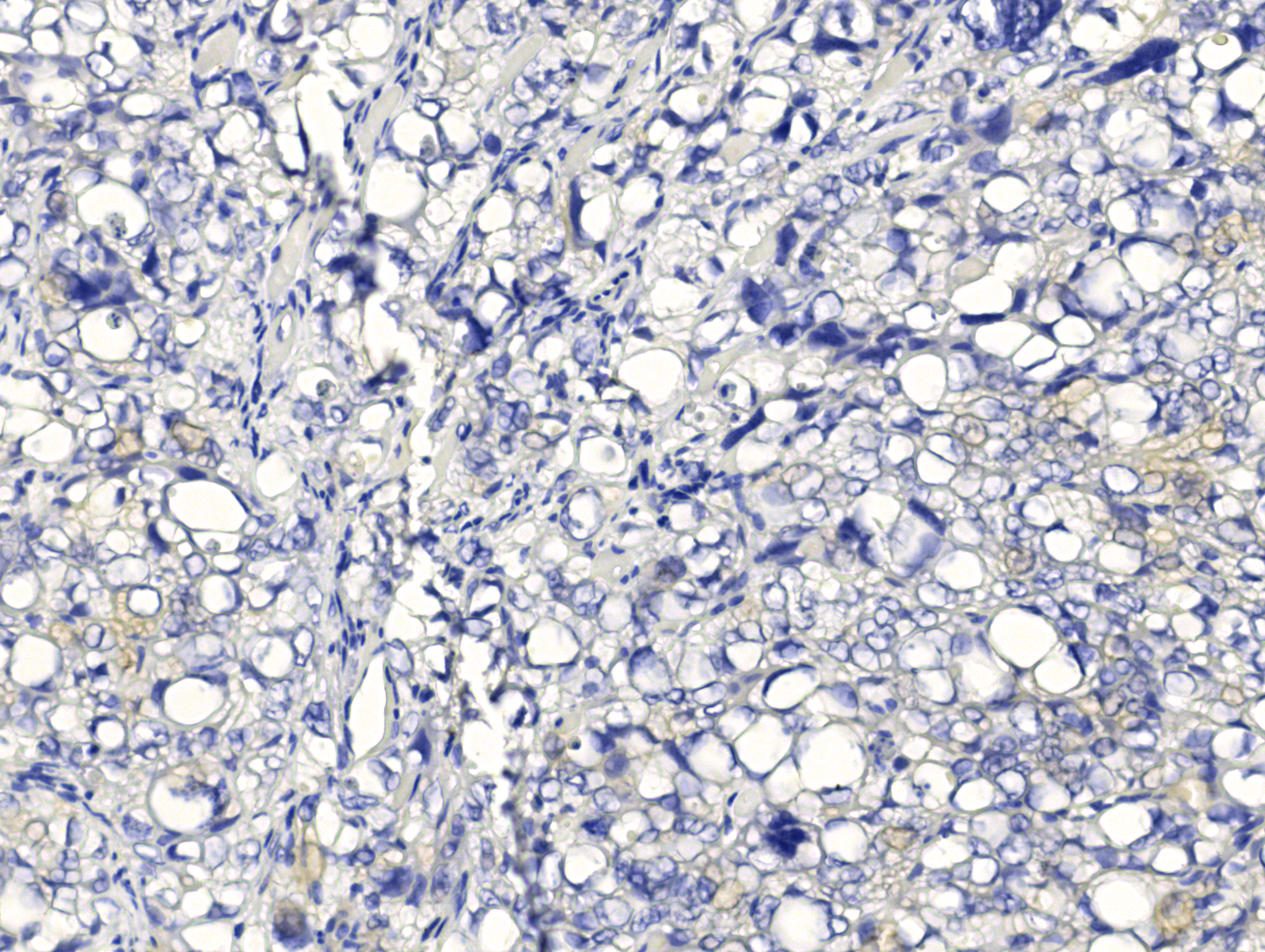

Supplement: Supplementary file 11 — Source data Fig. 6 [file 44319_2024_180_MOESM11_ESM.zip › Figure 6/6A/Lactate shDDR1.tif]

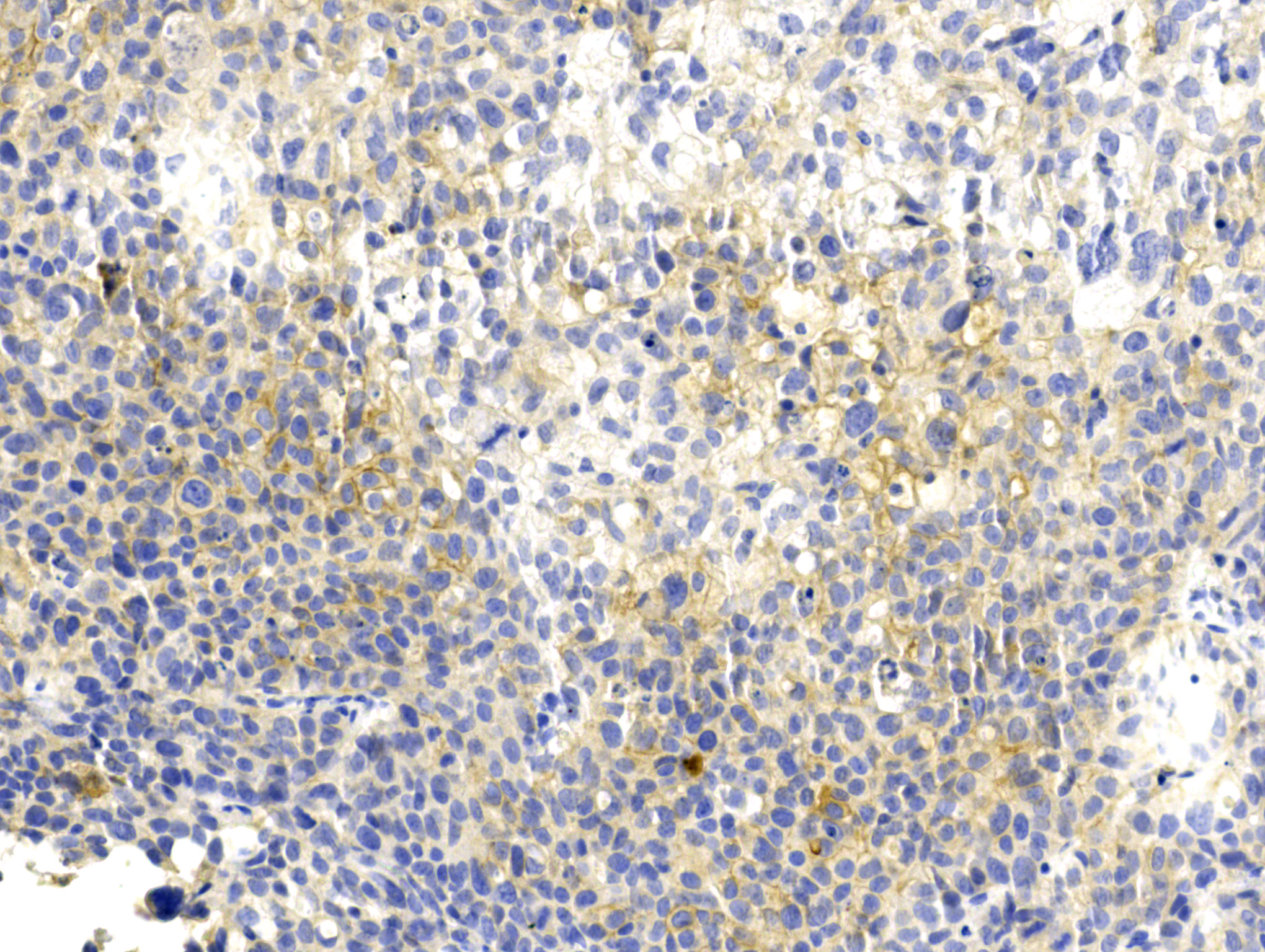

Supplement: Supplementary file 11 — Source data Fig. 6 [file 44319_2024_180_MOESM11_ESM.zip › Figure 6/6A/HPF Scr.tif]

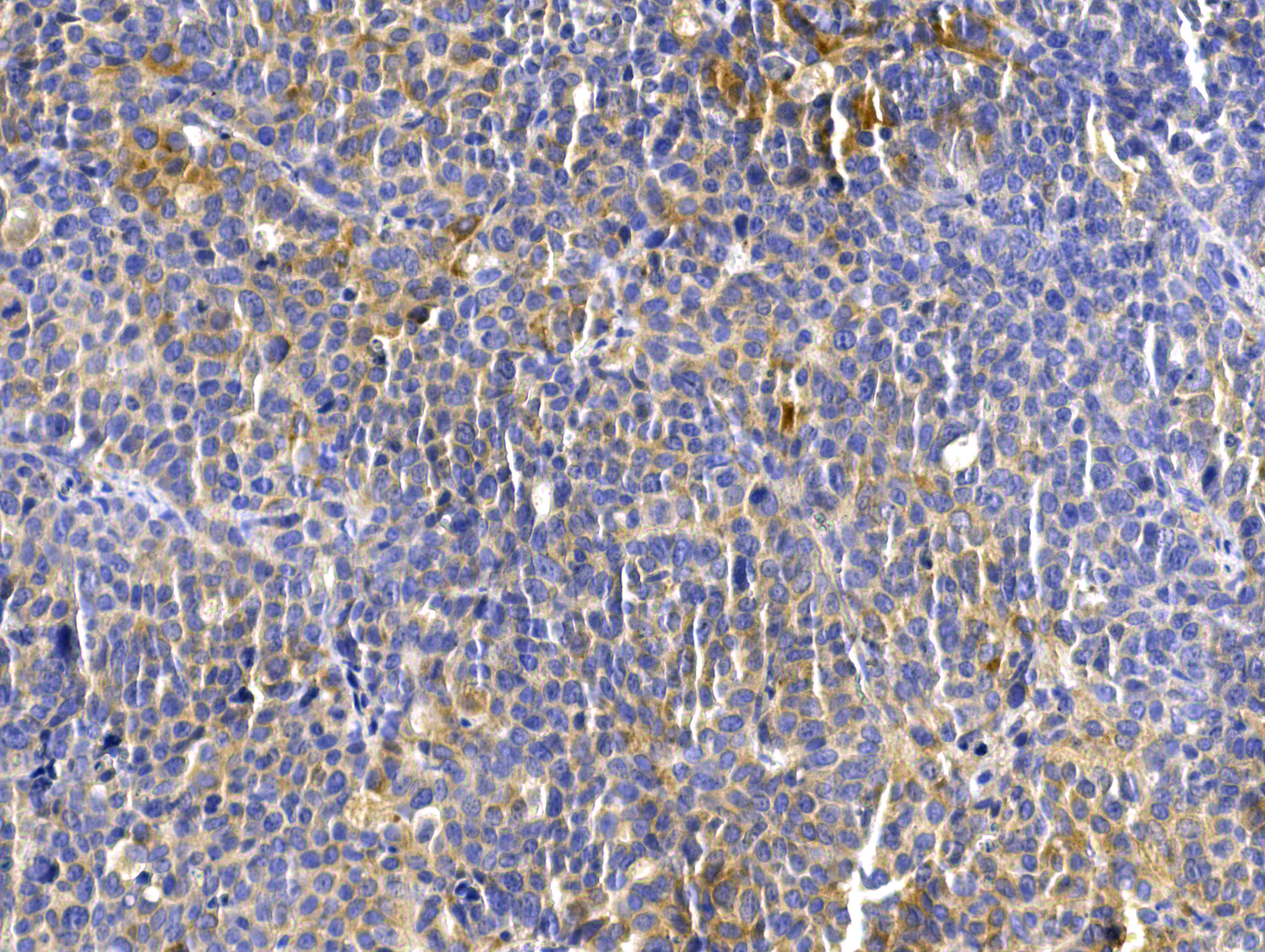

Supplement: Supplementary file 11 — Source data Fig. 6 [file 44319_2024_180_MOESM11_ESM.zip › Figure 6/6B/scr HPF.tif]

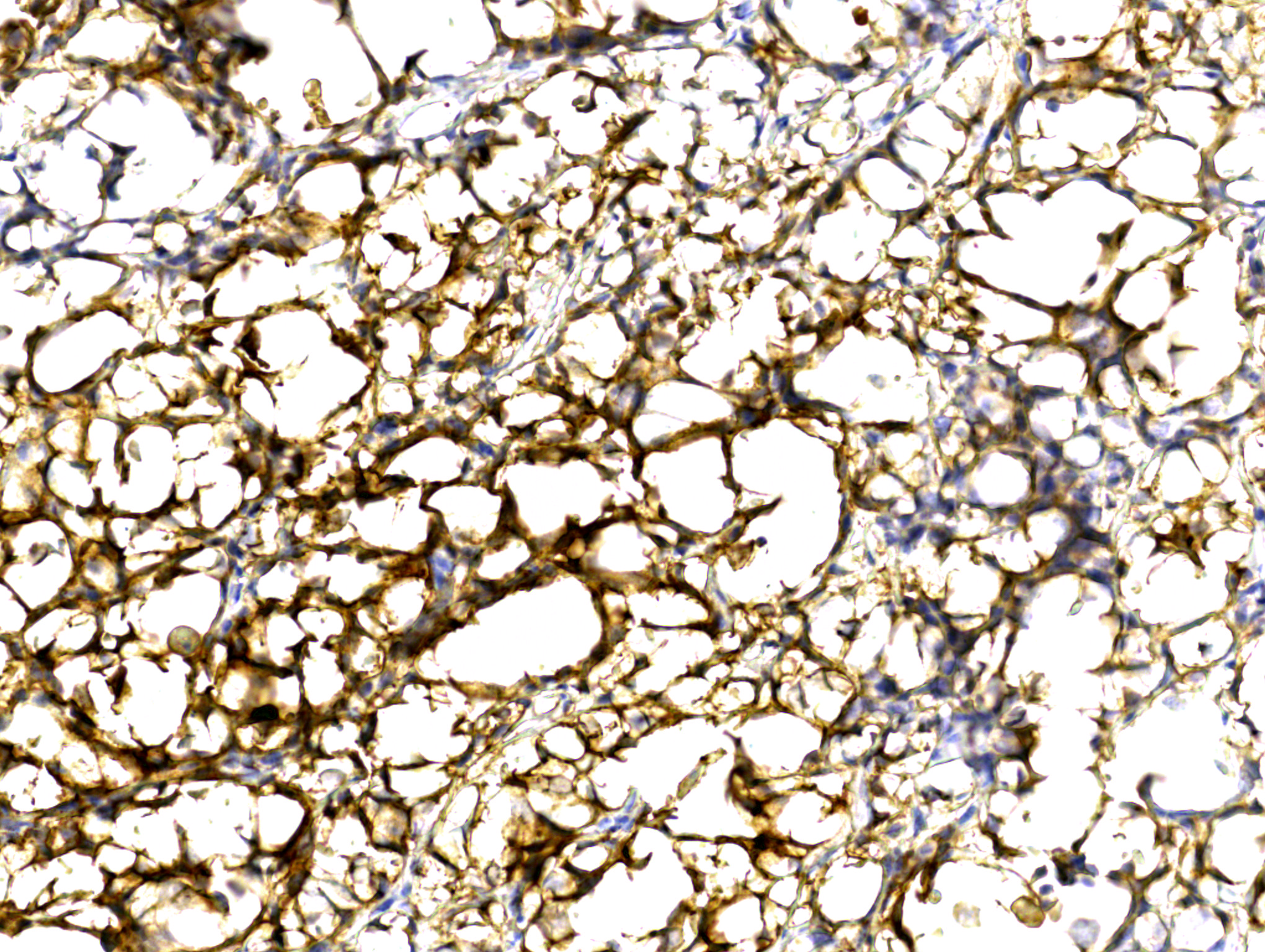

Supplement: Supplementary file 11 — Source data Fig. 6 [file 44319_2024_180_MOESM11_ESM.zip › Figure 6/6B/scr CAF.tif]

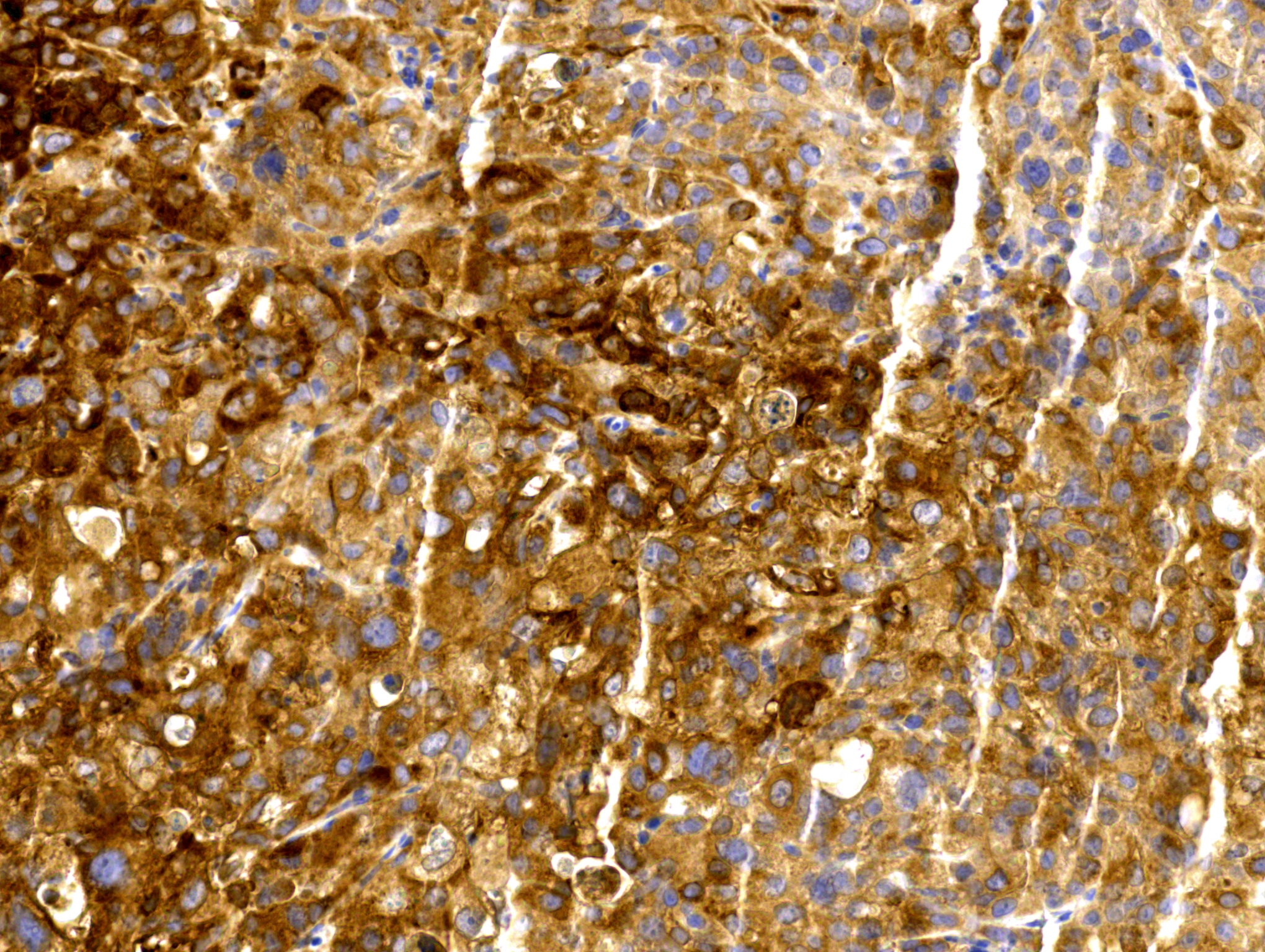

Supplement: Supplementary file 11 — Source data Fig. 6 [file 44319_2024_180_MOESM11_ESM.zip › Figure 6/6B/scr Lactate.tif]

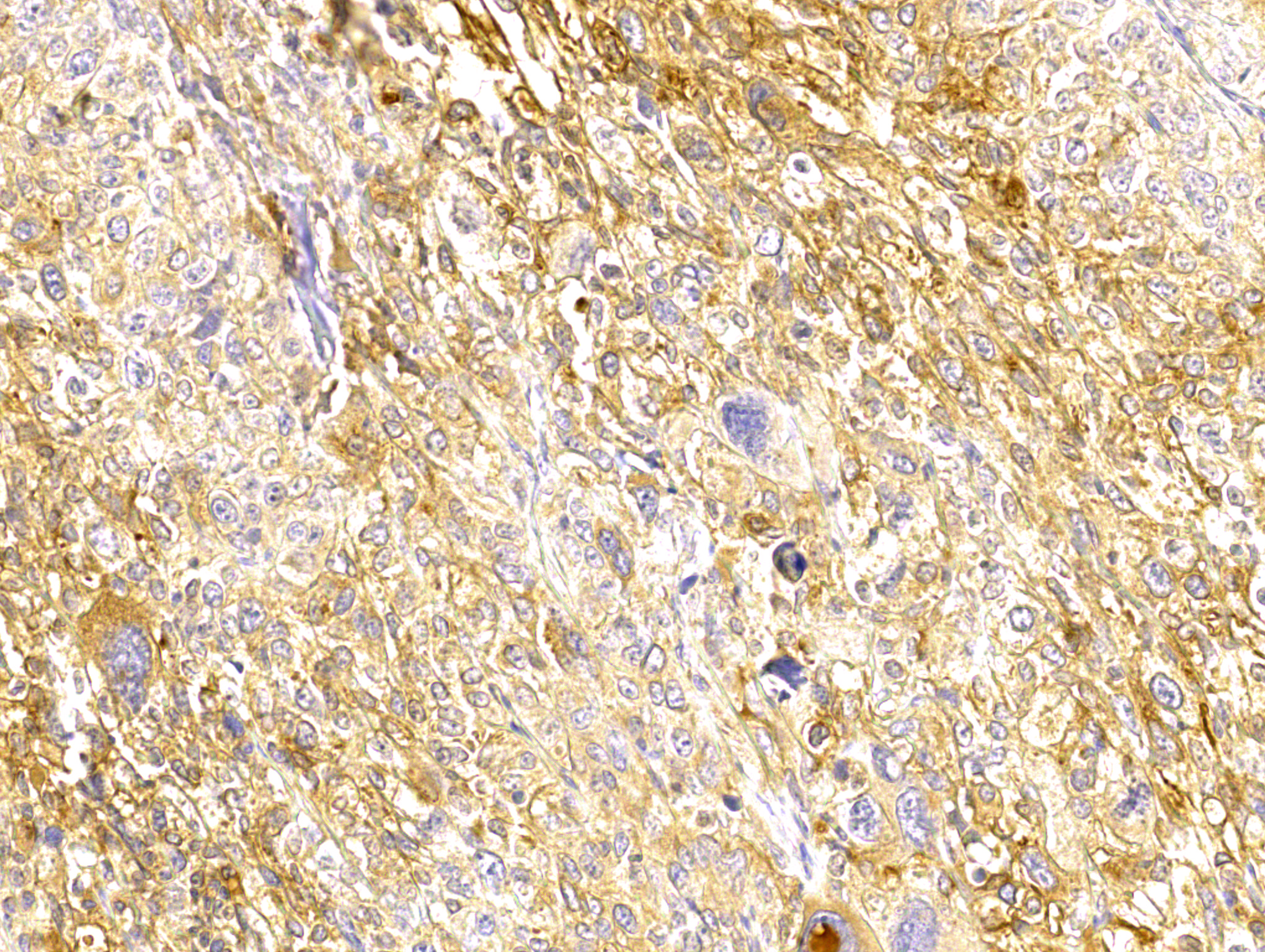

Supplement: Supplementary file 11 — Source data Fig. 6 [file 44319_2024_180_MOESM11_ESM.zip › Figure 6/6B/shP4HA1 Lactate.tif]

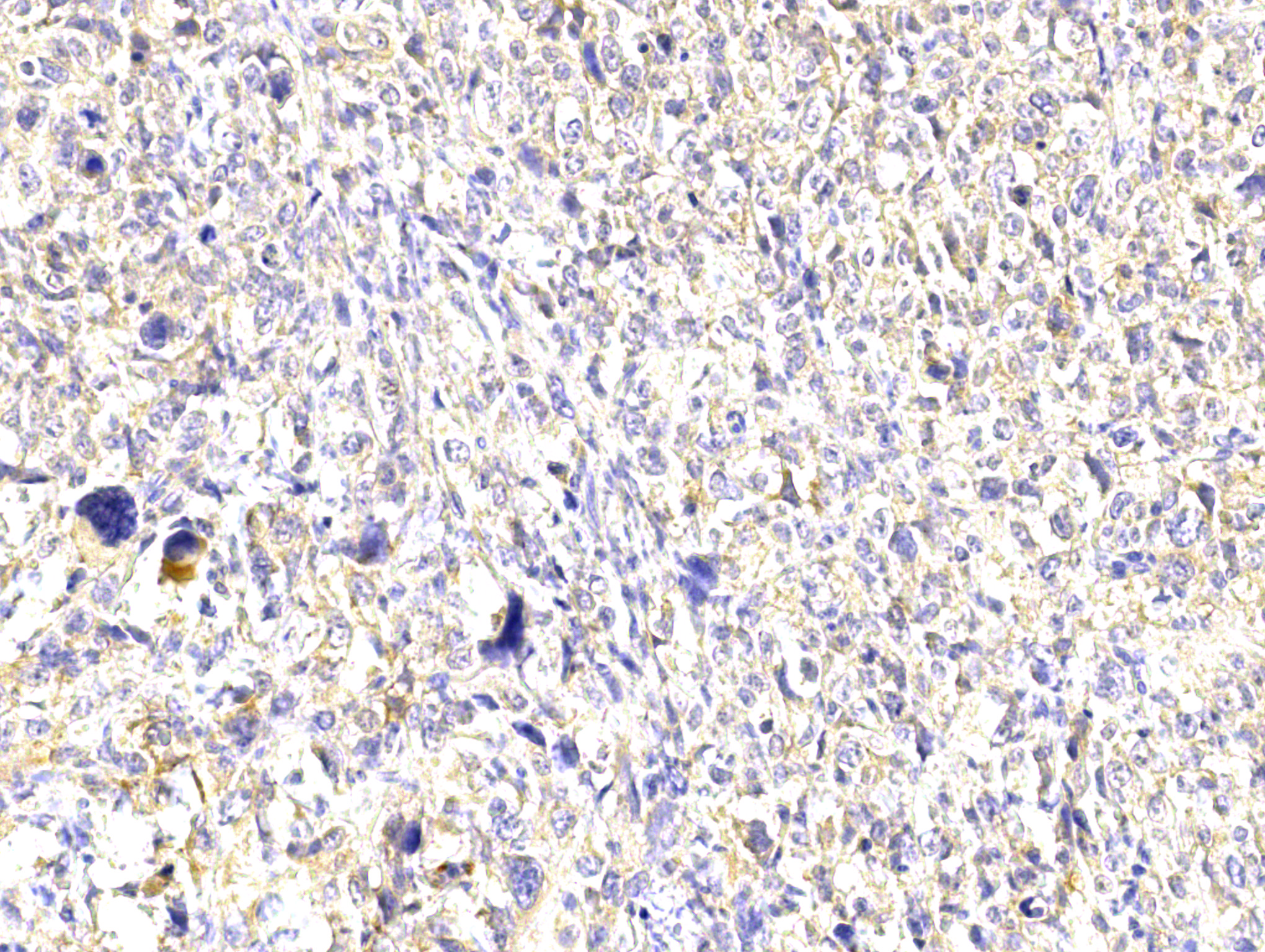

Supplement: Supplementary file 11 — Source data Fig. 6 [file 44319_2024_180_MOESM11_ESM.zip › Figure 6/6B/shP4HA1 HPF.tif]

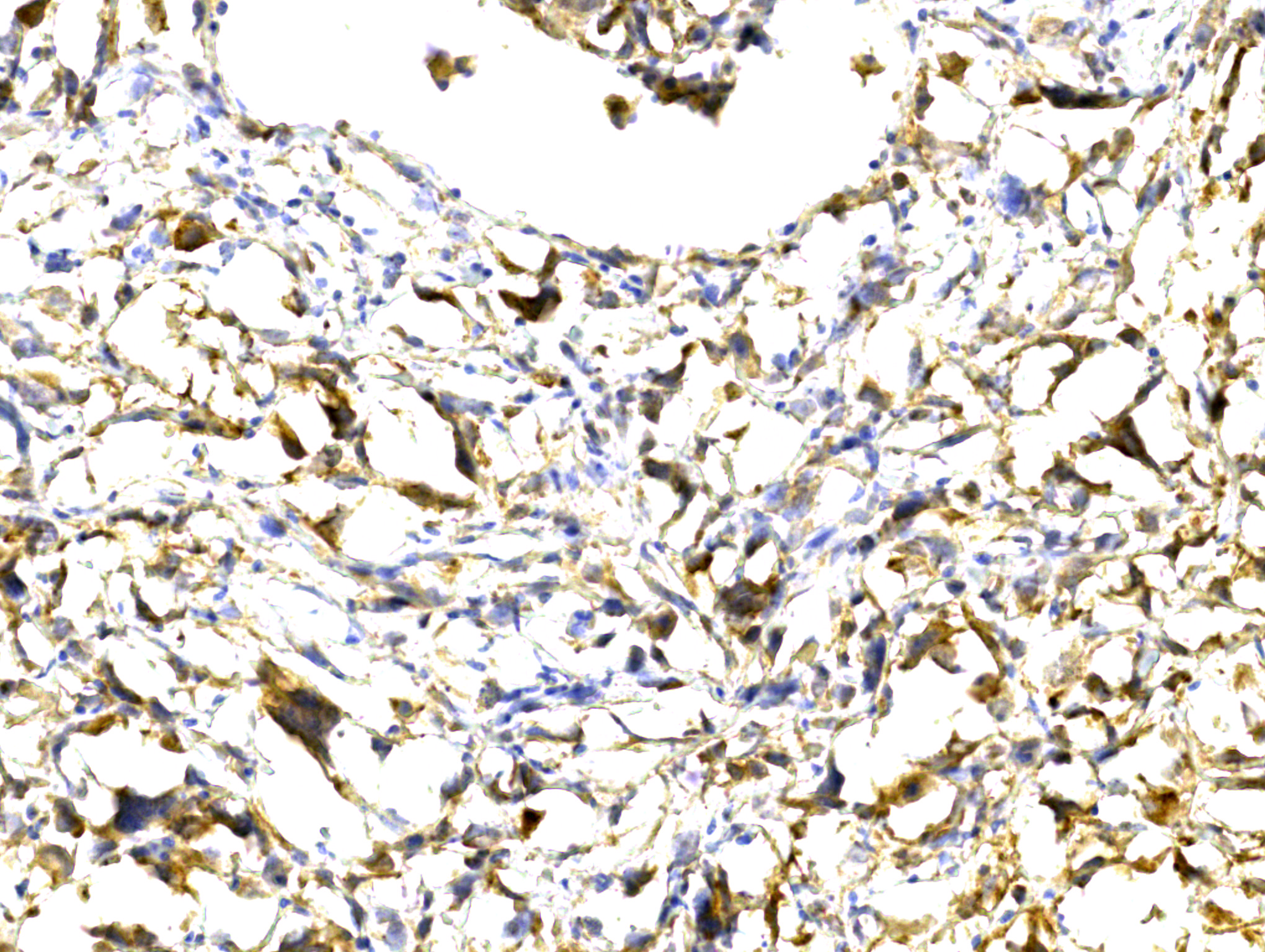

Supplement: Supplementary file 11 — Source data Fig. 6 [file 44319_2024_180_MOESM11_ESM.zip › Figure 6/6B/shP4HA1 CAF.tif]

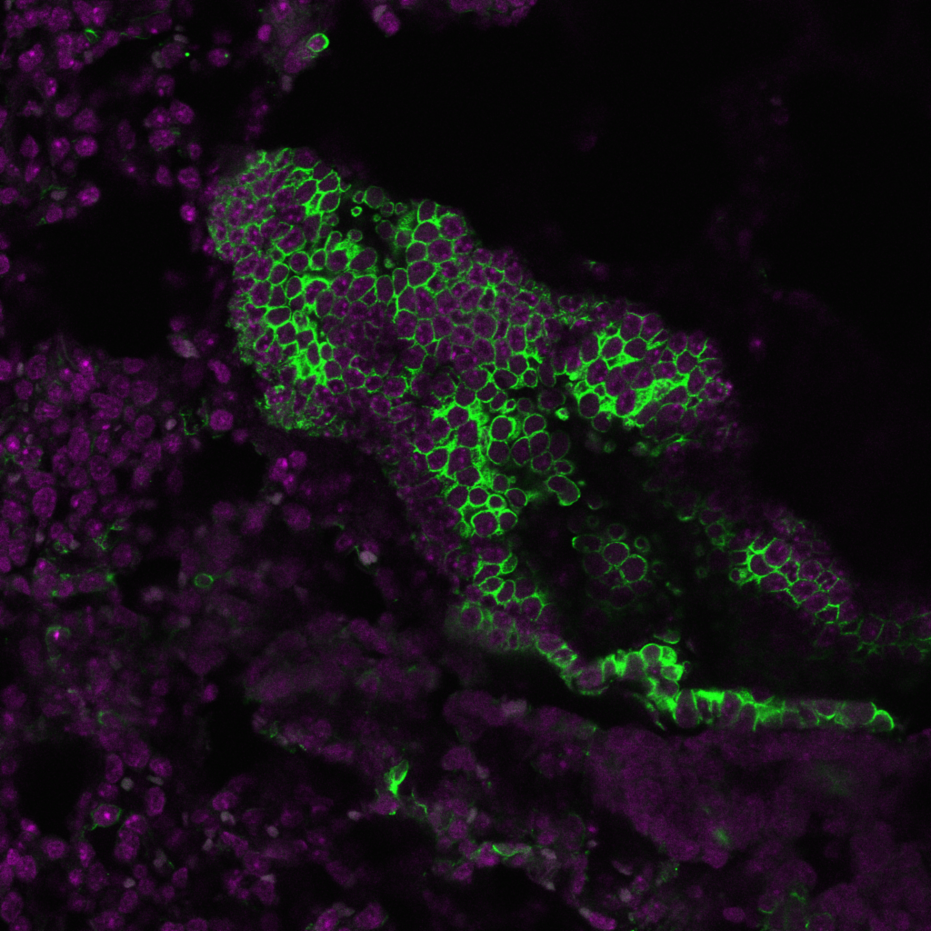

Supplement: Supplementary file 11 — Source data Fig. 6 [file 44319_2024_180_MOESM11_ESM.zip › Figure 6/6D/PanCK Scr_Lactate.tif]

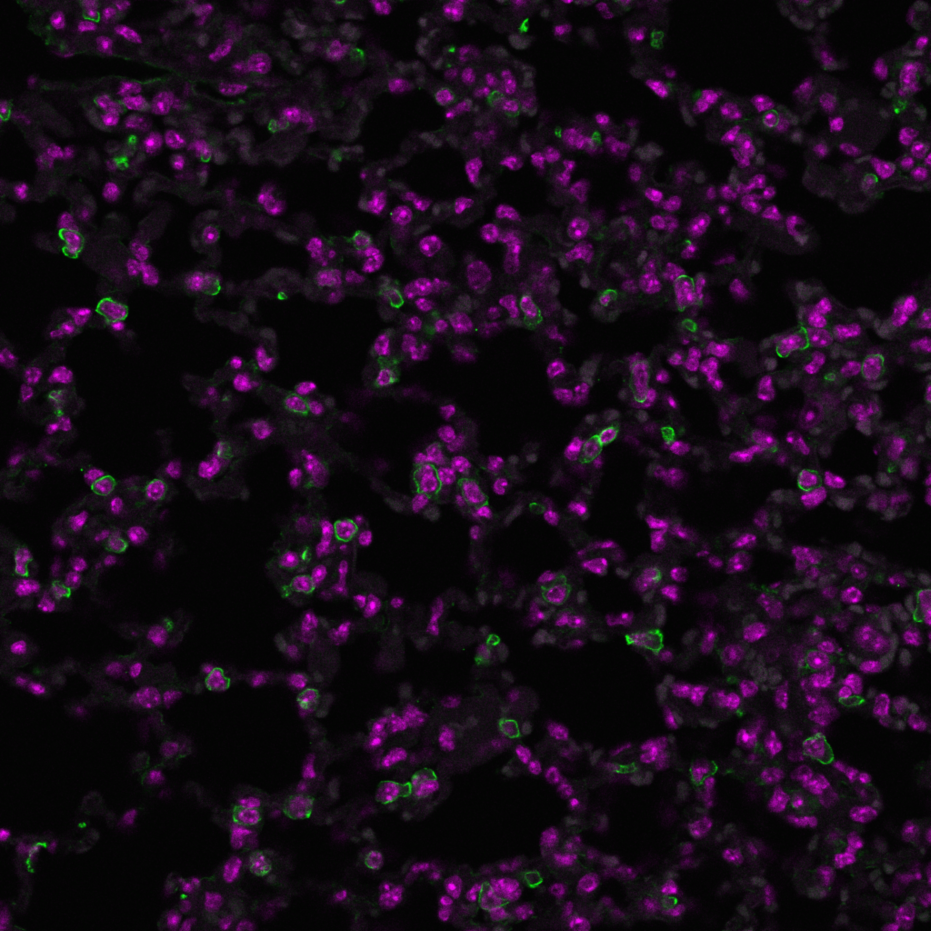

Supplement: Supplementary file 11 — Source data Fig. 6 [file 44319_2024_180_MOESM11_ESM.zip › Figure 6/6D/PanCK Scr_HPF.tif]

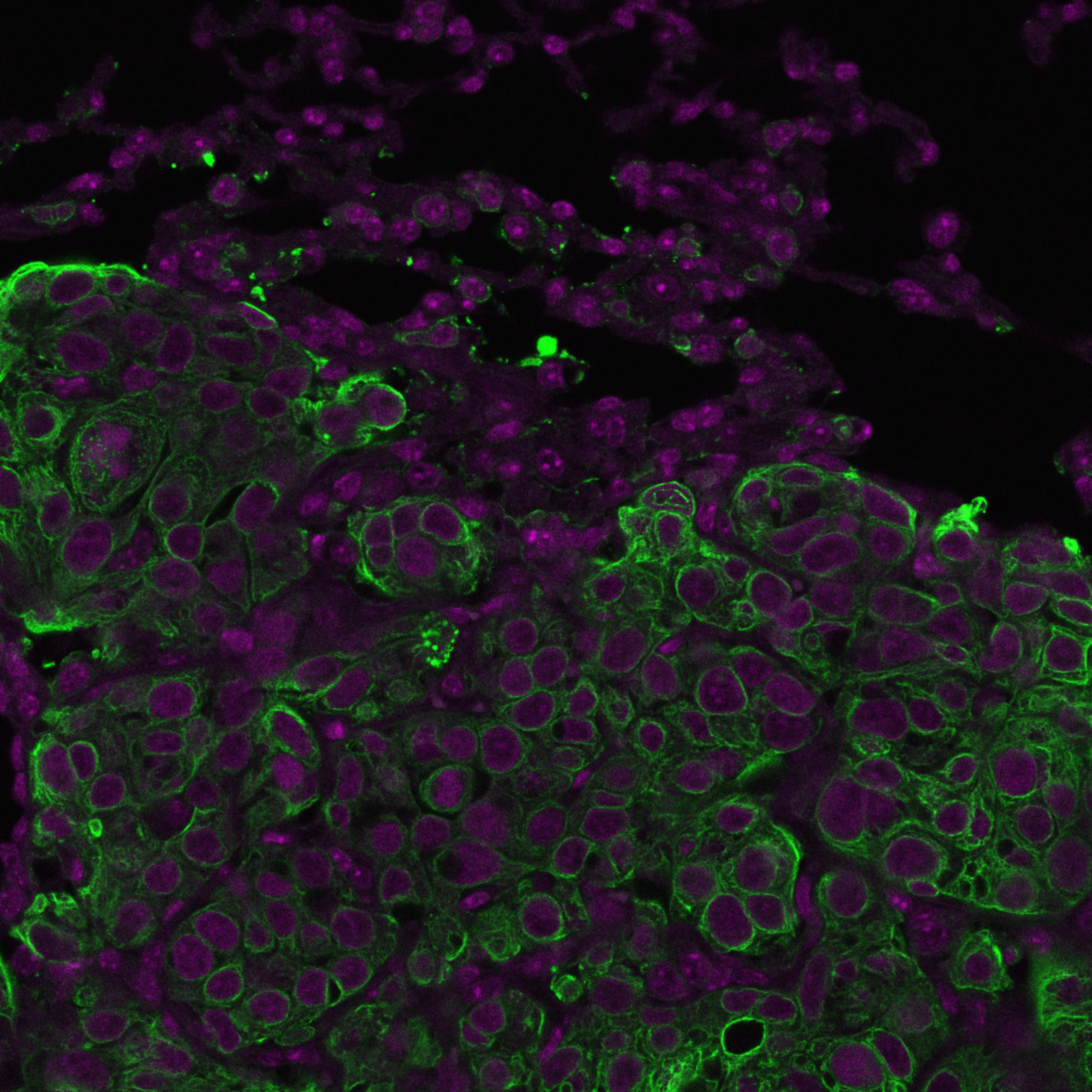

Supplement: Supplementary file 11 — Source data Fig. 6 [file 44319_2024_180_MOESM11_ESM.zip › Figure 6/6D/PanCK Scr_CAF.tif]

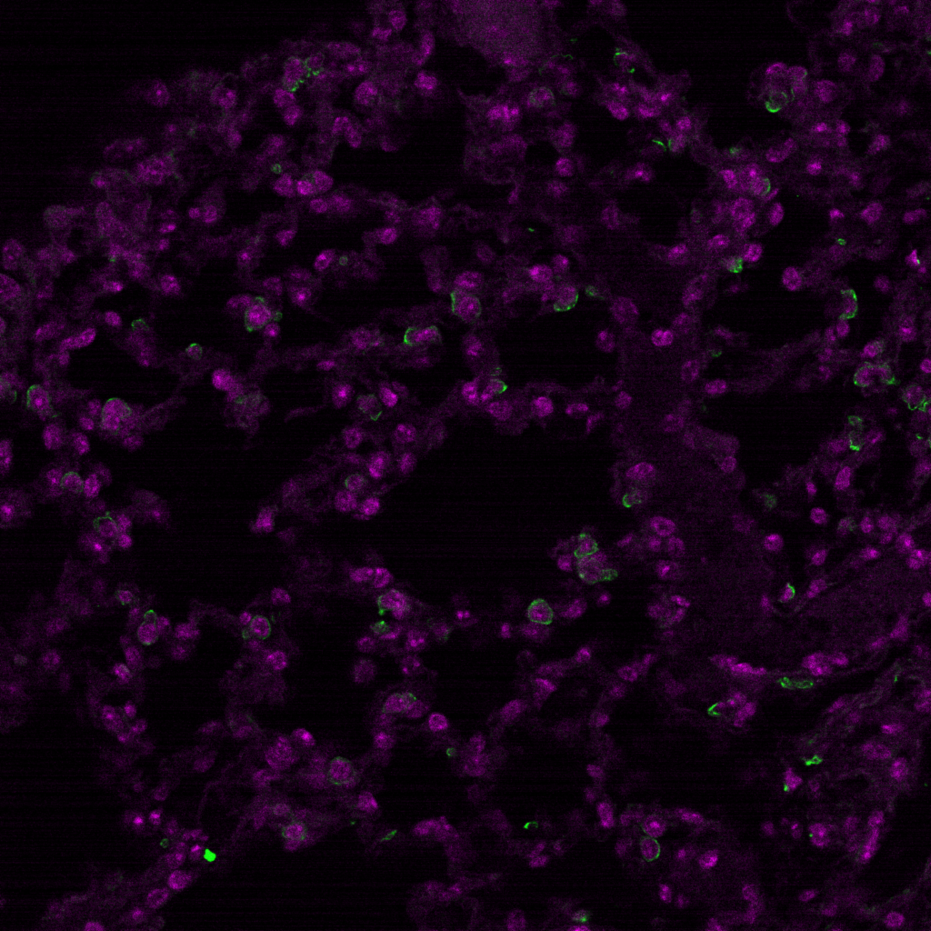

Supplement: Supplementary file 11 — Source data Fig. 6 [file 44319_2024_180_MOESM11_ESM.zip › Figure 6/6D/PanCK shP4HA1_HPF.tif]

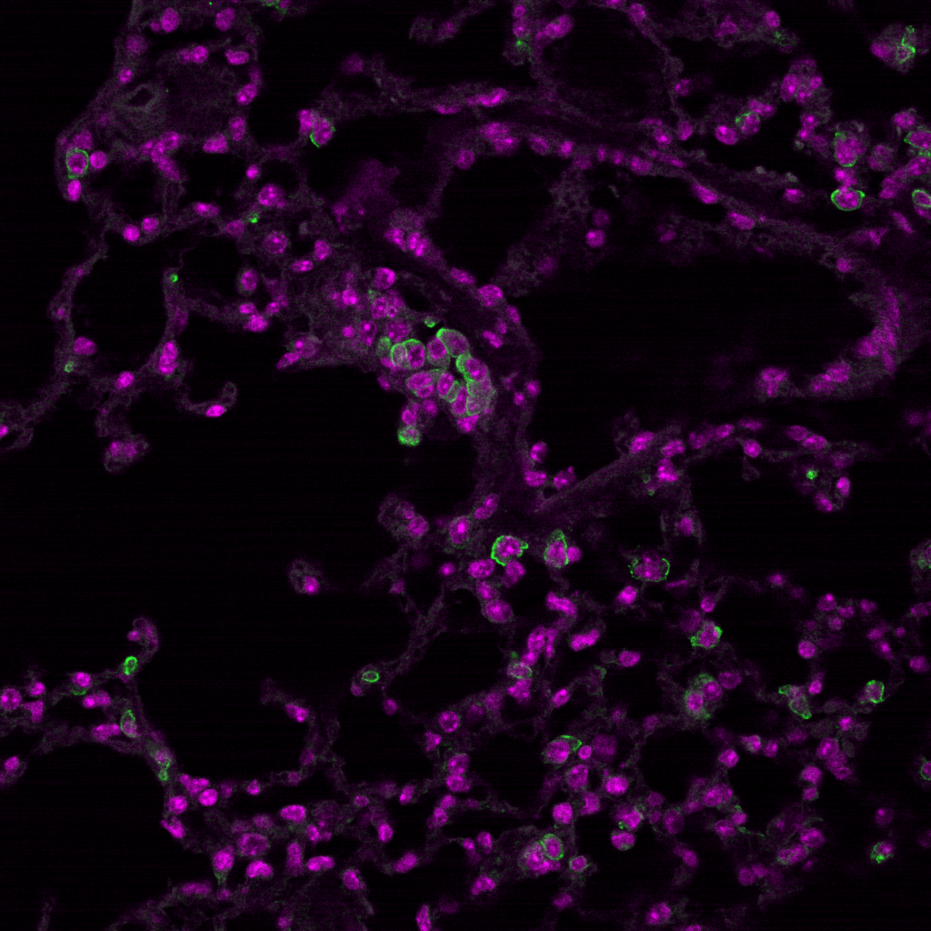

Supplement: Supplementary file 11 — Source data Fig. 6 [file 44319_2024_180_MOESM11_ESM.zip › Figure 6/6D/PanCK shP4HA1_Lactate.tif]

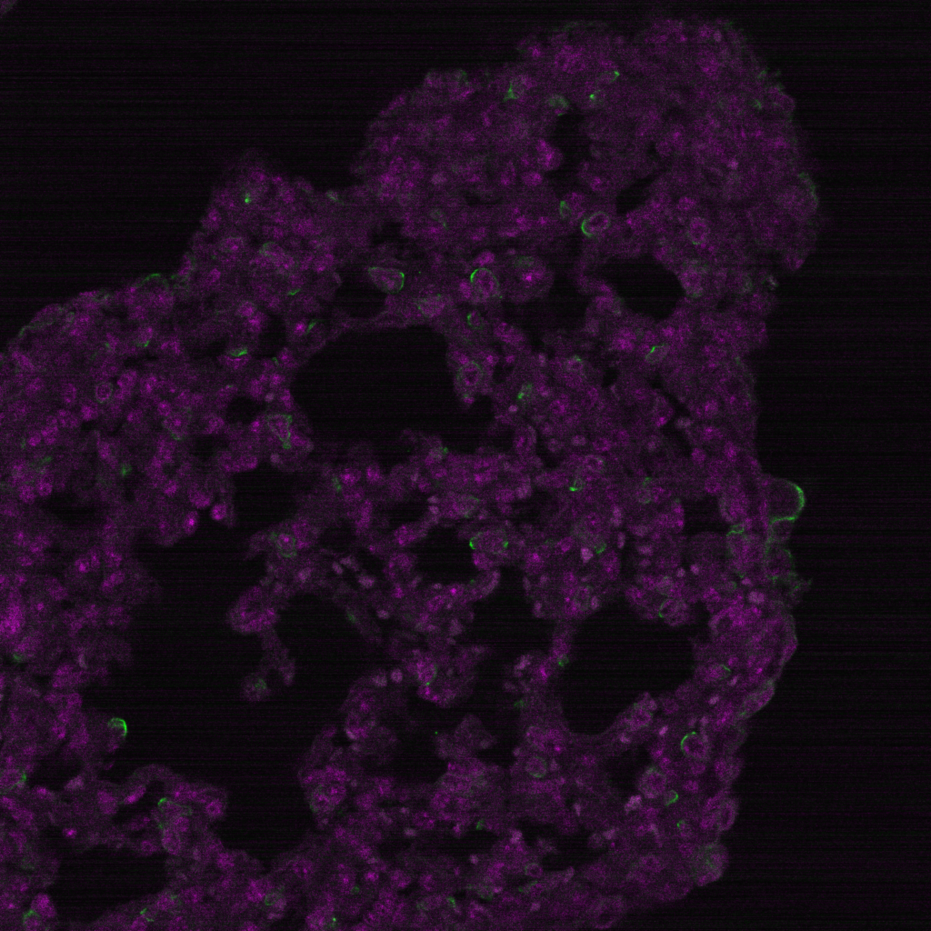

Supplement: Supplementary file 11 — Source data Fig. 6 [file 44319_2024_180_MOESM11_ESM.zip › Figure 6/6D/PanCK shP4HA1_CAF.tif]

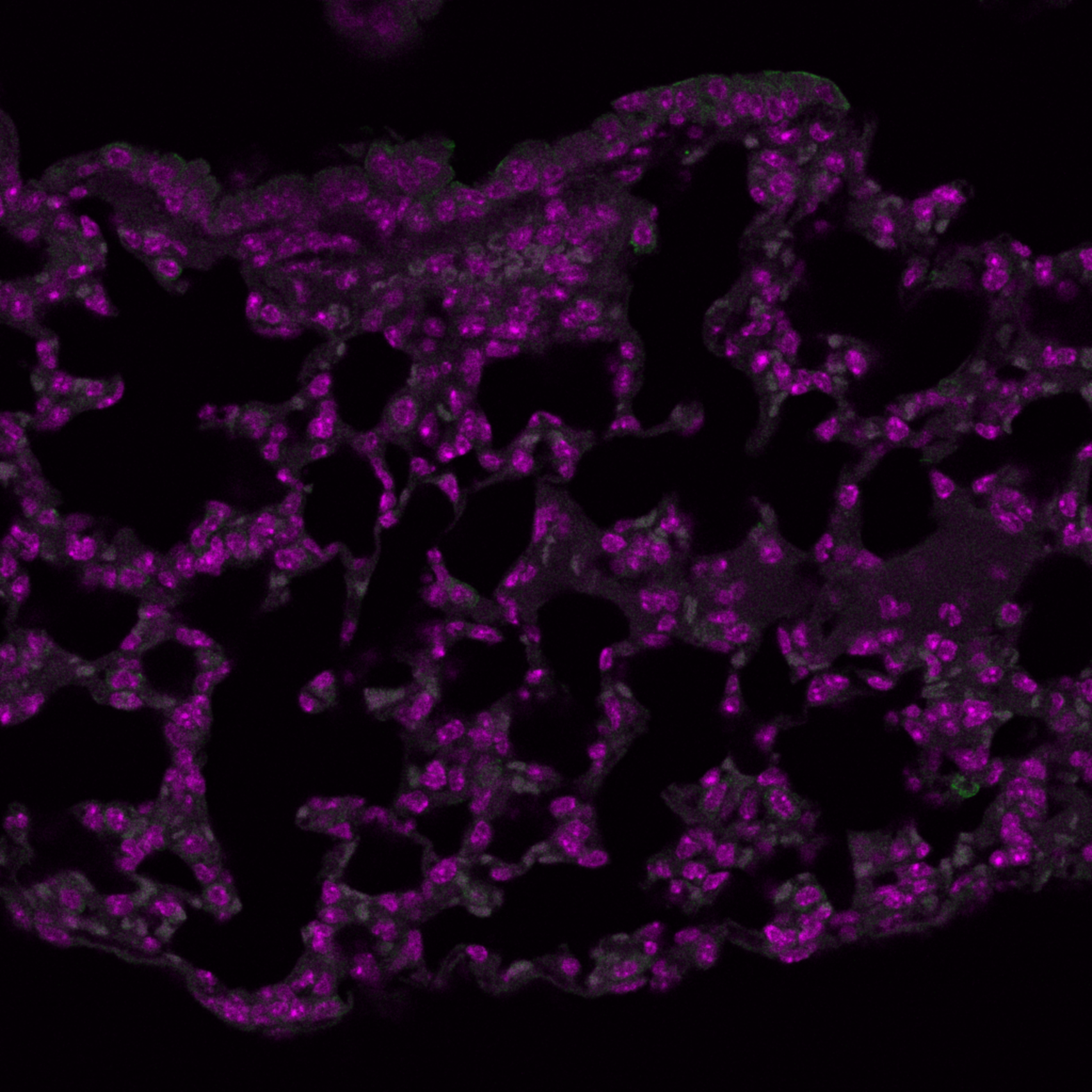

Supplement: Supplementary file 11 — Source data Fig. 6 [file 44319_2024_180_MOESM11_ESM.zip › Figure 6/6D/PanCK shDDR1_Lactate.tif]

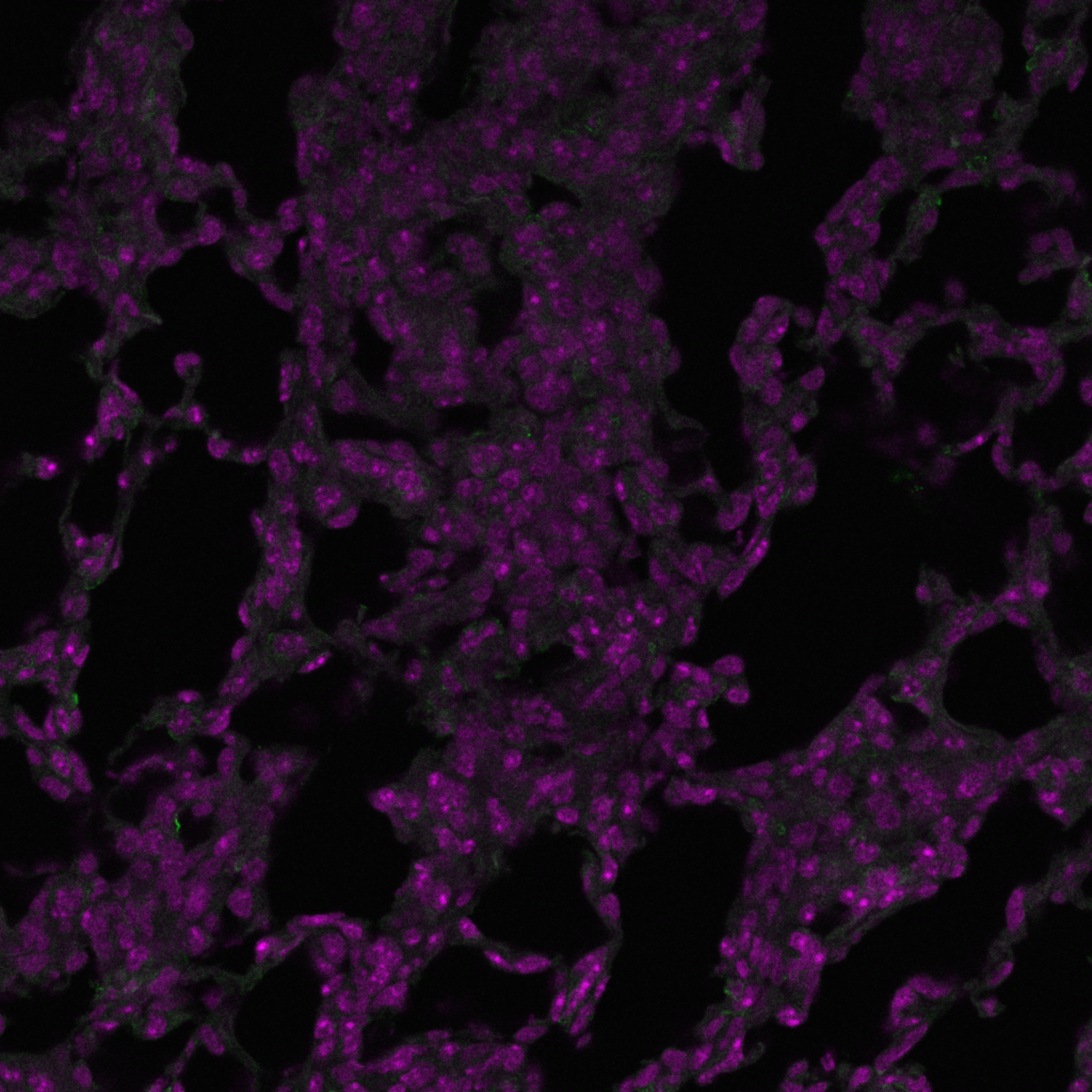

Supplement: Supplementary file 11 — Source data Fig. 6 [file 44319_2024_180_MOESM11_ESM.zip › Figure 6/6D/PanCK shDDR1_CAF.tif]

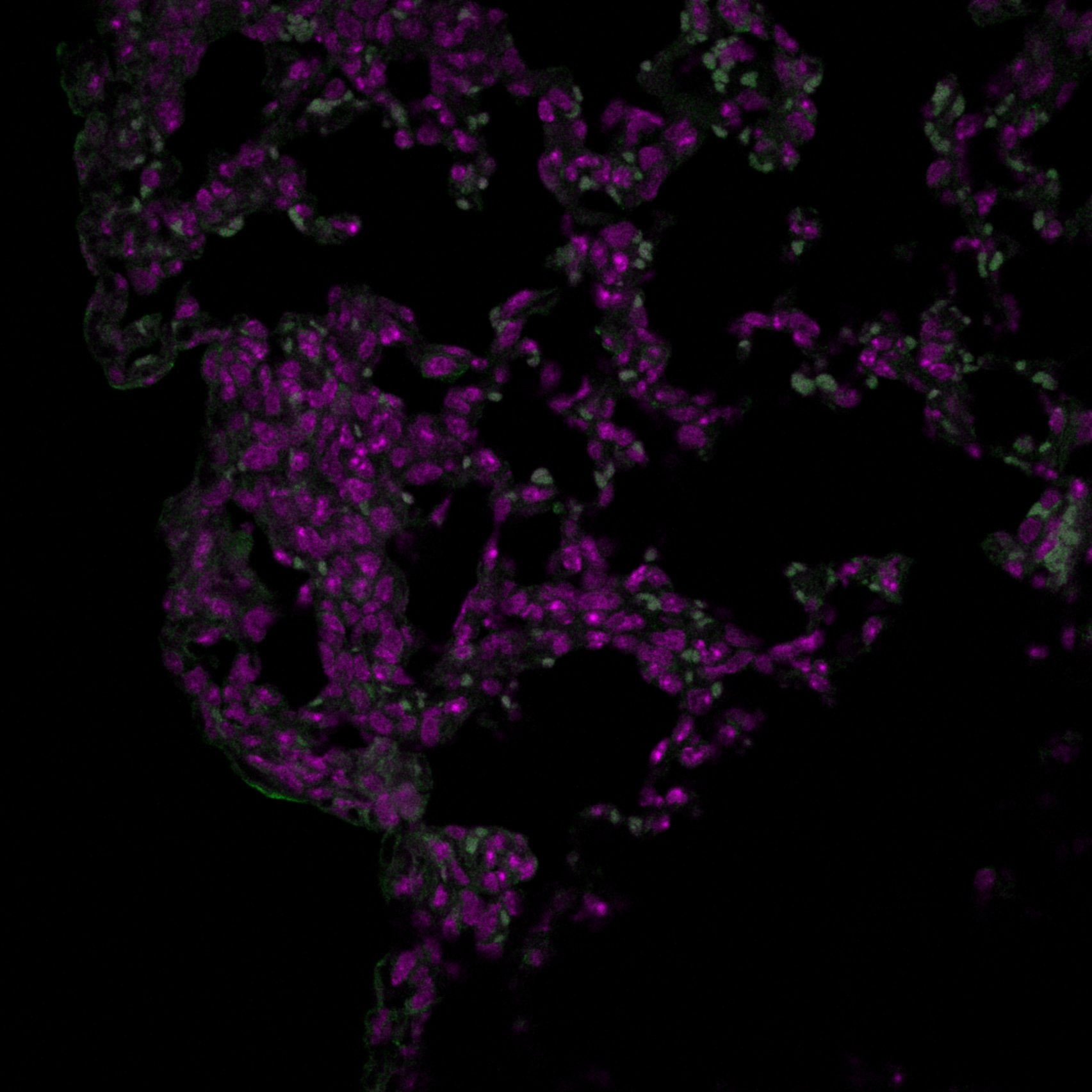

Supplement: Supplementary file 11 — Source data Fig. 6 [file 44319_2024_180_MOESM11_ESM.zip › Figure 6/6D/PanCK shDDR1_HPF.tif]
