# Supplementary material for: Lactate supports cell-autonomous ECM production to sustain metastatic behavior in prostate cancer
Source: EMBO Rep. 2024 Jun 21;25(8):19. doi: 10.1038/s44319-024-00180-z (PMC11315984; doi:10.1038/s44319-024-00180-z)
Supplement: Supplementary file 12 — Expanded View Figures [file 44319_2024_180_MOESM12_ESM.pdf]

Expanded View Figures

Figure EV1. Collagen signature is enhanced in CAF-derived LA-treated PCa cells.

(**A**) Dotplot showing Reactome-enrichment of genes associated to the cell ECM pathway in LA-treated DU145 cells. Circle size is proportional to the enrichment score. Red dots highlight the significantly enriched pathways. (**B**) qRT-PCR analysis for COL1A1 mRNA level in DU145 and PC3 cells treated with HPF-CM, CAF-CM or LA. (**C**) Immunofluorescence analysis of extracellular COL1A1 on decellularized matrix derived from DU145 treated as indicated ± MCT1i; scale bar: 10 µm. Quantification of fluorescence signal was reported. (**D**) Immunofluorescence analysis of intracellular COL1A1 on DU145 cells, silenced for MCT1 (siMCT1) or a non-targeting control (siCTR). Nuclei (blue) were stained with DAPI. Scale bar: 10 µm. Box plot showing the quantification of fluorescence signal per cell: centerlines show the medians; box limits indicate the 25th and 75th percentiles; and whiskers extend to the minimum and maximum. $n = 9$-27 cells from three biological replicates. (**E**) Enrichment plots of the Hallmark Hypoxia showing a positive association between this MSigDb datasets and the LA-exposed DU145 gene expression profile. NES, normalized enrichment score. (**F**) Representative western blot analysis of P4HA1 in 22Rv1 cells, exposed to LA and to a serum-free medium as control (not treated, NT). GAPDH was used as loading control. Representative western blot analysis of P4HA1 in PC3 cells treated as indicated ± MCT1i. Beta-actin was used as loading control. (**G**) Representative western blot analysis of P4HA1 and MCT1 in siCTR and siMCT1-DU145 cells. Beta-actin was used as loading control. Data information: bar graphs in (**B**–**D**) represent means ± SEM of $n = 3$ biological replicates. Significance was determined using one-way ANOVA, followed by Tukey's multiple comparisons test (*$P < 0.05$; **$P < 0.01$; ***$P < 0.001$; ****$P < 0.0001$). Source data are available online for this figure.

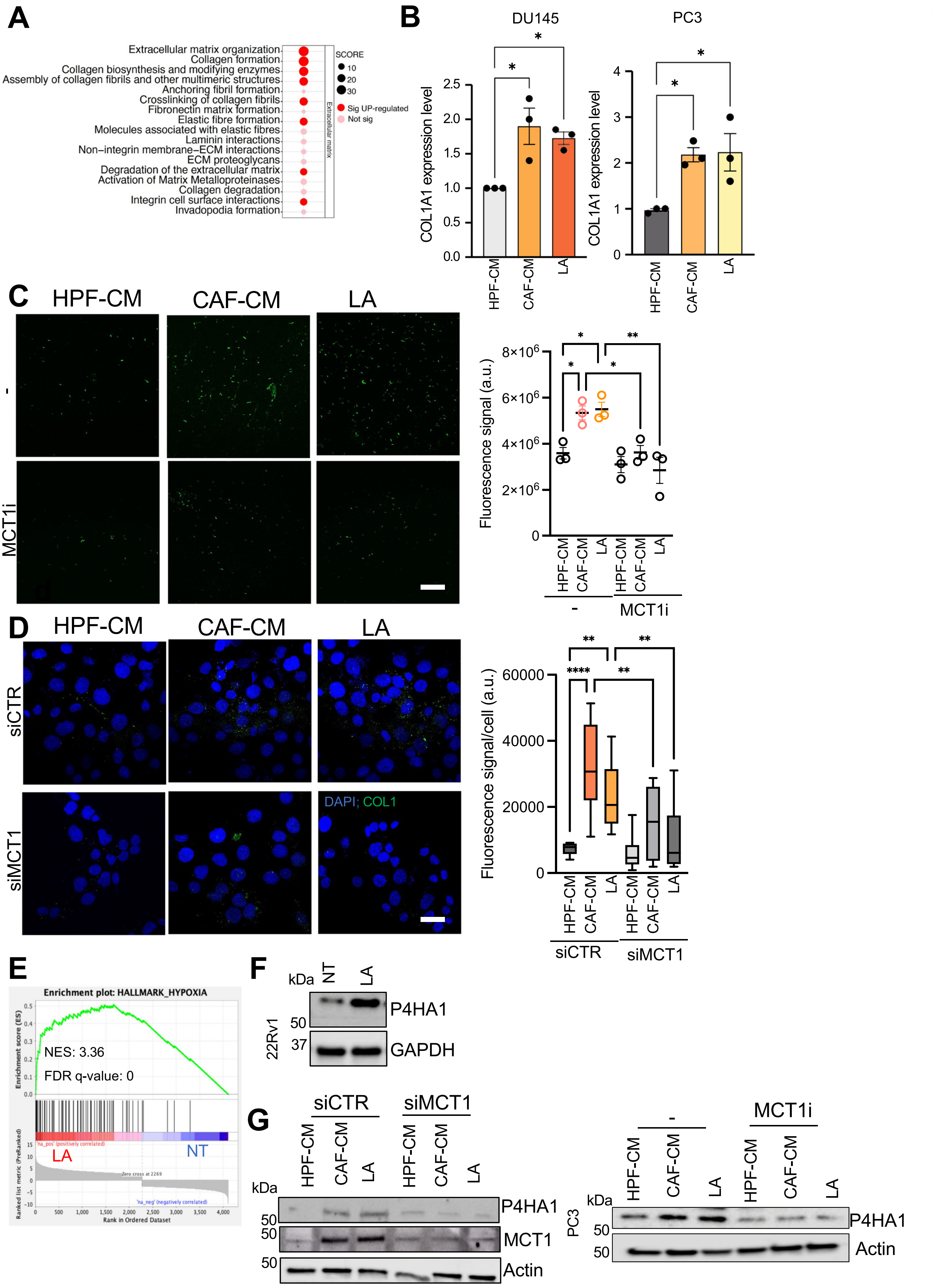
A
Extracellular matrix organization
Collagen formation
Collagen biosynthesis and modifying enzymes
Assembly of collagen fibrils and other multimeric structures
Anchoring fibril formation
Crosslinking of collagen fibrils
Fibronectin matrix formation
Elastic fibre formation
Molecules associated with elastic fibres
Laminin interactions
Non−integrin membrane−ECM interactions
ECM proteoglycans
Degradation of the extracellular matrix
Activation of Matrix Metalloproteinases
Collagen degradation
Integrin cell surface interactions
Invadopodia formation
Extracellular matrix
SCORE
10
20
30
Sig UP-regulated
Not sig
B
DU145
PC3
COL1A1 expression level
HPF-CM
CAF-CM
LA
C
HPF-CM
CAF-CM
LA
-
MCT1i
Fluorescence signal (a.u.)
MCT1i
D
siCTR
siMCT1
DAPI; COL1
Fluorescence signal/cell (a.u.)
E
Enrichment plot: HALLMARK_HYPOXIA
Enrichment score (ES)
NES: 3.36
FDR q-value: 0
LA
NT
Ranked list metric (PreRanked)
Rank in Ordered Dataset
F
kDa
NT
P4HA1
GAPDH
22Rv1
G
siCTR
siMCT1
MCT1
Actin
PC3

© The Author(s)

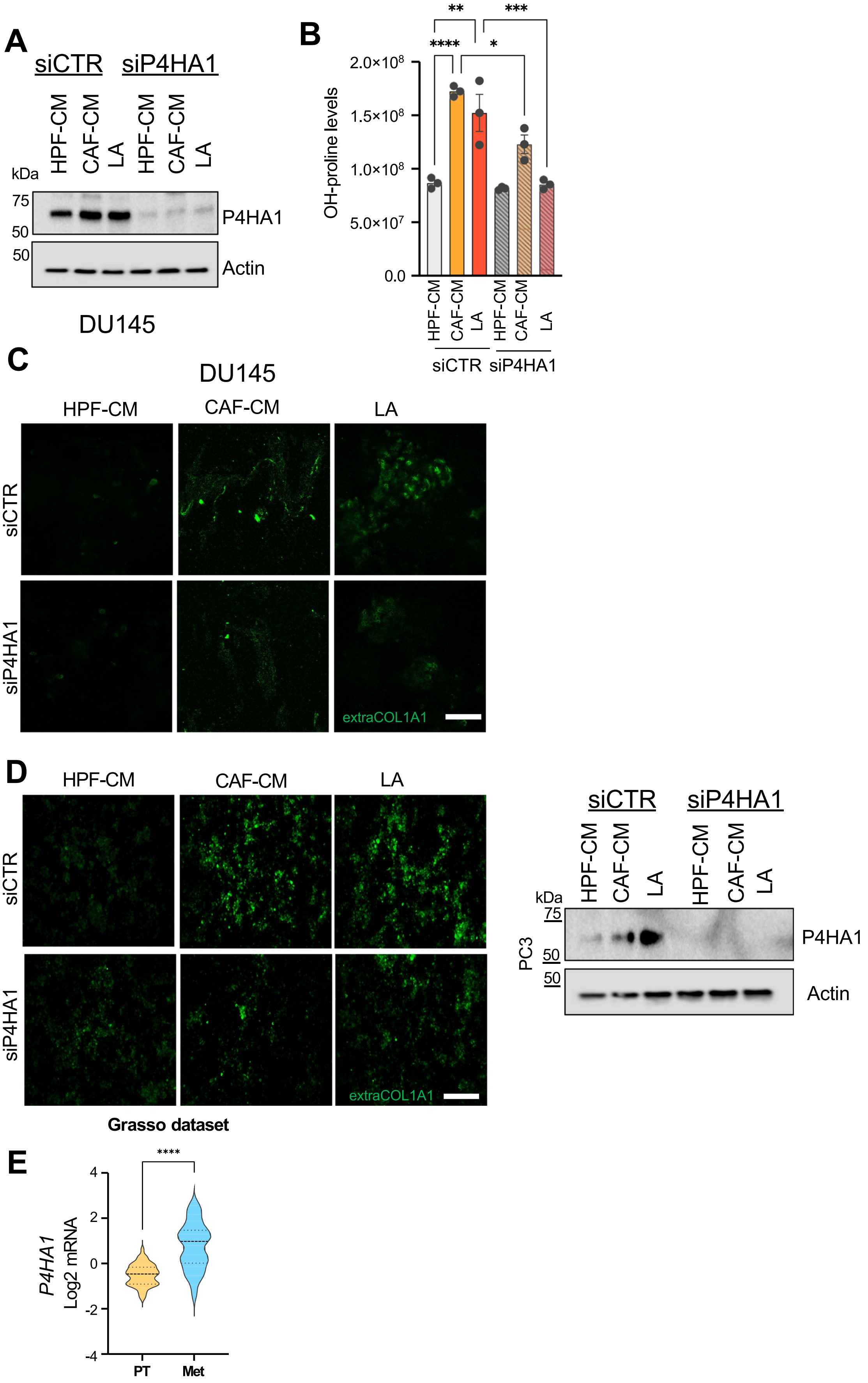
A
siCTR
siP4HA1
HPF-CM
CAF-CM
LA
HPF-CM
CAF-CM
LA
kDa
75
50
50
P4HA1
Actin
DU145
B
OH-proline levels
2.0×10^8
1.5×10^8
1.0×10^8
5.0×10^7
0.0
**
***
****
*
HPF-CM
CAF-CM
LA
HPF-CM
CAF-CM
LA
siCTR
siP4HA1
C
DU145
HPF-CM
CAF-CM
LA
siCTR
siP4HA1
extraCOL1A1
D
HPF-CM
CAF-CM
LA
siCTR
siP4HA1
extraCOL1A1
siCTR
siP4HA1
HPF-CM
CAF-CM
LA
HPF-CM
CAF-CM
LA
kDa
75
50
50
PC3
P4HA1
Actin
Grasso dataset
E
****
P4HA1
Log2 mRNA
4
2
0
-2
-4
PT
Met

Figure EV2. P4HA1 sustains the LA-induced collagen deposition in PCa cells.

(**A**) Representative western blot analysis of P4HA1 in DU145 silenced for P4HA1, treated as indicated. Beta-actin was used as loading control. (**B**) GC-MS analysis of hydroxyproline levels in siCTR and siP4HA1-DU145 cells, treated as indicated. (**C**, **D**) Immunofluorescence analysis of extracellular COL1A1 on decellularized matrix derived from DU145 (**C**) and PC3 (**D**) cells, treated as indicated and silenced for P4HA1; scale bar: 10 μm. (**E**) Violin plots representing P4HA1 mRNA expression in primary (PT) and metastatic (Met) tumor specimens of Grasso dataset ($n = 88$). Data information: bar graphs in (**B**, **E**) represent means ± SEM; (**B**) $n = 3$ biological replicates. Significance was determined using one-way ANOVA, followed by Tukey's multiple comparisons test (**B**) (*P < 0.05; **P < 0.01; ***P < 0.001; ****P < 0.0001), or unpaired two-tailed t test (**E**) (****P < 0.0001). Source data are available online for this figure.

 © The Author(s)

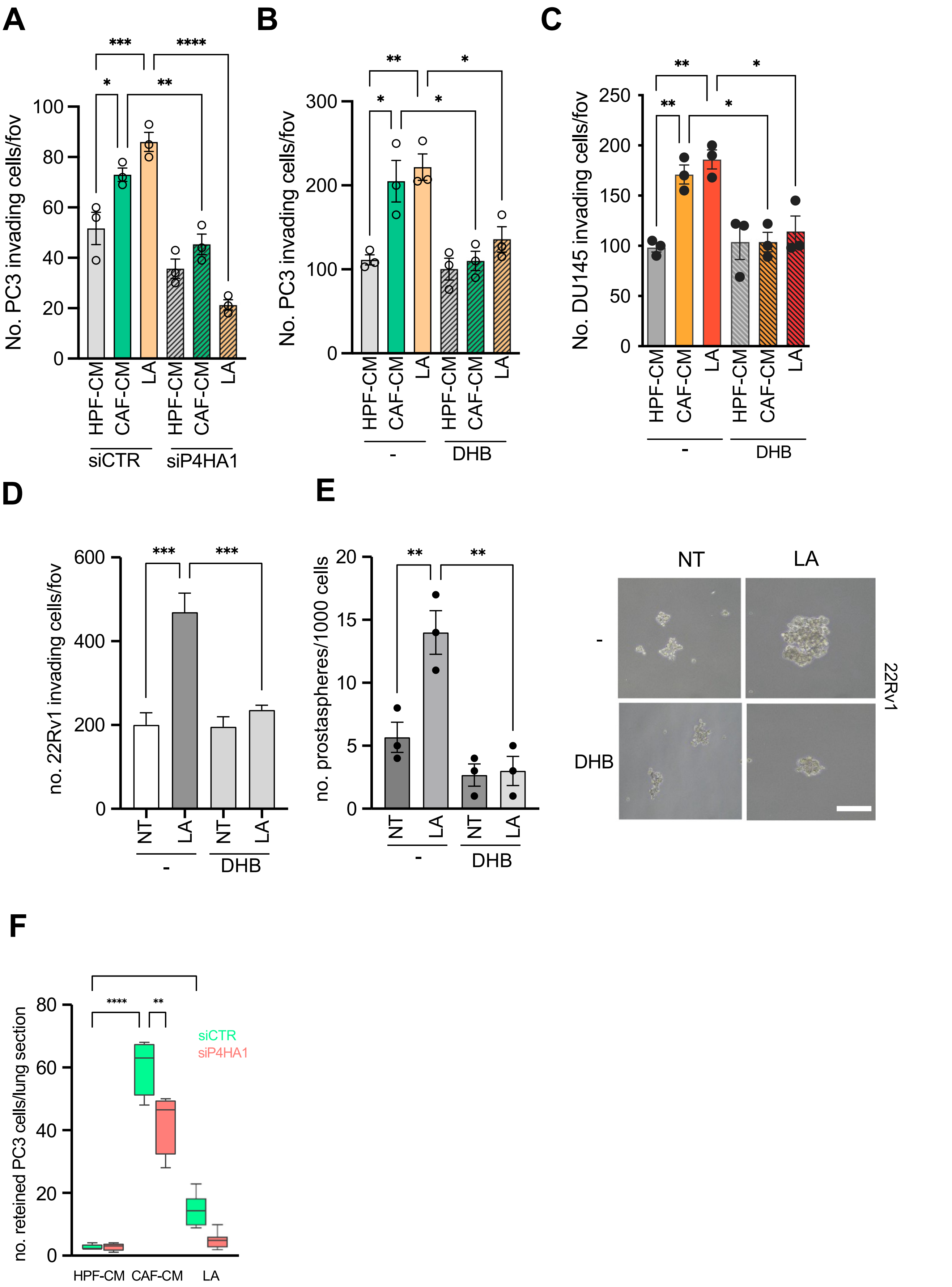
A
***
****
*
**
No. PC3 invading cells/fov
100
80
60
40
20
0
HPF-CM
CAF-CM
LA
HPF-CM
CAF-CM
LA
siCTR
siP4HA1
B
**
*
*
*
No. PC3 invading cells/fov
300
200
100
0
HPF-CM
CAF-CM
LA
HPF-CM
CAF-CM
LA
-
DHB
C
**
*
**
*
No. DU145 invading cells/fov
250
200
150
100
50
0
HPF-CM
CAF-CM
LA
HPF-CM
CAF-CM
LA
-
DHB
D
***
***
no. 22Rv1 invading cells/fov
600
400
200
0
NT
LA
NT
LA
-
DHB
E
**
**
no. prostaspheres/1000 cells
20
15
10
5
0
NT
LA
NT
LA
-
DHB
NT
LA
-
DHB
22Rv1
F
****
**
no. reteined PC3 cells/lung section
80
60
40
20
0
siCTR
siP4HA1
HPF-CM
CAF-CM
LA

Figure EV3. Targeting LA-induced P4HA1 impairs PCa cell invasive and colonizing abilities.

(**A**) Invasion assay performed on PC3 cells treated as indicated, silenced for P4HA1. (**B**) Invasion assay performed on PC3 cells treated as indicated, ± DHB (20 μM). (**C**) Invasion assay performed on DU145 cells treated as indicated, ± DHB (20 μM). (**D**) Invasion assay performed on 22Rv1 cells treated as indicated, ± DHB (20 μM). (**E**) Prostaspheres formation assay (representative pictures at day 10 are shown, scale bar: 100 μm) for 22Rv1 cells treated as indicated, ± DHB. (**F**) Lung retention assay performed on PC3 cells, treated as indicated, then labeled with CellTracker dyes (green for siCTR and red for siP4HA1) before being 1:1 injected into the tail vein of SCID mice. Quantification of green and red fluorescent cells per lung section field of view was reported as box plot: centerlines show the medians; box limits indicate the 25th and 75th percentiles; and whiskers extend to the minimum and maximum; $n = 4$–6 sections. Data information: bar graphs in (**A–F**) represent means ± SEM, (**A–E**) $n = 3$ biological replicates, (**F**) $n = 4$ mice/group. Significance was determined using one-way ANOVA, followed by Tukey's multiple comparisons test (*$P < 0.05$; **$P < 0.01$; ***$P < 0.001$; ****$P < 0.0001$). Source data are available online for this figure.

 © The Author(s)

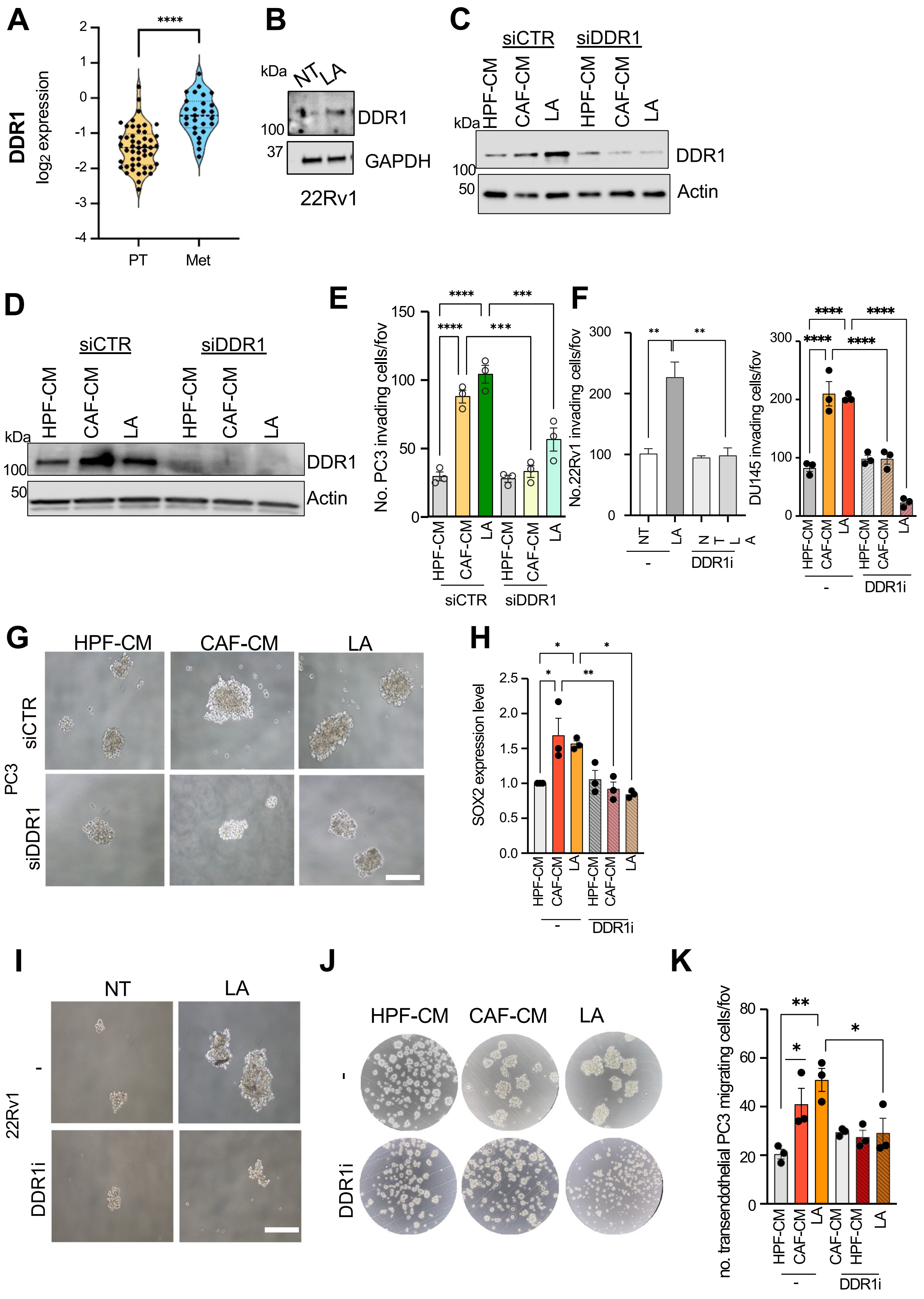
A
DDR1
log2 expression
****
PT
Met
B
kDa
NT
LA
100
37
DDR1
GAPDH
22Rv1
C
siCTR
siDDR1
HPF-CM
CAF-CM
LA
HPF-CM
CAF-CM
LA
kDa
DDR1
Actin
100
50
D
siCTR
siDDR1
HPF-CM
CAF-CM
LA
HPF-CM
CAF-CM
LA
kDa
100
50
DDR1
Actin
E
No. PC3 invading cells/fov
siCTR
siDDR1
F
No.22Rv1 invading cells/fov
NT
LA
NT
LA
DDR1i
DU145 invading cells/fov
HPF-CM
CAF-CM
LA
HPF-CM
CAF-CM
LA
DDR1i
G
HPF-CM
CAF-CM
LA
siCTR
PC3
siDDR1
H
SOX2 expression level
HPF-CM
CAF-CM
LA
HPF-CM
CAF-CM
LA
DDR1i
I
NT
LA
22Rv1
DDR1i
J
HPF-CM
CAF-CM
LA
DDR1i
K
no. transendothelial PC3 migrating cells/fov
HPF-CM
CAF-CM
LA
CAF-CM
HPF-CM
LA
DDR1i

© The Author(s)

Figure EV4. DDR1 activation is linked to the LA-sustained aggressiveness in PCa cells.

(**A**) Violin plots representing DDR1 mRNA expression in primary (PT) and metastatic (Met) tumor specimens of Grasso dataset ($n = 88$). Each plot means an individual specimen. (**B**) Representative western blot analysis of DDR1 in 22Rv1 cells treated or not (NT) with LA. GAPDH was used as loading control. (**C**, **D**) Representative western blot analysis of DDR1 in siCTR and siDDR1 DU145 and PC3 cells treated as indicated. Beta-actin was used as loading control. (**E**) Invasion assay performed on PC3 cells silenced for DDR1, treated as indicated. (**F**) Invasion assay performed on 22Rv1 and DU145 cells treated as indicated, ± 7rh (DDR1i, 250 and 500 nM, respectively). (**G**) Representative pictures for prostaspheres formation in PC3 cells silenced for DDR1, treated as indicated. Scale bar: 100 µm. (**H**) qRT-PCR analysis for SOX2 mRNA level in DU145 cells treated as indicated, ± DDR1i (500 nM). (**I–L**) Representative pictures for prostaspheres formation in 22Rv1 (**I**) and DU145 cells (**J**), treated as indicated, ± DDR1i. Scale bar: 100 µm. (**K**) Transendothelial migration performed on DU145 cells treated as indicated, ± DDR1i. The number of cells migrating were quantified per field of view (FOV). Data information: bar graphs in (**A**, **E–K**) represent means ± SEM; (**E–K**) $n = 3$ biological replicates. Significance was determined using unpaired two-tailed *t* test (**A**) (****$P < 0.0001$), or one-way ANOVA, followed by Tukey's multiple comparisons test (**E–K**); (*$P < 0.05$; **$P < 0.01$; ***$P < 0.001$; ****$P < 0.0001$). Source data are available online for this figure.

 © The Author(s)

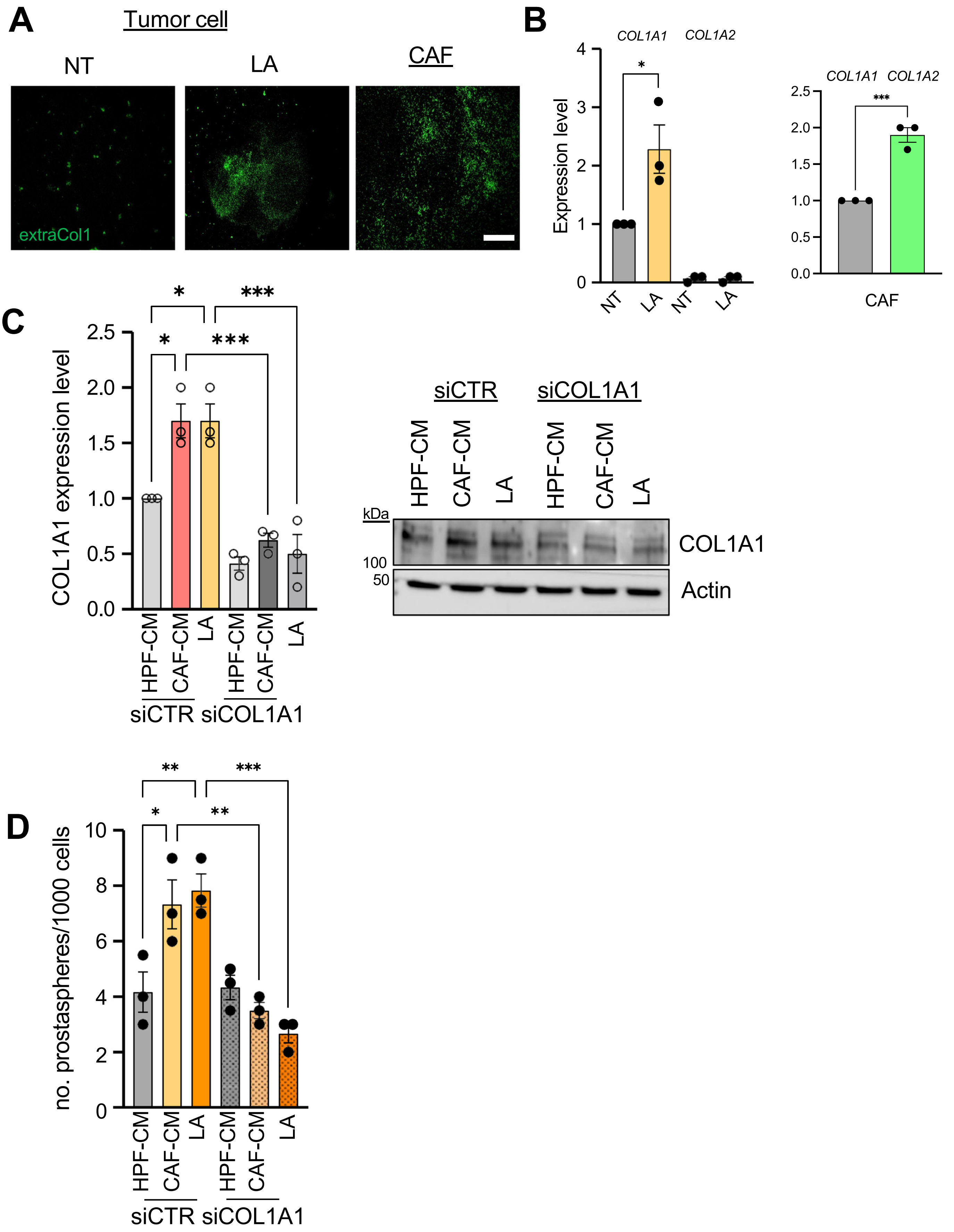
A
Tumor cell
NT
LA
CAF
extraCol1
B
COL1A1
COL1A2
Expression level
NT
LA
NT
LA
CAF
C
COL1A1 expression level
HPF-CM
CAF-CM
LA
siCTR
siCOL1A1
kDa
100
50
COL1A1
Actin
D
no. prostaspheres/1000 cells
HPF-CM
CAF-CM
LA
siCTR
siCOL1A1

◀ **Figure EV5. Tumor-derived collagen boosts LA-induced aggressive traits in PCa cells.**

(**A**) Immunofluorescence of extracellular collagen I on decellularized matrices derived from tumor cells, treated with LA for 4 days or not (NT), and from CAFs. (**B**) qRT-PCR analysis for COL1A1 and COL1A2 in PC3 cells treated or not with LA, and in three different CAFs (each point represents a CAF specimen). (**C**) qRT-PCR analysis for COL1A1 and western blot analysis of COL1A1 in PC3 cells, silenced for COL1A1 and treated as indicated. (**D**) The number of the prostaspheres-derived PC3 cells silenced for COL1A1, treated as indicated, was quantified and plotted. Scale bar: 100 μm. Results are shown as means ± SEM. Data information: bar graphs in (**B–D**) represent means ± SEM, $n = 3$ biological replicates. Significance was determined using one-way ANOVA, followed by Tukey's multiple comparisons test (**B–D**); *$P < 0.05$, **$P < 0.01$, ***$P < 0.001$. Source data are available online for this figure.

 © The Author(s)
